# Supplementary material for: Multiple origins of prokaryotic and eukaryotic single-stranded DNA viruses from bacterial and archaeal plasmids
Source: Nat Commun. 2019 Jul 31;10:3425. doi: 10.1038/s41467-019-11433-0 (PMC6668415; doi:10.1038/s41467-019-11433-0)
Supplement: Supplementary file 1 — Supplementary Information New [file 41467_2019_11433_MOESM1_ESM.pdf]

SUPPLEMENTARY INFORMATION

Multiple origins of prokaryotic and eukaryotic single-stranded DNA viruses from bacterial plasmids

Darius Kazlauskas[1], Arvind Varsani[2,3], Eugene V. Koonin[4], Mart Krupovic[5]*

[1] – Institute of Biotechnology, Life Sciences Center, Vilnius University, Saulėtekio av. 7, Vilnius 10257, Lithuania
[2] – The Biodesign Center for Fundamental and Applied Microbiomics, School of Life sciences, Center for Evolution and Medicine, Arizona State University, Tempe, AZ 85287, USA
[3] – Structural Biology Research Unit, Department of Integrative Biomedical Sciences, University of Cape Town, Rondebosch, 7700, Cape Town, South Africa
[4] – National Center for Biotechnology Information, National Library of Medicine. National Institutes of Health, Bethesda, MD 20894, USA
[5] – Institut Pasteur, Department of Microbiology, 25 rue du Docteur Roux, Paris 75015, France

* - correspondence
E-mail: krupovic@pasteur.fr

SUPPLEMENTARY NOTE 1
Analysis of bacterial mobile genetic elements encoding viral-like Reps

The majority of the Reps from pCRESS7 and pCRESS9, both represented in members of the phylum *Tenericutes*, are encoded by extrachromosomal plasmids (Supplementary table 1). By contrast, only 6 of the 237 (2.5%) Reps found in other groups are plasmid-borne (Supplementary table 1), with the rest being encoded in bacterial chromosomes. To characterize the provenance and potential function of these Reps, we performed a detailed genomic context analysis of selected genes from each Rep group. In the majority of cases, when the Reps were encoded on sufficiently large genomic contigs, the *rep* gene was located in the vicinity of a gene encoding an integrase of the tyrosine recombinase superfamily (Figure 2C). Further analysis showed that the loci encompassing the two genes as well as a variable number of other genes were flanked by direct repeats corresponding to the attachment sites (Supplementary figure 3a-i, Supplementary table 1), a typical feature of integration of circular dsDNA molecules mediated by tyrosine recombinases[1]. The majority of the analyzed mobile genetic elements were integrated into diverse tRNA genes. However, we also identified several elements integrated into protein-coding genes. For instance, elements encoding Reps from pCRESS2, -6 and -8 have recombined with the 3'-distal region of the gene encoding 30S ribosomal protein S9 (Supplementary table 1).

The Rep-encoding elements carry from 2 (e.g., plasmid pRGRH0065) to over 20 genes (e.g., element LacLac-E1; Supplementary figure 3). Despite this variability, based on the shared content of signature genes, the elements could be assigned to one of three families: (i) elements from Rep-based pCRESS1-3, -5, -8 and certain members of pCRESS6 share genes encoding FtsK-like DNA segregation ATPases (cl28087), single-stranded DNA binding (SSB) proteins, and occasionally, Sec10/PgrA surface exclusion domain protein, which specifically inhibits the ability of cells to uptake homologous plasmids (IPR027607); (ii) elements from pCRESS4, -6 and -7 encode a conserved MOB_V-family plasmid mobilization protein (PF01076); (iii) all elements carried by phytoplasma, irrespective of their placement in the Rep-based phylogeny, encode plasmid copy number control proteins [2], a distinct SSB protein (PRK06752), and a conserved protein of unknown function (Figure 2C). Notably, none of the elements encoded any homologs of currently known viral structural proteins. Such pattern of gene sharing and incongruence with the Rep-based grouping (Figure 1) is consistent with the recombination and horizontal spread of these elements between different bacterial species. The latter conclusion is supported by phylogenetic analysis of the Rep sequences from each of the 9 pCRESS groups (Supplementary figure 3a-i). A notable case of horizontal plasmid transfer is observed in pCRESS7, where two elements found in alpha-proteobacteria and gamma-proteobacteria are nested among elements from *Clostridia*. Collectively, these observations indicate that viral-like Reps in bacteria are encoded by diverse extrachromosomal and integrated plasmids.

Sequence motifs shared by bacterial and CRESS-DNA virus Reps

A detailed comparison of the conserved motifs in the nuclease and helicase domains of the viral-like bacterial and CRESS-DNA virus Reps (Figure 3) supports the inferences made from the clustering analysis (Figure 1). An asparagine in motif C of the helicase domain, which interacts with the γ-phosphate of ATP and a nucleophilic water molecule[3], is conserved across all known CRESS-DNA viruses as well as the bacterial Reps of pCRESS2 and pCRESS3, but not in pCRESS1 or the YLxH supergroup. pCRESS3 bacterial Reps are most similar to those of smacoviruses, nanoviruses and circoviruses. Most notably, these proteins share the unique modification of the motif II, HUQ, in the nuclease domain, not found in other known prokaryotic plasmid or virus Reps. By contrast, algae-infecting bacilladnaviruses are more similar to the bacterial pCRESS2 Reps, especially within the helicase domain, where the conserved aspartate residues in the Walker B motif are replaced by glutamates. The uncultivated gastropod-associated circular DNA viruses (GasCSV) appear to be chimeric with respect to the nuclease and helicase domains. Whereas the former is more similar to the nuclease domain of circoviruses, the latter shows the highest similarity to the helicase domain of pCRESS2 bacterial Reps. A similar recombination hotspot between the two domains has been previously observed in many uncultivated CRESS-DNA viruses [4,5]. Sequence motifs of pCRESS9 and *P. pulchra* plasmid Reps are most closely similar to those of geminiviruses and genomoviruses. Furthermore, all Rep sequences in this assemblage share the GRS motif located between motifs II and III (Figure 3), which is not found in other CRESS-DNA viruses and is

thought to enable the appropriate spatial arrangement of motifs II and III [6,7]. Another synapomorphic character shared by geminiviruses, genomoviruses and pCRESS9 plasmids is the replacement of the arginine finger, which is conserved in the helicase domain of other CRESS-DNA viruses[8], with an asparagine residue. Notably, in *P. pulchra* plasmids, the arginine finger motif is well-conserved, suggesting an ancestral position of this group with respect to geminiviruses, genomoviruses and pCRESS9.

Further phylogenetic and statistical validation of the Rep tree topology

To test the robustness of the PhyML tree, we performed the following additional analyses: (i) maximum likelihood phylogenies were constructed using other methods, namely, RAxML and IQ-Tree, with alternative branch support methods, including the classical bootstrap and the more recently introduced ultrafast bootstrap procedures; (ii) phylogeny was reconstructed using the 20-profile mixture model which, similar to Bayesian CAT models but in the maximum likelihood framework, allows 20 substitution models along the sequences in the alignment[9]; (iii) statistical analysis of the unconstrained and 3 constrained tree topologies was performed. The IQ-Tree and RAxML trees had topologies nearly identical to the topology of the PhyML tree, although the branch support values estimated with the bootstrap procedure in RAxML tree were slightly lower than the aBayes and ultrafast bootstrap values for the PhyML and IQ-Tree trees, respectively. To account for potential differences in site-specific amino acid replacement patterns, we used the C20 mixture model, which yielded a topology nearly identical to that in the single-model maximum likelihood analyses (Figure 5 and Figure S5). To further scrutinize the robustness of the phylogenetic tree, we constructed a set of constrained trees with alternative topologies and compared these to the unconstrained tree using several statistical tests, including the approximately unbiased test[10]. All tests rejected the trees with alternative topologies (Supplementary table 2). Collectively, these results indicate that the obtained tree topology is highly robust and is likely to accurately reflect the evolutionary history of Reps encoded by CRESS-DNA viruses and plasmids.

SUPPLEMENTARY TABLES

Supplementary table 1. Characterization of the integrated and extrachromosomal plasmids from pCRESS1-9, including information on their size, integration coordinates, integration targets, size of the attachment sites.

Organism	Plasmid/ Element	Accession number	Coordinates	Integration target	att length	RC-Rep accession
**pCRESS1**						
Firmicutes; Clostridia; Clostridiales						
Butyrivibrio sp. MB2005	ButMB-E1	NZ_AUJP01000012	6964..14931	tRNA-Gly	33	WP_026524352.1
Firmicutes; Bacilli; Lactobacillales						
Streptococcus anginosus DSM 20563	StrAng-E1	NZ_KB891980	77855..88340	Intergenic	24	WP_003030931.1
Streptococcus iniae YSFST01-82	StrIni-E1	NZ_CP010783	1794043..1799231	YbaB-like protein	13	WP_003102166.1
Streptococcus oralis ATCC 35037	StrOra-E1	NZ_AEDW01000014	134347..143668	Intergenic	16	WP_000032131.1
Streptococcus suis 10581	StrSui-E1	NZ_ALKQ01000044	47978..60499	FanG protien	88	WP_029694263.1
**pCRESS2**						
Firmicutes; Clostridia; Clostridiales						
Clostridium celerecrescens 152B c7	CloCel-E1	NZ_JPME01000007	145850..156771	tRNA-Gly	24	WP_038278663.1
Clostridium citroniae WAL-17108	CloCit-E1	NZ_JH376425	170718..179404	tRNA-Gly	20	WP_007865724.1
Clostridium methoxybenzovorans SR3	CloMet-E1	NZ_ATXD01000006	2200538..2210845	tRNA-Gly	20	WP_024346025.1
Clostridium viride DSM 6836	CloVir-E1	NZ_JHZO01000006	39765..67907	30S ribosomal protein S9	14	WP_051546484.1
Lachnospiraceae bacterium 6 1 63FAA	LacBac6.1-E1	NZ_GL890549	384948..394690	Intergenic	32	WP_009246639.1
Roseburia inulinivorans DSM 16841	RosInu-E1	NZ_ACFY01000152	42399..50222	SAM methylase	34	WP_044928503.1
Hungatella hathewayi	HugHat-E1	CZAZ01000001	167507..174549	Intergenic	16	CUP05665.1
Ruminococcus sp. 5 1 39BFAA	Rum51-E1	ACII02000003	33727..44938	tRNA-Trp	41	EES75484.2
Ruminococcus sp. CAG 108	RumCAG-E1	NZ_HF987453	47135..59333	tRNA-Trp	45	WP_021882760.1
Firmicutes; Erysipelotrichia; Erysipelotrichales						
Erysipelothrix rhusiopathiae str. Fujisawa	EryRhu-E1	AP012027	1352967..1361071	GMP synthase	15	BAK32345.1
Solobacterium moorei DSM 22971	SolMoo-E1	NZ_AUKY01000086	6149..9424	tRNA-Ala	15	WP_037404274.1
Tenericutes; Mollicutes						
Mollicutes bacterium HR2	MolHR2-E1	NZ_JRFF01000028	15562..27894	tRNA-Arg	56	WP_036328238.1
**pCRESS3**						
Actinobacteria; Actinobacteria; Propionibacteriales						
Propionibacterium acnes HL005PA4	ProAcn-E1	NZ_GL383162	76237..81150	tRNA-Pro	18	WP_002529618.1
Actinobacteria; Actinobacteridae; Actinomycetales						
Mobiluncus mulieris ATCC 35243	MobMul-E1	NZ_GG668519	73683..78607	tRNA-Val	23	WP_036342632.1
Actinobacteria; Actinobacteridae; Bifidobacteriales						
Alloscardovia omnicolens DSM 21503	AllOmni-E1	NZ_ATVB01000007	45220..53134	tRNA-Ala	19	WP_022856850.1
Bifidobacterium pseudocatenulatum	p4M	NC_003527	N/A			NP_613078.1
Bifidobacterium pullorum LMG 21816	BifPul-E1	NZ_JGZJ01000010	37044..44621	tRNA-Ala	51	WP_043170238.1
Bifidobacterium longum	BifLon-E1	NZ_JNVX01000024	104187..111387	tRNA-Ala	35	WP_021975256.1
**pCRESS4**						
Firmicutes; Clostridia; Clostridiales						
Ruminococcus sp. CAG:488	RumCAG-E1	CBBI010000010	34399..48707	tRNA-Arg	50	CDA18875.1
unclassified						
uncultured prokaryote	pRGRH0065	LN852756	N/A			CRY93789.1
uncultured prokaryote	pRGFK1613	LN854124	N/A			CRY97508.1
Actinobacteria; Corynebacteriales						
Gordonia malaquae NBRC 108250	GorMal-E1	BAOP01000004	266143..277064	intergenic	36	GAC78794.1
**pCRESS5**						
Firmicutes; Bacilli; Lactobacillales						

Streptococcus suis 92-1400	StrSui-E1	NZ_ALLO01000011	691..6646	tRNA-Leu	50	WP_024399566.1
Streptococcus sp. SR1	StrSR1-E1	NZ_JATR01000010	946..12807	tRNA-Leu	15	WP_033583888.1
Streptococcus oralis strain 727_SORA 19_27736_643863	StrOra-E1	NZ_JUVM01000007	17067..27612	tRNA-Leu	13	WP_049478725.1
Lactococcus garvieae I113	LacGar-E1	NZ_AMFD01000003	12360..23367	Intergenic	31	WP_017368666.1
**pCRESS6**						
Firmicutes; Bacilli; Lactobacillales						
Enterococcus faecium strain LMG 8148	EntFae-E1	NZ_LOHT01000285	29084..38080	30S ribosomal protein S9	25	WP_061343647.1
Lactococcus lactis subsp. lactis	LacLac-E1	NZ_JNLP01000001	1815245..1831903	tRNA-Leu	50	WP_032941943.1
Lactococcus lactis subsp. cremoris A76	pQA504	CP003136	N/A			AEU41945.1
Streptococcus suis YS39	StrSuiYS39-E1	NZ_ALMO01000016	34857..43776	Intergenic	22	WP_024400359.1
Firmicutes; Clostridia; Clostridiales						
Butyrivibrio sp. XPD2006 G590	ButXPD-E1	NZ_ATVT01000008	119846..125578	tRNA-Leu	20	WP_022765681.1
**pCRESS7**						
Firmicutes; Clostridia; Clostridiales						
Clostridium bolteae 90B3	CloBol-E1	NZ_KB851181	29144..34918	Intergenic	21	WP_002578150.1
Proteobacteria; Gammaproteobacteria; Vibrionales						
Vibrio anguillarum RV22	VibAng-E1	NZ_AEZB01000083	1220..7071	Intergenic	13	WP_019282500.1
Tenericutes; Mollicutes; Acholeplasmatales						
Aster yellows witches'-broom phytoplasma AYWB	pAYWB-II	CP000063	N/A			ABC65794.1
Aster yellows witches'-broom phytoplasma AYWB	pAYWB-IV	CP000065	N/A			ABC65805.1
Ca. Phytoplasma australiense	pCPa	NC_010918	N/A			YP_001966814.1
Onion yellows phytoplasma OY-M	OniYel-E1	NC_005303	756287..761444	Intergenic	46	WP_011161011.1
Periwinkle leaf yellowing phytoplasma	p09PLY-1	NC_019244	N/A			YP_006961027.1
Periwinkle leaf yellowing phytoplasma	p09PLY-2	NC_019247	N/A			YP_006961042.1
Rehmannia glutinosa' phytoplasma	pPARG1	NC_014123	N/A			YP_003617079.1
Proteobacteria; Alphaproteobacteria; Rhodospirillale						
Acidiphilium sp. CAG:727	AciCAG-E1	FR898463	2638..14917	Intergenic	16	CDE19587.1
**pCRESS8**						
Firmicutes; Bacilli; Lactobacillales						
Lactobacillus mucosae LM1	LacMuc-E1	NZ_CP011013	2081576..2091749	tRNA-Thr	102	WP_006499656.1
Lactobacillus reuteri 100-23	LacReu-E1	NZ_AAPZ02000001	1366292..1380782	tRNA-Thr	23	WP_003665528.1
Leuconostoc mesenteroides subsp. cremoris ATCC 19254	LeuMes-E1	NZ_GG693387	185148..195379	Ribosomal protein S9	18	WP_036093565.1
Oenococcus oeni DSM 20252	OenOen-E1	NZ_AQVA01000012	15290..30347	tRNA-Leu	18	WP_002821392.1
Streptococcus mitis SK629	StrMit-E1	NZ_JPFU01000002	82438..92128	Intergenic	24	WP_042900192.1
Lactobacillus taiwanensis DSM 21401	LacTai-E1	AYZG01000001	4476..14624	30S ribosomal protein S9	21	KRN00682.1
Lactobacillus amylovorus DSM 16698	LacAmy-E1	NZ_JQBQ01000011	3735..15652	tRNA-Thr	14	WP_056985318.1
Enterococcus faecalis F01966	EntFae-E1	NZ_KE351541	26540..37054	30S ribosomal protein S9	51	WP_016622553.1
Lactobacillus vaginalis	pC107	KP172590	N/A			AKG47101.1
Firmicutes; Bacilli; Bacillales						
Staphylococcus epidermidis	pSAP110B	NC_013384	N/A			YP_006939186.1
Viridibacillus arenosi FSL R5-213	VirAre-E1	NZ_ASQA01000035	81188..94595	Intergenic	26	WP_051448806.1
**pCRESS9**						
Tenericutes; Mollicutes; Acholeplasmatales						
Onion yellows phytoplasma	pEcOYNIM	NC_019167	N/A			YP_006959585.1
Paulownia witches'-broom phytoplasma	pPaWBNy-1	NC_010405	N/A			YP_001708784.1
Paulownia witches'-broom phytoplasma	pPaWBNy-2	NC_010406	N/A			YP_001708790.1
Periwinkle little leaf phytoplasma	pPLLHn-1	NC_019290	N/A			YP_006961991.1
Wheat blue dwarf phytoplasma	pWBD1	NC_019535	N/A			YP_007008175.1

Wheat blue dwarf phytoplasma	pWBD3	NC_019536	N/A	YP_007008179.1
Candidatus Phytoplasma australiense	pPAPh2	NC_010854	N/A	YP_001965305.1
Candidatus Phytoplasma australiense	pPASb11	NC_010856	N/A	YP_001965310.1
Aster yellows witches'-broom phytoplasma AYWB	pAYWB-I	NC_007717	N/A	WP_011412950.1
**P. pulchra-like**				
Eukaryota; Rhodophyta; Bangiophyceae; Bangiales				
Pyropia pulchra	plasmid	AF106327	N/A	AAF36423.1
Pyropia pulchra	plasmid	AF106328	N/A	AAF36424.1
Pyropia pulchra	plasmid	AF106326	N/A	AAF36422.1

Supplementary table 2. Topology testing for the phylogenetic tree of Rep proteins.

Tree	AU[1]	deltaL[2]	RELL[3]	KH[4]	SH[5]	WKH[6]	WSH[7]	ELW[8]
1 (Unc.)	0.998	0	0.998	0.998	1	0.998	1	0.998
2	0.00291	254.27	0.00212	0.00233	0.00375	0.00233	0.00618	0.00214
3	0.000864	287.83	0.0003	0.00046	0.00057	0.00046	0.00154	0.000308
4	4.47E-15	569.37	0	0	0	0	0	1.93E-61

Tree 1: Unconstrained topology;
Tree 2: plasmids and viruses form two monophyletic groups;
Tree 3: positions of the geminivirus/genomovirus clade and that including all other CRESS-DNA viruses are switched;
Tree 4: regrouped according to the host organisms, i.e., branch including plant-associated geminiviruses and genomoviruses is moved as a sister group to plant-associated nanoviruses/alphasatellites, animal-associated smacoviruses are grouped with circoviruses, whereas other unclassified groups of CRESS-DNA viruses are monophyletic.

1 p-value of approximately unbiased (AU) test.
2 logL difference from the maximal logl in the set.
3 bootstrap proportion using RELL method.
4 p-value of one sided Kishino-Hasegawa test.
5 p-value of Shimodaira-Hasegawa test.
6 p-value of weighted KH test.
7 p-value of weighted SH test.
8 Expected Likelihood Weight.

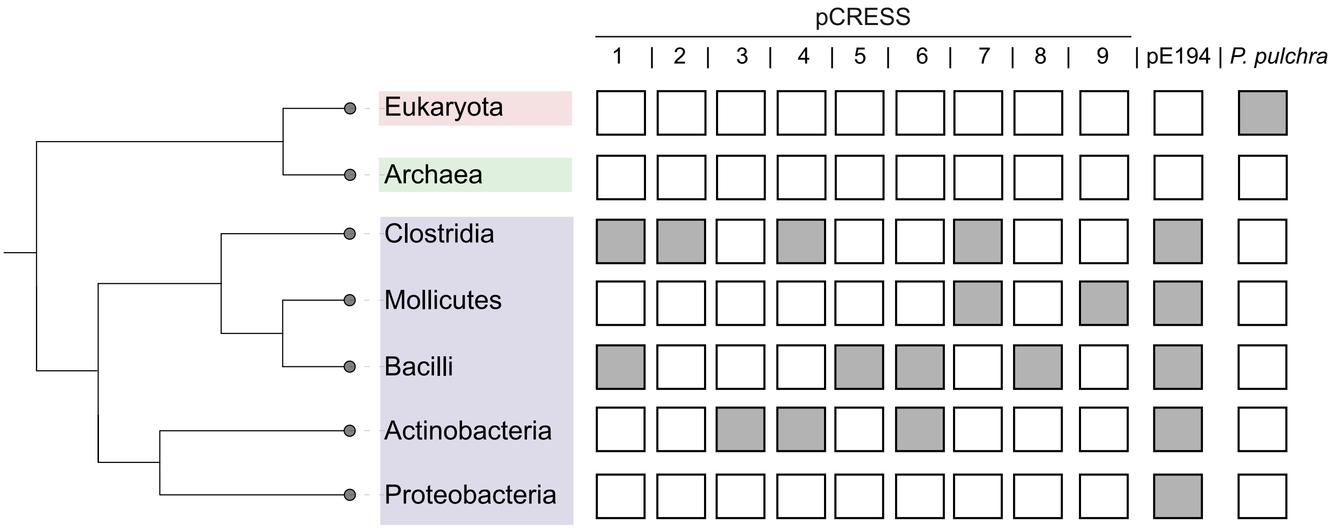

Supplementary figure 1. Taxonomic distribution of bacterial Rep proteins and their homologs. Tree of life was adopted from iTOL (https://itol.embl.de/). To get more robust view of taxonomic distribution and to dismiss recent horizontal transfers, taxa containing less than three species were filtered out.

a)

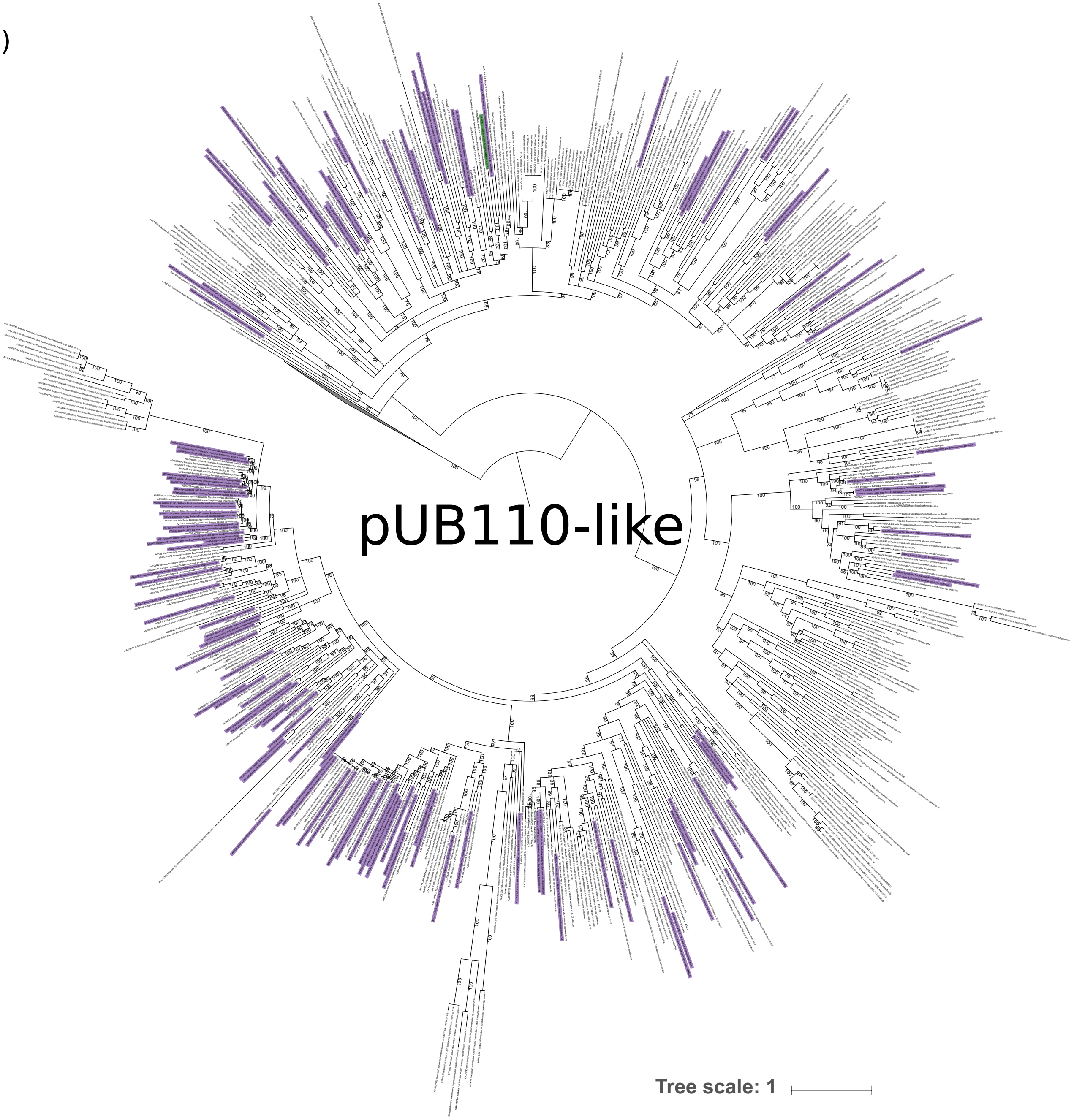

Supplementary figure 2 (continued)

b)

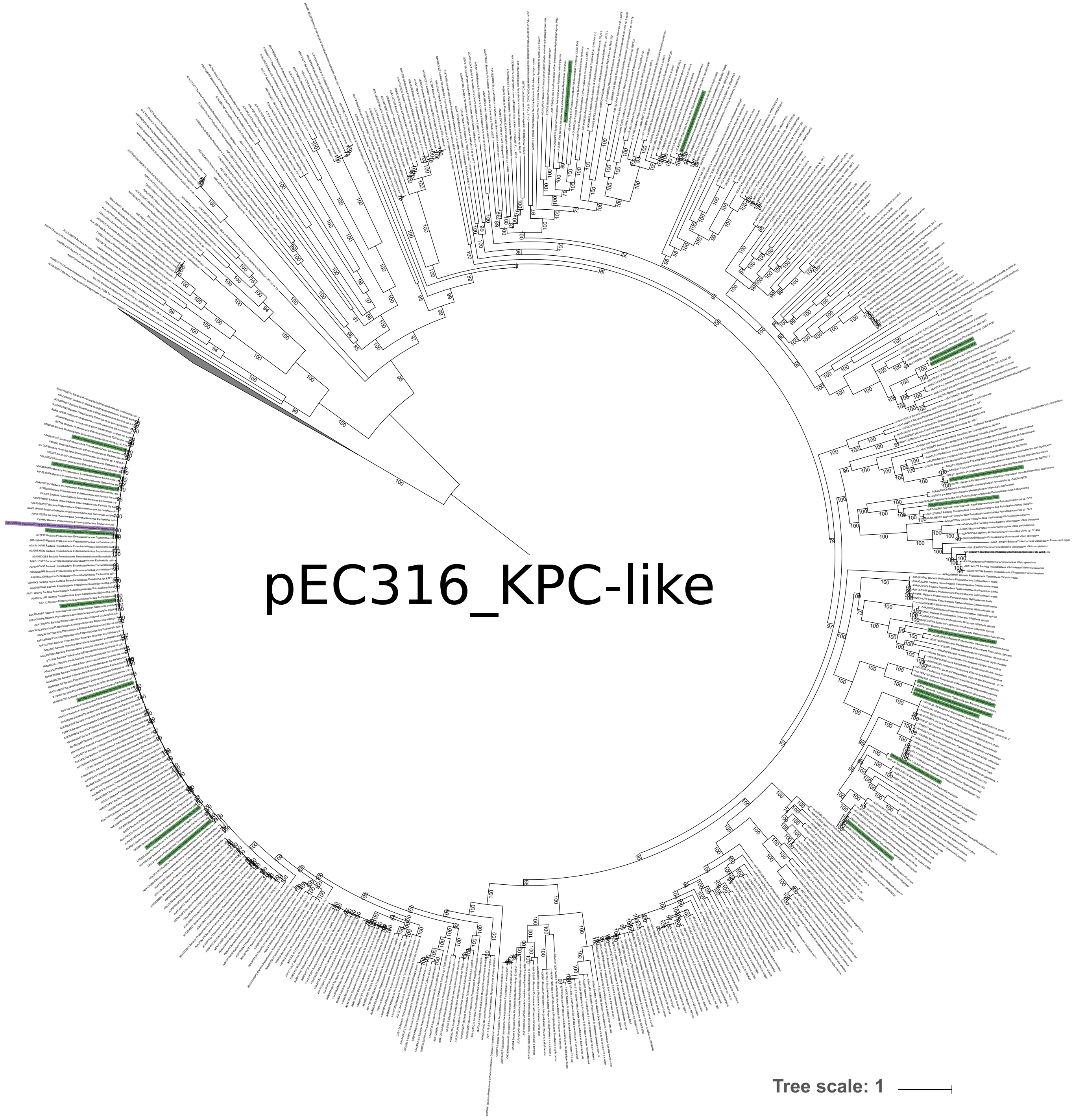

Supplementary figure 2 (continued)

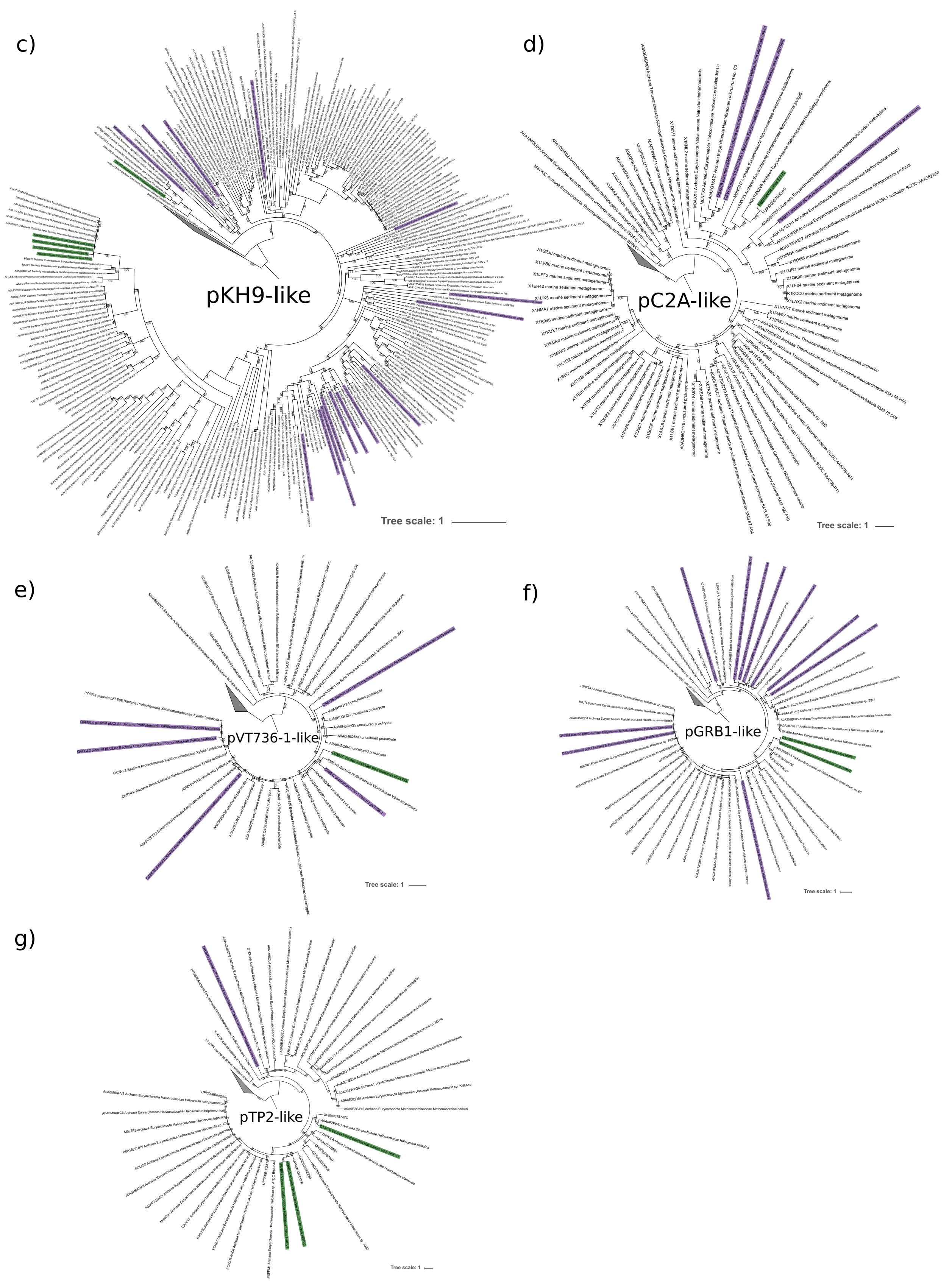

Supplementary figure 2. Maximum likelihood phylogenetic trees of Rep proteins from the 7 clusters (a-g) from supercluster 1 including both viral and plasmid sequences. Viral and plasmid sequences are highlighted on green and magenta backgrounds, respectively. All branches are labeled with the accession numbers of the sequences and taxonomy of the corresponding taxa.

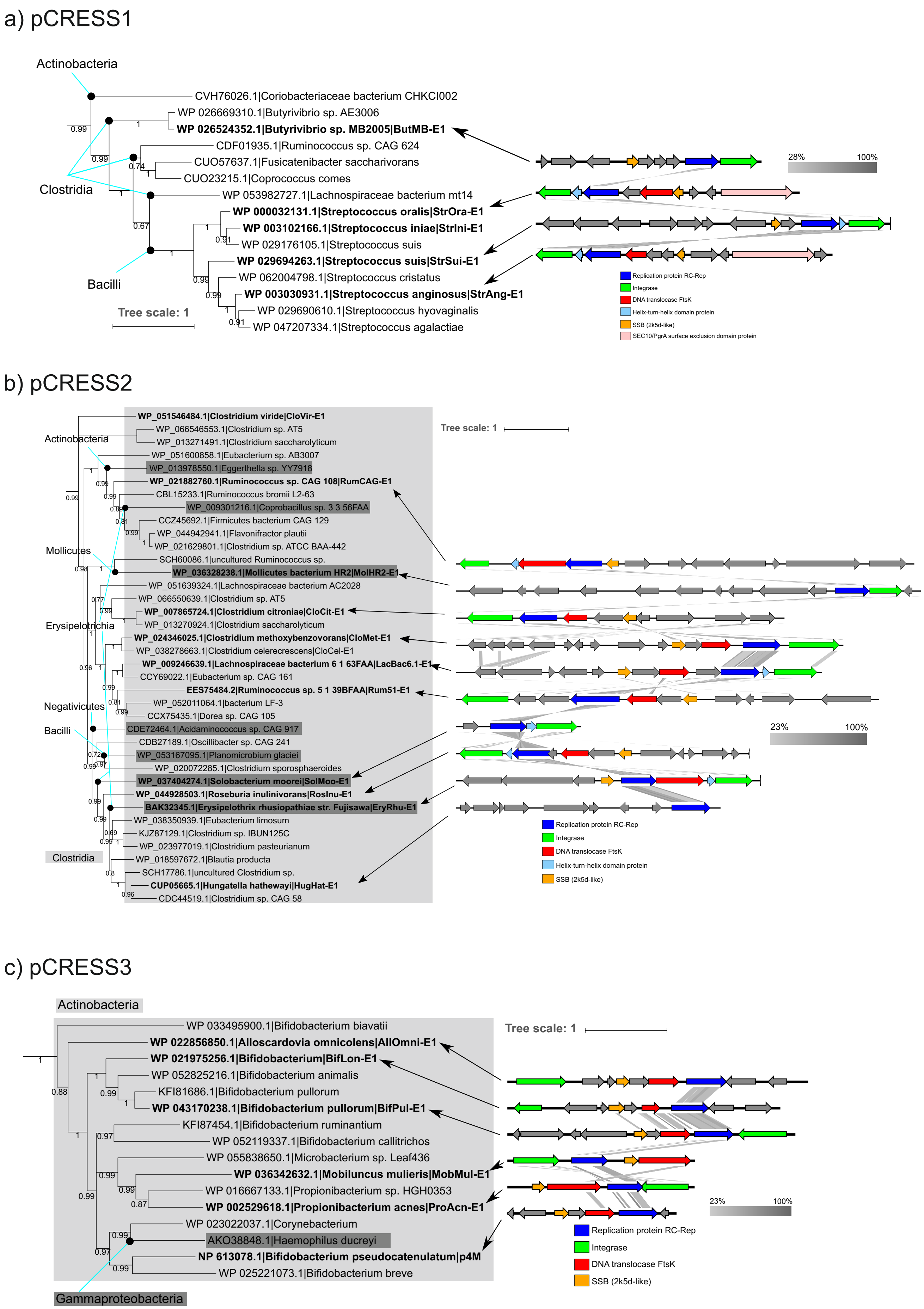

Supplementary figure 3 (continued)

d) pCRESS4

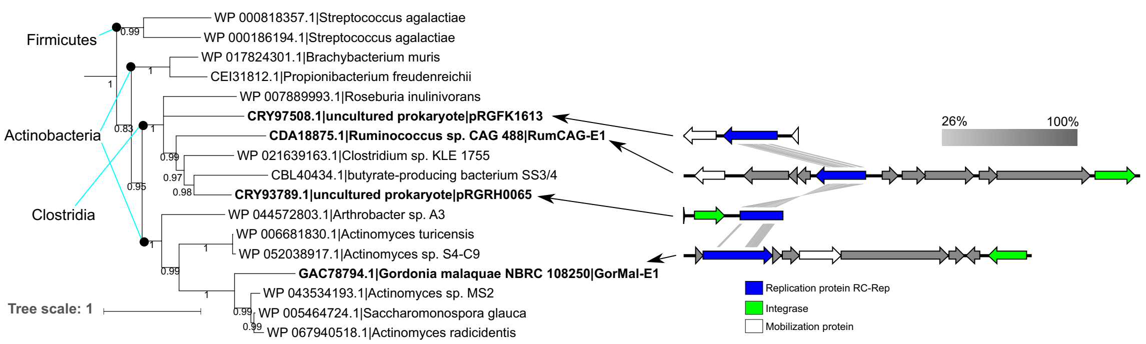

e) pCRESS5

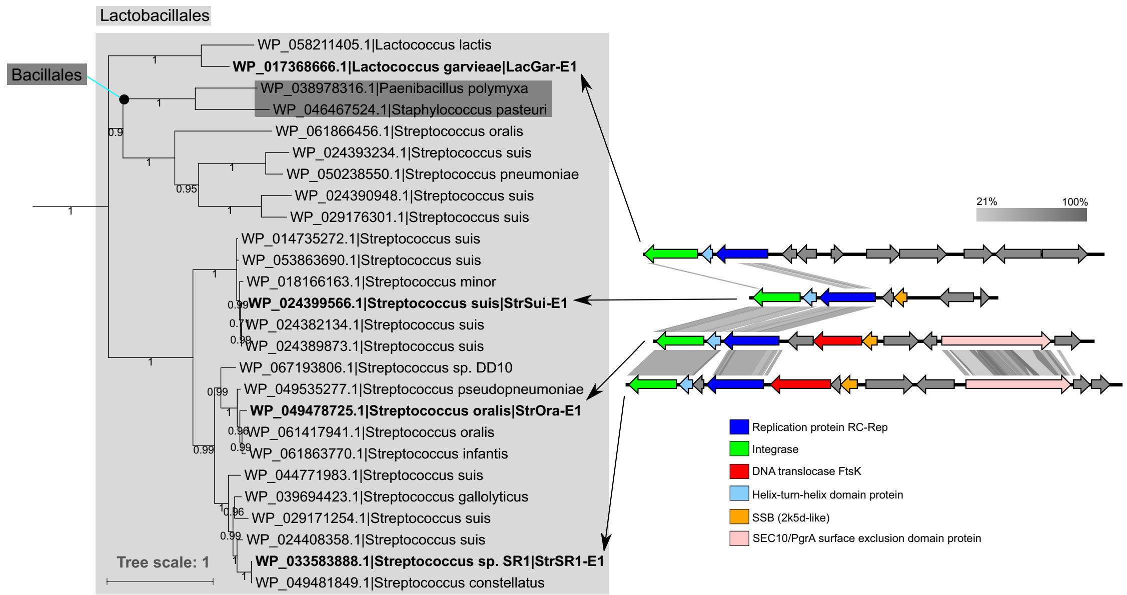

f) pCRESS6

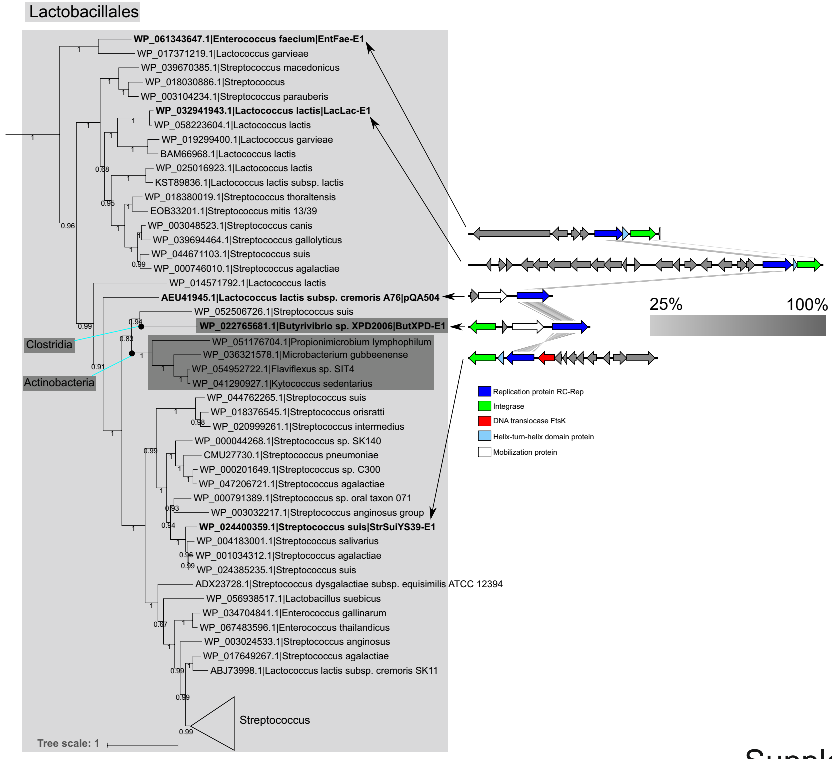

Supplementary figure 3 (continued)

g) pCRESS7

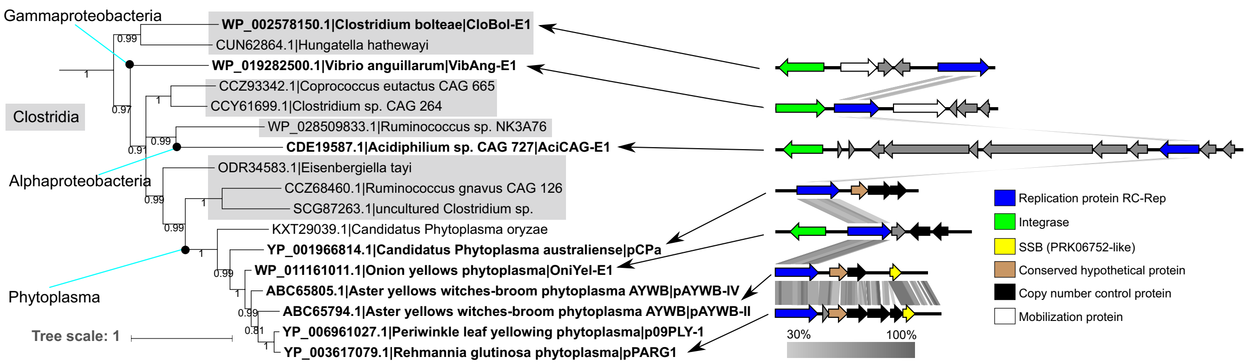

h) pCRESS8

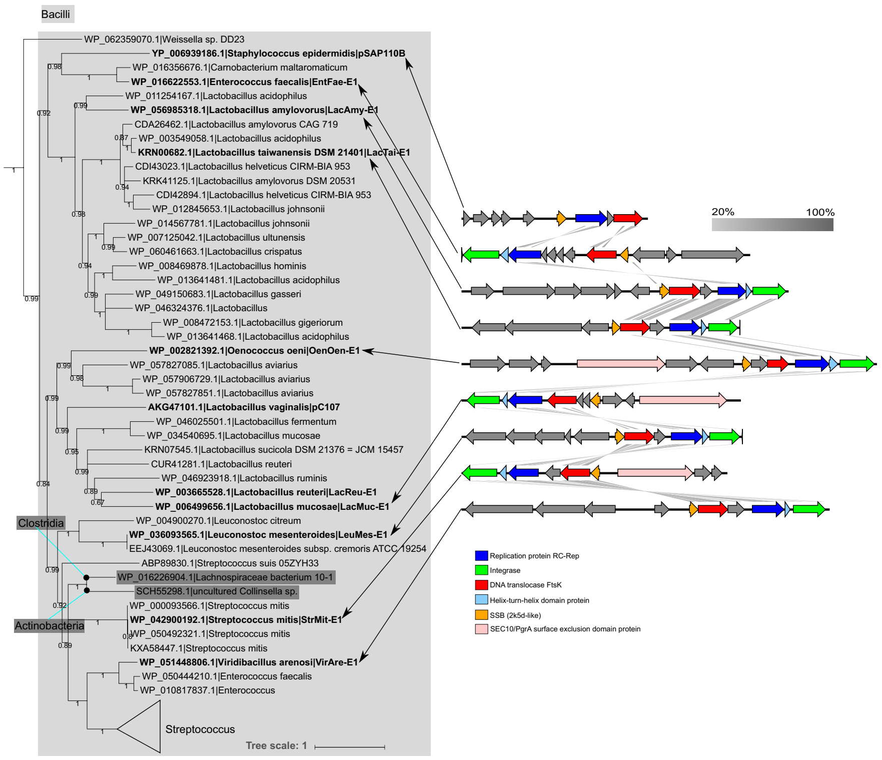

I) pCRESS9

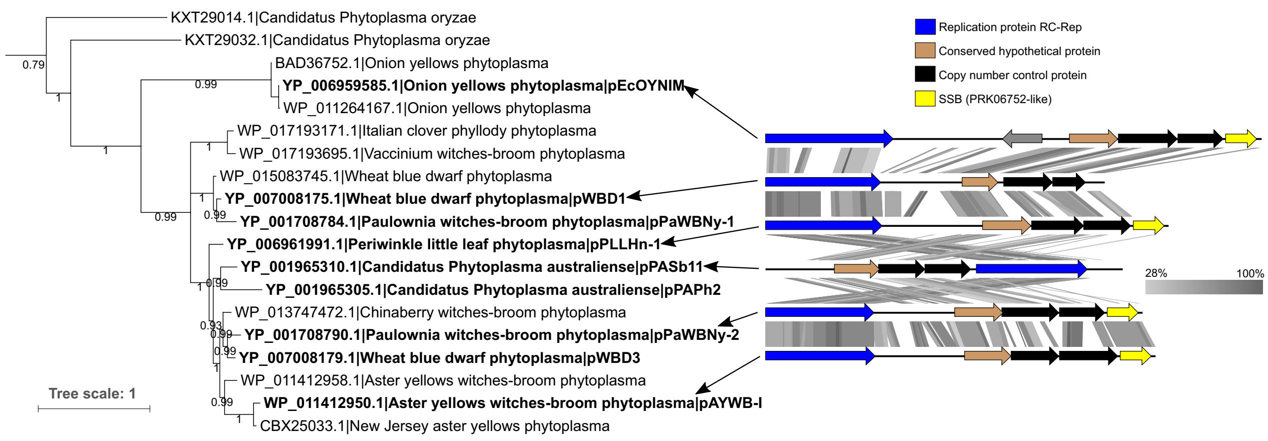

Supplementary figure 3. Phylogenetic trees and genome maps of representative integrated plasmids from pCRESS1 through pCRESS9 (a-i). Branches with support values of 65 and lower are contracted.

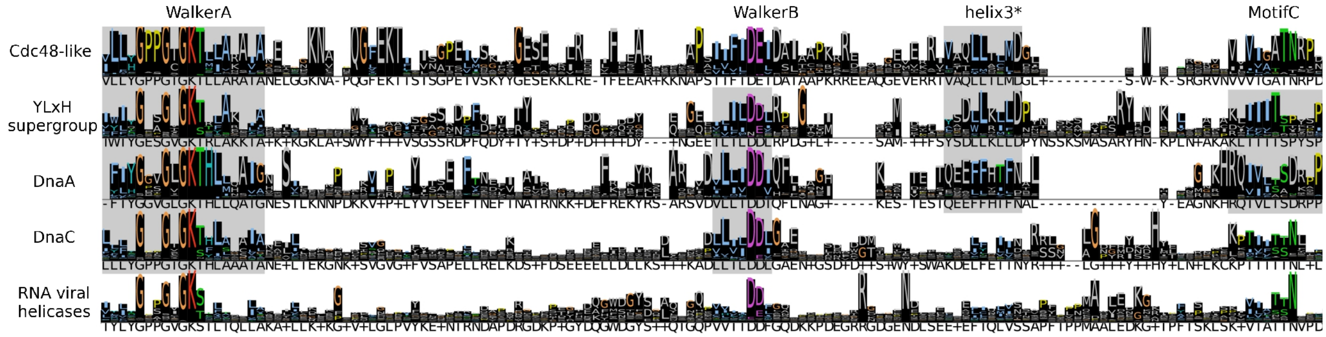

Supplementary figure 4. Comparison of the sequence motifs from AAA+ ATPases and superfamily 3 helicase domains. Similar motifs are shown in grey background.

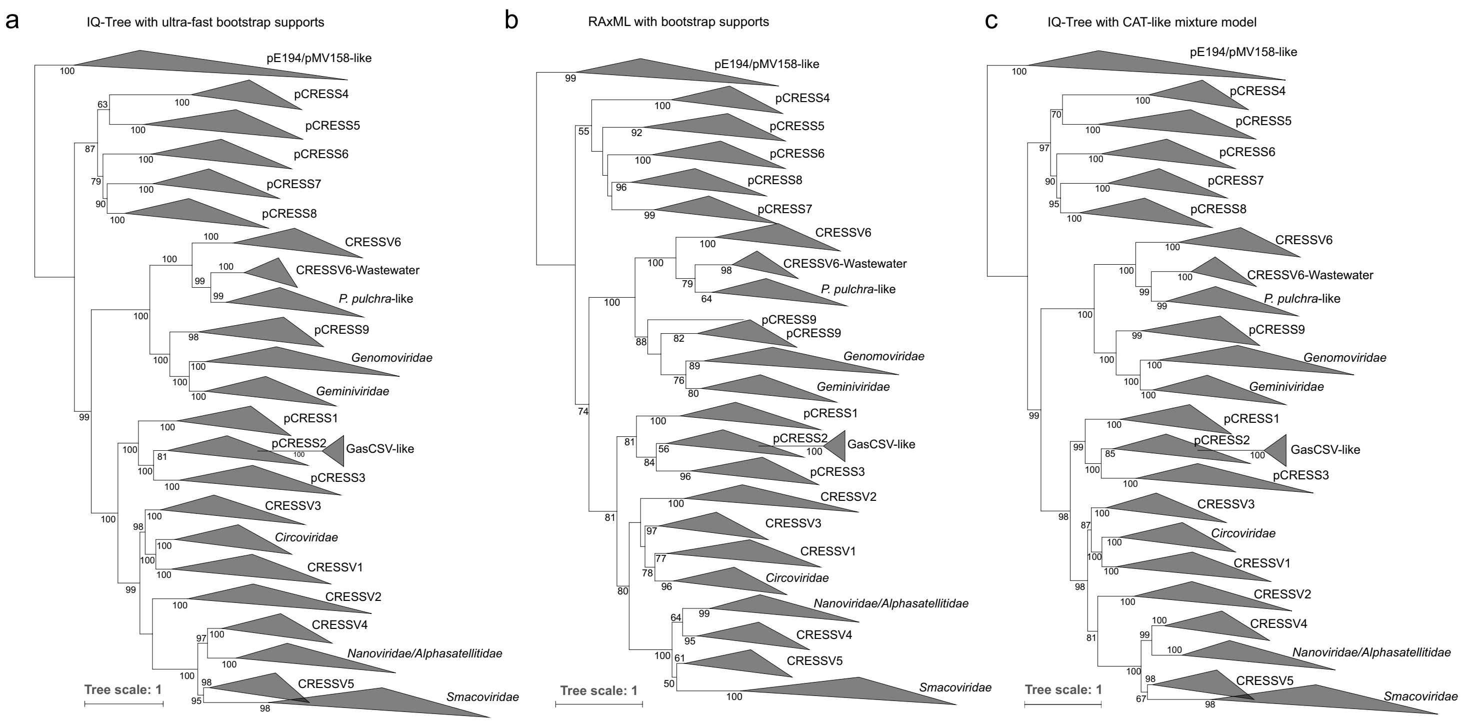

Supplementary figure 5. Maximum likelihood phylogenetic trees of Rep proteins. a) IQ-Tree phylogeny with ultra-fast bootstrap supports. b) RAxML phylogeny with bootstrap branch supports. c) IQ-Tree phylogeny reconstructed using the 20-profile mixture model (C20) which allows 20 substitution models along the sequences in the alignment.

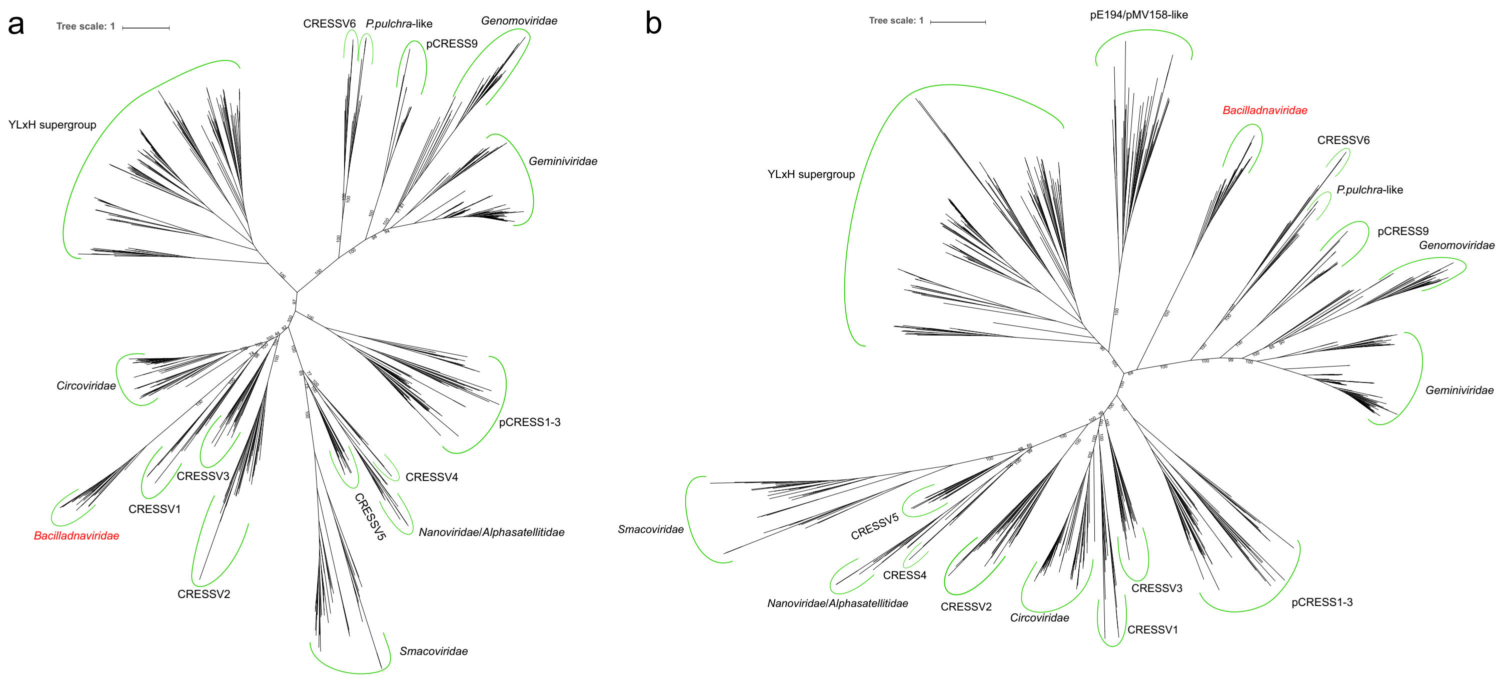

Supplementary figure 6. Maximum likelihood phylogenetic trees of Rep proteins using different sequence sampling: a) pE194/pMV158 cluster is absent; b) pE194/pMV158 cluster is present. The trees were constructed using PhyML.

SUPPLEMENTARY REFERENCES

1 Grindley, N. D., Whiteson, K. L. & Rice, P. A. Mechanisms of site-specific recombination. *Annu Rev Biochem* **75**, 567-605 (2006).

2 Chen, W., Li, Y. & Wu, Y. F. Molecular characterization and tissue specific copy number of three plasmids from wheat blue dwarf phytoplasma. *J Plant Pathol* **96**, 69-76 (2014).

3 George, B. *et al.* Mutational analysis of the helicase domain of a replication initiator protein reveals critical roles of Lys 272 of the B' motif and Lys 289 of the beta-hairpin loop in geminivirus replication. *J Gen Virol* **95**, 1591-1602 (2014).

4 Quaiser, A., Krupovic, M., Dufresne, A., Francez, A. & Roux, S. Diversity and comparative genomics of chimeric viruses in Sphagnum-dominated peatlands. *Virus Evol* **2**, vew025 (2016).

5 Kazlauskas, D., Varsani, A. & Krupovic, M. Pervasive chimerism in the replication-associated proteins of uncultured single-stranded DNA viruses. *Viruses* **10**, E187 (2018).

6 Nash, T. E. *et al.* Functional analysis of a novel motif conserved across geminivirus Rep proteins. *J Virol* **85**, 1182-1192 (2011).

7 Varsani, A. & Krupovic, M. Sequence-based taxonomic framework for the classification of uncultured single-stranded DNA viruses of the family Genomoviridae. *Virus Evol* **3**, vew037 (2017).

8 Kazlauskas, D. *et al.* Evolutionary history of ssDNA bacilladnaviruses features horizontal acquisition of the capsid gene from ssRNA nodaviruses. *Virology* **504**, 114-121 (2017).

9 Quang le, S., Gascuel, O. & Lartillot, N. Empirical profile mixture models for phylogenetic reconstruction. *Bioinformatics* **24**, 2317-2323 (2008).

10 Shimodaira, H. An approximately unbiased test of phylogenetic tree selection. *Systematic biology* **51**, 492-508 (2002).
