# Supplementary material for: Multiple origins of prokaryotic and eukaryotic single-stranded DNA viruses from bacterial and archaeal plasmids
Source: Nat Commun. 2019 Jul 31;10:3425. doi: 10.1038/s41467-019-11433-0 (PMC6668415; doi:10.1038/s41467-019-11433-0)
Supplement: Supplementary file 3 — Dataset 2 [file 41467_2019_11433_MOESM3_ESM.docx]

**SUPPLEMENTARY DATA 2**

# ALIGNEMNT USED TO PRODUCE THE TREE SHOWN IN FIGURE 2A

>pCRESS6|WP_034704841.1

M---KKQA------------------------------------SLTCVMIVQQLKEE---------FWFQW

ED-TIITEAENG-NIQPLMEELNKRFHNR--G--YE-VNELYGIIHDKDERLVWNQEQMKN-V---IE--P-

KEKHAHILVKF--AKGDTLNNLSVTAG-----VDP-QYIE--KAK----SG--R----YGYDNLLSYLVHAK

----------------D----QD--KYQYSPDEVV-TVSG-----EE----YTSVYNRRMETWVRGRATKEA

KA--TDLS-VD-YLVSEILDGKL-TKSQ----VLLTNEF-------YKVYA--LHKRKINDAFDTAGENKSY

QTIADLDAGNF-KKTILFIMAESGAGKTVLSKKIISIL-Q-SVALKQTEQ--RW-DYCLTAS-NN----AFD

EY-NGQDVLFLDDIRG---DS-L----SVSDWLKLLDPYTI-SP-ISARYHNKMGSA-K---VIIITSTKTP

SEFFS--IA---KSN-----------FH-EDLGQFFRRIDLLISI----E-D-----------------DKF

HLSKFEKAPPASTQ-FPYIMKPP---SHYFRFDGT-Y-YKN--QALDKVT-KVTIRNMQWNKK----K----

SVTKA--RKSKKVTDH-----NK-------------------------------------------------

-----------------------

>pCRESS6|WP_067483596.1

M---KKQA------------------------------------NLSCVMLVQQLEPE---------FWIGW

DD-VIIEEAHNG-NIRPLIEELVKRFEKD--G--CE-VNEAYGILHDKDLISVWNQDEMKN-V---EE--L-

KAKHVHILIKF--GKGDTLNSLAVKAG-----VAP-QYLE--KAK----SG--R----YGYDNLLSYLVHAK

----------------D----QD--KHQYFPDEVV-TVSG-----EE----YTSVYNRRMETWVRGRATKEA

KA--TDLS-VD-YLVSEILAGKL-TKSQ----VLLTNEF-------YRVYA--LHKRKINDAFDTAGESKSY

QTIADLDAGNF-KKTTLFIMAESGAGKTVLSKKIISIL-Q-SVALKKMEQ--RW-DYCLTAS-NN----AFD

EY-NGQDIIFLDDIRG---DS-L----SVSDWLKLLDPYTI-SP-ISARYHNKMGSA-K---AIIITSTKTP

SEFFS--IA---KGN-----------FH-EDLGQFFRRIDLLINI----E-D-----------------DKF

HLSKPEKAPPASTQ-FPYVIKPP---SHYFHFDGT-Y-HKN--KALDMVT-KTVIRNMQLNKK----K----

PVTQP--AKA-NVTDH----LKK-------------------------------------------------

-----------------------

>pCRESS6|WP_049499636.1

M--RKKEA------------------------------------NLTTIMLVQQLEEK---------YWLSP

DYKMPVRQAKNG-DCLPLLKMIVNNLESN--N--II-VKEAYIIKHDKDKISTWDANQKKN-V---VQ--D-

KAVHIHALLKF--ERGASLSKIALAIR-----VEP-QYLE--KMK----SG--R----YGYDNCLAYLVHAK

----------------D----ES--KHQYQPDEVV-SLRG-----ED----YTSIYQRSIEMWVKGRATKKA

KE--TNFS-ID-WLIEQVLDGKL-TKSN----IMLTDEY-------YEIYG--QHKRKVNEALDTAGERRSY

RTIAELEAALF-KKTVIFIQANSGVGKTKFSKELISAI-Q-AIAVK-NGL--NW-ETCVTAS-TN----AFD

EY-NGQEILFLDDIKG---DS-F----TVSDWLKLLDPYTI-SP-ISARYHNKMGSA-R---VIIITNTKLP

VELFY--FA---KNN-----------YN-EDLGQFIRRIDLLVHI----H-D-----------------DIF

HVCPHEKREEESTI-IAFGTNIRVRHSYTFQKSHA-I-QRN--EALEEIL-ETVMTNMNWNKA----K----

KVITA--SDQTNNDNLK--TQQK-------------------------------------------------

-----------------------

>pCRESS6|WP_045759092.1

M--GKKEA------------------------------------NLTAIMLVQQLEEK---------YWLSP

DYKGPVQQAKNG-DCRPLLEMIIKKLKSS--D--II-VKEAYIIRHDKDKISTWDSNLMTN-T---VE--D-

KAVHVHALLKF--EKGASLNKIALAVE-----VEP-QYLE--KLK----SG--R----YGYDNCLAYLVHAK

----------------D----ET--KYQYRPEEVT-TVLG-----ED----YTSIYNRSIETWLKGRATKKA

KE--TNLS-VD-WLVERILDGEV-TKRN----IMLTDEY-------YAIYG--QHKRKVNEALETAGERRSY

RTIAELEAGKF-KKTVIFIQASSGVGKTKLSKDLIELI-Q-ASAVK-NGL--NW-EACMTAS-TN----AFD

EY-SGQEILFLDDIKG---NS-F----TVSDWLKLLDPYTI-SP-ISARYHNKMGSA-R---VIIITSTKLP

VELFY--FA---NGN-----------HN-EDLGQFVRRIDLLVHI----H-N-----------------DIF

HVCPHEKTAEESTI-EAFGTNIRVHHSYTFQKSPA-I-QRD--EALEEIL-ETVTTNMNWNKT----K----

KVIDA--SDQTNNDNPN--NQQK-------------------------------------------------

-----------------------

>pCRESS6|KXT86702.1

MNKNKKEA------------------------------------NLTAIMVVQQLEEE---------HWLSS

DYKTPLQQAKEG-NCRPLLEMIIEKLEEQ--D--IL-VKEAYIIKHDKDKVSTWNPVEMKN-I---IE--T-

KVEHVHVLLKF--EKGASLNKLALAIQ-----VEP-QYLE--KLK----SG--R----YGYDNCLAYLCHCK

----------------D----EN--KHQYQPEEVT-TVRG-----ED----YTSIYHRSMETWTKGRATKKA

KE--TALS-VD-WLIEKILSGEV-TKSN----ILLTNEY-------YAIYG--QHKRKINEALDTAGERKSY

QTIADLEAGNF-KKTILFITAESGVGKTRYSKKLITLL-Q-QIALK-QGQ--KW-DYCVTAS-TN----AFD

EY-NGQEILFLDDIKG---DS-L----TVSDWLKLLDPHMI-SP-ISARYHNKMGSA-K---IIIITNTKEP

ISFFE--QA---KGN-----------MG-EDLGQFVRRIDYLIQI------E-----------------DKF

HLSIPIKHIPQKPN-NEVLWYEPRHYSFKFSKIGE-Y-DKN--IATNKLV-KQVIRNMQWNKY----K----

KVINA--SDQTTNDNPN--TQQK-------------------------------------------------

-----------------------

>pCRESS6|KEQ49321.1

MNKNKKEA------------------------------------NLTAIMLVQQLENE---------HWKNW

QDKTALKQARDS-NIRPLLETVTDKLNKA--D--IT-VKEAYGIKHDKDEINVWNQKLMKN-V---IE--K-

KAEHIHFLFKF--EKGASLNRIALAVG-----VEP-QYLE--KLK----SG--R----YGYDNCLAYLVHAK

----------------D----ET--KYQYQPEEVA-TVLG-----ED----YKSIYHRNMATWVKGRATKKA

KE--TSLS-VD-WLIEKILVGEV-TKRN----IMLTNEY-------YAIYG--QHKRKINEALDTAGERKSY

QTIDDLESGEF-KKTILFIKAESGVGKTLYSKKLITLV-Q-NIALR-FGK--RW-DYCITAS-TN----AFD

EY-NGQEILFLDDIRG---DS-L----NVSDWLKLLDPYTI-SP-VSARYHNKMGAA-K---LIIITSTKEP

LSLFK--QS---KGN-----------TG-EDLGQFVRRIDYLVEI------T-----------------DTF

RLAVPIKNLPTSET-IQHSWHESPHHSFIFSKFSC-N-SIN--EATNKIV-KRVIQNMQWNKK----K----

KVINA--SDQTNNDNPG--TKQK-------------------------------------------------

-----------------------

>pCRESS6|WP_014623544.1

M--AKKEA------------------------------------TLTAIMVVQQLEDE---------FWLSS

DYQEPLQQAKDG-DCRPLLELIIKNLESN--D--II-VKEGYIIKHDKDKVSVWDSEKMKN-I---IE--N-

KTEHIHALLKF--EKGASLTKIALAIG-----VEP-QFLE--KLK----SG--R----YGYDNCLAYLVHAK

----------------D----ES--KHQYQPDEVV-TVKG-----ED----YTSVYHRSMETWVKGRATKKA

KE--TNLS-VD-WLIEKILSGEI-TKSN----IMLTDDY-------YAIYG--QHKRKINEALDTAGERKSY

RTVAELESGKF-KKTVLFITAESGVGKTHYSKQLIALL-Q-NIALK-HGQ--IW-DYCVTAS-TN----AFD

EY-NGQEILFLDDVKG---NS-L----TVSDWLKLLDPYMI-SP-ISARYHNKMGSA-K---VIIITNTKEP

IRFFE--QA---KGN-----------IE-EDLGQFIRRTDYLIEI------S-----------------DSF

KLYVPIKHTPSYQT-IPHPWYEPPHYSYKYSKTNE-L-PIN--LATDKLI-KKVIQNMQWSKQ----K----

KVINA--TDQSNKDNPN--NQHK-------------------------------------------------

-----------------------

>pCRESS6|WP_049476139.1

M--VKKEA------------------------------------NLTAIMLVQQLEDT---------YWEQW

EDKTFIKLARDG-DCKPLLQAVVDKLNDD--N--IS-VKEAYGIKHDKDEITIWNQEEMKN-A---TE--K-

KAEHVHFLFKF--DKGASISKIALAVG-----VEP-QYLE--KLK----SG--R----YGYDNCLAYLVHAK

----------------D----ET--KFQYSPEKVV-TLLG-----ED----YVSIYNRSMETWMKGRATKKA

RE--TSLS-LD-WLIEKILSGEI-TKSN----IMLTDEY-------YAIYG--QHKRKINEAIETSGERKSY

KTISELENSEF-KKTVIFITAESGIGKTALSKQLIRIL-Q-TVAIK-FNQ--NW-DFCVTAS-TN----AFD

EY-NAQDILFLDDIRG---DS-L----TVSDWLKLLDPYMI-SP-ISARYHNKMGAA-K---VIIITSTKKP

ISFFE--SA---KGN-----------IG-EDLGQFIRRIDYLLTI------D-----------------KFF

SLSIPMKSKVMDIS-SSYPWYEPRHHSYCFSESEQ-Y-TKN--NALNHLV-KTVIRNMQWNK-----K----

ERITH--TDQSTKDTL---TQPK-------------------------------------------------

-----------------------

>pCRESS6|WP_003035134.1

M--VKKEA------------------------------------NLTAIMLVQQLEDT---------YWETW

EDKTPIKLARDG-NCKPLLQVVVDKLNNE--N--IS-VKEAYGIKHDKDEITVWNQDEMKN-V---TE--K-

KAEHIHFLFKF--EKGASLSKIALAIG-----IEP-QYLE--KLK----SG--R----YGYDNCLAYLVHAK

----------------D----ES--KFQYSPDEVV-TLLG-----ED----YLSVYNRSMETWMKGRATKKA

KE--TALS-VD-WLIEKILSGEI-TKSN----IMLTDEY-------YAIYG--QHKRKINEALDTSSERKSY

KTISELENGEF-KKTVIFITADSGIGKTTISKQLIRIL-Q-KVSLK-FGQ--NW-DTCVTAS-TN----AFD

EY-NGQDILFLDDIRG---DS-L----TVSDWLKLLDPYMI-SP-ISARYHNKMGSA-K---VIIITSTKKP

VSFFE--AS---KGN-----------IG-EDLGQFIRRIDYLLTI------N-----------------DSF

WLSLPIKSKKTDTT-SSNPWYEPRYHSYCFSEVEQ-Y-TKN--NALDHLV-KTVIRNMQWNTT----K----

KVINA--SDQTNNDNQN--IQQK-------------------------------------------------

-----------------------

>pCRESS6|WP_017649267.1

M---AKSS------------------------------------NLTAIMLVQQLEEK---------YWLFS

DYKTAIQQARDG-NCQSLLEAVVKHLEVE--N--IK-VDEAYGIIHNKDTVTVWNVQEQKN-V---DE--T-

KEDHIHFLFKF--AKGASLEKLALSIG-----VEP-QYLE--KLK----SG--R----YGYDNCQAYLVHAK

----------------D----ES--KYQYSADEVV-TVLG-----ED----YISLYNRKMESWIRGRAKKEA

QD--TNLS-ID-WIISEILAGKL-SKNQ----ILLTDDY-------YKVYG--QHKRKINEAIDTAGERKSY

KTISELEAGQF-KKTIIFINAESGVGKTAISKKLIGIL-Q-TVALK-FNQ--NW-DFCVTAS-TN----AFD

EY-NGQDILFLDDIKG---DS-L----TVSDWLKLLDPYMI-SP-ISARYHNKMGSA-K---VIIITNTKEP

MHFFE--QA---KGN-----------IG-EDLGQFVRRIDYLLTI------D-----------------ETF

NLSTPKKLNQLASS-YNLPWYEPKIYSYSFSEPNQ-Y-SKN--EALDLLV-KTVIRNMQWNTT----K----

KVINA--SDQTNNDNQN--IQQK-------------------------------------------------

-----------------------

>pCRESS6|ABJ73998.1

M--VDK-T------------------------------------NLKAIMLVQQLEDK---------HWELW

DDKTAIQAAREG-NCRPLLEEVVTRLSSE--N--IK-VEEAYGIIHNKDTISVWNTEKMKN-I---DE--P-

KANHVHFLFKF--DKGASLQKLALAMG-----IES-QYLE--KLK----SG--R----YGYDNCQAYLVHAK

----------------D----DS--KYQYSANEVT-TILG-----ED----YVSLYNRKMKSWIRGRAKKEA

QE--TNLS-VD-WIIAEVLAGQL-TKNQ----ILLTDEY-------YKVYG--QHKRKINEALDTAGERKSY

KTVAELEAGKF-KKTVIFVKADSGIGKTALSKKLIGLL-Q-MVAIK-FNQ--VW-DFCVTAS-TN----AFD

EY-NGQDILFLDDIRG---DS-L----TVSDWLKLLDPYMI-SP-ISARYHNKLGSA-K---VIIITSTKHP

FKFFE--NA---KGI-----------VG-EDLGQFIRRIDYLLTI------D-----------------GSF

NLSTPQKLNNSSIT-HRYKSLEPNTTSHSFSQPNQ-Q-SRN--AVLDLLI-KTVIRNMQWNST----K----

KYLNS--VEQKELQTEH--SDSKPDEIIESFPHLGKVEISKVRSNSS-------------------------

-----------------------

>pCRESS6|WP_053092713.1

-------------------------------------------------MLVQQLEDE---------HWKTW

QDKTALKQARDN-NIRPLLETVTDKLNRA--D--IT-VKEAYGIKHNKDEVNVWNQESMKN-V---IE--K-

KAEHIHFLFKF--EKGASLNRIALAIG-----VEP-QYLE--KLK----SG--R----YGYDNCLAYLVHAK

----------------D----ET--KHQYQPEDVT-TVLG-----ED----YKSIYHRSMATWVKGRATKKA

KE--TNLS-VD-WLVEKILAGDI-TKEN----IMLTNEY-------YAVYG--RHKQKVNEAIETAGEQKSY

QTIAELKEGKF-KKTILFISAPSSAGKTRFAKELIDII-Q-KVALK-FNQ--RW-DYSLTAS-TN----AFD

DY-NGQEILFLDDMRG---SS-M----TASDWLKLLDPYMI-SP-ISARYHNKIGSA-K---VIIITSTKKP

IPFFE--IA---KEN-----------DN-EDSGQFVRRIDYLITI------D-----------------KSY

NLSQPQHTTPQIND-FIDTWQNRKFHSFKFAKSKS-Y-TKN--KTIDILV-KTVIKNMQWNKH----K----

KVINA--SDQTNIDNPN--NKQK-------------------------------------------------

-----------------------

>pCRESS6|WP_027972054.1

M--VKKDA------------------------------------NLTAIMLVQQLEEE---------YWLSP

DYKEPLQQAKEG-NCRPLLELIIKKLREN--D--II-AKEAYIILHNKDTVTIWNPNELKN-E---VK--N-

KEEHVHALLKF--EKGASLNKIAFAVG-----IEP-QYLE--RLK----SG--R----YGYPNCLAYLVHAK

----------------D----EN--KHQYQPEEVV-TVLG-----ED----YVSIYHRSMETWIKGRATKKA

KE--TDLS-ID-WLIDKILAGEV-TKSN----IMLTDSY-------YAIYG--QHKRKINEALDTAGERRSY

QTIAEMEAGKF-KKTIIFIQAESGAGKTRLSKKFIALL-Q-KVALK-FGQ--TW-DFCVTAS-TN----ALD

EV-NGQEILLLDDLRG---SS-L----TVSDWLKLLDPYMI-SP-ISARYHNKIGSS-K---VIIITSTKKP

IDFFE--VA---KDN-----------VG-EDLGQFIRRIDYLLEL------G-----------------DKV

ALSVPEKQTKHKLDEDKIPWMLPPFASYDFSQKKL-Y-SIN--EAIDILV-KTTIRNMQWNTQ----K----

KVINA--SDQTNNDNPN--TQQK-------------------------------------------------

-----------------------

>pCRESS6|WP_032497992.1

M---VQHT------------------------------------NLTAIMLVQQLEKE---------YWKSE

DYEHSLEQAKSG-NCRPLLDLIIEKLEKQ--G--IL-VKEAYIIKHDKDKIVTWDSDKLEN-V---ST--N-

KAEHVHALLKF--EKGASLNKLALAIQ-----VEP-QYLE--KLK----SG--R----YGYDNCLAYLCHIK

----------------S----EN--KHTYKPEEVI-TARG-----ED----YTSIYHRRMEIWVRGRATKKA

KE--TLLS-VD-WLVEKILSGKL-TKNN----ILLTDEL-------FSIYG--QHKRRINEALETIGERKSI

QTIAELEAGKF-KKTIIFITADSGMGKTQYSKKLITIL-R-NIALK-HGQ--TW-ECCVTAS-TN----AFD

EY-NGQEILFLDDIRG---ES-L----TVSDWLKLLDPYMV-SP-ISARYHNKMGAA-K---VIIITTTNDP

LSFFR--KA---KGS-----------FG-EDLGQFVRRIDYLIQI------T-----------------DMF

HVSASIKNTPLVQT-DEIPCYENRHYSFKFSKIGA-Y-EKN--KATDKIV-KQVIRNMQWNKT----K----

KVIDA--SGQTNKDN-----SLL-------------------------------------------------

-----------------------

>pCRESS6|WP_015647385.1

M--IKKEA------------------------------------KLTTIMLVQQLENE---------YWLSS

DYQKPLQDAKSG-NCRPLLEMIVEKLEKH--G--IT-VKDAYIIKHDKDKVSIWNPNEMKN-I---FE--N-

KEDHVHALFKF--EKGASLNKLALAIQ-----VEP-QYLE--KLK----SG--R----YGYDNCLAYLCHSK

----------------Q----EN--KHQYQPEEVI-TVRG-----EE----YISIYHRSIETWIKGRATKKA

KE--TNLS-VD-WLIEKILAGDL-TKSN----IMLTDEY-------YSIYG--QHKRKINEALETAGERRSY

RAIAELEAGKF-KKTVLFINAESGVGKTQFSKNFIRLL-K-NVARN-FGD--SW-DSCVTAS-TN----PFD

EY-NGQEILFLDDIKG---DS-L----TVSDWLKLLDPYMI-SP-ISARYHNKMGSA-K---LIIITNTKEP

LSFFE--QA---KGN-----------IG-EDLGQFVRRIDCLLTI------D-----------------KNF

NISLPEKTVNPVEN-STHPWIEPKISSYNFANPIA-L-SSN--ETLDYLI-KTIIQNMQWNK-----K----

KAISD--TKQNTEDNPN--IQQK-------------------------------------------------

-----------------------

>pCRESS6|CGE81062.1

M-VIKKEA------------------------------------RLTAIMLVQQLKKD---------YWLSS

EYHFPLQEAIKG-NCLPLLEKIVEKLEQQ--D--II-VKEAYIIKHDKDETSVWDSKQMKN-I---LE--K-

KEEHVHVLLKF--ENGASLNKIALATK-----VKS-QYFE--KLK----SG--R----YGYDNCLAYLVHAK

----------------D----ET--KHQYSPEEVF-TVRG-----ED----YKNIYHRSMETWIRGRATKKA

RD--TKLS-VD-WLIEQILTGKI-NKRT----IMLSNEY-------YTIYG--QHKRKINEALDTAGEHKSY

RTVAELEAGKF-KKTILYITAESGAGKTQFAKRLIKLF-Q-KIALN-YGQ--IL-DYCVTAS-TN----AFD

EY-NGQEILFLDDIKG---DS-L----SISDWLKLLDPYMI-SP-ISARYHNKMGSA-K---IIIITNTKEP

ISFFE--HS---KGS-----------VG-EDLGQFVRRIDYLIEI------G-----------------NNF

HLSIPIKQKSKSQS-LSSVYYDNHQYSYEFSKVGE-Y-SIT--NATNEIV-KKVIQNLQWKES----K----

EDVFE--IKQFSENNP--------------------------------------------------------

-----------------------

>pCRESS6|WP_039677656.1

M--PKKES------------------------------------TLGAIMLVQQLENK---------YWLSS

DYNTAIQQAKDG-NCRPLLEIIIKKLEDN--G--II-LKEAYIIKHDKDEISIWDPQNMKN-I---IK--N-

KAEHVHILLKF--EKGASLNKIALAIE-----VEP-QYLE--KLK----SG--R----YGYDNCLAYLVHAK

----------------D----ES--KYQYQPEEVT-TLLG-----ED----YVSLYHRNMKTWVKGRATKKA

KE--TNLS-ID-WLIEKILAGEV-TKSN----IMLTDDY-------YTIYG--QHKRKINEALDTAGERKSF

QAIEDIDSGKF-KKTIIFLQGESGQGKTKLSKSIINIA-Q-RIAFN-NGY--PW-DSCSTAS-TN----AFD

EY-NGQDVLFLDDMRG---DS-L----TVSDWLKLLDPYTI-SP-ISARYHNKMRAA-K---LIIITSTKAP

LEFFS--LA---KGN-----------FG-EDLGQFVRRIDLLAEV------G-----------------DNI

KLSKPVKLESMTSP-FSIPLSTLSSHSFNFQNFEV-F-NRN--MAIDYII-KTIIRNMQWNK-----K----

ERITH--TDQSTKDTL---TQPK-------------------------------------------------

-----------------------

>pCRESS6|WP_044774450.1

M--VKKEA------------------------------------NLTAIMLVQQLEEE---------YWLSP

DYKEPLQQAKEG-NCRPLLELIIKKLREN--D--VI-AKEAYIIKHDKDTVTIWNPSESKN-E---IK--N-

KEEHVHALLKF--EKGASLKKIALAIS-----VEP-QYLE--KLK----SG--R----YGYDNCLAYLVHAK

----------------D----ET--KHQYQPEEVI-TIKG-----ED----YTSIYHRSMETWTKGRAIKKA

KE--TDLS-VD-WLIEKILAGEI-SKSN----ILLTDEY-------YAIYG--QHKRRINEALDTAGERKSY

EAIADLEAGKY-KKSAIYVLADSGVGKTKFCMELIHRL-Q-NIAKEDYTY--NL-SYCLTAS-RN----AFD

AY-QGEEILFLDDIRG---DA-L----SVSDWLKLNDPFMI-SP-ISARYHNKMGSA-K---LIIITSTLLP

SVFFS--QA---EGN-----------KN-EDNGQFIRRFDYQVHI---PE-S-----------------DKF

LLSTPEKNEPIEGQ-YAF----PIFHSYSFSTASEVL-DKD--AAMERII-NTILDNMNL-KN----K----

KVINA--SDQTNNDNPN--TQQK-------------------------------------------------

-----------------------

>pCRESS6|WP_020997784.1

M--VKKEA------------------------------------NLTAIMLVQQLEEE---------YWLSD

DYKSVIQKAKDG-NCRPLLEIIIQKLEEH--D--II-AKEAYIIKHDKDKVTIWNPNKMKN-E---IN--D-

KEEHVHALLKF--EKGASLKKIALAIG-----VEP-QYLE--RLK----SG--R----YGYDNCLAYMVHAK

----------------DF---PE--KYQYSPDEVI-TVLG-----ES----YKSIYQRKIETWVRGRATKKA

KE--TDLS-VD-WLIEKILAGEI-SKSN----ILLTDEY-------YAIYG--QHKRRINEALDTVGERKSY

QAIIDLETGNY-KKSALFIMADSGVGKTKFSMELIHHL-Q-NIAKENYDY--NI-SYCLTAS-RN----AFD

EY-QGQDILFLDDIRG---DS-L----SVSDWLKLTDPFMI-SP-ISARYHNKMGSA-K---LIIITSTLLP

SVFFS--QA---VGN-----------KN-EDNGQFIRRFDYQVYI---PE-S-----------------DKF

LLSVPEKNEQVEGQ-NAF----PVFHSYSFSTATEVL-DKD--TAMERII-NTIFDNMNL-KN----K----

KVINA--SDQTNNDNPN--TQQK-------------------------------------------------

-----------------------

>pCRESS6|WP_003024533.1

M--SRTQA------------------------------------NLTAIMLVQQLEEE---------YWIDW

KDITSLTQAREG-NSLPLLQEVVKRLNQA--D--IS-VSEAYGILHDKDTISIWNQEQLKN-V---TE--L-

KKKHVHFLLKF--EKGASLQKIALSIG-----VEP-QYLE--KLK----SG--R----YGFENCLAYLVHAK

----------------D----IE--KHQYSPDEVV-TLLG-----EN----YTSIYNRRMQVWLKGRATKEA

RE--TNLS-VD-YLISEILKGNI-TKNN----ILLTNEY-------YKVYS--LHKRKFLEAFETFGERKGY

QAIADLEAGKF-KKSVFFIHAESGKGKTRLAKHLIQLI-Q-SEARK-QGE--NW-EFCLTAS-TN----AFD

EY-NGQDILFLDDIRG---DS-L----TLSDWLKLLDPYTI-SP-ISARYHNKMGSA-K---VIIITSTRTP

IEFFQ--LT---KGS-----------IN-EDSGQFIRRIDYLLKL----S-D-----------------KGY

QLAIPLPQ--------------SLIPSFSLGKPRL-Y-SRG--KAIYKLV-KAVSRNMQWNQ-----K----

KTVSD--TDQSYRDSQT--TQQK-------------------------------------------------

-----------------------

>pCRESS6|ADX23728.1

M---KKQA------------------------------------KLTCAMIVQQLEKD---------FWLNW

D-NFFIDQAREG-DLIPLLEEIEKRLVSH--N--CI-ISELYAIKHDKDKLVIWNEEEKST-I---EE--L-

KASHVHILIKF--EKGTTLSQLAYILG-----IEA-QYIE--KAK----SG--R----YGYDNLLAYLVHSK

----------------D----KA--KFQYSPNEVI-TLKG-----ED----YLSVYNRRKQIWFKGRATKEA

KE--ANLS-LD-YVISEILEGHI-TKSQ----VLLTNDY-------YKVYA--LNQRKIEDAFAAYQEKKGF

VTIQSLENREF-RKTIIFITGKTASGKTSLAKEIIKSI-K-DIAFR-QGE--TW-EHCITAS-TN----SFD

NY-NGQEILFMDDVRG---YG-L----TATDWLKLLDPYNI-SP-ISARYKNKLGYA-K---VIIIASSVEP

SLFFH--SA---KNY-----------HY-EDPSQFIRRLDALVKI------D-----------------TTY

QLSIPHKIQMVNHS--------EMLSDYYFKNIAK-G-KHE--KIIQKVL-SMFLKNMKWK-----------

--------DEKNNN----------------------------------------------------------

-----------------------

>pCRESS6|WP_056938517.1

M---AKDS------------------------------------TLTCVMIAQQLQPE---------FWHGW

D-EQPIIQAQNG-DARDLLDNVVQRLDKS--N--VK-VSEAYGIIHNKYTETIWNAEKQQN-S---IR--Q-

KEDHVHFLLKF--DKGNTINNLAMTIG-----VEP-QYLE--KAK----SG--R----YGYDNLLAYLVHAK

----------------D----KD--KFQYNPKDVT-TAVG-----ED----YLSVYNRRRETWLRGRATKEA

QN--SMQS-VD-YLIAQVLQGKL-TKSQ----IMSDEDL-------YMVYG--LNSSKINGAFTVIGERKSI

TAQRDIEASKF-KKKIIFISGTAGVGKTKFGKLLVRQI-Q-KAVQKEHNC--YW-ECCVTAS-TN----PFD

EY-SGQEILFLDNVRG---ET-L----GFLDWLKLLDPHNI-SP-ISARYHNKFGVA-K---VIIITSPVPP

YQFFN--HP---KFN-----------SM-EDLGQFYRRIDFWISF----S-N-----------------NKL

LVCNPIRDFWHHNS----KYHYLHNSSYRFSKNGL-Y-KKT--NAIQRIL-KLINNNMKWKKK----K----

EQITT--AG-----------KLK-------------------------------------------------

-----------------------

>pCRESS6|WP_000201649.1

MATKKKEA------------------------------------LLTSVAITQYFDPK---------YWNGW

D-EELIKSA----NVEKILEEIVRRVGEI-----AT-VSEAYAIKHDKDTSIGFDSVTRTT-T---TK--L-

KKPHIHALLKF--EKGATLTDFAVQIG-----LKP-EYLE--KAK----SG--R----YGYDNLLAYLIHAK

----------------D----KD--KYQYSPDEVI-SLTG-----KD----YLKVYHERHLSWLKGKAKKEV

KQ--TYKD-ID-LLIDNILNGNI-TKKE----MLLNKDY-------HMLYA--VHKSKVNEVFRTIGEIKGT

MTQHELENKKF-KKTIFFIFGLSGLGKTKFARTLTKSL-I-QLAKL-NDQ--NW-QSVLTAG-TN----MFD

EV-NGEEILLLDDVRG---DS-L----TASDWLKLLDPYNI-SP-ISARYQNRLGAS-K---VIIITSSKHP

LTFFY--HA---KGN-----------TN-EDLSQYIRRIAHLVTL---RG-NNDN--------------ITF

HESQPKRTINR-VV-KIPGTDQTTSLSYDFTPDNE-AASKE--ELLSMLV-STVGLYNKWEHWDYNKI----

KTPSE--TLASE-DEVA-DNQEK-------------------------------------------------

-----------------------

>pCRESS6|WP_047206721.1

MATKKKEA------------------------------------LLTSITITQYFETK---------YWNGW

D-DELIKSE----NIEKILEEIVRRVSEI-----AT-VSEAYAIKHDKDTSIGFDSVTRTT-T---TN--L-

KKPHIHALLKF--EKGATLTELAVKIG-----LEP-QYLE--KAK----SG--R----YGYDNLLAYLIHAK

----------------D----KD--KYQYTPDEVF-TLKG-----KD----YLEVYHERHLSWLKGKAKKEV

KQ--TYKD-ID-LLIDNILNGTI-TKKE----MLLNKDF-------HMLYA--VHKSKVNEAFRTIGEIKGT

MTQNELENKKF-KKTILFIHGVSGTGKTTLANQIVQNL-I-QLAKL-NNQ--NW-QSVLTAG-TN----MFD

EV-NGEEILLLDDVRG---DS-L----TASDWLKLLDPYNI-SP-ISARYQNKIGAA-K---VIIITSSKHP

LTFFY--HT---KGN-----------NR-EDLSQYIRRIAHLVTL---RG-NSEN--------------ITF

HESQPKRTIDR-VV-KIPGTDQTISLSYDFTPDNE-AASKE--ELLSMLV-STVGLYNKWEHWDYNKI----

KTPSE--TVASE-DEVT-DNQEK-------------------------------------------------

-----------------------

>pCRESS6|CMU27730.1

MNTNKKEA------------------------------------ILTSVLLTQQFSNG---------FWDDW

N-KELLESA----DIEKILEEIVRRVSEV-----AT-VSEAYAIKHDKDTSIVFNNELQET-T---TK--P-

AKSHIHALLKF--DKGATLSTLSKKIG-----LAE-QHLE--KAK----SG--R----YGYDNLLAYLIHAK

----------------D----KD--KYQYSPDEVF-TLMG-----NN----YLKVYHERKLSWLKGKAKKEV

RQ--TYED-ID-LLIDNILNESI-TKNE----ILLEQKY-------RTLYA--VHKARINDTFRTVGEIKGT

RTKYELDNEEF-KKTILFIHGSTGLGKSKFAKELTKDI-V-QLAKL-NGQ--NW-QSVVTAA-TN----IFD

EV-NGEEILFLDDVRG---DS-L----TASDWLKLLDPFNI-SP-ISARYQNKMGAA-K---VIIITSSKYP

LDFFY--DT---KGN-----------DR-EDLSQYVRRIECLATI---KG-NDKN--------------PKF

YVSYPQRMEEP-VK-TILENEQEVSLSYDFTDDSL-LNSRQ--DLLSTLL-SKIAINNQWDILELDKS----

KTPSE--TLASE-DEVE-DNQEK-------------------------------------------------

-----------------------

>pCRESS6|WP_000044268.1

M-SKRKET------------------------------------FLGSILVTQQFDID---------YWNGW

D-KETIESE----DIKKILAEIIERVNQV-----AT-VAEAYAIKHDKDFTELFDIETQST-L---TK--P-

VEPHIHALLKF--SKGATLPELAAHIG-----IEP-QYLE--KAK----SG--R----YGYDNLLAYIIHAK

----------------D----SD--KHQYNPDEVI-TLLG-----KD----YQEVYQERQKSWLQGRAKKEV

QK--TQED-ID-LLLDDILNERI-TKQE----LLLNPSY-------HLLYV--VHKTRINEAFRAIGEIKGT

RTKQDLENGLF-KKTILFIYGKSGLGKTRLAKELVSLL-E-QLASV-NGQ--KW-QSVLTAG-TN----IFD

EV-NGEEILLLDDVRG---DS-L----TASDWLKILDPYSI-SP-ISARYQNRIGSA-K---VIIITSTKHP

LEFFY--HT---KGN-----------DR-EDLSQYIRRFDFLISL---ES-EREN--------------LVY

FESSPTKVYQR-RR-KIPKTDIDVYLSYDFSANAR-LANKS--YLLELVL-AKIGLNNQWENWDY-KL----

KTSSD--TLASDTDEAEHSTEEK-------------------------------------------------

-----------------------

>pCRESS6|WP_001034312.1

M--NKKEA------------------------------------NLSCIMIVQRLEPE---------YWQGW

D-EHLINSAKNG-ELTPLLEEVVNRVSEI-----AT-VSDAYAIIHDKDINEVYDLETQDI-N---TK--P-

EKPHAHILLKF--SKGETLINLSLQLG-----IEP-QYIE--KAK----AG--R----YAFDNFLAYLIHAK

----------------D----SD--KFQYDPKEVI-TLHG-----KD----YLEVVTERYKSWKKGRAKKDI

SK--SNTS-LD-EIYLQILNQQI-SKQE----ILSDPEL-------QILYA--LNKTKINEAFMTLGEIKSN

ATKQALENGEF-KKTIIFITGKSGLGKSRFAKTFVKEL-I-SLANI-NNH--SW-SDVVTAG-TN----IFD

EV-NGEEILLLDDVRG---DS-F----TASDWLKLLDPYNI-SP-ISARYHNRMGSA-R---VIIITSTKHP

LEFFI--HT---KGN-----------EK-EDLSQFIRRTTSLVTL---YR-DSPSCD------------TRY

FHSSPKSVPNR-RL-KVPHTDIEIYMSYDFENNSE-I-SKE--NLQEFLL-AQVSINNRWD---LDKI----

KNSSED-TLASNSDEFQISTGEQYPTVPQL------------------------------------------

-----------------------

>pCRESS6|WP_024385235.1

M--SKKEA------------------------------------KLTCIMITQQLESE---------YWLGW

D-EDIINGAKDG-DLSLLLEEVVNRVSEI-----AK-VSDAYAIIHDKDVNGIYDLETREI-K---TV--P-

ERNHAHILLKF--SKGATLISLALQLG-----IEP-RYIE--KAK----SG--R----YAFDNFLAYLIHAK

----------------D----SD--KFQYDPKEVI-TLHG-----KD----YLEVVTERYKSWKEGRAKKDI

SK--SYTS-LD-EIYLQILNQQI-TKQE----ILSDPKL-------QILYA--LNKTKINEAFMTLGEIKSN

ATKLALENGEF-QKTIIFITGKSGLGKSRFAKTFVKEL-I-SLANT-NNY--SW-SDVVTAG-TN----IFD

EV-NGEEILLLDDVRG---DS-L----TASDWLKLLDPYNI-SP-ISARYHNRMGSA-R---VIILTSTKHP

LEFFF--DT---KGN-----------EK-EDLSQFIRRTTSLVTL---YS-DSPFSN------------TRY

FYSFPKSVTNR-RV-MVPHTDIEVYLTYDFENNSV-I-SKE--KLLEFLL-AQVSINNRWD---LDKI----

KNSSED-TLASNSDE---------------------------------------------------------

-----------------------

>pCRESS6|WP_004183001.1

M--SKKEA------------------------------------KLSCIMIAQQLEPK---------YWHGW

D-ENLINSVKNG-ELTPLLEEVVNRVSEI-----AM-VSDAYAIIHDKDVNEIYDLETHTI-K---TE--P-

ENVHVHILLKF--SKGATLISLALQLG-----IES-QYIE--KAK----SG--R----YAFDNFLAYLIHAK

----------------D----SE--KFQYDPNEVT-TLHG-----KN----YLDVVTERYKSWKKGRAKKDI

SK--SNTS-LD-DIYLQILNQQI-SKQE----ILSDPKL-------QIIYA--LNKTKINEAFMTLGEIKSN

ATKLALENGEF-KKSIIFITGKSGLGKSRFAKTFVKEL-I-SLANI-NNY--CW-SDVVTAG-TN----IFD

EI-NGEEILLLDDVRG---DS-L----TASDWLKLLDPYNI-SP-ISARYHNRMGSA-R---VIIITSTKHP

LEFFF--HT---KGN-----------EK-EDLSQYIRRIDSLVTL---YR-ETPKSS------------VEY

LQSSSESVANR-IV-KVPSTDVEVSLIYDFKHNGK-M-SKE--ELLEFLL-AQVSLNNKWS---LDKI----

KNSSED-TLASNSDE---------------------------------------------------------

-----------------------

>pCRESS6|WP_024400359.1

M--SKKEA------------------------------------KLSCIMIVQQLEPE---------YWHGW

D-EDIINKAKEG-ELTLLLEEVVNRVSEI-----AT-VSDAYAINHDKDVNEIYDLETNKI-K---TE--L-

ASPHVHILLKF--TKGSTLTNFALQLG-----IEP-QYIE--KAK----VG--K----WGYDNLLAYITHQK

----------------D----PE--KFQYDPKEVI-TLQG-----KD----YLEIVKERYKVWQQGRVKKDI

SK--SNAS-LD-EIYLQILNQQI-SKQE----ILSDPKL-------QILYA--LNKTKINEAFMTLGEIKSN

ATKQALENGEF-KKTIIFITGKSGLGKSRFAKTFIKEL-I-SLANT-NKY--RW-SEVVTAG-TN----IFD

EV-NGEEILLLDDVRG---DS-L----TASDWLKLLDPYNI-SP-ISARYHNRMGSA-R---VIIITSTKHP

LEFFF--HT---KGN-----------EK-EDLSQFIRRTDTLVSL---TE-DFVNGP------------VRF

FQATPEKVSNR-KV-KIPHSDINVNLSYDFKKNYQ-S-DKE--SMLEFLL-AQICINNHWD---IDT-----

------------------------------------------------------------------------

-----------------------

>pCRESS6|WP_000791389.1

M-KNKKQA------------------------------------KLTCVMIAQQFDSQ---------FWIGW

D-KDLILSK----SIKDILTEMVNRANTV-----AT-VSEAYGIKHDKDTLELYDSETDSF-Y---QE--L-

KSEHGHFLLKF--SEGATLIDLALAIG-----VEP-QYIE--KAK----SG--R----YGYDNLLAYLIHSK

----------------D----PD--KFQYSASDVI-TILG-----KD----YLEIYKEKFSSWSKGRTKKEV

RN--ASEQ-ID-SLIANILNGKI-TKSE----ILLSSNH-------LQLYA--LYKTKINEAFMTLGEVKAT

RTKKALENGDF-KKSIFFITGKSGLGKSVLSRELVGDL-I-QLAEN-NEQ--TW-SSVATAG-TN----IFD

EV-NGEEILLLDDVRG---DS-L----TASDWLKLLDPYNI-SP-ISARYHNKMGAT-R---VIVITSTKHP

LEFFF--KT---KGN-----------EI-EDLSQFIRRFDSLITI---DN-TAETTP------------IRY

FQSLPKRVVNR-RV-KIPYTGFETYLSYDFENNSE-M-NKK--DLLDFLV-AQVSLNNKWD---LDKI----

KNSLEDGTLASSSDEVDTEDSKSPDNSIVAQ-----------------------------------------

-----------------------

>pCRESS6|WP_003032217.1

M---AKES------------------------------------KIQCFMVVQYFSEK---------YWKDW

D-VSFITEAQEG-DIAPLLNEMVKRISKI-----TT-VSEAYGIVHDKDEEELYDAETQQF-Y---TK--P-

IDSHGHLLFKL--SEGMPIIKLSDIIG-----IEP-QYIE--KAK----RG--R----YGYDNLLSYLIHSK

----------------E----SN--KHQYSPQEVV-SAAG-----KN----YMEVFKERFSSWKKGRVKKDI

KL--SEDE-ID-NLILDIIQEKI-GKSE----ILLDEKY-------HMIYT--TFKTRINEAFAVLGEIKGN

RTKKAIENGEF-KKTVLFIHGRSGLGKTRFAKELASKI-Q-GLALR-NGQ--KW-DLATTAG-TN----IMD

DL-NGEEILLLDDVRG---ES-L----TASDWLKLLDNYNI-SF-SSARYKNILPSA-R---IIIVTSTKHP

LEFFY--KC---KDN-----------ER-EDLSQFIRRFDSLITL---QN-CQEGDN------------IQF

FQASPRKTSNV-KR-KIPNRETIVTLNHDFEKNNF-M-NKG--ELVEFLL-AQVSLNNKWE---LEQE----

KSLSD--TFSGAADK-----PSK-------------------------------------------------

-----------------------

>pCRESS6|WP_018376545.1

M--GKNKA------------------------------------ILTVIAGSQQLRTE---------LWQSY

S-EKAIQKAIEG-APDELLKEMIEHLSSV-----AK-ISDAYIIVHDKDTKYVWNPELKKY-E---QV--L-

KSFHVHFLFKF--SEGATLTEIANALN-----LEI-QYLE--KPK----SG--R----YAYDNLLSYLVHAK

----------------D----HN--KFLYSPSEVV-TLLG-----ED----YETIYNERIHVWEKGRAKKTS

QQ--AKFD-VE-ALIMDIIVGKI-KKTE----VLSSPDL-------YPIYA--FNKSKLNEAFETYAERKSL

KTLKDLELGLF-QKTIIFFQGASGLGKTTLAKELTHEI-Q-LLSKA-NGE--DW-EAIITAA-TN----PFD

DV-KGEEIILLDDVRG---QA-L----SASDWLKLLDPFTA-SP-ISSRFKNRSGAV-K---TIIITSRKPP

LEFFF--KT---KDS-----------GY-EDLSQFIRRIHHFITL---RT-DGTGN-------------TIY

SSSFPERKQTPIRR-KIPHKDITVSLDYDFSTAKE-M-GSK--DLLKFLI-ETVRLNNRWE---ISYE----

ESKKA--ITSKPTKAES--D--G-------------------------------------------------

-----------------------

>pCRESS6|WP_020999261.1

M--TKHKA------------------------------------NLTIVAGAQQLRTE---------LWKSY

P-EEIIQKALDD-TPEELLREMVKHLSSV-----AT-VSDAYIIVHDKDKKEVWNPELQKY-E---QV--L-

KASHVHFLFKF--SEGGTLSDIANALG-----LET-QYLE--KPK----SG--R----YAYDNLLAYLVHAK

----------------D----SN--KFHYSPSDVV-TLIG-----EN----YETLYNERIRAWEKGRAKKTS

KL--AKFD-VE-ALIIEIIEGKI-KKER----ILSSPDL-------YPIYA--FNKTKLNEAFETYAEQKSM

KTLKNLELGLF-QKTIIFIQGSAGLGKTTLAKKLAREL-Q-LLAQA-NGE--DW-EYIITAA-TN----PFD

DV-KGEEIILLDDIRG---QA-L----SSSDWLKLLDSFTA-SP-ISSRFRNRSGAV-K---TIIITSSKHP

LEFFF--KT---KDS-----------GY-EDLSQFVRRINYLITL---RM-GNSGD-------------TIY

SNSHPKRKHTPIIR-KIPNKEVTVSLDYDFSQAKT-M-SAE--DTLAFLI-ETVRLNNHWE---ISYE----

EYKKA--ITSKPTKTES--D--S-------------------------------------------------

-----------------------

>pCRESS6|WP_044762265.1

M--TQKQS------------------------------------NLTVVAGTQQLEFE---------FWKNY

P-KELIQKAIDG-NAKELLHKMVEYLSTI-----VV-VADAYIIIHNKDWSEVWNPSLNEY-E---KV--L-

KTIHVHFLFKL--AEGATLPEIANALG-----LET-QYLE--KPK----SG--R----YAYDNLLSYLIHAK

----------------E----PD--KFFYSPSEVV-TLMG-----RD----YQTIYNERIRTWEKGRAKKTS

KQ--AKND-VE-VLIMDIIKGKI-KKDE----ILNNPEF-------YAIYA--FNKSKLNEAFETYAERKSL

KTYKDLELGLF-QKTIIFLQGASGLGKTTLAKELAHKL-Q-FLSRV-NGE--DW-ETVITAA-TN----PFD

DV-RGEEIILLDDVRG---QA-L----SASDWLKLLDPFTA-SP-ISSRFKNRSGAV-K---TIIITSSKSA

LEFFF--KT---KDS-----------GY-EDLSQFVRRINHFITL---RT-DSSGN-------------IIY

SSSFPERKHTPIQR-KIPNKEVIVSLDYDFSTAKE-M-SSE--NLLEFLM-ETVRLNNHWE---ISYQ----

DSKKA--ITTTDQSKSD--DLNQ-------------------------------------------------

-----------------------

>pCRESS6|WP_022765681.1

M--ARTES------------------------------------DLTACGIAQELNPE---------LWDWT

DEEKAAF----P-DLQRVTAIIAKRLEDK--G--IQ-LKSLRGVIHDSDTQQKWNEAMKTY-D---VT--L-

KHLHAHWVIEF---KGAKLTDIALAIG-----LAS-QYVE--KPK----KG--R----YAVDNMLAYLIHIK

----------------Y----PD--KYQYSPTAVY-TYCG-----RD----YMDIYAERESVWKKGIATVKK

LA--AAED-ID-WLEEQILEGKI-KKTQ----IFLTDSY-------FKTYS--HYRQRCEDAFACYTDKKIY

TAIEAMKNKEF-LLTTYFITGASRKGKSRFAEDLAQRI-IDENEKR-TGI--TW-SYCRTPT-SN----PLD

DY-AGEEIIIMDDSRG---CT-L----TAEAWLTFLDPNFC-NP-AGARYHNKPGIPAK---VLIITSTKSM

LEFFF--YT---KGI---GGGS----RS-EAMDQFFARVFSRIEV---ID------F------------DTY

IIDKIEEKAESYTL---TQDGEELELRYAPVSLGT--ATKE--EAISLLSEAVLARCGKENYASD--K----

KRAKI--NEEREKHKEEQEQKKIIVENSKIQEVPLYDAVRTKELDTAYAIYKLYRDGLMPKESFEKYLKEEY

HPTLQVKTIEDFEILPYWASQDP

>pCRESS6|WP_054952722.1

MAVAKQEN------------------------------------NPTSIGLTQYLDPS---------YWAWA

AEDPNGAALLQQ-GAEAILAYVVQRLEAT--G--CE-VVEAYGIVHDKDEREVWSDTEKAL-V---VE--P-

KPEHLHAVIKFASAKSAPLDRLAFGIG-----VEP-QYVE--KPG----RG--R----YAFDNMLSYLTHVK

----------------Y----AD--KHQYAPSEVA-TVRG-----PD----YLGIDAQRRETWQKGRAHVKK

KI--VAEN-FE-DMRERVLQGEI-TRDQ----IMLTDEL-------FDIYS--RHQREIDDALSAYGQRRAY

RAAAKLRAGEF-STHVVFVHGDAGIGKTRFATDFITEA-I-NAANA-HGE--RW-QVYRAAT-GN----PLD

DW-RGEEVLLLDDLRA---SA-M----DANDWLLLLDPYNA-SP-AKARYKNKGEVAPR---LIVITATIEP

VEFFY--YARQ-MGN-----------VD-EALDQFIRRLASVVKV---YRADDI---------------NRY

LVQHIGKIEPYEWHQCSVPAAASRELTYGPETSAE-H-DAD--GAVAELL-GGLAVRSPD------------

------------------------------------------------------------------------

-----------------------

>pCRESS6|WP_041290927.1

MAVAKQEN------------------------------------NPTSIGLTQYLDPS---------YWTWA

AEDPNGAALLQQ-GAGPILAYVVQRLEAI--G--CE-VTEAYGIVHDKDEREVWSDTEKAL-V---VE--P-

KPDHLHAVIKFASAKSAPLDRLAFGIG-----VEP-QYVE--KPG----RG--R----YAFDNMLSYLTHVK

----------------Y----AD--KHQYAPSEVA-TVRG-----PD----YLGIDAQRRETWLKGRAHVKK

KV--VAEN-FE-DMRERVLQGEI-TRDQ----IMLTDEL-------FDIYS--RHQREIDDALSAYGQRRAY

RAAAKLRAGAF-STHVVFVHGDAGIGKTRFATDFITSA-I-DATNT-HGE--RW-QVYRAAT-GN----PLD

DW-RGEEVLLLDDLRA---SA-M----DANDWLLLLDPYNA-SP-AKARYKNKGEVAPR---LIVITATIEP

VEFFF--YARQ-KGN-----------VD-EALDQFIRRLASVVKV---FRADDI---------------NRY

LVQHIGKIEPYEWHQCSLPASASRELTYGPESSVE-H-DAD--SAVVELL-SGLAVRSPD------------

------------------------------------------------------------------------

-----------------------

>pCRESS6|WP_036321578.1

M--ARKAA------------------------------------NPTLVMLVQQIKPE---------CWQ-H

STDPVFAGLLEQAAAASFSQYLVVLLESV--G--LV-IEEAHCILHGDDTQLGWDDATASM-V---EV--L-

KYLHLHLLLKFKSETSAAVEKLAAILG-----VEV-QYVE--DKS----RGKIT----QQHDNGLAYLTHVK

----------------Y----PD--KFQYPPEQVA-SVRG-----ME----YQQVYRERYPAWRKGRAHVKA

KR--AKLE-FE-PFREMVLQGEL-TRDQ----IMLTDEY-------FDIYS--RHQREIDDALSAYGQRRAY

RAAAKLRAGAF-STQVVFIHGEAGVGKTRFANAFIQEA-I-SCAKG-HGE--RW-QVYRAAT-TN----PLD

DW-RGEEIMLLDDLRA---SA-M----DANDWLLLLDPHNA-SP-ARARYKNKGEVAPR---LIVITATIEP

VEFFF--FARQ-KGN-----------VD-EALDQFIRRLQSVVRV---FREDDI---------------LRY

LVQPVVKGDPYKRAISTKQASGVLSLSYGPAGGGA-H-DAG--AAVDELL-DGLAEHSRD------------

------------------------------------------------------------------------

-----------------------

>pCRESS6|WP_051176704.1

M--S-GGN------------------------------------KLQIFHITQQLRPE---------LWSWV

N-DPKVQELAEVDRANRLLSLLVLLLEQT--T--LK-IVEAHGIIHNCDEQNT-AKIGQPA-V---ME--P-

KFPHLHATVKF--KPTFKIARIAKILG-----VET-QFVE--KPNRIPPQG--S----GSHDAQLAYLIHAK

----------------D----ED--KYQYEPEQVA-TVAG-----QD----YLEIQKQREAEWFLGRGKKRH

KL--AALS-VD-ALLEQVLSGNI-TKEN----VLLSDEL-------FRVYS--ELSDKFNRAFVVAGERKAY

RAAEAMKNGDF-HTAIFFLYGAAGSGKTRLANLLVDQL-I-ESTN--------W-SLYRAAS-RH----SLD

DW-QGQEIILMDDVRA---SA-M----SASDWLTLLDPYNP-NP-ASARYQNKLAVAPR---LVIITASIDP

VTFFY--YARA-KGD-----------VD-EALDQFIRRLMASVNV---INTDGM---------------PRY

YYKRLGLLSERDELVRHRQGVDTPELTRGVVDSIK-C-DEP--EIIARLS-LDVANSSPDQIWREKNR----

WVLLPSNPSFNGYYEGFKPLEIDGSDIEFFDDDDEVDYSDLYSDYLPPD---------------CDLAS---

-----------------------

>pCRESS6|WP_052506726.1

M--ANKD-------------------------------------KLSMCMITQAWLSN---------HWDSW

N-DDDINNA----NLSLVTQKFVEYLSEA--G--AE-VVACYGITHDRDTREVWNDTVKSY-V---LE--P-

KHLHGHWVVKFASDKGLTLSDIAVAVG-----LAP-QFIE--KAK----PG--R----YGYDNMLSYLIHAK

----------------D----AE--KFSYSANDVV-SVVK-----NE--PAYSEIYAERKSAWEKGRAKKTA

KRVTSDEL-VE-ELYEKVINGEV-THDQ----ILLTDEY-------YAVYS--RNIDRFEKGFKAYTDRKIL

QAVRDLEAGKF-NLSVLYFQGQAGHGKTATAVQLAQSL-V-VKAAE-RGE--RW-SICQAAA-TN----PVD

DY-NGEEILIMDDLRG---NA-M----RATDWLKLLDPYNS-SP-NSARYKNKRVVS-R---YIFITSIQDV

YEFFY--YT---KNA---GAD-----RS-EPLDQFMRRILALVKV---VKVGDEKRI------------GVY

PQVSYDYRKRVENDKKDKGYD-ELTLHHGFYAKGLHFTSDNFGQFVEHLFDDEKRVNMLEDERNAVNQ----

NAPVDLLPLLELEDIVA--DDKK-------------------------------------------------

-----------------------

>pCRESS6|AEU41945.1

MKPKAKRK------------------------------------QASVFGFTQQFKAD---------MWDWA

DDEKAVCFPNGP-DTARIMKRVAERLKKA--N--AKDLFKYSAIIHDKDMSFAWDTKTNSK-V---IV--P-

KELHMHAVIEL--PSKRDLSFISTAIG-----IRP-EQIE--VPR-----G--R----YGRENMLAYLVHAK

----------------D----GD--KYQYSPSEVE-TFNT-----WD----YMSYYISRVDTWNYRKATVVK

KA--NSVK-AD-WLVKEVQAGRV-SKET----IMLTDDY-------AEVYA--DNMRAINDAFQYYAERQGF

KTLEALRNNEF-EMSVYYIQGAPRMGKTFFAKKFVNDL-IDTRFAK-TGE--RW-RSYETAD-TQ----PMD

DY-AGEEIVFMDDLRA---SA-M----TASGWLKLLDPLTT-AP-MSARYKNKQKAT-R---VIVITSYLDP

FTFFS--YV---KGV---GG------SN-EALEQFIGRLSLIAKV---LRFDGDDRVIEIDGIVQSSEAKHF

VVGSNTTLLTTDEVKLLPRVEREHIKSTKFFNENIVSGDVS--MALEFMI-DDVMTKNDSSKKHIIGE----

KPRAGFHEDVKSWNEYL--NDSE-------------------------------------------------

-----------------------

>pCRESS6|WP_025016923.1

M----LDK------------------------------------RVRSFIFTQYLNPK---------YWEWE

NDE--LFDDYIN-NKQAIFEEIFARANEL------EGLKICALIVHDKDE---------NK-----NG--L-

KDAHIHGYLEF--SKQKTIASISSVLA-----IEP-QYVE--APK----KG--R----YGRLNCLAYLIHAK

----------------N----LD--KYQYSPEEVE-TFET-----FD----YEEFIDDNLEDFKKYNATDKR

KK--GEIG-LD-LALQEVQQGKL-KLRE----IMRDENL-------ALLYA--NHMNQFNDSFNFYGLRNAM

LRLDELEQGKY-DLTVLYIQGAPGIGKSFLAREVAQKV-R-EYGNK-HGF--AS-DVFSASS-SN----PFD

DY-YGEDILILDDLRQ---ES-L----KVSDWLKLFDPLNT-AR-MSARYRNKMIVP-R---LVILANYQSI

EQFFGSFNF-----------------KN-EDINQFIRRISFSSKI---TEKSFGIPPFD----------RIY

SLSASKKLNEPENMRISKNE--FITLNYGTKLLFT-YDDKE--KFINELL-TQHVYPRIF-------S----

EK----------------------------------------------------------------------

-----------------------

>pCRESS6|KST89836.1

MFKKMRDK------------------------------------RVKSFIFTQYQQAD---------FWDWD

EDKKILFENSRN-NQAKIFNEIYNRLKNL------EGLKIIALVNHDKDK---------NA-K---GE--L-

KHPHIHGYIEF--EKLKTIASVSACLG-----IEP-QYVE--VPR----KG--R----YGRLNCLAYLVHAK

----------------N----LD--KYQYSPEEVQ-TFET-----FD----YQEFIDDNLEDFKKYNATDKR

KK--NEIG-LD-LALQEVQQGKL-KLRE----IMRDENL-------ALLYA--NHMNQFNDSFNFYGLRNAM

LRLDELEQGKY-NLTVLYIQGAPGIGKSFLAREVAKKV-R-EFGNQ-NSF--ES-DIFSASS-SN----PFD

DY-YGEDILILDDLRQ---ES-L----KVSDWLKLFDPLNT-AR-MSARYRNKMIVP-R---LVIVANYQSV

EQFFGSFNF-----------------KN-EDINQFKRRINFTSKI---SEKSFGLPPFD----------RIY

SLSASKKLSEPENRRISNNE--FITVNYGTKLLFS-YDDRN--RFINELL-TQHVYPRIF-------P----

QKEKKGN-----------------------------------------------------------------

-----------------------

>pCRESS6|WP_018030886.1

M---SDRK------------------------------------RATTFIFEQQLKSD---------YWDWE

EEKKKLFQDWKE-NRVAIFKEIHRRIQTLEESIP----AKVAFIIHDKDK---------KY-N---LT--L-

VEPHIHGYIEF--ASRRDLNHLASVLG-----LLP-QYIE--PSG----RG--K----YGKVNSKAYLIHAK

----------------N----PD--KYQYSPDDVE-TFGT-----FD----YKEFFQEHQLDFIKRSATVKR

EK--SDEE-LD-SVFQAIVNGTL-TEDD----IFANEEL-------TFLWS--YNQTKLDEAFRAYGKIASK

RTLRQLESGEF-QPAVIYIHGSSGIGKTSLALELIEQI-I-KRAKE-YNY--NW-KMYSAGT-KN----IFD

EY-FGEEIILLDDPRY---DS-L----LPADWLKLLDPLNK-SH-LSARYKNKLVIG-R---IIIITNYKSL

KSFFG--KI-----------------QH-EDLNQYIRRFNNVLEI---SKK--NEKSEKD---------RFF

NLSQIQELREAHDYLG------ESSLLFGEEKVYS-TDDKE--AFINHVL-EYYIFPRIL-------P----

ETKSAPLDQ---------------------------------------------------------------

-----------------------

>pCRESS6|WP_003104234.1

M----SRK------------------------------------RTTTYIFEQQLHAE---------YWDWE

DDKKALFSNWER-NKTKIFQEIHRRIKTLEEDIP----AKVAFIVHDKDI---------KY-G---IK--P-

VEPHIHAYIEF--ASRRDLSVLASTLG-----LLP-QYIE--PSG----QG--K----YGKVNSKAYLIHAK

----------------S----PD--KHQYAPSEVE-TFGT-----FD----YVAFIEDNRADFSKRYAVAKR

EK--SDES-LD-KVFQEIINGNL-TEDD----IFADEEL-------TFLWS--YNQTRLDEAFRAYGKIASK

RTLRELENGEF-KPTIIYVHGSSGIGKTTLALEVIEEI-I-KRAKE-QGL--NW-KMYSAGA-KN----IFD

EY-FGEEVILLDDPRS---DS-L----LPADWLKLLDPLNK-SY-LSARYKNKLVIG-R---LIVITNYQSL

KSFFG--KI-----------------QN-EDLNQYIRRFNNVLEI---SKKKGNHDKEKD---------RFY

NLSEVKELSYNDYYQMGYDQ--EIQLHFGEEDIYC-TDNKA--DFINRVL-DEHILPRIL-------P----

QIKKRPQNPAREKGNA--------------------------------------------------------

-----------------------

>pCRESS6|WP_039670385.1

M---AKRK------------------------------------QTTTFIFEQQLKGE---------YWDCS

DEKKALFEDWKS-NKQEIFQEIYNRVKVLETDSS----LKIALIVHDKDK---------TF-G---NN--L-

VFPHLHGYIEF--SNKRDLSVLALNLG-----LYP-QYIE--PSG----RG--K----YGKINSKAYLIHAK

----------------S----PD--KYQYQPSEVE-TFGT-----FD----YERFIQENLDSFTKQSAKVKR

EQ--SDES-LD-MIFQEIIKGKL-TEDD----IFADEKL-------TFLWA--YNQQKFDEAFKAYGKISAK

TTLRQLENGEF-KPTILYIHGQSGIGKTSLAYDLVAEI-V-LQAEK-QDL--NW-KTYNAGS-KN----IFD

EY-FGEEIIVLDDPRY---DS-L----LPSDWLKLLDPLNK-SY-LSARYRNKLVIG-R---VIVITNYMSL

SEFFR--QI-----------------PK-EDINQYLRRFNNVIEI---TKSGND---------------RQY

NLSEIRELSVPNSDG-------YKFLNFQEFEVMS-STDKK--EFLDKIL-EEYIYPRIL-------P----

DKQKIQLVKNESDCKK--------------------------------------------------------

-----------------------

>pCRESS6|WP_018380019.1

M---TKRK------------------------------------QTTTFIFEQQLQSD---------YWDWS

DDKKDLFDDWEA-NKRAIFQEIYDRVKTIDEENP----IKIGLIVHDKDV---------SF-G---SR--L-

VKPHVHGYIEF--KTKRDLNILALSLG-----LLP-QYIE--PSG----KG--R----YGKINSKAYLIHAK

----------------S----PD--KYQYEASEVE-TFDT-----FD----YEQFIAENKEDFAKQSATRKR

EK--SDES-LD-LVISKVQKGEL-SYQE----VMEDDEL-------AFLFA--NNQQKFRESFNFFGEREAF

LRLKSLERGDY-QLTVLYIQGEPDVGKSTLAKEIALKV-K-AKMND-IGL--RG-DIYSASS-SN----PFD

NY-YGEEILFLDDLRE---YN-L----SASDWLKLFDPLNS-AR-MSARYQNKLVIP-R---LVIMPVYKTP

KTFFG--EV-----------------QA-EDLNQFLRRINFLLDI---SLKHETDD-------------RLY

NVSELVKRKAVDFYERKDGS--TVVLNFKYEDMFC-SDDKE--WFVNKLL-EDCVYPRIL-------P----

KKVKDVTND---------------------------------------------------------------

-----------------------

>pCRESS6|EOB33201.1

MEKMAKRK------------------------------------QTTTYIFEQQLKPD---------FWDWS

EDSKKLFLNWEN-NKIEIFKEIYERVRLISESED----LKIALIIHDKDI---------SY-G---TK--L-

VEPHIHGYIEF--SNKKDLNVLALSLG-----ILP-QYIE--SSG----RG--K----YGKINSKAYLIHAK

----------------D----KD--KYQYSASDVE-TFDT-----FD----YEAFINQNREDFEKYAATKKR

EK--SDES-LD-LTLSKIQLGEL-TYND----VMEDDSL-------AFLFG--NNQQKFREGFNFYGERQTF

LRLKSLERKEY-QMTVIYIQGDSDIGKSELAKNIALQA-Q-AKLNE-VGL--RG-DIYSASS-SN----PFD

NY-LGEDILLLDDLRE---DT-M----RASDWLKLLDPLNS-AR-MSARYQNKLVVP-R---LIIMPVYMSP

KLFFG--RI-----------------KA-EDLNQFLRRINFLVDV---SLKHGSEVE------------RLY

NISEVVKRKGIDFYEKKDGT--IAVLNFRYEDLFC-AHDRD--VFIEKIV-EECICPRIL-------P----

KEVKDVTND---------------------------------------------------------------

-----------------------

>pCRESS6|WP_044671103.1

M---GKRI------------------------------------INKRFLFEQQLKSK---------FWDWS

IQDKQLFDNWET-NKGKIFRLIFDRVRTLVEE---DEFVEFAIVIHDKDI---------SY-G---TK--L-

VEPHIHGYIDY--PKRIDLSKVASALG-----VER-ERIE--PKS----KG--A----YTRINALAYLIHAK

----------------D----KD--KYQYPVSDVE-TFDT-----LD----YETFINQNKEDFENFSATRKR

EK--AEER-LD-LVLSKVQSGEL-TYLD----VMKDDKL-------AFLFA--NNQQKFRESFNFYGEREAF

LRLQALQRGDY-QLTVLYIQGKPGIGKSTLARDLALET-Q-KRLNA-KGL--KG-EIYSAGS-KN----PFD

NY-YGEEILLLDDLRK---DS-I----SGTDWLKLFDPINS-AR-MSARYQNKLVVP-R---LVILSAYMSP

KTFFG--QI-----------------ET-EDLNQYLRRINFSSEI---SLKYGMDD-------------RYY

SVAQVKEHKEVGYYQRSDGS--SIVLNFDFEDLFS-MQDKS--QFITKLL-DDYIYPRIA-------PCEVG

KNV---------------------------------------------------------------------

-----------------------

>pCRESS6|WP_003048523.1

M---TKRI------------------------------------RNRRFLFEQQLKTS---------FWDWS

IQEKQLLDDWEK-NKDKIFRLIFDRVRTLVEE---DEFVEFAIVVHDKDV---------SY-G---TK--L-

VEPHVHGYIDF--PKQIDLSKVASALG-----LER-ERIE--PKS----KG--A----YTRINALAYLIHAK

----------------D----KD--KYQYPVNDVE-TFDT-----LD----YETFINQNIEDFEKYAATRKR

EK--SDES-LD-LVLSKVQKGEL-NYLE----VMEDDEL-------AFLFA--NNQQKFRESFNFFGERETV

LRLKDLKKGNY-QLTVLYIQGEPGIGKTHLANELILEV-S-KRLRE-NGL--KG-EYYPASS-KN----PFD

NY-YGEEILFLDDLRE---DS-L----SASDWLKLFDPLNS-AR-MSARYQNKLVVP-R---LIVMTAYMSP

KQFFG--NI-----------------KT-EDLNQYLRRVNFSTEI---AKKHGMED-------------TFY

SVSEVKKNKANDHNQRSDGS--SVVLNFNYEDLYS-SQNKD--EFITKLL-EEYIYPRIL-------P----

KNVKDVTNG---------------------------------------------------------------

-----------------------

>pCRESS6|WP_039694464.1

M---AKRI------------------------------------RNRRFLFEQQLKSE---------FWNWS

IKEKQLLDDWEA-NKNEIFRTIFDRVRSLVEE---NEFVEFAIIVHDKDI---------SY-G---TK--L-

VEPHVHAYIDF--PKQMDLSKVASALG-----VER-ERIE--PKS----RG--A----YTRINALAYLIHAK

----------------D----KD--KYQYPVSDVE-TFDT-----LD----YETFINQNREDFEKYSATRKR

EK--SDES-LD-LILSKVQTGEL-NYID----VMENDNL-------AFLFA--NNQQRFRESFNFFGERETV

LRLKDLKQGNY-QLTVLYIQGKPGIGKTHLANEIILSV-A-EKLRE-YDL--KG-ECYSASA-KN----SFD

NY-YGEEILLLDDLRE---DS-L----SPSDWLKLFDPLNS-AR-MSARYQNKLVVP-R---LIVMTAYMSP

KQFFG--QI-----------------ET-EDLNQYLRRVHFSTEI---AEKHGMKE-------------RFY

SVSGVAKNNANDYYQRSDGS--TVILNFGYEDLYS-SQNKD--KFINKLL-ENYIYPRII-------P----

KENKIINYKERQYVCS--------------------------------------------------------

-----------------------

>pCRESS6|WP_000746010.1

M---KKRI------------------------------------INKRFLFEQQLKPK---------FWDWS

VQDKQLLDDWET-NKDKIFRLIFDRVRTLVEE---DEFVEFAIVVHDRDI---------SY-G---AK--L-

VEPHVHGYIDF--PKKFDLSKVASVLG-----VER-ERIE--PKS----KG--A----YTRINALAYLIHAK

----------------D----KD--KYQYPASDVE-TFDT-----LD----YEAFINQNKEDLEKYAAKKKR

EK--SDES-LD-LVLSKVYKGEL-TYFD----IMKDNNL-------YYLMA--NNRQKFLEGFDIFGERESV

LRLEALQNGEY-DLTVLYIQGKPGIGKSTLARDIALEV-Q-GALEN-VGL--RG-GSYSASS-KN----PFD

NY-SGEEILILDDLRE---DS-L----APADWLKLFDPINS-AR-MSARYRNKLVVP-R---LVIMSAYMSP

KQFFG--QI-----------------QE-EDINQYLRRVNYSSEI---ARKHGMEE-------------RFY

SVSEVRENRENGHYQRPDGS--SVVLNFDYEDLFC-SQDKD--DFIRKLL-EDCIYPRIL-------P----

KKAKDVTND---------------------------------------------------------------

-----------------------

>pCRESS6|WP_019299400.1

M----NKI------------------------------------RIKNFVFEQYFDQE---------FWDWE

EDKKEWFMNWKE-FKENIFQEIYDRLKFYDTE--DKNIK-VAMIVHDKDK---------KW-N---DK--L-

IEPHIHVYVEL--PTKRSIERIADRIG-----ISQ-HFIE--PKG----KN--R----FFPFNEKAYLIHAQ

----------------Q----PD--KYQYEVNEVE-TFET-----ID----YEQFVLENEKEFLKRSATVRY

KT--IDES-LD-LIFDKVLFGKI-TYDE----IMEDETL-------YRLYA--NNEQKFISAFNSFAQYKAR

KTLKALKNKEF-KMSVIYIQGKSGIGKSHLAGEIVEKI-C-EESEK-LSY--EGVSVYSASA-SN----PFD

EY-KGEEILLLDDLRP---DS-L----ERADWLKVLDPMNK-SR-ISARYRNKAIAS-R---VIVLTNTETA

EQFFK--NI-----------------KN-EDLDQYIRRINLTVGI---DEKQFPR-FDY-D--------SFY

RLSESKKLLEPKRERVNEDE--FIELRYGFEPVFS-TENKE--EFLDRLV-HKEILPKSF-------P----

V-------------K-------G-------------------------------------------------

-----------------------

>pCRESS6|BAM66968.1

M----KKK------------------------------------RIKVFVFEQYFDSE---------FWDWD

E-KKSWFQNWEE-NKDKIFQEIYDRLKFYDTP--ETQLK-IALIVHDKDR---------KN-N---GQ--L-

IDPHVHVYVEL--PTKRSLELVADRIG-----ISQ-HFID--ANG----RN--K----YFSFNQKAYLIHAQ

----------------Q----PT--KHQYELSEVE-TFGT-----ID----YEKFILDNKDDFLKRSATVRY

KE--INES-LD-LIFDKVLFGQI-TYDE----IMEDDSL-------YRLYA--NNEQRFISAFNSFSQYKAR

KTLKALRNCEF-KLSVVYIQGNSGIGKSHLAQEIVEKL-C-DESEQ-LGY--NGVSIYSASS-SN----PFD

EY-KGEEIILLDDLRP---ES-M----ERADWLKVLDPMNR-SR-ISARYRNKVIAA-R---VIVLTNTETA

ESFFK--NI-----------------KN-EDLDQYVRRINLNVGI---TEKQFPR-FEY-D--------NYY

NLSETKKLGEPKKERISANE--FLERRYGFQPVFS-SDDKE--LFLKKLI-FDELLPKSF-------P----

K-------------DKGVIRDVS-------------------------------------------------

-----------------------

>pCRESS6|WP_050484011.1

M----EDK------------------------------------RIKQFVFEQQLKAE---------YWDWE

KDRLKIFEHWEE-NKLLIFEEIYRRLKTLESE--KVKLECIAIIVHYLDK---------ND-L---NE--F-

IFPHVHLYGKY--TDKRTLARIAKVLG-----IKE-QYIE--PKN----GN--K----YFEENQLAYLTHAQ

----------------Q----PD--KYQYPTHEVE-TFGT-----FD----YSNFILINTKKFEKQSATVKR

KR--TDES-LD-LINQQILKGEL-FLED----ILADDDL-------FLLYS--NHKLQFKQAFDSYAERLAF

KNLKDLTTGKY-KLTVMYFQGKSSLGKSYLARTIAQKV-R-EYAEE-NKF--KS-RIYSASS-SN----PFD

DY-YGEDIILLDDIRP---DS-L----RKAGWLKLLDPINT-SR-MSARFTNKQVVP-R---LILITNTQLP

EQFFN--IF-----------------KD-EALDQYIRRINFCTIL---SEKQQGKGYEG-G--------VYY

QLSQTKRLFSPKVRNISAYE--KEEINFDLEAIFS-TDNQE--EFTEKLL-ARYLLPRIY-------P----

KTEKD--FDF---LKSKIIEKAD-------------------------------------------------

-----------------------

>pCRESS6|WP_058223604.1

M---IEEK------------------------------------RIKQFVFEQQLKAE---------YWDWE

DEKLELFDDWQA-NKALIFEEIYRRLKTLESD--KVKLECISLIVHSLDK---------ND-L---YE--L-

VFPHVHLYGKY--SDKRTITRIAKVLG-----IKV-QYIE--PKD----KN--R----YFEENQLAYLTHAQ

----------------Q----PD--KYQYPPLEVE-TFGT-----FD----YSNFILSNAKKFEKQSATVKR

KR--TDES-LD-LINQQILKGEL-FLEN----ILADDDL-------FLLYS--NHKLQFKQAFDSYAERLAF

KNLKDLTTGKY-KLTVMYFQGKSSLGKSYLARTIAQKV-R-EYAEE-NKF--KS-RIYSASS-SN----PFD

DY-YGEDIILLDDIRP---DS-L----RKADWLKLLDPINT-SR-MSARFTNKQVVP-R---LILITNTQLP

EQFFN--IF-----------------KD-EALDQYIRRINFCTIL---SEKQQGKGYEG-G--------VYY

QLSQTKRLFSPKVRNISAYE--KEEINFDLEAIFS-TDNQE--EFTEKLL-ARYLLPRIY-------P----

KIEKD--FEF---LKSKMTESSD-------------------------------------------------

-----------------------

>pCRESS6|WP_061343647.1

MA-QSKRN------------------------------------QLKSFYGTQQLEQE----NNEETFWNFE

DDNISLIKFPRK-NQRRIFEIIATRFRKSDNG--N--IAKIAFVLHDKDK---------DD-T---GN--F-

IKPHVHWLLEL--KNKRDLDEIAYKFF-----VHP-QQIE--KGS----KG--K----NCFLGRIGYLTHQA

----------------E----PD--KFKYDVHDVE-TFGT-----FD----YADYISKNSAYFKKRLAFNRK

QQ--AKMD-VD-YYLQQVQQGIL-FLDD----IFLDLNL-------YNVYA--NNKQKFREAFDAYSELNSF

RTNRDKRLGIF-DFTTIFIYGRSGLGKTTIAMAILDRL-K-ELAKS-DGI--KW-RSYSGSA-KN----AVD

DY-KAEELILFDDLKQ---DS-F----LIADWLKILDSRNE-ST-ISGRFHNKPLSA-R---LIILTTIESP

FKYFD--FG-----------------KD-EPKEQFIRRLSYIINV---SSFNMDDEQMIK---------TNF

LINTPNKQ-----------------DNYHLKEKYK-EIGMD--NLLNSEWINEIYERIKG-------E----

GRK-----------------WIT-------------------------------------------------

-----------------------

>pCRESS6|WP_017371219.1

MTIKAKTI------------------------------------QAKTFFGMQYLEKE---------YWHFD

EETLSLMTDPRK-NQKEIFALVCEKFRQDYQD--D--IVTLAIVLQDKDI---------NE-N---GK--L-

EAPHLHWAIHL--KERTTLNKIAKAFQ-----VEP-QYIE--TGN----QG--K----NAMIGRLAYLTHQT

----------------E----PD--KFHYDPQDVE-TFGT-----FD----YVNFVNNNKMKFKKLLATKRY

RA--TKYE-LN-HLLQQVQVGKL-FIED----ILSDEKY-------YFVYA--NNLAKFKEAFEAYAQRNSL

LTIQDRVAQKF-EFTSIYIYGKSGSGKSEIAYDILKQI-E-LLSQE-VGL--RW-HSYFGGS-KN----AVD

DY-KGEELLLFDDVRP---ET-F----SPADWTKILDYKNK-SA-LSGRFHNRPLSN-R---LVLMTNTQSP

FEFFK--F------------------EN-EPIEQYLRRLTYVIRV---EAVGTMDEEMKQ---------TKF

TILAPDRA-----------------CNYHLREVYQ-LTGKA--ELYNSKWLDTLFKQLMN-------K----

EKKPSLPTLDGKSDSTI--CEVI-------------------------------------------------

-----------------------

>pCRESS6|WP_014571792.1

M-VQQTQI------------------------------------QNIRFHITQFLKKE---------YWQNP

DISAVDFK-----DSIAFQKQIFMALIPL------G-LVKFASIIHDKDL--------QLD-Q---VT--P-

KPPHIHAIVEF--EKKKDINVVALALG-----LEP-QYLD--TAK----RG--R----YAEENLLAYLIHAK

----------------D----KT--KYQYSPKEVS-TFGT-----FN----YMAYEEEHRKRWKNQAIVAKG

KK--KDEQ-LP-ILLEAIREGAL-NREQ----VLENPDS-------LFLYQ--RHIMEFQAAFEGYLLKAEA

ETKKAIKSGEL-KLTTLYIYGLSNAGKSRFAETLGETL-K--AKVR-GCE--NW-TSYTTAS-TN----PFD

EY-TGEQIVILDDLRA---KS-M----TAENWLKILDPERI-SK-SAARYHNKIISS-H---LIIITAPISP

QSFFH--QI-----S-----------ES-EELNQFMRRLSLTIEI---SQGKKE---------------RLY

TVNSIERKKNNGR---------PTFLEKPLHEKKS----LN--KALGSCI-TKLLNND--------------

KTINKKPSDIKAATLSDDSSPLG-------------------------------------------------

-----------------------

>pCRESS7|CDE19587.1

M-------------------------------------------KLKQYEVVTQPE-----------YL---

----------------KS-P-LQDIL----RK--YRTIKQWAYILHDKDKD---------------------

ASSHYHIYINFG-QQTVDSKDVAGWFG-----IPE-QFVN--KV---------E----GRKTDMLMYLTHS-

-----------------ND--SQQHKHQYDFSEVV-AN-------FD----FKSEIEQA-------------

-----------------KIIGDF-EKYS----Y--AQQLDYIHSLAVSEQP--KCFDRLQKLWKLQCQWLSL

-------NSDR-NLKVIFVTGKSGTGKTYFARKYMRA----------HE---LD-CYVSSSS-ND----PLD

MY-MGQKGIIFDDLRD---EA-F----EFADILKLLDNNTS-TA-MKSRFTNKVLNC-K---VMIITSFIPI

KYWYK--SVRY---------------SC-DGIEQLYRRINMYVHI---TEDEIV-VYD-GLSDKGD------

---PHGLGKVYV---------------NEVKKLKEE-----NRKQREDAFGDFD------------------

FLTEKKDKF---------------------------------------------------------------

-----------------------

>pCRESS7|CCZ68460.1

M--RKR-----KTNQGNL--------------------------SMRRCEIVSNLE-S----EDGEKLF---

----------------DI-ERMKQVLEE--KS--KTCIKEFSYIIHDKDVYTEEDERKNEK-Y-KCGE--L-

KPKHIHLLLRF-FNQPQKLKNIAGWFQ-----IPP-NFVS--KI---------H----NRWDSAVLYQIHA-

-----------------NC--PE--KYQYDISEVT-AN-------FK----IENVINNF--MKRNSID----

-S--I----LM-D----ILNGEI-PEYQ----R--SV-IP------PLFRV--HYAREINEAFRCRVQNLQE

------TVKSR-KMECIYITGSSQAGKTTLAKKIAEE----------KG---LP-YYISSSG-TD----FLG

EY-ALEPCVILDDIRP---SS-I----NLSELLKLLDNNTV-SA-VKSRYKNKCLNC-K---LLIITTVLDI

ETFYH--NVF---S-----------EED-EPMIQFKRRCGTHLRM---NKERIY-ISR--WDSLKK------

---EYTEETEYL---------------NDILDR-YMPKEDQTEQDVINYVSE-T--MPFL-------K----

QADESEKMHGF-----------------------EIIDDLE-------SPFK--------------------

-----------------------

>pCRESS7|SCG87263.1

M--RQKYKKENKNKSDSD--------------------------SKRRIEIVSRLT-D----DNGKILF---

----------------DI-NTIPDII----KK--HSCIEKYAYIIHDKDVYTEEDELANPN-H-KKGT--L-

KPPHIHLYLHF--NSPQHIPQIAKWFN-----IGK-QFLE--VC---------H----GREIDVLAYLVHLT

----------------PSS--AG--KYQYNPLEIV-SN-------FD----VQEALNNT--LHQADID----

------------LICQKIMSGEI-CEYN----K--VQMIG------PQILF--DSGYKIERAFKIQQETFEE

------NCKNR-DTLAIYIEGPAQVGKTTFAKTIAKD----------KS---FA-YYISSSS-ND----LLG

DY-KQEPVVILDDLRP---DS-I----GISDLLKLLDNHTA-ST-FKSRYKNKYLNC-R---IIIITSTLEI

SRFFE--EVA---A-----------GKA-EPIEQLKRRCRIHIRM---NEKKIY-ISC--WDAAKK------

---DYVLARIQD---------------NDIPKF-YHVDKELSRKEINKFVDD-I--IPRS-------I----

GRRPNNGEIIL----------------------------------------V--------------------

-----------------------

>pCRESS7|YP_006961027.1

M-------------------------------------------KLKICEIVINKT-----------LI---

----------------TK-TKIETILE---AK--TKAIQNYAYILHDKDTYQNDKEAQLNG-K-KVGD--I-

KAPHWHIYLRF--NYAYDTKHISQWFN-----TQE-NFVS--KI---------K----GRFSDALMYMIHA-

-----------------NR--SD--KHQYDEKEVV-SN-------FD----WKSEAQQDIFNRKYKMD----

-A--R----LK-EIIDKIESGEW-KRYD----L--INKIN------GYENN--IYYSAIKKAFERRIDFLE-

-------EMKR-EMECVFITGMSGSGKTTLAKKIAED----------NG---YK-AYVSSAS-ND----VLD

NY-KGQECIILDDLRS---YC-L----VLSDLLKMLDNNTA-SS-VKSRYKNKVLEC-K---LIIITTVKSI

DDFFD--DIF--NK-------------D-ESITQLKRRCKFHIKI---DSKYIY-TSV--WNDVDK------

---KYNKPYKKP---------------NNLLNE-FQI-KRLSEKEEKDKIKK-V--LKTD-------L----

-DEDS-------------------------------------------------------------------

-----------------------

>pCRESS7|YP_003617079.1

M-------------------------------------------KLKICELVINQK-----------LI---

----------------TK-TKIETILE---TK--KKAIQNYAYILHDKDIYQNEKEAQLNG-K-KVGD--I-

KAPHWHIYLRF--NYAQDTKHISQWFN-----TQE-NFVS--KI---------K----GRFSDALMYMIHS-

-----------------NR--FD--KHQYDEKEVV-SN-------FD----WKSEAQQDIFNRKYKMD----

-A--R----LK-EIIDKIESGEW-KRYD----L--ITKIN------GYENN--IYYSAIKKAFERRIDFLE-

-------EMER-KMECVFITGMSSSGKTTLAKKIAED----------NG---YK-AYVSSGS-ND----ILD

NY-KGQECIILDDLRS---YC-L----GLSDLIKMLDNNTA-SS-VKSRYKNKVLEC-K---LIIITTVKSI

DDFFD--DIFK-DK-------------D-ESIIQLKRRCTYHIKI---DSKYIY-TSV--WNDVKK------

---KYNEPHIKP---------------NDLLDE-FQI-KELTEQEEKEKIKK-A--LKID-------L----

-D----------------------------------------------------------------------

-----------------------

>pCRESS7|ABC65805.1

M-------------------------------------------KLKICELVINSD-----------KI---

----------------NK-TKIENILE---LK--KKAIQNYAYILHDKDIYQNEKEATLNG-K-NIGD--L-

KKPHWHIYLRF--NYAHDTKHISQWFN-----TQD-NFVS--KI---------K----GRFSDALMYMIHA-

-----------------NR--SD--KHQYDEKAVV-SK-------FD----WKSEAQQDIFLREYKID----

-S--R----LE-EILSKIQSGEI-KECN----S--TNHIS------IIENN--IYSSAIEEAVKYRNNTLK-

-------GMDR-QMECVFITGLSGCGKTTLAKKIAKN----------KK---YQ-TYISSGS-ND----VLD

DYSRGEECIILDDLRS---NC-L----GLSDLLKMLDNNTS-SS-VKSRYKNKVLEC-K---LIIITTVKSI

DDFFE--DIF--KK-------------D-ESIIQLKRRCKLHIKI---DSKYIY-SSI--WNPLEM------

---KYDLIDKKP---------------NNLLNE-FQL-KTLSKKEAKEFIKQ-I--TNT-------------

-DDI--------------------------------------------------------------------

-----------------------

>pCRESS7|WP_011161011.1

M-------------------------------------------KLRICELVINSN-----------LI---

----------------NQ-SKIENILE---AK--KNAIQNYAFILHDKDIYQNEKEAQLNG-K-KIGD--L-

KTPHWHIYLRF--NNAYDVKHIAQWFN-----TEE-NFVS--KI---------K----GRFSDALMYMIHA-

-----------------NR--TD--KHQYDEREVV-SD-------FD----WKSEAQQDIFLRKYKID----

-T--R----LK-DILTKIHSGEI-KEYN----I--TNYIS------IIEHN--IYSAAIEKAFKYRTNTLK-

-------GIER-NMECVFITGMSGSGKTTLAKKIAKD----------KN---YQ-TYISSGS-ND----VLD

DY-QGQECIILDDLRS---NC-L----GLTDLLKMLDNNTS-YS-VKSRYKNKVLEC-K---LIIITTVKSI

DDFFE--DIF--KK-------------D-ESIIQLKRRCKLHISL---DSKYIS-YSM--WNPVKM------

---EYEKPFKKP---------------NNLLNK-FQI-KALSEKEQKEFIKK-I--TNID-------L----

-DED--------------------------------------------------------------------

-----------------------

>pCRESS7|ABC65794.1

M-------------------------------------------KLRICELVINAN-----------KI---

----------------TK-SKIENILE---LK--KKAIQNYAYILHDKDTYQNEKEAQLNG-K-KIGD--L-

KSPHYHIYLRF--NYAYDTKHIAQWFN-----TQD-NFVS--KI---------K----GRFSDALMYMTHA-

-----------------NS--SD--KHQYDEKAVV-SD-------FD----WKSEIQKTNLNKQFKMNY---

-A--R----LK-EINSQIISGEI-KEYN----I--DERIN------VDEYY--VYSAAIEKAFKYRVITLK-

-------RIKR-QMECVFITGQSGSGKSTLAKKIAKD----------NK---YN-AYISSGS-ND----ILD

DY-RGEECIILDDLRS---NC-L----GLSDLLKMLDNNTA-SS-VKSRYKNKVLEC-K---LIIITTVKSI

DDFFE--DIF--RK-------------D-ETIIQLKRRCTYYIEI---DSKYIY-YSY--WDSIQN------

---KYDLLNKEP---------------NNLLNE-FKI-KSLSKQEARLKFKK-S--LKIN-------LPTKD

KDDI--------------------------------------------------------------------

-----------------------

>pCRESS7|YP_001966814.1

M-------------------------------------------KRRICELVIKAD-----------LI---

----------------KQ-TEIEKVLE---SK--KKVIQSYAFILHDNDKYLNEKEAKENG-K-SVGD--Y-

KIPHWHIMLRF--HQSQEFKYIAKWFN-----TTE-NFVS--QI---------K----GRFTDALLYLTHA-

-----------------NR--AD--KHQYQNHQVV-SN-------FD----WESEANQDTFMRKYKLD----

-T--R----LV-DILNKINSGEI-KEYN----I--TNHIT------IIENN--IYSAAIEKAFKYRNSKLR-

-------EMDK-KMECVFITGQSGSGKTTLAKQISKN----------KN---YT-PFISSGS-ND----VLD

GY-KGQECVILDDLRA---DC-F----GVSDLLKMLDNNTA-SS-VKSRYRNKYLEC-N---LIIITTTKTL

NAFFD--TVF--NK-----------SDD-EDVKQLKRRCRIHIKL---DLKNIT-YSI--YNPNTS------

---EYEEVEKTP---------------NRIREI-FKT-KEQSKEEKKEFIKS-T--LNVE-------L----

------------------------------------------------------------------------

-----------------------

>pCRESS7|KXT29039.1

M--NP-----------GF--------------------------RLRRCEIVIKED-----------LI---

----------------KK-DFIDKVLK----K--KKIIKKFAYILHNRDVLED-------------GS--L-

KNPHYHIVLVL--KTPYDVEYIASWFK-----TSS-NFVE--KI---------K----GNMSDILNYLTHK-

-----------------ND--LS--KFQYEDYEVV-SN-------FN----WKKTRDEGS-IKKYRLD----

-K--R----LK-DLLTKIMNGEI-KEYN----I--SKKIT------VYENN--IYATALERAFKFRTNFLK-

-------GVKR-NMDCIFITGKSGSGKTTYAKYLADQ----------KG---YS-IYVSSGS-ND----ILD

DY-QSQECIILDDLRP---EC-L----GLSDLLKMLDNNTA-ST-VKSRYKNKVLEC-N---LMIITTTIPI

KKFFD--LVFY-KK-----------EKK-ETIVQLQRRCKVHIRM---DKKNIY-YST--FNSIKD------

---CYEKEFVRE---------------NKILKE-FKI-KELNEQEQLEKI--------IE-------I-TGD

KDIVDAISSDEKGKIKKIKPK------SHHKPL--KLEKHK-------LLRK--------------------

-----------------------

>pCRESS7|ODR34583.1

M---------------PV--------------------------KLKMCDIVSDTT-----------HL---

----------------SL-ETIQATGD-------KACIRHYAYILHDKDKNKD-------------GT--D-

ATPHYHVFLRF--EDTQDTKYVAKWFG-----IAE-NFVG--KI---------N----GKWTDALLYLTHE-

-----------------NA--PS--KFQYPETDVV-SN-------YD----WKKEKNAK--LTSQSLK----

-A--R----EA-EIVSLIANGTI-KKYN----Y--NEYIT------PVEYV--RLNASIKAAMNYRADMLSH

-------NHNR-QLEVVYIVGGSGCGKSTYAKRLAEE----------KG---LS-CYVSSGS-ND----VLD

DY-GGQDCLILDDLRP---SC-L----GLSDLLKLLDNNTS-TS-VKSRYKNKILEC-S---LIIITTVKEI

DEFFS--KVF---E-----------HED-EPLKQLKRRCRTMIRL---SADTIE-ISV--YNDTSD------

---RYDPVNTYS---------------NPIAEL-YKT-EPLTQAELTAKVEKDF--CISG-------M----

EAYVPPAPVVSGDGFMEIVPD------GEPPFK---------------------------------------

-----------------------

>pCRESS7|CCZ93342.1

M--ENEKAEKKKKDRTPK--------------------------TWKTCEIIQQLE-----------YM---

----------------SA-EDVGSGLD-------HNAIKDYAYILHDKDVNDD-------------GS--P-

KAAHWHIYIRF--KDSTPTDSICKWFG-----ITS-NYIG--RI---------Q----GRFADALAYATHR-

-----------------NV--PE--KYQYLDEEVK-SN-------FD----FVKERDTA--RSKEADK----

-Q--R----KA-EISDLIISGVI-REYN----Y--TDYIT------IQEYD--RFRKSIDNAFKYRLDKIK-

-------GENR-DMEVIYIFGDSSCGKTTYAKELAAQ----------NE---YS-CYVSSGG-ED----MLD

DY-KGQDCVILDDLRA---ND-I----NFSSLLKLLDNHTQ-SM-VRARYHNKFLEC-K---LMIITTSKSM

EELFR--ELP---G-----------SDN-EDITQLRRRCKLYIKM---TPLTMT-IRM--WQPESL------

---KYLYVQTLD---------------NPVHGR-YAA-RDKSLDEAKAYVNN-V--LFFP-------K----

VA--TAETMAKGEGFRELTDD------EADLFS--DQMKL----------PL--------------------

-----------------------

>pCRESS7|CCY61699.1

M--KEKKGE-------PS--------------------------SWRTCEIVQQLE-----------YM---

----------------SQ-EDVEAGLD-------HNAVQDYAYILHDKDVHED-------------GT--P-

VAPHWHIMIRF--KRPVQTESLCKWFG-----IKS-NMIG--YI---------L----GTFGDAVAYLTHR-

-----------------NK--PE--KYQYLDEEIK-SN-------YD----FKVEVEKA--LSKKKAS----

-Q--R----KE-EIIELIRSGIV-REYN----Y--TEYIT------ALEYD--KFKRAIDNAFTYRRDTLK-

-------SLDR-HMNVIYIYGGSGTGKTTYAKQLAIN----------KG---LS-CYISSGS-ND----PLD

GY-KGQDCLILDDIRP---GD-F----LLSDFLKILDNNTQ-ST-VKSRYKNKLLEC-Q---YLIVTTSFDI

PVFFD--LLL---D-----------SEG-ESVKQMERRCTLKIQM---NTSTMT-TYV--YQPVSG------

---KYKKVSILK---------------NPVSEV-YNK-RDLTDEECQAYVDS-L--LLPG-------K----

EA-----SLSDLGFKADVPPD------VALAFEQWNQEEFK-------QDNL--------------------

-----------------------

>pCRESS7|WP_019282500.1

M--DSKK---------II--------------------------TMRQCELVSNIE-----------HL---

----------------DL-EYIKEKLESLATN--GKSVTDYAYIVHDKDTYEKDGETSDGK-KFKKGD--L-

KAPHVHLMMKF--KSPQKVHCIAKWFK-----VKD-NNIN--KI---------K----SKWVSALRYLIHA-

-----------------NH--PE--KHQYNVDEVI-AN-------FD----YSEEKDKIN-THNNKKK----

-Q--R----KE-EIVNNISKGEW-KALD----LYNTDNIT------ELEFV--EFSADINKALNYRQTFLQL

------NNKGR-DMNVIYISGGSGSGKTTLAREYAEK----------KG---HS-IYVSDGG-KN----PMD

NY-MGQDCIILDDFRP---DV-M----GFSDLLKLLDNHTS-SM-VNARYYNKFMEC-K---LLIITTIDDL

PDFFK--KMQ---D-----------TKG-EPIKQFERRCKTKMLV---DSDTVR-FYS--Y--AID------

---KYEFSGLIP---------------NPLKDREYAPNASVDVDDFAEAFGV--------------------

-------------------------------SDQISKSEIK-------VVGF--------------------

-----------------------

>pCRESS7|WP_002578150.1

M--SKAK------KVTDP--------------------------CRVICEFQNQIE-----------YT---

----------------SE-SSFKNIVNNL-YK--RGIILDYAYIVHDKDTYTEQDELDDIK-N-KAGN--L-

KKTHIHGMLRL--NNSYKFSTIANWFD-----VTA-QRIR--KI---------E----TSYAAACAYLIHR-

-----------------NN--PE--KFQYDPSMVI-SS-------FD----YNEKAQLTNENHMKKLKK---

------------RILEEVEAGTL-RGYN----F--HENYA------FSDRV--ALRSYLNNAID--EIIKTK

----LNSNKER-DLEVIYIHGSSGAGKTTYAKMTAKA----------RG---LI-YATSGED-RD----PVE

TY-DSHPCMILDELRP---SS-M----KLTSFLKLVDNNTE-SM-AGARYHGKAFEC-K---LIIITSILPI

EEFFK--NLQ---A-----------NDN-ETAIQIKRRCKTMFDM---DRDNIE-IYE--WDDCNL------

---EYFYCGRKK---------------NPIPTL-YHI-EPKSIEELRAKACEVAGIDLSE-------LDISP

KEPEVIGIVG--IPFEAVYPTIPPDIATKIKHNPWLYLNEHPEHKAIPLNQL--------------------

-----------------------

>pCRESS7|CUN62864.1

M--SEKTVT-----------------------------------TPTLCEVQTQIP-----------FL---

----------------PK-EKMLSIIAEHTTA---GHIKEWAYILHDKDIKED-------------GT--P-

KEPHWHIELRL--TRGRRLTDIASWFG-----LPT-SCIQ--TSK-------------GRYEPMLQYLIHE-

-----------------NS--SE--KYQYAESEVV-AN-------FN----YSERMQVIRDIQAKKN-----

----AKDKRIS-EIVELIANGTI-REFN----I--DEFVT------VREYD--KYRSHIKNALEYRSIILE-

------KQNTR-NMEVIYIYGKGGTGKSSFAEAIAKE----------RG---FS-FKRSASE-RD----PLA

TY-KGQDSFALDDVRG---NT-F----EFQDWQGVLDNFQD-RP-GSSRFHDKHFEC-K---LLFITTTDSA

EDFWK--EMSA--------------KPN-EDSHQFFRRIKTVIHM---TGDEIL-CKR--YDEETH------

---SFGKEHIME---------------NDTYSRFDVHSE--TDEEQKEKLAHSL-------GVDKSRLKQKI

KVEKPEGVIGEPIPFETVYPTIPKDIIEQVKHDPWKYLKAHPEHKAVPLNQL--------------------

-----------------------

>pCRESS7|WP_028509833.1

M--PDK--------------------------------------SWRTCEITQDCC-----------YW---

----------------TP-ENVHKFI----DG--WSSVKDYAYILHDKDKKDD------------NTT--P-

REPHIHLMLRF--SCAVHTSNIAKKVG-----IPE-NRIQ--KM---------K----S-WSAALNYLTHR-

-----------------DE--HKPWKHVYDTAEVI-SN-------FD----WQLESESAHQAKQLRADK---

-G--R----EK-EIVEAIASGEI-RLFN----L--SEHIT------SYEEN--LYSKAIKTAFNRRTRDLKL

-------KNER-NMEVIFISGESGVGKDTFAREWCKD----------KG---LS-YF-TTGN-NDKS--PFD

DY-MGQDVIIWSDARD---DV-Y----KPAQIHTMLDNHWS-ST-QKARFVDQVLNC-Q---YFIITSVKPL

NEWYK--NFY---S-----------KEG-EDIKQLYRRIKTWYDM---NDKDIS-CRI--YDQNLN------

---EYIYTDSYLNIYGHKSEYLKMLSWNGMSDL-FRR-TSNIVEELEKNNANFF--DVYE------------

---------DETSELRSCLDAIHDIQANFYEHGKEIRYDKEIDGIVVDDIYE--------------------

-----------------------

>pCRESS8|WP_033683822.1

M---------------------------------------V---KARNVMFVQQIE-----------YF---

-------------QSSNL-QDIIEYMT----N--ILKPHRFAGILHDKDIGQD-------------GN--L-

VAPHIHLVLQF--ESARSLNNLAKLTK-----QPI-QCFE--QW---------R----GSVNNAYSYLVHHT

----------------ESN--QD--KYQYSPKEVI-AN-------FD----YLLLLDTIER-NVTKR-YE--

IN--DTMI-ID-NLLDLLYTGNI-TKSE----I--EQRL----T--GSQYA--KARQKIETVYLKRLETQAE

LWRQEMIDKNE-IVTIIWLFGKAGTGKTRLARQYAEQ--Y--------D---LN-YFITGSI-RD----PFQ

QY-NLEHVIILDELRP---HQ-F----DYSDLLKMFDPYNVKAM-ASSRYFDKPLLA-N---IYIITSPYSP

YDFFL--ELTK-KRK----------SHI-DSWGQLMRRLSLVVEV---KKEHLQ-FYK--YEPMDQ--MF--

---YIDHGNKLP---------------NPFKNQ-----TDREE-------TIQDKSYQ-L------------

--------------FLEL---NK-----------K----------K--E-RN----E---------------

-----------------------

>pCRESS8|EFO53527.1

M---------------------------------------V---KARNIMFVQQIE-----------YF---

-------------QSSNL-QDIIEYMT----N--TLKPIRFAGILHDKDIGQD-------------GN--L-

VTPHIHLVLQF--ESARSLNNLAKLTK-----QPI-QCFE--QW---------R----GSVNNAYSYLVHHT

----------------ESN--QD--KYQYSPKEVI-AN-------FD----YLLLLDTIEK-NVTKR-YE--

IN--DTMI-ID-NLLDLLYIGDI-TKSE----I--EQRL----T--GSQYA--KARQKIETVYLKRLETQAE

LWRQEMIDKNE-IVTIIWLFGKAGTGKTRLARQYAEQ--Y--------D---LN-YFITGSI-RD----PFQ

QY-NLEHVIILDELRP---HQ-F----DYSDLLKMFDPYNVKAM-ASSRYFDKPLLA-N---IYIITSPYSP

YDFFL--ELTK-KRK----------SHI-DSWGQLMRRLSLVVEV---KKEYLQ-FYK--YEPMDQ--MF--

---YIDHGNKLP---------------NPFKNQ-----TDREE-------TIQDKSYQ-L------------

--------------FLEL---NK-----------KRKGMNKCKKFK--Q-LP----T---------------

-----------------------

>pCRESS8|WP_049523992.1

M---------------------------------------V---KARNVMFVQQID-----------YF---

-------------KTPNI-QAIIKELT----D--VLKPIRFAGILHDKDIGSD-------------GT--A-

VAPHIHLILQF--ESARSLNNLAKLTS-----QPI-QCFE--QW---------R----GSINNAYSYLVHHT

----------------SSD--QD--KYQYSPKEVI-AN-------FD----YLFLLETIEK-NVRKR-NE--

VN--DSMI-ID-NLLDLLYAGEI-TKSE----I--EERL----T--GSQYA--KAKHKIDTVHLKRLEIQAE

SWRHEMKNKNA-IVTIIWLFGKAGTGKTRLARQYAEQ--F--------D---PN-YFITGSI-RD----PFQ

QY-NLEHVVILDELRP---HQ-F----DYSDLLKMFDPYNDRAM-ASSRYFDKPLLA-N---VYIITSPYSP

YNFFL--ELTK-KKQ----------SHI-DSWGQLMRRLTLVIEI---SKEYLQ-FYK--YSPLDQ--MF--

---YVDHMKKLS---------------NPFKDE-----NEKTN-------LIHNNSYE-L------------

--------------FLEL---NQ-----------E----------ENNE-KH----I---------------

-----------------------

>pCRESS8|WP_024410839.1

M---------------------------------------V---KARNVMFVQQIE-----------YL---

-------------NSSNL-QGIIHDIT----E--VLKPIRFAGILHDKDKGQN-------------GD--T-

VEPHIHLVLQF--ESARSLNNLAKLTE-----QPI-QCFE--QW---------R----GSVNNAYSYLVHHT

----------------ESN--QD--KYQYSPKEVI-AN-------FD----YLLLLDTIEK-NVTKR-HE--

VN--DTMI-ID-NLLDLLYTGEI-TKSE----I--EQRL----T--GSQYA--KAKAKIEAVHLKQLENKSC

EWQKEMREKNE-KSIVIWLFGKAGTGKTRLARRYAKQ--F--------S---ET-YFITGSI-RD----PFQ

HY-QMEPVIILDELRP---HQ-L----DYSDLLKMFDPYNVKAI-ASSRYFDKPLLA-N---VFIATSPYSP

YDFFL--ELRK-GRH---I------ADV-DNYQQLMRRLTLVLEV---SKQFIE-MYS--YDKHLG--LF--

---LKDGKQKLP---------------NPYYQE-----EVADYN--------ETERLE-L------------

--------------FKNL---TE-----------K----------GMQD-EK----I---------------

-----------------------

>pCRESS8|CYX46115.1

M-------------------------------------------KARNVMFTQQVS-----------YL---

-------------TSGNL-TDIIEEIR----E--KMKPKRIAGIIHDKDLDEN-------------SQ--L-

VKPHVHIVLQF--DSARSLNNIAKLFN-----QPV-QCLE--AW---------R----GSVNNAYSYLVHHT

----------------KSA--SN--KHIYDPKEVV-AD-------FD----YIELLEKIRQ-NVTKQ-SK--

IN--DSVI-IN-NLLDLLYEGAI-SKQE----I--ESRL----S--GSQLA--KAQSKIETIHLKRLEQNAR

EWQKEMRERNE-KSTVIWLFGKAGTGKTRLAKHYARQ--Y--------S---DT-YFITGST-RD----PFQ

HY-QMEPVVILDELRP---HQ-F----DYSDFLKLFDPYNEQVM-ASSRYFDKPLMA-N---TYIVTTPYSP

YDFFL--ELRK-SRH---I------AQV-DSYQQLMRRLTLVLEV---SKQFIE-MHT--YDTYLQ--LF--

---LKDGKQKLP---------------NPFYRE-----EKADYG--------EQERFN-L------------

--------------FKEL---TE-----------E----------GKQD-ET----S---------------

-----------------------

>pCRESS8|CYW87437.1

M-------------------------------------------KARNVMFTQQVS-----------YL---

-------------TSGNL-TDIIKEIR----E--KMKPKKIAGIIHDKDLDEN-------------GQ--L-

VKPHIHIVLQF--DSARSLNNIAKLLN-----QPV-QCLE--AW---------R----GSVNNAYSYLVHHT

----------------QSA--SN--KHIYDPKEVV-AD-------FD----YIELLEKIRQ-NVTKQ-SK--

IN--DSVI-IN-NLLDLLYEGAI-SKKE----I--EQKL----S--GSQYA--KAKAKIEAVYLKQLENRAS

EWQKEMREKNE-KSIVIWLFGKAGTGKTRLARHYAKQ--F--------N---EI-YFITGSI-RD----PFQ

NY-QLEPVIILDELRP---HQ-F----DYTDLLKLFDPYNEQVM-ASSRYFDKPIMA-N---IFVITSPYSP

YDFFL--ELRK-SRH---I------AQV-DSYQQLMRRLTLVLEV---SKQFIE-MYN--YDKHLG--LF--

---LKNGKQKLP---------------NPYYQE-----EAQNHN--------ETNQFE-L------------

--------------FKDL---TE-----------K----------GIQN-EK----I---------------

-----------------------

>pCRESS8|WP_051448806.1

-------------------------------------------------MYQQQLS-----------HL-PS

S---I--------KRKTL-NSIVKSLV----E--RLEPLKIAAIIHDKDVNEE-------------GD--S-

IEKHVHVVLQF--ENQRSLERLAKVLN-----EPI-SSFQ--QW---------R----GNVNNAYSYLVHQT

----------------KEA--KG--KYQYQLEEVK-AN-------FD----YPALMSDITK-KIEQK-IK--

MK--DSEI-IK-SLLDQLGAGEL-SKDE----V--ILNL----T--GSQFA--KAKKQINDVYQQVQEQKSK

FWLEEQKSKNE-PITVIWIYGSSGTGKTALAKKYADE--Q--------N---VK-YFITGSS-RD----SFQ

HY-DGEHLVILDELRP---TT-F----NYDDLLKMLDPFGENPK-APSRFFDKSLMI-D---IFIITSPYSP

KQFYD--EIFR-HKK-----------TV-DSFGQLQRRITFVQFI---TQDYFE-MQN--YNDLEK--KY--

---ISVEDTRQK---------------NYLLEK-----LKKKYN------TNGRQTYD-K------------

--------------FNQLLT------------------------------------K---------------

-----------------------

>pCRESS8|WP_050444210.1

M--ST-----------------------------------T---KAVNVMYEQQLR-----------YL-PP

T---I--------KRKTL-NSIIKLLI----E--CLKPKKIAGIIHDKDINDK-------------GE--V-

IEKHVHVVLQF--QHARSLKNLARLLK-----EPV-SAFQ--KW---------H----GNVNNAYSYLVHRT

----------------ADA--QE--KYPYSLEEVK-AN-------FD----YPQLMKNISK-KITGS-NR--

QR--DNEI-IK-QLLDRLGAGEL-TREE----V--ISNL----T--GSQFA--KAKKNIQDVHEQVQADKAK

IWLEKRKEKGE-STTVIWIYGQSGAGKTLLAKKYATK--K--------E---QD-YFITGSS-KD----SFQ

HY-QGEHIVILDELRP---KT-F----PYDDLLKMLDPFGETPK-APSRFFDKSLMV-D---VFIITSPYSP

KQFYD--EIFR-RKK-----------TI-DSFKQLQRRINYVQFM---SSDYFE-MQE--YDVYSG--SY--

---ISVPHSRKK---------------NTLIDN-----RNSPAP------LDNKKIHQ-E------------

--------------FSAFFPDDK-----------K----------GIEI-KH----E---------------

-----------------------

>pCRESS8|WP_010817837.1

M--ST-----------------------------------T---KAVNVMYEQQLH-----------HL-PP

T---I--------KRRTL-NSIIKQLT----E--CLKPEKIAGIIHDKDVNDE-------------GV--P-

VEKHVHVVLQF--QHARSLENLARLIK-----EPV-SAFQ--KW---------H----GNINNAYSYLVHRT

----------------TDA--QE--KYLYSLEEVK-AN-------FD----YPELMKSISK-KINKS-NK--

QR--DNEI-IK-QLLDRLGAGEL-TREE----V--ISNL----T--GSQFA--KAKKHIQDVHEQVQADKAK

IWLEKRKEKGE-PITVIWIYGQSETGKSLLAKKYAAK--K--------A---KN-YFITGSS-KD----SFQ

YY-QGEHIVILDELRP---KT-F----PYDDLLKMLDPFGENPK-APSRFFDKSLMV-D---VFIITSPYSP

KQFYD--EIFK-RKK-----------TI-DSFKQLQRRISYVQFM---SSDYFE-MQE--YDIYSG--SY--

---ISVPHSRKK---------------NTLING-----TKHPAT------LDNKKIHQ-E------------

--------------FSDFFPDDK-----------E----------GSET-NH----E---------------

-----------------------

>pCRESS8|WP_016226904.1

M--SDKENC------------R------------------V---RSKNMMYTQQIR-----------HL-P-

--------------VGST-DQLVSRVK----K---MAPKRYALIVHDSDVNEQ-------------GE--P-

AEDHVHVMLSF--ENARSINSIAKELG-----DEP-QSIE--MW---------K----GKAENGYSYLIHAT

----------------KDS----GNKYQYPPSRVI-AN-------FN----YQEEIKRITE-EVERS-RQ--

-----TAN-SK-ILLDSLYKGEI-SKEE----L--EKRL----S--GSQYG--RMRRQIEDVWCKHLQFQAA

KWREEMKKSGK-RIKVIWISGEAGTGKTSLAKEYAEK--S--------G---RP-YFITGSS-RD----IFQ

NY-SGEHTIILDEFRA---DM-M----KYPDLLRILDPFGSQVM-APSRYNDKPLAC-D---LILITSPYNP

VEFYR--QLFS-RAP---A------NFV-DSLEQLLRRISLTIEM---DASHIR-AVE--YDKEAE--AY--

---RAVDNAEKE---------------NRYSRH-----YREQD-------STASNPKE-L------------

--------------FESMFREAPCVEPGSLASVGK----------EGDN-EQ----D---------------

-----------------------

>pCRESS8|SCH55298.1

M--TGSAP--------------------------------R---KTRAMMYEQQLC-----------HL-PA

-------------SV----DEMYRRIE----A---LAPKRYAGIVHDHDITDA-------------GR--P-

AADHLHVMMEF--ANPRSVRSVAKSLG-----DKA-ERLE--AW---------K----AGTENGFSYLCHRT

----------------DGA--RS--KYQYDPSLVR-AN-------FD----YPAALASIES-RVSKA-RS--

-----HSN-IK-ILLDDLLEGRI-DREG----L--ISQL----S--GSEYA--RAKRQIEDVYARRLQVSAA

EWRAKMRDEGR-RVQTIWIFGPAGTGKSSLAKQYAQS--K--------G---EP-FFVSGST-RD----VFQ

GY-AGQHTIILDELRP---SS-I----PYADLLRVTDPYAIEVM-APARYADKAIAA-D---LIIVTTPYNP

MEFYC--EQVR-GAT---VGR---PDDI-DGFGQLERRLSLVVEM---QQKEIC-LSE--FRVELG--TY--

---WPADGSSRP---------------NPYSSF-----AR-GS-------SGSGDSAH-L------------

--------------YEALL---------------D----------SGTV-TA----D---------------

-----------------------

>pCRESS8|WP_042900192.1

M---------------------------------------P---KQRAIMYTQQMR-----------LA---

-------------ILSDW-KKEIDRIV----K--LLEPLLWAGILHDKDVNED-------------GE--T-

VEPHIHLMMYF--KHARSPHSIAWEIN-----ERI-ERLE--FF---------K-----HPNNGFSYLVHQT

----------------KDA--QN--KYQYPISEVI-SN-------FD----FAKKLENIRK-QVERN-QS--

KK--EGEL-IR-EYLDMLYDGLL-TLEE----I--ESEL----T--GSQYA--KASTRLKAVAEKRQERLGR

EFLNRMKYEQK-TKQVVYIYGESGLGKTRLAKTYAEN--K--------N---TS-YFVTGSS-RD----PFQ

SY-QNQETIIIDELRP---DS-F----RYDDLLKILDPYNFDVF-LPSRYIDKALTA-E---LIFITSPYSP

KELYD--NFQT-S------------KRI-DRFDQLERRIQTAILV---EKDNIF-YTH--YNYESR--EY--

---EKDDSNFFT---------------NPFES----------------------------------------

--------------------------------------------------------Q---------------

-----------------------

>pCRESS8|WP_050492321.1

M-------------SSEKKTNK----------------KIP---KQRAIMYTQQMR-----------LA---

-------------ILSDW-KKEIDRIV----K--LLEPLLWAGILHDKDVNED-------------GE--T-

VEPHIHLMMYF--KHARSPHSIAWEIN-----ERI-ERLE--FF---------K-----HPNNGFSYLVHQT

----------------KDA--QN--KYQYPISEVI-SN-------FD----FAKKLENIRK-QVERN-QS--

KK--EGEL-IR-EYLDMLYDGLL-TLEE----I--ESEL----T--GSQYA--KASTRLKAVAEKRQERLGR

EFLNRMKYEQK-TKQVVYIYGESGLGKTRLAKTYAEN--K--------N---TS-YFVTGSS-RD----PFQ

SY-QNQETIIIDELRP---DS-F----RYDDLLKILDPYNFDVF-LPSRYIDKALTA-E---LIFITSPYSP

KELYD--NFQT-S------------KRI-DRFDQLERRIQTAILV---EKDNIF-YTH--YNYESR--EY--

---EKDDSNFFT---------------NPFES------QPIHR---------ANSFFT-Q------------

--------------FETTIQRK----------------------------------E---------------

-----------------------

>pCRESS8|WP_000093566.1

M-------------SSEKKTNK----------------KIP---KQRAIMYTQQMR-----------LA---

-------------ILSDW-KEEIDRIV----K--LLEPLLWAGILHDKDVNED-------------GE--T-

VEPHIHLMMYF--KHARSPHSIAWEIN-----ERI-ERLE--FF---------K-----HPNNGFSYLVHQT

----------------KDA--QN--KYQYPISEVI-SN-------FD----FAKKLENIRK-QVERN-QS--

KK--EGEL-IR-EYLDMLYDGLL-TLEE----I--ESEL----T--GSQYA--KASTRLKAVAEKRQERLGR

EFLNRMKYEQK-TKQVVYIYGESGLGKTRLAKTYAEN--K--------N---TS-YFVTGSS-RD----PFQ

SY-QNQETIIIDELRP---DS-F----RYDDLLKILDPYNFDVF-LPSRYIDKALTA-E---LIFITSPYSP

KELYD--NFQT-S------------KRI-DRFDQLERRIQTAILV---EKDNIF-YTH--YNNESR--EY--

---EKDDSNFFT---------------NPFES------QSIHR---------ANSFFT-Q------------

--------------FETTIQRK----------------------------EQ----Q---------------

-----------------------

>pCRESS8|KXA58447.1

M--PKTKRKEILEMSSEKKTNK----------------KIP---KQRAIMYTQQMR-----------LA---

-------------ILSDW-KKEIDRIV----K--LLEPLLWAGILHDKDVNED-------------GE--T-

VEPHIHLMMYF--KHARSPHSIAWEIN-----ERI-ERLE--FF---------K-----HLNNGFSYLVHQT

----------------KDA--QN--KYQYPISEVI-SN-------FD----FAKKLENIRK-QVERN-QS--

KK--EGEL-IR-EYLDMLYDGLL-TLEE----I--ESEL----T--GSQYA--KASTRLKAVAEKRQERLGR

EFLNRMKYEQK-TKQVVYIYGESGLGKTRLAKTYAEN--K--------N---TS-YFVTGSS-RD----PFQ

SY-QNQETIIIDELRP---DS-F----RYDDLLKILDPYNFDVF-LPSRYIDKALTA-E---LIFITSPYSP

KELYD--NFQT-S------------KRI-DRFDQLERRIQTAILV---EKDNIF-YTH--YNYESR--EY--

---EKDDSNFFT---------------NPFES------QSIHR---------ANSFFT-Q------------

--------------FETTIQRK----------------------------EQ----Q---------------

-----------------------

>pCRESS8|WP_036093565.1

-------------------------------------------------MYTQQLQ-----------HL---

-------------PFQDV-AAFQSRL-----E--NINVAEYAFIIHDQDT-VD-------------GH--P-

VTSHIHAVLRY--QNARSVDSVAKQVS-----DKA-QYIE--IW---------N----GNYANAYAYLVHKT

----------------DGA--SR--KYQYPLDSVT-SN-------FD----FKERIESFSS-TGNRT-----

----NQLA-IK-YILDEIVSGKILTKEE----A--YQLLP------GSLLS--KSVNNINSAFQIRQVLEAE

RWRKDKKASGE-RIHVIWIFGVAGTGKTRFAIDFFKK----------LN---MK-YFKSGSS-KD----PFQ

GY-SGQHGIILDDLRP---NEGL----SYADLLRFLDPWNLEAM-AASRYFDKGLQA-D---YVIITSPLGP

VEFYD--SLFT--------------DEM-DAFDQLTRRLETVLYF---DRQSIM-ECE--LMSLNYKLQY--

---RASTSLKVE---------------NRWYVP-----EEEQSEGHFNLSEIQN------------------

----------------------------------------LFKDEEETNTDE--------------------

-----------------------

>pCRESS8|EEJ43069.1

M--PK---------------------------------------RSRTFMYTQQLQ-----------HL---

-------------PFQDV-AAFQSRL-----E--NINVAEYAFIIHDQDT-VD-------------GH--P-

VTSHIHAVLRY--QNARSVDSVAKQVS-----DKA-QYIE--IW---------N----GNYANAYAYLVHKT

----------------DGA--SR--KYQYPLDSVT-SN-------FD----FKERIESFSS-TGNRT-----

----NQLA-IK-YILDEIVSGKILTKEE----A--YQLLP------GSLLS--KSVNNINSAFQIRQVLEAE

RWRKDKKASGE-RIHVIWIFGVAGTGKTRFAIDFFKK----------LN---MK-YFKSGSS-KD----PFQ

GY-SGQHGIILDDLRP---NEGL----SYADLLRFLDPWNLEAM-AASRYFDKGLQA-D---YVIITSPLGP

VEFYD--SLFT--------------DEM-DAFDQLTRRLETVLYF---DRQSIM-ECE--LMSLNYKLQY--

---RASTSLKVE---------------NRWYVP-----EEEQSEGHFNLSEIQN------------------

----------------------------------------LFKDEEETNTDE--------------------

-----------------------

>pCRESS8|WP_004900270.1

M--KEKSPETNHSADSQLMTAE----------------VGK---KYRQFMFVQQLR-----------YL-G-

--------------Y-DL-TALNERI-----D--IIKPLEFAYVVHDKDLTED-------------GE--L-

IEPHLHLALRF--ENPVSLKRLAESLE-----TEP-QYIA--QW---------K----GAANNLYSYLIHRT

----------------ETA--SD--RYQYEVNEVV-AN-------FD----FPGKIERIEQ-AIQGR-KG--

KR--DGTL-LN-ETLDALLEGNI-TLFD----A--FEIL----P--GRIAG--KHRKKLESAYQTRMELNAK

VWLENKKALGK-PIQVFWFYGPAGVGKTRYAKQYLSR--I-------DD---GE-IFISGSN-RD----PFQ

NY-SKSHKVILDDIRP---NT-F----SYEELLRIFDPWNMEVS-VGSRYSDKNLQV-D---IFIITSPLAP

DVFVD--TLQV--------------FELDDNFDQLLRRLTTVMYF---DDQYIYPAVP--YLGMGKEIHY--

---KLDEDNKVI---------------NQWSGH-----QGEQEHGLQLVREAGRKTIE-K------------

--------------FLEMMSQEQDKLTDGKGIRKE----------RTDE-RD----D---------------

-----------------------

>pCRESS8|WP_062359070.1

M--NK---------------------------------------KYRTFGYTQQLS-----------HL-N-

---------------QRL-EDFPRLM-----E--EAGVEQYAYIVHDKDRTAD-------------GV--L-

KASHVHVVMKF--ENPRSLQAVSKIFH-----DSP-QYVEP-KT---------R----NGYNNMLAYLTHRT

----------------KGA--TM--KHQYDPDAVA-AN-------FD----YSDALEKITA-KVEQR-DS--

-----KTR-LD-ELLERLMDYSI-TYEE----A--VGEL----T--ASEFS--RYHQRLKVTAEYGRQQYAK

RWIKEHEDNHK-PIEVIWITGPAGSGKTVEAKKIARE--K-------SD---KD-FYVAVGV-RD----PFQ

LY-TDENVIILDDLRA---KT-F----DYNTLLQLLDPFNS-TM-ADARYSNKTLIA-D---TIIITSVYSP

LDFYH--MLVP-EIY----------RDV-DTYKQLERRISQIIR----------------------------

------------------------------------------------------------------------

--------------------------------------------------KR----C---------------

-----------------------

>pCRESS8|WP_008469878.1

M--KK---------------------------------------RFRQFMYTQDLD-----------HL-PF

----------------NI-KSLQSLI-----K--TEPLEEWAYIIHDKDKNED-------------NT--P-

IRKHLHLVLKY--KNPQTLHHISNLLT-----DKD-QYFE--IW---------N----GRINNAYSYLIHAT

----------------NEA--RE--KYQYSPSEVT-AS-------FN----FEKRISKIKD-SISNS-----

-----NNK-VQ-TYIQRYAEEEI-SYKQ----L--IDQI----G--LINIA--KNKRLIDTIQNLLISKHHQ

EWLHNF--EGK-KMICLWLWGDAGVGKTTYAKKLLSQ---------------EN-YIVLGSS-ND----YFQ

NY-TDESFAIINDLRP---ED-W----KYADLLRLLDPYEHNKM-APSRYHDKELNL-E---MIIITTPYSP

QSFYH--NSRI-FN-----------PKI-DSFEQLKRRIF-PIHI---TPEFIRKEKSYGYFA---------

------------------------------------------------------------------------

--------------------------------------------------------N---------------

-----------------------

>pCRESS8|WP_046324376.1

M--AEI--------------------------------------RARQFMYMQDFA-----------HL-KI

----------------DF-TELTNIL-----T--KANIQEWAYIIHDQDLDQG-------------NR--L-

IRKHLHVVLKY--ANPQILSHVVRLFN-----DKP-QYLE--VW---------Q----GRISNAYSYLIHAT

----------------LEA--KD--KYQYDPNSVV-AS-------FD----FPARITEIQS-SVNQSRL---

----NSKV-VA-NFLTQYANEEI-DYQE----L--ADII----G--LAEVA--KRKSVIDNITKLIADKKHE

EWLHEY--HDR-KAETIWLWGEAGVGKTRYANKLVRG---------------EK-VAILGSS-RD----YFQ

DY-HGEHYVILNDLRP---ND-F----RYADLLRLLDPYEHDKV-APRRYHDVKLNV-E---MLIITTPYSP

FEFYQ--HVKI-AD-----------EKT-DTFEQLKRRVH-AIHI---TPKFIAEVFQ--------------

------------------------------DNE-----SEF-------------------------------

----------------------------------------------EELFGG----R---------------

-----------------------

>pCRESS8|WP_049150683.1

M--QEL--------------------------------------RARQWMYVQYFK-----------YL-PF

----------------KI-NNLTEIL-----T--KDNCQEWAYIVHDKDTKED-------------GS--L-

ISPHVHVLIKY--SNPQTLKHVANLFK-----DKP-QYFD--IW---------K----GRINNGYSYLIHST

----------------SEAINKG--KYKYSPNEVV-AS-------FD----FSKRIEKIQR-QVLNKKI---

----KSNN-SN-LFIEKYAEGEI-SYND----L--ENII----G--VVQVA--RHKTIIDHINQINASNKHK

EWLKSF--KGK-PMETHWLWGAAGVGKTRYAKWLAKN---------------DK-VAILGSS-RD----YFQ

EY-HGEHIVILNDLRP---ND-F----NYGDLLRLLDPYEHNKM-APRRYHDVYLNL-E---MLIITTPYSP

WSFYK--QCKI-DN-----------PEV-DTYKQLNRRVH-AVHV---TKDFISDIVP--------------

------------------------------YEF-----NMN-------------------------------

----------------------------------------------FENLGF----E---------------

-----------------------

>pCRESS8|WP_014567781.1

M--G----------------------------------VPR---RARQFMYMQDVD-----------HL-KI

----------------KE-KSLKSIL-----N--KSGALEWSFIKHDQDIDEN-------------GK--L-

IRPHYHVILKY--EYPRSILSVAQIFK-----DQT-QYVE--IW---------S----GRIANAYSYLIHET

----------------EEA--AT--KHHYQDNEVV-AS-------FD----FHKRMEEIRK-KIKKS-----

-----SKY-VM-EMIERYANNKL-TYEE----L--AKEL----G--VMPMA--KHQQLIDRISQVQEEEAHK

RWLTKM--KGK-SMKVIWLYGAAGVGKTRMAEIMLSK---------------HK-YVILGSS-RD----YFQ

DY-HGEHYIVLNDLRP---ND-F----PYSDLLRLLDPYQHDKA-APSRYHDKKLSA-E---EIIITTPYSP

YDFYR--NISI-HD-----------KSV-DTIDQLLRRVI-PIKI---TPSFFKSVLE--------------

------------------------------KRA-----KRRA------------------------------

--------------------------------------------------KS----N---------------

-----------------------

>pCRESS8|WP_007125042.1

M--K----------------------------------KEI---RARQFMYVQDLN-----------HL-SV

----------------KE-SDFKQIL-----N--QSGAMEWAYIKHDKDKDQE-------------GK--I-

IRPHIHAVLKY--ENPQKLSTIANLFN-----DQA-QYVD--VW---------K----GRIANAYSYLLHET

----------------EEAREQG--KHVYKDTEVV-AS-------FD----FPARMKNIRT-KISKS-----

-----PKY-IS-SLINQYAEGKI-QYQE----L--EDQI----G--ISQLA--RRKKLIDQIDELKADKEHK

EWLKKF--EGK-PMHTLWLYGEAGVGKTRYAEYLLRK---------------KK-YVILGSS-RD----YFQ

TY-NGEHFIILNDLRP---ND-F----NYSDLLRILDPYQHDKA-APSRYRDKKLSV-E---EIIITTPYSP

KDFYR--TTKI-DD-----------RQV-DTVDQLLRRIQ-PIHV---TPKFIKRRLK--I-----------

------------------------------KRP-----KHTD------------------------------

----------------------------------------------QD---N----A---------------

-----------------------

>pCRESS8|WP_060461663.1

M--K----------------------------------KKVKEPRARQFMYVQDLD-----------HL-KV

----------------DE-KDLSTIL-----K--QSGALEWAYIKHDKDKDQD-------------GK--T-

IRPHIHVVLKY--ENPQKISSVASMFK-----DQP-QYVG--VW---------K----GRIANAYSYLLHET

----------------EEAQAQG--KHVYKASDVV-AS-------FD----FEARMKSIRA-KVTKS-----

-----PKY-VS-SLIDQYAESKI-TYDE----L--ESQI----G--ISQLA--RRKKLVDQITELRAEKEHE

KWLKDF--KGK-KMKVLWLYGAAGVGKTRFAEYLFRN---------------KK-YSILGSR-RD----YFQ

DY-CGEHFVILNDLRP---RD-F----SYSDLLRILDPYQHDKS-APSRYHDKKLNL-E---EIIITTPYAP

TDFYK--YVFI-DD-----------RRV-DTDEQLLRRIQ-PIHI---TKSFIKKRLK--T-----------

------------------------------KKS-----KQNG------------------------------

----------------------------------------------QN---N----A---------------

-----------------------

>pCRESS8|WP_013641481.1

M--K----------------------------------------RFRQFMFVQDVE-----------HM-NF

----------------KF-EELPNIL-----K--SESSSEWAYIKHNHDNDKE-------------DT--K-

VRDHIHVVLKY--RNPQTVAHVAKLFK-----DKS-NNVQ--IW---------I----GRINNAYSYLVHNT

----------------DNA--TS--KHQYSIEDVK-AS-------FD----FKKRIKDIEE-NVSLA-----

----KQRN-IK-EAINGFAEGDI-DYKE----L--IETL----G--IVNVA--KNRNLIDSIQKIREQVIQH

EWWNQF--NGK-QMASLWLWGEAGVGKTTYAERILSN---------------EK-YIVLGNS-ND----YFQ

YY-NGEHYIILNDLRP---GD-L----KYADLLRILDPYA-IKY-TFGRYHNHPLLA-E---MIIITTPYSP

REFYK--NTRI-AN-----------RKI-DSLTQLQRRIF-EIHI---TKDFILKENK--Y-----------

------------------------------QRN-----SKEG------------------------------

----------------------------------------------S----S----D---------------

-----------------------

>pCRESS8|WP_003549058.1

M--K----------------------------------KAI---RARQFMYTQDLD-----------HL-PF

----------------KK-EDLKTLL-----E--KSSAEEWAYILHDKDIGKN-------------GK--T-

IRPHFHVVMKF--KDAKTISRVAKLFN-----DKQ-EYIE--VW---------R----NTIGNAYSYLIHET

----------------SNA--KD--KHHYDPIEVV-SS-------FD----FETKIKQIRK-KVAKL-----

----SKKD-ID-DLIDDYSNEIL-TKTD----L--QEKI----G--VLEMA--KHKTLLDHIDDILAYKKHQ

KFLKDF--QGQ-KCTTYWIYGESGIGKTKMVREILEQ--L-------HP---NN-FVVLGSQ-RD----HFQ

EY-RGEEFIVINDLRP---RD-Y----EYGQLLTLLDPWEIDKM-APARYHDKYLNA-R---AIYITTPYSP

MAFYN--ESGI-VN-----------SLI-DSFEQLNRRIL-SLHL---TSDTYNQMKA--DLIRDD------

---QIAEAIWKI---------------KKTKST-----SQTD------------------------------

----------------------------------------------NDK-SN----D---------------

-----------------------

>pCRESS8|KRN00682.1

M--K----------------------------------KAI---RARQFMYTQDLN-----------HL-PF

----------------KK-EDLKTIL-----E--KSSAEEWAYILHNKDIDKD-------------GK--T-

IRPHFHVVLKF--KDAKTISRIAKLFN-----DKQ-EYIE--VW---------R----NTIGNAYSYLIHET

----------------RNA--KD--KHHYSPTEVI-SS-------FD----FETRIKQIRK-KVAKP-----

----SKKD-ID-DLIDDYSNEIL-TKTE----L--QEKI----G--VLEMA--KHKTLLDHIEDILAYKKHQ

QFLQDF--KGQ-KCTTYWIYGESGIGKTKMVREILEQ--L-------HP---NN-FVILGSQ-RD----HFQ

EY-RGEEFIVINDLRP---RD-Y----EYGQLLTLLDPWEIDKM-APARYHDKYLNA-R---AVYITTPYSP

IAFYQ--GANI-VN-----------GLI-DSFEQLNRRII-TLHL---TEDNYDEMKA--DLIKED------

---QIAEAIWKI---------------KKQKNT-----SHTD------------------------------

----------------------------------------------SEK-NN----D---------------

-----------------------

>pCRESS8|CDA26462.1

M--K----------------------------------KAI---RARQFMYTQDLE-----------HL-PF

----------------KQ-EQLKEIL-----E--KSDAEQWAYILHDKDVNEK-------------GE--P-

IRPHFHVILKF--KDAKTISRIAKLFN-----DQQ-QYIE--VW---------H----NTINNGYSYLIHET

----------------TNA--QN--KHHYDPSEVV-AS-------FD----FVTRIKQIRE-KVNKP-----

----SKHD-IE-NFIDDYSNEQL-TKEG----L--QEKI----G--VLEMA--KHKTLLDHIEDILAYKKHQ

QFLKDF--KGQ-KCTTYWIWGSSGIGKTKLVREVLEE--L-------HP---NN-FIILGSQ-RD----HFQ

EY-AGQEFIVINDLRP---ND-Y----DYGQLLTLLDPWEIDKM-APARYHDRYLNA-R---SIYITTPYDP

LSFYF--ECNI-SN-----------QVV-DSFEQLKRRIV-SLKL---TEDTYSDLKK--ELIEDE------

---EIAEAIWKI---------------KSQKNT-----SHAD------------------------------

----------------------------------------------SDK-SN----D---------------

-----------------------

>pCRESS8|CDI43023.1

M--K----------------------------------KKI---RSHNFMYTQDLD-----------HL-PV

----------------SK-DELKDRL-----E--KSGAEEWAYILHDKDIDEN-------------GK--K-

VRPHFHVMIHF--RDAKTISRVSKIFN-----DHQ-QYIE--AW---------H----SIINNGFSYLIHET

----------------TNA--KS--KYHYDPQEVV-AS-------FN----FEDKINEIRQ-KVKKP-----

----SRQA-ID-NFIDDYSNEEI-TKEE----L--QDKI----G--VLEMA--KHKTLLDHIEDILAYKNHQ

QFLKDF--KGQ-KCKVYWIYGVSGIGKTKLVREILEK--R-------HP---ED-FCILGSQ-RD----HFQ

EY-KGQGFVVINDLRP---ND-Y----DYGQLLTLLDPWEIDKM-APARYHDRYLNA-R---AIYITTPYDP

LSFYF--ECNI-AN-----------QLV-DSFEQLKRRII-PLQL---TKNNINKVKH--DLITDD------

---KLSEATWKI---------------KSQKNT-----SHAD------------------------------

----------------------------------------------SDK-SN----D---------------

-----------------------

>pCRESS8|KRK41125.1

M--K----------------------------------KII---RVRSFMYTQDLD-----------HL-PF

----------------KP-EELKDRL-----E--KSGAEEWAYILHDKDTDKD-------------DK--E-

VRPHFHVMLHF--KDAKTISRVAKVFA-----DQE-QYIE--AW---------H----STINNGYSYLIHET

----------------NNA--KK--KHHYSPSEVI-AS-------FD----FEEKIKEIRK-NVKKP-----

----SRKE-IE-NFIDNYSNEKL-TKEE----L--QEKI----G--VLEMA--KHKILLDHIDDILAYKKHQ

QFLKDF--KGQ-RCRTYWLYGVSGIGKTKLIREILEE--R-------HP---KD-FFISGSS-KD----HFQ

EY-KGQHFIVINDLRP---RD-Y----EYGQLLTLLDPWEIDKT-AKRRYKDVFINV-C---AYFISTPYSP

LNFYN--ECRI-DN-----------RLV-DTFSQLERRVI-ALHL---TKDNREKIKQ--DLITDD------

---KLFEAIWKI---------------KSQKNT-----SHAN------------------------------

----------------------------------------------SNK-SN----D---------------

-----------------------

>pCRESS8|WP_012845653.1

M--SKTFALKRKTARTELIYYKYITIHCVICVGYKNEMKVI---RSRNFMYTQDLD-----------HL-PF

----------------NK-DTLKTRL-----E--KSGAQEWAFILHDKDVDEN-------------NK--K-

IRPHFHVMLRF--KDAKTISRISKIFN-----DKQ-QYIE--VW---------K----NSINNGYSYLIHET

----------------SKA--KN--KYHYKDSEVV-AS-------FD----FKSKINSIRR-KINKP-----

----SKQA-VD-NYIEDYANEII-SKED----L--QNNI----G--VLEMA--KHKNLLDHIEDILAFKKHQ

KFLKEF--KGK-QCKVIWLYGKAGVGKTRLIRNFLEH--Y-------YP---NN-FIILGSQ-RD----HFQ

EY-KGQNYIVINDLRP---ND-Y----EYGQLLTLLDPWENDKM-APARYHDKYLNA-K---AIFITTPYSP

KDFYN--TCNI-EN-----------IFI-DSFDQLKRRII-SFHI---TENNLSQLSQ--DITTEL------

---NL-------------------------KNK-----SKRT------------------------------

----------------------------------------------KEK-NNGSTIN---------------

-----------------------

>pCRESS8|CDI42894.1

M--K----------------------------------KTV---RSRNFMYTQDVD-----------HL-PF

----------------KK-DNLASIL-----E--KSGAQEWAFILHDKDVDES-------------KK--K-

IRPHYHVMIRF--KDAKTITKISKIFG-----DKA-QYIE--AW---------H----NTINNGYSYLLHET

----------------NGS--RN--KYHYDVSEVT-AS-------FD----FKSKIESIRR-KVKKP-----

----SRQA-IE-DYIEDYSNGIL-TREA----L--QAQI----G--VLEMA--KHKTLLDHIDQILDQKKHN

EFLEEF--KGQ-KCVTYWLWGESGVGKTRLVREALEKC-I-------EP---TN-FCILGSQ-RD----HFQ

VY-EGQNHIVINDLRP---ND-Y----SYGQLLMLLDPWENDKM-APARYRDKYLNA-K---SIFITTPYDP

FSFYN--GCYI-EN-----------MVV-DSFEQLKRRIL-PLHV---TTKNADQIKE--ALIKDL------

---EIRKAIFQI---------------NAKQKA-----SH--------------------------------

----------------------------------------------TDQ-SN----D---------------

-----------------------

>pCRESS8|WP_011254167.1

M--C-----------------------VINFRMKEKGKKIV---RARQFTYVQDTD-----------HL-KV

----------------EP-KQFQDFL-----S--KSGAVEWAYILHDKDADQN-------------NK----

IRDHYHVVLKY--ANPQTISKIANIFK-----DKE-QYVQ--IW---------N----GRIDNAYSYLIHET

----------------SDA--QN--KYRYSPKEVV-AS-------FN----FEKRIEKIRT-SIKRK-----

SS--FKND-IE-EKLKDYAENII-SLYE----L--RKSI----GDYAFTTP--SIQRHIKEIKKLHDEDNHQ

KWLRSF--DGK-KMKVIWLYGEGGTGKTRCARAMTKD---------------DD-VVVLGSS-ND----YFQ

AY-DGQRVIIINDLRP---SD-F----KFGDLLKLLDPYEHSKE-APRRYRNVKLNL-E---KIIITTPYSP

ISFYN--HCYI-ED-----------KKI-DKIEQLTRRITQTIEV---TNEFTKKFLK--------------

--------------------------------------EHER------------------------------

----------------------------------------------DEK-SD----S---------------

-----------------------

>pCRESS8|WP_008472153.1

M-------------------------------------GEL---RSHNFMYVQQTK-----------YL-KV

----------------KP-EEFSEVL-----N--GIRAVKWAFIKHDKD------------------E--G-

VEEHYHVILHY--EHSSRISTVANIFD-----DDP-ERVQ--IW---------D----NRWNNACGYLIHAT

----------------KNSVKDG--KYPYDVSEVT-AN-------FD----FAKKITEIQS-RVSGA-----

------KQ-IE-NVIKEYGNGDI-DRDE----L--ELKL----G--DAELA--KNHIWISRIDDIRAQRKHE

EFLKDF--EGH-AQETIWLWGAAGVGKSRYADFLTQG---------------KE-TAKLGSS-RD----YFQ

DY-HGESFVILNDLRP---NE-F----SYADLLRITDPYQHDKS-APRRYHDLKLNL-K---MLIITSPYSP

DDFYE--YCKV-NN-----------YQI-DTFDQLKRRIH-AIHI---TSEFMKEVMP--------------

------------------------------DEF-----GQDD------------------------------

----------------------------------------------EWQ-GF----M---------------

-----------------------

>pCRESS8|WP_013641468.1

M-------------------------------------GEL---KSHNFMYVQQTK-----------YL-KI

----------------KP-DQFIELL-----N--GRKSTNWAFIKHDKD------------------D--G-

VEEHYHVILHY--KYAARLSTVSHLFD-----DDP-ERIQ--IW---------D----DRWNNACGYLVHAT

----------------SNSINDG--KHSYDVSEVT-AN-------FD----FDEKMKEIQS-RVSGS-----

------KN-IE-KVITQYGNYEI-SREE----L--ELKL----G--DAELA--KNHVWISRIDDLHAEREHE

NFLKEF--KGR-AQETLWLWGEAGVGKSRYADFLTKG---------------KK-TAKLGSS-RD----YFQ

DY-KGENYVILNDLRP---NE-F----SYADLLRLTDPYQHDKA-APRRYHDLKLNL-K---TLIITSPYSP

EDFYE--YCKV-DN-----------YQI-DTFEQLKRRLH-VIHV---TDELMKQVMP--------------

------------------------------DEF-----GEDDL-----------------------------

---------------------------------------------SDLI-GF--------------------

-----------------------

>pCRESS8|WP_056985318.1

M--I----------------------------------QKV---KSRQYMMVQDLD-----------KL-PY

----------------DL-DKLKEIL-----S--GLKAKEWAFIEHDKDKSEN-------------GG--L-

VTPHVHAVIKF--ENERMLDTLADTLK-----VKP-QYLQ--VW---------K----GRINNAYSYLIHLT

----------------SGA--KN--KHIYSPKEVV-AS-------FD----FPKRISEITN-RVS-------

-----KQE-IK-DALNMYANGGL-SQTE----L--KTKI----G--NLAYA--QNLETIKKLNTVLDNQAHQ

EWLKSF--QGQ-KMTVDWYYGKAGVGKTRLALKEAKE----------SG---EQ-YCVLGSS-ND----YFQ

DY-DSQHVVILDELRP---ND-L----KYGDLLKIMDPYQHDKH-APRRYRNVALNI-E---KLIITTPYKP

ETFYK--MTKI-AD-----------RRV-DTVEQLKRRISKVINV---TPELAKKEF---------------

------------------------------------------------------------------------

----------------------------------------------GDN-HE----K---------------

-----------------------

>pCRESS8|WP_057906729.1

M-----KNKMIKKNN-----------------------KAI---RSNAYMYVQQLD-----------KL-PL

AKDQS--------IDKKI-SVLIKRIK----N--VPEIKRYALIIHDKDSDKD-------------GN--A-

IKPHVHVMLEL--DKQRSVNKIAKALD-----DSS-ERLE--SMIKKYK----R----HGIENGYAYLIHQT

----------------QGA--EK--KYQYSPEKVK-AN-------FD----YPKYIKKLQQ-RVRVT-NK--

KS--DKEF-IK-EVLNNYLAGKI-SEIE----A--KRKVL-EAN--PLMLP--RFLRQLDAVKSTKFEIESD

EWFKNRSENNK-SKSVVWISGHGGTGKTVLAKMIAEN--V-------MK---SA-YYMSGSD-KD----YFQ

DY-NGEHCVILDEFRP---DK-I----SYSDLLKMLDNNRFDVN-APSRYHDKKILA-D---LIIITSPYNP

ARYYQ--NDES-IR-----------PTV-DGFEQLDRRITMTLCV---EKDKIS-LMK--YKGYKA--EKIP

AP--GSGKFVRE---------------IPYYME-----QSNFENY----ISYI------K------------

--------------DDKNYKEKINKIIPKVLEKLHDFEKNIDKNIGLKL-GE----K---------------

-----------------------

>pCRESS8|WP_057827851.1

M-----KNQEVKKYN-----------------------NAI---RSNAYMYVQQVD-----------NL-PL

AKGQS--------LDKKV-NALIKRVK----S--VPEVKRYALIVHNKDSDEG-------------GN--T-

IKPHIHVMLEL--DKQRSVNKIAKALD-----DSS-ERLE--SMTKKYK----R----HGIENGYAYLIHRT

----------------QGA--EK--KYQYSPEEVK-AN-------FD----YPKYIKKLQQ-KVRVV-NK--

KS--DKEF-IK-EVLNNYLAGRI-SEIE----A--KRQVL-EAN--PLMLP--RFLRQLDAIKNTKLEVEAD

EWFQSRSKGDT-PKSVIWISGSGGTGKTVLAEMVARN--I-------TG---PD-YYLSGSD-RD----YFQ

DY-NGEHCVILDEFRP---DK-I----TYSDLLKMLDNNRFDVS-APARYHDKKILA-D---LIVITSPYDP

ARYYQ--NVED-IR-----------PAI-DGFDQLDRRLTMVLSV---EKDKIS-LMK--YAGYKT--EKKP

AP--DGGSFTSK---------------KPCYTE-----KSSFKNY----ISDI------K------------

--------------NQKEYKKKEAEITPKMLAGLHHFEK-IGKNIGLKMEGR----K---------------

-----------------------

>pCRESS8|WP_057827085.1

M--LQKEQQKAEKTASSP--------------------------RSRGVMYVQQLD-----------KM---

-------------KFSSL-DALKRRVQSL------SKLKRFAMIVHDKDTNGD-------------NE--L-

VKPHVHVMLEF--ESPRMLSAIAKELD-----DAP-EHFE--SMTHN------K----NGINNGFAYLVHRT

----------------KNA--SN--KYQYDPDKVI-AN-------FN----YQGFLDKLKT-EVNEGRYE--

----GKMG-IS-NLLGDFINGKL-TKIE----A---KEIAKNSK--PGQFA--HICKRIDESEVQMEELKAD

KWIQEMQGKHE-AKKVFWIYGPAGVGKTTLAKMITES----------LD---KG-YFTSGSS-RD----YFQ

NY-HGEHCILIDELRP---NV-I----DYSDLLRILDPYNYNCN-VPSRYHDHRLTA-N---AIVITSPFSP

GDFYI--HQRD-LNT-----------EV-DAFKQLHRRLNYVIQV---KPLKIR-LME--------------

-------------------------------------------------------------RSATPPQYGPR

REVMNYVMVAIESGKFQENYDQDSRCILSVLKKLISGNTNPKEKSTISKTTK--------------------

-----------------------

>pCRESS8|WP_016356676.1

M--TK---------------------------------------KYRNFMYENQLK-----------YM-KE

---RQ--------IPIDI-NMLAKYVE----E--VLNPAEYAIILHDKDKSNE-------------SE--L-

VAPHYHIALKF--ENPRNVNSVAKKFN-----DLP-QNFE--IW---------L----NRPNNLYSYLIHKT

----------------SDA--HS--KFQYDVTNVV-AN-------FD----FTERINKITK-SIHRG-----

----RNEE-IR-HLIDSFGNGEI-TLKL----L--MEEL----S--PTEYA--RNENQINIIKKLLANKRFE

AFKKRMDSEEK-RIEVFYLFGGTGTGKTRFAKTRYKE----------------N-RYITGSN-RD----LFA

NY-DGETVVILDELRP---NS-I----SYNELLKITDPFNFENV-VGSRYLDKKLVA-E---TIVITTPFSP

EEFYQ--TLKG-EQT---YNENHDSSNI-DKKEQLFRRIN-VFKF---DDVYIY-PLI--WDQTKR--VY--

---KELSEFKQE---------------NQWSES-----VTFKTE------NDIEKATKNL------------

--------------FKMS------------------------------------------------------

-----------------------

>pCRESS8|WP_016622553.1

M--TK---------------------------------------KFRNFMYENQIE-----------YM-KK

---RN--------IPIEI-DKLSKYVK----E--TLNPVEYAIILHDKDESTE-------------NK--L-

VAPHYHIALKF--ENPRKVNNVAKVFN-----DSP-QNFE--IW---------L----NRPNNMYSYLIHKT

----------------NQA--KE--KFQYDIDDVV-AN-------FD----FKERINKITK-SIKRN-----

----RKEE-IQ-HLIDSFGNGKV-TLNQ----L--MNEL----S--PTEYA--RNEHQITIVKKLLANQRFE

EFKSRMENEQK-KIEVFYLFGDTGTGKTRFAKSRYKE----------------N-RYITGSN-RD----LFA

NY-DGESILILDELRP---NS-I----SYNELLKLTDPFNFENV-AGSRYFDKKIVA-E---KIIITSPFSP

NDFYR--ALKS-DKS---HPT---INDV-DKKEQFFRRIN-VYKF---NYDYII-PML--WNDDKK--VY--

---QELLHLKIE---------------NHWSES-----IIFKTE------NDIEKATRVL------------

--------------FEIASPQLND--------------------------KG----V---------------

-----------------------

>pCRESS8|ABP89830.1

M--SLSRFERNKMKKKRKMSEKV---------------PSI---RHTAYMFCSTVE-----------SV-SI

---------------KNL-QEIIKLFQ----E--TLNPFEIAGIIHDKDID---------------------

TEPHYHIIVRF--KNAVWLNSIINKLS-----QNS-NFFE--AW---------K----GKVNNAYSYLIHRT

----------------EDA--SE--KHQYTVDEVI-AN-------FD----YAERIENIES-KIQSN-SRK-

EK--TINV-VR-NLINKIIAGDI-SFDE----A--IKEV----D--GYTLV--KYDREFSRAKKRRTEIDFE

NWKENALKNGF-KREIIWLYGPSGTGKSRLCKHFAKS--L--------G---KP-FYTTGSS-RD----PFQ

NV-ASQETIIIEEIRPGRGGN-F----NYADFLLIIDPFNADAT-ASSRFFDKPIIA-T---TIIINTPFSP

FQFYE--SISK-QVG---FD-----KKI-DTVIQLIRRITLLQEV---TDKSII-TYK--FDNEKN------

---KYIEYERID---------------NPYYDC-----DKDKIEFN----SDVYNKYKEM------------

--------------TLKISQEE-----------------------NDDR-QN----D---------------

-----------------------

>pCRESS8|CUR41281.1

M--SDEGNKIT---------------------------------RSQIVMYVQQLS-----------YL---

-------------PFNSL-DALERRLKELKKD---KGLINWAYIIHNKDR-KN-------------GK--A-

IEKHIHLDLRF--KTRMSVKSIAKMLD-----DET-ERIE--VFTKRGQSL--E----QSWINALSYLIHRT

----------------AKS--KD--KYPYDPNEVK-AN-------FD----YIKTIKNAEK-SIVGA-----

----------N-KIVDQFLKEEI-DYDT----A---EMLLSNYG--AKVLS--KNKKILDDAQNFLNRKHYK

EWVKDKKKTNQ-SIIVVWIWGEAGTGKTSFCKDFMNE----------RN---IE-YYEASGH-ND----PFQ

NY-AGEKGLILDELRP---RNYI----TYSDLLKILDPYDYDKT-AVARYHNKYLMC-D---YIFVTSPFSP

YSFYK--NAQV-KD------------TN-DSLDQLHRRISILLHF---TSKDIIEVKVEGNTYKEI------

--------SRIK---------------NVWSQEARGQQDNK-------------LSLNEL------------

--------------------------------------------------TDLKGND---------------

-----------------------

>pCRESS8|KRN07545.1

M--NGGKRLKIDKTKNEKAGRSY---------------------RSTVWMYEQQLE-----------HL-PF

---------------ANI-DALKRRVDSL-ID--NYNLDKFAMIQHAKDVNAE-------------GK--R-

VKSHIHLVMTF--KERVAANSLGKVFG-----DQP-QQFE--RMTKRGNSA--K----KGADNAFMYLIHQT

----------------DNS--RD--KYQYRVEDVV-AN-------FD----YANFVSRKRA-QID-------

---------PR-DIIELLGAGEI-QEEQ----A--RAMMM-GAS--ANTYF--KYSRRISEVALGANKLKFE

KWLKNKIETKE-SIKVIWIYGGAGTGKTRYAVEFADK----------RK---IS-FFKTVTT-ND----PFE

GY-NGQKILIIDELRP---ET-L----KYPDVLHLLDPMSYEKK-TVARYHNSNIMA-D---FIFVTTPYDP

LTFYE--KITK-LD-----------RSV-DSFEQLRRRIGLLLHF---KKKSIDAEYL--EKNSNS--RCGW

NY-CVSQSNE-----------------NPYLND-----NRSVNF-----------SLEDL-------EQYG-

------------------------------------------------K-SD----DKK-------------

-----------------------

>pCRESS8|WP_003665528.1

M--PENSKNEGSEDKKKGETPKV---------------------RSRVWMYTQQVE-----------DL-PF

---------------DSI-DALIRRVKTV------PNLDKIAWIVHDKDINKK-------------GK--K-

VTPHVHVGFTL--TKRTTISRMSKILN-----DRT-QQIT--CFTKRGQSV--A----NSTKNLMGYLIHHT

----------------REA--KQQGKHQYAPSKVH-AN-------FD----YPSYVEQTEE-ITS-------

---------TR-DILDEYANENI-SRDQ----A--ESLLK-LQG--GSDLA--HNLRNLDALDSYILEEKRR

RWVQKMKKAKK-PIYVVWLSGAAGTGKTTYAKRYAEK----------HK---LT-YFVTTSQ-ND----PFQ

GY-RGQQVLIIDEIRP---ET-L----SYADLLQICDPYLYEKN-LTARYRNPSFQS-S---IVFLTSVYTP

LEMYN--AM-R-VK-----------RKI-DTFNQLKRRIGMNLDF---SNREI-TSFV--YDFDYK--RQQW

FT-MRVESIP-----------------NPYSTS-----GLGNMF-----------TFNEL-------DQYGE

Q-------------ISSEESLKYKNHQSR-----------------PQQ-TD----D---------------

-----------------------

>pCRESS8|WP_006499656.1

M--MAKKTETISKSKP----------------------------RSAVLMLVQQVQ-----------LL-PN

---------------NPK-HFLKSKCEIL-KR--RYGLKKYAFILHDQDKSSK-------------NN--DL

VVPHYHLVMQF--DHRVDVAAVAKIFE-----QGI-EHFE--VMTKRGTST--K----TAAKNSFAYLVHAT

----------------DNS--RD--KVPYDPQKVT-AN-------FN----FRKFLADSES-ELS-------

---------TA-DILDGVAEGNI-TKDQ----A--FDMLR-AQG--ARILV--HNKKSVETMAEEYQRKHHL

EWLKKRNEEGK-GIPAVWCFGQGGTGKTSYAKHFAEE----------HG---LS-YFVTSGS-ND----PFQ

GY-QGEEVLIIDELRP---DV-L----PYSDLLQLLDPFNFEKR-LKARYFNPFFSS-N---FIFVCTVMGP

IEFYN--SMAI-AH-----------KNI-DTFEQLRRRLAMVLLF---DHHKI-AQVI--PDF--R--NGQW

YY-APTDYKA-----------------NPFANQ-----GHVNLL-----------SMGML-------DELGK

E-------------QGTLLK------DDK-----------------GSK-NT----K---------------

-----------------------

>pCRESS8|WP_046025501.1

M--AEQTDSLKGT--------A----------------------RSRVMMFEEQDK-----------FF-EA

---------------SDIGEFLKQSCQKM-AS--KYGLEEWAFIHHDKD------------------E--P-

AEPHYHLTMYF--KGRPMVSSIAEMLG-----TTP-KQIE--IMTKRGTKV--E----TARKNAFMYLIHAT

----------------LNA--RREGKFPYPPEKVV-AN-------FD----FIRFARYNLM-QVT-------

---------PE-GILEDLGVGKL-TKTQ----A--REKFM-SLG--ATVLA--KYNRKINDVAEASLAIQYT

KWRKEREAKHY-QLMTFWFCGPTGTGKTRYAKYLAEN--V-------FK---MP-YFVSGGQ-RD----AMQ

DY-EGEHLIMWDELRN---N--V----YYLELLRLLDPYNYDKA-ISSRYFNKNLMP-E---VMIITSPYRP

DELYK--IMDI-SD-----------RKQ-DKLNQLTRRVPLIYEF---QKEHI-LILK--WN-EYN--QEYW

EYDELPSVEELI---------------KADSEK-----DSSPAE-----------PFYGI-------DDYLQ

KHSDTNVPD--------KEKPPYDSPKSRKGTNDSNELSGTLSHHKGGR-DN----D---------------

-----------------------

>pCRESS8|WP_034540695.1

M--KIQSTNNKAT--------SP---------------------RSKVLMFEEQDQ-----------HFVDS

---------------SSIEEFLTDRCEVM-IK--KYGLKEYAFIHHDKDTN-N-------------GQ--P-

VKPHYHLTMYF-GDNRPMVSSIADALD-----TTE-NQIE--IMTKRGTKV--E----TARVNALMYLIHAT

----------------RNA--RRQGKYQYPATDVI-AN-------FD----YVKFVKDHML-NDD-------

---------PT-DILDDLGNGKI-VRTQ----A--RSRMM-ALG--AQVLA--KYNRKIDEIAEASLAIQNE

VWRKEHEDSHS-ELKVYWFFGQTGTGKTRFAKYLAKE--I-------FK---MP-YFVTGAR-RD----AMQ

DY-EGQHLIIWDELRD---D--V----EYSELLRLLDPYNFDKA-ISSRYYNKNLMP-D---IVIITSPYSP

DELYS--VMRI-TD-----------RKI-DKVDQLVRRVPIIHEF---CHDKI-IVRK--WN-SYE--KRYW

EKVRLPSVEEYV--------------------N-----QSLPINSLQLFQIDQFLPFYGL-------NYYKQ

H------PEIDPFTNETIEKPRYDSPKLRNGAQDSNRLSGSLSHLTGGD-DN----D---------------

-----------------------

>pCRESS8|WP_046923918.1

M--KVINSDKKTKEKKQEEKQE------------EKPKKVQ---RSSVFMFEQQVG-----------YM-PM

----------------PI-DQLYEKAK----H--AKGLVELCYILHDKDRKED-------------GT--L-

KTPHYHMSMYF--DHRKTVNSVAKMLN-----SKP-QQIE--VMTQQGQST--V----EAKNNAFAYLCHRT

----------------KNA--QEQGKYQYSPDEVT-AN-------FD----YPKWLAEQES-KVNNA-----

----------N-DILELLNDRHI-TKDQAIERI--KNEF----G--GVAYS--RNAKKIETIAYANLRADYK

DWLKRMKEKKR-DVKVLWLYGYAGTGKSHFATDLATD----------KN---LT-Y-KKLTS-KH----LFD

DI-KDQDVLIFDDFRP---DT-L----PYSSILQIFDPLNLGVS-LDARYHNAYLMS-E---YIIVTTPFSP

YEFYQ--SMYI-RN-----------RKI-DTFEQLSRRIYATMHF---TTDEIY-TVE--PKIQRYVDDFPI

YK-YFETGESIP---------------NAWSQI-----AVNGG-------KKKSHSFAD-------------

--------------FTATFENAVCIDLKDGQKKSPVKKTDENADTESDNLPF--------------------

-----------------------

>pCRESS8|AKG47101.1

M--SDI--------------------------------------RSQVIMFTQQLK-----------HL-DC

----------------SQ-DELIKKVN----K--LPYLDQYAFIIHNKDVNTD-------------NS--P-

ITSHIHLVLCF--KQRVRITSIARNLD-----QKE-QYFE--SMTKRGKDI--E----TSRNNAFAYLIHNT

----------------TQA--KKEGKYQYSPNKVT-AN-------FD----YVKLINNLKQ-IIFYS-----

---------PK-QVLADFNSGNI-NKLE----A--LKRIK-ESN--SPRIP--QYVASINKIEEINIQLKQK

NWIAEHEKSQK-PIAVVWVYGFSGTGKTEFAKHIAKKY-S-------ID---NR-YDFTGST-RD----LFQ

NI-GTASSLIIDEIRP---KD-I----KFNDLLKITDPFNYRKF-APARYKDRAIIA-D---TIIFTSPYSP

VRFFS--KYKL-DNN--------------DTFRQLQRRITLTIEI---TTKQIIQLEP--VTKPTIKLTQDE

LLNAIAINQTYS---------------TTYIQR-----AISKNTFIPHTPQQAKISLSDL------------

--------------------------------------------------------L---------------

-----------------------

>pCRESS8|WP_002821392.1

M--AGNS-------------------------------RPT---RSRGWMFVEQLK-----------SL-NF

----------------RT-SQIQNRI-----R--KAGPEKWAYIIHDKDVNAQ-------------GE--P-

IPAHIHLMMSF--KSAVTAITLAKHFS-----TTP-ERFE--NMTKGRN----K----FGIINGFNYLVHRT

----------------QNSI-DKVGKVRYDPNDVK-AN-------FN----FLDLIKQTEQ-AIKRSKKSNP

KS--ERES-VN-FILDQFGDGLI-NKAT----A--RIRLE-KLG--GHILA--QNSTKLQNLDKERAEIDYM

NWRKMMIGNHF-VKTTIFIFGETGCGKSLLAKKIASQ--S-------YP---GS-VYFSGGS-ND----PFQ

DY-EGERAVILDELRP---GI-I----EYPDLLRILDPYSWDTA-THSRYHNSKLQA-Q---LFIITTPYDP

YFFYR--FTKD-LV-----------RFM-DPFEQLNRRISMTVKV---DRNFIY-EMK--FNGVNR------

-FIPVYNSKPIK---------------NPIGKM-----IKNQSE---------NFSLSDL------------

----------------------------------LNFQIKKTDEGEKKD-DS----K---------------

-----------------------

>pCRESS8|YP_006939186.1

M--TTSKR------------------------------------RITKFMYTQQLK-----------YL-NL

----------------SI-EQLKNNLE----N--DAYIQDFAMINHNKDLDEN-------------NQ--N-

VAEHLHVFIKL--NQQKTIDYVADLVD-----DKA-QYIE--FFDKSNK----S----RNEQNGYLYLLHKT

----------------KSA--EH--KHQYSVDDLI-VKDGRWI--ED----YENNLKKYQS-----------

KR--RKTV-VQ-SILNDYADRII-DEKE----L--KDSL----T--NLELA--KNKKLINDIKQVLIEFDFQ

TYLEQE--RYK-NKQVVWIFGKSSTGKSMMSQLLAKDY-I-------SDI--ND-IYVTSSN-RD----PFE

DY-QNQKVLIIEEFRN---EN-I----GTNELLQLLDKTNGQVR-VGSRYSNKKIMA-D---LIIINTIYEP

KYFMN--F--------------------DEPIYQLLRRIDKLVKL---DNQKIE-TLE--YDSKKD------

---DFNVIKSIA---------------NNVENM-----TLKKIL----------------------------

----------------------------------------------GDD-FK----L---------------

-----------------------

>pCRESS4|WP_000818357.1

M------------------------------------AEKKIRVRARVYSVVQYEFNP----RTGEDLF---

----------------NE-QVIKNAI-----LNKETSLDNWAYIRHDKDKYVEGDDIPE---GSKIGD--V-

RPPHWHVLLKFK--NQIEFSTIAKLFN-----VPE-NLVE-----------KKT--GAGAFFDYLYYMTHED

----------------DKQRKLG--KHVYERDEVEMIN-------EVTANEMWEHVDIREDRRAKKLSK---

------GETVE-VFLDKLTSGKM-TMKQ----VFERDS---------VVFA--------EN--ATLFRRARR

SYLKY-APTPL-VRTNYHISGAGGTGKTLIAKSMARAM-FPDKF---DD---EI-FFVVGDG-RV----AFD

EY-DGQPIIIWDDFRA---KE-LLNAFERGTMWKIFAIHPDKVS-VHVKNGETTLIN-T---VNIITSVEPF

TEFVN--GLAA-AFK---DN------KS-EDVGQAYRRFPIFIEV---SKNNFKMFATLGLT---GGEREQY

EPLINIEVNTIELA------------------------KNQ-SKINNQKVFKNVISVHNK------------

------------IVN-VNSKNDEIKEIEHVEE-TTDFFNKIIIDESKGDKDV--------------------

-----------------------

>pCRESS4|WP_000186194.1

M------------------------------------TEKTKSKRVRRVKIIQYENNP----VTGESLF---

----------------DE-QNILNAL-----AYFSHRTKRWAWVKHDKDVITEADIRDE--SPEDIGK--P-

KGTHYHVVMELT--NPASISAIAKRFG-----VPA-QYVE-----------VIETKTHDAVLDCIAYLTHED

----------------AKQQQYG--KHLYDRDEVYISD-------SN----IWIDVDNQKAREALTKGGS--

------RADAR-VIIEKISQ-GM-TLSQ----VYEFDN---------VMAV--------EN--KNLFKTARQ

EYLKN-APVPP-VRTNYYVYGEGGTGKSLSAKVLARSL-RPDITK--DE---EL-YFVVGDG-AV----PFD

GY-DGQPIIIWDDWRA---LD-LLSHFDRSLVWKLFAINPERIS-VNVKYGSTSLIN-A---VNIVTCVDPY

LKFME--ELAG-EYT---DKRGTKY-KK-EDSRQAFRRFPFFIEV---TAESLLIARNRGLS---KNEIAQV

EPLLKFENGMMQLA---------------------KQQERGGRKEVADILEQPL------------------

------ALMESEAKKYQGTHDDDFEPIKALKILPLDDEC---------------------------------

-----------------------

>pCRESS4|CBL40434.1

M--TNENMTTATTG------------------------TTEPKKTYRTFEIMQYEYNP----KTGEDLF---

----------------NR-AVIMKAL-----AH--KTIKQWIYVRHDRDKNDD-------------GT--P-

KAPHWHVYIYCN--PAKSLDDISKWFG-----VPT-NMIE-----------LKV--GKHSFLDCAEYFTHEK

--------------------QPT--KALYDDTKLY-SN-------MP----WRQMLNERDEKRAKYGDED--

------IQFRD-LQRVDVLNFGK-TLKQ----CKIDDP---------VLYV--------KE--MQILKKCRL

EYLYT-QPVPK-SRMNIYVTGQGGVGKGHTCKALARAL-YPELDD--DE---DI-FFTIGAK-NA----TFE

GY-DGQPVIIWDDRRD---YS-LLEELGRENVFNVFDTIPQNLR-QNIKYGSVKLLN-A---INIVNSVQPY

TEFLD--GLSG-EYK---AKDGTVY-KS-EDKQQSYRRFPFCIEV---KNFSYVFYENQGFVNHDKKDYLHI

T--SSVRR-------------------GNADEVHAYFTDRKKIREMEAKLFAYPIQTIKE------------

------------------------------------------------------------------------

-----------------------

>pCRESS4|CRY93789.1

M-------------------------------------------KARIFNIMQYEKHP----ETGEKLI---

----------------DE-DVIKVAL-----AH--KSIKQWAYIDHDADVYSLRDEADD--GRRKAGE--T-

KPKHWHIVCRCQ--AAVEVSTIARWFK-----IPE-NFVD-----------VPK--GQGAFLDCVEYLTHER

----------------EEQQALG--KRLYEDERVR-AN-------FD----FREDLEERAEKRLKYGRD---

------TDPKT-AMWYDVMFNGL-TLKQ----ALERDR---------WAYM--------EM--LEKLKKARL

DYISR-MNPPD-TRINYYVEGKGGVGKGLISRAIARSL-YPQYDD--DY---DI-FFEVGAK-GA----PFE

GY-DGQPVIIWNDRRA---YD-LLQELGRGNVFNVFDSHPTKQR-QNIKYGSINLCN-E---VNIVNSVQPY

AEFLD--GLAG-EYE---DKNGNKR-GV-EDKGQSYRRFPFMVIV---HEEDFDFMINKGFIVGEKADYSEY

LKHRNICANFRRIH-------------------EVCGQNEKLAKQIETKAVKKITDTHNE------------

----------------------------------TPREPTFGEWLDENNKKE--------------------

-----------------------

>pCRESS4|WP_021639163.1

M--EKRGKGGAFGARKILGKLIIRS-------------IMAKKKGFYNYNIMQYEKHP----ETGDELL---

----------------TE-EKIKSVV-----AKY-KSIDRWAYIIHDKDVYTEANEEAN--PDHKAGT--K-

KPKHFHIALKMKD-NCIELDTVAKWFG-----ILP-NYVE-----------IPK--GRGAFLDCVQYMTHER

----------------TEQQEQG--KFRYADLEIQ-AN-------FD----WRAELDKRDEMKAKWGKGE--

------LSDRQ-VMGQRIMLEGL-TLRQ----VKAEDP---------LLYA--------DN--LEFFRKMRG

VYLSD-LEPPK-TRINYYLCGDAGAGKGVMSKAVARAL-FPELIN--DE---EI-FFEVGAD-NA----LFE

GY-DGQPVLIWHDRRA---GN-LIKELGRSNVYNVFDTHPSKQR-QNIKFSSVGLIN-R---VNIVNSVQPY

VEFLE--TLAG-AKD---DNDSTN--DG-AEKSQAYRRFPLIINI---HPEEFDIYINKGFI-SQHETYLEY

EEHKGFYANMRKVA-------------------ETCSKYETLRREIEGMVVKPVVEQHHR------------

------------------LDERKTKEQLDAQRKRHERAASLRKWIEENPLIYAMGIIPYEGPSPFEE-----

-----------------------

>pCRESS4|WP_007889993.1

M------------------------------------------VMPKVFNIMQYCKHP----ITGEVLI---

----------------TE-EQIKSLF-----D--RRTIKLLAYILHDEEDFDEEDEENDEEHWKKAGD--K-

KPPHFHVVFRTD--RNTDLETVADWLG-----IPV-QYVD-----------YRKRDGQLTFVDLLRYLTHES

----------------EKEQAKG--KHRYPDEKVI-AN-------FD----FRAMIDEADIREARYGN----

------KSPKD-YYRHKVAYEGM-SISE----VIAENE---------DAYL--------KD--MTFLDKCRS

KYLAAFAKMPD-LRINIYLDGAGGIGKNTASKAIAHVL-YPDME--------KA-YFEAGGA-NT----SFE

GY-DGEPVIIWNDCRS---TD-LVQRFERNELFDILDPHPTDAR-HNIKFGSVRLTN-P---INIINGIEPY

NKFLD--GLAG-AYV---DKRGVMH-SG-EDSSQAYRRFPIIMCL---REDDYDLLFNKGVF-NGTREYMEY

ISYNGLVGSFAKVS-------------QRLAG--------QAKEVVIVDMTKPVLDSVIK------------

------------------LKDNDIKKIEDVEDIPDEFKNYGKKKEDVQTSEEKAKNWVWTPGK---------

-----------------------

>pCRESS4|CRY97508.1

M------------------------LIYEVIYSEKMTAQPKKELKSRIFNCLQYEKNP----KTGADLF---

----------------TE-ANILKCV-----AH--KSITRYAYIRHDKDVVTEWDVENG--TEADLGQ--P-

KGVHWHIVLETAK-GLMPVSTIARWLG-----IPE-SMVE-----------IPK--GRGAFIDCVEYLRHSD

----------------IRQELKG--KYVYEADEVK-AN-------FD----WQTEVTEMVLRKTKYERP---

------LSQAD-FLKNEVLYNGM-RLAE----VQERYP---------SIYM--------KE--QTVFDRLRM

KYLVERAPLPA-SRINFYIEGLTGYGKDTMARSIARGL-FPELAKQCDE---DV-YFEIGGK-KV----TFD

SY-DGQPVIIWSEFRA---ET-FVNALGYEEVLGAIDIIPKNNR-HHKKFGAVKLIN-S---VNIVTSTEPY

AEFLK--GLIP---------------ESDPDPSQANRRFPLIIPI---HVKDFDILINSGYL--GADTYRDY

TAYKNIVGSFGALA-------------------RRLNTRPELLIRAESELIKPVIDAHGV------------

EFKGMSDEEILDVFRHEGYGIEKTYRDKTEDELKAEYQTYLKRCYENDKASCKAYTDTYNEWLGSYWVNECM

PYEVWLSQTARKICDE-------

>pCRESS4|CDA18875.1

M--------------------------------------STNKITSRTWELVTDVKHP----ETGEELI---

----------------NK-EKIDTVL------KSHASIKEFAYILHDKDTYTDEDIKKM--STHSVGD--I-

KPAHFHVVMRFA--RAQELDSLSEWFG-----IDK-NFFE-----------KKK--GRNSFFDSVLYLTHQS

----------------DKEQSKG--KFVYSEDEVF-CH-------FEEYSSFREFVEACEINKEKYGKAN--

------ICIKD-KLRLDVLYNGM-SIRQ----VKKNYP---------MEYN--------DD--MEGLQKRRG

DYLKD-APLPP-YRISFYISGSGGAGKGLFSEALARAL-IDPDGEMCDD---EI-FCYVGSD-SV----CFE

NY-DGQPVLIWDDCRH---NE-LFKKLDRGTIFNVFDTKPKRIS-QKKKYSQTNLIN-P---INIVNSVEPI

NGFLD--GLAG-QYY---DKSESRY-EN-DQKAQSYRRFPICINV---HPTYYDIHVYEPFFGGEYSVIYKY

K--------------------------PFVDMVRTYGSDSKEYKDCCLKALKPVIELFEQ------------

------------------------------------------------------------------------

-----------------------

>pCRESS4|WP_017824301.1

M--DSADPEPTEVDPGGNQYAEG--------------WHYAEQIKGRIFTITTDCVHP----DTGEVLL---

----------------TV-ETIEKRL-----SK--SSVEYFAWVLHDKDCFTESDLEGN--PRAVLGE--H-

KPDHFHVVMQLK--NQASVGQVARGYK-----LHP-GCVR--------------KEGQGTFLDCIEYLTHEH

----------------EKQQKLG--KHLYADDEVH-SN-------FD----WRMAVDERVAARKQGFHSGAA

------AKKM--KIRLAVMNGQM-TLKQ----VREDEP---------GVYV--------QD--LEKLQKLQQ

DFRLH-QPAPR-HRTNYFIGGMAGTGKTQLAKLFARTL-FRDLDAD------EC-YYVATDP-RV----PLQ

NY-KGQPVIIWDDYNA---LD-LMEALGRSGVWQVFDDHPSATD-VNIKYGATRLVH-T---VNIITKTTPY

AEFLD--GLAG-EYT---DKSGKQH-KA-EDRNQSWGRFPVVFEV---TIHSIVMLLNRGFV-DDTDDFLAY

EEVARMRASMREVC-------------AGLDSIEDDAEREAATYQVGDFLLRPMLDQHAN-QPVASRKATEV

VGELLSGIEILDGEALEKDAKEEAGRASDLKRLEAEALAHAKTIGAPVEYLTPSGQRAAMCPDGVTWGWTNG

SPLHLVEGGAR------------

>pCRESS4|CEI31812.1

M--SGRRARLRRKAAQEHASATGAAMQSEDTTDPASDYADSDDLGGRVFLITQNERYL----DTTRVLM---

----------------SE-ERIGKAV-----GK--KGMTDWAWIKHDQDQYTVEEAEKL--PGVVAGH--R-

KADHFHIAVRRK--SFSTLGQIARAFG-----VPP-NAVE--------------IKPQGAFMDLIEYLTHEH

----------------PNQIAAG--KYHYDDGKVH-AS-------FD----WRPALDEHKLARAAKAGQRAS

------LKKRD-AVREAVMLGEM-TLKQ----VREDER---------AIYI--------QD--LDKLQKLRQ

DFMLH-QPAPR-HRTNYYIGSPAGTGKTQLAKLFARML-YPDLDAD------EC-YHVATDA-RV----PLQ

NY-KGQPVIIWDDYSV---PG-LLAALSREGVWQVFDDHPSASD-VNIKYGSVRLVH-A---VNIIAKTTPY

AEYLD--GLAG-EYT---DASGNVH-EA-EDRNQSWGRFPVVFEV---TPDSIEMSVNRGFV-NDTDDFLAF

QKVARMRASMKAVS-------------RGLDAIESDDEREAATFQVGEVLLRPIIDQHHA-QPVADRAGADV

VGELLAGIEVIDGETLEKHEAEEAANAAEAADVADVDRRLRRQDLESVATELAARNGCQACYSDVLSLPEYD

GHVLLPSGWMCGSGHAVAVHA--

>pCRESS4|WP_067940518.1

M----DFAEMDVPGADPGDEVEAVEKAAESSE-----QVKRARMQSRVFSVMQYREHP----ETGEVML---

----------------TQ-EQIDEGL-----AALGDRLHRWAYVWHPYDRLVEVDEGTG--ETVCCGI----

KGLHAHMVLWVADDPRPTIRTVSDAFS-----IPS-ARVK---------PPEHKGAAEKAFFDLAEYLPHES

RGRDATPGDRHYLVDKTQEGKPG--KYQYGRGRIV-AN-------FD----FGRELDAHMAMRRTAAEGGG-

------SAKLS-KLFQAVDKGSL-TLKQ----VRDQEP---------AIYF--------AKGNLAHFQKLRG

DFLSH-QDAPE-SVMNFYVFGEGGTDKDLLAKALARAL-TPDAD--------KP-YFKVGGE-NV----SWE

GY-DGEPVVIWEDMRV---GD-MIRTASRGMLFRILGPWREPKPVVNIKGSKTQLLN-R---VNIVTGPEGY

EEFLR--GLAG-EYESMQGGVRVKH-HP-ENLGQGFRRFPVIIPV---AEREFSIFVNSGVL-NGTREYQSY

ERYEHMRQDLELLA-------------RKCKAIKDTAERERVRGAIEARTVAPIVEQHDRIPQSSVEVAVAP

SEPSPRDRCICDEWQ------------------------GYALDHTESCPIRTDAEAALAEMRQREAEREAA

AEVHRQAELERKVARLRANGG--

>pCRESS4|WP_005464724.1

M----DFAELDVPGADPGDEVEPVEKTAESPE-----QAKRARMQSRVFSVMQYREHP----ETGEVML---

----------------TQ-EQIDEGL-----AALGDRLHRWAYVWHPYDRLVEVDEGTG--ETVCCGI----

KGLHAHMVLWVADDPRPTIRTVSDAFS-----IPS-ARVK---------PPEHKGAAEKAFFDLAEYLPHES

RGKNAIPGERHYLVDKTQEGMPG--KYQYGRGRIV-AN-------FD----FGRELDAHMAMRRNAAEGGG-

------RAKLS-KLFQAVGKGSL-TLKQ----VRDQEP---------AIYF--------AKGNLAHFQKLRG

DFLAY-QDAPE-SVMNFYVFGEGGTGKDLLAKALARAL-APDAD--------KP-YFKVGGE-NV----SWE

GY-DGEPVVIWEDTRV---GD-MIRTASRGMLFRILGPWREPKPIVNIKGSKTQLLN-R---VNIVTGPEGY

EEFLR--GLAG-EYESMQGGVRVKH-QA-ENLGQGFRRFPVIIPV---AEREFSIFVNSGVL-NGTREYQRY

ERYEHMRQDLELLA-------------RRCKAIKDTAERERVRGEIEARTVAPIVEQHDRIQPSAEEIAAA-

EEAAKCDREVVEEQQRRRLAELEEHNRELQLCTCSTPQAGVYARHGDECLALSEDE------RQRRAE----

---AKQKVLDAKVARLRANGG--

>pCRESS4|WP_043534193.1

M----DFAELDVPGADPSDDVAPVEKASESAD-----QKKRARKVSRVISVMQYHQHP----ETGEVIF---

----------------TQ-EQLDEGL-----AALADRLYRWAYIWHDSDRLVEVDEGTT--EMVCCGL----

KGLHVHIVLWFKDDPRPTVRTVSDALT-----VPS-PRVR---------VPKNAGAAEKAFYDLCEYLCHET

RGASGIVGDRHYLVDKSQQGQPG--KYQYGRGRVV-AN-------FD----FSASLDAHMATRHDAAEGGT-

------GAKLS-KLFQAVGQGTL-TLKQ----VRDQEP---------AIYF--------AKGNLAHFQKLRG

DYLAY-QDAPE-SVMNFYVFGEGGTGKDLLAKALARAL-APDAE--------RP-YFKVGGD-NV----SWE

GY-DAEPVVIWEDMRV---GD-MIRTASRGMLFRILGPWREAKPIVNIKNSKTQLLN-R---VNIVTGPQDY

EEFLR--GLAG-EYESMQGGVRVKH-EA-ENLGQGFRRFPVIIPV---AEREFSIFVNSGVL-NGTREYQSY

ERYEHMRQDLELLA-------------RRCKAIKDTAERERVRGEIEARTVAPIVEQHDRIQPSAEEIAAA-

EEAAKRDREVVEEQQRRRLAELEEHNRELKLCTCATPQAGVYARHGDECPALSEDE------RQRRAE----

---AKQKALDAKIARLRANGG--

>pCRESS4|GAC78794.1

MPETSDFAELAVPGADPGDDVLPVEKGAESKA-----QKSRARTQSRVFSVMQYRKNP----DSGVVML---

----------------TQ-EQIDQGI-----KTLGGRLHKWAYIWHPYDRLVEVEESTG--EVTCCGV----

KGLHAHMVLWIADDPRPSIRTISDAFA-----IPS-AVVR-------TPKEEHKGAAEKAFYDLCEYLPHES

RGSDAIPGDRHYLVDKTQPGKPG--KYQYGRGRVV-AN-------FA----FGRELDAHMATRHNAATDG-G

S-----GAKLS-KLYQAVGSGSL-TLRQ----VREREP---------AIYF--------AKGTIAHLQKCRD

DYLLR-APLPP-FRTNYYIGGPARTGKSTVAETLARQL-YPGLALD------EA-IYMVGRP-GV----AFQ

SY-DGQPILIWDDYRP---LS-IIEAIQRDSIWPVLDIDPKRVQ-VNKKFGAVSLLN-S---VNIITGIQSY

VEFLD--GLAG-EYTDKKTGERV---EV-EDKDQAYGRVPLVAAV---TSETIDFYLNRGFA-GRSDSYQDF

DPVARIRANMGRVI-------------DTIDALPTEAAKEQFREAAGARMLGGMVQAHLD-PTPAALTAAEA

LAELEATTTVEGPEELAAEALANDSATAAEAMVAADERSAQERYACADGPAWSRNPFVPEAQPEMARYRTER

L----------------LSHP--

>pCRESS4|WP_006681830.1

MPSAPTLAPEGGTPSCENDCLSTWNTTKNDWN---SEYANEP--GGRVFMITQNLSHP----STGQTLI---

----------------TT-EQVGKAL-----AK--KGVKRFAWILHDKDVYTSAEVAKN--SALVQGS--P-

KAPHVHVVIQRS--SFASIAQVARAFG-----VPP-QCVE--------------PKPPSAFLDLVEYLTHEN

----------------PKQQAAG--KHLYDDSEVH-ASKG-----WD----WRTDLEEHKIARQEKGLGKAL

------QRRRK-EAALKVAGGEW-SLDH----VRKHDL---------ELWS--------APGVMSHLKGLRA

DYLAS-LAPPL-EVVNFYVFGPGGVGKDLLAHALARSL-NPTAE--------KP-YFTVGGS-NV----SFE

DY-DGEEVIIWSDFRA---ST-MLDACDRGLLFRVLGPYRDAKVIVNVKGSHTQLVN-R---VNIVTGPDDY

KTFLN--GLAG-EYA-YTNRMGVKV-TS-ENKDQAYRRFPLIIPV---QEGEFSIYVNLGFL-NGTREYDQY

ERHERLRHNLELLN-------------RRCQAITDAAERESAIRQIEARTVAPILEQRARVIEPEPTPEEIE

AGERAEAN-----------ARTWAVAHQALDIIEADRRAAEYKAAEERRKANELAREQREREMLAAKKAELA

AAGQLDLIPKVGIEYIDGKPR--

>pCRESS4|WP_052038917.1

MPEGPTLAPEGGTPSCENDCLSTWNTTKNDWN---SEYANEP--GGRVFMITQNLSHP----STGQTLI---

----------------TT-EQVGKAL-----AK--KGVKRFAWILHDKDVYTSAEVAKN--SALVQGA--P-

KAPHVHVVIQRS--SFASIAQVARAFG-----VPP-QCVE--------------PKPPSAFLDLVEYLTHEN

----------------PKQQAAG--KHLYDDSEVH-ASKG-----WD----WRTDLEEHKIARQEKGLGKAL

------QRRRK-EAALKVAGGEW-SLDH----VRKHDL---------ELWS--------APGVMSHLKGLRA

DYLAS-LAPPL-EVVNFYVFGPGGVGKDLLAHALARSL-NPTAE--------KP-YFTVGGS-NV----SFE

DY-DGEEVIIWSDFRA---ST-MLDACDRGLLFRVLGPYRDAKVIVNVKGSHTQLVN-R---VNIVTGPDDY

KTFLN--GLAG-EYA-YTNRMGVKV-TS-ENKDQAYRRFPLIIPV---QEGEFSIYVNLGFL-NGTREYQQY

ERHERLRHNLELLN-------------RRCQAITDAAERESAIRQIEARTVAPILEQRARVIETEPTPEEIE

AREKAELS-----------TRTWAVARQVLEVIEADRRAAEHKAAEERRKANELARKQREREMLAAKKVELA

AIGKIDLIPKVEIEYIDGKPR--

>pCRESS4|WP_044572803.1

M--SDTESNVESTAKSFLTESVDDSTNAGGTEAPKSAWHYAEALKGRCFEIVQDEKHP----DTGAILL---

----------------TR-ERIEKVL-----AKRP--NDLHSWVRHDRDQYTDEDLVKN--PRAVLGE--Y-

KVPHFHIAEKRK--NEASVGQVARAYD-----VAP-QYVR--------------VKPITAFLDLVDYQTHGL

----------------ERQQDLG--KNLYDDSEIH-AN-------FA----FREEVDKRVAKRINSGTGP--

-REGVKQTPID-KLAMRIQEDGL-TLRL----AKEEDP---------LSFN----------RAPGRMEKSRA

TYLRH-LPPPS-SRINFYFEGEGGVGKDLLAKALARTL-IPGNWVPGVN---DP-FFSVGGE-NV----GLD

GY-DGQPVIIFEEARA---GN-LIRSMGRKELFAFMNPFPEKQS-LNVKYGATQPVN-T---ITIFTGPDDY

DTFLD--GLAG-EFI---DKSGERH-KA-ENKPQARRRIPIIIPV---REGSFDLLVNKGFA-DNTRDFMEY

HVYRNIRQNIEQVQ-------------IRSKGILDEQRRIEIHRAIEAGQVAPIVEQYHR------------

------------------------------------------------------------------------

-----------------------

>pCRESS5|WP_024390948.1

M--SGKNK------------------------------------RSRLFFGMRNYKFE----THDTEG---I

TWDI---------TEDDWKNEIKKQFEAVSDPQP----TELTYIFHDKDIDTD-------------GE--K-

KALHVHFVARFE--NAIYYDTTIEKFK-----CEP-RNFE--KG--------------RSETSALLYLTHTT

----------------SEAIKMK--KRRYNVSELN-VLTFLLG--EELEDWYR---LKIAGREGSNKVST--

------DEDVA-RIIDELSEGLM-TIDDVKNDL--KQAFD--PTTATMTWM--KNKRYFKEAVAEYYQNKY-

---YDWLEKGR-TFQLVYIQGSSGIGKTSFAREIGKEFNLRATAIHNAP------NDTKGAR-YD----FLS

GY-ENEAVTVFDDLRP---NT-F----GYTEFLNLFEKERV-SK-YSSRFNDKAWFA-E---VAVITKSTSI

NDWTS--KLAYSKSASASD-------KP-NVLYQPRRRFSLIIDV---NDDEVVLSSYVLTDRKKMLHELQP

I--------------------------FKCPKKTDDEKSGFWDKKFQKKLLKAVMVSLGF------------

VEPTKADLKSVSIEADENTVKKAEELLAK-QKGGS-------------------------------------

-----------------------

>pCRESS5|WP_029176301.1

M--TTKRK------------------------------------RARLYFGMRNYKFE----THDQEG---R

VDDI---------TEKDWREKVSQELENVQA-------DELTYIFHDRDIDTD-------------GE--K-

KALHVHFVARFA--NPMDYEPTREKFG-----CEP-RNFE--KG--------------RSESSALLYLTHTT

----------------PESIKAK--KTRYNVQELT-VVTIREG--EDLEEWYR---IKIAGKAGSLKTNT--

------DDDVA-SIIDELAEGTM-LLTDVKEEL--KQRFD--STTATMTWM--KNKRYFKEAVAEYYQDKY-

---LEWLEKGR-TFSLIYLEGPSAIGKTKFANKIARRVNIPEGWVHNAP------NDTPGAR-YD----FLN

GY-EQEVVTVFDDLNP---KT-F----GYTEFLNLFEKERV-AK-YSSRFNDKAWFA-E---VAIITKSTSI

DSWTT--SLSYSKTDKSGK-------TA-NILYQPRRRFSLIINI---EHDLVKISKYVLINPKTNAHELQV

L--------------------------FKPPKGTF------FDDNFQEEVLDEISKYLGL------------

TEATEEDVAEV-LQADSDTVDRLSALLEDSQNGENVQEEF--------------------------------

-----------------------

>pCRESS5|WP_024393234.1

M--SKTI-------------------------------------KSRLFFGQRNYDFE----THSFNEKNTK

ISDI---------SKDDWKEKIKKLFEEIDEY------SFLALIFHDRDIKES-------------NE--L-

KALHCHFVIRFN--NPRSYSNILELTK-----CEE-RNFE--RS--------------TNEGAILRYLTHTT

----------------PEAMRAE--KTRYNVSELL-VKEK-----ESLERWYR---KKIKSNIGKKETDTKV

------VDFVN-DLAYRLSVGEF-KPINAREMLI--AEFG--NEFGQSIYR--KEKKKFQEDYADFLETKK-

---KDLLLNGK-ELSTIYIDGFSEVGKSTFAQDLANAINKEVLDTYLAAK-----KKAGAQS-WD----WIS

KY-KDEYITIFNDVDP---YD-F----NFTYFLGTFETKIL-VD-VGSRYKDKTWFS-E---YAIITKSTDI

HEFVN--KICGNREDNRQE-------HF-NIRYQVQRRFSLIIKI---EKNKVTLSKFNKKGLLKTKATFKF

D----------------------------------DLQKEFWNGSIRKEIIQECLSLLDL------------

------------------------------------------------------------------------

-----------------------

>pCRESS5|WP_050238550.1

M--SKI--------------------------------------RSRLFFGQRNYEYE----THTLNEENEK

TSEE---------TESEWKQRILELF-SISDY------NYLALIFHDRDIKEH-------------NE--L-

KGLHCHFVIRFD--NPRSYDSILELTS-----CQE-RNFQ--RS--------------TNQGAILRYLTHTT

----------------PEAMRDE--KTRYNVSEIY-LKTD-----EELEVWYR---TKIKANLGRKEADTKV

------TDFVA-DLAYRLSIGEF-KPHTAREKLI--AEFG--NEFGQSIFR--KEKKKFQEDYNDFLDSKK-

---RDMLLNGK-ELSTIYIEGPSEVGKSVFAQDLANSINRDILDTYLASK-----HNKSSGT-WD----WIS

KY-KDEFVTIFNDLDA---NL-F----SFTDFLGTFEQKIL-VD-VSSRYKDKTWFS-E---YAIITKSSDI

DEFVN--KLCYSREDNKSE-------HQ-NIRYQVQRRINLIVKI---EKNKLTLSQFNKLGVLKTKKIFEY

D-----------------------------------NINDFWHKQIRKEIIDECLTLLNI------------

------------------------------------------------------------------------

-----------------------

>pCRESS5|WP_061866456.1

M--AK---------------------------------------RVRRFSISRNKQYE----THQLDG-GQQ

VSEI---------SDTDWKEKVKSTFEYLATEYGS--LKSIALIFHDRDLTSD-------------GE--R-

KGLHCHMILEFR--NPVTITSLEKFKFEAGKSFQS-RNVE--AS--------------KSSSGSYRYLTHTT

----------------DKAMMER--KTRYEVQELI-VAEYITG--EDLELWYR---DKIKGTIRPEKLEF--

------DEALQ-EAFFKVRTGEI-FDEEVEAFL--RERFT--EMQATELVI--KNKKFIDNSRQMYQKEVF-

---EDMQNNGR-NLKTFFISGSSGLGKSRFAKDLARRINKSINSIYTAP------TAKDGKT-YD----FDS

EY-KAQDVTIFDDVDA---KS-F----GFQEFLNIFDKDNI-TK-ISSRYTNKAWVS-H---YAIITKASKI

RNWIE--RVASQEYEKD---------KR-GQEVQVSRRFDLWIEL---DFDNNNQVNFYQFKHYPDDNKKSQ

W--------------------------KKVARKEITMKEFQSDSSAREEIFDII------------------

------------------------------------------------------------------------

-----------------------

>pCRESS5|WP_058211405.1

M--AKNGK------------------------------------RFRTWNITLNYDYE----IHNEKG----

------------MTAEEWKNKIRQLIAFNTDD------GWCAYVFHEKDILED-------------GL--P-

KSLHVHILKNFK--EAKTQTAVMKMFN-----VSREANCT--NA--------------RSITSSARYLTHRT

----------------SQAMDDG--KHQYNIDEVQTIN-------CD----YLELIKNKSDRTISQKEV---

------DEIVM-GLSINIGNGKL-YWLKARELLI--DKFD--EIEGIKLWN--KYSRIFEKNFKEYIQHKA-

---EDYKLKGR-NLTTFFIWGDSEVGKTWLAKCMCLLL---------SDRIHMVPASGKNKT-FD----IAG

LY-DGEKASLWNEVSG---LE-L----SNKEFLDRFDPKTY-SP-SNSRGKDKHCLS-D---YFFLTSTDDL

ETVVN--NLMPNIEEF----------EI-KRRHEINRRLPIEIKC---INLGYKQTEFVIRLYDPKNRDRFT

---------------------------LCSVICKNIESENQMKKAAKEILTILG------------------

------------------------------------------------------------------------

-----------------------

>pCRESS5|WP_017368666.1

M--EQAK-------------------------------------RFRTYNITLNYDYE----VHDEQG----

------------MTKEQWKEKITQLIASNVSHK-----DWCAYVFHDKDLLTD-------------GL--P-

KPLHVHILVGFE--NGKTQTAVMKIFN-----VSRPKNCQ--HT--------------NTIAGSARYLTHRT

----------------EQAMIDG--KFPYEVHAVITIN-------CD----YLTLIKGKEKVNKQRNI----

------DEFVA-ELSEKIYLGKL-YPTNIRELFF--KEFD--RSLADRLYK--KYRKEFEKDFQEYLQHKG-

---EEYKIKGR-DLATIYIWGVSEGGKSHIAKRMGLLL---------TDRLHLIPASGKNKT-FD----PSG

LY-QGEEVSIFNEISG---KE-F----NNKEFLGLADPRNY-SP-INSRGKDKHWLA-S---YLFLTSTDSR

ETFIK--NLIPKQTDSF---------EQ-RQRHEIARRVPYEIRC---VSLGYMRTQFHLGIYDSKNQEMFS

---------------------------VGSVICEHIDDEKAMQKSALEILKKFG------------------

------------------------------------------------------------------------

-----------------------

>pCRESS5|WP_038978316.1

M--SNYQKDDDFLAT-----------------------------RARTFSGQRNYEYD----THELDD----

------------LTEHEWRKQMVDMFTAIQDDC-----EYCYFIFHDKDVLPN-------------GD--K-

KGLHVHFVIKFK--NPRVIRSIMKTFG-----ISRTENIS--KV--------------KSVKGSLSYLLHIT

----------------KQARKDG--KFIYGQDRLYKVGTD-----DDNFEHFN---KLTVSDKDDKDIK---

------DEIVE-EVLSQVTSKGE-MAD-----IKILKKFPTHKNLVSDIYY--NYTTKREYAEREYFADKL-

---RYRNKHGR-WLRNLYITGKGGTGKTTLANKLGYAFAVHVGA-----------AKSPDKT-YD----PMG

TY-KNQKVTILNEMQG---SL-F----DYREIMNVFDDHQQ-AP-VSSRTKDINWTA-D---YLIMTSSKSF

ERFRN--ETLRYGGKHLVEELPTGQNGDEYKAFQFTRRFSNYIEIF--TSKGQKYINVFQFNFKKRGFVLQT

QRDADIDQNYKEYNVETLCNPHKEYCDDTLDKIQQESKADLDRNELDKFIEDNKKVGVTL------------

NDMIKMYDENPLKELEEKNAKRAQKIKENENFKELNERRGITEDMIDEFVKS--------------------

-----------------------

>pCRESS5|WP_046467524.1

M--AKVQKDDDYAKS-----------------------------RFRSFVIQRSYDYD----VTVYDETEKP

DDAP---------TPDEWMDKIIAQFKKEGAK-----ADYYYFIFHDADYLPD-------------GT--L-

KSLHVHIVIHFK--NPRTVGAVYKAFG-----VSRFENIS--KA--------------KSIKGALKYLLHIT

----------------PQAINDG--KTVYGMDKLY--------DLDDVYSHYKELINDNKNQEEAEEKRVQK

QV----IARCK-SLLTDIATKGT-APGEWLETLW--EDFEDYEDIVSDVYV--NYKKKFQEMEKEYFYRLT-

---QKKKREGR-DLRNIFVSGEGNSLKSSVAKEIALRYAYHIAS-----------PPSDGKT-YD----FVS

LY-KNEKVSILNEMVS---DA-F----NPREFMNVFDDFQI-GS-VSSRFKDINWLA-D---KTVMTTSDTF

SEFRS--NTFRYGGSQYYDKYGKFI-EK-DVFYQFSRRVEHYVRL---DNLDKKVMSVYHFDKKKYGYVLQG

VEMLNQKSNYPQFLVDAIYNTDEIREDEREKEQQEHKLTINNQEELESFVVKKLQQNITL-----KDKIVEH

EEKVKEEELKRKERIRQSNAKDTLRRNVLKKHEAFNAKVGVTEKDKVEFLEQK-------------------

-----------------------

>pCRESS5|WP_061417941.1

M-----K---INQND-----------------------------RKRKFFGQISYEYE----HNANSE----

------------LTEEDFRDSVIQRIKDYCKNED----DRYHIIFHDKDLKDD-------------GS--S-

KPLHAHFYIDFK--HAHTYSSVYKALS-----ISREQNLE--FV--------------RSSIKTCRYLTHRN

----------------ERNMEEH--KFPYEVKDVI-SSEN-----AN----YIDDIMGKIKNHSKEKADNGL

EI----DEYCL-ELSYQISEEGL-LIMEAKQQLF--EQFT--QRIAQKAWN--SNKRQFEENRQEFIQKEF-

---IRMSKGER-NHTGIYIQAEGGTGKSYLARLLAEE----------HDRLGAHTPSINKKR-FD----LGS

GY-KGEKTIVINELDA---SCGM----TFRELFQVLEPDSA-TQ-LSSRFKDAYIIN-D---LTIITNSDTY

WDWCD--SWFG---------------KKNIEYHQLMRRIRFVIKMVHDEKNKVIKIELWNYTATRDLKEKAK

QAFKKLET-------------------YTLNSV---ENEEEFRKIASDILERIK------------------

KEKNIDSKRKVITTD------------------STDQSE-CND--N--------------------------

-----------------------

>pCRESS5|WP_061863770.1

M-----K---INQND-----------------------------RKRKFFGQISFEYE----HNANSE----

------------LTEKDFRESVIQRIKDYCKNEE----DRYHIIFHDKDLKDD-------------GS--S-

KPLHAHFYIDFK--HAHTYSSVFKALS-----ISREQNLE--FV--------------RSSIKTCRYLTHRN

----------------ERNMEEH--KFPYEVKDVI-SSEN-----AN----YIDDIMGKIKNHSKEKSDSGL

EI----DEYCL-ELSYQISEEGL-LITEVKQQLF--EQFT--QRIAQKAWN--SNKRQFEENRQEYIQKEF-

---IRMSKGER-NHSGIYIQAEGGTGKSYLARLLAEE----------HDRLGAHTPSINKKR-FD----LGS

GY-KGEKTIIINELDA---SCGM----TFRELFQVLEPDSA-TQ-LSSRFKDAYIIN-D---LTIITNSDTY

WDWCD--SWFG---------------KKNKEYHQLMRRIRFVIKMVHDEKNKVIKIELWNYTATRDLKEKAK

QAFEKLQT-------------------YSLKSV---ENEEEFRKIASEILERIK------------------

KEKNIDSKRKVITTD------------------STDQSE-CSD--N--------------------------

-----------------------

>pCRESS5|WP_049478725.1

M-----K---INQND-----------------------------RKRKFFGQISYEYE----HNANSK----

------------LTEKDFRESVIQRIHDYCKNEE----DRYHIIFHDKDLKDD-------------GS--S-

KPLHAHFYIDFK--HAHTYSSVLKALS-----ISRKQNLE--FV--------------RSSIKACRYLTHRN

----------------ERNMEEH--KFPYEVADVI-SSEN-----AN----YIDDIMGKIKNHSKEKADNGL

EI----DDYCL-ELSYLISEEGL-LTTEAKQQLF--EQFT--QRIAQKAWN--SNKRQFEENRQEFIQKEF-

---IRMSKGER-NHTGIYIQAEGGTGKSYLARLLAEE----------HDRLGAHTPSINKKR-FD----LGS

GY-KGEKTIVINELDA---SCGM----TFRELFQILEPDSA-TQ-LSSRFKDAYIIN-D---LTIITNSDTY

WDWCD--SWFG---------------KKNKEYHQLMRRIRFVIKMVHDDKNKGINIELWYYNANRDLKEKAK

KEFKKIQT-------------------YTLSSV---KNEEEFRKIASDILEKIN------------------

KENNIDNKRKVIATN------------------LTDQSK-GSD--N--------------------------

-----------------------

>pCRESS5|WP_049535277.1

M-----K---INQND-----------------------------RKRKFFGQISFEYE----HNANQE----

------------LTEEDFRESVIQRIKDYCKNED----DRYHIIFHDKDLKDD-------------GS--S-

KPLHAHFYIDFK--HAHTYSSVYKALS-----ISREQNLE--FV--------------RSSIKACRYLTHRN

----------------ERNMEEH--KFPYEVSEVI-SSNN-----GN----YIDDIMGKIKNHSKEKADNGL

EI----DEYCL-ELSYQISEEGL-LVMEAKQQLF--EQFS--QRIAQKAWN--SNKRQFEENRQEFIHKEF-

---IRMSKGER-NHKGIYIQAEGGTGKSYLARLLSEE----------HDRLGAHTPSINKKR-FD----LGS

GY-KGEKTIVINELDA---SCGM----TFRELFQVLEPDSA-TQ-LSSRFKDAYIIN-D---LTIITNSDSY

WDWCD--SWFG---------------KKNKEYHQLMRRIRFVIKMIHDEKNKVIKIELWNYTATRDLKEKSK

QAFRKLET-------------------YTLKSV---ENEEEFRKIASNILKKIN------------------

DAKNINTK-KVIATN------------------PTDQSK-GSDNSN--------------------------

-----------------------

>pCRESS5|WP_067193806.1

M--AKNK---IDRNA-----------------------------RKRKYFGQISYDYE----YNANSS----

------------LTEDDFRKSVEQRIKELCQHEN----DKYYFIFHDKDLKDD-------------GT--P-

KPLHAHFYIEFK--NPRVYSTVYKALQ-----ISRQENLE--FV--------------RSTIKACRYLTHRN

----------------ERNMEES--KFPYEVSEVI-ASKN-----GT----YIDDIMGKVKNHSVEKSDEGL

EV----SDYCL-ELSYKISEDGL-LPSEAKQHLF--EQFT--QRTAQKAWN--SNKRQFEDNRQEYIQKEF-

---IRMSKGER-HHNGIFIFAEGGTGKSFLSRLLAEE----------HDRLGAHTPSINKKR-FD----LGS

GY-QGQKTIIINELDA---TCGM----AYRELFQILEPDSA-NQ-LSSRFKDAYIIN-D---LTIMTNSESY

WSWCN--SWFG---------------KNKKEYHQLMRRIRFVIKMYHDDTNQEIIIELWHYYATRDLKEKAR

QAFKKIDT-------------------FHLKDI---QDEEAFRNIASNILKKIQ------------------

E--TTLTPQKATADT------------------STDQSETTSD--S--------------------------

-----------------------

>pCRESS5|WP_044771983.1

M--ANA----IKPTD-----------------------------RKRKFFGQISYDYE----HNGDTS----

------------LTEVDFKQSVEKRIKALCSNQE----DKFYIIFHDKDQKDD-------------GT--P-

KPLHAHFYIDFK--NPRAYSAVFKALS-----ISRQENLE--AV--------------RSSIKACRYLTHRN

----------------ERNMAEH--KFPYEVSEVI-QSPN-----GN----YIDDIMGEIKHHSKEKSDNGL

EV----DDYCL-ELSFQISSEGL-LPLEAKENLF--EQFT--QRTAQKAWN--QNKRQFEENRQEYIQKEF-

---ERMSRGER-NHNSIYIQGDGNSGKSFLARLIAEQ----------HDRFGAHTPSINKKR-FD----LGS

GY-KGQKTMIINEFDA---SCGM----VYRELFQILEPNSA-NQ-LSSRFKDAYTIN-D---LTIITNSENY

WDWVD--DWFP---------------KK-KEYHQLMRRIRYVIKMYHDHQNK-LNIELWYYHGIRDLKEKSK

LQFQRIKD-------------------WMLDPV-TNDSEPELTMLAQDILSEIQ------------------

NRQFNHKQIKKVAVK------------------STDHSD-SND--NSKNLQD--------------------

-----------------------

>pCRESS5|WP_024408358.1

M--VKKKP--IDPNA-----------------------------RKRKYFGQISYEYE----KNADPN----

------------LTEDDFKQSVEKRIKELCQNDD----DVFYYIYHDKDINED-------------GT--P-

KYLHVHFVIIFK--NAHSYQSVYNALK-----ISRQENLE--VV--------------RSSIKACRYLTHRN

----------------ERNMAEG--KYPYSVEEVI-QSSN-----GN----YINSIMGKIKNHSTEKSEDGL

EV----DEYCI-ELSYLISSDGL-LPQEAKKALF--EQFT--QRTAQKAWN--TNKRTFEENRLEYIQQEF-

---EHMSRGER-NHNGIYIQGDGNSGKSFLARLIAEQ----------HDRLAAHVPSINKKR-FD----LGS

GY-KGQKTMIINEFDA---SCGM----AYRELFQILEPDSA-NQ-LSSRFKDAYIIN-D---MTIITNSESY

WDWVD--DWFP---------------KK-KEWHQLMRRIRYVIKMYHDDQNK-LIIELWYYHAIRDLKEKAK

LQFNKIKE-------------------WKLDSI-TKDSEPELTTVAQEILTEIQ------------------

NMQANSNQIKKVAVK------------------STDHSD-SND--NSKNLQD--------------------

-----------------------

>pCRESS5|WP_039694423.1

M--TKKKQKNIDPNA-----------------------------RKRKYFGQISYEYE----KNAYPN----

------------LTEDDFKKSVEKRIKEYCQNDE----DTFYIIFHDKDTNND-------------GT--L-

KTLHAHFYIDFK--NARPFSTVFNALK-----ISRQENLE--FV--------------RSSIKACRYLTHRN

----------------ERNMAEG--KYPYSVEEVI-QSSN-----GD----HINSIMGKIKNHSKEQSEDGL

EV----DDYCL-ELSYQISSEGL-LPLEAKENLF--EQFT--QRTAQKAWN--QNKRQFEENRQEYIQKEF-

---ERMSRGER-NHNSIYIQGDGNSGKSFLARLIAEQ----------HDRLGAHTPSINKKR-FD----LGS

GY-KGQKTMIINEFDA---SCGM----AYRELFQILEPDSA-NQ-LSSRFKDAYIIN-D---LTIMTNSEKY

WDWVD--DWFP---------------TK-KEYHQLMRRIRYIIKMYHDNHNK-LIIELWYYHAIRDLKEKAK

LQFNKIKE-------------------WKLDSI-TKDSEPELTTVAQEILTEIQ------------------

NMQANSNQIKKVAVK------------------STKQSD-SND--NPNKVGD--------------------

-----------------------

>pCRESS5|WP_033583888.1

M--VKKKA--IDPNT-----------------------------RKRKFFGQISYEYE----KNADPK----

------------LKECDFKKSIDKRIKALCKHDE----DVYYYIYHDKDKKED-------------GT--P-

KCLHVHFVIIFK--HAHSYQSVYKSLQ-----ISREENLE--FV--------------RSVIKACRYLTHRN

----------------ERNMAEG--KYPYNVDEVI-QSPN-----GN----YINSITGEIKNHNKEQSDDGS

EV----DEYCL-DLSYQISSDGL-LPLEAKKDLF--EQFT--QRTAQKAWN--QNKRQFEENRLEYIQKEF-

---ERMSRGER-NHNGIYIQGSGNSGKSFLARLIAEQ----------HDRLGAHIPSINKKR-FD----LGS

GY-KGQKTMIINEFDA---SCGM----SYRELFQILEPNSA-NQ-LSSRFKDAYIIN-D---LTIMTNSESY

FDWVD--AWFP---------------KK-REYHQLMRRIRYILKMYHDDYNK-LNIELWYYNSIRDLKEKAK

LQFQKIKV-------------------WKLEPI-TKDSEDELTLIAQELLAEIQ------------------

NNHINSKQTKKVAVK------------------STDQSD-SND--NPKNLED--------------------

-----------------------

>pCRESS5|WP_049481849.1

M--VKKKA--IDPNT-----------------------------RKRKFFGQISYEYE----KNADPK----

------------LKECDFKKSIDKRIKALCKHDE----DVYYYIYHDKDKKED-------------GT--P-

KCLHVHFVIIFK--HAHSYQSVYKSLQ-----ISREENLE--FV--------------RSVIKACRYLTHRN

----------------ERNMAEG--KYPYNVDEVI-QSPN-----GN----YINSITGEIKNHNKEQSDDGS

EV----DEYCL-DLSYQISSDGL-LPLEAKKDLF--EQFT--QRTAQKAWN--QNKRQFEENRLEYIQKEF-

---ERMSRGER-NHNGIYIQGSGNSGKSFLARLIAEQ----------HDRLGAHVPSINKKR-FD----LGS

GY-KGQKTMIINEFDA---SCGM----SYRELFQILEPNSA-NQ-LSSRFKDAYIIN-D---LTIMTNSESY

FDWVE--AWFP---------------KK-REYHQLMRRIRYILKMYHDDYNK-LNIELWYYHSICDLKEKAK

LQFQKIKV-------------------WKLEPI-TKDSEDELTLIAQELLAEIQ------------------

NNHINSKQTKKVAVK------------------STDQSD-SND--NPKNLED--------------------

-----------------------

>pCRESS5|WP_029171254.1

M--VKKKA--IDPNI-----------------------------RKRKFFGQISYEYE----KNANPK----

------------LTEDDFKKSVEKRIKEYCQHDE----DTYYVIFHDKDINDE-------------GT--L-

KPLHAHFYIEFK--NARPFSPVYKALK-----ISREENLE--FV--------------RSSIKACRYLTHRN

----------------ERNMAEG--KYPYNVDEVI-QSSN-----GN----YIEDIMGEIKNHSKEKSDDGL

EV----DDYCL-ELCYQISSEGL-LPEEAKERLF--EQFT--QRTAQKAWN--TNKRQFEENRQEYIQQEF-

---ERMSRGER-NHNGIYIHGLGNSGKSFLARLIAEQ----------HDRLGAHTPSINKKR-FD----LGS

GY-KGQKTIIINEFDG---SCGM----AYRELFQILEPNSA-NQ-LSSRFKDAYIIN-D---LTIMTNSETY

WDWVD--GWFE---------------EKKREYHQLMRRIRYVVKMFHNDQNK-LIIEMWYYHAIRDYKEKAK

LQFSKVKE-------------------WTLNSI-TKDSEIELTIVAQELLAELQ------------------

VNHTNNKQTKKVAEK------------------STDQSD-SND--NSNQLED--------------------

-----------------------

>pCRESS5|WP_053863690.1

M--AKNDLSGV---------------------------------KARRYIAQVSYTYQ----KNADPK----

------------LTEDAFREKIIKLVKALCTHDD----DKFYIIFHDKDIDQN-------------GE--L-

KSLHAHIYFEFK--NSRFYSSLFKTLE-----ISRDKNLQ--VV--------------KDKAKVCRYLTHRN

----------------EKDINEG--KYQYEISEII-SSKN-----SD----YRLDICGKSKRADKKKSEQ--

EE----VDCCL-EISYQICDQGL-LLNQAKEKLF--EEFD--RLIAQQLWN--RNKKQFEINRQEYIDKEF-

---AHMSKGNR-NHIGLYIQGSGGTGKTTLARFLAEE----------RDEHGQHAPSTNKTR-LD----LGS

GY-DGQRTIVINEFDA---SCGI----SFRELFQILEPDAV-TQ-LSSRFKDAHIIN-D---LTIMTNSDDF

IEWAD--AWFP---------------KK-KEYHQLMRRIPFFVKLL--TKNGKTIAELYHYKATKDKGDKCK

EAYEKVKS-------------------YNLGKT---LDEEHLRKQAKKLLSDIR------------------

SVHQKKIKKATTSKS------------------STDQSEKRSSNSFVRNKQ---------------------

-----------------------

>pCRESS5|WP_014735272.1

M--AKKDLSKV---------------------------------KARRYIAQVSYTYQ----KNADPN----

------------LTEDAFREKIIRLVKALCTHDD----DKFYIIFHDKDIDQN-------------GE--L-

KSLHAHIYFEFK--NSRFYSSLFKTLE-----ISRDKNLQ--VV--------------KDKAKVCRYLTHRN

----------------EKDINEG--KYQYEISEII-SSKN-----SD----YRLDICGKSKRADKKKSEQ--

EE----IDCYL-EISYQICEQGL-LLNQAKEKLF--EEFD--RLIAQQLWN--RNKKQFEINRQEYIDKEF-

---VRMSKGNR-NHIGLYIQGSGGTGKTTLARFLAEE----------RDEHGQHAPSTNKTR-LD----FGS

GY-DGQRTIVINEFDA---SCGI----SFRELFQILEPDAV-TQ-LSSRFKDAHIIN-D---LTIMTNSDDF

IEWAD--AWFP---------------KK-KEYHQLMRRIPFFVKLL--TKNGKTIAELYHYKATKDNGDKCK

EAYEKVKS-------------------YNLRKT---LDEEHLRKQAKKLLSDIR------------------

SVHQKKIKKATTSKS------------------STDQSEKRSSNSFVRNKQ---------------------

-----------------------

>pCRESS5|WP_018166163.1

M--AKKDLSEV---------------------------------KARRYIAQVSYTYQ----KNADPD----

------------LTEEAFREKIIRLVKTLCTHDE----DKFYIIFHDKDLDEN-------------GE--L-

KSLHAHIYFEFK--NSRFYSSLFKTLE-----ISREKNLQ--VV--------------KDKAKVCRYLTHRN

----------------EKDITEG--KYQYEISEII-SSQN-----AD----YRLDICGKSKKADNRKSEQ--

EE----TDFCL-EISYQISECGL-LLKQAKEMLF--EEFD--RLNAQQLWN--RYKKQFEINRQEYIDKEF-

---IRMSKGSR-NHIGLYIEGSGGTGKTTLARFLAEE----------RDEHGQHAPSTNKTR-LD----FGS

GY-DGQRTIVINEFDA---SCGI----SFRELFQFLEPDAV-TQ-LSSRFKDAHIIN-D---LTIMTNSDDF

IEWAD--AWFP---------------KK-KEYHQLMRRIPFFVKLL--TKNGKTIAELYHYKATKDKGDKCK

EAYEKVKS-------------------YNLGKT---LDEEHLRKQAKKLLSDIR------------------

SLHQKKIKKATTSKS------------------STDQSTKRSSNSFVRNEQ---------------------

-----------------------

>pCRESS5|WP_024382134.1

M--AKKDLSEV---------------------------------KARRYIAQVSYAYQ----KNADPD----

------------LTEEAFREKIIRLVKALCTHDD----DKFYIIFHDKDIDQN-------------GE--L-

KSLHAHIYFEFK--NSRFYSSLFKTLE-----ISRDKNLQ--VV--------------KDKAKVCRYLTHRN

----------------EKDINEG--KYQYEISEII-SSKN-----ND----YRLDICGKSKRADKRKSEQ--

EE----IDFCL-EISYQISECGL-LLNPAKEMLF--EEFD--RLNAQQLWN--RYKKQFEINRQEYIDKEF-

---IRMSKGNR-NHIGLYLEGSGGTGKTTLARFLAEE----------RDEHGQHAPSTNKTR-LD----LGS

GY-DGQRTIVINEFDA---SCGI----SYRELFQILEPDAV-TQ-LSSRFKDAHIIN-D---LTIMTNSDDF

IEWAD--AWFP---------------KK-KEYHQLMRRIPFFVKLL--TKNGKTIAELYHYKATKDQGDKCK

EAYEKVKT-------------------YNLGKT---LDEEHLRKQAKKLLSDIR------------------

SLHQKKIKKATTSKS------------------STDQSTKRSSNSFMRNEQ---------------------

-----------------------

>pCRESS5|WP_024399566.1

M--AKKDLSEV---------------------------------KTRRYIAQVSYAYQ----KNADPD----

------------LTEEAFREKIIRLVKMLCTHDE----DKFYIIFHDKDLDEN-------------GE--L-

KSLHAHIYFEFK--NSRFYSSLFKTLE-----ISREKNLQ--IV--------------KDKARVCRYLTHRN

----------------EKDINEG--KYQYEISEII-SSKN-----SD----YRLDICGKSKRADKRKSEQ--

EE----IDFCL-EISYQISECGL-LLNPAKEMLF--EEFD--KLNAQQLWN--RYKKQFEINRQEYIDKEF-

---IRMSKGNR-NHIGLYIEGSGGTGKTTLARFLAEE----------RDEHGQHAPSTNKTR-LD----LGS

GY-DGQRTIVINEFDA---SCGI----SFRELFQILEPDAV-TQ-LSSRFKDAHIIN-D---LTIMTNSDDF

INWTD--SWFP---------------KK-KEYHQLMRRIPFFIKLL--TKNSNTVAELYHYKAAKDIGDKCK

EAYEKVKT-------------------YNLGKT---LDEEHLRKQAKKLLSDIR------------------

SLHQKKIKKATTSIS------------------STDQSTKRSSNSFVRNEQ---------------------

-----------------------

>pCRESS5|WP_024389873.1

M--AKKDLSEV---------------------------------KARRYIAQVSYAYQ----KNADPD----

------------LTEEAFREKIIRLVKALCTHDD----DKFYIIFHDKDIDQN-------------GE--L-

KSLHAHIYFEFK--NSRFYSSLFKTLE-----ISRDKNLQ--VV--------------KDKAKVCRYLTHRN

----------------EKDINEG--KYQYEISEII-SSKN-----ND----YRLDICGKSKRADKRKSEQ--

EE----IDFCL-EISYQISECGL-LLNPAKEMLF--EEFD--RLNAQQLWN--RYKKQFEINRQEYIDKEF-

---IRMSKGNR-NHIGLYLEGSGGTGKTTLARFLAEE----------RDEHGQHAPSTNKTR-LD----LGS

GY-DGQRTIVINEFDA---SCGI----SYRELFQILEPDAV-TQ-LSSRFKDAHIIN-D---LTIMTNSDDF

IEWAD--AWFP---------------KK-KEYHQLMRRIPFFVKLL--TKNGKTIAELYHYKATKDKGDKCK

EAYEKVKT-------------------YNLGKT---LDEEHLRKQAKKLLSDIR------------------

SLHQKKIKKATTSKSGSIISVVGKTTVEIMEPF---------------------------------------

-----------------------

>pCRESS1|CUO57637.1

--------------------------------------MANDT-VSRSWFAVLP--YPEKI-YKG-SP----

-------------------EEILEQMKQQWI-GDN--PLKKGHW--AYCI----------------S---KE

GMPHVHMVLEGS--VSMRFSAVRKCY--------GKAHLEPTR---------------GSRKMVLQYIHKR-

-----------------------G-KFA---EK-------GERVVC--FVSYGNI----EGNK----T----

KTLINQNAILD-RIEELI-EDGK-TPNE----IMGED---------IRLR---KEETLVRK---AFFAKRYR

E-----TPPYR-KVTVVWHLGESGSGKTFSYTKLCES----------RGE--EE-IYFCAEY---ANGGAFD

LY-SGEKILFLDEIRS---TS-L----PYETLLTLIGPYRT-Q--IHCRYANAFALW-E---EVHICSILAP

EDLYK--GMVKSEER-----------ER-DCIQQLLRRINKYVYHY--KEGN---KYK--------------

--------TVEV-------------------EAKRYTSYEELKYSVKK-GNEFS------------------

---------------------------------------------YI------------------NET----

------NTPFREE--------KE

>pCRESS1|CUO23215.1

---------------------------------------MADT-VSRSWFAVFP--NPEQHGYEG-SP----

-------------------EDIIEKLKQEWI-GSN--PLRKGWW--GYCI----------------S---EK

GLPHVHMVLEDT--GSCRFTKVKKAY--------PTAHLEPTK---------------GNKKQVLAYIKKE-

-----------------------P-PFD---EK-------GEQVLV--FTSYGNI----EGNK----R----

FAVSNTNDTLA-TIEMLI-EEGM-TPNQ----IMAED---------IRLR---REENLIRK---CYFAKRYK

E-----TPPIR-TVKVVWHCGDSGCGKSYSYIDLCEK----------YGD--DL-VYFFSDY---ANGGGFD

GY-CGEPYLFMDELKQ---DS-L----PFELLLTITQGYRS-Q--IHCRYSNCITLW-N---EVHITSIFSP

EDIYA--GVVSRENQ-----------GK-DTIKQLLRRITKYVYHY--KVGD---EYK--------------

--------AYEL-------------------AGDQYIDFDDLKRRATG-TE-FM------------------

---------------------------------------------QI------------------DDE----

------DIPF------------D

>pCRESS1|CDF01935.1

--------------------------------------M---E-NSRSWFCVFN--NPAEHGYTG-EP----

-------------------QEVCERLKEEWV-NGS--DTRTGAW--AFCK----------------S---KS

GLLHVHMVLEDT--KSMRFTAIKSSYC-------QGMHFEPTK---------------GNKKQADDYINKR-

-----------------------G-KFE---DK-------EEEILY--ICYHGEI----KGKQ---------

----GKRTDLD-CISDLI-TDGL-KPSE----ILEEN---------PRYY---TKENIIKK---MYFRKRYA

E-----TEFTR-DVKVFWHYGSSGSGKSYSRKQVVEK----------YGE--EE-IYYLTTF---GS-GAFD

NY-EGQKVLWIDDYR----GE-F----RFQELLRYLDVYKA-E--LPARYNNVKALW-N---EVHITSVLTP

QLCYS--EACR-DN-------------L-DRIEQLLRRITCLVYHYKVDNDY---LTI--------------

--------NF--------------------------SPYETLCNMQNRVFSLKK------------------

---------------------------------------------QI------------------ADY----

----------ETII-------ND

>pCRESS1|WP_003102166.1

--------------------------------MTK--EFDSQF-KASSFCCTLN--NIDKTNKTY-SD----

-------------------EEMVEHLIYLWVDGKE--ETRSAAA--NYEI----------------G---DS

GNHHSHLILEAK--NQARFSAIKKLY--------PGIHVELTR---------------GTREEVIAYLNKS-

-----------------------G-KHK---EK-------AHTTVV-AMINHGVI----EANQ----Q----

----GKRSDLD-TIQELL-EAGL-TPES----IMRQN---------LSYR---KFSKMIKE---HYYQMQVE

N-----APLVK-DIKVYWHLGSSGTGKSFTQILLKQD----------HGK--DN-VYVLSDY---GT-GGLD

NY-TGEPILFMDEFK----GD-I----DYQAFLKLLDVYPN-Q--VHARYSNVYALW-D---AVHISSIFTP

NQLYE--MLVPEERR-----------KN-DPIKQLFRRIHFIVYHFKTIDEQ----FK--------------

--------TLTM-------------------TMEEYHKLTNLKINFEKLCK---------------------

-------------------------------------------------QYHQND----------KNTNEYN

Y--------------------KC

>pCRESS1|WP_029176105.1

--------------------------------MTK--ELDSQF-KASSFCCTLN--HIDKPNKSY-SE----

-------------------EDMVNHLIYLWVNTQE--DKRSAAA--NYEI----------------G---DN

GVHHSHLILEAK--NQTRFSAIKKLY--------PAIHVELTR---------------GSREQVIAYLNKS-

-----------------------G-KHE---EK-------SHTTVV-PMINHGII----EANQ----Q----

----GKRTDLE-TIQELL-ETGL-SPEQ----IMRQN---------LSYR---KFSKMIKE---HYYQIQVE

N-----APLTK-DMKVYWHMGGPGSGKSFEQVQLKKT----------FGT--DS-VYVLSDY---GT-GGLD

NY-TGEPILFMDEFK----GD-I----DYQTFLKLLDVYPN-Q--VHARYSNIYALW-D---TVHISSIFTP

YQIYT--MLVPDEKR-----------KY-DPIQQLYRRIHYIVYHSKTIDNE----YK--------------

--------SLTM-------------------TMNDYLTLVKNKLDFDNLIQ---------------------

-------------------------------------------------NYITED----------DASSDYT

YTPNQKTTADKTSTDQSE---SS

>pCRESS1|WP_000032131.1

--------------------------------MSK--ELNSQY-KASSFCCTLN--NIDKPNKNY-SP----

-------------------EEMVEHLIYLWVDGKE--ETRSAAA--NYEI----------------G---DN

GNHHSHLILEAK--NQTRFSAIKKLY--------PTIHVELTR---------------GTREQVIAYLNKT-

-----------------------G-KHE---EK-------AHTTVV-PMMNHGII----EANQ----Q----

----GKRKDLD-IIQELL-EEGL-SPEE----IMRQN---------LSYR---KFSKMIKE---HFYQLQVA

N-----APLVK-KMKVYWHLGGSGTGKSYMQVRLKEI----------FGI--ED-VYVLSDY---GT-GGLD

NY-MGESILFMDEFK----GD-I----DYQAFLKILDVYPN-Q--VHARYSNVYALW-D---KVHISSIFSP

YQIYK--MLVSPDKQ-----------KN-DPITQLYRRIHFIVYHVKINDNE----YK--------------

--------EITF-------------------TMEQYLNLMEQKQCFEDIAQKLI------------------

---------------------------------------------SKGINTVTDDVLSEIKKEIADSSS---

--------------DQTK---SN

>pCRESS1|WP_062004798.1

--------------------------------MTTK-EISSTF-RASSFCCVLN--NVDKNQDTY-TP----

-------------------EEIVDYLMELWI-GRN--ENSVCAV--NYEI----------------G---DK

GTHHCHMVLEDK--QSFRFSTLQTLF--------PTIHAEITR---------------GTKEEVLAYFEKK-

-----------------------G-KHE---EK-------AHTIVV-PMKLYGEL----RANR----Q----

----GQRSDLD-YIQQQL-EEGA-TPEE----IMLDH---------LEYR---AYSKMIRE---HYYQLRLR

D-----TPDHK-ELKVYWHTGDSGSGKSYTQVRLKQE----------FGR--ES-VYVWSDY---QN-GGLD

GY-QGEGILFMEEYK----GE-M----NYAEFLKVTDRYPH-Q--MHARYSNVFALW-E---EIHITSIFSP

KQVYN--IMVPEEKR-----------TA-DSVDQMMRRINKVIYHFKVTNEEAKILYK--------------

--------QLIF-------------------SVADYNSHSKEQI--ERFAYQFD------------------

---------------------------------------------RSNPDVLLYD----------FEKDAFH

STMNPIGNKGKNSLKSSL-KSEK

>pCRESS1|WP_003030931.1

--------------------------------MTNS-NISSDF-RANSFCCVLN--NVDKTNEPL-SP----

-------------------EEIVDFLMERWI-ARN--ENVVCAI--NYEI----------------G---DN

GVHHCHMVLEDK--KAFRFSALQKLY--------PTIHAEITR---------------GTKEQIIAYLEKS-

-----------------------G-EHE---EK-------AHTIVV-PMKVHGEL----RARN----Q----

----GHRSDFD-YIQKQI-ENGA-TPEE----IMMGN---------LEYR---KYSKMIRE---HFFQHRLA

Q-----TPDIK-DMKVYWHVGESGSGKSHTQVNLKKE----------FGR--DN-VYIWTDF---DN-GGLD

LY-CAEPILFMDEFK----G--M----SYKEFLKVTDVYPV-Q--LHARYTNTIALW-N---EIHITSIFTP

KEAYG--LMVPESQQ-----------EI-DSYKQLQRRLTNVIYHFKIREDDGNFKYK--------------

--------TITF-------------------TPEDFERHKQEQI--EKFAYLFD------------------

---------------------------------------------KFNPNVLNYD----------FTKDAFH

LSMNPINNKKKKATTLKLTKANK

>pCRESS1|WP_047207334.1

--------------------------------MTKANNISSDY-RANSFCCVLN--NVDKTDMPL-SP----

-------------------EEIVDFLMERWI-ARN--ENVVCAV--NYEI----------------G---EN

GTHHCHMILEDK--KAFRFSALQKLY--------PTIHAEITR---------------GSKEEVIAYLEKL-

-----------------------G-KHE---EK-------AHTIVV-PMKVHGEL----RAGN----Q----

----GFRSDLH-FIQHQI-ENGA-TPEE----IMRGN---------LEFR---KYSKMIRE---HFFQHRLS

Q-----TPDTK-ELKVVWHVGDSGSGKSHTQVQLKQI----------YGR--EN-IYVWTDH---DN-GGLD

LY-CAEPILFLDEFK----G--M----AYKEFLKVTDVYPA-Q--LHARYTNTLALW-N---EIHIASIFTP

KHAYN--LMVPEGQQ-----------EI-DPYEQLQRRLTKVIYHFKTEEIDGSFQYK--------------

--------SIIY-------------------SPEDFDNHTLQQI--EKHAYLFD------------------

---------------------------------------------KWNREKLVYN----------FEQDAFH

HSMTPINNQKNKATTQKPTKASK

>pCRESS1|WP_029690610.1

--------------------------------MTNY-NVSSDF-RANSFCCVLN--NVDKTHAPL-SH----

-------------------EEIVDFLMERWI-ARN--ENVVCAV--NYEI----------------G---DN

GVHHCHMILEDK--QAFRFSALQKLY--------PTIHAEITR---------------GSKEQVIAYLEKS-

-----------------------G-EHE---EK-------AHTIVV-PMKIHGEL----RSGN----Q----

----GFRSDLD-YIQKQL-DNGA-TPEE----VMMQN---------LGFR---KYSKMIKE---HFFQKKIK

E-----TPDVK-DIKVVWHWGESGSGKSFTQTTLKET----------CGR--DQ-VYVWNDH---EK-GGLD

FY-NAEPVLFMDEFK----G--M----PYKDFLIVTDVYPT-Q--LHSRYTNTFALW-M---EIHIASIYTP

KHAYN--LMVPEGRR-----------EI-DSYQQLKRRLSEIVYHFKVTSEDNENVYK--------------

--------TISF-------------------TPDDFDKYSKEKI--EQFAYLFD------------------

---------------------------------------------KWNPEVLHYD----------FSKDAFH

HSMNPIDNKKKKTTTPKPTKASK

>pCRESS1|WP_029694263.1

--------------------------------M------RSTY-RAHSFCCVLN--NVDKLGYPF-SP----

-------------------KRMIEILIDFWM-EDS--EDRSCAV--NYEI----------------G---ED

GVHHCHMILEGK--QAIRFSALQKLY--------PTIHAELTR---------------GTKEEVFAYLNKT-

-----------------------G-KHE---EK-------AHTIVV-PMMIHGEI----RASK----Q----

----GKRTDLE-IIEQLL-EEGQ-SPEE----IMCRN---------IGYR---RFSKLIKE---HYYQMRLR

M-----SPRFK-EMDVIYHVGASRSGKSYQQIRLMDK----------YGV--GN-VYVWSDY---QN-GGLD

NY-MGEKILFMEEFK----GE-L----SYQEFLRVTDSYVQ-Q--FHARFTNIYALW-E---KVHISSIFPP

KELYK--LMVSNSLR-----------AT-DTMEQMMLRISKVVYHFKVKN-QGKVQYK--------------

--------SISF-------------------SIEDFNRHTIEQI--EKFAYQFD------------------

---------------------------------------------KANPNVLNYN----------FETDAFL

YTMNPIKHLKKKAITPTDQRKDQ

>pCRESS1|WP_053982727.1

--------------------------MKIVTCGVTMGKFSKSI-KGRAWIGTVQIANMEKVEYE--NP----

-------------------EQLAEFISETWS-ASG--KRRVAGV--AVCV----------------S---AK

GLYHAHVVLYGN--LTT-LGNVAKIL--------FDSHIEPQL---------------GGKKELKSYLLKE-

-----------------------P-PYD---EK-------GEQVLH--TKGLDNI----QDTK---------

----GKRSDLE-DIEELL-EQGF-TPRE----IM-EN---------FPYR---KYEKMIKS---AFIDKRIQ

E-----TPLLK-EKKCIWIVGESGTGKSYYYYQLCQE----------HGV--EN-IYFATDF---EN-GGLD

FY-IEQPILFMDEFK----GD-M----RFAQLLVMLDKFRA-Q--VHCRYSNCFCLW-S---TVVITSVFPP

DEVYA--GMVDDAKK-----------DR-DKIDQLIRRLDVIEYRY--KQDG---EYR--------------

--------TFSI-------------------PAKEYVDYDDLKKRA--------------------------

------------------------------------------------------------------------

---------------------EK

>pCRESS1|WP_026669310.1

M------------------------RSDELAQLEKNKSSSYDK-QSKVFLLTLN--NPQK--YGY-TH----

-------------------EFIIDTIH-KFKH------VKYWCI--CDEI----------------G---KS

GNYHSHLYILLG--KKKRWSSVKRAF--------PHSYIVKVM---------------GSPQECRAYIRKE-

-----------------------GERHK---EK-------KDTNLPETFYEEGTI----PTFK---------

-LSNDRVEMLI-QIEDMI-NQGM-RPEQ----IMEQS---------VVFR---QFETIIRK---SFFAKRLK

E-----TPPLR-QIKIVWHLGASGSGKSFSYTQLCGQ----------YGE--DE-VFFASDY---SNCALFD

GY-EGQRVIFLDEVKT---DS-F----KYGLLLQIFQGYKG-Q--IHSRYNNVYSLW-T---EIHATSIFCP

DELYD--EMVPLSSR-----------SV-DSKTQLLRRITDYCYHW--KDDE---GYH--------------

--------VFQI-------------------PAAEYKSYKDLKERAEGKTNEFK------------------

---------------------------------------------NTDESDDIPFVD---------------

-----------------------

>pCRESS1|WP_026524352.1

M------------------------RSDELAQLEKNKSSSYDK-QSKVFFLTLN--NPQK--HGY-TH----

-------------------DFIIDTIHTKFRH------FTYWCM--CDEI----------------G---ES

GNYHTHIYILLG--KKKRWSSVQRAF--------LHSHIEKVM---------------GSPQECRAYIRKE-

-----------------------GERHK---EK-------KETNLSETFYEEGTI----PTFK---------

-LSNDRVEMLI-QIEDMI-NQGM-RPEQ----IMQQS---------VVFR---QFETIIRK---SFFAKRLK

E-----TPPLR-QIKIVWHLGASGSGKSFSYTQLCEQ----------YGE--DE-VFFASDY---SNCALFD

GY-EAQKVVFLDEVKT---DS-F----KYGYLLQILQGYKT-Q--IHARYCNIQSLW-T---EIHATSIYAP

DEIYD--EMVAVPNR-----------TI-DSKTQLLRRITDYCYHW--KDEE---GYH--------------

--------SFQI-------------------PSSEYKSYKDLKERAEGKADDFK------------------

---------------------------------------------DADESDDVPFKD---------------

-----------------------

>pCRESS1|CVH76026.1

M--------------------------------------------ARSWFVTSN--NPHIRGDGTDTD----

-------------------DDACEAFNNRMRTPD----RSGAVV--AFER----------------GE--Q-

GTLHLHGLLCSK--SDMGKSTLIEKF--------PQTDFRETR---------------GSVDDCLDYLHKRG

-------------------------RHADKAETSL----------MQEPYQWGDYL----------------

----GGGKTFE-RIDAML-DQGM-TPND----IFALG---------TKYA---YYGQEIQRRYNALMADKA-

--------RSRDALRCVYHTGDSGSGKSFT-YKLLEQ----------QG---RS-VYYTADY---DH--PFD

SY-NGEDVLFLDELRS---YS-F----ETPQLLSIMESYRH-E--VPARYSNRLAVY-S---EVHLSSIFPP

EKIVP---------------------PN-EPLKQLLRRIDEVVYHA--TAYGRYVTVS--------------

------------------------------VQGAEYQNVKQLERLAIERLAEVE------------------

------------------------------------------------------------------------

-----------------------

>pCRESS2|SCH60086.1

M----------------------------------------PK-QSCNWCFTIN--NPSKFD----TD----

-------------------EKVINFIMQYE-------EVNYYVF--QRER----------------GH--NE

NTEHIQGFIQFK--NRKRGTTLQNMF--------PPQHGEFAN---------------GTAQQASDYCKKS-

-----------------------------------------DTRI-GDVQEWGEL----RVTK----G----

----GKQLTNE-DILQRI-KEGA-DDIR----ILEEF---------PQLW---NQIDRLQKVRDLYVFDKWR

N-------VFR-DVQVTYICGQSGTGKTRS---VMEQ----------YGY--DK-VYRITDY---KH--PFD

SY-HGQDVIVFEEFR----NS-L----PIDNMLNYLDGYPL-E--LPARYMNRIACF-T---KVYIISNWNF

EEQYT--AIQH-K-------------YY-ETWNAFVRRIDKIVTY---KDG---------------------

---------------------------FEEIDLKSYKLKYNLDKDENLQP----------------------

------------------------------------------------------------------------

-----------------------

>pCRESS2|WP_036328238.1

M------------------------------------------NRSRNWLLTIN--YKED--TPT-NN----

-------------------DELLDYIKDIK-------SLTYTAF--QLEQ----------------GE--K-

GTKHHQIYISFE--HAKSFETIKKYF--------PKAHIEAMK---------------GTPEQASEYCTKP-

-----------------------------------------DTRL-LEPIIYGEL----PIK----------

----GKRTDLE-DIYKMI-ASGF-SDMQ----IRETY---------PSQYI--RYNHKFKEIRQEILEEQFN

T-------LFR-KIDVVYLVDLPGTGKTRY---IMEK----------YGY--KN-VFRVSNY---KN--PFD

TY-KGEDVIVFEEFR----SK-L----PIENMLNYLDGYPT-R--LPARYGDKVACY-T---KVYIVSNWEY

TEQYK--NIRE-L-------------YP-TTMQALDRRINFVGNL---QEIKAYDKEQEEIKNLF-------

------------------------------------------------------------------------

------------------------------------------------------------------------

-----------------------

>pCRESS2|WP_044942941.1

M--------------------------------------GNNS-QSRKWALVIN--NPLE--AGL-DH----

-------------------STIKEILQR---F-----SPAYHCM--ADET----------------A---ST

GTYHTHLFFYAP--SPVRFSTIKNRF--------PTAHIEKAY---------------GSVQDNRAYIRKD-

-----------------------G-PWK-DTEK-------AETSVPGTFEEWGEI----PPEQ----A----

----EKHPEMF-RLVQNI-RDGM-TTTE----IIDDN---------PAMA---FRVRDIDLLRQVLTAEKYA

V-------ENR-PLEVSYLYGASGAGKTRS---IYET----------HDP--RS-IYRVTNY---RAKISFE

GY-HGQEVLVFEEFS----GQ-I----PIEDMLNYLDIYPL-S--LPARYNDKTACY-T---KVYITTNLPL

EKQYR--DEQW-D-------------RP-ETWRAFLRRIHTVVEY---LPDG---STV--------------

------------------------------------------------------------------------

---------------------------------------------IHKKGGFPYDQK---------------

-----------------------

>pCRESS2|WP_021629801.1

M--------------------------------------GSNS-QSRKWALVIN--NPSE--AGL-DH----

-------------------SAIKEILQR---F-----SPAYYCM--ADET----------------A---ST

GTYHTHLFFYAP--SPVRFATIKNRF--------PVAHIEKAY---------------GTVQENRAYIRKE-

-----------------------G-RWA-DTDK-------AETSVPGTFEEWGEA----PPER----A----

----EKHPEMF-RLVQNI-RDGM-TTTE----IIDDN---------PAMA---FRVRDIDLLRQTLTAEKYA

V-------ENR-PLEVSYLYGASGAGKTRS---IYEA----------HDP--RS-IYRVTNY---RAKISFD

GY-HGQEVLVFEEFS----GQ-V----PIEDMLNYLDIYPL-S--LPARYNDKTACY-T---TVYITSNLPL

EKQYR--GEQW-D-------------RP-ETWRAFLRRIHNIIEF---LPDG---TTV--------------

------------------------------------------------------------------------

---------------------------------------------QKKKGGWPCDQKR--------------

-----------------------

>pCRESS2|CCZ45692.1

M--------------------------------------GYNA-QARKWLMVIN--NPAE--AGL-GH----

-------------------AAITEIVLK---F-----HPTYFCM--ADEI----------------A---TT

GTFHTHIFFCTR--SPVRFSTIKKRF--------PTAHIERAY---------------GTPRENKEYISKT-

-----------------------G-VWA-DTDK-------VETSVPGTFAEWGEL----PADS----E----

----DKAPEMF-QLMQEL-RSGK-STME----VLEEH---------PNLA---FRIRDIELLRQTILAEKYS

A-------ENR-KLEVTYLYGASGVGKTWG---IFEQ----------HDP--WE-ICRITNY---RGRISFD

GY-NGQDVLVFEEFN----SQ-V----PIEDMLNYLDIYPL-H--LPARYNDRVACY-T---KVYLTSNLPL

EKQYR--AEQW-D-------------RP-ETWRAFLRRIHNVIEY---LPDG---STV--------------

------------------------------------------------------------------------

---------------------------------------------QHKKGGFPCDTK---------------

-----------------------

>pCRESS2|CBL15233.1

M--------------G-----------------------SSNP-QSRKWLLTIN--NPDD--YEL-DH----

-------------------NSVKNTLHL---F-----SPDYFCL--VDEI----------------A---TT

GTKHMHIFIYSK--SPIRFSTLKNRF--------PVAHIDKAN---------------GSVMENRDYLRKE-

-----------------------G-KWQ-GSDK-------EQTNLIDTFEEVGNV----PKPV----D----

----ENSPDMS-ALIEEI-ENGL-DTYE----IIKLH---------PKYA---FRIKEIDTLRQTVLSNMFR

E-------KKR-QVTVYYIYGKSGTGKTRG---IYQK----------HRA--PD-ICRITAY---RRTINFD

SY-HGQSVLVFEEFV----SQ-I----PIEDMLNYLDIYPL-M--LPARFNDKVACY-D---TVYITSNISL

GEQYS--EVQH-Y-------------KP-ETWKAFLRRINFLVEY---TDVN---TYT--------------

--------VT---------------------EINKVKE----------------------------------

------------------------------------------------------------------------

VKADD------------------

>pCRESS2|WP_021882760.1

M-----------------------------------RIISRDI-QCRKWLLTIN--NPDE--HKF-SE----

-------------------SEIENILNT---F-----KFRYACL--SREI----------------G---EN

GTPHIHLFIYAK--SRIRFSTIKKRF--------PTAHIDKAY---------------GSVVDNIAYITKT-

-----------------------G-KWE-NTDK-------AETSVEGSFKEYGEA----PSAL----E----

----EHSPELS-QILDDI-VSGM-STSE----IITEY---------PQHI---FRVNAIDTVRQTFLADKYR

E-------RMR-SVCVTYIHGASGVGKTRG---IYKH----------FPA--ES-ICRITSY---SKNVKFD

SY-CGQDVLVFEEFA----SQ-I----PIEEMLNYLDVYPL-M--LPARYTDKVACY-T---KVIITSNLPL

NKQYV--NEQI-E-------------KQ-KTYNAFLRRINYVIEY---DKKG---NVK--------------

--------KK---------------------TLHKEVSLDEKDT----------------------------

------------------------------------------------------------------------

-----------------------

>pCRESS2|WP_051600858.1

M-----ELKDITTSKS-----------------------K-DP-QSRGWMLTIN--FNGA--SPL-TE----

-------------------DALIELIQM---N-----TFDYACF--AFEK----------------G---EQ

GTLHVHIYIHSE--NPRRFSTMKMTF--------PRAHIEKAL---------------GSPAEIRDYIKKD-

-----------------------G-KWK-DSEK-------AETSIPGTFRELGKI----PTPG----A----

----SRSNNKNQKLLEDI-TAGK-STAE----IIKDS---------PDYI---FKINCINTAREELLNTDHQ

N-------SFR-DVTVYYVYGATGTGKTYS---IYQC----------YDA--KD-ICRITDY---PDNVRFD

AY-MGQKVLVLEEFR----SE-I----PISSMLNYLDRYPL-K--LPARYYDRQACF-T---TVIITSNIPL

EEQYL--AIQD-V-------------QP-ETWRALIRRINYVRHY---NRNG---VID--------------

--------DY---------------------TISADPSGKIIYHPLLRPG--VS------------------

---------------------------------------------DAPEAFNRRRNEPFPHVYDSPTLVTTN

VTEEDILTSYSLFDYIEEEGIGN

>pCRESS2|WP_013978550.1

M---------------------------------------SDV-QSRKWFFTFN--NPAE--HGD-TH----

-------------------ESVAGRFSEL--------SLAYWCL--GDEI----------------GA--ET

GTYHTHGFIYSP--SPIRFTRLKKLF--------PFAHIEKAN---------------GTCKENRDYVAKE-

-----------------------N-KFS-ND----------PTKVEGSFEEHGEL----PKER----E----

----PKEDRKE-RLYAMV-EAGL-TTEE----IIELD---------KSFI---FQANTIDGLIQRRLASRHK

G-------VNR-SVAVIYIWGETGTGKTRS---IQER----------HSA--DG-ICRITSY---RNGVSFD

AY-KGEPVLVFEEFN----SQ-I----AIEEMLNYLDVYPL-M--LPARYSDKVACF-T---QVYITSNIPL

EKQYP--EVQR-T-------------RP-ATWRAFLRRIGKIAHH---LPDG--------------------

-------------------------------SIEETLMERG-------------------------------

------------------------------------------------------------------------

-----------------------

>pCRESS2|WP_009301216.1

M---------------------------------------GNP-QSRKWNLTIN--NPKD--YGL-TR----

-------------------EIITDRMNSLF--------PNYYCI--SDEV----------------S---QS

GTPHTHIFIYRK--SPIRFSTIRSKF--------PTCHCEKAH---------------GSVLENKEYVSKT-

-----------------------G-KWQ-GTEK-------EETKVEGSFFEWGEI----PNEK----Q----

----EKNPLNY-EVIKDL-EDGK-VIGE----IVSDR---------PELI---FKVKQIEALKEALLIKN-A

N-------KFR-SLSVIYCFGESGVGKTRM---VYEC----------HEP--ID-ICRITNY---RKKMSYD

VY-HGEKVLLLDNFQ----NS-L----CIDDLIALLDIFPM-Y--LPARFYDRYSVY-E---FVYLLSVLPL

ENQYK--DIQK-H-------------YP-LKWNALINKISKIIEI---KETG---EVI--------------

--------EH---------------------KKERYIIHNEKD-----------------------------

------------------------------------------------------------------------

-----------------------

>pCRESS2|WP_038350939.1

M--------------------------------------K-DT-RTRKWQITIN--NPLE--KGF-SH----

-------------------DYIKAQLEK-F-K-----SCVYWCM--SDEV----------------G---EQ

ETFHTHIYMACS--NAVRFSTVKNRF--------EGAHFEMAQ---------------GTSQQNRDYVFKE-

-----------------------G-KWA-HTSK-------EETNIKESHEEWGEL----PIER----Q----

----GQRNDMA-DLYDMI-KQGY-SDFE----IMEES---------PAFL---MNIDKIEKARQIITSEKYK

N-------TFR-ELEVTYIYGKTGSGKTRS---VMEK----------YGY--PN-VFRITDY---QH--PFD

NY-HSQDVVIFEEFR----SS-L----KIQDMLNYLDGYPL-E--LPCRYANKYACY-T---KVYIITNIPF

EEQYD--NIQH-V-------------SP-ETFNAFKRRIHKILHY---KDKN--KIEK--------------

---------------------------------EDYFDQMSLL-----------------------------

------------------------------------------------------------------------

-----------------------

>pCRESS2|KJZ87129.1

M-----K--------------------------------N-DS-RSRKWQITIN--NPVD--KGY-TH----

-------------------ENLKNILNNGF-K-----NIVYWCM--SDEI----------------G---EN

KTYHTHIFLACS--GAVRFSTVKKRF--------EGAHFEMAN---------------GTSKQNREYVFKE-

-----------------------G-KWE-TSNK-------KETNIADTHEEYGDC----PIER----Q----

----GQRNDLI-DLYDSI-KAGL-SNYD----IIEDN---------PNFM---FDVDRIERARQMVRDEQYK

N-------TFR-ELEVTYIYGKTGCGKTRG---VMEQ----------YGY--SN-VFRITDY---NN--PFD

SY-KGQDVIIFEEFR----SS-L----KIQDMLNYLDGYPL-E--LPCRYANKIACF-T---KVYIITNIAL

EHQYD--SIQK-E-------------FN-ETWNAFLRRIHKIKYF---NGND---VDT--------------

--------FN---------------------SVNEYFDR---LTLVND----NA------------------

---------------------------------------------------EIF-----------KQES---

-------I---------------

>pCRESS2|WP_023977019.1

M-----K--------------------------------N-DS-SSRKWQLTIN--NPVD--KGF-TH----

-------------------EVLKEKLKE-F-K-----NLIYWCM--SDEI----------------G---EN

KTYHTHVFIACS--GAVRFSTMQNRF--------KGAHFEMAR---------------GTCKQNREYVFKE-

-----------------------G-KWQ-GDKK-------QDTNLPDTHEEYGDC----PVER----Q----

----GQRNDLI-DLYDMI-KGGM-TNFD----IIEDN---------PSYM---LEIDRIEKVRQTVRDEQFK

N-------TFR-ELEVTYIFGSTGSGKTRG---VMEY----------FGY--SN-VFRVTDY---DH--PFD

SY-KGQDVVVFEEFR----DS-L----KISDMLNLLDGYPL-E--LPCRYANKIACY-T---KVYIITNLDL

NDQFK--GVQV-K-------------HP-ETWKAFLRRIHKVIHY---TKNS---VDE--------------

--------Y----------------------KLQEYLDRD--LVPVDD----GT------------------

---------------------------------------------------SPF-----------EQEKI--

-------I---------------

>pCRESS2|WP_018597672.1

M--------------------------------------G-DL-QSRKWQLTIN--NAVD--KGF-TH----

-------------------EHIIELANT-F-K-----SLTYMCL--SDEV----------------GG--ET

QTHHTHVYLAFR--SAVRFTSLQKKF--------MGAHFEVAK---------------GTSQQNRDYVFKE-

-----------------------G-KWS-KDVK-------GETNLRDTHYEQGEM----PVER----Q----

----GKRNDLE-DLYDMI-KQGM-DNYQ----ILEEC---------PQYM---LNVDKIERCRQIVREEKYK

N-------TWR-DLHVTYIYGETGSGKTRT---VMEK----------YGY--EN-VYRCTDY---DH--PFD

SY-KGQDVIAFEEFR----SS-L----RVRDMLNYLDGYPV-E--LPCRYANKVACF-T---QIYIITNIPL

NEQYT--DLQR-A-------------QM-ETWQAFLRRIHEVHVY---VGGQ---VYK--------------

--------G----------------------SCEDYING---FLPPV-----GK------------------

---------------------------------------------------TPF-----------DEK----

-----------------------

>pCRESS2|CUP05665.1

M-----GEN-----------V------------------K-DS-QSRKWQLTIN--NPVE--KGF-TH----

-------------------EKLNSILAS-M-A-----SVIYYCM--ADEI----------------G---EN

QTYHTHVFLCGR--SGIRFSTLKKQF--------EGAHFEMAK---------------GTAEQNMQYVSKT-

-----------------------G-KWL-NDRK-------RETCVDGTFEEYGEM----PIER----Q----

----GKRNDLD-DLYGMI-KDGL-TNYE----IMEQM---------PEAL---LNLDKIEMTRQTIIQEKYK

N-------QWR-DVQVEYIYGDTGSGKTRS---IMEQ----------YGY--SN-VFRVTDY---LH--PFD

GY-KNQDVVIFEEFR----SS-I----RFTEMLTLIEGYPV-E--LPCRYANKYACY-T---KVYIITNVPL

SKQYP--AVQL-D-------------ES-VSWLAFLRRIHKVKKY---TYEG---IQE--------------

--------S----------------------HIEITKDG---FRTVLD----GE------------------

--------------------------------------------------FIPFREVVNGSKNVVERLA---

-------L---------------

>pCRESS2|SCH17786.1

M-----E--------------------------------K-DS-QSRKWQITIN--NPAD--KGF-TH----

-------------------ERIRQELES-M-K-----SVIYWCM--ADEV----------------G---EN

GTYHTHLYLQGK--GAVRFSTIKKHF--------EGAHFEMAK---------------GTAMQNREYVSKT-

-----------------------G-KWE-NDKK-------HETCVDGTFEEWGEM----PIER----Q----

----GARNDIA-DLHAMI-KQGL-SNYD----IMEQV---------PEAM---LMLDKIEQARQTIVQESYK

T-------KWR-NMSCYYIYGDTGTGKTRS---IMEQ----------YGY--EH-VFRVTDY---SH--PFD

NY-RGQDVVIFEEFR----SS-F----RVSDMLNYLDGYPL-E--LPCRYANKYACY-T---KVYIISNIPL

SEQYR--NQPQ------------------ETFEAFLRRLNGVLHY---TGHG---IEK--------------

--------S----------------------RIELIGSG---FRLITN----GE------------------

--------------------------------------------------ITPF-----------DKMEL--

-----------------------

>pCRESS2|CDC44519.1

M-----A--------------------------------K-DT-QSRKWQLTIN--NPVE--HGF-TH----

-------------------DKIKDLIYL-M-K-----PVIYWCM--ADEI----------------G---EE

GTYHTHIFICGR--SGIRFSTLKRAF--------ESAHIEMAK---------------GTSLQNKEYVSKT-

-----------------------G-RWE-KDKK-------HETCVEGTFEEYGDM----PIER----Q----

----GKRNDLD-DLYSMI-KEGM-SDYD----ILEQG---------SDYM---LNLDMISKTRQILVQEKFK

N-------TFR-KLDIVYIWGETGTGKTRS---VMEG----------HGY--GN-VYRATDY---LH--PFD

NY-AGQDVILFDEFR----SS-L----ALTDMLKYLDGYPL-E--LPCRYANRYACY-T---KVYLISNIPL

SEQYP--NVKR-D-------------EY-GSWLAFLRRIQTVKYF---SQGT---VKV--------------

--------S----------------------NVSVDSNG---FREIPA----GE------------------

---------------------------------------------ELMLFDSPN-----------DVGT---

-------D---------------

>pCRESS2|BAK32345.1

M-----N--------------------------------Q-DS-RSRKWQITIN--NPHD--KSI-TH----

-------------------EAILDTIEE-M-N-----GILYYCL--SDEI----------------G---ES

GTYHTHIYLVSN--NAIRFSTMKNKF--------PTAHFEIAR---------------GTSTDNRDYIFKL-

-----------------------G-KWK-EHKK-------AETNLTDTHKEWGEI----PLER----Q----

----GARNDLA-DLYDMI-KQGM-DNFD----ILEST---------PENM---MKLDKIERTRQVIKESEYR

N-------TFR-KLTVTYISGDTETGKTRY---VMES----------NGY--EN-VYRITDY---KH--PFD

GY-QGQPVICFEEFR----SS-L----PIASMLNYLDGYPL-E--LPCRYSNKIACY-T---SVYIVSNISL

EKQYE--YEQE-N-------------ET-ETWNAFLRRIHHEIVH---SKNG---QA---------------

------------------------------------------------------------------------

-----------------------------------------------------------------EKEG---

-------N---------------

>pCRESS2|WP_044928503.1

M-----DTEV-----------------------------R-QI-QSRKWQLTIN--NPLE--KGY-TH----

-------------------DAIKKNVCT-L-K-----SLRYFCM--SDEV----------------G-----

KTHHTHVYILFQ--SPVRFSTIKRLF--------PEAHIEKAY---------------GSSIQNRDYIFKE-

-----------------------GEKWS-KDKK-------RETNLPDTHEEWGEM----PTER----Q----

----GERNDLT-ALYELI-SEGK-SNYD----ILEEQ---------PEFI---TQIERMDKVRQIIQEESYK

D-------IFR-NLEVDYLYGDTGSGKTRS---IMEK----------FGY--AN-VFRVTNY---KH--PFD

QY-KGQDVIMFEEFQ----SS-I----HINQMLIYLDGYPV-T--LPCRYSDKVACY-T---KAYILSNIDL

KEQYP--DIQT-Y-------------SP-ETWKAFLRRIHKVQVF---KNGK---VET--------------

--------Y----------------------SLFDYLNE---FQMLTA------------------------

----------------------------------------------EQMTFCPFKEG---------------

-----------------------

>pCRESS2|WP_037404274.1

M-----GE-------------------------------S-DT-RSRKWQLTIN--NPIE--HNL-KH----

-------------------NTIKEYLSN-L-D-----NLIYWCM--CDEI----------------GG--QE

KTYHTHVYIHLK--NAIRFSTLKAMF--------TTAHIEKAN---------------GTAIQNKEYILKQ-

-----------------------G-KYA-DTEK-------ESTNLKDTFEEMGII----PEES----Q----

----GKRTDLI-EIFEMA-KDGI-KTVD----ILSEY---------PSAL---LYIDKIEKVRTEIQREKFK

N-------TFR-KLDVTYIYGKTGAGKTRY---VMDS----------FGY--EN-VYRVTDY---KN--MFD

SY-SLQDVIIFEEFR----SS-V----HVKDMLSYLDGYPI-D--LPCRYSNKVACF-T---KVFIISNIDL

LAQYT--DIQR-K-------------EV-ETYRAFLRRINKIIYF---DYSQ---KEE--------------

--------FE---------------------SVNSYLESRGVKI----------------------------

------------------------------------------------------------------------

-----------------------

>pCRESS2|CDE72464.1

M--------------------------------------K-NT-QSRKWQLTIN--NPLE--KGC-SH----

-------------------EEIKKAMES-F-S-----TCEYWCM--CDEI----------------GL--EE

HTPHTHVFIYTT--NGTMFNTVKNIF--------PIAHIEHCK---------------GTYAQNRDYIRKE-

-----------------------G-KYK-NDEK-------TKTNLTETFEEFGTM----PTER----Q----

----GGRNDLA-DLLDMI-TAGL-DTST----ILEQY---------PNYM---LQLDKIERTRQIMLESKYK

N-------VFR-EMEVQYIFGAPGSGKTRG---VMEK----------YGF--DK-VYRVTDY---KN--PFD

GY-HGQDVIIFEEFR----SS-L----KIGDMLNYLDGYPL-S--LPCRFSNKQACY-T---KVFIISNIPL

TAQYN--DLQS-E-------------QT-ATWKAFLRRLNGVVEY---LANG---NVN--------------

--------NYK--------------------TIEEYYSWQGM------------------------------

------------------------------------------KGCKLIQEDLPF------------------

-----------------------

>pCRESS2|CDB27189.1

M--------------------------------------CYTTNKARKYQLTFN--NPVQ--HGF-TH----

-------------------AVIKTTLAS-F-P-----GIQYWCM--CDEI----------------G---EQ

GTPHTHLYLYSP--NAILFSTLQQRF--------MGAHMEAAK---------------GSHRENRDYIRKE-

-----------------------G-RWL-DDTK-------HETNLPQTFEESGPL----PAEQ----N----

----KRESISS-EILELV-QSGA-SNAE----ILLQY---------PSAM---NRLQHIETARQTLLEERYR

N-------QWR-NLEVTYLWGPTGVGKTRS---VMEL----------YSY--EN-VYHVTNY---DH--PFD

DY-RGQNVILFDEFR----SS-L----PVADMLKYLDGYPL-M--LPCRYSNKVACY-T---KVFLISNIPL

SAQYP--NVQL-S-------------EP-ETYRAFCRRINQGLEM---QAD---------------------

------------------------------------------------------------------------

------------------------------------------------TGKEPF------------------

-----------------------

>pCRESS2|WP_053167095.1

M-----IDSNKQTDESVEKVIA---------------PKK-DS-KSRKYLLTIN--NPTK--DMI-TH----

-------------------ESIQEILATKF-K-----SLLYACM--SDEV----------------A-----

SQHHKHVFLAFS--SPVRFSQIKKYF--------PSAHIDKSR---------------GTAENNRHYVFKE-

-----------------------G-KWA-DTNK-------AETNLKETHWEWGEI----PNER----S----

----GNRTDLA-ELYELI-QDGF-SNAE----IIEQN---------PDNI---LFLQHIDRTRKAILEEKFK

S-------NWR-NLDVTYIFGPTGTGKSRH---VMEK----------YGY--EN-VFRITDY---LH--PFD

SY-RQQDVIVFEEFT----SS-L----KIQDMLNYLDGYPL-E--LPSRYSNKQATF-T---KVYILSNSPL

RKQYE--TVQI-E-------------KI-EVWRAFLRRIKKVLLF---DKQG---THK--------------

--------EF---------------------TTDEFLHW---TAPANI----LA------------------

---------------------------------------------------EKF----------GISKLI--

-------K---------------

>pCRESS2|WP_009246639.1

M-----EKTEENIVQN-----------------------N-NI-QRHAFQVTIN--NPLK--HGF-NH----

-------------------LKIKKTLIEKF-A-----TLRYFCM--ADEI----------------G---KQ

GTPHTHIYVCFK--SRVRFSTVQKYF--------PTAHIEKPH---------------ASVQSNIDYICKR-

-----------------------G-KWE-NTDK-------ADTKVEGTFEEWGTV----PLQK---------

----GTRPDME-ELYQMI-DAGY-SNAE----ILAIN---------NDYI---LDIDKLDKVRTMLLIEKYK

G-------KRRINLKVIYISGATGTGKTRG---VLDE----------HGD--EN-VYRINDY---QH--PFD

GY-SCQPVLAFDEFR----SS-L----KLSDMLNYCDIYPI-D--LPARYANRFACY-E---TVYIISNWEL

EQQYK--EVQE-D-------------NP-ESWRAFLRRIHEVRIY---DRDG---KVT--------------

--------NYE--------------------SVEKYLKRKEEFCTLTS------------------------

------------------------------------------------EDDCPFEK----------------

-----------------------

>pCRESS2|CCY69022.1

M-----QQENI----------------------------K-NV-QRNAFQLTIN--NPVE--YGY-TH----

-------------------EKIKETLIMNF-T-----TLKYFCM--ADEI----------------G---GQ

GTYHTHIYVVFS--SRVRWSKVKKNF--------DEAHIEIAK---------------GSAQSNVEYIKKT-

-----------------------G-KWA-ETNK-------AETSVEGTFEEWGEI----PTQR---------

----GKKADME-ELYEMI-KNGY-SNAE----ILAIN---------NDYI---LNIDKLDKVRTMLLTEKYK

N-------ERRLDLKVIYIYGATGTGKTRG---VLDE----------HGN--SN-VYRVSDY---LH--PFD

GY-GTQEVIAFDEFR----SG-I----KISDMLNYCDIYPI-E--LPARYSNKFACY-S---RVYIISNWSL

EMQYS--EVQK-N-------------SP-ESWQAFLRRIHEVHHY---HADG---TLD--------------

--------VYD--------------------SVEKYLHRDEEFHVISD------------------------

--------------------------------------------------------------------EELK

QIKIYRLR---------------

>pCRESS2|WP_052011064.1

M--------------------------------------------------TIN--NPKE--HGF-SH----

-------------------NEIHEILKNNF-K-----TLIYYAL--SDEC----------------G-----

TCYHTHLFLVFS--SRVRVSTIHRNF--------EGAHIEIAR---------------GSISDNINYLKKE-

-----------------------G-KWKENKEK-------QEKRIEGTFEEYGTR----PPDS----Q----

----GKRTDMS-ELYQYI-NDGL-SNAE----ILALN---------QDYI---LNVDKLDKVRNILLTNRFK

E-------EVRLDLQVIYISGATGTGKTRG---VFEK----------DGY--VN-TFRVTDY---AH--PFD

AY-CCQETIVFDEFR----NS-L----KLSEMLNYLDIYPV-D--LPSRYNNKVACY-R---KVYIISNWKL

EQQYS--YEQI-N-------------DR-ESYLAFLRRIHKVVTY---KDNG---EKV--------------

--------QYN--------------------SVEEYLNRGKERE----------------------------

------------------------------------------------------------------------

-----------------------

>pCRESS2|CCX75435.1

M-----HREGEII----------------------------DP-QSYMFQLTIN--APVE--KGY-TH----

-------------------EKIVNIIRSKF-K-----TNIYFCL--ADEQ----------------G-----

ERYHTHIFIVFS--SRVRFSMVKRYF--------PEAHIEKCR---------------GNVSENVSYIKKS-

-----------------------G-KWELDEIK-------QEKKIEGTFEEHGIQ----PSDS----K----

----GKRSDLS-ELYRMI-QDNM-TNAE----ILAVN---------QDYI---MQIDRLDKVRTTILMERFK

E-------TVRLDLEVIYIFGKTGTGKTRR---VLEE----------NGY--IN-VYRVTDY---NH--PFD

SY-TAQQAICFDEFR----SS-L----KLKEMLLYCDIYPI-E--LPSRYSNKFACY-N---KVYIVSNWEL

EKQYS--ELQR-E-------------DK-ESWQAFLRRIHKVIYY---KDIN---EII--------------

--------EYP--------------------SVQAYLERNSEFRTIGD------------------------

------------------------------------------------DEENPFDKG---------------

-----------------------

>pCRESS2|WP_024346025.1

M--------------------------------------AKDN-QSRKYNMTIN--NPAD--GGY-TH----

-------------------EYIQKTLSTLF-K-----SFAYGAL--ADEV----------------G---EQ

GTPHTHVFACFS--SAVRFSMIKKHF--------PTAHIESAK---------------GSIAQNLDYIKKG-

-----------------------G-KWT-GTDK-------ADTSVPGTYKEFGER----PPEN----L----

----GKDKDLE-TLYHMIVDEGL-SNAE----IIRIN---------QDYI---MQIDKLDKIRTTHLQDKFK

G-------ERRLDLEVTYMFGATGTGKSRG---ILDE----------YGD--EN-VYRVTDY---DH--PFD

HY-SCEPVLVFEEFR----SG-L----PLSDMLNYLDIYPI-T--LKARYSNKFACY-T---KIFVVTNWEL

EKQYA--ERQI-T-------------DR-ASWRAFLRRIHKVVQY---VSKG---NKI--------------

--------VYN--------------------SVEEYLNRDNEFAPVGK------------------------

-------------------------------------------------SQTPFGK----------------

-----------------------

>pCRESS2|WP_038278663.1

M--------------------------------------KKDK-QSRKYQLTIN--NPTD--SCF-SH----

-------------------DKIKEYLVTRF-K-----SFEYGAM--CDEI----------------G---EQ

GTPHTHIFICYR--SPVRFSMIKKHF--------PTAHIEAVA---------------GSIEQNIDYLKKE-

-----------------------G-KWA-ETAK-------AETSVPGTFEEWGDR----PPEN----L----

----GKDKDLE-ALYHMVVDEGL-SNAE----IIRLN---------NDYI---MQIDKLDKIRTTYLQDKFK

G-------ERRLDLSVEYTFGSTGAGKSRG---ILDE----------FGD--AN-VYRVTDY---DH--PFD

HY-SCEPVLVFEEFR----SS-L----PLSDMLNYLDIYPI-T--LKARYANKYACF-S---RIFIVTNWEL

EKQYA--ERQI-T-------------DK-ESFQALLRRIHKVKHY---VSKD---EVI--------------

--------IYD--------------------SVDDYLNRNNKFIPIKK------------------------

-------------------------------------------LSKEEQLEIPFDK----------------

-----------------------

>pCRESS2|WP_007865724.1

M-----TTKKKNI--------------------------NNNR-RSRKYNCVFN--NTDK-HKNC-SH----

-------------------QAIKEKLSN-W-E-----NIIYWCM--CDEI----------------A-----

KTPHTHLFVQFK--NPVYFSSIKKTF--------PSAHIEEAQ---------------GTAEENRAYIRKD-

-----------------------G-KWE-NTEK-------ESTNLKETFEEWGTM----PQTG----Q----

----GRRSDLA-NLYQMI-KDGY-SNVE----ILEIN---------PDNL---LNLQHIDKARLEILSSRYK

A-------ERRMNLLVTYVSGATGYGKSRY---ILDN----------HGD--SN-VYRVTDY---KH--PFD

TY-SGEDVIVFEEFR----SD-L----PIGNMLNYLDVYPL-Q--LPARYNNRQACY-N---FVYIVSNWKL

DDQYH--NIRL-E-------------QK-ETWNALIRRIHKVRIY---TAPG---EWQ--------------

--------EY---------------------DTIDYLHG---FQPVDR------------------------

-------------------------------------------------ADTPFNN----------------

-----------------------

>pCRESS2|WP_013270924.1

M-----TTKKKDT--------------------------NSNR-RSRKYQLTFN--NPEK-HKNC-SH----

-------------------QAIKEQLLN-W-E-----NIIYYCM--CDEI----------------A-----

KSAHTHLFIQFK--NPVYFSSVKKSF--------PTAHIEEAQ---------------GSAEENRAYLRKE-

-----------------------G-KWV-NTEK-------ETTNLKEAFEEWGTM----PQTG----Q----

----GRRSDLA-NLYQMI-KDGY-SNVE----ILEIN---------PDNL---LNLQHIDKARLEILSNRYK

A-------ERRTNLVVTYVSGATGYGKSRN---ILDN----------HGD--SN-VYRITDY---KH--PFD

TY-SGEDVLVFEEFR----SD-L----PIGNMLNYLDIYPL-Q--LPARYNNRQACY-N---FVYIVSNWIL

EDQYH--NIRL-E-------------QP-ETYKALMRRIHKVRVY---ESPG---KWK--------------

--------EC---------------------DTESYLHG---FQYTDQ------------------------

-------------------------------------------------KDTPFSRT---------------

-----------------------

>pCRESS2|WP_066550639.1

M--------------------------------------KKDT-RSRKYQLTWN--NPQD--RGE-TH----

-------------------DKLKEILMKKWGD-----SVVYYCL--SDEI----------------G---ET

GTPHTHMFVCYQ--NAVRFSSIKDSY--------PSAHIEVAK---------------GSPESNRAYIRKD-

-----------------------G-KWA-ETAK-------AETVVEGTFEEYGFI----PKEG----Q----

----GRRNDLN-QLYEQI-KAGY-TNAE----LLEND---------PDNM---LRLSYIDRTRNELLIEKYK

G-------TRRLDLQCIYVFGETECGKTKT---ILDE----------HGD--EN-VCRVTDY---KH--PFD

HY-AMEDVLVFDEFR----SD-L----PIGAMLDYMDIYPL-Q--LPARYNNKTACY-H---YVYLVSNWKL

EDQYH--DEQL-E-------------HK-SSWAAFLRRIKKVREY---TGRD---AYV--------------

--------EY---------------------SKEEYFNR---FQPVKE------------------------

------------------------------------------------QDGNPFLNDK--------------

-----------------------

>pCRESS2|WP_020072285.1

M-----K--------------------------------K-DT-RHRKYLLTIN--NPGA--K-W-SH----

-------------------EKICAVLGK---M-----QLKYWCM--ADEQ----------------GL--QE

QTPHTHVFLVAN--SAIRFSTVKGYF--------PTAHLDPAC---------------GTSEENRAYVQKS-

-----------------------G-KWA-GDEK-------SDTSIPGTFEEGGVL----PTEN----P----

----GQRTDWD-IALAML-EDGH-SAMD----VIRVQ---------THLM---RYRSTLEQIRQELIAEQFR

D-------TFR-ILETTYIYGTTGLGKTRF---VMER----------YGY--EN-ICQITGY---QH--GFD

KY-QSEDVIVFDEFS----SS-L----KIQDMNNFLDGYPL-M--LPCRYANRVACY-T---RAYIISNIPL

EYQYA--NVRL-D-------------TP-AVWNAFIRRIHKVVHF---TGEN---QYD--------------

--------EI---------------------TTKEYFAS---RQKMLDGW--IE------------------

---------------------------------------------IENTGDLPFDTDKCKNPN---------

-------Q---------------

>pCRESS2|WP_051639324.1

M-----IYE------------------------------K-EK-RKRKWLLTIN--NPQD--KGI-TH----

-------------------DSIKQSLQP---Y-----TLNYYAI--VDET----------------G---AH

GTYHYHVYIYFK--NAIHFSSLKKLF--------PTANIQQAM---------------GNSLQNRSYLLKSA

----------------PEHKQPDG-KYE-YKDN-------TGTNHTNTFEEFGEC----PTEI----R----

----GKRNDLE-RMYELI-KEGY-SNSE----IIEAG---------KTAI---LHIEKLNKLRHSYLIDYYK

G-------TRRLNLKVHYISGKTGLGKSRD---ILDE----------YGD--EN-VYRVTDY---QH--PFD

SY-QNENVLVFEEFR----SL-I----RLSDMLNYLDIYPC-V--LPARYSPKIACY-D---TVFIVSNWEF

ESQYY--ELQQ-DPL-----------QI-TTYEAFKRRINYVKIY---TENG---ITK--------------

--------YN---------------------SLNDYLNRNKGFFTITE----GQ------------------

------------------------------------------------EEDIPFK-----------------

-------N---------------

>pCRESS2|EES75484.2

M-----AQK------------------------------N-DI-QRNKYFLTIN--SPEK--FGY-TH----

-------------------EVIYQVASN-F-K-----TFQYVAV--VDEQ----------------G-----

SNFHTHVLLVFK--SRVRWSTVQDKF--------PHAHIEEGK---------------GDINQILQYMRKE-

-----------------------G-KWLLDEKK-------QEQKIEGSFESWGDR----PVDT----K----

----EKVSEFS-ELYDLV-YDEV-PTGE----IIKFN---------PKYI---RYIDKIAPMRIEIMNEKYR

G-------KRRLDLKVIYVFGLSGTGKTRM---ILDR----------HGD--EN-VFRVTDY---FH--PFD

SY-NMQQVLCLEEFR----DS-L----TITQCLNLLDIYTV-E--LPARYANKLGIY-K---TVYMVSNWEI

GKQFK--SVQQ-E-------------HP-ETYHAFRRRFHYLLDF---REKN---VHA--------------

--------WNSRDFDMGFTARVQKLLEIIDGEIDESETFKEIGQIIQKME--KP------------------

---------------------------------------------DTFESAVAKRKEWISKCREKKLDINKD

IFHAPVPNNIRNLFPMNTSANM-

>pCRESS2|WP_051546484.1

M-----TKNN-----------------------------KLPLKTMRRIFLTIE--NPKT--YGY-TP----

-------------------HKLKSMIKK-M-K-----SLIYGCY--CYET----------------G---EC

GTEHVHIYLSLK--NSIRFSTIQNHF--------PSAHIEKSE---------------GNHDECIAYIQKD-

-----------------------G-KWS-NTEK-------ASTNQKKTFWEYGKR----PEVK----R----

----RRKKKMRLSVLDLI-RTGK-SNLE----IVQIY---------PSFI---SKMKALDEIRQEFLKEKYG

H-------TNR-HVECTYIYGDTRTGKTSD---IIKK----------YGA--EN-VYRITSYGLTAH--PFD

GY-RGQDVLVFDEFR----AA-M----PFSNMLTYMEGYPL-E--LPARYGNKVACY-T---KVIVISNDPL

KNQYT--SVDR-S--------------S-QSWMAFLARFMSVQHY---TNNG---ITE--------------

--------YG---------------------SATEYYEQTMNNKALIA------------------------

------------------------------------------------------------------------

-----------------------

>pCRESS3|WP_055838650.1

M------------------------EKLRQEESAAPAKNPKDP-EARRWMLTLP--ESEY------DQ----

-------------------AEIEKRLGK----------YKAVVG--QLEA----------------AP--TT

GYLHWQLYLETK--SALRFSTLRKLF--------PKGHYKPAR---------------DTRIQCVRYCTRE-

-----------------------------------------DKRASGAVINLGQL---DLTLK----Q----

----GKRTDLD-SYSEQIMLEGK-SADE----VIHDD---------PRAV---IYASHLWQLELIRDRETWG

K-------KFR-ELEVHYIHGGTRTGKTSA---LFET----------YGY--EA-IYRVPNW---KN--PFD

GY-RGQDILLLDEYN----TS-V----PMVDLLKLLEGYPL-E--VSARYSDKIAKF-T---TVFIVSNLEL

SEQHQ--TIQH-E-------------HP-KQWAALGARLTSVSEL---VIEGEGDDARGKLRVHKGTPP---

-------------------------------QLDHYLTGKPFFDED--------------------------

------------------------------------------------------------------------

-----------------------

>pCRESS3|NP_613078.1

M------------------------------------EDKRET-GYTDWLLTIR--RELP-DGSERTV----

-------------------DDVVNALQGI---------FDAAIG--QPEK----------------GE---G

GYRHYQIFAQGK---RQRFSTLKKKL--------TDAHVEPRK---------------GSVSEAVGYCSKE-

-----------------------------------------KT--DGDGFQFGQI---DRHEKEDSHQ----

----GERSDLA-RLKARA-EAGE-TVSQ----ILLSEDG-------ELAA---RYLGWLRATCDAAQAAKYR

T-------KVRDDLEVNFLYGETGVGKTSH---VYES----------EGI--GT-VYTVTDY---AH--AFD

KY-EGEGILLLDEFT----GQ-F----PMPLMLKLLDKWPM-Q--LPARYSNRWAAF-S---RIWVVSNLPP

NNLYS--YAPE------------------SQRRAFFRRFAHFYKM---DEAHQLIEEPNPLQPVVSEFDRLN

A-----------------------------------------------------------------------

------------------------------------------------------------------------

-----------------------

>pCRESS3|WP_021975256.1

M----------------------------------------PS-TARDWMLTIS--AEKH------TR----

-------------------QDVEELLDI----------LGAYIF--QQEE----------------GG--KS

DYPHFQAFLQLQ--TPVHMGTLKNKF--------KDAHIEMRK---------------GTVQDCVDYCSKE-

-----------------------------------------ETRV-DGPWRGGEI---NLKDQ----Q----

----GSRSDLA-ELRRQI-MDGA-SVSE----VLLNDDA-------CQAA---RYTRYLSELATARDRVKYG

R-------QLR-DITVHYLWGDPGVGKTKY---IYDN----------NPI--EN-IYRVTDY---RH--PWD

EY-EGQSILVLDEFD----SQ-F----SWDQLLVFLDRYPV-M--LPARYNNHVACF-T---TVWIISNEPL

SKQYP--ERTG------------------EKRNALLRRISTNQRM---LKGGELQAGE--------------

----------------------EMGLLEDDLFKASEQLSKE-------------------------------

------------------------------------------------------------------------

-----------------------

>pCRESS3|KFI81686.1

M-------------------------------------SERDR-QSRDWMLTVP--AEDH------TQ----

-------------------DEVRTLFERI---------STGAVF--QHET----------------GA--TT

GYEHFQCFLQMK--SPMRFSTLKNHL--------TDAHIEPRH---------------GSVEDCVAYCTKP-

-----------------------------------------DTRA-DDPVYVGEI---DMKDR----Q----

----GRRSDLI-AFREQI-LDGV-PVQQ----VLLDDTE-------AKAA---HCTKWLNAFSEACARQEYG

N-------KLR-DVSVHYLYGAPGVGKTRY---VYDR----------YPF--ED-VYRVTDY---AH--PFD

EY-DRHRVLVLDDYD----SQ-L----PWEQLLSYLDRYPV-T--LPARYHNHQACF-D---TVWIISNLPL

TAQYP--DITG------------------ARRLALLRRITDCTHM---LADGTLVKEP--------------

-------------------------------LPGQREEVNR-------------------------------

------------------------------------------------------------------------

-----------------------

>pCRESS3|WP_043170238.1

M-------------------------------DDEEEGKHKDS-QSRDWMLTIR--AEGH------TE----

-------------------DDVKALFEKI---------GVGAVF--QREI----------------GG--KT

EYEHFQCFLQVK--TPMRFSTLKNHL--------TDAHIEPRR---------------KTVEDCVNYCTKE-

-----------------------------------------ETRA-GEPIYVGKI---NMKDK----Q----

----GQRSDLI-GFREQI-LGGM-SVQE----VLLGDTE-------AKAA---HCTRWLGELEAAYVRKEHG

G-------KLR-DLDVHYLYGAPGVGKTRY---VYDK----------YPI--ED-IYRVTNY---KH--PFD

EY-NRHKVLVLDEYD----SQ-L----PWEQLLCYLDRYPV-T--LPARYHNHQACF-T---TVWIISNLPL

SAQYP--DIVG------------------ERRFALIRRLTDCSYM---TPEGELIKEP--------------

-------------------------------LPGRQEGGSP-------------------------------

------------------------------------------------------------------------

-----------------------

>pCRESS3|WP_052825216.1

M-----EEATAKEEDEVKGDKNVEG------------AKGEDK-QSRDWMLTIP--AGRH------TE----

-------------------DDVAELLERI---------CSGAVF--QREK----------------GE--ET

DYEHFQCFLQIP--SPMRWSTLKNHL--------ADVHIEVRE---------------HSVESCVNYCSKD-

-----------------------------------------DTRI-GETRYIGKI---RMQDQ----Q----

----GKRTDLS-DLREKI-LNGA-SVED----VLLEDTE-------SKSA---RYVKWLSELAAARDKKKYG

R-------QMR-NVEVHYLWGAPGVGKTSY---VYER----------YPI--ED-IYRVTDY---QH--PFD

EY-DRQPVLVLDEYD----SQ-F----DWEKLLCYLDRYPL-M--LPARYHNRQACY-T---TVWIISNKSL

DEQYP--LVQG------------------ERRLALTRRLSDVRHM---GEARELIIPD--------------

----------------------GNGHSFKDIPVRSNQERPA-------------------------------

------------------------------------------------------------------------

-----------------------

>pCRESS3|KFI87454.1

M------------------------------------NGNNNP-SATDWMLTVS--AEQN--GREITE----

-------------------ADLVDAFED-----------WSWMG--QREE----------------GG--HT

GYRHYQLFMQAT--SRIRLSTVRARL--------EVNYIEPRR---------------YSVASCVAYVSKK-

-----------------------------------------ETRQ-AGPFIHGDF---DMHED----Q----

----GKRTDLE-ELRDAV-VKGA-SVNE----ILNDELS-------LKAA---RFMPWLEKMVGARQAARFS

Q-------EDR-EVTVHYLWGKPGLGKTRS---VLD-----------GDR--SQ-IFRVTNY---EH--PFD

DY-SGQSTLVLDEFA----GQ-L----PFQLLLNVLDRYPC-K--LPCRFHDTWAGW-T---TVWIISNKPL

ERQYQ--DVEP------------------QVRAALDRRITTNEEF---KSDEEFAAIVARAEAEVNEDLAFL

E-----------------------------------------------------------------------

------------------------------------------------------------------------

-----------------------

>pCRESS3|WP_022856850.1

M-----EHE----------------------------KVK-DA-QSRDWMFTLK--IEND------------

--------------------TLTTLLGMLGSW-----TALEYVF--QHER----------------GS--KS

GYDHFQGFLRCK--SSNRFSTVKNHF--------ITVHIEARK---------------GSPRQAYDYCTKE-

-----------------------------------------DTRI-EGPWQSAHI-MDNLDLA----T----

----GKRNDIE-DARELI-ESGL-TPRK----IMLADSE-------GRFA---HLTTYIETYYQARLSNEYA

T-------KER-DVLPIYLYGETGSGKSRW---VADN----------LGY--PD-VYTVSDY---TH--PYD

GY-TDQKILVFDEFH----SQ-R----PIEEMLRLLDPYPV-E--LPARYHNKQACY-R---LVIVISNFPL

ATQYE--TAEY-P-----------------QRAAFQRRFSNSIDM---AKME--------------------

---------------------------RSHLHLESTQIPEQS-EDEPVVL--SP------------------

---------------------------------------------EMQEIFGLPAQE---------------

-----------------------

>pCRESS3|WP_052119337.1

M-----TDKDRTVTGDG------------------------QK-YSRCWMLRIS--KIEDEWHGTPRH----

-------------------QDICSAFHDAY-G-----SGFAING--QLEK----------------GT--KT

GYLHFQVLLITK--YEKKGQAIIDAI--------AIGGTEKLR---------------KNIYAGVRYTSKD-

-----------------------------------------ATRV-ERYDPFGDADILNGEQT----Q----

----GARNDLN-ELRRAI-VDHM-TVDD----ILRDDLS-------IKSA---RYVSWLDRLQRANSVTPHA

T-------EQR-DVKAHYLYGSPRIGKTRL---IYDN----------ISV--NQ-FYRVTDY---QH--PFD

SY-VGQKVLVLDEYD----SQ-F----PITSINNFLDRYGC-E--LPARYHNSWANW-D---EVWVISNLPI

NSQYS--DDNT-D-----------------KKNAFIARFTDITYM---DKSG---LFY--------------

--------D----------------------AADDDVLSDMLDAERNQRP--WG------------------

---------------------------------------------EGNYITSPWVFGKSRFIENPDLGFL--

-----------------------

>pCRESS3|WP_023022037.1

M------------------------------------RKCSKSTQSRSWMATIS--AEKM------HR----

-------------------EEIEEALEQ-----------YSYIG--QLEK----------------GE--EG

SYRHWQLLIDGNT-SPIRFSTLKNKL--------PTAHLEPRR---------------GPIQQAIEYATKE-

-----------------------------------------ETRVEEPRLEHGTI---RHGDE----R----

----GRRKDVD-IVREAVLEKGL-SVDE----IFLQV---------PEAA---RMTSFVERLVAARERAQN-

-------SAPR-EVEVMWLYGPPGTGKTSL---AVD-----------IGG--DD-FYRVTDY---SH--PFD

SY-AGEKTLILDEFD----GS-M----PLSLVLNILDVWPM-S--LPARYANKAAAY-T---QAVMVSNESP

WGYYL--WEPA------------------SRRQGLARRIDTIIHI---DSYGAHDETRLRDKFFA-------

------------------------------------------------------------------------

------------------------------------------------------------------------

-----------------------

>pCRESS3|AKO38848.1

M-------------------------------ENNRMSGGKNT-RHRRWFLTVP--AEGE--KGV-SR----

-------------------DELEQALEP----------YDAFLG--QLEQ----------------GKGTN-

QYRHWQLILVHP--EPVRFSTLRRKL--------PTAHLEPVR----------------DLRASLAYVQKE-

-----------------------------------------DTRVDEPPLVKGKI---SPGPG----Q----

----GHRSDLD-TLRSRI-LDGQETADE----LILSD---------TGAW---RHSRLVGDLVSARDRSRQE

G-------KLR-DVQVRVVFGDTGTGKTSA---ALSGL---------QAL--GS-VCRVTHW-GGAG--TFD

GY-DGQDSLVLDEFA----GQ-P----PLTELLTWLDVFPV-T--LNARYRARQAAF-V---RVVLCSNAPP

WTWYP--WAPK------------------AQRAALARRLHLVEEW---SGS---------------------

---------------------------WDNVTVTEIPSSEVMRRMTANPPGKLG------------------

------------------------------------------------------------------------

-----------------------

>pCRESS3|WP_016667133.1

M-------------------------------------------------LTIK--ADDF------TR----

-------------------EEVEEKLKK-----------YDYLG--QLEC----------------GE--ES

GYLHWQVLIENK--TAIKFETLKNKF--------RTAHCEVAK----------------NLFACRKYVSKE-

-----------------------------------------ETRVGEELLRGGKFV----------------

----TKGDAVS-DLRHAILFEDK-SADE----LILSD---------GRYR---PYVAYAKELERIRDASKFG

L-------SPRTSVNVRYLYGAPGVGKTWG---VYDE----------FGY--PD-VYSPGTY---IH--PWD

EY-QSQRVLLLDEFD----GQ-I----EFELLLKVLDIYPL-T--LPCRYQNKYAAW-D---TVIMVSNNPL

ESLPYRDRVSA------------------SKWAALLRRINVYEEM---VSRGVCESRLQKTSL---------

------------------------------------------------------------------------

------------------------------------------------------------------------

-----------------------

>pCRESS3|WP_002529618.1

M----------------------------------------TTDRCRDWMLTLP--EEYY------SR----

-------------------DIVEDKLRS-----------YDYIG--QLES----------------GK--ES

GYRHFQIYVENK--NAIKFETLRSKF--------PRGHYEPRR---------------ESKSQCLKYCTKS-

-----------------------------------------DTRVGHSLLAGGKFVLKDRLPR---------

----TKRDISA-EISEKMLKENV-PAST----LIQD----------PRYA---QFLKYIEALETIRLKNLG-

-------LEDRDALEVHYLYGPSRVGKTYK---ILHG----------LNYDLTD-IYRVSNF---KY--PWD

NY-EGQSVLLLDEFA----GQ-I----SFEFLLQVLDKYQL-E--LNARYRNKWACW-T---QVWIVSNLPM

ESLPYYRRVSP------------------EQWRALCMRFTSYQRM---ESDRSLVNVPFPSAS---------

------------------------------------------------------------------------

------------------------------------------------------------------------

-----------------------

>pCRESS3|WP_036342632.1

M------------------------------------------RATRDWMLTIP--ASKF------DK----

-------------------KEVENRLKK-----------YQYIG--QLEK----------------GKTDT-

EYLHWQVFVHGTIGAAIRFDTLRNKF--------DQVHLEPRC---------------GTILDCINYVTKL-

-----------------------------------------DTAVAKKDDDYEYITIRIRQDGQDNWDDLRK

NARTKSRISNE-EIYQEI-ISGK-TAGQ----IINDH---------PELG---MQFLKIKALENGIKEEQFR

G----LQTEDRENIEVNYLWGPPGAGKSWH---VLNE----------AGYDRRD-IYRKNGY---QH--VWD

NY-QGQRVLILEDFT----GQ-I----GIEELLQVTDIYAT-E--LDARYSNHYAGW-E---VVWIISNLRL

DDLLK--KYPK------------------ELRPALVSRITNVYLM---EDREMIN-----------------

------------------------------------------------------------------------

------------------------------------------------------------------------

-----------------------

>pCRESS3|WP_025221073.1

M-----SGPKEPETGAEKEKKALGE--------------KVET-RSRDWCVTQH--LEAS--DSI-TL----

-------------------EDYVDHVREFF---------DAGAL--QMEL----------------GEHTDT

GIPHIQAFFQGK---PKRFSTVVRFL--------EHPYVDKRR---------------GTVKQAVSYAQKD-

-----------------------------------------DTRVGTSPVVFGEI---NMRES----Q----

----GKREDLL-DLRKMV-DAGL-TVDE----ILLEDVE-------GKAA---RYVGWLDRLVAARDAKKMS

E-------RLQRDLHCTFIWGKTGVGKTRY---ALEQG---------RSL--GK-VARIVDY---RH--PWD

MV-DDTDVIVCDEYN----GQ-L----DLTEFLTILEGYGA-SP-MRARYRNRWPNY-S---QVYVLSNTAL

NEMYS--YEPS-E-----------------RRRALFRRFDRIQYM---FVKQ---FGK--------------

--------NRGERRLVDI-------------DQKKYGAAYLVEPPISEIL--AQ------------------

---------------------------------------------VDPLRAEDLFLPSSADGEDIVKEGNHI

ITA--------------------

>pCRESS2|WP_066546553.1

M----------------------------------------STARTRRMQITCN--NPEE--KGL-SS----

-------------------DRIKEIMQR---W-----KTEYYCF--CFET----------------G---EQ

GTDHFHLYVKFV--NPQSTRVLSKAF--------GNAHVEIIRS--------------SSSSQNRDYIRKE-

-----------------------G-AYL-DSEK-------KETNHIETFYESTEC----PDES-EEIQ----

----GRRTDIE-RMISLV-QDGA-SNVE----IVQAV---------PSMA---LKISALDQYRQAFYEEQGK

Q--------YR-DVTTIYIYGRTRTGKTSS---VYAN----------HDP--SE-ICSVMDY---KGG-VWD

QY-DTTRVLLLDEYR----SS-L----KISELLAICDGQPH-T--LRCRYSNRVCLH-D---TVYIISNISL

LQQHK--EIQR-D-------------EP-ESWEALLARIKIVRHY---YDVG---KYR--------------

--------DY---------------------SVQEYLQVEADGMIENPFV----------------------

---------------------------------------------FCDSSDTPFVTEKKEVQNGKHSIIF--

-----------------------

>pCRESS2|WP_013271491.1

M----------------------------------------ST-RSRRMQITCN--NPLD--KGF-TS----

-------------------EKIKEIMQR---W-----KTEYYCF--CYET----------------G---ES

GTHHFHLYTKFC--HAQITDTISKTF--------GNAHIEVIRN--------------SSSIDNRDYIRKE-

-----------------------G-AYL-DSEK-------KTTNHIETFYESCEC----PIDG-KENQ----

----GHRNDID-LMISLV-QDGA-SNME----VVQAV---------SSMA---LRIPAIEQYRQAYWEEKGR

G--------YR-HMDIWYIYGKTRTGKTSY---VYQS----------HHS--SE-IYSVVDY---KGG-IWD

KY-DTTRVLLLDEYR----SA-L----PFSLILALCDGQPL-T--LNCRYANRVCLH-E---TVYIVSNISL

LEQYP--NIQR-E-------------EP-ESWNAFLARINHVRHY---YEIG---QYK--------------

--------DY---------------------TVEEYLTTEQNPHLKEFE-----------------------

---------------------------------------------SCNPDSTPFKNTSPIPIQLELPPL---

-----------------------

>pCRESS3|WP_033495900.1

M-----AGISEQDGESLSLVREIPD-------DEKKKKLNRHK-RSRAWMVTIA--SK----YVCGDW----

-------------------SVILDELNDMTA------YGWSYMG--QAEE----------------GTGKRR

GMRHGHFIV-YT--PRMRMGTFADHF--------PQAHIDPVE---------------RTPKVVERYVTKY-

-----------------------------------------DTRLGGPWESEGFD----PAGQ---------

----GKRTDLK-TRDDAV-KAGA-IPDM----LMRDD---------PKLI---AYDRYLDRAYEIAMKEKGR

--------KMR-DVHTLYVYGETGVGKSLWAW-LYGK----------RHN--AD-VYRLTDY---AH--PWD

NY-RGESVVIVEDFD----GR-M----RLDDVLRWTDRYPV-E--LTARYANKHALY-T---TVIFTSNRPM

SGWYG--YDEF-E----------------PKQGPINRRVSTVCASL--GSDGDTISFR--------------

--------HVAGPEWPETLADEADGHG----EKPETAPAKAPQPVADDEAYPVD------------------

---------------------------------------------MSSPTALPFVVGADGIPIDLE------

-----------------------

>PpulchraPlasmids|AAF36424.1

M--VNLKK-----------------------------KKPSFRLNARLFFLTYPCQSG--------------

----------------LTKELILRELRKIV--------SDIHTVVVSKER----------------GESGD-

GYDHFHVLLEAK--TKKNYKD-PRCFDILGVH--G--KYE----------------TVRNRKRSLKYICKEG

DVVSENV---------DVTALLSAFKKPFDR--------------FVFKCKYL---DENPSRLIAEAKNSDE

YN----QDYF--EIYVDYLS----SPKRYEKFIAEY----------------RKDSTPPKRLWIHKLKMSEM

DWAEKIVEEGV-VEKSLYIHGKPGIGKTNMARLMFN----------------DK-IAIIKHLDKL-----KE

ASVDQSVAIGFDDVNLKSGK--Y----TREDCINIVDGEVG-SQ-IDVKYGMVVLEPYV---PKVFISNLLP

EMVYK------------------------GYDKAVERRLRIIYLE---SAEGLFQAIYKH------------

---------------------------ERDVPLESKDDYVEMHESTFGFLVEWV------------------

------------------------------------------------------------------------

-----------------------

>PpulchraPlasmids|AAF36423.1

M--VKTKK-----------------------------GNGSFRLNAKIMFLTYPCQSG--------------

----------------LTKEVILQELKKKI--------FDIHSVVMSKER----------------GESGD-

GYDHFHVLLETK--TKKNYKD-PRCFDILGVH--G--KYE----------------STRNKKRAMKYICKEG

NVLCEGI---------ELGALLSTFKKPFDR--------------FMFRCRYL---GENPSELISRSRNSSS

YV----QDDF--EIYNDYLI----SPKKYKQYILES----------------VKVSSPPKRLWIHKLKMDEM

IWAKTIVSNDH-ISKSLYIHGAPGVGKTNMARLMFD----------------DK-MFIVKHVDKL-----KE

ADVESSVAIGFDDVNLNTDK--Y----TREDCINIVDPEIG-SQ-INVKNSMISLEPYV---PKVFISNFLP

ERVYK------------------------GYDEAVERRLKVICLE---SAEGLVQAIYKR------------

---------------------------EKDVPLESKSDYVEMHESTFKYLVDWV------------------

------------------------------------------------------------------------

-----------------------

>PpulchraPlasmids|AAF36422.1

M--LPSNR-----------------------------SKKSFRIAAKTFFLTYPCQSG--------------

----------------LTKELILLELGKII--------TDIHSVVMSKER----------------GESGD-

GYDHFHVLLEAK--SRKNYKS-PRCFDILGVH--G--KYE----------------TVKSKKKALKYICKEG

DVLSEGV---------DVEALLSTFKKPFDR--------------FMFRCRYL---SENPSVLISRSRNSSS

YV----QDDF--EIYNDYLI----SPKKYKQYILES----------------VKVSTPPKRLWIHKLKMDEM

IWAKTIVSNDH-ISKSLYIHGASGVGKTNMARLMFD----------------DK-MFIVKHVDKL-----KE

ADVESSVAIGFDDVNLNTDK--Y----TREDCINIVDPEVG-SQ-INVKNSMVSLESYV---PKVFISNFLP

ERVYK------------------------GYDEAVERRLKVIYLE---SAEGLVQAIYKH------------

---------------------------EKDVPLESKSDYVEMHESTFKYLVDWV------------------

------------------------------------------------------------------------

-----------------------

>pCRESS9|YP_007008175.1

M--K----------------------------------KNKFKLNTKDIFLTYS------------------

-------------KCPLGKDKIHNHIKQLIISKK----KEISYIISNTE-----------------NHQDH-

KEIHTHVLFQLT--KRTTFHG-ERFFDIEGFH--P--KIE----------------TARDIEKSIDYIKKDG

DFIEEGTPRYEKYVRQNQK--EERKQLIYDEIDRLK---------DEYY--ND---EKLTLQQVKKTLDDFI

KNLDRDFYYEQIDLIEKILKKKFIKQAE----LALIDKLQNHIPCTGYDLNTFKVDKSTQEIGIAITNQLK-

--------VEK-RPLSIVIEGPSRLGKTEFIISYLNYHR-------------IHFNYIRGSFDFS-----KE

NYNDSFKVDVYDDISMNYIK--------SSGLLKNIIGGQR-GFIVDVKYPPKRLLSGNKLSIFLVNPDISF

ESYCEEDE------------------KHAGETYKYIKSNCIFINV---DNK---------------------

-----------------------------------------LYDDPQDKSLKDI------------------

------------------------------------------------------------------------

-----------------------

>pCRESS9|WP_015083745.1

M--K----------------------------------KNKFRLQTKDIFLTYS------------------

-------------KCPLGKDKIHNYIKELLVSKK----KEISYIISNTE-----------------NHQDH-

KEIHTHVLFQLN--KIIQIEN-QRFFDIEGFH--P--KIE----------------NARDIEKSIDYIKKDG

DFIEEGTPRHKKYVRQNQK--EERKQLIYDEIFRLK---------NEYY--HD---EKLTLNQVKKTLDEFI

INLDRDFYSEQIDLIEKILKKKFIKQAE----LALIDKLQNHIPSTGYDLNTFKVDKSTQEIGIAITNQLK-

--------VEK-RPLSIVIEGPSRLGKTEFIISYLNYHR-------------IHFNYIRGSFDFS-----KE

NYNDSFKVDVYDDISMNYIK--------SSGLLKNIIGGQR-GFIVDVKYSPKRLLSGNKLSIFLVNPDISF

ESYCEEDE------------------KHAGETYKYIKSNCIFINV---DNK---------------------

-----------------------------------------LYDDPQDKSLKDI------------------

------------------------------------------------------------------------

-----------------------

>pCRESS9|YP_001708784.1

M--K----------------------------------KTKFKIKTKDIFLTYS------------------

-------------KCPLGKDKIHNHIKQLMASKK----KEIQYLITNQE-----------------NHKDH-

KEIHSHVLFQLT--KSATFNG-ERFFDIEGFH--P--EIE----------------VARDIEKSISYIKKDG

DFIEEGTPRHKKYVRQNQK--EERKQLIYDEIFRLK---------NEYY--HN---EDLTLQQVKKTLDEFI

INLDRDFYLEQIDLIEKILKKKFIKKDE----LALLDKLQNHIPSTGYDLNTFKFNKSTQEIENAIVEQLK-

--------VDK-RPLSIVIEGASRLGKTEFIISYLNNQK-------------VHYNYIRGSFDFS-----KE

NYNDNFKVDVYDDISINYIK--------SAGLLKNIIGGQG-GFIVDVKYSPKRLLSGNKLSIFLVNPDISF

ESWCEEDE------------------KYAGETHKYIKTNCIFIYV---PDK---------------------

-----------------------------------------LY-----------------------------

------------------------------------------------------------------------

-----------------------

>pCRESS9|YP_001965310.1

M--KKENKMKQE-------------------------TKTNFRLQTKDIFLTYS------------------

-------------KCPLGKEKIHNHIKQLMESKN----QKIAYIISNTE-----------------NHQDH-

KEIHTHVLFQLN--KRCNLTS-QRFFDLDGYH--P--KIE----------------NTRDVEKAIEYIKKDG

DFVEEGTPNRKKYVRQNQK--EERKQLIYDEIFRLR---------DEYY--ND---DKLTINQVKKTLDDFI

INLDRDFYLEQIEFIKRVLKEAFAKKEE-----KLADDEELITP--DYSFESFKTNSTTNEIIAAIQSQLS-

--V---QSLSK-RPKSIVIEGPSRIGKTEFLLSYLTHIN-------------LHYNYIRGEFDFS-----KE

SHKNAYKINIFDDISIPQIN--------KEGLFKNIIGGQR-GFRFNVKYAPKRFIAGKKINIFLINPDISF

KGYCEWSY------------------KKGHKFHEYIEDNCIFIYV---SDK---------------------

-----------------------------------------LY-----------------------------

------------------------------------------------------------------------

-----------------------

>pCRESS9|YP_001708790.1

M--E----------------------------------KNKFKINAKDIFLTYS------------------

-------------KCPLGKDKIHNHIKELMASKK----KEIQYLITNQE-----------------NHKDH-

KEIHSHVLFQLT--KRTTFNG-ERFFDIEGFH--P--EIE----------------TARDIEKSINYIKKDG

DFIEEGTPRHKKYVRQNQK--EERKQLIYDEIDRLK---------EQYY--ND---DNLTLSQVKRTLDKFI

INLDRDFYYEQISFIKTILNEVFTKKKE-----ELEDEEEFFKP--DYSFDSFKSNSKTNEIIDAINQQLS-

--V---QSLGE-RPKSIVIEGFSRLGKTEFILSYLTHIN-------------LHYNYTRGDFDFS-----KQ

SHKNAYKVNIFDDISIPKIR--------KEGLFKDIIGGQK-GFKYNVKYAPKRTIAGKKLSIFLVNPDISF

EDYCEWSE------------------NNGHKFHEYIRDNCIFIYV---PDK---------------------

-----------------------------------------LY-----------------------------

------------------------------------------------------------------------

-----------------------

>pCRESS9|YP_007008179.1

M--K----------------------------------KTKFKLNTKDIFLTYS------------------

-------------KCPLGKDKIHNHIKQLLVSKK----KEISYIISNTE-----------------NHQDH-

KEIHTHVLFQLT--KPTTFNG-ERYFDIEGFH--P--KIE----------------NARDIEKSIDYIKKDG

DFIEEGTPRYKKYVRQNQK--EERKQLIYDEIDRLT---------DEYY--HD---EKLTFSKVKKTLDNFI

KNLDRDFYYEKIDFVEQVLNKKFTKKKE-----ELEDEEEFFKP--DYSFDSFKSNSKTNEIIAAIETQLS-

--V---QSLGE-RPKSIVIEGFSRLGKTEFILSYLTHKN-------------LHYNYTRGDFDFS-----KQ

SHKNAYKINIFDDISIPRIR--------KEGLFKQIIGGQK-GFKHNVKYAPKRTIAGKKLSIFLVNPDISF

ENYCEWSE------------------NNGHKFHEYIKDNCIFIYV---PEK---------------------

-----------------------------------------LY-----------------------------

------------------------------------------------------------------------

-----------------------

>pCRESS9|WP_013747472.1

M--K----------------------------------KNKFRLQTKDIFLTYS------------------

-------------KCPLGKDKIHNYIKELMISKK----QEISYIISNTE-----------------NHQDH-

KEIHTHVLFQLT--KQLSIRN-QRFFDIEGFH--P--KIE----------------NARDIEKSIDYIKKDG

DFIEEGTPRHKKYVRQNQK--EERKQLIYDEIMRLK---------DEYY--ND---DSLTLNKVRKSLDDFI

LNIDRDFYLEQSELIDRILNKKFTKKKE-----ELEDEEEFFKP--DYSFDSFKSNSKTNEIIAAIQTQLS-

--V---QSLGE-RPKSIVIEGFSRLGKTEFILSYLTHKN-------------LHYNYTRGDFDFS-----KQ

SHKNAYKVNIFDDISIPQIR--------KEGLFKQIIGGQK-GFKYNVKYAPKRTIAGKKLSIFLVNPDISF

ENYCEWSE------------------DNGHKFHEYIKDNCIFIYV---PDK---------------------

-----------------------------------------LY-----------------------------

------------------------------------------------------------------------

-----------------------

>pCRESS9|WP_011412958.1

M--K----------------------------------KTKFQKNAKDIFLTYS------------------

-------------KCPLGKDKIHNYIKEIMISKK----QEISYIISNTE-----------------NHQDH-

KEIHTHVLFQLT--KRFNIQS-DRFFDIEGFH--P--RIE----------------TARNIEKSISYIKKDG

DFIEEGTPRHKKYVRQNQK--EERKQLIYDEIDRLI---------EKYY--HD---DKLTLNQVKKSLNDFI

KILDRDFYYEKIDFVEQVLNKKFTKKKE-----ELEDEEEFFKP--DYSFDSFKSNSKTNEIIEAINQQLS-

--V---QSLGK-RPKSIVIEGPSRLGKTEFILSYLTHIN-------------LHYNYTRGEFDFS-----KQ

NHKNAYKINIFDDISLTRIR--------KEGLIKDIIGGQK-GFSYNVKYAPKRTIAGKKLSIFLVNPDISF

ENYCEWSR------------------NKGYKYHEYLEDNCIFIYV---PDK---------------------

-----------------------------------------LYH----------------------------

------------------------------------------------------------------------

-----------------------

>pCRESS9|YP_006961991.1

M--K----------------------------------KTKFRLNTKDIFLTYS------------------

-------------KCNLGKDKIHNHIKELMASKK----QEISYIISNTE-----------------NHQDH-

KEIHTHVLFQLT--KRLNIKS-ERFFDIEGFH--P--KIE----------------NARDIEKSIDYIKKDG

DFIEEGTPRHKKYVRQNQK--EERKQLIYDEIFRLK---------DEYY--ND---DNLTLTQVKKTLDEFI

INLDRDFYLEQIDLIEKILKKKFIKKDE-----ELDEEEEFSTA--AYSFDSFKTNSETNKIIDAIDSQLN-

--IINSKKTNK-RIKSIVIEGPSRLGKTEFILSYLTHKN-------------LQYNYIRGEFNFS-----KE

SHKNAYKVSIFDDISIPEIR--------RAGLLKNIIGGQR-GFEYNVKYSPKRTVLMRRLSIFLVNPDISF

ESYCEWSR------------------DNGHKFHEYLEDNCIFIYV---NNK---------------------

-----------------------------------------LYDDPQDKALHDN------------------

------------------------------------------------------------------------

-----------------------

>pCRESS9|WP_011412950.1

M--K----------------------------------KTKFRLNTKDFFMTYS------------------

-------------QCDLGKEKIYHHIKQLMASKN----QEINYLSVCLE-----------------NHADN-

NGVHSHVFLQLK--KRYQVVN-NRFFDIDGKH--P--EIE----------------RARTVQGSVDYVKKDG

DFIEEGTPKDKKYITKNEN--DKLKKIIYDEIDRLK---------NEYY--YD---DNLTFNQVKKSLDDFI

LKTDRDFYYEKIDLIKKILNERFTKKKE-----ELEDEEEYSKP--DYSFNSFKTNSKTNEIIAAIQTQLS-

--V---QSLGK-RPKSIVIEGPSRLGKTEFILSYLTHIN-------------LHYNYTRGEFNFS-----KE

NHKNAYKINIFDDISLTKIR--------KEGLIKDIIGGQK-GFTVDVKYAPKRNISGKKLNIFLVNPDISF

ENYCEWSR------------------NDGHKFHEYIEDNCIFIYI---PDK---------------------

-----------------------------------------LYEEINE------------------------

------------------------------------------------------------------------

-----------------------

>pCRESS9|CBX25033.1

M--K----------------------------------KTKFRLRTKDFFMTYS------------------

-------------QCDLGKEKIFNHLKQLMASKN----QEINYLSVCLE-----------------NHADN-

NGVHSHVFLQLK--KYLDIQS-GRFFDIDGKH--P--EIE----------------RARTVQGSVDYVKKDG

DFIEEGTPKDKKYITKNEN--DKLKQIIYDEIDRLK---------NEYY--HD---DKLTLNQIKKSLNDFI

LKTDRDFYYEKIDLIKKILNERFTKKKE-----ELEDEEKFSKP--DYSFNSFKSNSKTNEIIAAIQTQLS-

--V---QSLGK-RPKSIVIEGPSRLGKTEFILSYLTHIN-------------LHYNYTRGEFNFS-----KQ

SHKNAYKINIFDDISLTKIR--------KEGLIKDIIGGQK-GFTVDVKYAPKRNISGKKLSIFLVNPDISF

ENYCEWSR------------------NNGHKFHEYIEDNCIFIYI---PDK---------------------

-----------------------------------------LYEEINE------------------------

------------------------------------------------------------------------

-----------------------

>pCRESS9|YP_001965305.1

M--KKENKMKKE-------------------------TKTNFRLRTRDIFLTYS------------------

-------------KCPLGKEKIHNHLKQLLASKK----KEIKYIISNNE-----------------NHQDH-

KEIHTHVFIQLK--KQIEITN-QRFFDIEGYH--P--KIE----------------TARDVEKSVSYIKKDK

DFIDEGEYIQKKYVRQNQK--EERKQLIYDYICEKR---------IEYYNDND---NSLNINKIRKEIDDFI

LKIDRDFYYEQIELIDRILNRRFIRKKE-----ELADTIKLF-GKSHYQFNSFKTNSETNEIIDAINSQLL-

--------SEH-RPKSIVIEGESRMGKTQFILSYLTHKN-------------LQYNYIKGEFDFS-----KK

TYKDYYKIDVYDDFGVPEIN--------SQGLQKNIIGGQE-CFTCNVKYAPKRQLSGNKLSIFLVNPDNSF

KGYCEWSR------------------NNGHKFHKYIEENCIFIYV---SDK---------------------

-----------------------------------------LF-----------------------------

------------------------------------------------------------------------

-----------------------

>pCRESS9|WP_017193171.1

M--KKI------NTKIKE-------------------TKNKFQFNSKDIFLTYS------------------

-------------KCSLGKNVIHNHIKNLMNEKK----KNIIYIISNTE-----------------NHADH-

KEIHTHVLLQLE--KRSNIKD-ARFFDIEGFH--P--RIE----------------NAQHIEKSIDYIKKDG

DFIEEGTPRIKKYVRQNQK--EERKQLIYDEIDRLQ---------IEYY--EN---EKLKMHEVKKTLDEFI

KKMDRDFYYEQIELIERILKKKFIKKEE----ENMNEEITENETESIYDFDSFKDNEITRKIIGAIQKEKD-

--------VSH-RPRSLVIEGLSRIGKTEFIISYLNKNL-------------IPFNYIRGSLDFS-----KE

IYKNEYKINVFDDISIFEIK--------KHGLLKNIIGGQR-GFNADIKYAPKRRIAGNKLNIFLCNEDISF

VRFCKKNK------------------EMGGKEYEYIEKNCLFFNI---DEK---------------------

-----------------------------------------LYKENEE------------------------

------------------------------------------------------------------------

-----------------------

>pCRESS9|WP_017193695.1

M--KKTKKTNNKNAKIKE-------------------TKNKFRFSSKDIFLTYS------------------

-------------KCSLGKNVIHNHIKNLMNEKK----KNIIYIISNTE-----------------NHADH-

KEIHTHVLLQLE--KIFQTEN-ARFFDIEGFH--P--RIE----------------NAQHIEKSIDYIKKDG

DFIENGIPRIKKYVRQNQK--EERKQLIYDEIFRLK---------KEYY--EN---ESLKINKIRKNLDEFM

IKIDRDFYLEQIELIERILKKKFIKKAE----ENMNDNINENETESIYDFDSFKDNEITRKIIAAIQKEKD-

--------VQH-RPRSLVIEGLSRIGKTEFIISFLNKNL-------------IPFNYIRGSLDFS-----KE

IYKNEYKINVFDDISIFEIK--------KHGLLKNIIGGQR-GFNADIKYAPKRRIAGNKLNIFLCNEDISF

VRFCKKNK------------------EMGGKEYEYIEKNCIFFNV---KEK---------------------

-----------------------------------------LYKEND-------------------------

------------------------------------------------------------------------

-----------------------

>pCRESS9|YP_006959585.1

M--KKTNN-----------------------------IKKETMFQAQNIFLTYS------------------

-------------QCDLSKEEIKTFIINLCNEKK----LQINYLIIGIE-----------------NHQDH-

KGKHHHVFFQLN--KQFRTRDL-TIFNIPKNIHEP--HIE-------------PGNPIKDTTDVRNYVKKDG

DFIEEGTFKHVRYIKLSKNKPTELESLTNNYFLKL------------------RKEISKTKNEIFKKLKLYA

ESLEPNYAFKNAKRFKNMVFEYIFESD-----IKELPIFDFCTFKKIPILI------SIYDILETQKEQLS-

------NSISK-RFKTLIVEGNSKSGKTQFFKSVLTNLE-------------LPFNYIKDDVDFS-----DE

NYDEDKCVNIYDDIDIYDIQ--------ARNLTKVVIGNQK-DSIVNMKYKPRTKIK-GDISIMLVNEDTSI

EKYCFDNF------------------KRGRKEYKYIRENAIFINL---DKHTITHYEYQKYENEQAVIQHFE

M--------------------------KNWDGLLYFLDQEN-------------------------------

------------------------------------------------------------------------

-----------------------

>pCRESS9|WP_011264167.1

M--KKTNN-----------------------------IKKETMFQAQNIFLTYS------------------

-------------QCDLSKEVIKTFNINLCNEKK----LQINYLIIGIE-----------------NHQDH-

KGKHHHVFFQLN--KQFRTRDL-TIFNIPKNIHEP--HIE-------------PGNPIKDTTDVRNYVKKDG

DFIEEGTFKHVRYIKLSKNKPTELESLTNNYFLKL------------------RKEISKTKNEIFKKLKLYA

ESLEPNYAFKNAKRFKNMVFEYIFESD-----IKELPIFDFCTFKKIPILI------SIYDILETQKGQLS-

------NSISK-RFKTLIVEGNSKSGKTQFFKSVLTNLE-------------LPFNYIKDDVDFS-----DE

NYDEDKCVNIYDDIDIYDIQ--------ARNLTKVVIGNQK-DSIVNMKYKPRTKIK-GDISIMLVNEDTSI

EKYCFDNF------------------KRGRKEYKYIRENAIFINL---DKHTITHYEYQKYENEQAVIQHFE

M--------------------------KNWDGLLYFLDQEN-------------------------------

------------------------------------------------------------------------

-----------------------

>pCRESS9|BAD36752.1

M--KKTNK-----------------------------VKKETIFKAQNIFLTYS------------------

-------------QCDLSKEEIKTFIINICDEKK----IQINYLVVGIE-----------------NHQDH-

KGKHHHVFFQLN--KRLQTRDL-TIFNIPKNNNEP--HIE-------------PGNPIKDTTDVRNYVKKDG

DFIEEGTFKHVRYIKLSKNKPTELESLTNNYFLKL------------------REEISKTKNEIFKKLKLYA

ESLEPNYAFKNAKRFKNMVFEYVFESD-----IKEFPIFDFCTFKKIPILT------STYEILETQKEQLS-

------NSISK-RFKTLIVEGNSKSGKTQFFKSVLTNLE-------------LPFNYIKDDVDFS-----DE

NYDEDKYVNIYDDIDIYDIQ--------ARNLTKVVIGNQK-DSIVNMRYKPRTKIK-GDISIMLVNEDTSI

EKYCFDNF------------------KRGRKEYKYIRENAIFINL---DKHTITHYEYQKYENEQAVIQHFE

I--------------------------KNWDGLLYFLDQKN-------------------------------

------------------------------------------------------------------------

-----------------------

>pCRESS9|KXT29032.1

M----------------------KTKNALVVSK----KSKPYRMHSRNIGFTYP------------------

-------------NLSLSKEEVQKIFRKRKKK------IYYKALRISRE-----------------LHED--

GEPHIHILIQLN--KKTEFCNAREFFALPTFN-----ELE-----------CSFYFAQSTPEHWYRYIGAYG

DVLDDGIFKFKQFSNKQKI------DFIYAAHLK-------------------ASENEISAFEAEKALNQYL

QELDVVIYYKQFPIRDRVIQENFYPKSSVVKRVIEH-----------SLQTFRLYHEKVQLIIKTFKEQFN-

---------SK-SPLTIVLEGLTQIGKTDLAELIVQELK-------------VPYNYTKIDFNFS-----RE

NYNDSYKICIYDDMGMEEVN--------SKKLMHALIAGRG-SFQTREPYGKKRTISGNKLNIFIVNRNKSF

KGWIEKNK------------------EWKRFEHEYVEPNVIIIDL---FKD-FKKDYPLFYKPEEIKEMNLY

K--------------------------KGVNGRSSEILAKLMFGDGKVEVIKE-------------------

------------------------------------------------------------------------

-----------------------

>pCRESS9|KXT29014.1

M--ETNTLIIPKNRSLVIFNVQN--------------DWKPYRLKTKMVGLTYS------------------

-------------RFPVLKEKFIQLLSKNKNK------INVIYDISCND-----------------YHMD--

SGLHIHALLVFD--KKIQLRDAQKVFALPFINRTA--CFE-------------KQKFGNHLDRFRAYIIKKG

NFIENGIY--------KSRQDEREKKNELDIE---------------------RLRGLIDPVEAEKQLKSFG

YKLDNVDKLR--DLALKMSKDTF-VFE-----LLEE----------TEIYDFDSYLFPFNMV---------E

KMVLKLMNSGV-RKKSLFIVGGTGIGKTRMIKTILRKLG-------------LKYSYIKGKIDFS-----PK

KFDDSRPVVIMDDITLQKIYK-L----DPEDGFKNFIGNGD-TTEVDVKMQKTATIT-GKLFIYIVNPDKHP

EKWCVKND------------------EFEKYDHIYIRKNIEVIEF---DKEKYNDKRPLFYTDEEKKYRQKL

---------------------------KGVNGRSSEVLARLMFGDGKVEVIEK-------------------

------------------------------------------------------------------------

-----------------------
