# Supplementary material for: Multiple origins of prokaryotic and eukaryotic single-stranded DNA viruses from bacterial and archaeal plasmids
Source: Nat Commun. 2019 Jul 31;10:3425. doi: 10.1038/s41467-019-11433-0 (PMC6668415; doi:10.1038/s41467-019-11433-0)
Supplement: Supplementary file 4 — Dataset 3 [file 41467_2019_11433_MOESM4_ESM.docx]

**SUPPLEMENTARY DATA 3**

# ALIGNEMNT USED TO PRODUCE THE TREE SHOWN IN FIGURE 5

>pCRESS5|UniRef50_W1I5Y6

MNK-----NER--QRAFNLVI--NKNAKF----------NEVKQII-E--NLC---NVAL

YGL-ILHDKDIAEDGQIK--EPHYHLYLKFKNARTFQSLIKQF-------EGA-HIES-V

IN-ENQSIKYLIHNTSNAQG-KYQYSIDELL-TNDFNKIQEI-----------L-KEEDY

IVEN-----IPKY-IASGIL-HPY-------S-FSPNTFKANWGMYKE-IITSY------

--------------------------KNR-----DD------------------------

----------------------S----LLVDE-VEQIEKELKK-Q---------------

------------------------------------------------------------

-----------------------------------------------EDQEEQ-------

---------------------TDEELPF

>pCRESS5|UniRef50_R5VXD3

MQK-----NPR--SRNWSLV--------Y----------LTKQELL-Q--RLCANENVRY

YAF-IVHDKDTLQDGSAK--PLHVHLALCLNSARTAQQICHRF-------TDI-TSNACF

GQ-PTRSNKAIFEYFTHSPD-KYQYSETEIF-SNNIDYFKND-----------E-KDEDN

ILND-----ILQG-KP-----------------------------LRE-LVKIY------

---------------------------GR-----EL------------------------

----------------------L----YHYSQ-FKEVALDIQQ-Q---------------

------------------------------------------------------------

-----------------------------------------------ETPKGT-------

---------------------VDENDPF

>pE194_pMV158-like|UniRef50_A8W662

------MAKEK--SRNFTFLL--YPDGF--P-----DWEERLEKI-----------GVPI

AIS-PLHDKDKNKNRTLK--KPHYHGIYIANNPVTAESVKNKLRVLSSEAKVQ-IIY---

ES-IESVYLYLTHESKDAKN-KYRYDKADIKHINNFDIDRYV-V--VDV-ET--------

--KNQILKILLQI-IRAYSI-PNVDL----------------------------------

----------------------------------------------H-------------

---------------------------DFEEN---------GS-SGI-DMNLFLTTESKS

S-----ILRLY---------------FDGAYQ--------------RGQRGLKSGE----

--E-----KNG-------------------------------------------------

----------------------------

>pE194_pMV158-like|3DKX_A

------MAKEK--ARYFTFLL--YPESI--P-----DWELKLETL-----------GVPM

AIS-PLHDKDKSKGQKYK--KAHYHVLYIAKNPVTADSVRKKIKLLGE-AMVQ-VVL---

-N-VENMYLYLTHESKDAKK-KHVYDKADIKLINNFDIDRYV-T--LDV-EE--------

--KTELFNVVVSL-IRAYTL-QNIDL----------------------------------

----------------------------------------------Y-------------

---------------------------DFDE-----------------------------

------------------------------------------------------------

------------------------------------------------------------

----------------------------

>pE194_pMV158-like|UniRef50_A0A1Y4QQC0

MT--VNKTKVK--ARSFAFIL--YPESV--P-----NWIDCLTKL-----------GVPM

AIS-PLHDLDQSKEKIYK--KPHYHVLYIAKNPVTAESVRIKVKVLGD-SHVE-IVD---

-N-IEYYFQYLTHESADAKK-KHKYSREDIVYLNGFDIERYI-T--LDE-SE--------

--KRELVNLILAL-IRKFKI-ENIDL----------------------------------

----------------------------------------------S-------------

---------------------------EFEMY---------GD-DGLPNMSRINDVSSKT

G-----LLRLY---------------FDGNYQ--------------RRKRQQNSIY----

--D-----EKE-------------------------------------------------

----------------------------

>pE194_pMV158-like|UniRef50_A0A0E9F9L7

-----MPAKSK--GRYFLAVL--YPDNM--R-----AWKDEIGDLL----------GYPY

AYC--IHDKDKTKEGEQR--KVHVHMILAFSNTTTIQNAMSIFSLSADGNKIE-IAS---

-S-VVNAYNYLIHDTESCLK-KHLYDRAERVTGNNFDIGDYE-Q--IST-AD--------

--KDRMLKEMCDY-IIDNQI-TNFDM----------------------------------

----------------------------------------------Y-------------

---------------------------IRTQD---------FD-T------SYFAVKSYS

G-----LLERL---------------TRGVFL--------------HGERSDKLEK----

--QHEQRHEKHNSAQII------------KSGK------------TSINTQRFK------

----------DCG---KTFTR-------

>pE194_pMV158-like|UniRef50_A0A174GG61

MA--ANAGETK--VKLWEGIC--YPENM--L-----DWREEIDDIL----------QVPF

AYA--IHDIDHDQKSKQR--KTHVHIIVVWGGNTTRKAIINVLNLSADGSSAE-PVN---

-N-IRHAYDYLIHDTASCKG-KELYPVEARIEGNNFDIGQLE-Q--LST-KD--------

--KQDMLFELVGF-IMVEKF-ETIDF----------------------------------

----------------------------------------------T-------------

---------------------------TALRE---------FP-E------QYREIVGYN

S-----ILERY---------------CRGNYL--------------NAERKRKARE----

--TG-------------------------ACGK--------------VDGSMEQ------

----------TTG---AE----------

>pE194_pMV158-like|UniRef50_W1I697

------MGRKK--VRYWQGIF--YPENM--I-----TWRDAIGSMC----------SLPL

EAI--LHDKDLNKDGTRR--KEHIHVIIAWNNTTGALSAFKTFSLSANGNTLE-AVQ---

-N-IGNAHNYMRHATDDAKG-KHLYAEEELLSFNNFDIGLFE-Q--LGV-VE--------

--EEQIKTQIEAM-ILEGRI-KNYKL----------------------------------

----------------------------------------------K-------------

---------------------------LMRQL---------KD-T------RYIKVEKNI

Y-----EFSTL---------------TRSMW-----------------QWEEEYRK----

--EYGQGYEKEFTGEV-------------ISDK------------TEADTEATE------

----------K-----------------

>pE194_pMV158-like|UniRef50_A0A1C6AUS2

APTSQVKKEQR--TRNFATVV--YPESA--P-----DWIDKLDQL-----------HVAA

LIS-PLHDKDTNPSGEPK--KPHYHVLLMFEGVKDYTQVKPIFAIGGVG--RE-MVN---

-S-ARGYARYLCHLD---PE-KAQYEPSEVRCLGGADYTDIT-N--LPT-DT--------

--RKML-AEIMGY-IQENEI-FSFEF----------------------------------

----------------------------------------------I-------------

---------------------------DLRLY---------HP-D------WFTLINTNG

W-----IVKEF---------------IKSLE---------E--RSVKAERLPKAD-----

-----------ETGEV-------------------------------LDSE---------

----------------------------

>pE194_pMV158-like|UniRef50_A0A0E9DRD0

MPG---KAQTR--TRNYATVI--YPESV--P-----DWIDRLSNT-----------HVAA

FIS-PLHDKDVNPDGTRK--KAHYHVLIMFEGVKTIAQAKTIFDIGGVG--CE-IID---

-T-IRGYARYLCHMD---PE-KAQYKADDVQALNGADYLSTI-S--LIT-DK--------

--YKVL-REIMAY-VRDQNL-LSYDL----------------------------------

----------------------------------------------L-------------

---------------------------DYAEN---------RQ-D------WFRVLDSGT

Y-----VISHY---------------IRSLA---------K--QSK--------------

------------------------------------------------------------

----------------------------

>pE194_pMV158-like|UniRef50_G8CNT2

MAR---KSVQK--TRGYACVI--YTDSA--P-----DWKERLIET-----------HIPA

LIS-PYHDRDINADGTPK--KPHYHVMIIFDGPRTLAQAEDVLTIGAAN-QVK-PLN---

-S-ITGYARYLCHLD---PD-KVQYDQGDVVALAGAEYDVYI-N--RSE-DK--------

--QQTL-REMQDF-VDRYAV-YSFQL----------------------------------

----------------------------------------------L-------------

---------------------------RYRQY---------NK-V------WYRHLESCS

Y-----TMKEY---------------LKSAY---------E--DQDSDLKYRIVD-----

-----------QTGEVV-----------KIGSK-ASRF-AAAPSYVIQDGKREI------

----------DPE---TGEII-E-----

>pE194_pMV158-like|UniRef50_A0A0E9F4G4

MP--KPKTVKK--VRAIAGIL--YADSA--P-----DWQDRIADL-----------HMMA

LVS-PLHDSDVQADGSPK--KPHYHVMLIFDGPVAVDRAAKLLQIGCID-YTQ-SIH---

-S-VTSYARYLCHLD---HD-KHPYNTQDVSAYGGADYELMC-Q--RSL-DK--------

---DTAITEMEDY-IDTHHV-YSFRF----------------------------------

----------------------------------------------A-------------

---------------------------QHRAN---------QP-T------WHRHLTDCG

W-----YIKEY---------------IKSAY---------D--DLTDQDKHD--------

------------------------------------------------------------

----------------------------

>pE194_pMV158-like|UniRef50_A0A158LH93

MAA-KMAAKDR--KRNWVFVV--YPESA--P-----NWREQLKEM-----------LVPG

FIS-PLHDKDVNADGSPK--KPHWHVMLTYKGVKSYEQVKAVTDLNAPP--PQ-ICK---

-D-IRAYARYLCHLD---PE-KAQYEPAQVENMCGTDYLENI-R--SAA-DT--------

--DTAL-SEMMDW-CMAERC-YSFRL----------------------------------

----------------------------------------------S-------------

---------------------------NYRLN---------RP-D------WFRVLQSRT

Q-----FLVAW---------------LKSMK---------E--ITQ--------------

------------------------------------------------------------

----------------------------

>pE194_pMV158-like|UniRef50_A0A0H5PZI7

MS-SSGNKTRR--TRTYTCIV--YPDSA--P-----NWRDIIDGY-----------HIEW

AAS-PLHDRDINADGSAK--KPHWHVLLCWDSVKTPEQAGEVAQVNGTI--VQ-PVQ---

-S-VRALLRYMCHLD---PD-KAQYDKAMLETHGGLDASSAL-R--TAD-VA--------

--VSVLVRDMLSW-CRDNGV-TELDL----------------------------------

----------------------------------------------M-------------

---------------------------DYAEC---------EP-D------WWDALHSCA

Y-----VVGQY---------------LKSRR---------------KLEM--QALQ----

--RT---------DE---------------------------------------------

----------------------------

>pE194_pMV158-like|UniRef50_G8CNR9

MPYRERKPDVR--ARAWTFIV--YPESA--P-----NWRDVLDGF-----------HLQW

ACS-PLHDRDVNATGEPK--KAHWHILLSFGGKKGYGQIWSISEINGTR--PQ-VCQ---

-D-QKALIRYFSHRD---PE-KAQYKASDIEARGGFDLEEYL-K--PTA-SE--------

--CMAMQDEMVEW-CLKYNV-TEFVL----------------------------------

----------------------------------------------K-------------

---------------------------IYIRE---------RP-D------WSAELRSC-

F-----QITQY---------------LKSRRH--------------GVDVKAYN------

-----------ETGET-------------------------------YE-----------

----------------------------

>pE194_pMV158-like|UniRef50_W1I557

---ENLKKDDR--TRNWTFIV--YPDSA--P-----NWEQILINQ-----------AVPW

FCS-PLHDSDLNADDSEK--KPHWHCALCFENKKNYEQILEIISLNCTI--PQ-KVK---

-S-MKSMLRYFIHAD---PE-KHQYEKSDIRCFGGAEYESFF-L--PTR-TD--------

--KMSFYGEIIDW-IEENDI-IEFDL----------------------------------

----------------------------------------------V-------------

---------------------------TYRMN---------EP-D------WASMLTNST

I-----FFNSY---------------ITSRRN--------------KRTANDI-------

------------------------------------------------------------

----------------------------

>pE194_pMV158-like|UniRef50_A0A0E9EV38

-----MSKNVK--KRNWAFVL--YPESA--P-----DWREQLQKT-----------GLQC

AIS-PLHERDINPDNTPK--KPHYHVILAYSGPTSYNVVKSLTDFNQPI--PQ-PLE---

-Q-VRGYYRYLSHKD---PE-KAQYDEREIRTINGFNIADFS-E--LTR-SE--------

--VTQIKRTLQGL-IRQYDI-IEYEL----------------------------------

----------------------------------------------M-------------

---------------------------DFQDE---------EM-N------VEYEVSNNT

L-----FFDRY---------------IGSRRHS-------S--RIKQSTENQQHEQ----

--HEQH--EEIKFGRT-------------------------------QAGRARM------

---------NDCG---KTWVI-------

>pE194_pMV158-like|UniRef50_U2EU97

------AKNVK--KRNWAFVA--YPESM--P-----NWLEILQET-----------GAPI

AIS-PLHDKDLNADEHEK--KAHYHVICCWDGPVRFTQAEKLAKVNGTI--PI-PLE---

-S-IRGYYRYFTHKD---PE-KHQYDESEIKCLNGFAITDFV-E--LSK-SE--------

--VVKIKYDILDL-IEKNGF-CEYDL----------------------------------

----------------------------------------------I-------------

---------------------------EFKN-----------------------------

------------------------------------------------------------

------------------------------------------------------------

----------------------------

>pE194_pMV158-like|UniRef50_A0A0H5Q0X0

MPYPANKKDVK--KRNWWFVL--YPESA--P-----DWRDRLRAT-----------GLPI

AIS-PLHDKDVYDNGQPK--KPHHHVILAYPGPTTYSVVCKLVQLGQPI--PQ-PLE---

-A-IRGGYRYLSHAD---PE-KYQYDAKDIETLNGFNILDYV-D--MTA-AE--------

--AVAMNKELVSL-IMELGF-ADYDF----------------------------------

----------------------------------------------I-------------

---------------------------EYLFN---------GT-D------VQYEYISHT

L-----FFTAY---------------LKSR----------Q--REKQQEKEDRQKL----

--SNGV--DDTGPGST--------------------------------------------

----------------------------

>pE194_pMV158-like|UniRef50_K9RZV9

---DKKTKAIK--KRHWAMVI--YPESL--P-----NWKEILQQT-----------GCEI

AY--ILHDKDIEPTGEPK--KPHYHVIMSYTGTTTFNNVKRITDLNAPI--PQ-GIE---

-S-VGGYYRYLTHKD---PE-KYQYDEKDIVHLNGFSPFNYI-E--LKK-GH--------

--ELKLKQEILAF-IKESGI-LLYEL----------------------------------

----------------------------------------------N-------------

---------------------------EYMNT---------GQ-D------LYFKVSENT

N-----YFSAIL----------KAKGLKAR----------K--DECKDEQ----------

------------------------------------------------------------

----------------------------

>pE194_pMV158-like|UniRef50_A0A1W6BZG0

MP-QKSNKETR--ARAWSFIA--YPDSV--P-----DWEQILTERF----------NLKW

ARS-PLHDSDINADETQK--KPHWHIVVTFTNVKSYEQVKEISDINATI--PQ-KVH---

-N-LQGLMRYFLHLD---PE-KAQYDFKDIKAV-GINAEEIL-G--QTD-FN--------

---KKLKNEIFEL-IRGNDL-FEYEL----------------------------------

----------------------------------------------L-------------

---------------------------DFQYD---------HE-Q------HFEYATTHT

I-----LFVAY---------------LRSRRH--------------KAESTA--KR----

------------------------------------------------------------

----------------------------

>pE194_pMV158-like|UniRef50_A0A0R3QHC2

----MKTKDTR--ARNWTFIV--YPESA--P-----DWRERLDGL-----------HVPW

AHS-PLHDKDVNPTGEVK--KAHWHVLITFAGNKSYQQMLEITKIKAPN--PQ-KCA---

-S-AKGLIRYFCHLD---PE-KYQYPTADIVSH-GVDVQGLL-E--LTR-SE--------

--LNQVLKEIFDF-IDDNTI-YSYDL----------------------------------

----------------------------------------------L-------------

---------------------------QVRSS--------GKD-D------WFDVCNRNT

L-----AIKEY---------------LKSAT---------T--DRNKTACEDE-------

------------------------------------------------------------

----------------------------

>pE194_pMV158-like|UniRef50_A0A0H5PZW4

---MSKSQAKR--GRNWVFVI--YPDSL--P----GEPFKTLDEE-----------KVPY

FLS-PIHDKDLNADETQK--KPHQHLILMFEGNKSEAQMNDLAKFGCPR--PK-MIQ---

-N-LRSMARYLCHLD---PE-KAQYLVEDVKSGYGADYSKII-N--LTE-NV--------

--YIVI-AEMMDF-INDNDI-RYYDL----------------------------------

----------------------------------------------L-------------

---------------------------EYRMN---------ND-T------WFRALDKCR

E-----NVYRY---------------IYSRAT--------------KLEKQEQE------

----------------------------KAAQRYMDYV-KYPPKHSSKDEETE-------

----------------------------

>pE194_pMV158-like|UniRef50_A0A1Y4G0S9

MAE-DGRRASK--TRNWAFVL--YPESA--P-----NWRELLAGM-----------HMPA

LVS-PLHDRDVDECGNPK--KPHRHVILMSDGPITQKRANALMEFNGTK-SAE-YVR---

-S-LRGYVRYLAHLD---PD-KAQYDPGEIEEFGGADLAALL-K--PSE-AG--------

--RYEVIGQMIDF-CADHSI-TEFEL----------------------------------

----------------------------------------------L-------------

---------------------------RYRKE---------RP-D------WFPVLDS-A

F-----LMSRY---------------LTSLRC--------------SATSKRRSD-----

-----------------------------------------------AE-----------

----------------------------

>pE194_pMV158-like|UniRef50_UPI000300949E

MSD-SNNVNRR--SRVWGFLA--YPESI--R-----DWIEFLTDEL----------NLVW

ACS-PLHDADIDKDGKPK--KPHYHCVMVFDGKKSYSQIKEITDISATN--PQ-VIN---

-N-LAGNIRYMLHLD---KS-KAQYLASDLKAFNGFDANEYL-F--SEK-SEL-------

--RLECLKAMRKF-IDENGY-TEFQI----------------------------------

----------------------------------------------F-------------

---------------------------DYAEN---------EP-D------WFEMLVNSA

Y-----VINVY---------------LKSKRH--------------QSLVKM--------

------------------------------------------------------------

----------------------------

>pE194_pMV158-like|UniRef50_A0A0H5PV05

MSKNTLPKDGR--VRNITFML--YEDSC--N-----DWKELLDEE-----------HIP-

HLW-IYHNKDINPTGEAK--KPHYHVVLCFDNKKSQKQCQAYADFGAAN-VYK-EVQ---

-S-LRGMARYLCHMD---PE-KAQYDSSEVHSC-AMDYNSIV-G--MAQ-DK--------

--YKAI-DEMIQF-CEEENI-ISFEL----------------------------------

----------------------------------------------L-------------

---------------------------MYRVH---------RF-D------WFKSLDNSS

F-----IIKEY---------------LKSKV---------E--MSIREE-----------

------------------------------------------------------------

----------------------------

>pE194_pMV158-like|UniRef50_W7D2V3

MHN-KKNAGLR--ARNWTLII--YLDSA--P-----NWKEILTET-----------GVPW

AHS-PLHDKDVDVNGCIL--KPHYHVVIKFSLLKSYQQMLKLTGLRAPN--PQ-VCV---

-S-LVGSVVHFTHSNK--KE-KYQYDQQEIESYNGLDIEELL-K--PTK-TE--------

--MNEILKEIRAF-IIENDI-QEFRL----------------------------------

----------------------------------------------C-------------

---------------------------QYDKE---------TS-N------WSRVITKHT

------LINQF---------------VTSYRH--------------SRDKRKEV------

-----------QVDEIQLESA-------KKSQKNAELQ-AL----GVKEYQESY------

----------ECQ---YSVQV-------

>pE194_pMV158-like|UniRef50_A0A0H5PZG0

MPFNEKRKGTR--SRGWACII--YPESA--P-----NWTETLNGA-----------HIET

LIS-PLHDKDVTAEGSPK--KPHYHVLGLWPQPVPAAAAAEYFGIGVTA-PPE-MVK---

-N-SKGYARYLVHMD---HD-KHRYSEHDVTALAGADWYSVA-L--DEG-EA--------

--VNAYLDEIESF-IDDNSC-ISYAL----------------------------------

----------------------------------------------C-------------

---------------------------GYREC---------RP-E------WTEVIHNTI

H-----LMA-Y---------------IKSFE---------E--IKMESLKNS--------

------------------------------------------------------------

----------------------------

>pE194_pMV158-like|UniRef50_A0A0H5QIL6

MPIDAKRAGKR--ARAWTALM--YQESA--P-----GWMEKLSNL-----------LVEA

LIS-PLHDQDVTVEGEPK--KAHWHIVVSYKNPTTYEIAKEVFDIGAVM-PPEPKVK---

-D-FKQMARYLCHLD---PS-KHRYDTADVKSFGAIDYQALV-M---SG-QD--------

--EDEMIDEMCEF-IDANMI-TSFQF----------------------------------

----------------------------------------------C-------------

---------------------------VYRNE---------RP-E------WRVLVHQYA

A-----LLSRY---------------IKSLA---------E--MEQER-RNL--------

------------------------------------------------------------

----------------------------

>pE194_pMV158-like|UniRef50_A0A0H5PZA0

MAKEAKRAGQR--SRNWAVIF--YPEDL--P-----DWQGILSEQ-----------HFGW

IEG-PLHDQDTNADGSPK--KPHVHTLLMFGGVKTAEQVGKLLKIGVAT--PQ-QAT---

-D-RSSVVRYMAHMD---PE-KAQYDPADIVGHNGADPAEIL-R--YSA-TE--------

--KREMVLAMEEF-IEQNNI-TELDF----------------------------------

----------------------------------------------S-------------

---------------------------AARYD---------RP-E------WHVILTQMT

V-----YFNAF---------------IRSRRH--------------KAEHGGQAPK----

--VD-------TTGEVI------------------------------EKG----------

----------------------------

>pE194_pMV158-like|UniRef50_A0A0Z8IYX5

MSD-LKHIPQR--STMWTFLL--YQESA--P-----NYVDILNDL-----------QIPW

IVS-PLHDADLDKDGNIK--KPHWHAAFFFDSLKSYKQVSSLVTLKAPS-HVQ-IIH---

-S-TTGFFHYLTHAE---PE-KAQYSLEDIQYGAGFNLEQFL-Q--SQN-ID--------

----SYLGSIIDL-VETENI-KEFDL----------------------------------

----------------------------------------------V-------------

---------------------------TYRQS---------DT-A------LLKLINR-A

Y-----FFSRY---------------LDSRRH--------------SRNNIKVIDH----

--LT-------LTEENI----------------------------SQKDED---------

----------------------------

>pE194_pMV158-like|UniRef50_A0A087EKU7

MSD-KNNPNEK--GRYWAGLI--YPDSC--P-----NWQELMQLS-----------GLQI

LVS-PLHDLDIDVKGELK--KPHRHVIAMWMNTTTRRNAQRFFEFNGPK-TII-RLE---

-S-PRGMARYLIHLD---PE-KAQYLPENVLAFNGADWQKIA-I--PDD-DK--------

--QEAM--SIVNL-VADNGI-QGYDL----------------------------------

----------------------------------------------L-------------

---------------------------KLEQE---------HP-D------LLDFARQTV

FC---EVIWSY-----------------------------H--ATASRRRRSIMTE----

-----------QTDNIANNQPQF-----GRSKARLMMD-ALP---SIRVGRKDYATVLNT

FIENSGIPWPKCK---R-----------

>pE194_pMV158-like|UniRef50_D3R6U9

------SLNDK--GRYWAGLI--YPDSC--P-----DWKATMQMS-----------GLQI

LVS-PVHDKDIDPKGKLK--KPHRHVVAMWTNTTTRRNATRFFEFNGPK-TIL-RVE---

-S-PRGMARYLVHLD---PE-KVQYLPEDVIEINGADWKKIA-L--TDE-ER--------

--PEVM--ELLEL-IEEWEV-HGYEL----------------------------------

----------------------------------------------L-------------

---------------------------KLEL-----------------------------

------------------------------------------------------------

------------------------------------------------------------

----------------------------

>pE194_pMV158-like|UniRef50_A0A0S2MGE2

MTH-RKTTNEK--GRYWAGLI--YPDSC--P-----DWEDRMKIS-----------GLEI

LVS-PVHDQDIDIAGELK--KPHRHVIAMWANTTTRQNAERFFEFGGPK-VIL-RLE---

-N-PRGMARYLIHMD---PD-KAPYSPEDVLEFNGADWKKIA-L--VEG-NQ--------

--SDAM--SIVKM-VEEENI-HGYDL----------------------------------

----------------------------------------------L-------------

---------------------------KAEAD---------YP-D------LLEFAKQVT

FC---EVIWSY-----------------------------N--SYF--------------

---------------------------------------ALPK--EDRHGKA--------

----------------------------

>pE194_pMV158-like|UniRef50_U2QZX4

MPVSPELKKLK--RRHWLYIV--YPESA--P-----DWKEQLALT-----------GVQF

AVS-PLHDRDLLVNGDLK--KAHWHMIVIFDGPTTFLTAASFRETHGPY--PK-VCE---

-N-LRGSFDYFTHKN---PD-KAQYLSDGIELYNGFKID-------LSA-KD--------

--VQRIKNELCEI-IIKQNI-TEYEF----------------------------------

----------------------------------------------N-------------

---------------------------LYQYY--------LEA-D------YCDVINN-T

Y-----HFNSL---------------INSFRH--------------NPEKIKARYK----

--VIQE--E--MKSEKM----------------------------EDKKNDS--------

----------------------------

>pE194_pMV158-like|UniRef50_A0A0H5Q8X5

----MSSNSSK--SRYWMFLM--YPDSM--P-----NWKQILTDT-----------GVAF

AIS-PLHDKDKKADGTPK--KPHYHVLVSYPNNTTIGPIDEIRAVNGTE--AK-RVF---

-A-AIGQYRYHLHMD---PE-KAQYDHSDRTILNGFDDKNLG-G--KSL-SE--------

--INRLKLDLKEI-CKQFVI-TSYDL----------------------------------

----------------------------------------------I-------------

---------------------------DIESE---------DG-E------ELIDILNNS

I-----FINAI---------------CDSVFH--------------KVSRVAQKTD----

--EAFE--SAIKTGEVKHISDI-----------------------INK------------

----------------------------

>pE194_pMV158-like|UniRef50_F0HMF3

MPYEQEKRKQR--RTNWMLVF--YEDQC--P------WRDELDEL-----------GMRT

LVS-PSHDADEARDGTLK--EPHRHLLAMYDNPVSYDQVVKDFALKSKN--VK-YVK---

-S-LPAMARYLTHMD---PD-KAQYDPEGVCEFGGADWRDLC-A--TTS-DK--------

--HLAL-REMRAF-IRENNI-VDFLF----------------------------------

----------------------------------------------W-------------

---------------------------DWDEH---------ND-E------WSRLLDSCC

Y-----AIEHY---------------MRSFRA--------------QEDRDALRAK----

--RV-------QTGAIQ----------------------------S--------------

----------------------------

>pE194_pMV158-like|UniRef50_U2TJ01

------GRAGK--ARAFAFLI--YPDSW--P------WEKDLRGL-----------HMPI

VVS-PIHDSDVTEDGELK--KPHYHGIVSWGNATTMNAALNLLEFGV--RHVE-PVG---

-S-YSAYCRYLCHMD---PD-KAQYDAADVVCLGGVP-DFER-K--LTE-SE--------

--MLAQRDEIMAL-CEGNGV-VEYDL----------------------------------

----------------------------------------------C-------------

---------------------------DFRYH---------RP-D------WRQDVTH-T

I-----FWRGY---------------FASARS--------------RGTGGGAVCS----

------------------------------------------------------------

----------------------------

>pE194_pMV158-like|UniRef50_A0A1Y3UDM3

---MADSSAVK--ARYWTFLV--YPESA--P-----DWLGALKRS-----------HGSY

AVS-PLHQP----DDETS--KPHHHVIYQHGNTTTLNGAKAAI-EDVPAGYVE-PVA---

-N-PSNMQRYLIHLD---PE-KEQFDGANSITLNGFPLDLTR-E--LSK-SE--------

--KARIRLDLMSI-IRENVV-TEYDF----------------------------------

----------------------------------------------IF------------

---------------------------GLDMG---------DP-D------MLDYASH-T

I-----LFEGV---------------IRSVRN--------------RA----KVVQ----

------------------------------------------------------------

----------------------------

>pE194_pMV158-like|UniRef50_A0A1B1IHL4

IQDESYANTDR--AKNYTLVV--YPDDM--P-----NWLEIMRED-----------MFDM

VIS-PLHDKDVNPDGEPK--KAHYHLLVSAGSWIRMGTLANWGKLKGIA-RPQ-KCS---

-N-PKGLVRYMIHQD---PE-KYQYNKADIRVIGQYDIEPFF-K--ATI-GE--------

--DRETRKEIMHF-IIDNDI-VEFDL----------------------------------

----------------------------------------------V-------------

---------------------------EYLVH---------NE-T------WDDYLNN-T

L-----YIKNY---------------VSSRRF--------------RDIERKREAE----

--LEKM--SIL----------------------EKDIE-ALK---SMKRA----------

----------------------------

>pE194_pMV158-like|UniRef50_K7YFJ8

MPKKYNAKNPP--CKTYGFLI--YPESA--D-----NFRQIISENF----------DGSW

SLS-PLHDSDMHEDGTLK--KPHYHGIITFDKKQRPSALKRILEVGANP-FVIANSE---

-R-VKGAYEYFTHQN---PE-KAQYNETGIQLFKGFDINDFK-S--LKE-LS--------

--KKAMLKDIFSF-VELNEL-VSFAL----------------------------------

----------------------------------------------M-------------

---------------------------SYKAY---------RP-E------WFEFLESHS

Y-----IVINF---------------IKSMS---------E--KEKKELPTE--------

------------------------------------------------------------

----------------------------

>pE194_pMV158-like|UniRef50_O31070

APNKKLAKN-T--ARHWTILI--YPESV--N-----NFRDILCNTM----------AFEW

VLS-PLHDKDLNDDGTPK--KPHHHLLLVFDNAVRINIILEICRINAPE-Y----AQ---

-Q-VRGGYEYLTHKN---PN-KAQYSESDIECFNGFDIKEHM-S--LKE-LK--------

--DDLILNEIFDF-VERNEI-VSFHL----------------------------------

----------------------------------------------L-------------

---------------------------AYRKF---------KP-E------WFNLISQYV

Y-----FITQH---------------IKSIA---------E--KLNK-------------

------------------------------------------------------------

----------------------------

>pE194_pMV158-like|UniRef50_S6F6F1

MS-TPKAPEPR--YRNWGLVL--YPESV--P-----NWEEILIEE-----------GVPF

AY--ILHDKDQDENIKLK--KAHYQIIMKYKNQKTKAQMADLTKVSSPA--PI-PLG---

-S-LEASARDLLHLDQ--PL-QHKYDLSEVQVILGLDFQYLI-R--PTK-TE--------

--QNAIMRDIRHI-IREHEI-NEIDL----------------------------------

----------------------------------------------W-------------

---------------------------DFDEI---------NP--------FYSMVDAKT

Y-----AISSY---------------INSCRH--------------KPKKRRDVTK----

--VS--------------------------------------------------------

----------------------------

>pE194_pMV158-like|UniRef50_K9RYD5

-----MEPTVK--KRYWAVIT--WPESM--N-----DWYDQLVSS-----------GLQ-

ATF-ILHDKDIDENGEPK--KPHYHFILAWENSTTYKNVCEVVDIFGSSLLPK-PIE---

-S-LKGKYDYHIHKN---KD-KYQYDERERCFVNGFNIVNFL-S--KTE-SY--------

--PIDIINIIHKY-IDDNNV-LHFEL----------------------------------

----------------------------------------------A-------------

---------------------------SHKAN---------GQ-K------FEYETVKYT

F-----YFTEI---------------TKNKTYI-------H--R--KAELREQIKQ----

-----------LKDEYMHID---------------DVM-ELLEKHKEKDEK---------

----------------------------

>pE194_pMV158-like|UniRef50_A0A0R1P770

MP----TKEPY--ARNWWFTC--DSETL--P-----KWRETLDKE-----------QTAW

IES-PLHDKDITTNGDPI--KPYLHIIMMYKGARSYSHMKAITDLDQEA--PR-VVN---

-D-TKCLVRFFVHED---PG-KHHYNRDEIICHGGADSEKYF-Y--ISP-RAKK-RAEK-

--NEEILKNIVIY-TMENKI-TNYDL----------------------------------

----------------------------------------------V-------------

---------------------------RYLDN---------EK-D------WLNVLTDHG

TM----LVSAL---------------IESIER--------------KQMEI---------

------------------------------------------------------------

----------------------------

>pE194_pMV158-like|UniRef50_Q48831

MT-KKKYEYEK--GRDWTFIV--YPESA--P-----NWRTVLDET-----------HLLW

IES-PLHDKDMNADGEIK--KSHYHILLTFDGPVTEKQVIKLIDLNTPL--LK-KLG---

-C-PRWFSVVIWHIW---TL-KNINQRDEIVGHCGADVESYF-E--LTK-TS--------

--KMSVMKTIITY-IYEIRL-IIMIF----------------------------------

----------------------------------------------N-------------

--------------------------------------------D-L----YSNILIGLT

YRTTTLAINKM---------------IDGMWL--------------KKKNELR-------

------------------------------------------------------------

----------------------------

>pE194_pMV158-like|UniRef50_UPI000481AAFB

MPASEYTKKLK--STWWAFIA--YPESV--P-----GWIKKLQAS-----------GLRI

AIS-PLHDKDKNAEGDAK--KAHWHGIAVSDKRISAIEANAIIRTKGPY--VQ-KCR---

-S-LHLAYRYFTHED---PE-KYQYEKDEIIKVNDFHLE-------PNK-YE--------

--VGKLQADIIRD-IKDHNL-DEWKV----------------------------------

----------------------------------------------M-------------

---------------------------EFIN----------SP-E------MMVIISK--

----PGAITSY---------------VRSLWK--------------KNHPEGTVQR----

--IR-------ESGE---------------------------------------------

----------------------------

>pE194_pMV158-like|UniRef50_S6CES9

MA----LNPKK--SRCWWFVQ--LVEYL--P-----NWRDQLHDL-----------MLPG

CF--IVHDRDTDDDEVPK--KPHIHCMIEFGSPVVAKSALESIPFGVTF--VK-PVP---

-N-KVGAYRYLLHYD---PD-KARYEQDEITHMAGFRVN------------------IS-

--YNIDFCDVYEL-INEMKI-SNFML----------------------------------

----------------------------------------------M-------------

---------------------------SFVEF---------RP-E------YVNYVSH--

----VNLVKTY---------------ITELNR----------------------------

------------------------------------------------------------

----------------------------

>Gemini|ADN84041.1

MP--L-QRRFRLNAKNFFLT---YPKC---SI----S-KEEALGQLLSIP-TPV--NKKF

IKIC----KELHENGE-----PHLHVLIQFEGKYQCTNNR-FFDLVSTTFHP--NIQGAK

--SSSDVKSYI---------DK------D-----G-DTLQW-GEFQIDGR---SARGG--

--QQTANDSYAK-AL-NAE--SAEQALQIIKEHQP-QHFVLQYHNLVSNITKIFHKPPEP

WVPPFQLSTFNNVPAIMSEWVNVNIS-DAAARPLRPISIIVEGPSRTGKTIWARSMGPH-

-------NYLCGHI--------DLNPKIYSNDAWYNVIDDVD-PHYL--KHFKEFMGAQ-

--RDWQSNCKYGKPIQIKGGI-PTIFL-CNPGPHSSYK--EFLGEE-KNT--SLHDWAKK

NA---IFVSIEEP--L--------------------FTT--TNQTAAQIREK--------

---------TSE----------TKKD--

>Gemini|AAN76737.1

MP--P-PKRFRVSSKNYFLT---YPRC---SI----S-KEETLSQLLNIE-TPT--NKKY

IKIC----EELHEDGQ-----PHLHVLIQFEGKFVCTNQR-LFDLVSPTFHP--NIQGVK

--SSSDVKAYI---------DK------D-----G-VTIEW-GQFQIDGR---SARGG--

--QQSANDSYAK-AL-NAD--CIDAAMTVLKEEQP-KDFVVQYHNIRSNLEKIFTPPTEP

WVPPFQLSTFNNVPLIMSDWVNENIS-DSAARPLRPISIIVEGPSRTGKTIWARSLGPH-

-------NYLCGHI--------DLNPRIYSNHSWYNVIDDVD-PHYL--KHFKEFMGAQ-

--RDWQSNCKYGKPIQIKGGI-PTIFL-CNPGPHSSYK--EYLSEE-KNK--SLNDWAQK

NA---IYVSITEP--L--------------------YST--VNQTASSSRQE--------

---------TDS----------T-----

>Gemini|AAB87607.1

MP--R-SGAFRVNAKNIFAT---YPRC---SL----P-KEEALELLRQIP-TAV--NKKF

IKVA----RELHEDGE-----PHLHVLLQFEGKLQITNPR-LFDLVSGNFHP--NIQGAK

--SSSDVKSYI---------EK------D-----G-DTISW-GEFQIDGR---SSRGG--

--QQSANDAYAA-AI-NSG--SPTKALNIVKELAP-KDFVLHYHNIKSNLDRIFSKPLEP

YSCPFRLNSFNNVPQIMKDWASINVV-DAAARPDRPISIVIEGESRTGKTMWARSLGAH-

-------NYLCGHL--------DLSPKVYSNNAWYNVIDDVD-PHYL--KHFKEFMGAQ-

--HDWQSNTKYGKPIQIKGGI-PTIFL-CNPGPNSSYK--SFLDED-KNK--DLKQWALK

NA---IFVDISFP--L--------------------YSN--SNKSQTQNS----------

----------------------------

>Gemini|AGK24653.1

MP--R-SGAFRVHAKNIFLT---YPRC---SL----S-KDEALELLLGVT-TPV--NKKF

IKVA----RELHEDGQ-----PHLHVLLQFEGKLQITNPR-LFDLVSRTFHP--NVQGAK

--SSSDVKSYI---------DK------D-----G-DTVSW-GEFQIDAR---SSRGG--

--KQSANDAYAA-AI-NTG--SPTKALQLLKEQAP-KDYVLQFHNIKGNLERIFMKVSTP

WTCPYDPRSFNNVPDVMLDWVSVNVK-DPAARPNRPISIVVEGDSRTGKTMWARSLGVH-

-------NYLCGHL--------DLSPKVYSNNAWYNVIDDVD-PHYL--KHFKEFMGAQ-

--HDWQSNTKYGKPIQIKGGI-PTIFL-CNPGPSSSYK--SYLDEE-RNS--SLKQWALK

NA---IFVSIAFP--L--------------------YTS--PDQSQTSTSEK--------

---------EAN----------PTTPC-

>Gemini|ABG90906.1

MP--R-QPSFRVSAKNVFLT---YPKC---SL----S-KETALELLKIVN-CPS--DKVF

IRVS----QEKHQDGS-----LHLHALIQFKGKAQFKNPR-HFDLQHPNFHP--NFQGAK

--SSSDVKSYI---------EK------D-----G-DYIDW-GTFQVDGR---SARGG--

--QQTANDAAAE-AL-NAG--SAEAALQIIREKLP-RDFIFQYHNLRSNLDRIFSPPPSV

YSSPFLSSSFNAVPDIISDWAAENVM-DSAARPDRPISIVIEGPSRIGKTVWARSLGPH-

-------NYLCGHL--------DLSPKVYSNSAWYNVIDDVN-PQYL--KHFKEFMGAQ-

--KDWQSNCKYGKPVQIKGGI-PTIFL-CNPGEGSSFK--LWLDKP-EQG--ALKNWATA

NA---IFCDVQSP--F--------------------WVQ--EEVSGAGAITR--------

---------SSE----------EGQEES

>Gemini|ACY79450.1

MA--P-PKRFKIQAKNYFLT---YPHC---SL----S-KEEVLDQLKKIQ-TPV--NKKY

IHIA----RELHEDGQ-----PHIHVLLQFEGKFVCTNQR-LFDLVSPTFHP--NIQGAK

--SSSDVKSYV---------DK------D-----G-DTLTW-GEFQIDGR---SARGG--

--QQTANDAAAE-AL-NSG--SKEAALQIIREKLP-EKFIFQYHNLCSNLDRIFSPPPSV

YSSPFSLSSFNNVPDIISDWAAENVM-DSAARPDRPISIVIEGPSRIGKTVWARSLGPH-

-------NYLCGHL--------DLSPKVYSNSAWYDVIDDVN-PQYL--KHFKEFMGAQ-

--KDWQSNCKYGKPVQIKGGI-PTIFL-CNPGEGSSFK--LWLDKP-EQE--ALKNWAVK

NA---IFCDVDSP--F--------------------WIQ--EEVSTSGANTR--------

---------SGQ----------EEAEED

>Gemini|YP_001040016.1

MP--R-QPGFRVSAKNIFLT---YPKC---SL----S-KEIALELLKAIK-CPS--DKLF

IRVS----QEKHQDGS-----LHLHALIQFKGKAQFKNPR-HFDLQHPTFHP--NFQGAK

--SSSDVKSYI---------EK------D-----G-DYIDW-GTFQVDGR---SARGG--

--QQTANDAAAD-AL-NAG--SKDAALQIIREKLP-EKFIFQYHNLVSNLDRIFQEPPAP

YISPFLCSSFNQVPEELEVWAAENVM-GAAARPWRPISIVIEGDSRTGKTMWARSLGPH-

-------NYLCGHL--------DLSPKVYSNDAWYNVIDDVD-PHYL--KHFKEFMGAQ-

--RDWQSNTKYGKPIQIKGGI-PTIFL-CNPGPTSSYK--EYLDEE-KNT--PLKDWAFK

NA---TFITLHEP--L--------------------FTS--TNQGPTPHSEN--------

---------ESS----------QT----

>Gemini|YP_002224032.1

MP--R-VNAFSVSAKNIFLT---YPKC---PL----S-KETVLDLLRNIS-CPS--DKLF

IRVA----QEKHEDGS-----LHIHALIQFKGKARFRNAR-HFDLIHPHFHP--NIQGAK

--SSSDVKSYI---------EK------D-----G-DFIDW-GFFQIDGR---SARGG--

--QQTANDAAAE-AL-NSG--SAEAALA-ISVKMP-RDYIFQYHNLKSNLERIFTPLRVD

YVSPYPLCSFDRVPEELEIWASENIV-SPAARPFRPISIVLEGDSRIGKTMWARSLGPH-

-------NYLCGHL--------DLSPRVYSNDVWYNVIDDVD-PHYL--KHFKEFMGAQ-

--RDWQSNTKYGKPVQVKGGI-PTIFL-CNPGPHSSYK--EYLDED-RNA--ALKNWAVK

NV---VFDSDSPP--F-------------------------KRRQRRQIRGG--------

----------------------------

>Gemini|ACB44970.1

MA--P-PTRFRINAKNYFLT---YPKC---SL----T-KEEALSQLQNLE-TPT--SKKF

IKIC----RELHEDGS-----PHIHVLIQFEGKFQCKNNR-FFDLVAPSFHP--NIQGAK

--SASDVKTYI---------DK------D-----G-DVLEW-GVFQIDGR---SARGG--

--QQTANDAYAQ-AI-NTG--NKEDALKVLKELAP-KDYVLQFHNLMTNLDRIFPSRIEV

YRSPFNVSSFDRVPPELVDWVSSNLR-CSAARPFRPIGLVLEGDSRTGKTMWARSLGPH-

-------NYLCGHL--------DLNPRVYSNDALYNVIDDVD-PHYL--KHFKEFMGAQ-

--RDWQSNTKYGKPIMIKGGI-PTIFL-CNKGPQSSYK--EFLDEE-KNA--ALKQWALK

NA---VFITLEGP--L--------------------YSG--RENVAPQEEEE--------

---------EHP----------QETS--

>Gemini|AGJ03640.1

MP--R-TPSFCVNAKNIFLT---YPKC---PI----P-KEQMLEILQSIN-CPS--DKLF

IRVA----QEKHQDGS-----LHVHALIQFKGKSKFRNPR-HFDVTSPIFHP--NIQGAK

--SASDVKAYI---------DK------D-----G-DVLEW-GVFQIDGR---SARGG--

--QQTANDAYAK-AI-NTG--NKEDALKVLKELAP-KDYVLQFHNLISNLDRIFQPRSEV

YVSPFSISSFDRVPPELVDWAGVNVV-CAAARPFRPISIVIEGDSRTGKTMWARCLGPH-

-------NYLCGHL--------DLSPKVFSNDAWYNVIDDVD-PHYL--KHFKEFMGAQ-

--RDWQSNTKYGKPVMIKGGI-PTIFL-CNKGPNSSYK--EYLDEE-KNA--ALKQWAIK

NA---VFITLEEP--L--------------------YSG--RENIAPTEEEE--------

---------EHS----------QEAS--

>Gemini|CBJ17676.1

MP--R-INSFCVNAKNIFLT---YPKC---PI----P-KEQMLEILQSIN-CPS--DKLF

IRVA----QEKHQDGS-----LHIHALIQFKGKAKFRNPR-HFDVTHPNFHP--NFQGAK

--SSSDVKSYI---------EK------D-----S-DYIDW-GQFQIDGR---SARGG--

--QQTANDAAAE-AL-NAG--SADAALAIIREKLP-KDFIFQYHNLKCNLDRIFTPPVEA

YVSPFSSSSFDQVPQELEEWAAENVV-SAAARPLRPISIVIEGDSRTGKTMWARSLGPH-

-------NYLCGHL--------DLSPKVYNNDAWYNVIDDVD-PHYL--KHFKEFMGAQ-

--RDWQSNTKYGKPVQIKGGI-PTIFL-CNPGPNSSYK--EFLDEE-KNA--ALKNWALK

NA---TFITLEGP--L--------------------YSG--SNQSAAQDSQE--------

---------GDQ----------ASTS--

>Gemini|AMK07575.1

MP--R-HHSFCVNAKNIFLT---YPQC---SL----S-KQIVLEILQNIS-CPS--DKLF

IRVS----SEKHQDGS-----LHIHALIQFKGKAKFRNER-HFDISHPHFHP--NFQGAR

--SSSDVKAYI---------EK------D-----G-DYVDW-GSFQIDGR---SSRRS--

--SHAAHDVAAE-AL-NAP--SKEDALNIIKEKLP-KDFIFQYHNLSANLDKIFKPQQQQ

YVSPFNVTTFNNVPNELSQWVYQNVV-DAAARPWRPMSIVIEGVSRTGKTLWARSLGKH-

-------NYLCGHL--------DLSPKVYSNDGWYNVIDDVD-PHYL--KHFKEFMGAQ-

--RDWQSNTKYGKPIQIKGGI-PTIFL-CNPGPSSSYK--EYLDEE-KNS--ALKEWALK

NA---TFITLREP--L--------------------FSG--TSENPTQDCQE--------

---------EDN----------SPEAN-

>Gemini|AFA26437.2

MA--P-PKRFLIYAKNYFLT---YPQC---SL----T-KEEALSQFQNLS-TPT--NKKF

IKIC----RELHEDGS-----PHLHVLIQFEGKYKCQNNR-FFDLVSPTFHP--NIQGAK

--SSSDVKSYM---------EK------D-----G-DTLDW-GEFQIDGR---SARGG--

--QQSANDAYAA-AI-NTG--SKSEALRVIKEIAP-KDYVLQFHNLNANLDRIFTPPVEV

YVSPFCSSSFDQVPDELEEWAAENVV-GSAARPLRPISIVIEGDSRTGKTMWARSLGPH-

-------NYLCRHL--------DLSPKVYNNDAWYNVIDDVD-PHYL--KHFKEFMGAQ-

--RDWQSNTKYGKPVQIKGGI-PTIFL-CNPGPNSSYK--EFLDDE-KHS--ALKNWALK

NA---TFITLEGP--L--------------------YSG--SNQSAAQASQE--------

---------GDQ----------ASES--

>Gemini|CAJ85998.1

MP--Q-PSRFKINAKNYFLT---YPDC---SL----A-KEETLEKIKALD-TPT--NKKY

IKIC----RELHQNGN-----PHLHVLIQFEGKYQCKNQR-FFDLVSPTFHP--NIQGAK

--SSSDVKSYI---------NK------H-----G-DTLEW-REFQIDGR---SARGG--

--QQSANDAYAQ-AL-NTG--SKSEALNVIRELAP-KHFVLQFHNLNANLDRIFAPPLEV

FISPFSSSSFDQVPKELEEWTAENVV-SAAARPWRPKSIVIEGESRTGKTMWARCLGPH-

-------NYLCGHL--------DLSPKVYSNDAWYNVIDDVD-PHYL--KHFKEFMGAQ-

--RDWQSNTKYGKPIQIKGGI-PTIFL-CNPGPNSSYK--EYLDED-RNA--ALKAWTLK

NA---EFFTLTGP--L--------------------YSG--TNQSPTPHSEE--------

---------EIN----------SQEEN-

>Gemini|AGF41094.1

MA--T-PKRFKIQAKNYFIT---YPKC---SL----T-KEEALSQIQNIQ-TPT--NKKY

IKIC----RELHEDGS-----PHLHVLIQFEGKFVCTNNR-FFDLVSPTFHP--NIQGAK

--SSSDVKSYI---------DK------D-----G-DTMEW-GEFQIDGR---SARGG--

--QQSANDTAAK-AL-NSG--SAEAALAIIREELP-KDFIFQYHNIKNNLDRIFAPPLEV

FISPFPSSSFDQVPEELEEWAAENVV-SAAARPWRPKSIVIEGESRTGKTMWARSLGPH-

-------NYLCGHL--------DLSPKVYSNDAWYNVIDDVD-PHYL--KHFKEFMGAQ-

--RDWQSNTKYGKPIQIKGGI-PTIFL-CNPGPNSSYK--EYLDEE-KNA--ALKNWTLK

NA---EFFTLTGP--L--------------------YSG--TNQSPTPHREE--------

---------EIN----------SQEEN-

>Gemini|AHL29198.1

MP--P-PSRFRINAKNYFLT---YPQC---SL----T-KEEALSQLQNLA-TPT--NKKY

IKVC----RELHDDGS-----PHLHVLLQFEGKYQCKNPR-FFDLVSPTFHP--NIQGAK

--SSSDVKSYI---------DK------D-----G-DIVEW-GEFQIDGR---SARGG--

--QQSANDAYAT-AL-NTG--SKSEALNVIRELAP-KDYVLQFHNLNANLDRIFAPPLEV

FVCPFLSSSFDQVPEELECWAADNVR-DAAARPWRPISIVVEGASRTGKTMWARSLGPH-

-------NYLCGHL--------DLSPKVYSNDAWYNVIDDVD-PHYL--KHFKEFMGAQ-

--RDWQSNTKYGKPIQIKGGI-PTIFL-CNPGPTSSYK--EYLDEE-KNS--ALRDWALK

NA---EFFTLTEP--L--------------------YSG--THQSPTQNSQE--------

---------EAH----------SEASR-

>Gemini|YP_764516.1

MP--R-AGRFSIKAKNYFLT---FPKC---SL----S-KEDALGQLKDLQ-TPT--NKKY

IKIC----RELHENGE-----PHLHVLIQFEGKYNCTNQR-FFDLVSPSFHP--NIQGAK

--SSSDVKSYI---------DK------D-----G-DTLEW-GEFQVDGR---SARGG--

--CQTANDAAAE-AL-NAG--SADAAMAIIREKLP-KDYIFQYHNLKSNLDRIFQAPPEV

YVSPFSSSSFNNVPEELEEWAAENVV-GAAARPWRPQSIVVEGDSRTGKTMWARSLGPH-

-------NYLCGHI--------DLSPRVYSNEAWYNVIDDVD-PHYL--KHFKEFMGAQ-

--RDWQSNTKYGKPIQIKGGI-PTIFL-CNPGPTASYK--EYLEED-KNA--ALKAWAIK

NA---TFITLSEP--L--------------------YST--TDQSPAPHSQE--------

---------EGS----------EA----

>Gemini|AGG08895.1

MA--P-PKRFKIQARNYFLT---YPHC---SL----T-KEEALSQLKNIE-TPT--NKKF

IKIC----RELHEDGS-----PHLHVLVQFEGKFVCTNNR-FFDLVSPTFHP--NIQGXK

--SSSDVKSYM---------EK------D-----G-DTIDW-GEFQIDGR---SARGG--

--CQNANDACAE-AL-NAG--SKEAALSIIREKLP-KDYIFQFHNLNSNLDRIFTPPLEV

YISPFSSSSFDQVPEELECWVSENVM-DAAARPWRPNSIVIEGDSRTGKTMWARSLGPH-

-------NYLCGHL--------DLSPKVYSNEAWYNVIDDVD-PHFL--KHFKEFMGAQ-

--RDWQSNTKYGKPTQIKGGI-PTIFL-CNPGPTSSYK--EYLDEE-KNS--ALKAWALK

NA---TFVTLEEP--L--------------------YSG--TNQSATPCREE--------

---------ESN----------SQTED-

>Gemini|CDW92215.1

MA--R--ASFRINAKHYFLT---FPKC---SL----T-KEEALEQLLKLD-TPT--NKKY

IKIC----KELHENGE-----PHLHVLLQFEGKFNCQNQR-FFDLVSPTFHP--NVQGAK

--SSSDVESYL---------DK------D-----G-DILEW-GEFQIDGR---SARGG--

--QQTANDAYAT-AL-NTG--SKQEALNVLRELAP-RDYTLQFHNLNSNLDRIFQAPPEV

YISSFSCSSFTQVPDELEEWVADNVR-DSAARPWRPKSIVLEGDSRTGKTVWARSLGPH-

-------NYLCGHL--------DLSPKVYSNSAWYNVIDDVD-PHYL--KHFKELMGAQ-

--RDWQSNTKYGKPVQIKGGI-PSIFL-CNPGQTSSYK--EFLDEE-KNK--ALKAWAVK

NA---TFVTLTEP--L--------------------FSN--ANQGSPQTGQE--------

---------ETD----------QA----

>Gemini|NP_620741.1

MP--R-SGRFSIKAKNYFLT---YPKC---DL----T-KENALSQITNLQ-TPT--NKLF

IKIC----RELHENGE-----PHLHILIQFEGKYNCTNQR-FFDLVSPTFHP--NIQGAK

--SSSDVKSYI---------DK------D-----G-DVLEW-GTFQIDGR---SARGG--

--QQTANDAYAK-AI-NAG--SKSQALDVIKELAP-RDYVLHFHNINSNLDKVFQVPPAP

YVSPFLSSSFDQVPDELEHWVSENVM-DAAARPWRPVSIVIEGDSRTGKTTWARSLGPH-

-------NYLCGHL--------DLSQKVYSNNAWYNVIDDVD-PHYL--KHFKEFMGAQ-

--RDWQSNTKYGKPIQIKGGI-PTIFL-CNPGPQSSFK--EYLDEE-KNQ--ALKNWATK

NA---IFVTIHQP--L--------------------FAD--TNQNTTSHRQE--------

---------EAS----------EA----

>Gemini|YP_006905839.1

MP--NAPKKFQINCKNLFLT---YPQC---SL----S-KEETLSQLTNLS-LPS--NPKF

IRIC----RELHQNGE-----PHLHVLVQFEGKVRLTNCR-LFDLVSPTFHP--NIQGAK

--SSSDVKAYL---------EK------D-----G-DVLDW-GQFQIDRR---SARGG--

--KQSINDAYAQ-AL-NAG--SKSEALRLIKELAP-KDYVLQFHNLNSNLERIFTPPVEI

YRSPFMSSSFDQVPDELEQWVADNVK-AAAARPWRPKSIVIEGDSRTGKTMWARSLGPH-

-------NYLCGHL--------DISPKVYSNDAWYNIIDDVD-PHYL--KHFKEFMGAQ-

--RDWQSNTKYGKPIQIKGGI-PTIFL-CNPGPTSSYK--EFLEEE-KNS--ALKRWTLQ

NA---TFITISEP--L--------------------YSG--TNQSQAQNRED--------

---------QEN----------TS----

>Gemini|BAF02752.1

MP--P-PRRFKLQSKNYFLT---YPKC---SL----T-KEEALEQLKSLS-TPV--NKLF

VKIC----RELHEDGS-----PHLHVLVQFEGKYVCTNNR-FFDLVSPAFHP--NIQGAK

--SSSDVKAYM---------DK------D-----G-DSIEW-GEFQIDGR---SARGG--

--QHAVNEVYAQ-AL-NSG--SKSDALQLIKELAP-KDYVLQYHNLSVNFDKIFTTPVDT

FVSPYPSSSFDQVPEELRQWAGENVK-DAAARPWRPISIVIEGESRTGKTMWARSLGPH-

-------NYLCGHL--------DLSPKVYNNDSWYNVIDDVD-PHYL--KHFKEFMGAQ-

--RNWQSNTKYGKPIQIKGGI-PTIFL-CNPGPASSYK--EYLAED-KNT--ALRSWALK

NA---TFVTINEP--L--------------------YSN--STEDTAQTCEE--------

---------ETN----------PQETN-

>Gemini|AFF58888.1

MA--P-PRRFRVSAKNYFLT---YPHC---SV----T-KDETLSQLRTIN-TQV--NKKY

IKVC----RELHEDGS-----PHLHVLIQFEGKFVCTNNR-LFDLVSPTFHP--NIQGAK

--SSSDVKSYI---------DK------D-----G-DTLEW-GEFQIDGR---SARGG--

--QQSANDAYAQ-AL-NTG--SKQDALRIIRELAP-KDFILQFHNLSSNLDRIFAPEVPV

YTSPFLSSSFNRVPEELEVWVSENVK-SAAARPLRPKSIVIEGDSRTGKTMWARSLGPH-

-------NYLCGHL--------DLSPKVYSNDAWYNVIDDVD-PHYL--KHFKEFMGAQ-

--RDWQSNTKYGKPVQIKGGI-PTIFL-CNPGPGASYI--EFLNEE-KQT--ALKNWALK

NA---EFVSLTEP--L--------------------FSS--PNQGPTQDFQE--------

---------EAN----------TTTEG-

>Gemini|AAX39336.1

MR----TPRFRIQAKNVFLT---YPKC---SI----S-KEHLLPFIQTLS-LPS--NPKF

IKIC----RELHQNGE-----PHLHALIQFEGKITLTNNR-LFDCVHPSFHP--NIQGAK

--SSSDVKSYL---------DK------D-----G-DTVEW-GQFQIDGR---SARGG--

--QQSANDAYAK-AL-NSG--SKSEALNVIRELVP-KDFVLQFHNLNSNLDRIFQEPPAP

YVSPFLCSSFDQVPVEIEEWVADNVI-DSAARPWRPNSIVIEGDSRTGKTIWARSLGPH-

-------NYLCGHL--------DLSPKVYNNAAWYNVIDDVD-PHYL--KHFKEFIGAQ-

--RDWQSNTKYGKPVQIKGGI-PTIFL-CNPGPTSSYK--EFLDEE-KQE--ALKAWALK

NA---IFVTLTEP--L--------------------YSG--SHQSQSQTIQE--------

---------ASH----------PA----

>Gemini|FM877473

MR----TPRFRVQAKNVFLT---YPKC---SI----P-KEHLLSFIQTLS-LPS--NPKF

IKIC----RELHQNGE-----PHLHALIQFEGKITITNNR-LFDCVHPSFHP--NIQGAK

--SSSDVKSYL---------DK------D-----G-DTVEW-GQFQIDGR---SARGG--

--QQSANDAYAK-AL-NSG--SKSEALNVIRELVP-KDFVLQFHNLNSNLDRIFQEPPAP

YVSPFPCSSFDQVPDEIEEWVADNVR-DSAARPWRPNSIVIEGDSRTGKTIWARSLGPH-

-------NYLCGHL--------DLSPKVFNNDAWYNVIDDVD-PHYL--KHFKEFMGSQ-

--RDWQSNTKYGKPVQIKGGI-PTIFL-CNPGPTSSYK--EFLDEE-KQA--ALKTWALK

NA---IFITLTEP--L--------------------YSG--SNQSQPQTIQE--------

---------ASH----------PT----

>Gemini|AGV02071.1

MP--A-PNRFKINAKNYFLT---YPKC---SL----T-KEEALSQFLNLE-TPT--SKKF

IRIC----RELMKMGL-----LHLHVLIQFEGKFQCRNNR-FFDLTSPSFHP--NIQGAK

--SSSDVKAYM---------EK------D-----G-DILDH-GVFQVDGR---SARGG--

--CHTANDAYAE-AI-NSG--SKAQALNILKEKAP-RDFLLQFHNLNSNLDRFFTPPVEV

FKSRYLSSSFDQVPEELEEWAAENVM-DAAARPERPLSLVLEGESRTGKTQWARSLGPH-

-------NYLCGHL--------DLSPKEYSNDAWFNIIDDVD-PHYL--KHFKEFMGAQ-

--RDWQSNTKYGKPVQIKGGI-PTIFL-CNPGPNASYK--EFLDED-KNS--ALKSWALK

NA---TFVFLTQP--L--------------------YSG--TNQSSTQGGEE--------

---------SAQ----------EETSGP

>Gemini|AAF75542.1

MP--Q-LKKFIINAKNYFLT---YPQC---SL----T-KEEALSQISALS-TPT--NKLF

IRIC----RELHEDGS-----PHLHVLIQFEGKFKCQNNR-FFDLTSPSFHP--NIQGAK

--SSTDVKAYM---------EK------D-----G-DVLDH-GVFQIDGR---SARGG--

--CQSANDAYAE-AI-NSG--SKAAALNILKEKAP-KDFVLQFHNLNSNLDRIFTPPIEK

TFLLFYLLLSTKFQKNLKNGLLANVV-SAVGGPLRPMSIVIEGDSRTGKTMWARSLGPH-

-------NYLCGHL--------DLSPKVYNNDAWYNVIDDVD-PHYL--KHFKEFMGAQ-

--RDWQSNTKYGKPVQIKGGI-PTIFL-CNPGPNSSYK--EFLDEE-KNK--ALKNWSLK

NA---IFVTLESP--L--------------------YTG--SNQSEAQASQG--------

---------GEQ----------ASTC--

>Gemini|ACV60535.1

MA--P-PNKFRINAKNYFLT---YPHC---SL----T-KKEALSQLKNLE-TPV--NKLF

IRIC----REFHEDGT-----PHLHVLIQFEGKFQCKNQR-FFDLISPSFHP--NIQAAK

--SSTDVKSYM---------DK------D-----G-DVLDH-GLFQIDGR---SARGG--

--CQSANDAYAE-AI-NSG--SKASALNILREKAP-KDYVLQFHNLNNNLDRIL-PSMEV

YVSPFSSSSFDRVPEELEEWAAENVV-SAVAGPLRPIIIVIEGDSRTGKTMWARSLGPH-

-------NYLGGHL--------DLEPKVYNNDAWYNVIDDVD-PHYL--KPFKEFMGGQ-

--RDWQSNTKYGKPVQIKGGN-PTIFL-CNPGPNSSYK--DYLDED-KNS--ALKYWALK

NA---IFVTLQGA--L--------------------YSG--SYQGATPHRQE--------

---------SNE----------ETKS--

>Gemini|NP_050017.1

MA--P-PKNLRINAKNYFLT---YPHC---SL----T-KEEALSQIQAIE-TPT--IKLF

IRIC----RELHEDGT-----PHLHILIQFEGKFQCKNPR-FFDLTSPTFHP--NIQGAK

--SSTDVKEYI---------AK------N-----G-DVLDH-GVFQIDGR---SARGG--

--CQSANDAYAE-AL-NSG--TKASALAILKEEAP-KDYILQFHNLNSNLDKIFSLRIEM

YISPF-LVSFDQVQRNLNEWVAANVV-KP-ARPLRPISIVVEGDSRTGKTMWARSLGPH-

-------NYLCGHL--------DLSPKVYNNDPWYNVIDDVD-PHYL--KHFKEFMGAQ-

--RDWQSNTKYGKPVQIKGGI-PTIFL-CNPGPHSSYK--EFLEEE-KNT--ALKNWALK

NA---IFVTIEGP--L--------------------FSG--SHQGATQNRQE--------

---------DN-----------------

>Gemini|AFB81519.1

MP----PKRNGFYSKNYFIT---YPKC---SL----T-KEEALSQLLNIQ-TPT--SKKY

IRIC----RELHEDGT-----PHLHVLIQFEGKFKCQNMR-FFDLVSPSFHP--NIQGAK

--SSSDVKSYI---------EK------D-----G-DILDW-GQFQIDGR---SARGG--

--QQTANDAYAA-AL-KGG--NKSEALRVIKELAP-KDFVLQFHNLNENLERIFQGPPAP

YGSLFSSSFFEQIQKKLEGGVAENVV-SAVGRPIRPISLVVEGDSRTGKTMWARSLGPH-

-------NYLGGHL--------DLSPRVYSNDAWFNLFDDAD-PHYL--KHFKEFMGAQ-

--KDWQSNTKYGKPVQIKGGI-PTIFL-CNPGPNSSYK--EFLDEE-KNS--ALKNWALK

NA---IFITLDRP--M--------------------FSG--TNQSTAQGSEE--------

---------AQQ----------EEESRS

>Gemini|ACI06063.1

MP----PRRNGIYSKNYFVT---YPKC---SL----T-KEEALSQLLNLQ-TPT--SKKY

IKIC----RELHEDGT-----PHLHVLIQFEGKFKCQNMR-FFDLVSPNFHP--NIQGAK

--SSSDVKSYI---------DK------D-----G-DILEW-GKFQIDGR---SARGG--

--QQTANDAYAA-AL-GKG--VRSEASQFGNELDR-KDFVLQYHNANANQDRIGQDTPVP

YPFPFSSSQSNQGTEEREESAKENVV-DAAARPLGPQSISTEGDSRTGKTMWCMSLGPH-

-------NYLCGHL--------DLSPKVYSNDAWYNVIDGVD-PHFL--KHFKEFMGAQ-

--RDWQSNTKYGKPVQIKGGI-PTIFL-CNPGPISSYK--EFLEEE-KNT--ALKNWAVK

NA---IFVTLEGP--L--------------------YSG--TNQSTAQGSEE--------

---------TQQ----------EEESRS

>Gemini|AFB83419.1

MA--P-PKRFKVQAILYVIT---YPQC---SL----T-KEEALSQIQAIN-TPS--KKKY

IKLC----RELHEDGS-----PHLPVLIRFEGKFVCTNNK-FFDLVSPNFHP--NIQGAK

--SSSDVKAYI---------NK------D-----G-DAFEW-GEFQIDGR---SPRGG--

--QQTANGAYAA-AL-NAG--SKSEALRVLKELAP-KIFGLQFHNLNANLTRIFREAPAP

YIFPFSPSSFDQVPEELGIWAIDNVV-DAAARPLRPRSIVIEGDSRTGKTMWARSLGPH-

-------NYLCGHL--------DLSPKIYSNDAWYNVIDDVD-PHFL--KHFKEFMGAQ-

--RDWQSNTKYGKPVQIKGGI-PTIFL-CNPGTNSSYK--EFLNEE-KNT--ALKNWALK

NA---IFITLEGP--L--------------------YSA--SNQSTAQGSEE--------

---------TQQ----------EEESRY

>Gemini|AFH68197.1

MP--P-AKPFKINAKNYFIT---YPKC---SL----T-KEDALSQLQNLE-TPV--NKKY

IRIC----REFHENGE-----PHLHVLIQFEGKYQCKNNR-FFDLVSPTFHP--NIQGAK

--SSSDVKSYI---------AK------D-----G-DILEW-GEFQIDGR---SARGG--

--QQTANDAYAA-AL-NAG--SKSEALRVIKELAP-KDFVLQFHNLNANLDRIFQEPPAP

YVSPFSSSSFDQVPEELEEWACENVV-DAAARPHRPQSIVIEGDSRTGKTMWARSLGPH-

-------NYLCGHL--------DLSPKVYSNDAWYNVIDDVD-PHFL--KHFKEFMRAQ-

--RNWQSKAMYWKSVQINGRM-LPIYL-SNPGPNSSYK--RILDDG-KNP--ALKIWTVQ

NA---IFVTFEGT--L--------------------YSG--SNQGTAQGSEE--------

---------TQQ----------GEESRS

>Gemini|ABD35287.1

MR--Q-PGQFRLNRKNFFLT---YSQC---PI----S-KEEALQQLINTQ-TPV--NKKF

IRVC----KELHEDGN-----PHLHALVQFEGKFCLTNPR-FFDLQSPNYHC--FITDAK

--SSSDVKAYI---------EK------D-----G-DILDW-GTFQIDGR---SARGG--

--QQTANDAYAR-AI-NTG--NKEQALDVVRELAP-KDYVLQFHNLNANLERIFMPPPQE

YISPFNCSSFDQVPEEIEEWAADNVM-SAAARPWRPISIVIEGDSRTGKTMWARSLGPH-

-------NYLCGHI--------DLSPRVYSNEAWYNIIDDVD-PHYV--KHFKEFMGAQ-

--RDWQSNTKYGKPVQIKGGI-PTIFL-CNPGPTSSYK--EFLDEE-KNI--GLKNWALK

NA---TFVTINQP--L--------------------YSG--SHQSPTQASQE--------

---------ETS----------QTEG--

>Gemini|AEY63664.1

M---R-PPRFRIQAKNIFLT---YPRC---SL----S-KEELLSFLVGLS-LPS--NLKY

VKVC----RELHQNGE-----PHLHVLLQFDGKITITDNR-LFDHVHPSFHP--NIQSAK

--SSTDVKSYL---------DK------D-----G-DTVEW-GKFQIDGR---SARGG--

--QQTANDAYAT-AL-NMS--NKGEAMSVIKELAP-KDFVLHYHNIKSNLDRIFEEPVAP

FVCPFPISSFTLLPPELVEWASTNVC-SSAARPWRPKSIVVEGESRTGKTMWARSLGPH-

-------NYLCGHL--------DLSPKIYSNDAWYNVIDDVD-PHYL--KHFKEFMGAQ-

--RDWQSNTKYGKPIQIKGGI-PTIFL-CNPGPSSSYK--EFLEEE-KNY--ALKEWADK

NA---EFVFLSEP--L--------------------FSA--EHQDQTQTRQK--------

---------EDD----------SATTS-

>Gemini|YP_003622552.1

MP--R-SGYFCVKAKNIFLT---YPRC---SL----T-KEEALSQLQSIQ-CPS--NKKF

IKIA----RELHENGE-----PHLHVLIQFEGKCQITNER-HFDLTSPRFHP--NIQGAK

--SSSDVKTYI---------DK------D-----G-DTIQW-GSFQIDGR---SARGG--

--CKNANDACAS-AL-NAG--SAEAALQIIKEQLP-RDYVFQYHNVISNLNKIFTPPTTI

YKSPFKVEQFNNVPEVLSQWASDNVK-ASAARPMRPISIVLEGESRTGKTMWARSLGRH-

-------NYLCGHL--------DLSAKVYSNDAWYNVIDDVD-PHYL--KHMKEFMGSQ-

--RDWQSNVKYGKPTQIKGGI-PTIFL-CNPGPRSSYK--EYMDEE-SNA--ALKEWALK

NA---IFYTLEEP--L--------------------FST--EHQGST-------------

----------------------------

>Gemini|ADW24243.1

MS--R-PKGFRVNAKNFFLT---YPRC---SL----S-KEAALEQLQNIQ-TNV--NKKF

IRVC----REFHENGE-----PHLHVLLQFEGKFQCRNER-FFGLVSETFHP--NIQGAK

--SSSDVKKYM---------EK------D-----G-DVIDF-GIFQIDGR---SNRGG--

--SQCANDAYAE-AI-NSG--DTTSALNILKEKAS-RDFIIHLHNIRANLNFLFAPPPTV

YETPFSIESFNNVPETLTSWAAENVV-CPAARPFRPISIVVEGESRTGKTMWARSLGRH-

-------NYLCGHL--------DLSAKVYSNDAWYNVIDDVD-PHYL--KHFKEFMGAQ-

--KDWQSNVKYGKPTQIKGGI-PTIFL-CNAGPRSSYK--MFLDEE-NNA--SLKEWALK

NA---IFYTLTEP--L--------------------FST--TNQGATQVGQE--------

---------TCN----------STQTN-

>Gemini|AKS48121.1

MP--T-PGRFVINAKNYFLT---YPRC---PL----S-KEEALSQLLALQ-TPT--NKKF

IRVS----RELHDDGT-----PHLHALLQFEGKFQTRNQR-FFDLVSQTYHP--NIQAAK

--SASDVKQYV---------EK------D-----G-DFIDH-GAFQVDGR---SARGG--

--KQSANDAYAE-AI-NAE--SKSEALTILKEKAP-KDYVLQFHNINCNLDRLFAPRVPV

YSSIYSIASFNNVPEGLRAWAATNVK-DTAARPDRPISVVIEGDSRTGKTMWARALGRH-

-------NYLCGHL--------DLSAKVYSNDAWYNVIDDVD-PHYL--KHFKEFMGAQ-

--KDWQSNVKYGKPTHIKRGI-PTIFL-CNPGPRSSYK--EYLDEA-NNA--SLKVWALK

NA---EFYTLQAP--L--------------------FSS--VDQGSTQVRQE--------

---------EEG----------QSNSPN

>Gemini|YP_009129272.1

MP--N-TRRFKVQAKNYFLT---YPRC---SL----S-KEEALEQILGLN-TPT--NKKF

VRVC----RELHEDGE-----PHLHVLIQFEGKYTCTNQR-FFDLVSPTFHP--NVQGAK

--SASDVKTYI---------EK------G-----G-EFLDH-GVFQIDAR---SARGQ--

--GQHLAEVYAE-AL-NAP--DASASLQIIKEKDP-KTFYTQYHNLSANAAKIFMAPPEP

FRCIYLSSSFNNVPEELDDWVSVNIV-DPAARPRRPKSIFIEGETRTGKTEWARSLGPH-

-------NYLCGHL--------DLSPKVFSNDALYNVIDDVD-PHYL--KHFKEFCGSQ-

--RDWQSNTKYGKPLLVKGGI-PTIFL-CNPGPTSSYK--EWLDEE-KNS--NLKNWALG

NA---VFVFLSAP--L--------------------FDP--TTSSP--------------

----------------------------

>Gemini|CAM91896.1

MP--R-AGRFKINAKNYFIT---YPQC---SI----T-KEEALAQIKSFS-YPT--NIKF

IRVC----RELHQDGL-----PHLHVLIQFEGKFQCTNQR-FFDLVSQTFHP--NIQGAK

--SSSDVKAYI---------EK------G-----G-EFLDY-GVFQVDAR---SARGE--

--GQHLAQVYAD-AL-NAS--SKEEALQIIKEKDP-KSFFLQYHNLSANADKIFMTPPNP

YVSNFLTSSFDQVPEELEIWVSENVM-DAAARPWRPMSIVLEGDSRTGKTMWARSLGPH-

-------NYLCGHL--------DLSPKVYSNDAWYNVIDDVD-PHYL--KHFKEFMGAQ-

--RDWQSNTKYGKPIQIKGGI-PTIFL-CNPGPTSSYK--EYLEED-KNA--PLKAWATK

NA---TFVTLHEP--L--------------------FSN--TNQGQTPHSEE--------

---------DTH----------ST----

>Gemini|AJM13604.1

MP--R-AGRFNINAKNYFLT---YPQC---SL----S-KEETLSLLLAKQ-IPV--NIKF

IRVC----RELHQNGE-----PHLHVLLQFEGKYQCTNQR-FFDLVSPNFHP--NIQGAK

--SSSDVKTYI---------EK------G-----G-DFIDH-GEFQIDAR---SARGQ--

--GQNLADLYAD-AL-NAS--NKEAAMRLIKERDP-KSYFLQYHNISANAEKIFAPKRVP

YESPYTPASFTNVPEELKLWVAENVM-GPAARPQRPKSIVIEGSSRTGKTMWARQLGPH-

-------NYLCGHL--------DLSNKVYSNNAWYNVIDDVD-PHYL--KHFKEFMGAQ-

--HNWQSNIKYGKPVQIKGGI-PTIFL-CNPGPQSSYK--EFLDEP-KNA--GLKQWALK

NA---TFITISEP--L--------------------YSC--ANQSSAQAGQE--------

---------EET----------T-----

>Gemini|YP_004958233.1

MP--R-VGRFKINAKNYFLT---YPQC---SI----S-KEEALQKLISLR-TPT--NIKF

IRVC----RELHQDGE-----PHLHVLIQFEGKYQCTNQR-FFDLESPTYHP--NIQGAK

--SSSDVKSYI---------EK------G-----G-DFIDH-GQFQVDPR---SARGE--

--GQCLADVYAE-AL-NAA--DKDSALQVIKEKDP-KNFFLQYHNISANANHIFAPKITP

YVSPYDPNSFDNVPEAMKEWASKNVM-GPAARPDRPLSIVIEGPSRSGKTKWARALGPH-

-------NYMCGHI--------DLSLKVYNNNAWYNVIDDVD-PHYL--KHFKEFMGAQ-

--HNWQSNVKYSKPVQIKGGI-PTIFL-CNPGPQSSYK--EYLEEP-KNA--QLKIWAEQ

NA---SFIYLEAP--L--------------------YTS--TNQSEPQTGEE--------

---------EAP----------EAQNN-

>Gemini|AIY31184.1

M-----PRLFKIYAKNYFLT---YPNC---SL----C-QEEALSQLKNLE-TPT--NKKY

IKLC----RELHENGE-----RHLHVLIQFEGKFQCKNQR-FFDLVSPNFHP--NIQAGK

--SSTDVKTYV---------DQ------D-----G-DLIDF-GVFQINSR---SARGG--

--QQSANDAYAE-AL-NSG--SKSEVLNILKENAP-NDYILQFHYLSSNLDRIFSPHLEV

YISPFLSSSFNQVPVELDEWVAENVV-STAARPWTPISIVIEGDSRTAKTMWARSLGPL-

-------NYLCGHL--------DLSPKVYRNDVWYNVIEDVD-PHYL--KHFMVFMGAQ-

--RDWQSNRKYGKPIQIKGGI-PSIFL-CNPRPTSTYR--EYLDEE-KNI--SLKNWALK

NA---IFVTLYDP--L--------------------FAS--INQGPRQDSQE--------

---------ETN----------KA----

>Gemini|AHA82274.1

MV--S-PGRVRENAKKYFLT---DPKC---SL----T-KGEALCQLQTRE-TQG--QKKF

LSIC----RELHVDGF-----RHIQVVIQFEGKFHCKNNR-CLELVSPSFHP--DIQGAK

--SSSDVKSYM---------EK------D-----G-DVLEW-GVFQVDGR---SARGG--

--CQSANDPYAE-AI-NSG--SKSQALNILREKSP-TYFVLQFHNLNTNLDRIFQPPSEV

YVSPFSISSFDRVPADLVDWVTSNVV-CAAARPFRPISIVIEGDSRTGKTMWARCLGPH-

-------NYLCGHF--------DLRPKVYSNEAWYNVIDDVD-PHYL--KHFKEIMGAQ-

--RDWQSNTKYGKPVQIKGGN-PTTFL-CNPGPNSSYK--EYLDEE-KNS--ALKTWALK

NA---TFVTLEGP--L--------------------YSG--TNQSTAQASQE--------

---------GDQ----------TSTS--

>Gemini|YP_001333687.1

M-----SGRFKKQGVSFFLT---WPKC---PV----T-KESALDQIQALT-LPT--NIVY

IRVC----EEKHQDGS-----PHLHALVQFQKKFICTNCR-LFDLSHPQFHC--HIETAR

--SSSDAKSYI---------EK------D-----G-VFCEW-GTFQVDGR---SARGG--

--QQTVNEAYAK-AL-NSG--SKDEALNIIKELVP-KDYVLQFHNLNQNLERIFAPPVNV

FEPPFPLSSFNNVPAVINQWVNDNIM-DAAARPFRPISIIIEGPSRTGKTLWARSLGRH-

-------NYLCGHL--------DLSPKVYSNEAWYNVIDDVD-PHYL--KHMKEFMGAQ-

--RDWQSNCKYGKPIQINGGI-PTIFL-CNPGPTSSYK--EFFEEE-KNK--AINDWAKK

NV---IYVTIEEP--F--------------------FNT--TNQESTSALEE--------

---------SNS----------SETN--

>Gemini|YP_115511.1

M-----GSRFVKTASSFFLT---WPRC---PI----N-KESALEQIKTLS-LPT--NIVY

IRVC----EEKHQDGS-----PHLHALVQFQKKYRCTNCR-LFDLSNPNYHC--NIQTAR

--SSSDAKSYI---------EK------D-----G-VFCEW-GEFKIDGR---SSRGG--

--QQSANDAYAK-AL-NSG--GKDEALTIIKELLP-KDYVLQYHNLNANLERIFAPPTTV

YMPPFQTTTFNNVPEALTDWVTTNVA-DSAARPFRPISIIIEGPSRTGKTLWARSLGPH-

-------NYLCGHL--------DLSPKVYSNNAWYNVIDDVD-PHFL--KHMKEFMGAQ-

--RDWQSNCKYGKPIQIKGGI-PTIFL-CNPGPQSSYK--EFFEEE-KNK--AINDWAKK

NS---IYITITEP--L--------------------YST--VDQASTSSRQE--------

---------SGS----------SETY--

>Gemini|YP_001285764.1

MA--P-NNRFRVSARNYFLT---YPHC---SL----T-KEETLSQLSNLV-CPT--NKKF

IKIC----RELHEDGS-----PHLHVLIQFEGKYVCTNKR-FFDLVSPTFHP--NIQGAK

--SSSDVKSYI---------DK------D-----G-DTLEW-GEFQIDGR---SARGG--

--QQDLNDLGAE-VW-NAT--SVNAAKQLVKEKQP-WTYLLQRHNIVANIEKEFEKPPEP

FVCPFPTTAFDNVPTMMKVWAQEFVT-NSAARPLRPKGIVIEGESRTGKTLWARSLGPH-

-------NYLCGHL--------DLNPNVFSNDAWYNVIDDVD-PHYL--KHYKEFMGSQ-

--TNWQSNRKYGKPVQIKGGI-PTIFL-CNPGPTSSYT--EFLEEE-KNA--KLKRWSNY

NA---IFITLNEP--L--------------------YST--EYQGPAPRGEE--------

---------EDH----------QETED-

>Gemini|AMP46444.1

MP--R-AGRFSVNAKNYFLT---YPNC---PL----D-KNEALSQLQAKQ-TPV--NKLF

IKVC----RELHESGE-----PHLHALVQFQGKYNCTNQR-FFDLESPTYHP--NIQRAK

--SSSDVKSYI---------DK------D-----G-DTTSW-GEYQIDGR---SARGG--

--KQDLNDLGAE-AL-NTG--SAEGAIKIIKERQP-WTFILQRHNIVSNLEREFIKPLEP

YVSPFHISTFTNVPTIIQDWASQNVK-SPAARPLRPQSIVIEGDSRTGKTVWARSLGPH-

-------NYLCGHL--------DLNNKVYSNDAWYNIIDDVD-PHYL--KHFKEFMGAQ-

--QNWQSNTKYGKPIQIKGGI-PTIFL-CNPGPTSSYK--EYLDEA-KNA--SLKLWAIK

NA---TFITLTEP--L--------------------FSP--NTTSETQRSQE--------

---------ENY----------QTTEDR

>Gemini|YP_008411025.1

MP--R-SGRFSINAKNYFLT---YPRC---SL----T-KEETIEQLRNLQ-YPT--NLKY

IKVC----RELHENGE-----PHLHVLLQFEGKYNCTSDR-FFDLSHPSFHP--NIQRAK

--SSSDVKAYI---------GK------D-----G-DTLDW-GEFQVDDE---ICKRR--

--PEICKRCIRL-SN-NAG--SKQEALNIIKELMP-KDYVLQYHNLHSNFDRIFTSAIPP

YKSPFLSSSFDQVPDELEEWVSENVL-PATARPERPISIVVEGDSRTGKTLWARSLGPH-

-------NYLCGHL--------DLSPRVFSNDAWYNVIDDVD-PHYL--KHFKEFMGAQ-

--RDWQSNTKYGKPIQVRGGI-PTIFL-CNPGPTSSYT--EFLDEE-KNK--SLKAWAIK

NA---RFITLFQP--L--------------------FSG--TNQSPTQPGQE--------

---------EDL----------PQEEN-

>Gemini|AAP73446.1

MR----APGFRISARNIFLT---YPKC---SL----S-KEEALEQLCRIE-CPS--DKLF

IRVA----QEAHQDGT-----MHLHALVQFKGKAQFRNAR-HFDLTHPHFHG--NVQGAK

--SSSDVKSYI---------TK------N-----G-DYIDW-GTFQIDGR---SARGG--

--RQTADDAVAS-AL-NSG--TVQGALNIIKELLP-HNYVFQYHNLRCNLERIFAPPIAV

YTSYYKPADFSQVPSVMTDWAENNVV-DPAARPRRPISIVVEGATRTGKTLWARSLGVH-

-------NYMCGHL--------DLSPKIFSNDAWYNVIDDVD-PHYL--KHFKEFMGAQ-

--MDWQSNIKYGKPTQIKGGI-PTIFL-CNPGPRSSYK--EFLDEE-QNE--SLKEWAYK

NA---VFVTLEQP--L--------------------FST--EHQGTASAGEE--------

---------AQN----------DSA---

>Gemini|NP_671468.1

MP--SRPKRFKINAKNFFLT---YPQC---SL----S-KEETLSQIKALN-TPT--SKKY

IKIA----RELHENGQ-----PHIHVLIQFEGKYQCTNYR-FFDLVSPSFHP--NVQGAK

--SSSDVKSYI---------DK------D-----G-DTLEW-GEFQVDGR---SARGG--

--CQTVNDSYAK-AL-NAS--SAEEALKIIREEQP-AHFFLQHHNLVVNASKIFQKPPEP

WVPTFQLSSFTNVPAEMQDWADQYFG-SAAARSERYMSIIVEGDSRTGKTMWARALGPH-

-------NYLSGHL--------DFNSQVYSNEVEYNVIDDIN-PNYLKLKHWKELIGAQ-

--KDWQSNCKYGKPVQIKGGI-PSIVL-CNPGEGSSYK--EFLDKE-ENR--ALHNWTIK

NA---LFVTLTAP--L--------------------YQS--TP-----------------

----------------------------

>Gemini|AMW86999.1

MP--R-KGSFLYKAKNYFIT---YRVL---SC----S-KEEALSQLQNLN-TPV--NKKF

IKIC----REFHQNGK-----PHLHVLIQFEGKFNCTNYR-LFDLVSPTFHP--NIQGAR

--SSSDVKSYV---------AK------D-----G-DTIEW-GVFQIDGR---SARGG--

--QQTANDAAAE-AL-NSG--TKEEAMKIIKEKLP-EKFLFQYHNLSSNLDRIFSKAPEP

WTPPFPLSSFTAVPDEMQQWADEYFG-GAAARPERPVSIIVEGDSRTGKTMWARSLGPH-

-------NYLSGHL--------DFNARVFSNEVEYNVIDDVA-PQYLKLKHWKELLGAQ-

--KDWQSNCKYGKPVQIKGGI-PSIVL-CNPGEGASYK--DFLDKE-ENS--ALRNWTNK

NA---KFITLTAP--L--------------------YQE--GTLASQEEGNQ--------

---------EAQ----------D-----

>Gemini|AGH29892.1

MP--P-PKRFKISSKNYFVT---YPHC---SL----T-KEEALAQLKLLN-TPT--NKKY

IKIC----RELHENGE-----PHLHVLIQFEGKYQCTNNR-FFDLVSPSFHP--NIQGAK

--SSSDVKSYI---------DK------D-----G-DTIEW-GEFQVDGR---SARGG--

--QQTSNDAAAE-AL-NAS--SKEEAMMIIKEKLP-EKFLFQYHNLSSNLDRIFKKAPDP

WVPPFPLSSFTNVPDEMQEWADEYFG-GAAARPVRPMSIIVEGDSRTGKTMWARALGSH-

-------NYLSGHL--------DFNSRVYSNDVEYNVIDDVT-PHYLKLKHWKELIGAQ-

--KDWQSNCKYGKPVQIKGGI-PSIVL-CNPGEGASYK--DFLEKE-ENA--SLKSWTLY

NA---KFIFLDSP--L--------------------YQT--ATQDCEEESNP--------

---------AAT----------D-----

>Gemini|CBH28932.1

MP--SKPPRFRVQAKNIFLT---YPQC---SL----T-KEEALSQLQAIH-LPS--NKKF

IKIC----RELHEDGQ-----PHLHILLQLEGKIQVTNNR-LFDLVSPNFHP--NIQGAK

--SSSDVKSYI---------DK------D-----G-DTVEW-GEFQVDGR---SARGG--

--QQTANDAAAD-AL-NAP--DKQTALRIIREKLP-EKYLFQFHNLNSNLDRIFSKAPEP

WVPPFPISSFINVPEEMQEWADDYFG-SSAARPLRPMSLIVEGDSRTGKTMWARALGPH-

-------NYLSGHM--------DFNSRVFSNEVEYNVIDDVT-PQYLKMKHWKELIGAQ-

--RDWQSNCKYGKPVQIKGGI-PSIVL-CNPGEEVSYK--EFLDKE-ENA--ALKSWTLH

NA---KFIFIDSP--L--------------------YQT--TSQSGEEERNS--------

---------P------------------

>Gemini|CBA18089.1

MP--P-PKRFKVNAKNYFLT---YPDC---SI----A-KETALEQLINLE-TPS--KKKY

IRVC----REFHENGK-----PHLHALVQFEGKFQCTNCR-FFDLKHPNAHA--NIQGAK

--SSSDVKSYI---------KK------D-----G-DYIDW-GTFQIDGR---SARGG--

--QQTANDAASE-AL-NSS--SKEEAMQIIKEKLP-EKFLFQYHNLSTNLDKLFKKAPEP

WVPPFQLSTFTNVPHEMQEWADDYFG-VVAARPDRPISLIVEGDSRTGKTMWARALGPH-

-------NYLSGHL--------DFNSRVFSNEVEYNVIDDVS-PHYLKLKHWKELIGAQ-

--RDWQSNCKYGKPVQIKGGI-PSIVL-CNPGEGASYK--EFLDKQ-ENA--ALRSWTLH

NA---KFIFLDSP--L--------------------YQT--TAQDRQEESDQ--------

---------TTA----------S-----

>Gemini|FJ665283

MP--P-PKRFKINAKNYFLT---YPQC---SI----T-KESAIEQLQNLQ-TPV--NKKY

IRIC----REIHENGE-----PHLHALIQFEGKFQCTNCR-VFDLKHPTSHA--NIQSAK

--SSSDVKSYI---------EK------D-----G-DYIEW-GHFQVDGR---SARGG--

--QQTANDAASE-AL-NAS--SKEEAMQIIKEKLP-EKFLFQYHNLSSNLDRIFTKA--P

WSPPFHLSSFTNVPREMQEWADDYFG-GAAARPERPISIIVEGDSRTGKTMWARALGTH-

-------NYLSGHL--------DFNSKVFSNHAEYNVIDDIA-PHYLKLKHWKELMGAQ-

--KDWQSNCKYGKPVQIKGGI-PSIVL-CNPGEGASYK--CFLDKE-ENA--ALKHWTIH

NA---KFIFLDSP--L--------------------YQS--STQSCEETSNQ--------

---------TTS----------R-----

>Gemini|ALV85583.1

MP--P-PKRFKVNAKNYFLT---YPRC---SI----S-KEEALSQLLALE-TPT--NKKY

IRVC----RELHEDGA-----PHLHALLQFEGKFQCTNCR-FFDLRHPQCHG--EYKSCK

--SSSDAKSYI---------EK------G-----G-DYIEW-GTFQIDGR---SARGG--

--QQTANDAAAE-AL-NAA--SKDEAMQIIKEKLP-EKFLFQYHNLSSNLDRIFSKPPEP

WASPFPLSSFTNVPDEMKDWVAQYIG-DAAARPVRPVSLIIEGDSRTGKTMWARALGPH-

-------NYLSGHL--------DFNSRVYSNEVEFNVIDDVS-PHYLKLKHWKELIGAQ-

--RDWQSNCKYGKPVQIKGGV-PSIVL-CNPGEGASYK--DFLDKA-ENS--ALKAWTLH

NA---TFVFLDSP--L--------------------YQT--STQGGETENNS--------

---------S------------------

>Gemini|AFD54490.1

MP--P-PRRFKIQSKNYFLT---YPKC---SI----S-KEEALAQLLALD-TPT--NKKY

IRVC----RELHGNGE-----PHLHALLQFEGKFTCTNCR-FFDLRHPSCHG--KYESCK

--SSSDAKSYI---------EK------D-----G-DYVEW-GDFQIDGR---SARGG--

--QQTVNDTYAK-AL-NAS--SVEEALQIIKEEQP-QHFFLQHHNVVANAYRIFQKAPEP

WTPPFPLSSFINVPEEMKAWADDYFG-SAAARPERPISIIIEGDSRTGKTMWARALGSH-

-------NYLSGHL--------DFNSKVYSNDVQYNVIDDIA-PQYLKLKHWKELIGAQ-

--KDWQSNCKYGKPVQIKGGI-PCIVL-CNPGEGASYK--SFLDKE-ENA--SLRQWTIH

NA---QFVFLNSP--L--------------------YQS--STYSGQEESNQ--------

---------EKT----------N-----

>Gemini|YP_002941855.1

MP--SEPRRFRVNCRNFFLT---YPKC---SL----S-KEEALSQLLALE-TPT--NKKF

IRVS----RELHEDGQ-----PHLHVLIQFEGKYNCQNNR-FFDLVSTTFHP--NIQGAK

--SSSDVKTYV---------EK------D-----G-DFIDH-GIFQIDGR---SARGG--

--QQSANDTYAK-VL-NSG--SVMEALNILREEQP-KDFVLQHHNIRSNLERIFQKAPEP

FVPPFPLSSFTLVPEEMQEWADSYFG-DAAARPERAISIIIEGDSRTGKTMWARSLGVH-

-------NYLSGHL--------DFNSRVYSNDVEYNVIDDIS-PHYLKMKHWKELIGAQ-

--RDWQSNCKYGKPVQIKGGV-PSIVL-CNPGEGSSYK--DFLDKE-ENA--SLRNWTLR

NA---QFVFLNSP--L--------------------YQT--TTQNRQEESG---------

----------------------------

>Gemini|YP_006590064.1

MP--P-PKRFRINAKNYFLT---YPQC---SL----S-KEAALEQIISLQ-TPS--SKKF

IKIA----RELHDDGQ-----PHLHVLLQFEGKFCCTNNR-LFDLVSPTFHP--NIQGAK

--SSSDVKSYV---------DK------D-----G-DTIEW-GEFQVDAR---SARGG--

--QQTANESYAK-VL-NAD--NLERALQILKEEQP-KDFVLHHHNIRSNLERIFAKAPEP

WAPPFHLSSFTLVPQEMQDWVNDYFG-GAAARPERPISIIIEGDSRTGKTMWARALGSH-

-------NYLSGHL--------DFNSRVYSNDVQYNVIDDIA-PQYLKLKHWKELIGAQ-

--KDWQSNCKYGKPVQIKGGV-PCIIL-CNPGEGSSYK--SFLDKE-ENA--GLKGWTLH

NA---KFVFLNSP--L--------------------YQS--STQSS--------------

----------------------------

>Gemini|ACV83312.1

MP--P-PKRFSVNAKNFFLT---YPHC---SL----T-KYEALSQLQALQ-TPT--HKKF

IRVT----RELHEDGE-----PHLHVLIQFEGKFKCHNQR-FFDLVSPTFHP--NIQGAK

--SSSDVKTYM---------EK------D-----G-DFIDF-GTFQIDGR---SARGG--

--CQSANDTYAK-VL-NAD--NPSTALNILKEEQP-RDYVLHLDKIRTHVQRLFAKAPRP

WVSPFQLSSFTNVPDEMQEWADKYFG-GAAARPERPISIIIEGDSRTGKTMWARALGPH-

-------NYLSGHL--------DFNSRVYSNEVDYNVIDDVT-PXYLKLKHWKELVGAQ-

--RDWQSNCKYGKPVQIKGGI-PSIVL-CNPGEGASYK--DFLDKH-ENV--SLKTWTLH

NA---KFIFLNSP--L--------------------YQT--TTQSSQTESDS--------

---------PKT----------H-----

>Gemini|AER09339.1

MP--L-SKPFRINARNYFLT---YPKC---SL----T-KEEALSQLQALA-TPT--NKKF

IKVA----RELHENGE-----PHLHVLLQFEGKFQCKNNR-FFDLVSPSFHP--NVQGAK

--SSSDVKSYV---------DK------D-----G-DTLEW-GTFQIDGR---SARGG--

--CQSANDTYAK-VL-NAE--SAAQALQILREEQP-RDFVLHLDKVQAHVQKIFAKAPEP

WVPPFPLSSFTNVPDEMQSWADEYFA-SAEARPNRPISLIVEGDSRTGKTMWARALGPH-

-------NYLSGHL--------DFNSRVYSNEAAYNVIDDVA-PHYLKLKHWKELVGAQ-

--RDWQSNCKYGKPVQIKGGI-PSIVL-CNPGEGSSYK--DFLDKE-ENS--ALRSWTLH

NA---RFVFLESP--L--------------------YQS--ATQSGETESHS--------

---------P------------------

>Gemini|YP_007250561.1

MP--P-PKKFRLSAKNIFLT---YPRC---SL----T-KEEALFQLQNIS-LPS--NKLF

IRVA----RELHEDGE-----PHLHVLIQLEGKVQIYNQK-LFDLSSTSFHP--NIQGAK

--SSSDVKSYM---------EK------D-----G-EFLDF-GVFQIDGR---SSRGG--

--IQTTNDSYAK-AL-NAG--SAESALQILKEEQP-AHYFLHYHNLVNNANRIFQKAPEP

WVPPFQLSSFNAVPDEMQEWADDYFG-GAAARPERPISIIVEGDSRTGKTMWARSLGPH-

-------NYLSGHL--------DFNSRVYSNEVEYNVIDDVA-PHYLKLKHWKELIGAQ-

--KDWQSNCKYGKPVQIKGGI-PSIVL-CNPGDGGSYK--DFLDKE-ENA--SLKQWTLK

NA---KFIFLNSP--L--------------------YQT--SSQER--------------

----------------------------

>Gemini|YP_619883.1

MP--P-PRRFRLQAKNIFLT---YPLC---SL----T-KDEALEQLQSIQ-LTS--NKRY

IKIC----RELHENGK-----PHLHALVQLEGKVQITNER-QFDLVSPTFHP--NIQGAK

--SSSDVKSYI---------DK------D-----G-DTLEW-GEFQIDGR---SARGG--

--QQTANDSYAK-AL-NAN--GVQEALQILKEEQP-RDFVKDFHNLKGNLEKIFTKAPEP

WVPPFSLSSFNNVPEELQSWADDYFG-GCAARTLRPISIIIEGDSRTGKTMWARSLGIH-

-------NYLSGHL--------DFNSRVYSNDVMYNVIDDVP-PHYLKMKHWKELIGAQ-

--TDWQTNCKYGKPIQIKGGI-PSIVL-CNPGEGASYK--YYLDKQ-ENS--HLKAWTHH

NA---EFVFLDAP--L--------------------YQT---------------------

----------------------------

>Gemini|AHX57826.1

MP--R-RGSFSVNAKNYFLT---YPQC---SL----T-KEDVLSQIQNLQ-TPT--NKKY

IKVC----RELHENGE-----PHLHVLIQFEGKYNCTNNR-FFDLVSPNFHP--NIQGAK

--SSSDVKSYL---------DK------D-----G-DVIEW-GEFQIDGR---SARGG--

--QQTANDTYAK-AL-NAS--TMEESLQIIKEQQP-AHYYLQYHNLVANATRIFRKPPEQ

WIPPFPLSSFNNVPEVLQEWADNYFG-DPAARPLRSKSIIIEGDSRCGKTMWARALGKH-

-------NYLAGHL--------DFNAKCYSDDVDYNVIDDVS-PSYLKLKHWKDLIGAQ-

--TQWQTNCKYGKPIMIKGGV-PSIVL-CNPGEGSSYK--DFLEKE-ENA--SLKRWTLY

NA---EFVFLDSP--L--------------------YQT--STQGSQEEGSP--------

---------A------------------

>Gemini|NP_040557.1

MP--P-TKRFRIQAKNIFLT---YPQC---SL----S-KEEALEQIQRIQ-LSS--NKKY

IKIA----RELHEDGQ-----PHLHVLLQLEGKVQITNIR-LFDLVSPTFHP--NIQRAK

--SSSDVKSYV---------DK------D-----G-DTIEW-GEFQIDGR---SARGG--

--QQTANDSYAK-AL-NAT--SLDQALQILKEEQP-KDYFLQHHNLLNNAQKIFQRPPDP

WTPLFPLSSFTNVPEEMQEWADAYFG-DAAARPLRYNSIIVEGDSRTGKTMWARSLGAH-

-------NYITGHL--------DFSPRTYYDEVEYNVIDDVD-PTYLKMKHWKHLIGAQ-

--KEWQTNLKYGKPRVIKGGI-PCIIL-CNPGPESSYQ--QFLEKP-ENE--ALKSWTLH

NS---TFCKLQGP--L--------------------FNN--QAAASSQGDST--------

---------L------------------

>Gemini|ALF37659.1

MV--RCSTPFYKKAKNIFLT---YPQC---SV----T-KEDALEQLININ-TPS--NKKY

ISIC----RELHENGE-----PHLHALIQFEGKVQIRNPR-YFDMQHRSFHC--NIQGAK

--SSSDVKSYV---------SK------D-----G-DHIDW-GEFQVDGR---SARGG--

--QQTANDAAAE-AL-NAG--NALEALQIIREKLP-EKYIFQYHNLKPNLEAIFLPPPDL

YQPPFPLSSFTRVPEIIQEWADSYFG-DPAARPYRYNSIIIEGDSRTGKTMWARCLGPH-

-------NYITGHL--------DFSLKTYSDNVLYNVFDDVD-PNYLKMKHWKHLIGAQ-

--REWQTNLKYGKPRVIKGGI-PSIIL-CNPGEGSSYQ--DFLNKS-ENE--ALRSWTLQ

NS---VFAKLTSP--L--------------------FDN--NQEASSQDQSS--------

----------------------------

>Gemini|YP_003778178.1

MP--RTPRQYRIHAKNIFLT---YPHC---HL----T-KEDALLQLQTIQ-CPS--TKKF

IRIC----RERHDNGE-----PHLHVLIQFEGKIQLYNPR-HFDLRDGGCHP--NIQGAK

--SSSDVKSYI---------EK------D-----G-DYIDW-GEFQIDAR---SARGG--

--QQTANDACAE-AL-NSG--TAEAALAVIREKLP-KDYIFQFHNLKPNLAAIFNPPPVG

YVPKYNHTQFV-LTDDILDWLESNFF-EESNSPDRPKSIIIEGPSRTGKTLWARSLGSH-

-------NYITGHL--------DFSTKVYNDDVSYNVIDDVD-PHYLKMKHWKHLIGAQ-

--KEWQTNLKYGKPRIIKGGI-PSIIL-CNPGDGASYQ--NFLDKP-ENE--ALKSWSLQ

NS---VFTTIEEP--L--------------------FNI--TSDQEVEDSTP--------

---------TV-----------------

>Gemini|KC108902

MP--RTPRQYRIHAKNIFLT---YPHC---HL----S-KEDALLQLQTIE-CPS--TKKF

IRIS----RELHENGE-----PHLHVLIQFEGKIQLYNPR-HFDLRDRGCHP--NIQGAK

--SSSDVKSYI---------EK------D-----G-DYTDW-GEFQIDPR---SARGG--

--QQTANDACAE-AL-NSG--TAEAALAVIREKLP-KDYIFQFHNLKPNLAAIFNPPPVG

YIPKYNHTQFV-LTDDILDWLESNFF-EESISPDRPKSIIIEGPSRTGKTLWARSLGSH-

-------NYITGHL--------DFSTRIYNDDVTYNVIDDVD-PQYLKMKHWKHLIGAQ-

--KEWQTNLKYGKPRIIKGGI-PAIIL-CNPGEGASYQ--TFLDKP-ENE--ALKSWSLQ

NS---IFTTIEEP--L--------------------FNI--TSDQEVQDSTP--------

---------SL-----------------

>Gemini|YP_009226627.1

MG--RTNKQYRIQAKNIFLT---FPHC---TL----S-KDEALEQLQNLA-CPS--NKKF

IRIS----RELHENGE-----PHLHVLIQFEGKVQICNPR-HFDLRHPSFHP--NIQGAK

--SSSDVKSYI---------EK------D-----G-DYLDW-GQFQIDGR---SARGG--

--QQTVNDASAE-AL-NAA--SAEAALAIIREKLP-HDFLFKYHNLKPNLAAIFAPPPAV

YTPPFTHTQFN-IPEEIRQWVNNNFI-EEASSPERPQSIIIEGPSRTGKTLWARSLGTH-

-------NYITGHL--------DFSARVYHDDVEYNVIDDVD-PHYLKMKHWKHLIGAQ-

--KEWQTNLKYGKPRIIKGGV-PSIIL-CNPGDGASYK--DFLDKS-ENE--ALKSWTIQ

NS---AFTTITEP--L--------------------YDN--ENIEEAEDDPP--------

---------TV-----------------

>Gemini|ALR86823.1

MT--P-PKRFRVAAKNFFLT---YPHC---FL----S-PTEALEQLKQLQ-IPV--NKKY

IRIA----KEHHDDKE-R--TPHLHALLLLEGKFHGQNPR-FFDLVSPNYHC--NIQGAK

--SSSDVKTSS---------QK------E-----G-EYSDW-GEFQIDGR---SSRGG--

--QQSANDAYAA-AI-NTG--SKQEALRVIKELAP-KDYVLQYHNLNANLAAIFAPPPEI

YQPPYTHNQFI-LPQDIQAWVDSNFT-EPATPPERPQSIIIEGPSRTGKTLWARSLGTH-

-------NYITGHL--------DFSARVYHDEVEYNVIDDVD-PHYLKMKHWKHLIGAQ-

--KEWQTNLKYGKPRIIKGGV-PSIIL-CNPGDGASYK--DFLDKP-ENE--TLKSWTIQ

NS---AFATITEP--L--------------------YIN--ENREEAEDSPP--------

---------TV-----------------

>Gemini|ABD67440.1

MP--R-EGRFAINAKNYFLT---YPKC---PL----T-KEDALEQLLALS-TPV--NKKF

IRIC----RELHEDGQ-----PHLHVLLQFEGKQQTKNQR-FFDLHSRCYHP--NIQAAK

--SCSDVKTYM---------EK------D-----G-DILDH-GTFQIDGR---SARGG--

--QQSANDAYAE-AL-NSG--SKLEALLILKEKAP-KDFILQFHNLNCNLSRIFTEPTQA

YESPFTLESFDKVPSYISSWAERNVR-DPAARPDRPISIVIEGDSRTGKTMWARAIGPH-

-------NYLCGHL--------DLNDKTYSNEAWYNVIDDVD-PHYL--KHFKEFMGAQ-

--RDWQSNVKYGKPTKIKGGI-PTIFL-CNPGPKSSYK--STWMKR-TML--RLNSQRMR

NSRIKYYIHINSP--L--------------------FQD--APQSNSSRVQNDGANNVQP

--------VEDS----------TGDAQV

>Gemini|AEE99005.1

YQ--HASAYLKYMPKNYFLT---YPNC---SL----S-KEEALSQLKNLA-TPT--NKKY

IKVC----REFHENGE-----PHLHVLIQFEGKYQCKNQR-FFDLSSPTFHP--NIQAAK

--SATDVKTYV---------EK------D-----G-DFIDF-GVFQIDGR---SARGG--

--QQSANDAYAE-AL-NSG--SKSEALNILKEKAP-KDYILQFHNLSSNLDRIFSPPLQV

YVSPFLLLLLIK-SRELKKGSPEKPV-SSAARPWRPISIVIEGDMQTGKTMWARSLGPH-

-------NYLCGHL--------DLSPKVYSNEAWYNVIDDVD-PHYL--KHFKEFMGAQ-

--RDWQSNTKYGKPIQIKGGI-PTIFL-CNPGPTSPFR--GNLGRR-KKY--ILEKLGSQ

EC------NLRHP--L--------------------RAT--VRKYQSRSNTR--------

----------------------------

>Gemini|APP87725.1

MP--RKPSSFRLNAKNIFLT---YPQC---HI----S-KESALEQLKAFH-YPI--HPVF

IKVS----AESHQDGQ-----PHLHALLQFKGKFQTTNQR-FFDLVSPSFHP--NIQGAK

--SSSDVKSYI---------EK------D-----G-DVISW-GEFQIDGR---SSRGG--

--VQSANDAYAE-AL-NSG--GKDQALQILKEKAP-KDYILHYHHLVGNLGRIFKTPPKE

YTPPFSLDSFNNVPDELWDWVWESGLGNAPAGPLRPKSLVLEGDSRTGKTLWARALGKH-

-------NYLSGHL--------DLNDKVFSLDADYNIIDDVD-PHYL--KHFKEFMGAQ-

--VGWQSNTKYGKPVQVEKTM-PSIFL-CNPGPNSSYK--EFLDEE-KNA--ALKNWALK

NA---IFVSLQEP--L--------------------FKA--QSPRASEDTAQ--------

---------TSQ----------DRQSS-

>Gemini|AGV02076.1

MP--RQPKELRVQSKNIFVT---YPQC---DI----P-KDEVLQMLQNLQWRVV--KPTY

IRVA----REEHSDGN-----PHLHCLVQLSGKPNIKDVR-FFDITHPRFHP--NVQPAK

--NSDAVKNYI---------TK------G-----G-DFCES-GQYKPYG-------GT--

--KANKDDVYRN-AV-NSG--TATEALDIIKDGDP-KTFIVQYHNIRSNLDRIFKKAPEP

WIPPYQLSSFVNVPDDMQEWADSFFG-DHAARPERPISIIIEGDSRTGKTMWARALGPH-

-------NYLSGHL--------DFNSRVYSNNAEYNVIDDIN-PQYLKMKHWKELIGAQ-

--KDWQSNCKYGKPVQIKGGI-PSIVL-CNPGEGSSYK--DFLDKE-ENV--ALKNWTLH

NA---KFIFLNSP--L--------------------YQD--QTQASQEEGHS--------

---------P------------------

>Gemini|AAL96826.1

MP--RNPDSFRLQARNIFLT---YPKC---DI----P-KNEALQMLQALSWSVV--SPTF

IRVA----REEHSDGS-----PHLHCLIQLSGKPNIKNER-FFDLTHPRFHP--NIHAAK

--DANAVKNYI---------TK------D-----G-DCCES-GQFEVPR-------GT--

--KNNKDDVYYN-AI-NAP--SVAEALEIIRAGDP-RAFIVSYHNIKANLDRLFKKAPEQ

WVPPFPLSSFTNVPDEMQEWADGYFG-GPAARPVRPVSLIVEGDSRTGKTMWARALGPH-

-------NYLSGHL--------DFNHRVYSNNVAYNVIDDVS-PHYLKLKHWKELLGAQ-

--KDWQSNCKYGKPVQIKGGI-PSIVL-CNPGEGSSYK--DFLDKA-ENA--SLKNWTIK

NV---IFITLDAP--L--------------------YQE--GTQASQEEGDQ--------

---------TTTN---------------

>Gemini|YP_003828907.1

MP--RSPKSFRVAAKNIFLT---YPQC---DI----P-KDEALKMLQELAWTVV--KPKY

IRVA----REEHSDGS-----PHLHCLIQLSGKSNIKNAR-FFDLTHPRFHP--NIQAAK

--DSNAVKNYI---------TK------E-----G-DYCES-GEYKVSG-------GT--

--KSNKDDVFHN-AI-NAG--TAEEALAIIKAGDP-KTFVVNYHNVKANIERLFQKDEDP

WVPPFQLSSFNNVPQEMKDWVDENVT-ARAAGPVRPRSIIIEGDSRTGKTMWARAIGPH-

-------NYLSGHL--------DFNARVYSNNVMYNVIDDVG-PQYLKLKHWKELIGAQ-

--RDWQSNCKYGKPVKIKGGI-PSIVL-CNPGDGASYK--AFLDKE-ENA--SLRAWTLK

NA---NFVFLNAP--L--------------------YQD--EAQNSQEEGRS--------

----------------------------

>Gemini|NP_066185.1

MP--RQPNSFRIQARNIFLT---YPQC---TI----P-KDEMLQLLQNLNWTVV--KPTY

IRVA----AERHADGT-----PHLHCLIQLSGKSNIKDCR-FFDITHPQFHP--NVQSAK

--DTNAVKNYI---------TK------E-----G-DYCES-GQYKVSG-------GT--

--KANKDAVYHN-AV-NAG--GVREALDIIKAGDP-KTFVTQHHNVKANLEHIFAKPPEP

WNPPYPLSSFTNVPEEMQEWADDYFG-RPVADSHKAKSIIIEGNSRTGKTCWARALGTH-

-------NYLCGHL--------DFNMRVYSNEVEYNVIDDVA-PTYLKMKHWKELIGAQ-

--RDWQSNCKYGKPVQIRGGV-PTIIL-CNPGEGSSFR--DFLDKP-END--GLRQWTLY

NA---IFIFLDTP--L--------------------FDN------G--------------

----------------------------

>Gemini|YP_003966137.1

MP--RQNKSFRLSSKNIFLT---YPQC---DI----P-KDEVLQLLQNLPWSVV--KPTY

IRVA----SERHADGT-----PHLHCLIQLSGKSNIKDCR-FFDITHPRYHP--NVQAAK

--DANAVHNYI---------TK------E-----G-DYCES-GQYKVSG-------SS--

--KANKDDVYHN-AV-NSG--GVKEALEIIKAGDP-KTFVIQHHNVLANLERIFKKPQET

WTPPFPLSSFNNVPDEMQEWADNYFG-PIESRPIRPKSLIIEGDSRTGKTCWARSLGKH-

-------NYLSGHL--------DFNPRVYSNEVEYNVIDDIA-PSYLKLKHWKELIGAQ-

--HDWQSNCKYGKPVQIKGGI-PSIIL-CNPGEGSSFK--DFLDKD-ENE--SLKRWTLY

NA---VFISISEP--L--------------------YNK--DD-----------------

----------------------------

>Gemini|AFM38721.1

MP--RQPNKFRLASRNIFLT---YPQC---SL----D-KNHVLELLQNLPWTIV--RPTY

IRVA----REIHSDGN-----PHLHILIQLSGKSNIKDER-FFDISHPSFHP--NIQAAK

--DTNAVKNYI---------TK------E-----G-DYCES-GQYKVSG-------AT--

--RTNKEDVYHN-AL-HAE--SMAAAIAIIKAGDP-VRWITQGHNIRSNLQFDFAKQKEP

WTPKFPLSSFNNVPEEMQEWADDYFG-DSAARPLRPRSIIIEGDSRTGKTQWARTLGSH-

-------NYISGHL--------DFNSKTYSDDVEYNVIDDVA-PNYLKMKHWKELIGAQ-

--TDWQSNCKYGKPREIKGGL-PAIVL-CNPGEGTSYK--DFLDKE-ENA--PLKRWTLQ

NA---TFIFLDSP--L--------------------YQT--QTQDCQIQGNT--------

---------S------------------

>Gemini|CRI68211.1

MP--LPPKSFRLQCKNIFLT---YPQC---DI----P-KDEALEMLRSLKWSVV--KPMY

IRVS----REEHSDGF-----PHLHCLIQLSGKCNIKDAR-FFDITHPRFHP--NVQAAK

--DSNAVKNYV---------TK------D-----G-DYYEY-GQCKFSG-------GA--

--KHNKDDVYYK-AV-NAA--SAEEALDIIKEDDP-KPFIVSYHNIKANIERIFTTPPKP

WTPPYPLSSFNNVPEDMQQWVAEYFG-SSAARPDRPISIVMEGDSRSGKTMWARALGPH-

-------NYLSGHL--------DFNYKVYSNDVEYNVIDDLT-PQYLKLKHWKELIGAQ-

--RDWQSNCKYGKPVQIKGGI-PSTVL-CNPGEGSRYI--AFVNKE-ENA--SWIAWTQK

NV---HFVILHAP--P--------------------YKS--TAQDC--------------

----------------------------

>Gemini|ACO88014.1

MA--SSSHRFRIQGRAFFLT---YSQC---PR----E-PKDVGEFLTSHSTLAS--HVVY

VRVQ----QEKHQDGN-----NHLHAIVCTSERRDIRDPR-IFDFGE--FHP--KIETCR

--SVSKSLKYI---------QK------E----AG-SFYEH-GTVPCDKR---TGRKR--

--KAEQDEWWHQ-AV-NSG--TIEEALQLVKDNEP-RTFWLQHHNLVTNARRIWSEVRAD

FVPKYSESSF-SVPRVLSDWVANNLR-AD-PLPDRPLSLIIEGDSRTGKTAWARSLGSH-

-------NYLSGHL--------DLNGAVFDNEASYNVIDDVN-PKYL--KHWKEFLGAQ-

--KDWQSNLKYGKPVLVKGGK-PAIVL-CNSDQS--YK--SFLDCE-ENQ--HLRSWTSK

NA---VFVDIQDA--L--------------------FSG--VSLT-REQTRE--------

---------DDPE--------ADSDPGD

>Gemini|FJ665634

MA--SSSHRFRIQGRAFFLT---YSQC---PR----E-PKDVGEFLTSHSTLAS--HVVY

VRVQ----QEKHQDGN-----NHLHAIVCTSERRDIRDPR-IFDFGE--FHP--KIETCR

--SVSKSLKYI---------QK------E----AG-SFYEH-GTVPCDKR---TGRKR--

--KAEQDEWWHQ-AV-NSG--TIEEALQLVKDNEP-RTFWLQHHNLVTNARRIWSEVRAE

FVPKYSENSF-SVPRVLSDWVTNNLR-AD-PLPDRPLSLIIEGDSRTGKTAWARSLGRH-

-------NYLSGHL--------DLNGAVFDNEASYNVIDDVN-PKYL--KHWKEFLGAQ-

--KDWQSNLKYGKPVLVKGGK-PAIVL-CNSDQS--YK--SFLDCE-ENH--QLRSWTSK

NA---LFVDIQDP--L--------------------FRG--VSLT-REQTRE--------

---------DDPE--------ADSDPGD

>Gemini|AFV91331.1

AQ--SGPSHFRIRAQNIFLT---YPRC---DL----D-PKDAGEIIQSKM-QSH--EPKY

ILFS----RELHSDGE-----YHLHGLLQLSRQFSSNNPR-IFDIGA--HHP--NIQSAI

--SPKSVRDYI---------LK------N----PI-TQFCI-GTYVPAKKGRKLGSRF--

--EENIRNNIMR-SISTAT--SKESYLSMVRKSFP-FEWATKLSQFEYSASKLFPEVTPE

YKSPFPTE-SLICNENIQDWVDNTLY-QP-NRTSRGLSLYICGPTRTGKTSWARSLGVH-

-------NYWQNNI--------DFS--VYNDNATYNVIDDIP-FKFC--PCWKALAGSQ-

--SDFTVNPKYGKKKRIKGGI-PCIIL-VNEDE-------DWLTCM-SSS--QKT-YFES

NV---VIYYMYEK--F--------------------FNF--VEE----------------

----------------------------

>Gemini|YP_006666531.1

TE--ASPANFRFRARSAFLT---YPKC---TL----E-PRDVVEHLYSKF-RKY--GPKY

CLVT----REHHSDGD-----YHLHCLFQLDKAFSTNDSS-TFNILD--YHP--NIQTAK

--SPTNVRDYC---------LK------N----PV-SKAER-GTFIPLKG--RTPKNT--

--ESKAKDSVMR-SINTST--DRASYLSMVRKAFP-FDWATKLQQFEYSASKLFPDVIPE

YTSPFPTE-NLMCNERITDWLDNTLY-QSAHPRTRKSGLYICGPNRTGKTSWARSLGKH-

-------NYWQMNL--------DFA--NYNNEAQYNVIDDIP-FKFC--PYWKALVGSQ-

--HEYTVNPKYGKKKLIKGGI-PSIIL-VNEDD-------DWMRAM-NDG--QRS-YFEG

NM---SIYYMSES--F--------------------IR----NEAL--------------

----------------------------

>Gemini|AIT39773.1

NE--GGPSTFRFDASEIFLT---YPRC---PV----D-PMDVGNYLWTML-LTL--SPKY

VLCT----RERHADGS-----FHLHAFVQLGYNLITANPH-HFDYKQ--YHP--NVQPVR

--SSKSVRDYC---------LK------N----PI-SQYSR-GRYTVGRGSRVGESSS--

--DWESKNAKMA-TIATAT--DRASYLGMIKTTFP-FEWATKLQAFEYSASKLFPEVEPE

YETPNWAV-SLRCPEAIADWANHNVF-QP-NDGTRRKSLYICGPTRTGKTTWARSLGTH-

-------NYYNNNI--------DFT--NYRDRAFYNVIDDIP-FKFC--PCWKSLVGCQ-

--KDYIVNPKYGKKKKIAGGI-PSIII-VNEDD-------DWLPKM-TAA--QRS-YFEA

NC---EVHYLYDS--F--------------------FAT--PDQAAPDL-----------

----------------------------

>Gemini|YP_006666527.1

DE--ESVDGFHFRGKNIFLT---YSRC---EI----D-PALITDALWDKF-SSH--KPLY

ILSV----RELHQDSG-----FHVHCLVQLTDQYRSRDSS-FADLGG--NHP--NIQTVR

--SATKVKEYI---------LK------E----PV-SQSAR-GKFVAPGGDRR---RS--

--DSAVKDERMR-YIRTAT--TRDDYLGMVRKSFP-FEWATRLAQFEYSASKLFPDITPQ

YQSQYQTT-DLTCHENLLDWYQENLQ-CYIDGAGRRKSLYICGPTRTGKKSWARVLGRH-

-------NYYNMQV--------DWA--TYDQEAQYNVIDDIP-FKFC--PHWKALIGCQ-

--KDFTVNPKYGKKKLIKGGI-PTIIL-VNEDE-------DWLADM-TPG--QVS-YFEA

NV---QIHYMTES--F--------------------IP----DPALRQRLSLNY------

----------------------------

>Gemini|AAK73446.1

SS--SSNRQFSHRNANTFLT---YPKC---PE----N-PEIACQMIWELV-VRW--IPKY

ILCA----REAHKDGS-----LHLHALLQTEKPVRISDSR-FFDING--FHP--NIQSAK

--SVNRVRDYI---------LK------E----PL-AMFER-GTFIPRKSD--SEVKE--

--KKPSKDEIMR-DISHST--SKEEYLSMIQKELP-YDWSTKLQYFEYSANKLFPEIQEE

FTNPHPQSSDLLCNESINDWLQPNIF-QSSDERSRKQSLYIVGPTRTGKSTWARSLGVH-

-------NYWQNNV--------DWS--SYNEDAIYNIVDDIP-FKFC--PCWKQLVGCQ-

--RDFIVNPKYGKKKKVKKSK-PTIIL-ANSDE-------DWMKEM-TPG--QLE-YFEA

NC---VIYIMSEK--W--------------------YSP--PELPPTEAVHSDR------

----------------------------

>Gemini|AF003952

SS--SSNRQFSHRNANTFLT---YPKC---PE----N-PEIACQMIWELV-VRW--IPKY

ILCA----REAHKDGS-----LHLHALLQPEKPIRISDSR-FFDING--FHP--NIQSAK

--SVNRVRDYI---------LK------E----PL-AVFER-GTFIPRKSD--SEVKE--

--KKPSKDEIMR-DISHAT--SKEEYLSMIQKELP-FDWSTKLQYFEYSANKLFPEIQEE

FTNPHPPSSDLLCNESINDWLQPNIF-QSSDERSRKQSLYIVGPTRTGKSTWARSLGVH-

-------NYWQNNV--------DWS--SYNEDAIYNIVDDIP-FKFC--PCWKQLVGCQ-

--RDFIVNPKYGKKKKVKKSK-PTIIL-ANSDE-------DWMKEM-TPG--QLE-YFEA

NC---VIYIMSEK--W--------------------YSP--PVLPPTEEV----------

----------------------------

>Gemini|AHM88378.1

AT--VATRPFKHRNANTFLT---YSRC---NL----D-PEAVGLHLWELI-GHW--NPAY

ILVS----REAHADGE-----WHLHALAQSVKPVHTTNQG-FFDIEG--FHP--NIQSAK

--SANKVREYI---------LK------N----PV-CQWEK-GTFIPRKQ---SESKN--

--SKPSKDDIVR-DIEHST--SREEYLSMLQKALP-YEWATKLQYLEYSASKLFPDTVEE

YTNPHPPTTLLREPTTIDNWVQSNLF-QN-NTGTRKLSLYILGPTRTGKSTWARSLGRH-

-------NYWQNNV--------DWS--CYDEDSVYNVIDDIP-FKFC--PCWKQLIGCQ-

--KDYVVNPKYGKKRRVKTSI-PSIIL-ANEDE-------DWLKVM-SPG--QLD-YFHQ

NC---VVYIMEER--F--------------------FGG--ELVSATAHPSNEV------

----------------------------

>Gemini|Q80GM6.2

SS--TPSRRFKHRNVNTFLT---YSRC---PL----E-PEAVGLHIWSLI-AHW--TPVY

VLSV----RETHEDGG-----YHIHVLAQSAKPVYTTDSG-FFDIDG--FHP--NIQSAK

--SANKVRAYA---------MK------N----PV-TYWER-GTFIPRKT---TEPNS--

--KKQSKDDIVR-DIEHST--NKQEYLSMIQKALP-YEWATKLQYFEYSANKLFPDIQEI

YTSPFPQSTALLDPTAINTWLENNLY-QQNSNSNRKLSLYILGPTRTGKSSWARSLGRH-

-------NYWQNNV--------DWS--SYDEDAEYNIIDDIP-FKYC--PCWKQLIGCQ-

--KDYIVNPKYGKRKKVSKSI-PTIVL-ANEDE-------DWLRDM-TPA--QQD-YFNA

NC---ETYMLEER--F--------------------FSL--PAVSATAHPSSEV------

----------------------------

>Gemini|AHM88382.1

NS--VASRSFKHRNANTYLT---YPKC---PL----E-PEAIGLTLWSLI-APW--EPAY

IIVC----REAHQDGT-----WHCHALAQSVKPVTTRNSR-FFDIED--HHP--NIQSAK

--SVDKVRAYI---------LK------D----PI-ALWER-GTFIPRKK---GDEHT--

--PKPTKDDIVR-DIEHST--SKQEYLSRLQNELP-YEWATKLQYFEYSANKLFPDIPEP

YIHPHPQTEELHCKETIDDWLKPNIF-QQ-LPSDRKQSLYIVGPTRTGKSTWARSLGRH-

-------NYWQNNV--------DWT--SYDEEAMYNIVDDIP-FKYC--PCWKQLIGCQ-

--KEYIVNPKYGKRKKVSRSI-PTIVL-ANEDE-------DWLKDM-TPA--QRE-YFEA

NC---VIYIMTEK--W--------------------FSP--V------------------

----------------------------

>Gemini|YP_003288768.1

ES--NSSRSFRHRNANTFLT---YSKC---SL----D-PEILGLSLWSKL-APW--TPAY

ILVA----REAHQDGT-----WHCHALAQSVRPVTTSDPR-FFDVNE--YHP--NIQSAK

--SVDRVREYI---------LK------D----PL-CQWEK-GTFVPRKK---GESSN--

--TRASKDDIVR-DIQHST--NKHEYLSMLQKELP-YEWATKLQYFEYSANKLFPEIAEP

YTNPHPPTQDLHCYERIEEWLNFNVY-QVQQEAGRARSLYIVGPTRTGKSTWARSLGRH-

-------NYWQNNV--------DWS--SYDEEAVLNVIDDIP-FKYC--PCWKQLVGCQ-

--KNYVVNPKYGKKKKVKRSI-PAVIL-ANEDE-------DWLRDM-TPA--QRD-YMEA

NC---EVYIMSEK--W--------------------FTP------A--------------

----------------------------

>Gemini|P0C647.1

GR--HSVRCFRHRNANTFLT---YSKC---PL----E-PEFIGEHLFRLT-REY--EPAY

ILVV----RETHTDGT-----WHCHALLQCIKPCTTRDER-YFDIDR--YHG--NIQSAK

--STDKVREYI---------LK------D----PK-DKWEK-GTYIPRKK---KEPAE--

--KKPTKDEVMR-EITHAT--SREEYLSLVQSSLP-YDWATKLNYFEYSASRLFPDIAEP

YTNPHPTTEDLHCNETIEDWLKPNIY-QQNAPGERKRSLYICGPTRTGKTSWARSLGRH-

-------NYWQNNI--------DWS--SYDEEAQYNVVDDIP-FKFC--PCRKRLVGCQ-

--KDYIVNPKYGKRRKVSKSI-PTIIL-ANEDE-------DWLKDM-TPA--HVE-YFEA

NC---DQYILLEK--F--------------------YKT--GEAGGSI------------

----------------------------

>Gemini|AHM88370.1

TP--PPRRNFQFKSANAFLT---YPRC---LL----T-PFEAGQHLWEVA-RPW--TPSY

ILAS----SESHQDGT-----PHLHVLLQTIRPMSTRDPG-FFDIQG--YHP--NIQASR

--SPNKTREYI---------LK------S----PI-TVYSR-GTFIPRAG-GAGYGST--

--PVPKRNEIMR-GIETTT--SKAEYLSEVQKAFP-FEWATKLQQFEYSAERLFPTLPSP

FVPPHPPSEDLNCYETIRSWKDENIF-QGDSRTSRPRSLYIVGPTRTGKTTWARSIDNH-

-------NYWQNGV--------DFL--KYRKSAKYNVLDDIP-FKFC--PCWKQLVGGQ-

--KDYTVNPKYARRMEVPGGI-PSIIL-VNYDE-------DWLKVM-TPA--QLE-YFYD

NC---VVYQMEEK--F--------------------YTP------S--------------

----------------------------

>Gemini|YP_006273070.1

PK--ASSSTFRYRSNNCFLT---FPHC---NS----C-PYGMVQHFWDLI-STW--SPIY

AVAS----VELHQDGT-----PHLHALLQTRKQISTNDPH-FFDFDG--HHP--HIQAAK

--NPTLCRDYI---------LK------G----PI-TFSEK-GAFIPRGR-GTSPRHS--

--NKRSRDDIMK-DIENST--NKSDYLSKVRRNFP-YDWATKLYNFEYSASKLFPEQQPE

YSNPHGQSVDLYCYETIQDWIDSNLF-QVHYCYMLNTPTYIVGPTRTGKSTWARSLGRH-

-------NYWQNNV--------DFT--VYDPEAAYNIIDDIP-FKFC--PCWKQLVAAQ-

--RDFTVNPKYGKKKLIKGGI-PSIIL-VNSDE-------DWLKTM-TPE--QQE-YFEA

NS---IIYMMEEK--F--------------------FGG--AEIV---------------

----------------------------

>Gemini|YP_009026388.1

ST--SQPRVFQFKAQNIFLT---YPRC---DI----S-VDVAARTLLTLC-HRF--QPLY

ILCS----QEHHADGS-----NHLHILLQTDKTMYTRNPH-YFDICG--HHP--NIQPAK

--SPDNVRAYI---------LK------D----PI-TSFEE-GSFQPRGS---IPRSG--

--NSGTKDSLMR-DINTST--SKDDYLTRVRNTFP-FDWATRLQQFEYSASKLFPEPVRE

YVNPFPPSEDLFCREIIDRWVDMDIT-DAFDAAQRRRSLYIVGPTRTGKSTWARSLGRH-

-------NYWQHMV--------DFT--AYDTLAKYNILDDVP-FKFC--PNWKQLVGCQ-

--RDFIVNPKYAKRKEIPGGI-PCIIL-QNPDD-------DWLPVL-SPS--QMD-YFVN

NC---DVYVMKEK--F--------------------FGG--DTPVPEAQEDV--------

---------PDG----------TGSS--

>Gemini|YP_003915159.1

AA--SLPLSFNVRSQHVFLT---YPRC---PI----P-PKDAGSFLKKLC-KRY--NIQY

MYIA----QELHQDGE-----PHLHAFLQFDKVFRTTSAK-YFDFFE--FHP--NIQAAR

--NPEKTLEYC---------QK------N----PA-DFYED-GVFVKPKA--SRKRKL--

--ASFTRDKKMK-QIANAT--SRDEYLSMIRKAFP-FDWAIRLQQFEYSAKALFPEAPIQ

YQPQFVSN-DMSDHPVIGEWLDTEFF-TQEGPHHRRRSLYICGPTRTGKTSWARSLGTH-

-------HYWQHSV--------DFLT-EWNKNAIYNVIDDIP-FKFV--PCWKGLVGSQ-

--FDITVNPKYGKKKTIPNGI-PSIIL-ANEDE-------DWLQTM-SPQ--QAD-WFHG

NC---VVYYLQES--F--------------------IPP--SSDVA--------------

----------------------------

>Gemini|AFN80669.1

VT--PGEKAFSLRTKHVFLT---YARC---PI----S-PEEAGQKIADRL-KNK--KCNY

IYIS----REFHADGE-----PHLHAFVQLEANFRTTSPK-YFDLGE--FHP--NIQAAR

--QPASTLKYC---------MK------H----PE-SSWEF-GKFLKPKV---NRSPT--

--QSASRDKTMK-QIANAT--NRDEYLSMVRKSFP-FEWAVRLQQFQYSANALFPDPPQT

YSAPYASR-DMSDHPVIGEWLQQELY-TVRSPGVRRRSLYICGPIRTGKTTWARSLGTH-

-------HYWQHSV--------NFLE-EWNCQAQFNIIDDIP-FKFV--PCWKGLVGSQ-

--YDLTVNPKYGKKKRIPNGI-PCIIL-VNEDE-------DWLTSM-STQ--QVD-WFHG

NA---VVYHLLET--F--------------------IPS------E--------------

----------------------------

>Gemini|AFN80601.1

DA--PSSSCFKVRARNLFLT---YSKC---NL----T-AVFLLEYISSLL-KKY--CPTY

IYVA----QEAHKDGS-----HHLHCIIQCSKYVRTTSAK-FFDVGE--FHP--NVQNPR

--MPKKALAYC---------KK------S----PI-SDAEY-GVFQEIKR-PRKKKVD--

--APSTKDAKMA-DIKSST--NKEDYLSMVKKSFP-FDWATRLQQFQYSAESLFPSTPPP

YVDPFGMP-SQDEHPVIGAWLRDELY-TD-SPAERRRSLYICGPIRTGKTSWARSLGSH-

-------NYWQHSV--------DFL--HVVQNAKYNVIDDIP-FKFV--PCWKGLVGSQ-

--KDITVNPKYGKKRLLSNGI-PCIIL-VNEDE-------DWLQQM-QPN--QAE-WFNA

NA---VVHYMYES--F--------------------FEA--GPATA--------------

----------------------------

>Gemini|YP_004089627.1

PG--AVAACFEVRSRNIFLT---YSKC---HL----D-PVFMQEHLSSLL-RRF--EPTY

VYVA----REEHQDGS-----YHLHCLVQCKKYVRTKSAK-FFDVEE--FHP--NVQNAR

--MPHKVLAYI---------KK------N----PL-CFVET-GVFQASTK-QKKKKVD--

--APSTKDAKMA-EIKSST--CKEDYLSMVRNTFP-FDWATRLQQFQYSAESLFPSVPTP

YMDPFGMP-AQDEHPVIGAWLQAELF-SR-RPDERRRSLYICGPTRTGKTSWARSLGAH-

-------NYWQHSV--------DFL--NLVANATYNVIDDIP-FKFV--PCWKGLVGCQ-

--FDITVNPKYGKRRMLKNGV-PSIIL-VNEDE-------DWLKQM-QPS--QVG-WFET

NC---IIHYMYES--F--------------------FEA---------------------

----------------------------

>Gemini|YP_006666523.1

PG--AVEACFEVRSRNVFLT---YSKC---HL----E-PAYMLERLSRLL-KKW--DPTY

TYVA----REEHKDGS-----YHLHCLVQCRKYIRTKSAK-FFDVEE--FHP--NVQNAR

--VPHKVLAYI---------KK------G----PV-CFVEH-GAFKDEAK-KKKRKAD--

--APSTKDAKMA-SISQST--SREDYLGMVKKEFP-FDWATRLQQFEYSAQALFPCLPPP

YVDPFGMP-SQAEHQVLGAWLREELY-SQDSPAERRRSLYICGPTRTGKTSWARSLGCH-

-------NYWQHSV--------DFL--HVIPTARYNVIDDIP-FKFV--PCWKGLVGAQ-

--RDITVNPKYGKKRLLPNGI-PSIIL-VNEDE-------DWPQYM-QPS--QAA-WFED

NC---VVFYMNFR--F--------------------FET---TA----------------

----------------------------

>Gemini|YP_006666535.1

PG--AAEASFEVRAKNIFLT---YSKC---LL----D-PQEALRDITHKL-RKF--EPTY

VYVA----RELHQDGT-----FHLHCFVQCKKHVRTTRAR-FFDLEE--YHP--NVQNAR

--MPHKVLAYC---------KK------S----PV-SYAEE-GAYTESDV--RKRKID--

--ASTTKDAKMA-DIRSSK--SKEEYLSMVRKTFP-FDWATRLQNFEYSAERLFPSTPPP

YVSPFNMP-SQEEHPVLGAWLRAELY-TQGNPAERRKSLYICGPTRTGKTSWARSLGKH-

-------NYWQHSV--------DFL--NIIPDAEYNVIDDIP-FKFV--PCWKGLVGAQ-

--RDITVNPKYGKKRLLSNGV-PCIIL-ANEDE-------DWLQQM-QPG--QAD-WFNA

NC---EVHYMYET--F--------------------FKS-LGAATA--------------

----------------------------

>Gemini|YP_009021763.1

EH--GGPSGFRFQSRNIFLT---YPRC---NL----A-PELIGSFLLSLL-SPY--HVMF

ITVT----SELHKDGT-----PHIHALAQTDKRVHTYSPG-FFDVQG--FHP--NIQSAR

--SPQTVLSYI---------LK------S----PT-GTFNY-GSLRPRGTSRELANDP--

--GRDRKDVLMT-SIAGSS--SKQEFLNGVKKAFP-YDFCARLQNWEYAANKLF-DTPAV

YQPPFPDS-YFHCHENIHDWVRDNIY-EI-TPEARPLSLYICGPTRTGKSTWARSLGRH-

-------NYWQNNV--------DFT--SYDVEAKYNVIDDIP-FKYC--PCWKALIGGQ-

--KDFTVNPKYGKKKLIKGGI-PSIVI-VNDDE-------DWMRAM-TAS--QRS-YFER

NC---VVVYLYDS--F--------------------IKD--DVSSTSEECI---------

----------------------------

>Gemini|DQ458791

MP--SASKNFRLQSKYVFLT---YPKC---SS----Q-RDDLFQFLWEKL-TPF--LIFF

LGVA----SELHQDGT-----THYHALLQLDKKPCIRDPS-FFDFEG--NHP--NIQPAR

--NSKQVLDYI---------SK------D----G--DIKTR-GDFRDHKV----------

--SPRKSDARWR-TIQTAT--SKEEYLDMIKEEFP-HEWATKLQWLEYSANKLFPPQPEP

YVSPFTES-DLRCHEDLHSWRETHLY-HDNRNGIRHPSLYICGPTRTGKTTWARSLGRH-

-------NYWNGTI--------DFT--NYDEHATYNIIDDIP-FKFV--PLWKQLIGCQ-

--SDFTVNPKYGKKKKIKGGI-PSIIL-CNPDE-------DWMLSM-TSQ--QKD-YFKD

NC---VTHYMCET--F--------------------FAR--ESSSH--------------

----------------------------

>Gemini|KT214373

MP--RRASNFRLQGKSIFLT---YPQC---PL----I-PMFLIDYLYQLL-KNW--DPTY

ARVC----REEHQSRE-----PHLHCLVQRDKKIDIKDPR-FFDIKDPNYHP--NTQIPR

--RDADVADYI---------SK------G-----G-VFEER-GLLRASRR-------S--

--PKKSRDSIWT-TITEST--SKSEFLSRCRTEQP-YTYATQLRNLEYMASREWPEPPTV

YQPRW--THFPSVPESIKQWADENIF-TD-QKPDRPLTLIIEGPSKTGKTAWARSLGRH-

-------NYFCGGV--------DFS--FWDNHASYNVIDDIP-FQFL--PCKKELLGSQ-

--RDFTVNEKYRKKTRVPGGI-PTIVL-CNPDQS--YK--TAL----SSS--DMYEW---

--------E---------------------------------------------------

----------------------------

>Gemini|JX094280

MP--RNNNSFRLQGKSIFLT---YPKC---PL----T-PLFVIDYLYQLL-KNF--NPIY

ARVC----TENHQDGE-----PHLHCLVQLDKRFNTTSQR-YFDISDPNYHP--NCQVPR

--RDADVADYI---------AK------G-----G-QFEER-GILRASRR-------S--

--PKKSRDSIWT-NINEST--SKSEFLGRVQIEQP-YVWATQLRNLEYAANSKWPEQPSV

YIPKW--TVFNNVPEPIREWADTN--------------LFTVSPTKTGKTAWARSMGLH-

-------NYFCGGV--------DFS--VWNNFATYTVIDDIP-FQFL--PCKKELLGCQ-

--KDFTVNEKYRKKCRI-----PTIVL-CNPDQS--YK--AAL----MGS--EMYEWSLS

NV---IHVEIKDP--F--------------------F-----------------------

----------------------------

>Genomo|KM598389

MP-----SNFAFDGSHVFLT---YPQC---SL----S-KERVRDFLLVEL------GVRR

FLVA----SELHGDGQ-----PHIHAYAAWDSRRRLVGAG-CFDVDG--HHP--NIQKPR

--SAKAVAEYC---------GK------H-------DTEAL-RNFENAE-----LESN--

--RGNTG--WRS-ILRECP--DAKTFLARVEEHYP-RDLCLSLDRLLQFCEWRWGTERIG

YSGRTRELFL--EPDQLREWVRLS-L-EV-LMPERPISLLLVGASKLGKTEWARSLGPH-

-------MYFCGQF--------NLDDWN--DEAKYIVLDDFN-IKFF--PQWKSFFGAQ-

--KQFVLTDKYRKKRTVKWGR-PLIWL-CNADPRGAL----------SGA--ELE-WLQI

NA---MMVDLFTP--L--------------------F------TQ---------------

----------------------------

>Genomo|KJ547627

MV------------YTFSFT---YPQC---SL----E-RTELRDSLIQRV------NPEK

YLIA----REQHSDGG-----LHLHAYLHFGRRRRFTSAD-AFDVDG--FHP--NIQKPR

--SARNVIAYC---------SK------E----DTFDYT--SGE--PNG-----------

--------GWSE-ILGRTS--SKDEFLEEVRTHFP-RDYVLSLERLLFFCEWRFGRDETE

YTGRSRTEF--REPDTLTNWVNTNLL---------QVCIWTYGPSSLIWTQWARSISTH-

-------VYCQGMF--------NLD--EWNDKAKYVIFDDID-IKYF--PHWKSILGCQ-

--RDIQLTDKYRKKRRLRNGL-PCVWL-CNMDPRGAL----------SRT--QC--WIES

NC---DVVTLREP--L--------------------F--------E--------------

----------------------------

>Genomo|KJ547626

MT--------RFAAKQFFLT---YPRC---DL----D-LQLLLDGLTTAL-APR---L-R

HKIV----QERHGDEG-----LHVHAIIVCAERIDTSNPR-FFDVAG--FHP--NIQTVR

--NLRQAYTYL---------DK------E----PV-QAL--CNLDEPIP-----------

--KM----SWGE-LLEKAT--DAVEFMNLMKKYHP-RDYILSFTRLLDFAEVHFTEPPPL

YETPEGYSF------------------------YRPKSLVLIGPSRTGKTTWARSLGRH-

-------VYWNSLV--------NLD--VWSPLADYIIFDDVD-IDFL--PGYKCWLGAQ-

--KEFTVTDKYKKKRKIMWGK-PCIWL-CIKDPLLS-S--------------KVYRWISM

NC---VFVTVINK--M--------------------Y-----------------------

----------------------------

>Genomo|KJ547634

MP------RFQARAKAFIVT---FPQV---SE----D-VQQRFDHEGASLLDLK--DPSC

FRLG----RERHQDGG-----VHYHMYIGFDEVVHINRAN-LFDYFG--AHG--NIKSVR

--TPRTVYDYC---------GK------D-----G-DVRYE-RGDPPES-----VSRS--

--RGENGEKWHT-IC-DAP--DKDTFLSLCRQLAP-KDWLLSNSRILEYANTYYPEIPSP

YEGPPIAEME--RYPELERWLEQA---------ERVKSLILYGPSRTGKTLFARSLGEH-

-------AYFNLQF--------NMDGFS--DGVKYAVFDDIQGFEFW--HSYKGWLGAQ-

--KEFVITDKYRKKRTIKWGK-PTIMC-LNPN---FL----------KGV--DYE-WLQL

NC---VIIEVLDP--I--------------------C------HLSS-------------

----------------------------

>Genomo|YP_009115514.1

MS------SFSFHARYALLT---YAQC---DL----C-PFTIVDLLSTM--------GAE

CIIG----REHHQDGG-----IHLHVFVDFGRKYRSRRAD-TFDVGG--FHP--NISQSG

--TPEKGYDYA---------CK------D-----G-DVVAG-GLDRPVP---TEGRGG--

--DSKTHSIWSQ-IT-SAT--TRESFWDLVHDLDP-KSAVTCFTQLQKYCDWKYRYCPPA

YESPAGARFRNDTSDGRGDWLLQSGI-GG---A-RVKSLVLYGPSQTGKTTWARSLGAH-

-------IYQVGLL--------SGSECMKAPDVEYAVFDDIRGMKFF--PSFKEWLGCQ-

--PHVCVKELYREPRVIEWGK-PAIWC-SNADPRDDM----------SYC--DVQ-WMEA

NC---TFIEITEK--L--------------------L--------DWE------------

----------------------------

>Genomo|YP_003104796.1

M-------TFDFHAKYVLLT---YAQC---EL----D-AFRVMDKLSLL--------GAE

CIIG----REHHEDGG-----THLHCFAEFGRKFRSRKAD-VFDVDG--HHP--NITKSG

--TPEKGYDYA---------IK------D-----G-DVICG-GLGRPSV-----GRVG--

--TRPSDSKWAI-IT-SAS--NRDEFWELVHELDP-KAAATSFSQLQRYCDWKYQYHAPE

YESPAGAHFIGAELDGRNRWLEQSGI-GSS--E-RVKSLVLYGPSQTGKTSWARSLGKH-

-------IYCVGLV--------SGTECLKAPDVEYAVFDDIRGIKFF--HSFKEWLGCQ-

--PHLSVKELYREPKVIEWGK-PSIWC-SNADPRNEL----------LQV--DID-WMEM

NC---TFIEITEP--V--------------------V------EFN--------------

----------------------------

>Genomo|YP_009252368.1

MP-------FHFGARYALIT---YAQC---AL----D-GFRVMDHFSGL--------GAE

CIVG----REVHADGG-----IHLHCFIDFGRKFRSRRTD-IFDVDG--RHP--NIAPSG

--TPWRGYDYA---------IK------D-----G-DVICG-GLERPEE----PRSKR--

--VTKDWDPWTE-IT-NAR--DREHFWELVHHLDP-KAAACNYGQLAKYADWRFAAKPPV

YESPGGIEFVGGDVDGRDAWCDQSGI-RSG--P-RCMSLVLYGDSRVGKTLWARSLGAH-

-------VYTVGMV--------SGGELKKVDTVKYAVFDDIRGIKFF--PAFKEWLGAQ-

--AYVTIKELYREPALVRWGR-PSVWI-SNDDPRLVM----------EAS--DVS-WLEA

NC---IFVEITEA--I--------------------F------RAKR-------------

----------------------------

>Genomo|YP_009021043.1

MP-------FYFNARYALIT---YAQC---AL----D-GFRVMDHFSGL--------GAE

CIIA----REIHQDGG-----VHLHCFIDFGRKFRSRRTD-IFDVDG--RHP--NIEPSG

--TPWRGYDYA---------IK------D-----G-DVICG-GLERPEQ----PRSER--

--VKKDWDQWAE-IT-NAR--DRDHFWELVHHLDP-KAAACNYGQLAKYADWRFASVPPV

YESPGGISFIGGDVDGRDAWCEQSHI-RSG--P-RCMSLVLYGDSRTGKTLWARSLGTH-

-------LYTVGMV--------SGEELKKADDVKYAIFDDIRGIKFF--PAFKEWLGAQ-

--AYVTVKELYREPKLVKWGK-PSIWI-SNDDPRLGM----------DAS--DVS-WLEH

NC---YFVEVSSP--I--------------------F------RANTE------------

----------------------------

>Genomo|YP_009115515.1

LP--PR--SFNIHCRYVLLT---YAQC---GL----S-ADAVGERMASS--------GYK

CVIG----REDHADGG-----VHLHVFVDFGIKRRFRRAN-IFDVDG--CHP--NISPSG

--TPEKGYDYA---------IK------D-----G-DVVFR-SLDRP-------SAAG--

--AGKSVDKWAR-IT-GAV--DRESFWSLVHELDP-KSAACNFTSLQKYVDWKFAAVPPV

YESPAGISFRGGDVDGRDQWLLQSGI-GLG--P-ICMSICVYGESRTGKTLWARSLGPH-

-------IYCVGLV--------SGDECMKAQDAEYAVFDDIRGIKFF--PSFKEWLGCQ-

--AWVTVKCLYREPKLVKWGK-PSIWL-SNTDPRDHM----------ENS--DID-WMNK

NC---IFVEVNSA--I--------------------F------HASTE------------

----------------------------

>Genomo|YP_009115519.1

MP-------FNLHCRYALLT---YAQC---DL----S-PTAVGEFFDNL--------GYK

SVIG----RENHADGG-----VHLHCFVDFGRKRRFRRAR-VFDIEN--RHP--NVEPSG

--TPEKGWDYA---------VK------D-----G-DICYQ-SLDRPRES--SPSGGS--

--NGGTRDKWAS-IT-GAS--DREAFWDLVHELDP-KSAACCFTQLQKYCDWKFAPRTPE

YESPGGLEFIGGGLDGRDDWLLQSGI-GGR--ARRCMSICVYGESRTGKTLWARSLGPH-

-------IYCVGLV--------SGDECMKASTADYAVFDDIRGIKFF--PSFKEWLGCQ-

--AWVSVKCLYREPKLVKWGK-PSIWL-SNTDPRDYM----------ENS--DID-WMNK

NC---IFVEVNSP--I--------------------F------RANTE------------

----------------------------

>Genomo|KJ547638

MP------NFDIHCRYALIT---YAQC---DL----A-GPTVGEFFESS--------GYK

SVIG----RENHADGG-----VHLHCFVDFGRKRRFRRPR-VFDIEG--RHP--NIEPSG

--TPEKGWDYA---------VK------D-----D-DIVFQ-SLDRPGE-----SGGS--

--NGGTRDKWVA-IT-GAS--SRESFWDLVHELDP-KSAACSFTQLQKYCDWKFAPIPPV

YESPGGISFTGGDVDGRDDWLSQSGI-GSG--P-RCMSICVYGESRTGKTLWARSLGPH-

-------IYCVGLV--------SGDECMKANDAEYAVFDDIRGIKFF--PSFKEWLGCQ-

--AWVSVKCLYREPKLVKWGK-PSIWL-SNTDPRDYM----------ENS--DID-WMNK

NC---IFVEVNSA--I--------------------F------HANIE------------

----------------------------

>Genomo|AIF34843.1

MP--PRAGAFDIHSRYALLT---YSQC---DL----S-PATVGEFFTNL--------GFK

LIIG----RENHANGG-----IHLHCFVDFIRKRRFRAPR-CFDIEG--RHP--NVEPSG

--TPERGWDYV---------IK------D-----G-DVVFK-SLDRPVEE--HVAPRN--

--SSRSHDSWST-IT-GAS--DRESFWDLVHELDP-KSAACSFTQLQKYCDWKFAPSPPV

YGSPDGITFVGGETDGRDDWLLQSGI-GNR--P-RCMSICVYGESRTGKTLWARSLGSH-

-------IYCVGLV--------SGDECMKAETVDYAVFDDVRGIKFF--PSFKEWLGCQ-

--AWVTVKCLYREPKLVKWGK-PSIWL-SNTDPRDHM----------ENS--DID-WMNK

NC---IFVEVNSP--I--------------------F------RANTE------------

----------------------------

>Genomo|YP_009252356.1

M-------TFAINAKYVLLT---YAQC---DL----D-GFAVMDRISEL--------GGE

CIVA----RETHADGG-----THLHVFCDFGRKFRSRKTD-VFDVLG--YHP--NIEPSG

--TPEKGYDYA---------IK------D-----G-DVICG-GLARPTT-----SRTG--

--DSSANSKWTE-IT-SAT--NRDEFWELVHLLDP-KSAACSFGQLQKYCDWKFAVAPPT

YTSPTGVEFDDGSIDGRLDWLQQSGV-GSG--P-RCMSLCLYGESRTGKTLWARSLGAH-

-------IYCVGLV--------SGDECMKADDADYAIFDDIRGIKFF--PSFKEWLGCQ-

--AWVTVKCLYREPKLIKWGK-PSIWI-SNTDPRDNM----------ETS--DVH-WMNK

NC---IFVAVDSP--I--------------------F------RANTE------------

----------------------------

>Genomo|YP_009252353.1

MS------AFQVNSRYVLLT---YAQC---DL----D-PWAVNDLLSTL--------GAE

CIIG----RERHEDGG-----IHLHAFVDFNRKFRTRRSD-IFDVDG--HHP--NISQSG

--TPEKGYDYA---------IK------D-----G-DVVAG-GLGRPEG----KSGGG--

--DGSTHAKWTA-IT-QAE--NREQFWELCHELDP-KAAATSFTQLSKYADWRFAPDPPV

YEHPIGISFTDGDLDGRREWLDQAGI-GGG----RCKSLCLFGRSRTGKTLWARSLGQH-

-------IYCVGLV--------SGDECMKAPDVDYAIFDDIRGMKFF--PSFKEWLGAQ-

--AWVTVKRLYREPALVQWGK-PSIWL-ANSDPRNDM----------SQD--DVQ-WMED

NC---IFVECNET--I--------------------F------RANIE------------

----------------------------

>Genomo|AMH87666.1

M-------SFYFCARYGLFT---YSQC---DL----N-HWAVLDLFSGL--------GAE

CIIG----RELHEDGG-----THLHVFADFGRRFRSRSSK-IFDCEG--RHP--NVSASG

--KPDEGWDYA---------IK------D-----G-DVVAG-GLERPDG---VTRRSG--

--DSTSDQKWGE-IA-GAE--DRDEFWRLVHELDP-KSLVIHFPAISKYCDWKFSPRQVE

YVHPINIDFVGGEVDGRDHWLAQAGI-GLG--TRGMKSLCVYGKSRTGKTLWARSLGRH-

-------IYCIGLV--------SGAECARAEEVDYAVFDDIRGIKFF--HAYKEWMGAQ-

--AVVSVKLLYRDPKLVRWGK-PSIWL-SNKDPRCDM----------SQE--DVE-WLED

NC---IFVECNET--I--------------------F------RANTE------------

----------------------------

>Genomo|AGS12486.1

MP------QLEWNFRYVLVT---YAQC---DL----D-PWRVVERFSSL--------GAE

CIVG----REHHEDGG-----LHLHVFADFGRKFRSRKAD-ILDVDG--RHP--NLAPIR

--TPAKAYDYA---------IK------D-----G-DVVAG-GLERPVE-----GGVG--

--NGTSADKWSR-IT-QAE--DRDEFWALVHELDP-KAAACSFNALSKYADWRFAEKPPV

YEHDGRIEFVPGDADGRDDWVSQSGI-GLD--PGRVKSLVLFGGTRTGKTTWARSLGKH-

-------LYCIGLV--------SGAECSKGADAEYAVFDDIRGFGFF--HGYKEWLGAQ-

--PHVSIKQLYREPYYMKWGK-PSIWI-CNTDPRLDAY--GP-----ATP--DWE-WMEG

NC---HFIEVKDS--L--------------------ISS--ISHASTE------------

----------------------------

>Genomo|AMH87733.1

MP--SN--GLFVNSRYKLLT---YAQC---DL----D-PFDIVDLLSGL--------QGE

CIVG----RELHSDGG-----IHLHVFVDFGRKFRSRSVG-IFDVGG--RHP--NVVASG

--TPEEGYDYA---------IK------D-----G-DVVAG-GLERPTP---RESRAG--

--NGSAAAKWAT-IA-DAD--DREQFFELVKSLDP-KTFVTRLQDLQRFADWKFRSDPEP

YITPNNIHFPDDGTDGRAEWVHS--------------SLCLYGESRLGKTLWARSLGHH-

-------VYFCGLF--------SGKELLNNLDADYAVFDDMQGISFF--HGWKNWFGCQ-

--LNFQVKQMYRDPVNVTWGK-SCIWG-SNDDPRDSM----------KQV--DVD-WINK

NC---IFVEVTQP--I--------------------F------HANTE------------

----------------------------

>Genomo|AMH87708.1

MA--P---FILKNVRRALLT---YSQC---TL----D-PFAVSDHFSNL--------GAE

CIVG----RERHADGG-----LHLHVFVDFGRQFSSRKTD-VFDVGG--HHP--NIAKCR

--TPWKAYDYA---------IK------D-----G-DVVAG-GLDRPIE----ESGDG--

--SGTIDSTWHS-IL-AAE--TPEEFWALCHDLAP-RDLARSFPSLQKYCDWRYRPVTRP

YCTPDGYEFDTSRAPELNEWLLQANL-GLG--W-RPKSLVLYGDTRLGKTVWARSHGPH-

-------IYFCGLY--------SGAEAMKHDGADYAVFDDIQGIKFF--HGFKNWLGAQ-

--AEFQVKILYKDPVIIQWGK-PSIWL-SNSDPRLDL----------SPS--DAT-WLEG

NC---IFVELSVP--L--------------------FTS----RANTA------------

----------------------------

>Genomo|YP_009252365.1

MS------AFHFSARYVLLT---YPQS---EL----S-EWAVLDHISGL--------GAE

CIIG----REDHADGG-----THLHVFADFGRKKQSRRGD-YFDVGG--KHP--NVVPSG

--RPEGGWDYA---------TK------D-----G-NVVAG-GLGRP-------GTSG--

--LPKAPNPWRE-IV-GAE--GREEFLDLVRQLDP-KSFVLKHQEIVRYADIFFAEDREP

YVGPDGIRFELGMVPQLDEWRRESLG-DN---P-KSRSLVLYGPSRLGKTIWARSLGPH-

-------VYIMGLL--------SGAVLLRDPGASYAVFDDMRGLPMF--PSFKEWFGSQ-

--SLVTVKKMYRDPVQMRWGK-PCIWL-ANSDPRDQLK--ADITDR-IYE--DIA-WLEA

NC---VFVELSEP--I--------------------F------RANMHNSPE--------

----------------------------

>Genomo|YP_009252359.1

MP------TFYFSARYVLLT---YSQC---TL----D-EWDVLDHISSL--------GAE

CIIG----REDHADGG-----THLHVFVDFGHKKQSRRSG-FFDVGG--KHP--NVVPSG

--RAGEGWDYA---------VK------D-----G-NVVAG-GLPRP-------GSGG--

--LPSVENIWSS-IV-DAQ--SREEFLELVRALAP-KEFILRHKELLEYADRYYAERIEP

YVGPDGIEFELGMVPELAGWGRELVE-AD---S-EAKSLVLYGPSRLGKTLWSRSLGSH-

-------VYIMGML--------SGAVLLRDPDAQYAVFDDLRGIAMF--PSFKEWLGAQ-

--AVVTVKKLYRDPVQVSWGK-PCIWL-ANSDPRDQLK--ADITER-IYE--DIA-WLED

NC---VFVNVTEP--I--------------------F------RAST-------------

----------------------------

>Genomo|KT253577

MT------HFIFSARYVLLT---YAQS---DL----S-EWDILDHISSL--------GAE

CIIG----REDHADGG-----THLHVFVDFGRKKQSRRSD-FFDVGG--HHP--NITPSG

--RPECGYDYA---------IK------D-----G-DVVAG-GLARP-------GGGG--

--LPSLADKWRT-IV-SAE--SREEFFDLLRELDP-KTLVTRWSELCRYADHAYESKPEP

YVGPSGVEFELGMVPELVRWRGDTLA-DD---S--ELVLCYFGASRLGKTLWARSLGPH-

-------VYTMGIL--------SGHVLLRDPEVNYAVFDDMRGIGMF--PSFKEWLGAQ-

--AVVSVKKLYRDPVQVPWGR-PCIWL-SNADPRDQIK--SGLSDR-VEN--DIA-WLED

NC---IFVELLEP--I--------------------F------RASIG------------

----------------------------

>Genomo|AMH87678.1

M-------TFLFSARYVLLT---YPQC---TL----D-GWAVSDHLSAL--------GAE

CIVG----RENHSDGG-----THLHAFVDFGRKKQSRRSD-FFDVGG--HHP--NIAPSG

--RPERGYDYA---------IK------D-----G-DVVAG-GLARP-------GGGG--

--LPEIANKWSE-IV-SAE--SREQFFDLLRQLDP-KTLVTRWTELNKYADAAYAPTPEP

YVGSDGKQFELGMVPELARWGEQLVA-ND---P----NLVLYGPSRLGKTVWARSLGPH-

-------VYSMGIV--------SGKLLLRDPEAKYAVFDDMRGIGYF--HSWKEWLGAQ-

--SVVTVKELYRDPVQLVWGR-PCIWL-ANRDPRLELG--AYFTAR-FQS--DWA-WLET

NC---YFWGARTT--L--------------------F-----FRPNTD------------

----------------------------

>Genomo|YP_009109733.1

MP--PKANPYFADGRYFLLT---YAQC---TL----D-AWTVNDHLAFL--------GAE

CIIG----RELHADGG-----THLHAFCDFSRRFRSRRSD-VFDVGG--RHP--NLVPSG

--KPEGGYDYA---------IK------D-----G-DVVAG-GLSRP-------SGRG--

--VYEDVSSWSI-IV-GQE--SEGEFWKCVARLDP-RALCTNYNSLRAYANWRYRPAPVP

YEHPAGIEFELGMVPELAVWREIALG-AD---R----ILVIFGDTRLGKTLWARSIGPH-

-------IYTIGQM--------SGEVILRDPDADYAVFDDMRGLEFF--HGWKEWFGCQ-

--SVVTVKKLYRDPVQMPWGK-PVIWL-SNRDPRDELR--DSITNH-IEG--DIK-WLDG

NC---IFVELDHA--I--------------------F------RANTE------------

----------------------------

>Genomo|KT862241

MP-----KQFQFQARYVLLT---YAQC---DL----D-AWAVNDHLAFL--------GGE

CIIG----REHHADGG-----THLHAFCDFSRKFRSRRPD-VFDVGG--FHP--NIEASG

--RPEGGYDYA---------IK------D-----G-DVVAG-GLSRP-------GGRG--

--VYEDVSSWSI-IV-SQE--SEGDFWECVARLDP-RSLCTNYNSLRAYANWKYRPTPVQ

YVHPAGVEFELGMVPELAVWREIALG-AN---R-RAKSLVIYGDTRLGKTLWARSLGPH-

-------IYTIGQM--------SGEVILRDPDADYVVFDDMRGLDFF--HGWKEWFGCQ-

--TVVTVKKLYKDPMQMPWGK-PVIWL-ANRDPREELR--DSITCH-IEG--DIK-WLDG

NC---IFVELDHA--I--------------------F------RANTE------------

----------------------------

>Genomo|YP_009181999.1

MP------DFHCNAKYFLIT---YSQS---GL----D-EWAVNDHFSSL--------GAE

CIIA----REDHADRG-----THLHAFVAFERKFRSRRPD-IFDVGG--FHP--NIAPSG

--NPAGGYDYA---------TK------H-----G-DVVAG-GLERP-------GGGG--

--VSKTSTPWHW-II-AAS--SGGELRDLVRELAP-KEAILRWREIEGYIGNEFAEERPA

YVHPIGFEFELGMVPEL-----NLGL-AD----VRYKSLVIFGPSQTGKSTWARSLGNH-

-------FFARGKF--------NGREFVKVQSVDYYVLDDMEGLRFF--PGWKHFLGMQ-

--TWFNVRQFHRDPPMVKGGK-PCIWL-CNLDPRDEMY--ANLRHNQVDN--DVA-WLEA

NC---IFVEVHEP--I--------------------F------RANIA------------

----------------------------

>Genomo|KT598248

MP------DFHCNAKYFLIT---YSQS---GL----D-EWAVNDHFSSL--------GAE

CIIA----REDHADRG-----THLHAFVAFERKFRSRRPD-IFDVGG--FHP--NIAPSG

--NPAGGYDYA---------TK------H-----G-DVVAG-GLERP-------GGGG--

--VSKTSTPWHW-II-AAS--SGGELRDLVRELAP-KEAILRWREIEGYIGNEFAEERPA

YVHPIGFEFELGMVPELVEWRRNTLD-AD----VRYKSLVIFGPSQTGKSTWARSLGNH-

-------FFARGKF--------NGREFVKVQSVDYYVLDDMEGLRFF--PGWKHFLGMQ-

--TWFNVRQFHRDPPMVKGGK-PCIWL-CNLDPRDEMY--ANLRHNQVDN--DVA-WLEA

NC---IFVEVHEP--I--------------------F------RANIA------------

----------------------------

>Genomo|YP_009109727.1

ML---------VNSRYVLLT---YAQC---DL----D-PWSVSNHLSAL--------GAE

CIVA----REIHTTGG-----IHLHCFADFNRKFRSRSAR-IFDVDG--RHP--NVVPSG

--TPEKGYDYA---------IK------D-----G-DVRAG-GLGRPAP-----RGGM--

--SVGAHALVNV-AH-LCE--DTTEFLELHDEMDR-SGLIARFNNVRAYADWRFRPEPVV

YASPDGVDFRSGSTDGRDDWLVQSRI-GDE--PI-FCLLILYGPSLTGKTTWARSLGDH-

-------IFIQGVL--------SGKEVLNSESARYAVLDDIRGLKFF--PAWKDWLGGQ-

--RWISVKQMYRDPILLKWGR-PCIWC-ANRDPRADIR--RSIDKD-MED--DMD-WINA

NC---IFVYVDES--LVT------------------F------RASTE------------

----------------------------

>Genomo|YP_009252362.1

MT-----QMLFCNSKYVLLT---YAQC---DL----D-EWAVSDHLSSL--------GAE

CIVA----RELHTTGG-----VHLHVFVDFGRKFRSRRVD-IFDVEG--RHP--NVVPSG

--TPEKGYDYA---------IK------D-----G-EVVAG-GLERPRG-------GR--

--PGTVSGLEAI-AH-LCE--TQDEFLDIYGEVDT-RGLIKNFANVRSYAKWRYAGTLPK

YESPYSIGEFRRGSDGRDQWLAQSGI-RSG----RPKSLVLYGPSRTGKTSWARSLGTH-

-------VYFGGAF--------SGGDALACDDVKYAVFDDMRGIPFF--HGWKDWLGAQ-

--QEFMVKALYHDPKLFKWGR-PSIWC-ANRDPREEME--NHM---FCRG--DID-WLNA

NC---IFVEVNSP--I--------------------F------RASIE------------

----------------------------

>Genomo|AMH87702.1

MP-------LAVNAQHFLLT---YAHVE--EL----D-PFRIVDVLGAK--------GAE

CIVA----REFYNTGG-----FHFHVFCSFERNFRSRKAD-VFDVDG--YHP--NIEPSK

--NAAGGFDYA---------TK------D-----G-DIVAG-GLERPSR---TRGRAG--

--TTGAQATWGE-IT-AAE--SAEEFWRLCEELDP-KSMVCNFPALSKFAEWRFRPIPVP

YASPDGV-FDTSGYPIIEEWRSSVFD-EY---STYLTSLVLFGPTRLGKTTWARSLGSH-

-------IYFGGLF--------SAGEAMRCPEAEYAIMDDIAGIKFF--PRFKDWLGCQ-

--AQFQLKVLYKEPALYDWGK-PCIWC-YNVDPRAGM----------SVE--DIE-WLEG

NC---VFVEITSP--I--------------------F------HANTE------------

----------------------------

>Genomo|YP_009109725.1

MS-----QFCLKDAKYCLLT---YPQI---ET----E-AYEFPELASELF------VDAN

YVIG----RELHADGG-----YTSHCFLDFGRKFSSRDTR-IFDIQG--HHP--NIERVR

--TPRTAYNYT---------IK------D-----N-DVVAR-SGEYPPEN-NSRERQS--

--GNDTSSDWAT-IL-CAE--SRDEFFDLCKSLQP-RSLACSFLSLTRYADWRYRPVPTP

YQHPDDWSFNLESHSVLLDWVDESLR-GG-----QDRSLVMYGETRLGKTVWARSLGPH-

-------LYFCGLY--------SYKEASRAHEAEYAIFDDLQGIKFF--HGFKNWLGAQ-

--QEFQIKGLYRDPELLKWGK-PSIWC-SNTDPRQDL----------DYS--DRC-WLEG

NC---VFVEVTTS--L--------------------IG----------------------

----------------------------

>Genomo|YP_009164036.1

MP--SNR-FHIKGCRYALIT---YAQC---RP----RIPWAIVGLFSTH--------RAE

CTVG----RESHADGG-----IHFHALVDFGRQFGSRKAD-VFDVGG--RHP--NIQKSG

--TPEKGYDYG---------IK------D-----G-DVVAG-GLERPLS---GQSGSG--

--ARSSRVVWAT-IT-MAE--NAGEFWRLCHELAP-EH-VCRFPSYARATSCADRSLARS

LAARHGVTRNVCNADK-----------------CRRKSLVVVGESELDKTVWARSLGNH-

-------LFFSGLY--------SGAEAMRYADAEYAVFDGMQGIGSF--HGYKNWLGAQ-

--MNFQVKVLYPDPKMITWGK-PCIWL-SNEEPRN------------ESV--DQD-WLKE

NC---IFVDLWEN--IAG-----------------PI-----SHASTETL----------

----------------------------

>Genomo|AJD07464.1

-M-----PFHLKNRRYVLLT---YSQA---EF----N-YWAIVDMLSSH--------GAE

CIIG----RELDADGG-----THFHVFVDFGRLFSTRKTN-VFDVDG--HHP--NILPVK

--TPGEAFDYA---------AK------D-----G-DIVAG-GLERP-------GTDC--

--DYDIENFWAC-AG-ASQ--SGEEFLHFLDQLAP-RDLMRGFIQFRSYADWKWAVAPER

YVNPPGVMFDTGHAEQLSEWLSQANL-GSGVAH-SRKSLMLWGPTQYGKTTWARSLGNH-

-------IFFGSQF--------SGKLALDGQDAEYAVFDDWKGMKAL--PGYKDWFGCQ-

--WQISVRKLHHDAKLITWGR-PIIWL-CNKDPRLMHV--AT-----DDV--DWE-WMDD

NV---IFVELARP--LA------------------TF------RASTE------------

----------------------------

>Genomo|AMH87693.1

MP-----SFHLKNRRYVLFT---YSQA---DF----D-YWAVVDLLGDM--------GAE

CIIG----REVHADGG-----IHFHVFTDFGRLFSTRKVR-VFDVGG--KHP--NIKPIR

--TPAQAYDYA---------IK------D-----G-DVVAG-GLGRP-------GGDC--

--DWDPDNFWAA-AT-HTG--SSEEFLHFCDQLAP-RDFIRGFTNFRAYSNWKFNPGIPE

YDQPDGATYDTSAAPGIDEWLSQ------------RKSLVLFGPYGCGKTLWARSLARH-

-------IYFGSSW--------SGELAFAGDTAEYAIFDDWAGLKCL--PKYKDWFGAQ-

--WHISVRRLHHDAKLVEWGR-PIIWL-CNRDPRILSH--ER-----DDI--DWE-WMET

AC---HFVEVTGK--LV------------------TF------HANTE------------

----------------------------

>Genomo|YP_009109729.1

M-------TFILNARYFLVT---YPQS---GL----D-EWAVNDHFGSL--------GAE

CIVA----RENHAVRG-----THLHVFCDFGRKFRSRRAD-IFDVGG--FHP--NIERSR

--NPRKGALYA---------CK------D-----G-DIVAG-GLDVPGL-----ATSI--

--VSAAQDPGAT-LV-CAE--SQREFFELAEDICP-WDLITKFGSMHAYAKWKFPELAEP

YENPAGFTLADGAFPDLVSWRTGALE-HS-----RIKSLVLIGDALTGKTTWARMLGNH-

-------LYMKERY--------NAKQASLADGVDYGVIDDISGIKYF--PHWKSWFGGQ-

--PHIQVKILYKDERLVKWGK-PLIWI-ANRDPRDQLR--DMVSRD-CNN--DVY-WMEG

NA---IFVDIRQS--SI-------------------I-----SHANTE------------

----------------------------

>Genomo|AUM61807.1

-------MSFRVYSKTFLLT---YSQC---DL----P-KDAVKEYLNSIS------ELDE

YCIG----QEHHQDGN-----LHIHAWIKFKTKYQSRNPR-CFDING--FHP--NIMTAR

--SKKGSIAYV---------SK------E----DT-DPL---------------------

---QNIKDTYSE-IFDKAT--TSKEFMSLVLQNHA-RDYANNYDRLISMSKNHYKPEPVQ

YTP--KYKEFRNLPMDML---TINXN-DT----DRPRTLIIHGESRLGKTQWARSLGKH-

-------IYSRGCI--------IYDDFIRSPTAEYAVFDDLWDWNYF----LKDFIGAQ-

--QTVTITGKYRKPVQLNWSR-RSIFL-TNE-----FE--QW-----SQE--QQ--YLQA

NS---TIITLKNK--L--------------------Y-----------------------

----------------------------

>Genomo|YP_009351871.1

LP--LSSGKFRLAAKSVFLT---YPKC---HL----D-KEVFVKAIEKFKF--------Q

FYAV----REDHKDGT-----PHYHVIGEWSVKKNIKNPR-HFDIQK--YHP--NIGRTR

--NRLAAWRYV---------HK------T----KG-RSTHV-GGDMPE-----PVGIR--

--KEAKDDFWKE-AV-TIK--DRNTFISRFRQEAP-RDLIVHYSNVKAYADDEFKVEAPE

YCTPE-MSGYWDLPEELKEWVEENLT-HKS---ARPKSLCLYGETRLYKTIWARSLGPH-

-------SYMSGCW--------NAR--LLDDDKQYVVIDDVPLKDMF--KHFKQMLGCQ-

--NNFSVTDKYVKKLHFKWGL-PCIYL-ANQDPREYS----------DGT--HRR-WLNG

NC---VFVEVEKP--L--------------------FVA--SSGPGTPDSRQE-SQFLQ-

---------EAE----------QDESF-

>Genomo|KP153522

VE--AGPRTFRIDARHLELT---YSRC---NA----T-VEDITEFLETTV-SSA--QCHS

WFAA----REYHQDGV-----PHYHILVKFNKRRTFRNSR-EYDFNG--HHP--RIKPVR

--NVHDYFRYI---------SD------P-----S-KPSRD-TTFASTGW-RDGARGG--

--GRGRRDTWGD-IV-RAD--GKDEYFTLLKLHKP-RDYCLNLQRLEYTASRLFTAIT-P

YVPEYEYDTF-NVPEELHEWA------TVGLQP-RPKSLIICGPSRIGKTEWARSIGLH-

-------TYWYGQC--------NID--SWNDKMEYLIMDDFSPTKYL--PLWKGYFGAQ-

--KELNVTQKYRGIYTKLFRV-PVLWL-CNQIP--------------KDW--EQE-WLNM

NA---DTYFINDK--L--------------------YG----------------------

----------------------------

>Genomo|KJ938716

MP--P--REPRFNGIRFFLT---YARA---AI----S-IDDVADHLHQLA-------DSW

LEIV----QEDHPDGG-----IHYHVVLCFATRYQ-GALD-SFDVLG--HHP--NWTPIR

--NATNRRHYI---------RK------G-----I--RAKE-DQHTPDTR-GNVPEYS--

--SQAGRLNWGG-ILEQAT--TGEEFLALCRLHQP-KDWILRNEALTKYAASYYQEAREK

KSLPP--GT---LSSFLPRWMRYVYS-GSTPKPDRPKTLLLVGPTRLGKTHWAESLGRH-

-------SYMCGMW--------RSD--SFNDRDDYLILDDFD-FDFFH-GMRKAIWGAQ-

--PEFTTVDKWRKGVA-KWGK-PLIWL-CQKNPFVALK--NGRPVM-AEE--ERA-WYRA

NC---VEVHVTTK--L--------------------YPE---------------------

----------------------------

>Genomo|KM821748

-M--A--RTPRTNAQRYFLT---YSQA---AL----D-IDDLANFLHALA-------PSW

LEIV----QENHQDDG-----IHYHVVLCFDERFQ-RALD-VFDLDN--HHP--NIAIIK

--NASNRRHYI---------RK------G-----A-DRPKE-SEHTPDTR-GEVPPYS--

--TSAGRLNWGG-ILESAK--SEEEFLILVRVNQP-TEWVLRNDSIVKYAKTHYKAAREE

KVYPP--ES-WVVPPALDDWVAEVFS-DVTPRPDRPKTLLLVGPTRLGKSVWAKSLGRY-

-------SYMCGMW--------HSD--EFDESAQYLILDDFE-FDFFH-GMRKAIWGAQ-

--EVFTTTDKWRKGVA-RWGK-PTIWI-CQHNPFTAVK--TGLAVM-DET--ERD-WYKQ

NC---VEVHVNTK--F--------------------IRL--RTCSGRSNRRD--------

----------------------------

>pCRESS9|YP_007008175.1

MK----KNKFKLNTKDIFLT---YSKC---PL----G-KDKIHNHIKQLI-ISK--KKEY

IISN----TENHQDHKE----IHTHVLFQLTKRTTFHGER-FFDIEG--FHP--KIETAR

--DIEKSIDYI---------KK------D-----G-DFIEE-GTPRYEKY---VRQNQ--

--KEERKQEYY--KLTLQQ--VKKTLDDFIKNLDR-DFYYEQIDLIEKILKKKFIKQAEL

IDKGYDLNTFKV-DKSTQEIGTNQLKV------KRPLSIVIEGPSRLGKTEFIISYNHF-

-------NYIRGSF--------DFSKENYNDSFKVDVYDDIS-MNYISSGLLKNIIGGQ-

--RGFIVDVKYPPKRLLSGNK-LSIFL-VNPDI--SFE--SYCEED-EKH--AGETYIKS

NC---IFINVDNK--L--------------------YDDSLKDIPQSANKVEIE------

----------------------------

>pCRESS9|WP_015083745.1

MK----KNKFRLQTKDIFLT---YSKC---PL----G-KDKIHNYIKELL-VSK--KKEY

IISN----TENHQDHKE----IHTHVLFQLNKIIQIENQR-FFDIEG--FHP--KIENAR

--DIEKSIDYI---------KK------D-----G-DFIEE-GTPRHKKY---VRQNQ--

--KEERKQEYY--KLTLNQ--VKKTLDEFIINLDR-DFYSEQIDLIEKILKKKFIKQAEL

IDKGYDLNTFKV-DKSTQEIGTNQLKV------KRPLSIVIEGPSRLGKTEFIISYNHF-

-------NYIRGSF--------DFSKENYNDSFKVDVYDDIS-MNYISSGLLKNIIGGQ-

--RGFIVDVKYSPKRLLSGNK-LSIFL-VNPDI--SFE--SYCEED-EKH--AGETYIKS

NC---IFINVDNK--L--------------------YDDSLKDIPQGADKVEIE------

----------------------------

>pCRESS9|ATL14544.1

MK----KTKFRLNTKDIFLT---YSKC---PL----G-KDKIHNHIKELM-VSK--KQEY

IISN----TENHQDHKE----IHTHVLFQLTKRFNIKSER-FFDIEG--FHP--KIENAR

--DIEKSIDYI---------KK------D-----G-DFIEE-GTPRHKKY---VRQNQ--

--REERKQEYY--DLTLSQ--VRKSLDNFIKNLDR-DFYYEQIDLIEKILKKKFIKQTEL

LDKGYDLDTFKV-DKSTQEIGSDQLKV------KRPLSIVIEGPSRLGKTEFIISYNHF-

-------NYIRGSF--------DFSKENYNDSFKVDVYDDIS-INYISAGLLKNIIGGQ-

--RGFIVDVKYSPKRLLSGNK-LSIFL-VNPDI--SFE--SWCEED-EKY--AGETYIKD

NC---IFIYVNNK--L--------------------YDDALQDNLQGSDKVEIE------

----------------------------

>pCRESS9|YP_001708784.1

MK----KTKFKIKTKDIFLT---YSKC---PL----G-KDKIHNHIKQLM-ASK--KKEY

LITN----QENHKDHKE----IHSHVLFQLTKSATFNGER-FFDIEG--FHP--EIEVAR

--DIEKSISYI---------KK------D-----G-DFIEE-GTPRHKKY---VRQNQ--

--KEERKQEYY--DLTLQQ--VKKTLDEFIINLDR-DFYLEQIDLIEKILKKKFIKKDEL

LDKGYDLNTFKF-NKSTQEIEVEQLKV------KRPLSIVIEGASRLGKTEFIISYNHY-

-------NYIRGSF--------DFSKENYNDNFKVDVYDDIS-INYISAGLLKNIIGGQ-

--GGFIVDVKYSPKRLLSGNK-LSIFL-VNPDI--SFE--SWCEED-EKY--AGETYIKT

NC---IFIYVPDK--L--------------------Y-----------------------

----------------------------

>pCRESS9|YP_001965310.1

MK--ETKTNFRLQTKDIFLT---YSKC---PL----G-KEKIHNHIKQLM-ESK--NQKY

IISN----TENHQDHKE----IHTHVLFQLNKRCNLTSQR-FFDLDG--YHP--KIENTR

--DVEKAIEYI---------KK------D-----G-DFVEE-GTPNRKKY---VRQNQ--

--KEERKQEYY--KLTINQ--VKKTLDDFIINLDR-DFYLEQIEFIKRVLKEAFAKKEEL

ADDDYSFESFKT-NSTTNEIIQSQLSV---SLSKRPKSIVIEGPSRIGKTEFLLSYTHY-

-------NYIRGEF--------DFSKESHKNAYKINIFDDIS-IPQIKEGLFKNIIGGQ-

--RGFRFNVKYAPKRFIAGKK-INIFL-INPDI--SFK--GYCEWS-YKK--GHKFYIED

NC---IFIYVSDK--L--------------------Y-----------------------

----------------------------

>pCRESS9|YP_001708790.1

ME----KNKFKINAKDIFLT---YSKC---PL----G-KDKIHNHIKELM-ASK--KKEY

LITN----QENHKDHKE----IHSHVLFQLTKRTTFNGER-FFDIEG--FHP--EIETAR

--DIEKSINYI---------KK------D-----G-DFIEE-GTPRHKKY---VRQNQ--

--KEERKQQYY--NLTLSQ--VKRTLDKFIINLDR-DFYYEQISFIKTILNEVFTKKKEL

EDEDYSFDSFKS-NSKTNEIINQQLSV---SLGERPKSIVIEGFSRLGKTEFILSYTHY-

-------NYTRGDF--------DFSKQSHKNAYKVNIFDDIS-IPKIKEGLFKDIIGGQ-

--KGFKYNVKYAPKRTIAGKK-LSIFL-VNPDI--SFE--DYCEWS-ENN--GHKFYIRD

NC---IFIYVPDK--L--------------------Y-----------------------

----------------------------

>pCRESS9|YP_007008179.1

MK----KTKFKLNTKDIFLT---YSKC---PL----G-KDKIHNHIKQLL-VSK--KKEY

IISN----TENHQDHKE----IHTHVLFQLTKPTTFNGER-YFDIEG--FHP--KIENAR

--DIEKSIDYI---------KK------D-----G-DFIEE-GTPRYKKY---VRQNQ--

--KEERKQEYY--KLTFSK--VKKTLDNFIKNLDR-DFYYEKIDFVEQVLNKKFTKKKEL

EDEDYSFDSFKS-NSKTNEIIETQLSV---SLGERPKSIVIEGFSRLGKTEFILSYTHY-

-------NYTRGDF--------DFSKQSHKNAYKINIFDDIS-IPRIKEGLFKQIIGGQ-

--KGFKHNVKYAPKRTIAGKK-LSIFL-VNPDI--SFE--NYCEWS-ENN--GHKFYIKD

NC---IFIYVPEK--L--------------------Y-----------------------

----------------------------

>pCRESS9|WP_013747472.1

MK----KNKFRLQTKDIFLT---YSKC---PL----G-KDKIHNYIKELM-ISK--KQEY

IISN----TENHQDHKE----IHTHVLFQLTKQLSIRNQR-FFDIEG--FHP--KIENAR

--DIEKSIDYI---------KK------D-----G-DFIEE-GTPRHKKY---VRQNQ--

--KEERKQEYY--SLTLNK--VRKSLDDFILNIDR-DFYLEQSELIDRILNKKFTKKKEL

EDEDYSFDSFKS-NSKTNEIIQTQLSV---SLGERPKSIVIEGFSRLGKTEFILSYTHY-

-------NYTRGDF--------DFSKQSHKNAYKVNIFDDIS-IPQIKEGLFKQIIGGQ-

--KGFKYNVKYAPKRTIAGKK-LSIFL-VNPDI--SFE--NYCEWS-EDN--GHKFYIKD

NC---IFIYVPDK--L--------------------Y-----------------------

----------------------------

>pCRESS9|WP_011412958.1

MK----KTKFQKNAKDIFLT---YSKC---PL----G-KDKIHNYIKEIM-ISK--KQEY

IISN----TENHQDHKE----IHTHVLFQLTKRFNIQSDR-FFDIEG--FHP--RIETAR

--NIEKSISYI---------KK------D-----G-DFIEE-GTPRHKKY---VRQNQ--

--KEERKQKYY--KLTLNQ--VKKSLNDFIKILDR-DFYYEKIDFVEQVLNKKFTKKKEL

EDEDYSFDSFKS-NSKTNEIINQQLSV---SLGKRPKSIVIEGPSRLGKTEFILSYTHY-

-------NYTRGEF--------DFSKQNHKNAYKINIFDDIS-LTRIKEGLIKDIIGGQ-

--KGFSYNVKYAPKRTIAGKK-LSIFL-VNPDI--SFE--NYCEWS-RNK--GYKYYLED

NC---IFIYVPDK--L--------------------YH----------------------

----------------------------

>pCRESS9|ABC65268.1

-------------------------------------------------M-ISK--KQEY

IISN----TENHQDHKE----IHTHVLFQLTKTIEIKSQR-YFNIDG--FHP--RIETAL

--NIEKSISYI---------KK------D-----G-DFIEE-GTPRHKKY---VRQNQ--

--KEERKQKYY--KLTLNQ--VKKSLNDFIKILDR-DFYYEKIDFVEQVLNKKFTKKKK-

------------------------------------------------------------

------------------------------------------------------------

------------------------------------------------------------

------------------------------------------------------------

----------------------------

>pCRESS9|YP_006961991.1

MK----KTKFRLNTKDIFLT---YSKC---NL----G-KDKIHNHIKELM-ASK--KQEY

IISN----TENHQDHKE----IHTHVLFQLTKRLNIKSER-FFDIEG--FHP--KIENAR

--DIEKSIDYI---------KK------D-----G-DFIEE-GTPRHKKY---VRQNQ--

--KEERKQEYY--NLTLTQ--VKKTLDEFIINLDR-DFYLEQIDLIEKILKKKFIKKDEL

DEEAYSFDSFKT-NSETNKIIDSQLNINS-KTNKRIKSIVIEGPSRLGKTEFILSYTQY-

-------NYIRGEF--------NFSKESHKNAYKVSIFDDIS-IPEIRAGLLKNIIGGQ-

--RGFEYNVKYSPKRTVMRNR-LSIFL-VNPDI--SFE--SYCEWS-RDN--GHKFYLED

NC---IFIYVNNK--L--------------------YDDALHDNLQDSDKVEVE------

----------------------------

>pCRESS9|WP_011412950.1

MK----KTKFRLNTKDFFMT---YSQC---DL----G-KEKIYHHIKQLM-ASK--NQEY

LSVC----LENHADNNG----VHSHVFLQLKKRYQVVNNR-FFDIDG--KHP--EIERAR

--TVQGSVDYV---------KK------D-----G-DFIEE-GTPKDKKY---ITKNE--

--NDKLKKEYY--NLTFNQ--VKKSLDDFILKTDR-DFYYEKIDLIKKILNERFTKKKEL

EDEDYSFNSFKT-NSKTNEIIQTQLSV---SLGKRPKSIVIEGPSRLGKTEFILSYTHY-

-------NYTRGEF--------NFSKENHKNAYKINIFDDIS-LTKIKEGLIKDIIGGQ-

--KGFTVDVKYAPKRNISGKK-LNIFL-VNPDI--SFE--NYCEWS-RND--GHKFYIED

NC---IFIYIPDK--L--------------------YEE---------------------

----------------------------

>pCRESS9|CBX25033.1

MK----KTKFRLRTKDFFMT---YSQC---DL----G-KEKIFNHLKQLM-ASK--NQEY

LSVC----LENHADNNG----VHSHVFLQLKKYLDIQSGR-FFDIDG--KHP--EIERAR

--TVQGSVDYV---------KK------D-----G-DFIEE-GTPKDKKY---ITKNE--

--NDKLKQEYY--KLTLNQ--IKKSLNDFILKTDR-DFYYEKIDLIKKILNERFTKKKEL

EDEDYSFNSFKS-NSKTNEIIQTQLSV---SLGKRPKSIVIEGPSRLGKTEFILSYTHY-

-------NYTRGEF--------NFSKQSHKNAYKINIFDDIS-LTKIKEGLIKDIIGGQ-

--KGFTVDVKYAPKRNISGKK-LSIFL-VNPDI--SFE--NYCEWS-RNN--GHKFYIED

NC---IFIYIPDK--L--------------------YEE---------------------

----------------------------

>pCRESS9|YP_001965305.1

MK--ETKTNFRLRTRDIFLT---YSKC---PL----G-KEKIHNHLKQLL-ASK--KKEY

IISN----NENHQDHKE----IHTHVFIQLKKQIEITNQR-FFDIEG--YHP--KIETAR

--DVEKSVSYI---------KK------D-----K-DFIDE-GEYIQKKY---VRQNQ--

--KEERKQEYYNDSLNINK--IRKEIDDFILKIDR-DFYYEQIELIDRILNRRFIRKKEL

ADTHYQFNSFKT-NSETNEIINSQLLS------HRPKSIVIEGESRMGKTQFILSYTQY-

-------NYIKGEF--------DFSKKTYKDYYKIDVYDDFG-VPEISQGLQKNIIGGQ-

--ECFTCNVKYAPKRQLSGNK-LSIFL-VNPDN--SFK--GYCEWS-RNN--GHKFYIEE

NC---IFIYVSDK--L--------------------F-----------------------

----------------------------

>pCRESS9|WP_017193171.1

IK--ETKNKFQFNSKDIFLT---YSKC---SL----G-KNVIHNHIKNLM-NEK--KKNY

IISN----TENHADHKE----IHTHVLLQLEKRSNIKDAR-FFDIEG--FHP--RIENAQ

--HIEKSIDYI---------KK------D-----G-DFIEE-GTPRIKKY---VRQNQ--

--KEERKQEYY--KLKMHE--VKKTLDEFIKKMDR-DFYYEQIELIERILKKKFIKKEEM

NEEIYDFDSFKD-NEITRKIIQKEKDV------HRPRSLVIEGLSRIGKTEFIISYNPF-

-------NYIRGSL--------DFSKEIYKNEYKINVFDDIS-IFEIKHGLLKNIIGGQ-

--RGFNADIKYAPKRRIAGNK-LNIFL-CNEDI--SFV--RFCKKN-KEM--GGKEYIEK

NC---LFFNIDEK--L--------------------YKE---------------------

----------------------------

>pCRESS9|WP_017193695.1

IK--ETKNKFRFSSKDIFLT---YSKC---SL----G-KNVIHNHIKNLM-NEK--KKNY

IISN----TENHADHKE----IHTHVLLQLEKIFQTENAR-FFDIEG--FHP--RIENAQ

--HIEKSIDYI---------KK------D-----G-DFIEN-GIPRIKKY---VRQNQ--

--KEERKQEYY--SLKINK--IRKNLDEFMIKIDR-DFYLEQIELIERILKKKFIKKAEM

NDNIYDFDSFKD-NEITRKIIQKEKDV------HRPRSLVIEGLSRIGKTEFIISFNPF-

-------NYIRGSL--------DFSKEIYKNEYKINVFDDIS-IFEIKHGLLKNIIGGQ-

--RGFNADIKYAPKRRIAGNK-LNIFL-CNEDI--SFV--RFCKKN-KEM--GGKEYIEK

NC---IFFNVKEK--L--------------------YKE---------------------

----------------------------

>pCRESS9|ABC65385.1

IN---EKNKFRLYTKDIFLT---YSKC---PL----G-KEKIHNHIKELM-ASK--KQEY

IISN----TENHQDHKK----IHIHVLFQLTKRFNIQSDR-FFDIEG--FHP--RIKTAR

--NIEKSISYI---------KK------D-----G-DFIEE-VTLDIKIY---VRQNQ--

--KEECKQQYY--NLTLSQ--VKKYLDYFIKNLDL-DFYYEQIDLIEKILKKSLSNNMRW

KNK----------KSKPNQFLLLRIMI---------------------------------

----------------------------------------------VK------------

--------------------K-L-------------------------------------

------------------------------------------------------------

----------------------------

>pCRESS9|YP_006959585.1

----NIKKETMFQAQNIFLT---YSQC---DL----S-KEEIKTFIINLC-NEK--KLQY

LIIG----IENHQDHKG----KHHHVFFQLNKQFRTRDLT-IFNIPKNIYSP--HIEPIK

--DTTDVRNYV---------KK------D-----G-DFIEE-GTFKHVRY---IKLSK--

--NEELESEYQ--SKTKNE--IFKKLKLYAESLEP-NYAFKNA--------KRFKNMVFI

FESIFDFCTFKK-IPILISIYETQKEQ--SSISKRFKTLIVEGNSKSGKTQFFKSVTPF-

-------NYIKDDV--------DFSDENYDEDKCVNIYDDID-IYDIARNLTKVVIGNQ-

--KDSIVNMKYKPRTKIKGTD-ISIML-VNEDT--SIE--KYCFDN-FKR--GRKEYIRE

NA---IFINLDKH--T--------------------ITH--YEYQKYENEQAVI------

LL------LDQE----------NES---

>pCRESS9|WP_015060110.1

----NIKKETMFQAQNIFLT---YSQC---DL----S-KEEIKTFIINLC-NEK--KLQY

LIIG----IENHQDHKG----KHHHVFFQLNKQFRTRDLT-IFNIPKNIYSP--HIETIK

--DTTDVRNYV---------KK------D-----G-DFIEE-GTFKHVRY---IKLSK--

--NEELESEYQ--SKTKNE--IFKKLKLYAESLEP-NYAFKNA--------KRFKNMVFI

FESIFDFCTFKK-IPILISIYETQKEQ--SSISKRFKTLIVEGNSKSGKTQFFKSVTPF-

-------NYIKDDV--------DFSDENYDEDKCVNIYDDID-IYDIARNLTKVVIGNQ-

--KDSIVNMKYKPRTKIKGTD-ISIML-VNEDT--SIE--KYCFDN-FKR--GRKEYIRE

NA---IFINLDKH--T--------------------ITH--YEYQKYENEQAVI------

LL------LDQE----------NES---

>pCRESS9|WP_011264167.1

----NIKKETMFQAQNIFLT---YSQC---DL----S-KEVIKTFNINLC-NEK--KLQY

LIIG----IENHQDHKG----KHHHVFFQLNKQFRTRDLT-IFNIPKNIYSP--HIEPIK

--DTTDVRNYV---------KK------D-----G-DFIEE-GTFKHVRY---IKLSK--

--NEELESEYQ--SKTKNE--IFKKLKLYAESLEP-NYAFKNA--------KRFKNMVFI

FESIFDFCTFKK-IPILISIYETQKGQ--SSISKRFKTLIVEGNSKSGKTQFFKSVTPF-

-------NYIKDDV--------DFSDENYDEDKCVNIYDDID-IYDIARNLTKVVIGNQ-

--KDSIVNMKYKPRTKIKGTD-ISIML-VNEDT--SIE--KYCFDN-FKR--GRKEYIRE

NA---IFINLDKH--T--------------------ITH--YEYQKYENEQAVI------

LL------LDQE----------NES---

>pCRESS9|BAD36752.1

----KVKKETIFKAQNIFLT---YSQC---DL----S-KEEIKTFIINIC-DEK--KIQY

LVVG----IENHQDHKG----KHHHVFFQLNKRLQTRDLT-IFNIPKNNYSP--HIEPIK

--DTTDVRNYV---------KK------D-----G-DFIEE-GTFKHVRY---IKLSK--

--NEELESEYQ--SKTKNE--IFKKLKLYAESLEP-NYAFKNA--------KRFKNMVFV

FESIFDFCTFKK-IPILTSTYETQKEQ--SSISKRFKTLIVEGNSKSGKTQFFKSVTPF-

-------NYIKDDV--------DFSDENYDEDKYVNIYDDID-IYDIARNLTKVVIGNQ-

--KDSIVNMRYKPRTKIKGSD-ISIML-VNEDT--SIE--KYCFDN-FKR--GRKEYIRE

NA---IFINLDKH--T--------------------ITH--YEYQKYENEQAVI------

LL------LDQK----------NKS---

>pCRESS9|WP_042068233.1

----KVKKETIFKAQNIFLT---YSQC---DL----S-KEEIKTFIINIC-DEK--KIQY

LVVG----IENHQDHKG----KHHHVFFQLNKRLQTRDLT-IFNIPKNNYSP--HIEPIK

--DTTDVRNYV---------KK------D-----G-DFIEE-GTFKHVRY---IKLSK--

--NEELESEYQ--SKTKNE--IFKKLKLYAESLEP-NYAFKNA--------KRFKNMVFI

FESIFDFCTFKK-IPILTSTYETQKEQ--SSISKRFKTLIVEGNSKSGKTQFFKSVTPF-

-------NYIKDDV--------DFSDENYDEDKYVNIYDDID-IYDIARNLTKVVIGNQ-

--KDSIVNMKYKPRTKIKGSD-ISIML-VNEDT--SIE--KYCFDN-FKR--GRKEYIRE

NA---IFINLDKH--T--------------------ITH--YEYQKYENEQAVI------

LL------LDQK----------NKS---

>pCRESS9|WP_012662291.1

----KVKKETMFQAQNIFLT---YSQC---DL----S-KEEIKNFIINLC-NEK--KLQY

LLVG----IENHQDHKG----KHHHVFFQLNKRFKTRDLN-IFNIPKNIYSP--HIEPIK

--DTTDVRNYV---------KK------D-----G-DFIEE-GTFKHVRY---IKLSK--

--NEELESEYQ--SKTKNE--IFKKLKLYAESLEP-NYAFKNT--------KRFKNMAYI

YESIFNFCTFKK-IPILICIYKTQKEQ--SVISQRFKTLIVEGNSKSGKTQFFKSVTPF-

-------NYIKDDV--------DFSDENYDEDKYVNIYDDID-IYDIARNLTKVVIGNQ-

--KDSIVNMKYKPRN---------------------------------------------

------------------------------------------------------------

----------------------------

>pCRESS9|KXT29032.1

KN--KKSKPYRMHSRNIGFT---YPNL---SL----S-KEEVQKIFIQKE-SRK--RKYA

LRIS----RELHED-GE----PHIHILIQLNKKTEFCNAREFFALPT--FNK--NNETPE

--HWYR---YI---------GA------Y-----G-DVLDD-GIFKFKQF---SNKQK--

--IDDFIYILL---ISAFE--AEKALNQYLQELDV-VIYYKQFPIRDRVIQENFYPKSPV

VKREHSLQTFRLYHEKVQLIIKEQFNS------KSPLTIVLEGLTQIGKTDLAELIQPY-

-------NYTKIDF--------NFSRENYNDSYKICIYDDMG-MEEVSKKLMHALIAGR-

--GSFQTREPYGKKRTISGNK-LNIFI-VNRNK--SFK--GWIEKN-KEW--KRFEYVEP

NV---IIIDLFKD-----------------------FKK--DYPLFYKPEEIKESEILAK

LM------VEVI----------KE----

>pCRESS9|KXT29014.1

IP--KNRKPYRLKTKMVGLT---YSRF---PV------KEKFIQLLKELF-NSKKNKINY

DISC----NDYHMDSG-----LHIHALLVFDKKIQLRDAQKVFAL------P--NYQTAK

FGHLDRFRAYI---------IK------K-----G-NFIEN-GI--PKK-----SRKQD-

--ERKKNEDIERLVLKSGLIVAEKQL-SFGYKLDN----VLYWDKLRDKMSKTF-----V

FELIYDFDSLFEPFNMVEKMVLKLMNSGV-----RKKSLFIVGGTGIGKTRMIKTILKY-

-------SYIKGKI--------DFSPKKFDDSRPVVIMDDIT-LQYLPEDGFKNFIGNG-

--DTTEVDVKMQKTATITGGK-LFIYI-VNKHPEKWVK---------NDEYDHI--YIRK

NI---EVIEFDEKDKLF-------------------YTD------EEKKYRQKLSEVLAR

LM------VEVIE---------------

>CRESSV6|KT732829

GP--IRQRNFRINATRFLLT---YPQS---TF----D-VQLVWDFFNSLA---I--KPKR

AILC----REHHQDGS-----EHVHVAIEFERAVNTTRVS-IFDFGG--RHP--NIQSAR

--NWAACVNYC---------RK------E-----GCTAEDA-TVAS-----APGERAP--

--AESPYARAES-RT-IRE--WFEYCIAEG-----------IGYAYANAIWNQIHSVRPY

FEN--FSGIVSGPTLSHLRW-------DP-----EWHTIFLCGPSGLGKSSWALANAPV-

-------FLFVTDI--------DDLGFFDPEVHKALVFDEIR-CTKWPLTSQIKLATWD-

--TPVSIRIRYKI-AHLPAHV-PKIFTSCDWFPM-------------GDE--QIRR----

--RI-KAFNLY-D--GRSTESLWL------------------------------------

----------------------------

>CRESSV6|KM874358

-M--ARENRFRLQCRRLFLT---YPHC---TA----S-IQEVYDLIHSKK------EIKF

ARIC----IEPHSDGT-----PHIHAAVMFAKKVNICNAR-LFDIND--HHC--NMESIK

--NWPATLNYC---------KK------G-----D-NWEDF-DNDD-----DPDQP-M--

--QDNLFELAET-MP-SND--FWEYCRLKK-----------VPFAYASNA---MRKKKSI

-TDPYIIREDLGAYTITEDS-------PA-----TLASTVLVGPSGIGKTSWAIRESEK-

-------SLLVTHM--------DALRKL-NGSHKSIIFDDMS-FTHIPREAQIHLVDRF-

--QPRQVHVRYGT-ADIPAGI-QKIFT-ANQYPF-------------ADP--AIDR----

--RV-HNKNLY-------------------------------------------------

----------------------------

>CRESSV6|AJD07486.1

LS--TSTNSFRIQAKSFFLT---YPQC---SA----S-KQDLKNFLDTKG------RIVY

YLIG----QERHEDGN-----FHLHALVTYEKKINVKRTT-FFDLNG--FHP--NIQAAK

--NLPALKNYI---------SK------E-----D--IEPL-TSTV-----EPEE-----

--EDNLYDLARV-TP-EEN--YFEICRKRKVSIIS-----RVPFMYANQAFLKILKDASI

-GE-YVIGTITSTVLQNLQL-------PT-----DMTSLWVKGPSGIGKTTWALTVSNK-

-------ALFVRHL--------DTLREFRNGYHKTIIFDDMS-FQHLPRTAQIELVDRY-

--HPQQIHIRYAV-VNIPPNI-PKIFL-SNDSIF-------------YDE--AIFR----

--RL-TLVNLE-T--DQ-------------------------------------------

----------------------------

>CRESSV6|KT149395

--------MFRINAKNFSLT---YSNV---EQ---WD-KESLLRHLESLG------DGVI

GMVS----REQHQDGS-----THFHAWVQFPRKRDIRASN-FFDWQG--CHP--NVQATN

--NLRAWKTYI---------CK------D-----G-DH------TP-----EPATPTA--

--TGDLFVLCHS-ME-KHE--WVNYCIGKK-----------IGFQYMEYIWKDCHMDRDI

-ED-PPEAVMCAALEQFIYP-------SL-----DRSPLVLVGDTGCGKTTWAIKNAPK-

-------ICFVSHM--------DQLKHFDPQYHKSIVFDDMD-FKHLPRESQIHILDMT-

--TPRAIHRRYGV-TVIPAGT-KKIFT-ANSNPF-------------EDP--AIIR----

--R--RTLRIV-G--L--------------------------------------------

----------------------------

>CRESSV6|KM598390

-------MPFRFAARNVFLT---YSHQ---EG----N-MQELFQRLQEIK------PTNY

VVLS----KEQHGDGT-----DHFHAVLCFKRKLDTVNPR-LFDFNG--WHP--KIESPR

--SVAASITYV---------KK------D-----G-DFLEV---GT-----EPTNRTF--

--T-DLHAKCQE-ME-RAE--WEEYCIGEH-----------ISFAYCESIYNRTHPRQRI

YEG-ENDGELSLALERFSWG-------FP-----ESQSLILVGPSGCGKTLWAKRNAPR-

-------CLFISHI--------DRLTEFDERVHKSIIFDDMT-FNHYPLQAQIHIVDQY-

--DDRDIHVRYKT-AFIPKKT-PKVFT-CNEEPF-------------RHE--TIKR----

--RS-KTYNII-------------------------------------------------

----------------------------

>CRESSV6|KP153501

-------MPSFYNGKQFFLT---YPQC---QH----S-AAELAAFLTSVA------TCTY

YIVA----EEKHEDGT-----PHLHACIQYVETLRG-GVR-LLDFNG--HHP--NKQDPR

--KFEACKQYC---------RK------D-----G-NFIE-----------GPPEAII--

--RAAIAGLAPS-TD-KAL--WFDWCISKR-----------ITHGYAEWYWNSTR-EHDI

-NE-TVAGQMCESLAGYAFN-------RD-----QHRVIVLKGESGCGKTTWAKTHAPK-

-------ALFVSHI--------DSLKRFQPGFHKTIIFDDVD-FNHYPRTGQIHIVDWE-

--NPRAIHVRYGT-VEIPAGT-FKIFT-CNFDPL-------------TDD--AIRR----

--RV-KVVNVN-------------------------------------------------

----------------------------

>PpulchraPlasmids|OLY79419.1

TR--SSDGTFRLSAKSFFLT---YPNC---SL----T-KQEVYDEMKKPF------EMTK

YIIA----VESHEAGH-----PHVHVYLKSKNKLNVKSAR-YFDIGG--YHG--EYGTCE

--SASAVSGYV---------IK------G-----G-DFIT-----------NIDESEY--

--RRIVTNAVQ--FV-DAP--DFEEAMEIVMYNMG-RDWVKNPLAYEQGVRYIKRKSKK-

-----NPNYKFRELPLVSFW-------NP-----DKRVLWMHGGTGLGKTEFAKTLFYN-

-------PLLVKHI--------EQLKDLHS-DHDGIIFDDTK-FAAWDRNDQLLLTDVE-

--NDNAFGVKYGA-VHIPRLT-GRIFI-SNNPIF-------------LDS--AIRR----

--RI-FYIYID-D--LRLMEPQEYEDLS-----DNVFLP-PQ------------------

----------------------------

>PpulchraPlasmids|OLY79389.1

SL--STHGAV----------------C---LL----T-KQEVYDELKKPF------TISK

YIIA----MEVSGAGN-----PHIHVYLKTGSKLNIKNAR-YFDIRG--YHG--DYRSCL

--SLKKIACYV---------TK------G-----G-NFIT-----------NMLEAEY--

--ISDVANAVK--VI-EAE--GFEEAMEVIMYNLG-RDWLRNPMAYEQGIRYIKRRGTK-

-----NPNYKFVDLPILSSW-------DP-----LKYSLWLFGGAGLGKIEFAKTLFKN-

-------PLLVKHI--------EQLKGLHS-DHDGIIFDDTK-FDRWTRNDQLLLTDIE-

--NTNAFGVKYGS-VTIPRNT-GRIFI-SNNKIF-------------QYP--EIIK----

--RL-FIVRMK-D--LKIIEPGKYVEDT--------------------------------

----------------------------

>PpulchraPlasmids|OMJ21113.1

AP--AKKKAYRLASMSFFLT---YPKC---AL----S-KETVLAELKKIC------DIET

YIVA----RELHADGT-----PHIHAYLKVKKTINSKNPR-YFDIMG--YHG--NYESCR

--YPKNVMKYV---------MK------D-----K-NYIT-----------NSTESQS--

--KKSGLDTIN--VF-ACS--TVEEAIAVIRYNLA-RDYLRNPIGYEQGLAHILHPPKP-

-----NPAHRFRDVPELLNW-------DR-----QDKTLWLYGVTGTGKTSFAKTLYTM-

-------PLFVTHL--------EDLKKIKP-YHNGIIFDDMN-FVTFSREEMIKLTDLD-

--DDRSFSVKYTS-KTIPAKM-SRVFC-SNVSIF-------------DDK--AIRR----

--RL-KIVLVT-D--LRIVDADTDLNYN-----QHCFTA-ID------------------

----------------------------

>PpulchraPlasmids|OMJ28371.1

AP--AKKKAYRLASMSFFLT---YPKC---TL----S-KETVLAELKKIC------DVET

YIVA----RELHSDGT-----PHIHTYLKVKKTINSKNPR-YFDIIG--YHG--NYDSCR

--YPKNVMKYV---------MK------D-----K-NFIT-----------NKPESQS--

--KKSGMDKIN--VF-GSK--TVQEAISVVKYNLA-RDYLRGPVSYELGLNHIVHPPKP-

-----NINHRFRELPELRNW-------NR-----QEKSLWLYGLMELENQPFPK------

-----------------------------P-Y----------------------------

------IQIRYL------------------------------------------------

------------------------------------------------------------

----------------------------

>PpulchraPlasmids|OMJ13215.1

AP--AKKKAYRLASMSFFLT---YPKC---TL----S-KETVLAELKKIC------DVET

YIVA----RELHSDGT-----PHIHAYLKVKKTINSKNPR-YFDIKG--YHG--NYDSCR

--NPKNVMKYV---------MK------D-----K-NFIT-----------NSTESQA--

--KKS-------------------------------------------------------

------------------------------------------------------------

------------------------------------------------------------

------------------------------------------------------------

------------------------------------------------------------

----------------------------

>PpulchraPlasmids|OMJ11569.1

TR--RRRTGFRIAGLSFFLT---YPKC---TL----S-KDAVLAELKKKY------DFES

YIVA----KELHEDGS-----PHIHVYLRFSTVINRINPR-CFDILG--FHG--NYQTCK

--HVYKVMHYI---------MK------D-----R-DFLT-----------NMHPSEY--

--KKTGLDAVKR-VF-EAE--SYEEAIEIVK-----NDY---------------------

---------------------------------------------NLGR-----------

------------------------------------------------------------

------------------------------------------------------------

------------------------------------------------------------

----------------------------

>PpulchraPlasmids|OMJ09562.1

SS--RSSGNFRLEGKSFFLT---YPRC---NL----D-LNVILTELEKIM------SFDK

YIIA----REKHASGE-----PHIHVFLSRFEKKATRNPK-FFDILG--FHG--NYQTCR

--SSKSVMAYV---------AK------D-----K-DYIT-----------NMPESAF--

--PHLKKSAVQ--YF-EAE--TVEEALEVAKNDLG-RDYIRNAELFEKSARYLKERPVEP

----FDPSHRFIPPHGIRKW-------DR-----TKQALMLFGKTGTGKSSYAETLFNN-

-------PLVVNSL--------EDLKRLRP-VHDGIVFDEFN-PLKLPRETMIMLTNVE-

--FHSTVSVKHGS-VTIPRLL-PRVFL-TNVKIF-------------NDP--AIVR----

--RV-KTIPVNKD--VILIDSDDDTESD-----RLYFY----------------------

----------------------------

>PpulchraPlasmids|ETO15557.1

QE--VENQNFRLQAKNLFLT---YPQC---TL----Q-PEEALAILETKL------QINE

YLIA----VENHIQKG-----RHLHCYINCKKKVDIRSAH-RLDLDG--FHG--NYQGCR

--SLTAVKKYC---------TK------E-----K-NYIT-----------NIYNFAI--

--TAPAVAAVK--AI-EGD---VNSAINIIIANLA-RDYIRDSLRIEQSIKRLAKSNDE-

-------NYKFKNVNNVLNW-------NR-----SKHVLWLHGKTNTGKTQFALSLFKN-

-------PLLVRHI--------DQLKKLDN-SYDGIVFDDMN-FSYWKREEQIHIVDVE-

--ETTAVNVKHGH-VEIKKGL-PRVFT-SNNRIF-------------NDP--SIKR----

--RI-RYIKID-D--IRDISENEPEKSN-----DSVFES-ED------------------

----------------------------

>CRESSV6-Wastewater|AUM61713.1

KS--KKKKEFRINSQQFFLT---YPQC---PL----D-PKVVLDHILIEE------PLEH

YVIA----QEQHKDGN-----MHIHAYLLFAKKLNVRNEK-KFDIVT--YHP--NMQGVR

--SWKNVVKYV---------TK------D-----G-NYLT-----------NYDDSVI--

--DKLIKDNMKV-LA-KES--KVSEALSVL-SKTV-RDLVIHGNAIQKNLRSLAVKRK--

-----APEYSIEDFRVDFVW-------DR------RRTLVMWGPTDTGKTSLAKALLPT-

-------SLFIRHI--------DRLKEYDEEEFEGLILDDMS-FKHWPREAQIHLVDIE-

--NDSHINVKHGM-AVIPAGT-PRIIT-TNNMPAEILL---------DDG--AIAR----

--RI-QIVHIE-S--VK-----------LESGD---FSW-VRERNS--------------

----------------------------

>CRESSV6-Wastewater|AUM61719.1

TS--KKPKTFRLNTQTLFLT---YPKC---KM----T-PEQALEQLQLIV------KIEV

YLIA----EEQHMDGT-----MHLHAYLRLDHKMNIKDER-KFDLEL--YHP--NMQGAR

--SPKAVIKYV---------CK------D-----G-KYVT-----------NMSQATI--

--DEAIHNNVKV-KA-KES---FEEGMKVL-SKTF-RDLAIHGEAIERNMRARKKQKT--

-----EPKFSLDLFKVSFEW-------DK-----TK-TLVLWGPTNTGKTSLAKALLPN-

-------ALFIRHI--------DALKEYKTGQYTGLIFDDMS-FKHWPREAQIHLLDTD-

--NVSQINVKHSI-AIIPEGT-PRIIT-TNMMPAEILA--LNI----EEC--ALSR----

--RV-QLEWIE-R--VY-------------------------------------------

----------------------------

>CRESSV6-Wastewater|AUM61624.1

TQ--SKPKEFRLNSQQLFLT---YSQC---PI----N-LELILNHLKSLV------EIDK

YIVA----QEKHQDGN-----LHIHCYLLLKSKLNLKNPR-KFDYQE--YHP--KVEGCR

--SYKNVIKYV---------TK------D-----G-NYIT-----------NYERDIL--

--EKIINDNKKA-LA-VED--KVEEGMKIL-AKTA-RDLCIHGNTIRRNLESLVPKRR--

-----KIVFALSDFRIDFEW-------DR------TKTLILWGPTNTGKSSLAKALLPD-

-------SLFVSHI--------DRLRDYNSKSYTGIIFDDMS-FKHIPRDGQLHLVDNY-

--DDRDIHCRYAP-AFIPAGT-PKIIT-SNNPPSEILL---------LDP--AIAR----

--RV-QIVNIE-Q--VWGNPNPDTTTTTSRSNSGGDFEW-VKERFTSEDAERPA------

----------------------------

>CRESSV6-Wastewater|AUM62043.1

TQ--SKPKEFRLNSQQLFLT---YSQC---PI----D-LELILNHLKSLV------EIDK

YIVA----QEKHQDGN-----LHIHCYLLLKSKLNLKNPR-KFDYQE--YHP--KIEGCR

--SYKNVIKYV---------TK------D-----G-NYIT-----------NYEKDIL--

--EKIINDNKKA-LA-VED--KVEEGMKIL-AKTA-RDLCIHGNTIRRNLESLVPKRR--

-----KIVFPLSDFRIDFEW-------DR------TKTLILWGPTNTGKSSLAKALLPD-

-------SLFVSHI--------DRLRDYNSKNYTGIIFDDMS-FKHIPRDGQLHLVDNY-

--DDRDIHCRYAP-AFIPAGT-PKIIT-SNNPPSEILL---------LDP--AIAR----

--RV-QIVNIE-Q--VWGNPNPD--TTTSRSNSGGDFEW-VKERFTSQDAERPA------

----------------------------

>CRESSV6-Wastewater|AUM61738.1

SS---PKKQWRLNTRTLFLT---YPKC---NL----T-VELLLEKLEAPT-------VQD

WCIA----REEHQDGT-----PHLHCYLKLSRKVNIKDPS-KLDLKE--YHG--NYQGCR

--SPKCVLKYV---------TK------G-----G-KYIS-----------NMGE--V--

--EEHLK-KMEN-LA-KEG--KGAEAVNLLAPKGA-RDLLLYGDLIQKSMAKLKKAKSP-

-----QINFKLEDFSLDWTW-------DV-----TK-TLVLTGKTNLGKTTLSHCLIQEV

-------ALLIRHM--------DRVKEYDSNLWGGIIFDDMS-FKHLHREAQIALTDTA-

--FDTDIHVRYSV-ATLPRET-PRIIT-TNLDPYLILD---------YDE--AIRR----

--RC-QLVEVS-N--------------------NGVFSY-KNIQ----------------

----------------------------

>CRESSV6|KM510189

FD--AVLKGFRLSAKRYLLT---YAQC---PE----D-PDDIFRQLDRKR------AIAR

AVGC----IELHADGN-----PHIHIAVEFAKKLNSVDTK-YFDYES--YHP--NISPAQ

--SWPKCINYC---------RG------K-----N-KTLT--D--------ASGIRGP--

--KPDLFVQARA-GN-KEL--WLQWLFEHN-----------VQPTLGLEVWRSLHAPPAF

-ETREIAATFDQRLLYLQLP-------DD-----FTKSVVIIGPSGSGKTVWAFNRMFER

-------GLSVGDI--------DDLKLFRPEQHSCIVFDEVR-FGRFKLETQIALCDTA-

--RSRTIRCRHGN-GFIAANT-PRVFTCTGTLPF-------------QDY--QIER----

--RI-HIINLY-D--LWPPTF---------------------------------------

----------------------------

>CRESSV6|KP005454

TQ--ATAKSVRWSAKKFLLT---YSQC---CE----D-PEDIFSRIDALR------KVKR

AVGC----IELHQDGN-----PHIHIALEFETKLNTTNCR-YFDYKN--FHP--NVTVAD

--SWPKCINYC---------RG------K-----N-KTLI--ELYH-----AAEGGKR--

--KYDLFAVAAG-DN-QRA--WTQWCYEHD-----------ISPLWKSDVWRQLRTPVGR

-DEDARPTTIDTRFAFMVLP-------EH-----FKQSVVLLGPSNCGKTTWAVNHMVER

-------ILIVNEI--------DDLRRL-DATHKGIVFDEIR-FTKWPLHSQIALVDNA-

--LGRSIHCRNIN-AWIPKGT-PKLFTCTEKFPF-------------YNY--QVYR----

--RL-NIINLY-E--LWVREL---------------------------------------

----------------------------

>PpulchraPlasmids|OLY79699.1

ST--SSNNSFRLSGKKAIVV---YPGI---LT----D-RDLVLNELKKDR------PIQS

WCIN----LVNEENGE-----INMHVFLEFSTKVNIRNEG-YFDILG--IHG--IAKTVK

--DKTPVLRKI---------FR------N-----D-DFLT-----------NIKSDLV--

--EEFAGTPTE--IL-RSK--SLQNSLKILLRILS-RSLLMNPSRLFENIRKLTPRKNS-

-----ELNLRFREVPQISIW-------DR-----KKSSLWLKGPPGTGKTTFAKSLFNN-

-------PLIVNIN--------EALLNFNI-FHDGIIFDDMN-FTKYTVEFQIHMTDVT-

--DEKVIKIGNSK-INIPAET-PRVFT-SNVPIF-------------SEY--RVRR----

--RV-ALIKIN-K--LQLTEDGREFE---EPNKESEFHQ-V-------------------

----------------------------

>PpulchraPlasmids|AAF36424.1

LK--KKKPSFRLNARLFFLT---YPQS---GL----T-KELILRELRKIV---S--DIHT

VVVS----KERGEDGY-----DHFHVLLEAKTKKNYKDPR-CFDILG--VHG--KYETVR

--NRKRSLKYI---------CK------E-----G-DVVS-----------ENVD--V--

--TKALLSAFKK-VF-KCK--YLDENPSRLIFEIY-VDYLSSPKRYEKFIAEYRKDSTI-

----PRLWIHKLKMSELMDWAEKIVE-GV-----VEKSLYIHGKPGIGKTNMARLMFND-

-------IAIIKHL--------DKLKEASVDQSVAIGFDDVN-LKKYTREDCINIVDGE-

--VGSQIDVKYGM-VVLEPYV-PKVFI-SNLLPEMVYK--G------YDK--AVER----

--RL-RIIYLEQA--IYKHERDVPLESK-----ESTFGF-LVEWVQKKRGL---------

----------------------------

>PpulchraPlasmids|AAF36423.1

TK--KGNGSFRLNAKIMFLT---YPQS---GL----T-KEVILQELKKKI---F--DIHS

VVMS----KERGEDGY-----DHFHVLLETKTKKNYKDPR-CFDILG--VHG--KYESTR

--NKKRAMKYI---------CK------E-----G-NVLC-----------EGIE--L--

--GSALLSTFKK-MF-RCR--YLGENPSELIFEIY-NDYLISPKKYKQYILESVKVSSS-

----PRLWIHKLKMDELMIWAKTIVS-DH-----ISKSLYIHGAPGVGKTNMARLMFDD-

-------MFIVKHV--------DKLKEADVESSVAIGFDDVN-LNKYTREDCINIVDPE-

--IGSQINVKNSM-ISLEPYV-PKVFI-SNFLPERVYK--G------YDE--AVER----

--RL-KVICLEQA--IYKREKDVPLESK-----ESTFKY-LVDWVQGKRGL---------

----------------------------

>PpulchraPlasmids|AAF36422.1

SN--RSKKSFRIAAKTFFLT---YPQS---GL----T-KELILLELGKII---T--DIHS

VVMS----KERGEDGY-----DHFHVLLEAKSRKNYKSPR-CFDILG--VHG--KYETVK

--SKKKALKYI---------CK------E-----G-DVLS-----------EGVD--V--

--ESALLSTFKK-MF-RCR--YLSENPSVLIFEIY-NDYLISPKKYKQYILESVKVSTS-

----PRLWIHKLKMDELMIWAKTIVS-DH-----ISKSLYIHGASGVGKTNMARLMFDD-

-------MFIVKHV--------DKLKEADVESSVAIGFDDVN-LNKYTREDCINIVDPE-

--VGSQINVKNSM-VSLESYV-PKVFI-SNFLPERVYK--G------YDE--AVER----

--RL-KVIYLEQA--IYKHEKDVPLESK-----ESTFKY-LVDWVQGKRGL---------

----------------------------

>Circo|YP_007974237.1

MA-KKRSGPPP--AKRWVFTLNNPSP-------------KEIDH-ILGQ--DMN--MFDY

LVC----GKEM---G--EGKTPHLQGFANFSKKKTFNQVKKIFG-------SRCHIEKAK

-GTDKDNQQYC---------TK------E-----G-DVVEA-GAP-RS-------GGQ--

--RTDL-ASAVST-VLES--GSLRAVAE---S-HP-VQFVRYHRGLSE-LLKVA------

-----------G-KAKARDWKT--------------SVHVIIGPPGCGKSKWACNFAEP-

-----EVTF----WKPRN-KWWD----GYCGQ-EVVILDDFYG--WLPYDEMLRLCDRYP

LT----VETKGGTVP-F---LARTILITSNKVPQEWYSSES-----VP-TAEALFR----

--RITTLQLWKTTTT-------------------EKFV-DPPC-----------------

--EEFPYE---IN---------------

>Circo|AAZ78351.1

MP-NGRSGPQP--HKRWVFTLNNPSE-------------DERKK-IREL--PIS--LFDY

FIV----GEEG---N--EEGTPHLQGFANFVKKQTFNKVKWYFG-------ARCHIEKAK

-GTDQQNKEYC---------SK------E-----G-NLMEC-GAP-RS-------QGQ--

--RSDL-STAVST-LLES--GSLVTVAE---Q-HP-VTFVRNFRGLAE-LLKVS------

-----------G-KMQKRDWKT--------------NVHVIVGPPGCGKSKWAANFADP-

-----ETTY----WKPRN-KWWD----GYHGE-EVVVIDDFYG--WLPWDDLLRLCDRYP

LT----VETKGGTVP-F---LARSILITSNQTPLEWYSSTA-----VP-AVEALYR----

--RITSLVFWKNATE-------------------GQFV-SPPC-----------------

--PEFPYESSTVF---------------

>Circo|AIF76280.1

MV-RRRSGPPP--HKRWCFTINNPTP-------------AEERH-LREI--PVN--QVDY

LIA----GREV---G--AQGTPHIQGFVNFVKKKTLNQVKLFVG-------PRAHVEKAR

-GSDEQNRDYC---------SK------D-----G-DLVDV-GCP-RR-------PGA--

--RHDL-ASAVST-ALST--GDLGQVAQ---S-HP-ETYVKYHRGLAE-LIKAS------

-----------N-KLPRRAWKT--------------EVHVHVGPPGCGKSKWASEFADP-

-----EVTY----WKPRN-KWWD----GYASH-EVVILDDFYG--WLPFDELLRICDRYP

LE----VEVKGGLVP-F---LARTVIITSNKMPQEWYSSDA-----VP-HAEALYR----

--RITTLISWQTAMQ------------------APQYLPTCPC-----------------

--SEFPYE---IN---------------

>Circo|KJ641742

MV-RRRSGPPP--HKRWCFTINNPSP-------------AEERH-LREI--PVN--QVDY

LIA----GRER---G--AQGTPHIQGFVNFVKKKTLNQVKLFVG-------PRAHVEKAR

-GSDEQNRDYC---------SK------E-----G-DLVDV-GCP-RR-------PGA--

--RHDL-ASAVST-ALST--GDLGQVAQ---S-HP-ETYVKYHRGLAE-LIKAS------

-----------N-KLPRRAWKT--------------EVHVHVGPPGCGKSKWASEFADP-

-----EVTY----WKPRN-KWWD----GYASH-EVVILDDFYG--WLPFDELLRICDRYP

LE----VEVKGGLVP-F---LARTVIITSNKMPQEWYSSDA-----VP-HAEALYR----

--RITTLISWQTATQ------------------APQYHPNCPC-----------------

--SEFPYE---IN---------------

>Circo|AGL09969.1

MEM----AGHP--CERYCFTINNYSE-------------EDIEA-VKAFL-VPD--NAEY

AIV----GKEK---G--ENGTPHLQGFVNLKKKMRFNPFKAALG-------GRAHIEQAR

-GTDLDNKRYC---------SK------G-----G-DLLEV-GEP-GK-------QGK--

--RSDL-KEAVTL-LNSG--ANMTAVAR---A-YP-EVFIRYGRGLRD-YVITA------

-----------G-LSQQRAWKT--------------EVHVIVGVPGVGKSRHVSEQH---

-----SDIY----WKPRG-KWWD----GYCNQ-EVVCLDDYYG--WIPYDDLLRLCDRYP

LR----VETKGGTVS-F---VAKKIYITSNKQIKDWYNFEE-----LKVDPRALYR----

--RVTSYKVMREGG-------------------------YDVV-----------------

--MTGDNK---IN---------------

>Circo|YP_009021891.1

MIM----AGHP--CKRYCFTINNYLP-------------EDEAA-VKEFL-TEA--NCVY

AVV----GKEV---G--ESGTPHLQGFCNLKKKMRFEPFKRAIG-------GRAHIEQSR

-GTDVDNKRYC---------SK------G-----G-DLLEV-GEP-SA-------QGK--

--RSDL-KEAVTL-LNNG--GTMTDVAR---A-HP-ETFIRYGRGLRD-YVIQA------

-----------G-LTKPRAWKT--------------EVHVIVGPPGVGKSRHVQETAGE-

-----NALY----WKPRG-KWWD----GYTGQ-SHVVLDDFYG--WLPYDDLLRLCDRYP

LR----VETKGGTVE-F---VAKVIWITSNKQVKDWYDYEE-----LKVDARALYR----

--RLTTYQVMRQGG-------------------------YNVQ-----------------

--MTGDNK---IN---------------

>Circo|AKO84203.1

MA-RDSRRGNP--IRRWCFTINNPTP-------------EEEDA-VKN---LAP--DAKY

LIC----GREV---G--ENGTPHLQGFVNLKKTTRMGALKSRLG-------GRGHFEPAR

-GDDCSNQDYC---------SK------G-----G-DLIES-GEP-CK-------QGK--

--RNDL-HDAVST-LKET--KSLAAVAA---A-HP-ETYVKFSRGLRE-LLLIS------

-----------E-MATPRKWKT--------------EVNVLVGPPGCGKSRYCLETA---

-----PDAY----WKPRG-KWWD----GYDGH-SDVILDDFYG--WLPFDDMLRLCDRYP

LR----VETKGGTMN-F---IGRRIFITSNKLPHEWYNDE-------IGNKAALYR----

--RLTSVTVWDGGN-------------------------PVPQ-----------------

--FMFPYQ---IN---------------

>Circo|AIF76265.1

MK-----ASTP--AYRWCFTINNWTS-------------EEYGL-IEST--LRS--SVKY

AII----GKEV---G--EKGTPHLQGFANFKKKLRLSTLKKLPGF------TRAHVERAK

-GTDLQNQKYC---------RK------G-----N-TYLEI-GCP-SS-------QGK--

--KPEL-TVAVRK-LESN--GDLVKIAK---E-HP-EVYIRHGRGLRD-YVNTA------

-----------G-LVGQRAWKT--------------YVVVIVGEPGVGKTRYVNSECKE-

-----CSVY----WKPRG-PWWD----GYAGQ-EAVIFDDFYG--WIMFDELLRVCDRYP

LK----VPVKGAFVE-F---VAKKVYFTSNKPPEEWYDKEN-----IRGNIEAFFR----

--RINEYLVIKGEV-------------------------DG-------------------

---TPMYE---IN---------------

>Circo|AIF76253.1

MR-SRGRGAVP--VYRWCFTLNNWTE-------------EEYGL-IEST--LRS--SVKY

AII----GKEV---G--ESGTPHLQGFANFKKKLRLSTLKKLPGF------TRAHVQPAN

-GTDLQNQKYC---------QK------E-----K-NYLEI-GLP-SR-------RGR--

--SGEL-SKAVDL-LKNG--GNLSEVAD---V-YP-EVYIRHGRGLRD-YVTAA------

-----------C-LSGQRDWKT--------------RVIVIIGQPGVGKTRYVNSQCKG-

-----HSVY----WKPRG-PWWD----GYTNQ-ECVVFDDFYG--WVTFDELLRVCDRYP

LK----VPVKGSFVE-F---VAKFIYITSNEPPENWYDKEN-----IRGKIEAFFR----

--RIDEYLVIEGEE-------------------------DG-------------------

---EPVYE---IN---------------

>Circo|AIF76248.1

MS-SGRRRLAP--AYRWCFTLNNWTE-------------EEYGL-IEST--CRS--LAKY

LII----GKEV---G--ESGTPHLQGFVNFKKKLRLSALKSLPGF------TRAHVESAR

-GTDVENQKYC---------RK------Q-----G-AYLEI-GVP-SS-------RGK--

--SSTL-TEAVAT-LQNN--GDLRAVAQ---M-YP-EVYIRHGRGLKD-YVMTA------

-----------G-LVGQRSWKT--------------HVTVLIGRPGVGKTRYVNREVEG-

-----KQVY----WKPRG-PWWD----GYIQQ-EVVVFDDFYG--WVTFDELLRVCDRYP

LK----VPVKGAFVE-F---VARAIYFTSNKPPEEWYDKEN-----IKGSIEAFFR----

--RVNEYLVVEDGE-------------------------EG-------------------

---APAYE---IN---------------

>Circo|AIF76261.1

MLAMGMARGSP--ACRWCFTINNWTE-------------EEYGQ-ICSF--TED--KVKY

LII----GKEV---G--REGTPHLQGFVNFKKKLRRDTLKKLPGF------TRAHVEPAK

-GSDMENQRYC---------RK------Q-----G-KYLEI-GSP-GL-------QGK--

--SSAL-QEALKT-LENR--GDLAAVAT---A-YP-EVYVRHGRGLRD-YVNTS------

-----------G-LVPRRQSKT--------------VVTVVIGPPGVGKTKYVNDCIGD-

-----LTSY----WKPRG-PWWD----GYQQQ-ECVVLDDFYG--WVPFDELLRVMDRYP

LK----VPVKGAYCE-F---NSKKLFITSNKPPEEWYNEEN-----ICGTLQAMFR----

--RFNEVFRMKSGG-------------------------EP-------------------

--LTPQHE---IN---------------

>Circo|YP_009170674.1

MNK-KKGDETP--GRRWCFTINNYGA-------------PDLEC-VNEF--RCE--DVVY

AIC----GKEK---G--AKGTPHLQGFIHFTGNWRFNRVRNLLG-------GRAHIEKAR

-GNDDQNKAYC---------SK------E-----E-TYLEV-GTP-QF-------QGK--

--RNDL-GRVASA-LESG--ATLSEVAR---A-SP-EVFIRYGRGLRD-YMNVR------

-----------G-LVKPRDFKT--------------EVIVLVGEPGSGKSKYANELP---

-----GSKY----WKPRG-QWWD----GYNGE-DIVVLDDFYG--WVPYDELLRIGDRYP

LK----VQVKGAFVE-F---TSKMLVITSNKRPEEWYDKEK-----IA-DQSAMWR----

--RFDKMYYCERGEP---------------------------------------------

--KAYPHEWKEFE---------------

>Circo|ADD62475.1

ME-KRKKRQNP--AKRWCFTLNNYTQ-------------LEAIT-IEQLL-CTE--EVQY

AIV----GEEI---G--ENGTPHLQGFFNLKKKKRLTSLKAWLN-------DRAHYEEAK

-GSDEQNRRYC---------SK------S-----G-NLISF-GSP-QK-------QGQ--

--RNDL-VLAAEL-LESG--GNMAAVAD---L-YP-SAVIRYGRGLQQ-YWQLI------

-----------G--FSARDFKT--------------EVFVYVGPPGCGKSRAAAELGAAS

----GGKVY----YKPRG-EWWD----GYNGE-ATVIIDDFYG--WLKYDELLRLCDRYP

HR----VPVKGGFVQ-F---CSKRIILTSNIHVWMWYRFES-----Y--DASALMR----

--RINVYKLWNGS--------------------VCTFDDEKYN-----------------

--FLTPLK---YN---------------

>Circo|YP_004376332.1

MPTDAPRREQP--VKRWCFTLNNPTA-------------EERRH-IQEII-TAD--AVDF

AVI----GNEV---G--DSGTPHLQGFLNMKTKRRLGTMKKWFN-------ARAHYEAAK

-GTDLQNDEYC---------TK------G-----G-DYLRI-GEP-GK-------ERC--

--RNDL-QKAIDV-VRSS--GSMRAVAE---A-CP-ATFIRYGRGLRD-YANVM------

-----------Q-YRKPRDFKT--------------EVNVYVGDPGCGKSRKASELCAG-

-----TTVY----YKPRG-MWWD----GYDGQ-ENVIVDDFYG--WMPCDELLRVFDRYP

CK----VPVKGAYVE-F---VSTNIYVTSNKHVWQWYKFEG-----F--DPAAVMR----

--RVNVYLVYDNA--------------------GERFVN---------------------

--ESAMYD---VT---------------

>Circo|YP_009091696.1

MPQRPPKKEAP--HKRYVFTLNNYTT-------------EEYAR-IDNVG-ADG--LARY

MIT----GKEV---G--ENGTPHLQGFINLKVKKRFSQIKEMLG-------SRCHIEKAR

-GTDLENRVYC---------SK------E-----G-SFQEY-GSP-VG-------QGK--

--RSDL-DAAAET-LRTSL-GDLRSVAE---L-YP-SQFIRYGRGLRD-YASVL------

-----------G-LVKPRDFKA--------------NVTVITGPPGCGKSRYAADHAS--

-----GTPY----YKPRG-DWWD----GYHTN-ATVILDDFYG--WIKLDEMLRICDRYP

HQ----VPVKGGYVQ-F---LARDIFITSNKPVEEWFP--N-----C--DCSALFR----

--RINVYLTWNDE--------------------------ER-------------------

--IDTPYE---IN---------------

>Circo|YP_009000900.1

M------SKVS--VRRVCFTLNNYTE-------------EDLKF-IEEH--FKS--VSKY

LIV----GKKV---G--ENGTPHSQGFINLKKKTHFNSVKKLL--------PRAHIEKAK

-GNDQHNKDYC---------SK------Q-----E-VAFEH-GQI-QG-------QGK--

--RNDL-ADALAT-VANG--GDIKALED---S-HS-LVYAKYKRGILA-CIDDF------

-----------G-WKKPRDWKT--------------EVHVLWGIPGCGKSRYAREQA---

-----PEAY----YKPRG-EWWD----GYTGQ-EDVILDDFYG--WLKFDELLKICDRYP

YR----VPVKGSFVQ-F---TAKRIYITSNVAASEWYHFQG-----Y--DPTALYR----

--RMTSYLTWNDM--------------------CKEFRP---------------------

---------ININ---------------

>Circo|AFL02442.1

MTPMPSKEGSG--CRRWCFTLNNPTD-------------GEIEF-VRTL--GPD--EFYY

AIV----GREK---G--EQGTPHLQGYFHFKNKKRLSALKKML--------PRAHFERAK

-GSDADNEKYC---------SK------E-----G-DIPTL-GIV-AR-------DGH--

--R-AF-DGAVAA-VMTG--CKMKEVAR---E-FP-HIYVRHGRGLHN-LSLLV------

-----------G--SRPRDFKT--------------EVDVIYGPPGCGKSRWANEQP---

-----GTKY----YKMRG-EWWD----GYDGE-DVVVLDDFYG--WLPYCEMLRLCDRYP

HK----VPVKGAFVE-F---TSKRIIITSNKPPETWYKED------C--DPKPLFR----

--RFTRVWWYNIDK-------------------------QVRP-----------------

--DLLAHP---IN---------------

>Circo|ADU77009.1

MAPRRPPREAA--AKRWCFTLNNPTE-------------DEIKS-LDSW--LLS--EFHY

AIV----GKEV---G--EQGTPHLQGFIHLKQKKRLSQLKQLF--------NRAHWEKAR

-GSDEDNEKYC---------SK------E-----G-NLLTL-GIP-AR--------GN--

--RSDL-SGAVAA-VKAG--RAMTEVAR---E-FS-EIYVKYGRGLRD-LKLLI------

-----------G--QQPRDFKT--------------EVIVITGPPGCGKSRWAAEYP---

-----GSKF----YKMKG-EWWD----GYDHQ-DVVVIDDFYG--WLPFCELLRVTDRYP

HK----VPVKGAFVE-F---TSRVIIVTSNSPPDAWYSEEK-----C--CVQALFR----

--RINKWLVWNHDK-------------------------DAPD-----------------

--CMKKYP---IN---------------

>Circo|YP_764455.1

MP--PQKREAA--AKRWCFTLNNYTD-------------EEVSA-VKAW--NAS--EYHY

AVV----GREK---G--ENGTPHLQGYIHLKKKARLSTLKKLL--------SRAHWEKAR

-GSDSDNEAYC---------TK------D-----G-DILTL-GMP-VE--------GN--

--RSDL-SGAVAA-VKAG--SRMVDIAR---E-FS-EVYVKYGRGLRE-LALLI------

-----------G--QKPRDFKT--------------EFIVVTGPSGVGKSRYANEYP---

-----GTKF----YKMKG-DWWD----GYSNE-DVVVIDDFYG--WIPFCELLRLTDRYP

HK----VPVKGSYVE-F---TSKVIIVTSNTHPDSWYNEEK-----C--YLPALFR----

--RINKWLTWNAIR-------------------------DAPD-----------------

--CMKKYP---IN---------------

>Circo|KU230452

MP---PVRAAP--AKRWCFTLNNPTD-------------EEVQK-IKDM--NPS--EYHY

AIV----GKER---G--EQGTPHLQGFLHLKCKKRLNQLKEFL--------ARAHWEKAR

-GSDEDNEAYC---------SK------E-----G-EILTL-GMP-AK--------GN--

--RSDL-SGAVAA-VKAG--RAMSEIAR---E-FS-EVYVKYGRGLRD-LALLI------

-----------G--QKPRDFKT--------------DVILITGPSGVGKSRWAFEYP---

-----GSKF----YKMKG-EWWD----GYANE-DVVVIDDFYG--WLPFCELLRLTDRYP

HK----VPVKGSYVE-F---NSKTIIITSNTHPETWYNEEK-----C--YLQALFR----

--RINKWMFWDAMR-------------------------DAPD-----------------

--CVKKYP---IN---------------

>Circo|YP_009134739.1

MP--KQARSSP--CKRWVFTLNNPTE-------------QEVES-VKSL--PPS--EYHY

AIV----GKEK---G--EQGTPHLQGFLHLKKKVRLNQMKQLI--------PRAHFEIAR

-GSDEDNEQYC---------SK------E-----G-DILTI-GAP-AK--------GN--

--RSDL-AGAVAA-VKAG--RPMTEVAR---E-FS-EAYVKWGRGLKD-LALMI------

-----------G--QKPRDFKT--------------EVIVLTGPSGVGKSRWANEQE---

-----GTKY----YKMKG-DWWD----GYSNE-DIVVIDDFYG--WIPFCELLRLCDRYP

HK----VPVKGSYVE-F---TSKKIIITSNTHPNHWYNEEK-----C--YMQALFR----

--RINKWLFWDVFG-------------------------DAPD-----------------

--AVKKYP---IN---------------

>Circo|NP_573442.1

MA---PVRAAA--AKRWCFTLNNYTA-------------EEEAK-VRAL--LPG--EFHF

AIC----GKER---G--EQGTPHLQGFLHFKKKQRLSALKKLL--------ARAHWEKAR

-GSDHDNEEYC---------SK------E-----N-DILTI-GEP-VQ--------GN--

--RSDL-AGAVAA-VKAG--RRMVDIAR---E-FS-EIYVKYGRGLRD-LALMI------

-----------G--QKPRDFKT--------------EVVVITGPSGVGKSRLASEME---

-----GSKF----YKMKG-DWWD----GYSNE-DIVIMDDFYG--WLPFCEMLRLMDRYP

HK----VPVKGSYVE-F---TSKKIVITSNTHPESWYCPDK-----C--YLPALFR----

--RINKWMYWDGLR-------------------------DVPD-----------------

--AMKKHP---IN---------------

>Circo|YP_803546.1

MRPMAARRDSG--ARRWCFTLNNYTP-------------EEEET-ARNLIHDAD--KYAF

AII----GKEV---G--ESGTPHLQGFMHFKQKQRLTALKKLF--------PRAHFEKAR

-GSDQQNADYC---------GK------D-----G-ELTMI-GTP-SD--------NN--

--PSDL-AGAVAA-VKRG--SQMSEIAR---E-FS-EVYVKYGRGLRD-LRLLI------

-----------G--CPPRDFKT--------------EVIVLIGPPGCGKSKLANEME---

-----GSKF----YKMKG-DWWD----GYDNQ-DIVIIDDFYG--WLPYCECLRLCDRYP

HR----VPVKGAYVE-F---TSKKIVFTSNRHVDGWWKGE------I--EKSAFYR----

--RINVYKFYETGE-------------------------DMPG-----------------

--HMLPHP---IN---------------

>Circo|AEL28794.1

MPP-AEKRDSA--AKRWCFTLNNPTE-------------QEVQT-CSRF--VEA--EFHF

AIV----GREV---G--EQGTPHLQGFLHFRAKKRLSALKKLL--------QRAHWEKAR

-GSDAENKEYC---------SK------E-----G-NLTMV-GHP-CG-------ANT--

--TSDL-AEAVAA-VHAG--RRMIEIAR---D-FS-EAYVKYGAGLHR-LHLMI------

-----------G--SRPRDFKT--------------EVTVLWGPPGKGKSRWAADLP---

-----GEKY----YKMKG-DWWD----GYTGE-EVVVIDDFYG--WLPYCELLRLMDRYP

HK----VPFKGGYME-F---TSKHIVITSNSPPNEWYKSIE--------NKAAMYR----

--RFTRVLTWYPEE-------------------------GAPGLR---------------

--TTLPYP---IN---------------

>Circo|ABU48445.1

M---AKKSDYG--YKRWVFTINNPTF-------------EDYVS-VIEFC-TAE--NCKF

AIV----GEEK---GE-KEGTPHLQGFLSLRKNARAAALEENLG-------GRAWLSRAV

-GSDEENEEYC---------SK------E-----T-TYLRV-GTP-NR-------KGR--

--SSDL-NAAASE-VLAG--ALMTDVAR---K-YP-TTYIMFGRGLER-LRQLI------

-------------VETPRDWKT--------------EVIVLIGPPGSGKSRYAFEFPA--

-----ERKY----YKARG-KWWD----GYEGN-DVVVMDDFYG--WLPYDDLLRICDRYP

IR----VEYKGGMTQ-F---VAKTLIITSNREPREWYKCE------V--DCTALYR----

--RIDRYLVMTPDG-------------------------DAPE-----------------

--FMLPYK---IK---------------

>Circo|AHK80894.1

MA-MAKSGNYS--YKRWVFTINNPTF-------------DDYVH-VLEFC-TLD--NCKF

AIV----GEEK---G--ANGTPHLQGFLNLRSNARAAALEESLG-------GRAWLSRAR

-GSDEDNEEYC---------SK------E-----S-TYLRV-GEP-GG-------KGR--

--SSQL-ADATSA-VLAG--LPLTDVAR---K-YP-TTYVIFGRGLER-LRHLI------

-------------VETQRDWKT--------------EVIVLIGPPGTGKSRYAFEFPA--

-----ENKY----YKPRG-KWWD----GYSGN-DVVVMDDFYG--WVPYDDLLRITDRYP

LR----VEFKGGMTQ-F---AAKTLIITSNKEPRDWYRSE------F--DLSALYR----

--RINKYLVYNVDK-------------------------PAPS-----------------

--CSLPFP---IN---------------

>Circo|ADD62451.1

------MANRT--VRRFCFTWNDHPV-------------EAYEK-CEKF--IEK--FCKY

GIV----GEEY---AP-TTGMPHLQGFCNLNKPTRFSTIKKHLD-------NSIHIEKAN

-GTDEQNQKYC---------SK------S-----G-IFFES-GVP-NK-------QGQ--

--RNDL-QSLVEF-IHEKR-PTIRDIAT---E-HP-TTYIRYFRGIER-MLQLV------

-----------N-PIKQRDFKT--------------EVYYYWGPPGTGKSRRALEEAQAF

-NT--ESIY----YKPRG-LWWD----GYEQQ-DSVIIDDFYG--WIKYDEMLKIMDRYP

YK----VQVKGAFQE-F---TSKKIFITSNVDTDELYKFVG-----Y--TTAAFER----

--RITNKEYMA-------------------------------------------------

----------------------------

>Circo|ADD62455.1

------MANRT--VRRFCFTWNNYPD-------------TAYEK-CETF--IQK--FCKY

GIV----GEEL---AP-TTGTPHLQGFCNLRKPTRFSTIKKHLD-------NSIHIEKAN

-GSDEQNQAYC---------SK------T-----G-IFFEE-GSP-TK-------QGQ--

--RNDL-QLLVDD-VGHPD-IKTKDIAA---K-YP-STFVRYFRGIKE-LQRVV------

-----------H-PVAERMFKT--------------EVYYYWGPPGTGKSRRALEEAQAT

-GN--GSIY----YKPRG-LWWD----GYEQQ-DSVIIDDFYG--WIKYDELLKITDRYP

YK----VQIKGGFEE-F---TSKKIFITSNVDTDELYKFNG-----Y--NTAALER----

--RLTVKEYMS-------------------------------------------------

----------------------------

>Circo|ADU77011.1

------MPRQT--VRRFVFTWNNYPI-------------EAYDK-CEKY--LTK--FCKY

GIV----GEEI---AP-ETGTPHLQGFCNLHKPTRFSTIKKHLD-------NSIHIEKAN

-GSDIDNQKYC---------SK------S-----G-IFFET-GQP-SK-------QGQ--

--RSDL-AVVSNK-ILEGS-TTIRDIAT---Q-HP-ETYIRYFRGIRE-LQRLV------

-----------N-PISPRDFPT--------------QVYYYYGPPGTGKSRTALEEAKKI

-DP--TSIY----YKPRG-LWWD----GYTNQ-KCVIIDDFYG--WIKYDELLKICDRYP

YK----VQIKGGFEE-F---NSTHIWITSNVDTDLLYKFTG-----Y--CNAAFER----

--RITIKKYFA-------------------------------------------------

----------------------------

>Circo|AGJ74758.1

------MANAK--IRRFCFTWNNYPL-------------EAYTK-CEEF--IKN--KCKY

GIV----GEEI---CP-NTGTLHLQGFCNLHKPTRFNAIKRDLD-------NSIHIEKAN

-GSDIDNQTYC---------SK------S-----G-IFFES-GIP-SK-------QGK--

--RSDL-MQVVET-ITKSEVPTLEDIAT---K-HP-VEYIRYFRGIEK-LQSIL------

-----------R-PVCARRFKT--------------DVYYYWGAPGTGKSRTALEEAEKI

-TT--TSIY----YKPRG-LWWD----GYRQQ-TCVIIDDFYG--WIKYDEMLKIMDRYP

YK----VQVKGGFEE-F---TSKHIWITSNIDTDLLYKFSN-----Y--SNVAFER----

--RLTIKKHFIQLQ----------------------------------------------

----------------------------

>Circo|ADD62461.1

------MSNRT--VRKFCFTWNNYEF-------------DAYAK-CETF--LNN--FAKY

GIV----GEEL---CP-STGTPHLQGYVNLIKPTRFSTIKKHLH-------NAIHIEKAN

-GSDEQNQTYC---------RK------S-----G-IFFEK-GEP-IK-------QGQ--

--RTDL-QLLVKD-TMEPS-NTLKDIAT---K-HP-IAYIRYFRGIQE-LRRMV------

-----------L-PVPPRNYPT--------------EVRYYWGPPGSGKSRRALQEATEI

-ST--NGIY----YKPRG-QWWD----GYEQQ-SCVIIDDFYG--WIKYDEILKICDRYP

YK----VQIKGGYEE-F---TSTHIWFTSNVDTDLLYKFNN-----Y--INTAFER----

--RITIKEHIT-LS----------------------------------------------

----------------------------

>Circo|AKE49355.1

-------MNAN--VRRFCFTWNNYTE-------------EAYKK-CEDF--INT--YCKY

GIV----GEEI---AP-NTGTIHLQGFCNLHKPIRFNKIKKHLD-------NSIHLEKAN

-GSDEDNQKYC---------SK------S-----G-IYFEK-GTP-CS-------QGH--

--RSDL-QAVVET-IAQSN-STLQDIAT---K-HP-TAYIRYHKGIRE-YLRMV------

-----------K-PIQERQHKT--------------LVYYFWGPPGSGKSRRALEEAKAT

-N---QPIY----YKPRG-LWWD----GYNQQ-PNVIIDDFYG--WIKYDEMLKIMDRYP

YK----VQVKGGFEE-F---TSERIWITSNIDTDTLYKFIN-----Y--TNTAFDR----

--RITLKCHML-------------------------------------------------

----------------------------

>Circo|AGJ74756.1

-------MNST--VRRFVFTLNNYSE-------------RELNQ-ALFF--INN--YCKY

GII----GEEI---AP-TTETPHLQGFCNLTKPMRFSTIKKHLS-------DRIHIEKAA

-GSDKDNQKYC---------SK------A-----G-KFFEA-GRP-QS-------QGE--

--RNDL-AAVVDT-IQSG--ANIKSVAE---H-HP-AAFIKYHRGIKE-YIRTI------

-----------R-PVPVRDFKT--------------EVFYFWGEPGTGKSRTALAEAKGR

-AL--DSIY----YKPRG-LWWD----GYEQQ-ECVIIDDFYG--WIKYDEMLKIMDRYP

YK----VQIKGGFEE-F---TSKYIWITSNINTDDLYKFEG-----Y--VNTAFDR----

--RLSIKKHFN-------------------------------------------------

----------------------------

>Circo|YP_009110680.1

------MSNPT--VRRFVFTWNNYTS-------------EDYDK-CCEF--IKS--NCRY

GIV----GKEI---AP-TTGCPHLQGFCNLHKPMRFGAIKKHLH-------NSIHIEKAN

-GSDEDNKKYC---------SK------S-----G-ETFEQ-GHP-HK-------QGE--

--RTDL-QSCISD-IQSG--ATIKKIAE---D-HP-AVFIRYHRGIRE-YIRTV------

-----------M-PIKERDFKT--------------EVYYYWGPPGSGKSKRALEEAKQR

-G---TSIY----YKPRG-LWWD----GYQQH-DCVIIDDFYG--WIKYDEMLKIMDRYP

YK----VQIKGGFEE-F---TSKYIWITSNVDTDDLYKFIG-----Y--KTDALER----

--RITNKEYMD-------------------------------------------------

----------------------------

>Circo|AGJ74760.1

------MCNST--VRKFCFTWNNYTE-------------HDENK-CKDF--IAQ--YCKY

GIF----GKEL---AP-TTNTPHLQGYCNLSKPMRFSTIKKHLH-------NSIHIEKAN

-GSDEQNKEYC---------SK------S-----G-EIFEK-GTP-IK-------RGQ--

--RTDL-QSLLAD-IQDGN-RNIQTLAQ---S-HP-TTYIRYFRGIHT-YLNLV------

-----------H-PIAPRNFKT--------------DTYYYWGPPGSGKSRRALEEATAR

-CN--ESIY----YKPRG-QWWD----GYHQQ-EGVIIDDFYG--WIKYDELLKVTDRYP

YK----VQVKGSFEE-F---TSKHIWITSNVDTCDLYKFIG-----Y--CTDAIER----

--RITLKSYMS-------------------------------------------------

----------------------------

>Circo|YP_009021843.1

------MANCV--VRRFCFTLNNFTE-------------EEYEK-VTRF--IQD--YCKY

GIV----GNET---AP-TTGTIHLPGFCNLTKGMRFNNIKSKLA-------TRIHLEKAN

-GTDEQNQIYC---------RK------S-----G-TYFEK-GTP-VG-------QGK--

--RTDL-VSLVEG-IQNGQ-IRLSDIAK---D-HP-IAFIKYHRGIRE-YLQLT------

-----------K-PVQPRMFKT--------------WVYYYWGPTGSGKSSRALKEAMEI

-E---GEIY----YKPRG-LWWD----GYHQQ-DNVIIDDFYG--WIKYDEMLKIMDRYP

YK----VQVKGGFEE-F---TSRRIWITSNVDTDQLYKFIG-----Y--VSDAFDR----

--RITNKVYID-------------------------------------------------

----------------------------

>Circo|AEL87786.1

------MANSI--VRRFVFTLNNYTD-------------EHYTK-CCAF--ITD--HCKY

GIV----GKEV---G--EKGTPHLQGFCNLTKPMRFNAIKQHLH-------NTIHLEKAN

-GSDEDNQRYC---------SK------A-----G-NFFEK-GSP-VV-------QGQ--

--RSDL-SCVVDR-IRDG--KTITDIAT---E-FP-TQFIRYHRGIRE-LLQVI------

-----------K-PIPPRDFKT--------------YVYYYWGPPGSGKSRRALEEARAI

-DK--DSIY----YKPRG-LWWD----GYKQQ-KNVIIDDFYG--WIKYDEMLKICDRYP

YK----VQVKGGFEE-F---TSTRIWITSNVDIYSLYHFAG-----Y--NAEAIKR----

--RCTSIIEIN-------------------------------------------------

----------------------------

>Circo|YP_004152331.1

------MANAT--LRRFVFTWNNYNA-------------DDIEK-VVKF--IKE--KCKY

GIV----GEEI---AP-TTNTPHLQGFCNLKTPMRFKSIKTHLH-------TNIHIEKAK

-GTDLQNQEYC---------KK------G-----N-KWIEE-GTP-CT-------QGK--

--RTDL-ETLVSA-INSGT-NTQQKIAK---E-FP-ICYIKYHRGINE-YLKLI------

-----------H-PIEPRKEKT--------------WVYYYWGPPGTGKSRRALEEASQI

-NS--NSIY----YKPRG-LWWD----GYHQQ-ENVIIDDFYG--WIKYDELLKITDRYP

YK----VQIKGGFEE-F---TSKRIWFTSNVDTDKLYHFID-----Y--NNAAFER----

--RITCKIHIT-------------------------------------------------

----------------------------

>Circo|ADD62471.1

------MANST--VRRFVFTLNNYTE-------------DQYQK-CVEF--IST--KCKY

GIV----GKEV---GE-ENGTPHLQGFCNLHKPMRFGTIKKSID-------NAIHIEKAN

-GSDIDNQKYC---------SK------A-----G-NFFET-GTP-SQ-------QGR--

--RTDL-ESLVAG-IAAGA-NTTSKVAT---E-FP-AMYIKYHRGINE-YLKLA------

-----------F-PIKPRTEKT--------------WVYYYWGPTGSGKSRRALEEAQSI

-NA--DSIY----YKPRG-LWWD----GYHQQ-ENVIIDDFYG--WIKYDELLKITDRYP

YK----VQVKGGFEE-F---TSTRIWITSEKDTCDLYKYEG-----Y--NPASFER----

--RITCKINITYEK----------------------------------------------

----------------------------

>Circo|AIF76252.1

------MCDST--ARRFCFTLNNYTE-------------AEYQH-IVEF--INA--KCKY

GIV----GKEV---AD-TTGTLHLQGFCNLSKPMRFSAIKKSLN-------NRIHIEKAN

-GSDEQNQKYC---------SK------A-----G-NYFEK-GTP-AR-------QGQ--

--RTDL-DKLVRA-IQNGC-NTTQMVAK---E-FP-TAYIRYHRGISE-YLKLS------

-----------H-PIKPRDEKT--------------WVYYYWGPTGSGKSRRALEEARAI

-NP--DSIY----YKPRG-LWWD----GYHQQ-DCVIIDDFYG--WIKYDELLKICDRYP

YK----VQVKGGFEE-F---TSKRIWITSEKDTDRLYKFED-----Y--NPASFER----

--RITCKIFIDYKK----------------------------------------------

----------------------------

>Circo|AEL87790.1

------MANST--VRRFVFTWNNYCD-------------EDFVK-SCNY--IKE--FCKY

GIV----GKEL---CP-TTGTRHLQGFCNLIKPTRFSTIKQRLH-------NTIHIEKAN

-GSDEQNQNYC---------KK------S-----G-DFFEE-GNP-VT-------QGQ--

--RTDL-KLLVAD-IEQGI-TNIKDIAS---R-HP-TAFIKYHRGIDT-YLKLT------

-----------H-PIQPRNYKT--------------WVYYYWGPPGSGKSKRALEEAQSI

-NK--DSIY----YKPRG-LWWD----GYKQQ-ENVIVDDFYG--WIKYDELLKICDRYP

YK----VQIKGGFEE-F---TSTRIWFTSNVDTDLLYKFDN-----Y--NVAAFER----

--RITCKINIV-------------------------------------------------

----------------------------

>Circo|ADI48251.1

------MENKT--IRRFIFTWNNYTD-------------DDYDK-TCAF--IKG--NCKY

GIV----GKEH---AP-TTGVPHLQGFCNLSKPMRFSAIKKCLS-------NSIHIEKAI

-GSDEQNQKYC---------SK------A-----G-DFFEE-GLP-YT-------QGK--

--RTDL-EAVVRD-MEQGT-VRLEDIAA---K-HP-CAFIKYHRGIDQ-LLKLI------

-----------H-PIKPRDFQT--------------YVYYYWGEPGTGKSRRAKSEADAV

-NS--ASIY----YKPRG-LWWD----GYRQQ-ENVIIDDFYG--WIKYDELLKICDRYP

YK----VQIKGGFEE-F---TSKRIWITSNVDIYSLYKFEG-----Y--NAEAFKR----

--RITCMVEMNSNK----------------------------------------------

----------------------------

>Circo|ADD62453.1

-------MNST--VRRFCFTWNNYTV-------------ADTLT-VKDY--FVK--YCKY

GIA----GEEL---AP-ETGTPHLQGFCNLRKPQRFSAIKKHLS-------DRIHIEKAN

-GSDEQNQSYC---------KK------A-----G-HWFEQ-GVP-VK-------QGD--

--RSDL-KSVVST-IADGA-NTAQAIAE---K-HP-VEFIKYYKGISE-YLRLL------

-----------A-PIAPRKFKT--------------YVYYYWGPPGSGKSRRALEEAESL

-DP--DSIY----YKPRG-LWWD----GYRQQ-QSVIIDDFYG--WIKYDELLKITDRYP

YK----VQVKGGFEE-F---TSTRIWFTSNVDTDRLYRFDG-----F--TPEAFER----

--RITAKIHIT-------------------------------------------------

----------------------------

>Circo|ADY17982.1

-P-RMRRVNVT--IRRFVFTWNNYTP-------------SDFET-CITF--LDN--FCKY

GII----GKEK---CP-TTQTPHIQGFCNLSKPMRFNNIKKHLH-------NSIHIEKAN

-GSDEQNKIYC---------SK------S-----G-EFFEK-GHP-DK-------QGK--

--RNDL-DAVVLT-IQNGT-NTISNVAK---L-HP-ISFIKYHKGIKE-YINHV------

-----------N-PIRPRHYKS--------------EVYYYWGPPGTGKSKTALEKATAY

-NT--DSIY----YKPRG-LWWD----GYQQQ-TSVIIDDFYG--WIKYDELLKICDRYP

YK----VQIKGGFEE-F---TSKYIFITSNVDTCDLYKFRN-----Y--NTDAIER----

--RITEKIHFKNIF----------------------------------------------

----------------------------

>Circo|ADD62457.1

------MSNST--VRRFCFTWNNYTE-------------LNYAL-CQEF--IKK--YCKY

GIV----GKEL---AP-TTNTPHLQGFCNLQKPMRFSTIKKRLD-------NGIHIEKSM

-GSDTQNQTYC---------SK------S-----G-EFFEA-GDP-QC-------QGK--

--RNDL-QSVVDT-IQAGN-GSLSSIAN---E-HP-TAYIRYFRGIQE-YIKTV------

-----------R-PIPPRYHKT--------------EVRYYHGPPGSGKSRRALEEATAL

-ASDLNDIY----YKPRG-TWWD----GYKQQ-SCVIIDDFYG--WIKYDEMLKICDRYP

YK----VQIKGGFEE-F---TSKYIWITSNIDTNLLYKFND-----Y--NDTAFVR----

--RIEIKLLIE-------------------------------------------------

----------------------------

>Circo|AIF76266.1

------MANST--VRRFCFTWNNYTE-------------PDYEK-CKTF--IVE--LCKY

GIV----GKEV---AP-NTGTNHLQGFCSLKKPMRFSTIKKRLD-------NRIHIEKAN

-GSDDDNEKYC---------SK------A-----G-DFFVS-GDK-DK-------QGA--

--RNDI-QAVISA-IQNGEVTTPKMVAA---V-YG-SVYIKYHRGIEK-YLSLL------

-----------R-PVPERNFKT--------------ELRIYWGPPGSGKSRRALEEANSL

-G---SSVY----YKPRG-LWWD----GYEQQ-DSVIIDDFYG--WIKYDELLKICDRYP

YK----VQVKGSFQE-F---TSKYIFITSNTDIYSWYKFPG-----Y--ETAAIER----

--RAEIHEYIG---G---------------------------------------------

----------------------------

>Circo|ADD62473.1

MA--RGMANST--VRRFCFTWNNYSE-------------LEYVL-CCEF--IKK--YCCY

GIV----GKEI---AP-NTGTHHLQGFCNLEKPMRFSTIKKRLD-------SRIHIEKAA

-GSDSENQTYC---------SK------T-----G-NFFES-GTP-NQ-------QGR--

--RTDL-QAVVSA-IQNGEITTPKMVAE---L-HG-AVYIKYHRGIEK-ILHQL------

-----------H-PVPPRDIPT--------------ELRVYWGPPGSGKSRRALEEARTL

-G---GSIY----YKPRG-LWWD----GYEQQ-NCVIIDDFYG--WIKYDELLKICDRYP

YK----VQIKGSFQE-F---TSKYIFITSNVDTCDWYKFAG-----Y--NVNAIER----

--RITIKEYMD-------------------------------------------------

----------------------------

>Circo|AEL87792.1

------MANST--VRRFCFTWNNPTE-------------LDFAL-CSEF--IKK--YCKY

GIV----GKEI---AP-TTGTLHLQGFCNLQKPMRFSTIKQRLD-------NRIHLEKAN

-GSDEENQKYC---------SK------S-----G-DFFEE-GSP-GF-------PGR--

--RTDL-ESLVSS-IKNREVTTLKGVAE---M-HG-ACFIKYHRGIQA-YMQQM------

-----------V-PIQPRDFKT--------------EVYFYWGPPGTGKSRRAAEEARTR

TGR--DEVY----YKPRG-LWWD----GYEQQ-KAVVIDDFYG--WIKYDEMLKICDRYP

YK----VQIKGGFQE-F---KAEAIWITSNVSIDLLYRFTG-----Y--DPAALQR----

--RMTCIDYMD-------------------------------------------------

----------------------------

>Circo|AIF76249.1

------MSRTS--VRRFCFTWNDYTL-------------EDCAT-VEKF--ITE--YCKY

GIA----GEEF---AP-TTNQPHIQGFCSLHKPMRFSTIKKRLH-------NTIHIEKAN

-GSDEQNQTYC---------RK------S-----G-AYFEK-GTP-CF-------QGQ--

--RSDL-RAVVSA-IQSGK--NAAQVAK---E-YP-LEYIRYFRGIQE-LDLIT------

-----------N-AKPPRFYKT--------------WVYYYWGPPGSGKSKRALQEAQSL

-D---EEIY----YKPRG-KWWD----GYNGQ-DCVIIDDFYG--WIKYDELLKITDRYP

YK----VQVKGGFRE-F---TSRRIWFTSNVDTHELYKFIG-----Y--NNTAFER----

--RITCKINIV-------------------------------------------------

----------------------------

>Circo|AIF76254.1

----MTSRNPS--LRRFCFTWNNYTE-------------DDIWT-VQTF--ITT--QCKY

GIF----GKEL---AP-DTNTPHLQGFCNLVKPMRFSAIKELLS-------HRIHIEKAN

-GSDIQNQAYC---------SK------A-----G-EVFES-GTP-SS-------RGQ--

--RTDL-NAVLDD-IRGGE-TSLARLAT---A-HP-CTYIRYFRGIEN-YLKLV------

-----------H-PILPRDFPT--------------VVNYFYGPPGSGKSKRALEEAQEK

-SP--CSIY----YKPRG-LWWD----GYRQQ-DCVIIDDFYG--WIKYDEMLKIMDRYP

YK----VQIKGGFEE-F---TTKFIWITSNVDTDKLYKFEG-----Y--NPEAFNR----

--RLTTHVRFVFPEE---------------------------------------------

--SAGP----------------------

>Circo|YP_009021870.1

-------MNSV--VRRFCFTWHDYDC-------------EDVAK-TESF--INT--HCKY

GIF----GKEV---CP-DTRRIHLQGFCSLAKPKRFKWIKEQLS-------NRIHIEKAM

-GSDKENQQYC---------SK------S-----G-EFFEK-GSP-SEG------SGQ--

--RTDI-QSLLET-IQGGE-HDIRRIAE---K-HP-ACYIRYYRGIRS-YLNLV------

-----------A-PVSPRNFKT--------------EVRYYWGPPGSGKSRRSLEESSGL

-LD--GTVY----YKPRG-EWWD----GYMQQ-TSVIIDDFYG--WIKYDELLKICDRYP

HK----VPIKGGFEE-F---TSKYIFITSNVDVCDLYKFNG-----Y--TTAAIDR----

--RITIKENII-------------------------------------------------

----------------------------

>Circo|AFS65290.1

LPGTFNSMNST--LRRFCFTVNNYDA-------------ETELK-VKNF--LTN--NCKY

GIY----GREL---CP-TTKTPHLQGFANLSKPMRFRKIKESLC-------DTAHIEKAN

-GSDEDNKTYC---------SK------S-----G-EFFET-GSP-CQ-------QGR--

--RSDL-DAVVAT-ISTGE-RDIRRIAE---K-HP-NCYIRYGRGIRS-YLELV------

-----------N-PIPPRYFKT--------------RVVFFYGPPGSGKSRRALAEAQAI

-DP--DSIY----YKPRG-EWWD----GYHQQ-TSVIIDDFYG--WIKYDDLLKICDRYP

YK----VPIKGGFQE-F---TSKHIWITSNVDTPLLYKFEN-----Y--NVAALER----

--RLEIKELIE-------------------------------------------------

----------------------------

>Circo|YP_009047065.1

MA-----KNST--VRRFCWTLNNYTE-------------EDVDV-LQKD--LTE--LCKF

AIF----GRET---CP-STGTKHLQGFCNLQRPKRFNSIRQIFG-------GRAHIERAK

-GTDLENKEYC---------SK------G-----G-DVWEC-GTP-CA-------QGT--

--RTDL-EKVVSV-IEGGE-RSLKNLAI---Q-FP-IAYIKYYKGIEQ-YIRIA------

-----------H-GNSERDFKT--------------EVFYFWGPTGTGKSRKAREESLAL

-----GDTY----YKPRG-EWWD----GYTGQ-PCVVIDDFYG--WIKYDELLKICDRYP

YR----VPVKGGYEN-F---VSKYIWITSERCLEEVYRFIG-----Y--DCSSLRR----

--RLNKEIFFGYI-----------------------------------------------

-----------MS---------------

>Circo|ADD62477.1

MA-SRKQTNST--LRRFCWTLNNYTE-------------EDVTT-LQKD--LAE--LCKF

AIF----GRET---CP-NTGTKHLQGFCNLQRPKRFSSIRKLFK-------ERAHIEKAK

-GSDFDNKAYC---------SK------S-----G-EVWMH-GEP-SS-------QGA--

--RNDL-QEVVSV-IEGGE-RNIKAVAL---Q-FP-TTYIKYFKGIEQ-YIRIC------

-----------H-SSAERDFAT--------------QVSFFWGPTGSGKSRRAYEEAKAT

-G---EPIY----YKPRG-EWWD----GYCGH-ANVIIDDFYG--WLKYDELLKICDRYP

YR----VPVKGGYEN-F---VTKRIWITSEKPLEQIYRFIG-----Y--DCSSIRR----

--RLNTELYIGYE-----------------------------------------------

----------------------------

>Circo|YP_008130363.1

------MANST--IRRFCWTLNNYEE-------------DELAS-LQKD--LAE--LCKF

AIF----GREV---CP-TTGTKHLQGFCNLQRPKRFNSVRRIFG-------GRAHIEKAK

-GTDLDNKTYC---------SK------S-----G-EVWMH-GEP-ST-------QGA--

--RTDL-QEVVSC-IEGGE-KNLKRLAI---Q-FP-VAYIKYFKGIEQ-YIRIA------

-----------H-GSGERDFKT--------------ECFFYWGATGLGKSRAAREESKAT

-G---EDTY----YKPRG-EWWD----GYCGQ-PNVVIDDFYG--WIKYDELLKICDRYP

YR----VPVKGGYEN-F---CSKRIWITSNYPLDTIYRFLN-----Y--DKGPLER----

--RIDVIKHFE-------------------------------------------------

----------------------------

>Circo|ADU76993.1

MA-----PNKT--LRRFCWTLNNYTE-------------DDVDQ-LQKD--LPE--LCKF

TIF----GREV---CP-TTGTKHLQGFCNLQRPKRFNNIREIFK-------GRAHIEGAK

-GSDQDNQRYC---------SK------S-----G-EVWSH-GEP-CN-------QGA--

--RSDL-EEVVSI-IKGGE-RDVKAVAL---Q-CP-TAYIKYFKGIEN-YIRIY------

-----------H-ATPERDFKT--------------EVYFFWGPTGAGKSRTAREQALAT

-G---LRVY----YKPRG-DWWD----GYNGH-ECVIFDDFYG--WIKYDEVLKICDRYP

YR----VPVKGGYEN-F---IAKKIWFTSNKPLEQIYKFID-----Y--EPSAWRR----

--RLTVEREFFYE-----------------------------------------------

----------------------------

>Circo|YP_009237526.1

-------MPSS--YRRVVFTLNNYTN-------------EILTS-ICNY--AES--NCRY

AII----AKEV---AP-TTGTPHLQGFLHFKNPKTHKTLGKVL--------PGGHFLHAK

-GSDEDSQVYC---------SK------E-----D-PPWEF-GTM-CH-------QGK--

--RSDL-DDAILT-LESG--GDLKRVAM---E-HA-GAYVRYHRGFAA-YKSLV------

-----------C-ATAPRDFKT--------------KLVVLFGPPGTGKTRAAYELAG--

-----ENPY----PKPRG-EWWD----GYCGN-NGVIIDDFYG--WLKFDELLKISDRYP

YR----VPIKGGYEN-F---CSKIIIITSNIDISKWYKFDG-----Y--DPAALYR----

--RCTKYLRCEKDV-------------------------SME-----D------------

---------VKIN---------------

>Circo|YP_009116910.1

---------MT--SRKWVFTVNNYTD-------------DEWNE-LKRW--CMV--NTKY

AIM----GKEV---GP-QCGTPHIQGYLSANKPHRRSGMVEVC--------RRARWEAAK

-GNDTQNDDYC---------RK------A-----A-KVWRH-GQP-CTG------AGQ--

--RTDL-LRVAAA-VDSG--VSLREIAS---E-NP-SEFIKFHRGILA-YRNLV------

-----------R-PAVPRDFKT--------------ELHVFVGPPGSGKSRRAQELASSF

-----GTVY----RKVRG-PWWN----GYEQQ-QSVIIDDFYG--WVPFDELLRVADRYQ

HQ----VEIKGGFEE-F---NSKIIIITSNRPIKDWYKFDS-----Y--DPEALYR----

--RCSVYQHINMDH-------------------------TDY-----E------------

--IMEGIK---IN---------------

>Circo|AMH87650.1

MP-----EPKS--HRRYVFTLNNYSD-------------DDVAR-LQQL-------EARY

MVF----GKEV---AP-TSGTKHLQGFINFGRAVRFNSARKLVG-------GDGVTEASG

-GPNGDCFDYL---------PH------G-----N-DFVFL-GKP-TS-------QGR--

--RTDL-QRVADL-AQSG--TTAHTIAR---E-FP-VEFIKYGRGITN-LLRTI------

-----------N-PIPSRSKPT--------------IVVVLVGAPGVGKSRFANEVGSGL

-----GDTY----YKPRG-EWWD----GYCQQ-RSVIIDDFYG--WIKYDELLKICDRYP

HK----VPVKGGYEE-F---TSEYVFITSNSNVDQWYKFDN-----Y--SPAAIHR----

--RLSVYVGLMCDGS---------------------HFP---------------------

--WSPKFS-MQIKNKV------------

>Circo|AMH87652.1

MP--GDKPNKS--HRRYVFTLNNYGP-------------EDESR-LANL-------DGRY

LVY----GREV---AP-TTGTKHLQGYINFGRAIRFNTAREMVG-------GSGVTEAFG

-PPNGVCFDYL---------PH------G-----N-DIVSL-GKP-TS-------QGR--

--RTDL-QRVADL-AQAG--TTAHTIAE---Q-FP-VEFIKYGRGITN-LLRVI------

-----------N-PVKSRTKPT--------------NVIVLVGTPGVGKSRFANEVGSAL

-----GDTY----YKPRG-EWWD----GYYQQ-RSVIIDDYYG--WLKYDELLKICDRYP

HK----VPVKGGYEE-F---TSEYIFITTNSLIDLWYKFDK-----Y--TPKAILR----

--RLAVYVTLMEDAD---------------------HYD---------------------

--FSPQQE-CIVNNKI------------

>Circo|AIF76251.1

----MDLQNAR--RSRWCFTVNNYTT-------------EHIEQ-LRKLGSTLL--TSGY

LIF----GYEN---AP-TTNTKHLQGYIRLNKPKLFRQLQLLLP-------PATHIEATK

-GTERQNFRYC---------TK------S-----G-VFEEF-GIQ-RT-------QGQ--

--RTDL-EQAVHE-LHEN--GTILQVAT---N-NP-TVYVRNWRGLHE-LRNLL------

-----------I-PVKPRDFRS--------------NIYILYGPTRTGKSRAAQKIAG--

--D--TSTY----YKNRS-NWWH----GYHQE-ETVIIDDFYG--WIKWDELLKLCDRYP

YK----VETKGGYEE-F---TSKTIIITSNLPPEKWYKFEN-----F--DPTPLSD----

--RINAIVETGGTQ----------------------------------------------

--IKTYYE-KSFNNP-------------

>Circo|KT732825

MP--KAKRESP--AKDYCFTVNNYDD-------------DGVRL-LSGLV-QGG--IAGY

ICF----GYEC---G--ESGTKHLQGFIQFTRKVRFSTVQASLP-------KGTHIEKRR

-GTPEEASEYC---------KK------E-----G-NYHEE-GKL-SK-------SGN--

--RSDL-EAVAKE-VARG--ASDFQIAE---T-FP-AVFVRYYRGLRE-LRCVL------

-----------N-GNVQRSWKT--------------RCHVYYGYPGSGKSLQAE------

---------------PIG-----------------------YG--WIVFDELLKICDRYP

YR----VPIKGGYRN-F---VARDIFITSNSGPDSWYEREP-----I--KREAMSR----

--RFEYVIEFKKMCD---------------------HFV---------------------

--DKFDYE-LAI----------------

>CRESSV3|JX904407

MTK-----QQK--ARSWCFTYYPTNE-------------EDLLW-FKNLTT-TS--QIRY

FVM----GREL---CP-TTGKLHFQGYISYNNAKTFQQTKKWFQ-L-----DKIHIAPAK

-GNDFQNQVYC---------SK------E-----H-LLIEI-GEP-IK-------QGK--

--RSDI-VRAIDI-ITQT--NSVSAVLE---V-NN-YQAVRHCELWLK-YK---------

--------------EPCRPVQS-------------INVIWIHGSSGSGKTRKVYDDNSGN

------EIF----TPTSH-KWWE----GYDGH-QVVLIDDIRR-DFCKFHELLKLLDIYP

FR----VETKGGSRQ-V---QFKTIYITAPYSPIEMWE-GR-----CDEDLLQLTR----

--RITQTIDIDDN-----------------------------------------------

----------------------------

>CRESSV3|KJ641729

---------MY--LRNFVFTLNNYTE-------------DEYNF-IKNFE------QAQY

III----GKET---G--AQGTPHLQGYMELKKQVRFNTVKSVF--------PRMHIEPRR

-GTQKQAVDYC---------KK------E-----K-NFIEV-GKA-KM-------GGI--

--RNDV-ASVIEF-KNS----SFKSFFES--G-AD-IQAFNLLGKIKT-YM---------

--------------EEKRDYKT--------------FVIWCYGPSGSGKSRWAYEISRKY

------NPY----FKDDT-KWWD----GYDNN-SVTILDDFRA-SNMKFNTLLKLLDRYP

YL----IEIKGGYRQ-F---VSSIIIITCIQKHEDTYKILE-----NDEPLNQLTR----

--RINKIMSFPLQN-------------------------TME-----EIISE--SEIR--

--PDEEHE-NTIR---------------

>CRESSV3|JN857329

-------MGQQ--VRCCVFTLNNYTE-------------DDMIQ-LNEW-------DYKY

LIY----GKEI---GK-TNKTPHLQGYIEFNNRKYFSTLKAFN--------PNIHWEKRL

-GSQLQAINYC---------KK------E-----G-NYVEY-GDK-KV-------QGK--

--RNDI-SYYKEL-ALKE---GMKKVVE---E-GN-FQEIRTCELYLK-YK---------

--------------EEERNFKP--------------EVIWIHGESGSGKSLKASEE----

-----KDAY----WKDKS-QWWD----GYDKH-NTIIMDDFRS-SDMKFDYLLRLLDRYP

MR----VQVKGSSRQ-M---LSKKIIITSIYSPEEIYSYNN-----NREPINQLLR----

--RIDKTMKLEQV------------------------------------------RSR--

--SNDGYEIVDLQ---------------

>CRESSV3|KJ641718

--------MDR--SRCICFTLNNWTD-------------EEYQQ-LVNWD------QYEY

LII----GKEI---GK-TNNTPHLQGYIEFKKRISLSKLKKFN--------PKIHWEPRK

-GTQSQAITYC---------KK------E-----N-DFKEY-GIP-KK-------QGE--

--RTDL-VKARTI-VQEH---NMRTLLQL--P-VN-IQQIRMCEKYLT-YC---------

--------------EEQRSWKP--------------KVIWIWGPSGSGKSRLAHAMTEN-

-----EDRY---L-KDES-QWWD----GYDKH-ETIIIDDFRG-KQMNFTYLLKLLDRYA

MK----LQVKGGYRE-C---LAKKIIITSIFSPDRSYAFLN-----EEEPMKQLYR----

--RIDTFINMEEWDE-------------------------------FEKWTE--DRSR--

----------------------------

>CRESSV3|KJ641722

MQ-----------SVNWCFTLNNYTD-------------DDVKA-LKLV-------KCRY

ICL----GFEI---GE-EKKTPHIQGFIQFEKKVRLTAWKKIN--------KKIHAEIMK

-GNVDQAIAYC---------KK------G-----G-VYEEK-GDV-IK-------ERQ--

--RTDL-REAKKK-CAAE---GMRAITGE--E-YN-NQVIRSCEIMLK-YH---------

--------------EKVRDFKP--------------EVVWVYGNSGSGKTKYVKEKCAA-

-----EDTY----WKDST-KWWD----GYDRH-ETVVMDDFRA-SNMKMNELLKLIDRYP

HR----VECKGSFRQ-M---LSKKMYITSIMHPKEVYN--L-----PEEPVQQLLR----

--RIDKIVKVSKL-----------------------------------------------

----------------------------

>CRESSV3|HM228875

MQ-----------SVNWCFTLNNYTN-------------EDVNK-LKQV-------KCRY

ICL----GFEV---GD-KKQTPHIQGFIQFEKKVRLSVWKKIN--------KKIHAEIMK

-GTIEQAINYC---------KK------S-----G-TFEER-GEI-IK-------MGE--

--RRDL-KEAKKK-CAEV---GLRAITDC--T-YN-LQVIRNCQIMLE-YH---------

--------------EKERDFKP--------------EVIWIYGESGAGKTKYISEKCAE-

-----VDTY----WKDAT-KWWN----GYDRH-EITVMDDFRA-SNMKMNELLKLIDRYP

HR----VEIKGGFRQ-M---LSKKIYISSIMHPKDVYN--L-----PEEPVKQLLR----

--RIDTIIKI--------------------------------------------------

----------------------------

>CRESSV3|KM972726

MS-----------IQFVCWTLNNYSE-------------DEYQS-LIEFS------EWRY

IVI----GREV---G--ETGTPHLQGYGELKKRKKFAALKNLF--------PRVHWEQRR

-ASRDAAANYC---------KK------E-----G-RFEER-GSL-PK-------NAS--

--EKSS-KEAITR-VKSG--QTMRAILD---P-PN-LSGIRMCQIWLS-YN---------

--------------EPKRNFKS--------------EVFWYYGASGTGKTKLASEQA--G

-----PDAY----WHDGT-KWFD----GYDAH-EHVLLDDYRG-GNMKFNFLLKFLDRYP

LR----LEVKGGYRQ-L---LAKKIWVTSIKHPKEIYSFSE-----MDEPTEQLLR----

--RITTIKKMCSQ-----------------------------------------ERIY--

----NRFEANET----------------

>CRESSV3|KP153422

MA--DADLNTR--TKAVHFVSFETSN-------------ETVER-LASL-------KTDY

MII----GDEH---CP-TTGRHHLQCYLYTKNKITLSTIHKAL--------KKCKLFIAN

-GTAEQNQTYC---------SK------E-----K-ILYES-GTL-PSG------QGK--

--RTDL-DTTRAN-LLAG--GTLRDVVL---A-TS-YQSVKMAEQILK-YH---------

--------------EKKRDWKP--------------IVKWFWGATGTGKSLTAYDELDK-

-----DDTY---TAMSTG-KWWD----GYDAH-SCVIIDDMRR-DFCKFHELLRYLDRYP

TI----VETKGGTRQ-F---LAKQIIITSCYAPEDMYE--------TREDIQQLLR----

--RIDEVREFTAESL---------------------------------------------

--------INSIN---------------

>CRESSV3|KM874317

-------MSDK--HRSYSFTLNNYTD-------------DEVFA-LTSMCEINR--GVKY

LIF----GKEI---AP-TTGTPHLQGCIIFTSPRSFKALKKVVPF------NRANYKPTI

--SEPGSARYC---------KK------D-----G-DVYEY-GTP-PK-------QGK--

--RSDI-ENVRDI-LTSG--GNIRDVVS---A-TS-IQSVRMAEIHLK-YF---------

--------------EKKRNWKP--------------TVRWYYGATGTGKSKTAYEECDE-

-----HDPY---VAMSTG-KWFE----GYDAH-THVIIDDMRK-DFMKFHELLRMLDRYA

FM----VECKGGSRQ-F---LATDIYITSCYAPDEMFE--------SREDVNQLIR----

--RLDEIRKFE-------------------------------------------------

----------------------------

>CRESSV3|KM874304

--------MSR--SRNWCFTLHNYTD-------------DDIEI-FKNI-------KCRY

IIY----GKEI---CP-TTKRQHLQGYIQFDNQRTITAVKKFLGL------PKIHLEIAN

-GSSDDNKAYC---------SK------D-----G-DMYER-GDC-KQ-------QGR--

--RTDI-TDIKDM-ISNG--ATMRDIIP---A-TS-VQSVRMAEIHLK-YS---------

--------------EQKRTWKP--------------KVQWFYGPTGTGKTRTAYEIL---

-----EDPY---TTLDTG-QWWE----GYDAH-ENVTIDDMRG-DFMKYHVLLKLLDRYA

YI----VECKGGSRQ-F---LARHIIITSAFHPKDVFH--------TREDIAQLMR----

--RIDCVKEFK-------------------------------------------------

----------------------------

>CRESSV3|KP153408

M------TDTR--SRDWCFTLNNYTE-------------EEYDV-IHSL--SLS--QYKY

IVV----GKEV---G--ESGTNHLQGYIYFVNAKSMSAVKKMIS-------KRCHLESAK

-GSPLQAATYC---------KK------D----NN-DYYEN-GEL-PVI------QGK--

--RTDL-DEIRDI-LKQT--NKMSDVVM---A-KS-YQSVKMAEQILK-YH---------

--------------EKPRMEKP--------------YVEWYYGPTGTGKSKKAYEVLSD-

------ECY---TCLSTG-KWFD----GYDAH-KNVLIDDMRK-DFMKFHELLRLLDRYA

MR----VECKGGTRQ-F---VASHIIITSCYHPKDMFE--------TREDIQQLLR----

--RIDKIENFE-------------------------------------------------

----------------------------

>CRESSV3|JX904075

MS--DTDVLTR--SRSWCMTINNPTN-------------ADKAS-LCGL-------EVEY

MIV----GREF---ND-PDKTPHFQCYIRWKNAKTFSRMKKLF--------PRAHLKVAR

-GTDKDNKKYC---------SK------E-----E-VLIEK-GEA-SE-------QGS--

--RNDL-KGIIDM-VQQN--PSMDYIID---V-SS-LQAIRTAEKLLV-YR---------

--------------EPRRSWKT--------------EIYWFHGSTGTGKSRRAYELF---

-----PDAY---TAMDTG-QWWE----GYDGD-EVVIIDDMRR-DFLKFHQLLKLFDRYP

YR----VEVKGGSRQ-F---LAKTIVVTSCYSPEDMFDTR------DQEDIQQLLR----

--RITEIKSFDTI-----------------------------------------------

----------------------------

>CRESSV3|JX904139

MN--DINPKSR--SRAWCLTINNYTE-------------EELEC-IVSY-------SSEY

TIV----GNET---CP-TSGTKHLQCYIRMTNTKTFTKMKKDF--------PRAHIEMAG

-GNDLQNKAYC---------SK------E-----S-VLYES-GTP-SK-------QGK--

--RSDL-EKISNM-ILES--PRMNDMID---L-SN-LQAIRTAEKLLM-YK---------

--------------EPQRNWKP--------------TVMWFCGRSGTGKSKMAAELL---

-----PDAY---WAMDTG-KWWE----GYDGH-EHVIIDDMRA-DFLPYNHLLKLLDRYP

YR----LEVKGGSRQ-F---LAKTIIITSPYSPDQMFG--I-----MHEDNKQLLR----

--RIDKIKYFDNI-----------------------------------------------

----------------------------

>CRESSV3|JX904076

MD--DCNTKLR--KRGWFLTINNPTE-------------DKLKS-L-RYVH-----KSEY

GVI----GEET---CP-TTGTPHYHVYFRLKDAVTFSKIKKDI--------PRANIEPAK

-GNDQQCKDYC---------SK------Q-----N-LLVEH-GTI-SAG------QGK--

--RTDM-SKVKDL-ISED--PRMSSIIE---V-GS-LQSIRTAEKLLI-YK---------

--------------EPKRNWKT--------------EVYWFCGATGTGKSKLAFELH---

-----PDAY---VAMDTG-EWWE----GYDAH-EEVIIDDMRR-DFLKFHQLLKLFDRYP

YR----VQFKGGSRQ-F---LARTIIVTSCYSPEEMFE--------TREDIQQLLR----

--RLTKIKYF--------------------------------------------------

----------------------------

>CRESSV3|JX904581

--------MSR--KRDYCFT--DFVL-------------DE-TF-LQGL-------PYEY

LCY----GRET---CP-TTGKKHLQGYIYFKNAKTFSAVRKLL--------QPRSVRACK

-GSAEQNATYC---------SK------E-----N-DFLEF-GVR-PK-------QGE--

--RNDI-HDIMEN-IQTG--NTMRDIVS---A-TS-FQSIRIAEIQMK-YF---------

--------------EPIRQWKT--------------HVSWFYGKSGTGKSKLAYEMCE--

------DPY---MCMETN-KWWE----GYDAH-EDVIIDDYRR-DFCKFKVLLHLLDQYP

MR----VEAKGGSRQ-F---RAKRIIITTPKSPQETWE-GR-----TDEDLYQLTR----

--RIDIIKEFV-------------------------------------------------

----------------------------

>CRESSV3|KM598406

MA-----HISR--SRAWCFTLNNFTP-------------EELAE-IEN--------KSDY

GII----GDEI---G--EKGTRHLQGYLYWKNPRKFTFIKKII--------PRAHIEPAK

-GNPQQNITYC---------SK------D-----K-IIATW-GEP-PK-------QGK--

--RTDL-ETMREI-LKEP--EPMRKITE---S-LN-YQATRSAEKWLC-YN---------

--------------ERKRDWKP--------------EVTWYTGPSGTGKTRAAKEEL---

-----PEAY---WKSGTN-KWWD----GYDGH-KEVIIDDFRG-SSIAFTDLLRLLDRYP

MQ----VECKGGSRQ-F---LAEKISITSIYHPEKVYKNL------EEEPIEQLLR----

--RIDKIVNLTDTDV---------------------------------------------

----------------------------

>CRESSV3|KT149409

-------MNVR--SRDWCFTVNNYTY-------------DDYKV-CQELSV-----MCQY

VVI----GKEV---A--ETGTKHLQCYVYFENAKSFSKMKKLL--------PQAHIEKAG

-GTPQDASNYC---------KKGEQPKNELK--NA-DFWEH-GSL-PQP------SGT--

--RNDL-KEIREM-LAQG--KGMRDVIE---A-TS-YQSMKSAELILK-YK---------

--------------ERKRNWKP--------------KVIWLWGRSGIGKTRAVYEEC---

-----PNIYR--KSNNSG-KWWD----GYDAH-ENVLIDDVKD-TSEEYSMLLEILDRYD

VR----VQTKGATRE-F---LAKTIYLTSLEDPRIMYR--R-----FPEEGYELYR----

--RIDEIRHVT-------------------------------------------------

----------------------------

>CRESSV3|JX185418

-------MGSP--AIGWCFTLNNYTE-------------IDEQV-LQAL-------DVKY

LVY----GHEV---G--EGGTPHLQGYLELHKKKRFKQVKEMLG-------ERYHIEMRR

-GTAAEVDAYC---------RK------Q-----N-NVFTK-GTM-GG-------QGL--

--RSDL-DRVRLL-AAES---GMRAVAA---T-AN-YQQIKTAEIYLT-YN---------

--------------EEPRSWKP--------------TVYWIYGASGTGKTRKAYEICGTE

------DTY---VKVGTH-KWWD----GYDGH-ENVIIDDFRP-MGIPFVDLLGILDRYE

FR----VEVKGKMRQ-M---LAKTIVITTIYPPEVLYV-NL-----KDEPLQQLLR----

--RIDHFINFNLNS-------------------------DNI-----EHEFE--------

----D----EDIN---------------

>CRESSV3|KM874300

MTHKMAD--VR--KRAYCMTIHNWSE-------------ETVNQ-LKNL-------KYQY

LII----GNET---CP-TTDRKHLQVYAYFKNALSFKSIKKQF--------PTAHIEASK

-GTPNHNKTYC---------SK------E-----D-VLFEE-GTF-PE-------QGK--

--RSDI-EVCREI-VQSG--AGMREVVN---C-QS-VQGMRIAESYLK-YH---------

--------------EKPRKWKP--------------TVQWFYGESRSGKTREAYEILGD-

------DCY---TCLNTG-KWFE----GYDAH-ENVLIDDIRK-SFMPYDEFIKLIDRYA

FR----RRMLRKFPP-V---FSKKDNYHLLLSPRRCLE-GR-----Y-------------

------------------------------------------------------------

----------------------------

>CRESSV3|KM598404

---------MS--FQYHNFVFTSFKT-------------EDFER-VLGL-------DVQY

IIV----GKET---CP-ETKREHLQGYCELDKKKTMAGIKKLFS-D-----DSLHIEARK

-GTQKEAIIYC---------QK------E-----G-NWKEA-GVQ-RK-------QGS--

--RGDL-DGARVM-AVES---GMRAVS----S-MN-MQQIRVAEKFLS-YN---------

--------------EEVRDWKP--------------EVLWFWGDSGSGKSREARFISHGL

------DTY---VKNTGG-KWWD----GYDGH-EVVIIDDFRD-SWWALTYFLACTDRYE

FQ----VEVKGGFRQ-L---RAKMIIITSLFHPKYMYRAQA-----KGDTAYQILR----

--RIDSVVHFGSQK-----------------------------------------EVG--

---------ETIG---------------

>CRESSV3|KF738883

-------MGHQ--VKRWCFTLNNYTE-------------EEYKE-IEKI-------EWTY

III----GREV---G--EEGTPHLQGYIELKKKSSLNQIKKVN--------QKIHWEQLR

-GKPFQAADYC---------KK------E-----N-NFQEW-GHI-SMS-----GGGT--

--TEKI-GAIIEK-AKSG---GMREVLK---P-PK-LATVRLIEKYLS-YC---------

--------------EEERKEKP--------------YVHWNLGNITT-------------

--------Y---TGRMTP-NGGD----GYDKN-ETIVIDDFRA-SIMRFTYLLRILDRYP

MR----VEVKGGYRQ-L---NSKNIVITSIIHPESCYH--M-----ENEPIRQLMR----

--RIDKITETNNKN-------------------------NDV-----MLQATS-EESV--

---------EELG---------------

>CRESSV3|KT149403

-------MTTR--IRAWCFTLNNPTD-------------RDKRA-LIALAN-----TTRY

VIA----QLEH---AP-TTGTPHYQGYMVFHDGKSFSALHKAM--------PRAHFEPAR

-GTAQQNYDYC---------TK------A-----A-DVLEH-GTL-PQ-------QGE--

--RVDI-SQVREL-LRSG--VTYRELVD---D-VN-YQVLRIAQEWLR-VH---------

--------------EPARDFKP--------------EVRWFYGEPGAGKTRAALEWLESH

-----GEVF---TVETPA-KYWD----GYDGH-NCVLFDDLRA-DQCGFVRMLRLLDRYA

MR----VEVKGGSKQ-L---RARYIAITAPHRPEEIYR--T-----SSENIVQLTR----

--RISSVTHVTRDDP--------------------RYGN---------------------

--------SDELY---------------

>CRESSV1|KM874347

--------MSR--KRNWCFTLDGYT--------------DNIADMVSD---EEQ--KIRF

IIF----QEEI---CP-ESSRRHLQGYVEYTVSRRLAAVKLSLGS------ESVHLEGRR

-GTASEAAEYC---------EK------E-----D-SFAQW-GELGGE-------QGA--

--RSDL-ATVASA-VISG--KRVEEVAL---E-FP-TTFIRYNRGIEK-LTNVV------

-----------RRKEQMRNLR-------------MLKVVVLWGDAGTGKTRTAFELS---

-----PDHYILS--QDGQ-LWWD----GYEGQ-KHLIIDDFYG--WIKWGLFLRILDIYP

LQ----LPVKGGFTI-A---SWDVVFITSNCMPRNWYE---------RGMPAELAR----

--RLTHIVEFESYET---------------------------------------------

--------FDKIK---------------

>CRESSV1|KT149404

MS-----------SRRWVYTLFAAAE-------------DDYSN-LDDFERLRD--QFRG

LCH----QLEC---CP-TTSKVHLQGYVEFEKPQRMAALKKL-N-------STVHLETAK

-GTREHCVRYC---------TK------D-----E-TRVESLGT--TT-------QGR--

--RNDL-LETTLA-IVNGEL-SRDDVFD---T-RP-DLICKYARGINE-LLTYR------

-------------AKKERQGD-------------ELHTEVLWGDAGVGKTRYAYGRSTP-

-----EDVYILC--KGGA-LWWD----GYDGQ-SILVIDDFYG--WVEHSVLLRILDRYP

FK----IDIKGSSTY-A---NWKEVYITSNRHPSTWYTRA------PWTEDKALQR----

--RLGAIYECKATGS------------------------KVRTVDDIEELDNLL------

----------------------------

>CRESSV1|KM874309

MK--KEKLLMR---RNVVGTLTN--ER----------PH-EITS-L-----------IKY

FIG----QYEI---CP-SSKRRHLQFYCEFVGQQRLGAVRQLFG-------PTVHVEPRR

-GTVDEARTYC---------SK------E-----D-TPFEN-GTP-SK-------SGK--

--RSDL-ADIKEE-LDAG--SSLKSISQ---Q-HF-GQFLRYRKSFEA-YIVL-------

-------------NQDPRTWE--------------MENSILWGEPGTGKTKLAYDLKERD

----GVEGYPLMRNQNGN-VWFD----GYHGQ-EILLIDDYYG--WIPLAFLLQLLDRYP

MN----VQTKGGSVP-F---TSKKIIITSNKSPECWYNWSK-----FGKNFGAFER----

--RINQVFHYVKDKD------------------------KFPE-----------------

--------VSAVE---------------

>CRESSV1|KF133822

--------MSR--LRNVCFTLYNADP-------------EDVASKLSELVDQDS---LRY

AIF----QEEK---CP-ESDRLHLQGYLECTKPVRFGTLKDFLG-------STVHLERRR

-GNRQQARDYC---------RK------E-----E-T--EC-GDA-NK-------QGQ--

--RTDL-DAIREM-VVAG--ATEESIAD---E-YF-GTWARNHRAIAR-YKFL-------

-------------KSKPRDFK--------------PRVIIRWGVAGSGKTRGVYDTHETA

-------VYNVPRPNGGT-VWFD----GYDPH-EVVLLDDFYG--WLPWSMLLQMLDRYP

MS----VPKKGSTCN-F---RAKFIYITSNADPETWYDYSK-----PGIEFEALKR----

--RVDETHHFTTL-----------------------------------------------

----------------------------

>CRESSV1|FJ959078

MDS-SSSTGERFTARTFCVTVNNRDE----------V--EQVQE--SEFVE-----RVRY

VVW----QREL---AP-ETGRVHIQAYVELYMAAGIVMLKRLFNC------PTMHVEKRR

-GTQDQARNYC---------MK------E--TRDG-DPFEY-GTY-SK--TPGNGQGK--

--RNDL-TDAVEA-LEAG---GVEAVVS---K-HP-NTYVRYHRGIHA-LYQAKLEAIA-

-------------SRQQR----------------NVKTAVFYGEPGTGKTHCAFELARIT

----GEEIYILNAGRNQS-VWFN----GYQNQ-KILVVDEMNG-DWIGWQLLLRMTDKYP

LQ----CQTKGGMVW-A---MWEIVIFTSNAHWEDWYPYYA-----GGMDKAALKR----

--RIHKVVKFYGSEA--------------------KYYDTVATTDELEEVEDVV------

--TQQESQ-DEMS---------------

>CRESSV1|KP153497

--------MSR--GKSWCFTIHSRGE-------------GDCDW-LHPIGTFEW--GCQF

MIF----QMER---CP-ETGKLHLQGALQLDKQQRLSFMKKLH--------KTAHWEVMK

-GNWTQSIEYC---------SK------S-----E-TPWEA-GDR-PK-------MGQ--

--RTDL-ETIGKM-VKEN--KTNLELVD---T-LG-AGVSKFQKHISFTYS---------

--------------EKDSDRQAT-----------GVKVIVLYGPTGTGKTYAAVNSLTG-

-----GRAYHLNSQKATK-LWFD----GYENQ-KVLVIDDFDG--SVEFRYLLRMIDVYK

FP----AEVKGGMVW-G---VWDTVIITSNVHPASWYT-----------DTSPLKR----

--RIAEIRLCENQGT------------------------SWE-----ETV----------

--LSNDFE--------------------

>CRESSV2|KM821764

MSTRKAGTNPK--ANRWCYTLNNPE----------------PIP-WN-----ET--TQLY

HIY----GKEI---AP-GTGTPHHQGFICFKNQKYLTQVKELN--------DKAHWEIAK

-GTNQQASDYC---------KK------E-----G-DFIEK-GTL-PE--DP-QRLGKAN

--EVKW-RLINDL-AKAG---NLAEIDI---K-YP-KVWNTSYRNLKS-IKVDH------

-------------MKPLADLDE-------------PCGVWLYGASGAGKTMKARTDY---

-----PNAY----LKLPN-KWWD----GYQGE-ANVIIDDVDP-NHAYMGYLKIWADRYS

HL----AEVKGSTIV-V---RPRKLVITSQYRIKDIWAS-------EPATMSALLR----

--RFKEVYAFKKQNALDALVAEAEEEAQRPTNPSVIVKSRFI-----KNRRKSIQAKY--

--SSGEEE-SDIQSQETMEVLPEDISSD

>CRESSV2|KU043397

MP-----EFKV--GKRFCFTFNNYTE-------------EEEKK-LKE---LEK--NAKY

AVF----GHEE---GE--EGTKHLQGFVVWKTARRPGGLKKIN--------AKIHWENTH

-GTSKQASDYC---------KK------D-----GKDIFEV-GEI-PKDFEH-RKKGAAN

--KVLW-DNIYKL-AKEG---KFDEIPR--------NIYIRHMNNLHK-IH-DM------

-------------TMKDRSMDEFTNKD-------KDHFLWLWGGTGTGKSTNARRIAKKI

-EK-DKEPY----LKGLN-KWWN----GYKCQ-RVTIIEEANP-KACELAHFKQWADKWS

FP----AEIKCSSFDSI---RPEYIIVTSNYSIRECFP--------AEQDYEPLER----

--RFTEIE-LVHSFTEGNVTVSPEPLGN-TSNP--GSASDIPE--HEGSTQEFI------

---------TEIDAEGRRKVI-------

>CRESSV2|KU043406

MF-ATKTSRPH--ARRYCFTLNNYTR-------------DEEEA-IK-----QI--NCEW

LVF----GHEH---SS-GQGTPHLQGAITFNKRTYLATLKKLI--------PRAHFEIMQ

-GNPQQSKDYC---------TK------E----TS-SYFEK-GIM-PK--PQ-HQAGEAN

--EAKW-ESAYQA-AKEG---RFDDIPR--------SLWIKHMRSFKQ-IFED-------

-------------AKQDTDMTEYTDQD-------KHHFLWIWGPTGTGKSHTAHRIAKEL

----SPDPY----IKDLN-KWWN----GFTKQ-RVVIIEEADP-KRCELASFKKWADKWA

FT----AEAKGTVFPSC---RPEYIIVTSNYSIAECFP--------EDADRLPLER----

--RFTEQY-LPDRKL---------------------NVG---------------------

---------------------PE-----

>CRESSV2|KM573776

--------MSR--GKRWCFTINNYTE-------------EDTSR-CE-----RI--DCEY

LVF----GKEI---GE-EEHTPHLQGFIVFKNRKTFNVVKRIIG-------ENAHIEIAR

-GTVKEASEYC---------KK------E-----G-QYFEK-GEL-PP--EQ-NERGQAT

--KRKW-EETLKA-AKEG---RFDDIAP--------DLYIRYRSSLKA-IYQEE------

-------------VNK--NTKEITDFD-------KGHFYWIYGPTGTGKSHLARTMAASV

----DPDPY----LKGLN-KWWS----GYKMQ-KAVIIEEANP-ETCKLAPFKQWCDKWP

FT----AETKGGSFEGI---RPQYIFITSNYSINECFP--------DPNDSEPMKR----

--RCHEFF-KENKDSLG------------------LNDKEMP------------------

----------------ELEVL-------

>CRESSV2|KM573767

MS---KKIMSR--AKRWCYTLNNYTP-------------EEEQN-IQ-----NI--ECAY

HVY----GKEV---GE--NGTPHLQGFICFVNRKVFNAVKKLFKT------ERIHLEVSR

-GTIEEASEYC---------KK------D-----G-NFFEK-GNK-PE--ES-HRKGEAT

--KRKW-EEAREN-AKQG---KFEDIPA--------DMWIKYRRSWIA-EYTDN------

-------------LNK--DITEIKDID-------KDHFYWIYGPTGTGKSHLARQLAQAF

----DPDPY----LKGLN-KWWS----GYENQ-KVVIIEEWNP-EASKLGSLKQWCDKWP

FT----AETKGGHFVGI---RPERIIITSNWNIDECFN--------DSHESEPLHR----

--RIREIY-KKERETLATI--------------------EVI------------------

--------------------I------D

>CRESSV2|JX904107

MRT-HGTAKMK--SRHFAWTMNNPGP-------------DTEAA-VI-----AI--DCKF

ATF----GREV---GE--SGTPHLQGMTSFEHARSLSAIIKLLQ-KA---HPGTHVEICR

--DAYKSMVYC---------QK------D-----G-DTWQT-GTM-PK--KP-ADGGGME

--RDRW-KRAYEM-AAEG---NLEDIDA--------DIKLRFYGTLKK-IKEDH------

-------------QVTPTSLPT-------------LDFHWYQGSSGSGKSKFAHDEN---

-----PGYY----LKSPN-KWWD----QYEPG-QTVIIDEWDP-NHKVLASLKKWADHHP

FA----AEIKGGTRM-L---RPPKLIITSNYTIKECFP--------QENDHLPLLR----

--RFTVKQ-FGEPDA---------------------------------------------

----------------TFSL--------

>CRESSV2|JX904562

MA-SSSKRLKK--ARSFSFTWNNYTE-------------ADIAR-LK-----GI--EHQY

LVF----GKEV---GE--SGTPHLQGMINFKSPRSFASVMKEL--------EGAHVEKTI

--SSYDSMVYC---------KK------D-----G-DVYEH-GKP-PV--SQ-KAKGDME

--KARW-KRTREL-AAAG---KIEEVDD--------DIYVRFYGTLKR-IKEDH------

-------------QQVPPAQAE-------------LNFHWFYGASGTGKSRAAYAEN---

-----PNLY----IKNSN-KWWD----GYVDQ-PCVLIEEWDP-NLAMMASMKKWADHHP

FS----GEIKGGTKM-L---RPPKIIVCSNYTIQECFP--------NEQDWKPLER----

--RFKVRK-FGEDDA--------------------GSMC---------------------

----------------------------

>CRESSV2|KM821755

--------MAR--SRNYVFTVNNYDD-------------EDEHQWME-----VK--DCKY

ICV----GKEI---GE--SGTPHLQGYICFTQLKSLNQLKGFF--------PSAHFETKR

-GTHQQAADYC---------KK------D-----G-DFFEW-GTL-PV--DDCGSAGAAM

--DELM-GHTIEC-IRNG---DYKGIPN---A----THFIKAYRVLKE-QQQDR------

------------------TLDT-------------LTHQWRWGKAGCGKSKPARDAN---

-----PDAY----LKMCN-KWWD----GYTGQ-EVVIIEDFDP-DHKCLVHLKIWADRYA

FP----AEVKGGKID-I---RPKTIIITSNYHPRDIFD--------REPDLEAIMR----

--RFNVTH-FMNS-----------------------------------------------

----------------------------

>CRESSV2|KT732816

-------MPLR--SRGWCYTVNNWTE-------------NDEEL-VAK---LSE--QTLY

HIV----AREI---GE--EGTIHLQGYCYFKNFKSLAQFKALL--------PRAHLETQK

-GTPQEASDYC---------KK------D-----G-DYVES-GTL-PM--SQ-KRKGDLG

--REYW-EEQLSL-AKKG---KVEECDP--------KLQITHFNALNA-ISARY------

-------------APMPPDNED-------------IDNQWFYGPTGTGKSRKARGDN---

-----PGCY----LKMCN-KWWD----GYLGE-ETVLIEDFDK-KHEVLGHLKIWADRYA

FP----AEVKGSKVN-L---RPKKIIVTSNFHPNEIWS--------DLSTLDPILR----

--RFNVIK-FSNP---------------------FSYLS---------------------

----------------------------

>CRESSV2|JF755415

-------MTFR--NRGWCFTINNPTE-------------DDSLV-AL-----ED--VASY

VVA----GKEV---GE--EGTPHLQGYCYFKNPKSLAQLKVLL--------PRAHLEAQK

-GSHEQAIEYC---------KK------E-----G-DFHEV-GTP-PM--TQ-KRKGEIG

--KEFW-DEQLSL-AKKG---RLEEIDS--------------------------------

------------------------------------KGHWYYGSTGTGKSRKAREDN---

-----PNFY----LKGCN-KWWD----NYQGQ-EVAIIEDFDK-VHSVLGHLKIWADRYS

FP----AEVKGSQIN-I---RPKKIIVTSNYHPNEIWT--------DLATLDPILR----

--RFNIIK-FSQF-----------------------------------------------

----------------------------

>CRESSV2|JX904344

--------MSQ--SRNWVGTLNNWTV-------------DEYVQ-LH-----SI--EYKY

ICI----GKEV---AP-GTGTPHLQMCFFFKNKKSLRALQKIN--------GRAYWEHMR

-GTQEQAADYC---------KK------D-----G-DFEEW-GQFS----TP-KQKGETQ

--KIRF-ERAWEL-AKEG---NLDDIDS--------DIKLRFYGTLKR-IKHDH------

-------------MLQEILTDT------------ESQMEWYCGPSGTGKSRKAREEN---

-----PDAY----LKMCN-KWWD----GYEGE-DIVLIEDFDK-KHDVLVHLKIWADRYP

FL----AECKGSSMK-I---RPAKIIVTSNYHPTEIWS--------DVSDIEPILR----

--RFHVTK-FGNF-----------------------------------------------

----------------------------

>CRESSV2|KP153364

MS-----------ARNFTFTQNNYGD-------------TE----LD-----GV--QCRY

IIY----GKEV---GE--SDLLQLQGVICFNEKIRESAVRKKL--------PGCHIEIAK

--VLPAAIEYC---------KK------D-----G-DWTER-GDS-PM--TP-KEKGALE

--KRKW-DEIRVA-CEEG---RFEDLPD--------DIRYKNLR-LNK-MHRCE------

-------------ALRSRELES------------EAQHLWYWGEAGTGKSRKAREDH---

-----PRAY----LKMCN-KWWC----GYTEE-ETVLIEDFDK-KHDVLGHLKIWGDRYP

FL----AELKGDTMK-I---RPKQIIVTSNYHPSAIWF--------DEETLLPILR----

--RFKCVE-FKKLEA---------------------------------------------

----------------------------

>CRESSV2|JX904185

M------AAKK--FRNYVFTRNNYED-------------TT----FD-----TL--ECKY

IVY----GKEI---AP-TTGTPHLQGTVCFASPRSFEAVRKLM---------GCHIEVC-

-ADLQKSITYC---------KE------D-----G-DWTER-GVC-PM--TQ-KEKGESN

--AQRW-RDIRLA-AEEG---RMDDIPD--------DVRFHHIRTIEH-HR-DL------

-------------ASKKRKIAT------------EEQHLWYYGASGTGKSRKAREEN---

-----PDAY----LKMCN-KWWD----GYDDE-DVVIIEDFDK-KHDVLVHMKIWADRYP

FL----SEYKGGARK-I---RPQKIIVTSNYHPEEIWS--------EESDLGPILR----

--RFKLHK-FSTF-----------------------------------------------

----------------------------

>CRESSV2|JX904420

-------MKKQ--SRNFTFTLNNYTQ-------------EHLTT-LEILV-EQK--AVKG

IFY----GKEV---GE--SGTPHLQGFICFTTTKSMKQCIKAI--------PGAHVEFMK

-GTIEQNVAYC---------SK------D-----N-DFVTL-GTL-PM--TQ-KKKGEAE

--KERW-EEALAA-AKEG---RFDDIPA--------DIQFRYDRNIKR-IYAEN------

-------------KPKPQTLNK-------------LTNEWYCGPSGTGKSKQARDKY---

-----PDAY----VKLNN-KWWD----GYADE-ETVIIDDFDK-YDIALSGLKRWSDHYP

FP----AEFKGGVKV-I---RPKRIIITSNYTPEEIWE--------EEATLGPIRR----

--RFHITQ-FKQLKQ---------------------------------------------

----------------------------

>CRESSV2|KP153377

-------MSKG--NRAWCYTLNNYTE-------------EERDS-LR-----SL--KCAY

QVF----GYER---GA--ADTPHLQGYVQFAHQKTLSAVKKLL--------PRAHLEERR

-GTIDQAVEYC---------KK------D-----G-DFEEY-GKK-PM--SQ-KEKGKEE

--KNRW-KRILEK-ADEG---DEEWLRE---N-EP-NVAFKHMATFRS-HKKP-------

-------------RVGTLQYEE-------------TPHEWWVGPTGTGKSRKAHEEY---

-----PNHY----AKEKN-KWWC----GYTGQ-ETVIIEEADP-KTMELAALKVWADRYP

FP----GEIKGGRIEGI---RPLRVIVISNYTIEECFA--------NQNDVEPLRR----

--RFKEVK-FGER-----------------------PWH---------------------

----------------------------

>CRESSV2|KP153404

MF-KFSMPPSR--SRAFIFTWNNPTA-------------DTEAA-LE-----SL--AYSY

LTF----GRET---AP-TTGTRHLQGYIRFTDGKSLRSARRLL--------NGAHVEVAR

--TIRQAIEYC---------HK------E-----G-DFVEF-GTR-PV--DD-AARGDME

--KARW-EIAWTK-AKTA---DLEEIDA--------DIRVRCYSALTR-IQKDY------

-------------MVPPLPLPA-------------PCGLWIHGLSGVGKTFAVYQAY---

-----PDLY----SKNAS-KWWD----GYQNQ-DHILFDDMDP-DVGKAGRFKIWADERP

FI----ADIKGGSIS-I---RPKIFIVTSQYTIDECFG--------EIQTRMALSR----

--RFRIIE-KLSLDH---------------------------------------------

----------------------------

>CRESSV2|KC248416

--------MSR--NRNYVFTLNNYTP-------------VHEIT-LN-----SI--PHRY

LVY----GREV---AP-TTNTPHLQGYICFPNAKTISAVRRIL--------AGCHVEVAR

-GSHAQCRTYC---------IK------D-----G-DFYEH-GDL-PA--DP-REIGNAE

--ADRW-EDAWEK-AKAG---AIEEIPA--------DIRIRSYSVLRR-IGRDY------

-------------QPNLALLPA-------------TCGYWIKGESGAGKSHSCFTAY---

-----PDLY----PKGPS-KWWC----GYQNE-EVVLLDDVDP-SHGLIGGLKRWADKYP

FI----GESKGGSFK-I---RPKKFIVTSQYSIEDCFQ--------DVETRVALNR----

--RFRVINKLA--DQ---------------------------------------------

----------------------------

>CRESSV2|KT149398

---------MR--TRRIVFTYNNYPP-------------EYNAW-LD-----SL--GALY

CIA----GREV---AP-GTGTPHLQGYAQYVNPKSITAFRRLF--------MGCHVEPAR

-GTGSQSRTYC---------SK------D-----G-SFCER-GIP-PT--DE-NP-GNRE

--KQRW-EDARSL-AKEG---KFDQIPA--------DIYIRYIGNLHR-IYREI------

-------------LPPLEPLPA-------------TCGRWLLGRTGSGKSKGVRSAF---

-----PLVY----PKPLN-KWWD----GYDDH-THVLLDDVDH-NQSSIGNLKIWSDHYP

FI----AEKKGGSRL-I---RPELIIVTSQYSIRELFN--------DNELVLALER----

--RFQVIN-VNK-DE---------------------------------------------

----------------------------

>CRESSV2|KP153447

M------ANRR--SRGWCFTLNNYTP-------------EHEAL-LA-----AV--PCAY

MIF----GREV---GA--NGTPHLQGFVYFPNAKTFNGAKAVL--------PGCHLEAAM

-GSVAQNVEYC---------SK------D-----G-DVEER-GER-PL--SA-AEKGASE

--AQRW-KDARLA-AVSG---DIADVPD--------DIYIRYYRTLKE-ISKDH------

-------------MARPDGLDG-------------VCGLWLYGAAGTGKSRYAQEQF---

-----PEHY----MKMTN-KWWD----GYQGQ-DVVVMDDMDP-DHACLRHLKRWADRYP

FI----GETKGGAIS-I---RPKKFVVTSQYSIEDMFKNRDGT---DVETVAAIRR----

--RFEVKR-FGNA-----------------------------------------------

----------------PYNI--------

>CRESSV2|KT732819

MG---SQLTQR--SRNFCFTLNNYTE-------------SDTEA-VK-----AI--DCKY

MVV----GFEV---GE--KGTPHHQGYIRFENARSLGATIKQL--------PKPHVEVAK

-GNASQNIAYC---------TK------S-----G-VFFEK-GTR-PI--DS-ETCGRNE

--KRRW-TDARAA-AKEG---RFDDIPD--------DIYMRHVHAIKR-IRMED------

-------------GPKPTDLEPR-----------DTYGLWIYGPPGTGKSHFVRTNF---

-----RDHY----IKGAN-KWWT----GYCGQ-KYVVIDELSP-ASGPLSQMKKWADRWS

FE----AETKGGNSI-I---RPYMIIVTSNYSIEEVF---------SRVDAQAIKR----

--RFTEIK-FQ-------------------------------------------------

----------------------------

>CRESSV2|KT149394

---------MA--TRHVVFTINNWTE-------------EIWLE-VT-----QF--DL-Y

VVV----GKEI---GE--SGTPHLQGYGCFHKKQRYATLARKW---------KGHFEVSR

-GTPQQASDYC---------KK------G-----G-DYFEK-GEL-PE--SQ-SAKGEAT

--KKLY-EDAFSL-AQKG---EILSIPE--------PLRTRFYATYKK-VAKDY------

-------------MPKPESLSE-------------LKNLWICGEAGVGKTVLADLIL---

-----PDAY----SKNCN-KWWD----GYQNE-PGVIINDLGI-EHKVLGHLKLWGEHRG

FI----AETKGGALH-I---RPERIIITSQYEIGQVFE--------DWETRDAIRR----

--RYKQHKALIELAS---------------------------------------------

----------------------------

>CRESSV2|KP153360

---------MS--TRRIVFTKNNWTE-------------EDYRA-LM-----EE--PFSY

LII----GKEI---GE--KGTPHLQGYAEFTKKAKYGALAKKY---------KMHCEVSR

-GTQDEAIKYC---------MK------D-----G-DYAEK-G-------TK-KLDGSAE

--KNRW-EQARVS-AKEG---RLDDVPA--------DIYIRCYRTLKE-IAKDN------

-------------MPKPESLTE-------------LKNLWIYGPAGSGKTRLADAIV---

-----PISY----SKNCN-KWWD----GYQNE-PGVIINDVGK-EHSVLGHFKLWGEHRP

FI----AETKGGAIH-I---RPQRVIITSQYSLTQIWE--------DEETRDALAR----

--RYKVLHDIDRISK---------------------------------------------

----------------------------

>CRESSV2|KP153485

M------YMAR--NRNFVFTWNNYTE-------------DSEQF-LRSRVD-EG--VIKY

VGY----CKEI---AP-STGTPHLQGFCSFENKKSVQQVRVIL--------VGCHVETML

-GSISQNEDYC---------SK------A-----G-ELINY-GVQ-PV--TN-DNKGRAE

--QLRW-QRARES-AKLG---QFDDIDA--------DIFIRCYSTLKR-IRTDY------

-------------TAKPPPED--------------VTCYWIYGPTGTGKSHSVETTF---

-----PLCY----KKNMD-KWFD----GYQGE-ETVYLEDIDK-YQVKWGGLKRLADRWP

LL----VNTKGSMQY-I---RPKRIIVTSNYNLDEIWS--------DSGTLDPLLR----

--RFTVIL-KESQEQ---------------------------------------------

----------------------------

>CRESSV2|KJ547648

--------MSK--NRNFVFTWNNYSD-------------ASKTY-LS-----TL--ACKY

VAY----AEEV---AP-TTGTRHLQGFIAFTNAKTIQQARSKL--------PGCHVETMN

-GSIAQSEDYC---------SK------A-----G-TLTEH-GTK-PI--SN-DNNGRAE

--KLRW-QRARDF-AKEG---KLDEIDA--------DIFIRCYSTLKR-IKSDY------

-------------ATKPQPID--------------PVCIWIYGPTGTGKSHAVETRF---

-----PNCY----KKCMD-KWFD----GYAHE-DAIYLEDIDK-YQVKWGGLKRLADRWP

MQ----ASIKGAMAY-I---RPKFVLVTSNYRIDEIWT--------DPQTLEPLQR----

--RFTEIE-KLTQEQ---------------------------------------------

----------------------------

>CRESSV2|KT149412

--------MSR--HRNYCFTYNNYPN-------------TE--L-VD-----NV--NCKY

IAY----AHEV---AP-TTGTRHLQGYIAFHSTKTLAAARRLL--------PGCHLSAMA

-GSISQNDAYC---------SK------S-----G-TLIER-GEK-PA--SN-DDKGRAE

--KLRW-QRARDL-AKAG---NLDEIDA--------DIYIRCYSTLKS-ISKDH------

-------------MIKPEPVD--------------VKCFWIHGATGTGKSYCVETTY---

-----PDCY----KKSMD-KWFD----GYDNQ-DVVYLEDFDV-YQIKWGGMKRLADRWP

MQ----ASIKGSMKY-I---RPKIVIVTSNYTPNEIWM--------DPQTVEPLLR----

--RFKVIE-KVSQEQ---------------------------------------------

----------------------------

>CRESSV2|KP153369

---VSIEKPLV--SRGWCFTCNNYSE-------------SDYNG-FL-----AA--ESVY

LVI----GKEI---SS--TGTPHLQGYIYFRTEKSLRTLRKLS--------DRSHWEPAK

-GDSDSNFCYC---------SK------G-----E-NFIER-GTR-PA--TG-KRKAANG

--KENY-AETVEL-AKKG---KLDEIDP--------GHLLRFYGSIKS-LQKDN------

-------------LVRPSDSEE-------------LTGLWIYGPSGCGKSKYVRDTY---

----GQDFY----YKLAN-KWWD----GYRQE-ETVLIEDLGT-EHDKLGHLKLWSDRYA

FS----GETKGGMLS-C---RPKRIVVTSQYSIEEIFL--------DEKTQEALNR----

--RFTKLR-LFNLKS---------------------------------------------

----------------------------

>CRESSV2|KP153468

---SVSTMSFQ--SKRWCFTLNNYSA-------------EEQLA-FK-----AS--TVKY

AVI----GIEK---GE--SGTPHLQGFVVFMTAKRLSALKKIN--------PRAHYEQAK

-GTSLQASTYC---------QK------E-----G-VFEEF-GTL-PL--EP-TAVGNAS

--NESY-RKAMDL-AKLG---DFSAIEQ---E-WP-GLWLRHRNAFLS-APRDF------

-------------GIRPKDLDY-------------LPGIWIWGPAGVGKSRLAREVF---

-----PLSY----EKRLN-KWFD----GYSAQ-KAVLIEDIDQ-SHRYISHLKIWTDRYS

FP----AEIKGGAIQ-C---RPEHVLITSQYHIHDIWD--------DVQTREAINR----

--RCFVIY-IDKATSLSDVS---------DTNP----PSVLP-----V------------

-----------------LSVL-------

>CRESSV2|FJ959082

--------MSR--SRNWCFTYNNYDS-------------SP-----------QK--CMKY

LTY----GYEV---GE--SGTPHLQGFVIFKNAVA-KPSQYFK--------PAYHFEKAR

-GTPQQALEYC---------QK------D-----G-NFKEF-GEK-PK--TV-ADGGEAN

--KRRY-EEAFSA-AKEG---RMDDIPA--------DIYIRHYSTLKK-IRFDH------

-------------APPAQNNDV-------------LNNYWVYGPSGTGKSKSVREFF---

----GKSLY----VKNQN-KWFD----GYEGE-DFVLIDDVHP-NWSG-KTLKIWSDHYP

FS----PETKGGHIKMI---RPEGIIVTSNYTIEEMYE--------AEEDRQPIRR----

--RFKVIKEYNKRVA---------------------ASAELP-----PP-----------

---ATEVQ---------VAVIPPPATTD

>CRESSV2|KF738877

------GTTQR--SRGWVFTINNYNE-------------WDFVN-IS-K--LEE--KAQY

YIY----GKER---GE--EGTPHLQGFAYFKQRISFNGIRDIL--------TRAHVEIQR

-GFNAQAIDYC---------KK------E-----G-EFTEW-GEP-SR--------GAGQ

--KDKW-KDVLQL-ARQG---KVQEIEE---R-YP-AIFLRYFQKLCG-FYRPE------

---------------HSIILEN-------------FTNEWWWGPTGTGKFKKLNDDY---

-----PDPY----EKSLD-PWWD----NYQRE-EIVAIEEFEP-RCKINSFLKRWADRYP

FR----CEVKGAFLSKL---RPLKIIVISNYQLDECFP--------NSKDLDPLKR----

--RFKEIH-FP-------------------------------------------------

----------------------------

>CRESSV2|KP153483

------MSNKM--AKNWCMTLNNYDD-------------SEVAR-FESH--MRP--WCVY

YIY----GFER---GE--NDTPHLQCFFSLKAKKRMSCLKKIF--------PRAHFEVKS

ASTMEQASDYC---------KK------E-----E-NFIEW-GVL-PD--NS-TARGKAI

--SDNY-EETVEL-AKKG---DIEAINP--------EHVLKYYPTIKR-IAHDN------

-------------KKMPSDLLW---EE-------HPPNIWIYGPTGTGKSYRARAILQEN

-F---GQFY----SKMAQ-KWWD----KYDGE-EGVLIEDMDI-LHNYMGPMKIWADKYA

FP----VEVKTSGDR-I---RPKVIVVTSNYTIEQIWP--------DRSTHGPISR----

--RFKVIHVLRDAPAVGDLVETHGYQPQ--------YLEELA-----EE-----------

---------M---SES--DLL-------

>CRESSV2|KT732823

---DLAPSRGF--SKYWCFTAFASDF-------------EKERV-FS-----NS--DLSY

IIG----GEEL---CP-LSGRKHIQGFAAFASRKRLTACKKLF--------PSAHFERMR

-GSAREAIEYC---------KK------D-----G-KFVER-GIY-PC--VR-SSTGSAK

--VDRI-KDFIEL-ARAG---NVDLCEQ---R-HP-NQFLRYFSQLKN-LRKFD------

----------------IERLSE-------------A--LWLRGPSGIGKDASRSPLR---

-----VFFF----KKSQL-KWWD----GYNNE-NIILISDFDE-SCK-MVHLKIWADVYP

FN----AEVKGSTIK-I---RPKYIIVTSNFRMDMLF---------LGTALDALKR----

--RFMTFE-FSGSGLLKP-------------------YVDAS-----PSPSSSV------

---PNGGE-ST-----STSCAP------

>CRESSV2|KM598396

MGSGSSGPRRK--YKLYCFTSFAVD----------------EPS-YD-----PT--AMQY

LIY----GRET---CP-DSGRTHLQGFVAFKNRQYFTACKKYF--------GTAHVEACK

-GTFAENQEYC---------KK------D-----G-DYKEF-GTA-PE--IK-SGG----

---DVF-NDVLRK-AEAG---SIQEIKD---L-YP-GLYIRYKKTLES-IKRFN------

----------------AEQLEE-------------SCGIWLTGPPRSGKDYAVSTFF---

-----SSIY----SKMLN-KWFD----GYEGE-ECVHLSDMDK-NHV-MGSLKIWCDRYP

FR----AEIKGGTMV-I---RPKYIVVTSNYKLEDIF---------DGSMLSALQA----

--RFMVMC-YDPVDGLQH-------------------AIDVP-----DMAPKAG------

---SNALQ--------ESQVPKL-----

>CRESSV2|JX185415

--------MSR--CKNWCFTSFNVD----------------PPK-FD-----PI--TTEY

LCY----GRET---CP-TTKKSHLQGFICLKERKRLAGVKKLL--------GSVHLEASR

-GTVDQNIQYC---------SK------D-----G-EFTEF-GSR-PS--SG-PAT----

---SPF-ALAIAA-ATDG---RLDDVKS---T-HP-GIYLRYKKTLES-LQKFR------

----------------SDDLDG-------------SCGVWICGPPRIGKDYAVRK-I---

-----GNVY----CKALN-KWWD----GYLGE-PNVLISDVEP-DHFKIGYLKIWSDRYP

FI----AEIKGSSMK-I---RPEKIFVTSNFRLSDCC---------NGEILGALQS----

--RFTI---YDMFDNLVENG----------------DACNVP-----PAVQEAV------

---TTSKQ--------DFQVVPKAKSS-

>CRESSV1|KT862256

----------Q--AKVWTMTVKNWDL----------F--D-EER-IQNQTEGSK--IIRY

LSI----GKHT---GQ-QTGYQHCHFNLELEKKKTMAWIKRELF-NR----EDIHCEPRR

-GSREQCDSYL---------NK------D-----G-EFKVIINTQ-IK-------PGR--

--RTDL-DDIHDM-IKEG--ASLYDCYE---E-HF-GTVVRCERGLRD-YIALR------

-----------D-TILATKKKY------P-----APEVIVYVGPSGSGKSWHCSEDPDYE

-EG--G--YRFSIQMDSK-IYFD----GYNNQ-KTLWFDEFSG-KTMPFTKFCQIADRYP

GR----YETKGGSVLIY---GLKKILISTVEYPALWWGSDR-----YNKDPEQLFR----

--RITKCYYLGQPRI--------------------EYAIEFNPR---HLRTQYDGNV---

---K----EEPIA---EEAVKEDEE---

>CRESSV1|KF246569

----------Q--VKMWALTVKNWDL----------F--D-LEE-IGNYQDGQY--LVRY

LSI----GKHT---GE-KTGYQHCHLNLELEKRQYMTWIKRVLF-NI----PDMHCEKRQ

-GTREQCDTYL---------AK------D-----G-EFQVIVNRR-IE-------RGH--

--RSDL-DEIHDM-IKEG--KDLFDVYE---S-HF-ASTVRYSSGLEK-YIVLH------

-----------D-SKRARNSET------T-----APQVIVYVGPAGSGKSWHCFNDEDYK

-ES--G--YRFPIQMYEK-VYFD----GYNRE-KTIWFDEFNG-RSMPFGKFCQLADRFP

GI----YETKGGSVLIS---GLKKILISTISYPATWWGSNR-----FNLDPDQLYR----

--RLTKCYYLGPPRR--------------------EYAIEFNPK---ELLTKEQRHV---

---K----DDPIL---RDRLK-------

>CRESSV1|KJ206566

-MV-------K--YRKVFVTSFNTSA----------M--E-QDL-VDC-----D--VARY

VCM----SMEH---AP-TTGRIHYHLYIEFYSQKTMNTIKKILK-DA-----TANIQPAR

-GTPQEAVDYI---------KK------D-----GATWKEA-GTM-SN-------QGH--

--RSDL-DDVFQR-LQVG--DNILDIIE---G-HP-GTCIRYIKGILA-VKGLV------

-----------D-QRRQRQEAR------Q-----MPTVLVYIGKSGAGKSHACSQDPDYQ

-RS--G--YKYPVQGPSK-VYFD----GYTGE-STIWFDEFGG-SVLPFHVFLRLADKYE

TR----VETKGGSVCIT---GLQKILISTTTPPKLWWESRK-----FNEDPYQLWR----

--RLTRVYYIPRPSW---------------------FADLIQHP---ENFDELVREA---

-----ETRVDPVH---DD---DDSE---

>CRESSV1|KU043411

TMN-------R--MRCATVTSFNIDY----------I--K-RDV-INN-----S--RLTY

IVA----GLEV---CP-ETGKLHYQMYMEFEEKVTVKQIKRLLR-DN-----GAHVEPRY

-GTAKEAIEYC---------KK------D-----G-NFMEF-GKP-KQ-------QGE--

--RTDL-ADVYES-LKVG--RSLLDIIE---E-HP-GTYIRYFRGIER-VQDLF------

-----------R-RKQQKLEER------V-----QPTVLVYIGKSGTGKSHHCYHDPDYQ

-AS--G--YKFPVQQAGK-VYFD----GYDGE-STIWFDEFGG-SVLPFGVFLRLCDKWE

TR----VETKGASVCIT---NLRKILISTTTYPKNWWDSRK-----YQEDPKQLWR----

--RLTHVYYIPYLML---------------------YAEEIADP---ENFGDDVRRA---

---QEEERIDLVG---ES---DDDA---

>CRESSV1|KU043424

MPA-STGRPKR--FRFACITAWNMDA----------F--D-PQR-MHD-----K--GLSY

VVI----GRET---CP-RTGRHHLQCYFEAPNPKTVEQWQDVVC-DK----PRAHVECRH

-NEGDRAADYC---------KK------D-----G-DFIEY-GTA-PG-------QGA--

--RMDL-ADCKTM-IDEG--KQMIDLYE---K-HF-GTCVRCHRGLML-YKDLV------

-----------D-RKRRKEAEP------E-----PKEVVVYVGASGSGKSHHCWHDPDYR

-RN--G--YKYPLLAENK-VWFD----GYEGE-EVLWIDEFRG-SVFPFGLFLQVTDKWG

AR----VEVKGGSV-ET---FFKKILISTTVPPGEWYKCPN-----FLSNPQQLWR----

--RLTKVYWLGPVEH--------------------IYPELIPDP---EH-----------

-----DTRYDGV----------------

>CRESSV1|KM573766

MPA-------R--YSNWILTTWD------------------PYL-KENWWEMDN--LVQY

VTV----GHEH---CH-EEQREHWHIYFELNHRKNMASIKEWLN-DL-----TVHCEVRR

-GTGQQAIKYC---------QK------G-----F-DFWEL-GEP-KR-------QGS--

--RSDL-VNCKQL-LEEG--VSMIELAQ---A-HF-GDFVRYYRGLYQ-YADLL------

-----------S-REKQRTAGL------S-----EVEVTVFIGPAGSGKTYNCSKKAEYE

-EN--GMCYQFMQQQNGK-CYFD----GYEKQ-KCIWFDEFTG-STMQFNHWCRLADKYG

VR----VETKGGSVQIS---GLKRIIISTIIPPGEWWNSQS-----FRDDPEQLWR----

--RITEIYYCPK-ST--------------------TYYKLIPEED-WERICRPYTLK---

---V----IDD-----------------

>CRESSV1|KX388513.1

---LEAKRHKQ--LAEWT-TYG------------------------------DP--TTMN

FII----GLEY---TQ-NQDNFHWQGYVQFHKSLRFTQVKKYLK---S---YTAHVEQAN

-GTLEENIQYC---------SK------S-----A-SAKSY-GKP-VE-------SGE--

--RTDI-ERLYVD-VKSG--KSMACIID---H-HT-NTWFKYHGAVDK-YLEQK------

-----------V-ACQKRTCTV--------------TVWVDQG--QTGKTTRALYDINED

-IA--KTVFR--THEEGR-LWWD----GYDGQ-PTLVIDDCVH--LIKFDYWKSLVDGHP

MR----IQIKNGWKN-A---LWNRVIINSNRHPNNWWPDNS--E--ANLSMPYFKG----

--RIALINHVQKTDV-------------KG-NELYTFNPHVGPQAQREALRE--------

--------FNDYH---------NR----

>CRESSV1|KX388515.1

LK-FMSKRPQQ--LRHWTFTIGGEEA-------------QTITG-IED---WDA--TTMN

FII----GLEY---TQ-NEDNFHWQGYVQFHKSLRFTQVKKYLK-------STAHVEQAK

-GTLEENIQYC---------SK------S-----A-SAKSY-GKP-VE-------SGE--

--RTDI-ERLYVD-VKSG--KPMASIID---H-HT-NTWFKYHGAVDK-YLEQK------

-----------T-ACQKRNCK--------------VTVWVDQGQ--TGKTTRALYDIDED

-KA--KSVFR--THEEGR-LWWD----GYDGQ-PTLVIDDCVH--LIKFDYWKSLVDGHP

MR----IQIKNGWKN-A---LWNRVIINSNRHPNNWWPDNT--E--ANLNMPYFKG----

--RIALINHVQKTDV-------------KG-NELYTFNPHVGPQAQREALRE--------

--------FNDYH--------EDRNIVK

>pCRESS1|CUO57637.1

MA-----NDTV--SRSWFAVLPYPEK----KG----SP-EEILEQMKQWI-GDN--PLKK

GHW----AYCI---S--KEGMPHVHMVLEGSVSMRFSAVRKCY--------GKAHLEPTR

-GSRKMVLQYI---------HK------R-----GERFVSY-GNI-EGN------KNQ--

--NAIL-DRIEEL-IEDG--KTPNEIMG---E-DI--RLRKEETLVRK----AF------

-----------F-AKRYRETPPYR----------KVTVVWHLGESGSGKTFSYTKLCESR

----EEEIYFCAEYAA-----FD----LYSGE-KILFLDEIRS-TSLPYETLLTLIGPYR

TQ----IHCRYANAF-A---LWEEVHICSILAPEDLYKGMV--KSEERDCIQQLLR----

--RINKYVYHYKENK---------------------YKTEVEAK-RYTSYEEL-------

---KYSVKFSYIN--------PREEKDE

>pCRESS1|CUO23215.1

M------ADTV--SRSWFAVFPNPEQH---EG----SP-EDIIEKLKQWI-GSN--PLRK

GWW----GYCI---S--EKGLPHVHMVLEDTGSCRFTKVKKAY--------PTAHLEPTK

-GNKKQVLAYI---------KK------E-----PEQFTSY-GNI-EGN------KNT--

--NDTL-ATIEML-IEEG--MTPNQIMA---E-DI--RLRREENLIRK----CY------

-----------F-AKRYKETPPIR----------TVKVVWHCGDSGCGKSYSYIDLCEKY

----DDLVYFFSDYAG-----FD----GYCGE-PYLFMDELKQ-DSLPFELLLTITQGYR

SQ----IHCRYSNCI-T---LWNEVHITSIFSPEDIYAGVV--SREGKDTIKQLLR----

--RITKYVYHYKVDE---------------------YKAELAGD-QYIDFDDL-------

---KRRATFMQID--------PD-----

>pCRESS1|CDF01935.1

ME-----------SRSWFCVFNNPAEH---TG----EP-QEVCERLKEWV-NGS--DTRT

GAW----AFCK---S--KSGLLHVHMVLEDTKSMRFTAIKSSY---C----QGMHFEPTK

-GNKKQADDYI---------NK------R-----GEEYH---GEI-KGK------QGK--

--RTDL-DCISDL-ITDG--LKPSEILE---E-NP--RYYTKENIIKK----MY------

-----------F-RKRYAETEFTR----------DVKVFWHYGSSGSGKSYSRKQVVEKY

----EEEIYYLTTFG----GAFD----NYEGQ-KVLWIDDYRG-E-FRFQELLRYLDVYK

AE----LPARYNNVK-A---LWNEVHITSVLTPQLCYSEAC-----NLDRIEQLLR----

--RITCLVYHYKVND---------------------YLTNFSPY-ELCNMQNRV------

--LKKQIAIISIN---------------

>pCRESS1|WP_026669310.1

MVKKSSSYDKQ--SKVFLLTLNNPQK----GY----TH-EFIIDTIHKFKH------VKY

WCI----CDEI---G--KSGNYHSHLYILLGKKKRWSSVKRAF--------PHSYIVKVM

-GSPQECRAYI---------RK------E-----GDTFYEE-GTI-PTF------KND--

--REML--QIEDM-INQG--MRPEQIME---Q-SV--VFRQFETIIRK----SF------

-----------F-AKRLKETPPLR----------QIKIVWHLGASGSGKSFSYTQLCGQY

----EDEVFFASDYSNCT-ALFD----GYEGQ-RVIFLDEVKT-DSFKYGLLLQIFQGYK

GQ----IHSRYNNVY-S---LWTEIHATSIFCPDELYDEMV--PLSSVDSKTQLLR----

--RITDYCYHWKDDE---------------------YHVQIPAA-EYKSYKDL-------

---KERAEFKNTD--------PVD----

>pCRESS1|WP_026524352.1

MVKKSSSYDKQ--SKVFFLTLNNPQK----GY----TH-DFIIDTIHTFRH------FTY

WCM----CDEI---G--ESGNYHTHIYILLGKKKRWSSVQRAF--------LHSHIEKVM

-GSPQECRAYI---------RK------E-----GETFYEE-GTI-PTF------KND--

--REML--QIEDM-INQG--MRPEQIMQ---Q-SV--VFRQFETIIRK----SF------

-----------F-AKRLKETPPLR----------QIKIVWHLGASGSGKSFSYTQLCEQY

----EDEVFFASDYSNCT-ALFD----GYEAQ-KVVFLDEVKT-DSFKYGYLLQILQGYK

TQ----IHARYCNIQ-S---LWTEIHATSIYAPDEIYDEMV--AVPTIDSKTQLLR----

--RITDYCYHWKDEE---------------------YHSQIPSS-EYKSYKDL-------

---KERAEFKDAD--------PKD----

>pCRESS1|WP_053982727.1

VT-K-FSKSIK--GRAWIGTIANMEKAG--E-----NP-EQLAEFISEWS-ASG--KRRV

AGV----AVCV---S--AKGLYHAHVVLYGNLTT-LGNVAKIL--------FDSHIEPQL

-GGKKELKSYL---------LK------E-----PEQL----DNI-QD------TKGK--

--RSDL-EDIEEL-LEQG--FTPREIM----E-NF--PYRKYEKMIKS----AF------

-----------I-DKRIQETPLLK----------EKKCIWIVGESGTGKSYYYYQLCQEH

----VENIYFATDFEF-----YI----EQGAP-PILFMDEFKG-D-MRFAQLLVMLDKFR

AQ----VHCRYSNCF-C---LWSTVVITSVFPPDEVYAGMV--DDADRDKIDQLIR----

--RLDVIEYRYKQGE---------------------YRTSIPAK-EYVDYDDL-------

---KKRA----LE---------------

>pCRESS1|WP_003102166.1

MT-KEFDSQFK--ASSFCCTLNNIDKLFSFTY----SD-EEMVEHLIYWVDGKE--ETRS

AAA----NYEI---G--DSGNHHSHLILEAKNQARFSAIKKLY--------PGIHVELTR

-GTREEVIAYL---------NK------S-----GHTH----GVI-EA--NQ---QGK--

--RSDL-DTIQEL-LEAG--LTPESIMR---Q-NL--SYRKFSKMIKE----HY------

-----------Y-QMQVENAPLVK----------DIKVYWHLGSSGTGKSILLKQDHGK-

-----DNVYVLSDYGG-----LD----NYTGE-PILFMDEFKG-D-IDYQAFLKLLDVYP

NQ----VHARYSNVY-A---LWDAVHISSIFTPNQLYEMLV--PEEKNDPIKQLFR----

--RIHFIVYHFKTDE---------------------FKTTMTME-EYHKLT---------

---NLKINFEKLC--------QDKNT-N

>pCRESS1|WP_029176105.1

MT-KELDSQFK--ASSFCCTLNHIDKLFSFSY----SE-EDMVNHLIYWVNTQE--DKRS

AAA----NYEI---G--DNGVHHSHLILEAKNQTRFSAIKKLY--------PAIHVELTR

-GSREQVIAYL---------NK------S-----GHTH----GII-EA--NQ---QGK--

--RTDL-ETIQEL-LETG--LSPEQIMR---Q-NL--SYRKFSKMIKE----HY------

-----------Y-QIQVENAPLTK----------DMKVYWHMGGPGSGKSVQLKKTFGT-

-----DSVYVLSDYGG-----LD----NYTGE-PILFMDEFKG-D-IDYQTFLKLLDVYP

NQ----VHARYSNIY-A---LWDTVHISSIFTPYQIYTMLV--PDEKYDPIQQLYR----

--RIHYIVYHSKTDN---------------------YKSTMTMN-DYLTLV---------

---KNKLDFDNLI--------TDDAS-S

>pCRESS1|WP_000032131.1

MS-KELNSQYK--ASSFCCTLNNIDKLFTFNY----SP-EEMVEHLIYWVDGKE--ETRS

AAA----NYEI---G--DNGNHHSHLILEAKNQTRFSAIKKLY--------PTIHVELTR

-GTREQVIAYL---------NK------T-----GHTH----GII-EA--NQ---QGK--

--RKDL-DIIQEL-LEEG--LSPEEIMR---Q-NL--SYRKFSKMIKE----HF------

-----------Y-QLQVANAPLVK----------KMKVYWHLGGSGTGKSVRLKEIFGI-

-----EDVYVLSDYGG-----LD----NYMGE-SILFMDEFKG-D-IDYQAFLKILDVYP

NQ----VHARYSNVY-A---LWDKVHISSIFSPYQIYKMLV--SPDKNDPITQLYR----

--RIHFIVYHVKIDN---------------------YKETFTME-QYLNLM---------

---EQKQCFEDIA--------SGINTVT

>pCRESS1|WP_062004798.1

MTTKEISSTFR--ASSFCCVLNNVDKLFYFTY----TP-EEIVDYLMEWI-GRN--ENSV

CAV----NYEI---G--DKGTHHCHMVLEDKQSFRFSTLQTLF--------PTIHAEITR

-GTKEEVLAYF---------EK------K-----GHTY----GEL-RA--NR---QGQ--

--RSDL-DYIQQQ-LEEG--ATPEEIML---D-HL--EYRAYSKMIRE----HY------

-----------Y-QLRLRDTPDHK----------ELKVYWHTGDSGSGKSVRLKQEFGR-

-----ESVYVWSDYQG-----LD----GYQGE-GILFMEEYKG-E-MNYAEFLKVTDRYP

HQ----MHARYSNVF-A---LWEEIHITSIFSPKQVYNIMV--PEETADSVDQMMR----

--RINKVIYHFKVNE-------------------ILYKQIFSVA-DYNSHS---------

---KEQIEFDRSN--------LDFEK-D

>pCRESS1|WP_003030931.1

MTNSNISSDFR--ANSFCCVLNNVDKLFFFPL----SP-EEIVDFLMEWI-ARN--ENVV

CAI----NYEI---G--DNGVHHCHMVLEDKKAFRFSALQKLY--------PTIHAEITR

-GTKEQIIAYL---------EK------S-----GHTH----GEL-RA--RN---QGH--

--RSDF-DYIQKQ-IENG--ATPEEIMM---G-NL--EYRKYSKMIRE----HF------

-----------F-QHRLAQTPDIK----------DMKVYWHVGESGSGKSVNLKKEFGR-

-----DNVYIWTDFDG-----LD----LYCAE-PILFMDEFKG---MSYKEFLKVTDVYP

VQ----LHARYTNTI-A---LWNEIHITSIFTPKEAYGLMV--PESEIDSYKQLQR----

--RLTNVIYHFKIED-------------------FKYKTTFTPE-DFERHK---------

---QEQIEFDKFN--------NDFTK-D

>pCRESS1|WP_047207334.1

MTKNNISSDYR--ANSFCCVLNNVDKLFFFPL----SP-EEIVDFLMEWI-ARN--ENVV

CAV----NYEI---G--ENGTHHCHMILEDKKAFRFSALQKLY--------PTIHAEITR

-GSKEEVIAYL---------EK------L-----GHTH----GEL-RA--GN---QGF--

--RSDL-HFIQHQ-IENG--ATPEEIMR---G-NL--EFRKYSKMIRE----HF------

-----------F-QHRLSQTPDTK----------ELKVVWHVGDSGSGKSVQLKQIYGR-

-----ENIYVWTDHDG-----LD----LYCAE-PILFLDEFKG---MAYKEFLKVTDVYP

AQ----LHARYTNTL-A---LWNEIHIASIFTPKHAYNLMV--PEGEIDPYEQLQR----

--RLTKVIYHFKTEI-------------------FQYKSIYSPE-DFDNHT---------

---LQQIEFDKWN--------VNFEQ-D

>pCRESS1|WP_029690610.1

MTNYNVSSDFR--ANSFCCVLNNVDKLFFFPL----SH-EEIVDFLMEWI-ARN--ENVV

CAV----NYEI---G--DNGVHHCHMILEDKQAFRFSALQKLY--------PTIHAEITR

-GSKEQVIAYL---------EK------S-----GHTH----GEL-RS--GN---QGF--

--RSDL-DYIQKQ-LDNG--ATPEEVMM---Q-NL--GFRKYSKMIKE----HF------

-----------F-QKKIKETPDVK----------DIKVVWHWGESGSGKSTTLKETCGR-

-----DQVYVWNDHEG-----LD----FYNAE-PVLFMDEFKG---MPYKDFLIVTDVYP

TQ----LHSRYTNTF-A---LWMEIHIASIYTPKHAYNLMV--PEGEIDSYQQLKR----

--RLSEIVYHFKVSE-------------------NVYKTSFTPD-DFDKYS---------

---KEKIEFDKWN--------HDFSK-D

>pCRESS1|WP_029694263.1

MR-----STYR--AHSFCCVLNNVDKLFFFPF----SP-KRMIEILIDWM-EDS--EDRS

CAV----NYEI---G--EDGVHHCHMILEGKQAIRFSALQKLY--------PTIHAELTR

-GTKEEVFAYL---------NK------T-----GHTH----GEI-RA--SK---QGK--

--RTDL-EIIEQL-LEEG--QSPEEIMC---R-NI--GYRRFSKLIKE----HY------

-----------Y-QMRLRMSPRFK----E-----NMDVIYHVGASRSGKSIRLMDKYGV-

-----GNVYVWSDYQG-----LD----NYMGE-KILFMEEFKG-E-LSYQEFLRVTDSYV

QQ----FHARFTNIY-A---LWEKVHISSIFPPKELYKLMV--SNSATDTMEQMML----

--RISKVVYHFKVNQ-------------------VQYKSSFSIE-DFNRHT---------

---IEQIEFDKAN--------NNFET-D

>pCRESS1|CVH76026.1

----------M--ARSWFVTSNNPHIHYRGDD----DY-RTACEAFVMWRRGRNRTDRSG

AVV----AFER---G--EQGTLHLHGLLCSKSDMGKSTLIEKF--------PQTDFRETR

-GSVDDCLDYL---------HK------R-----GETYQWG-DYL-CG--GV---GGG--

--GKTF-ERIDAM-LDQG--MTPNDIFA---L-GT--KYAYYGQEIQR----RY------

-----------N-ALMADKARSRD----------ALRCVYHTGDSGSGKS-KLLEQQGR-

------SVYYTADYDP-----FD----SYNGE-DVLFLDELRS-YSFETPQLLSIMESYR

HE----VPARYSNRL-A---VYSEVHLSSIFPPEKIVP--------PNEPLKQLLR----

--RIDEVVYHATAGR---------------------YVTSVQGA-EYQNVKQLE------

---RLAIELAEVE--------PAFQAVG

>pCRESS2|SCH60086.1

-------MPKQ--SCNWCFTINNPSK----KTDTFDTD-EKVINFIMQY-E-----EVNY

YVF----QRER---GH-NENTEHIQGFIQFKNRKRGTTLQNMF--------PPQHGEFAN

-GTAQQASDYC---------KK------S------DT--EW-GEL-RV--TK---GGK--

--QLTN-EDILQR-IKEG--ADDIRILE---E-FP--QLWNQIDRLQK-VRDLY------

-----------V-FDKWRN--VFR----------DVQVTYICGQSGTGKTRSVMEQYGY-

-----DKVYRITDYKP-----FD----SYHGQ-DVIVFEEFRN--SLPIDNMLNYLDGYP

LE----LPARYMNRI-A---CFTKVYIISNWNFEEQYTAIQ--HK-YYETWNAFVR----

--RIDKIVTYKDGMI---------------------FE-------EI-DLKSYK------

--LDKDEN--------------------

>pCRESS2|WP_036328238.1

MN--------R--SRNWLLTINYKED----PT----NN-DELLDYIKDI-K-----SLTY

TAF----QLEQ---G--EKGTKHHQIYISFEHAKSFETIKKYF--------PKAHIEAMK

-GTPEQASEYC---------TK------P------DT--IY-GEL-PI-------KGK--

--RTDL-EDIYKM-IASG--FSDMQIRE---T-YP-SQYIRYNHKFKE-IRQEI------

-----------L-EEQFNT--LFR----------KIDVVYLVDLPGTGKTRYIMEKYGY-

-----KNVFRVSNYKP-----FD----TYKGE-DVIVFEEFRS--KLPIENMLNYLDGYP

TR----LPARYGDKV-A---CYTKVYIVSNWEYTEQYKNIR--EL-YPTTMQALDR----

--RINFVGNLQEIKA---------------------YDK------EQEEIKNLF------

----------------------------

>pCRESS2|WP_044942941.1

MG-----NNSQ--SRKWALVINNPLE----GL----DH-STIKEILQRF-------SPAY

HCM----ADET---A--STGTYHTHLFFYAPSPVRFSTIKNRF--------PTAHIEKAY

-GSVQDNRAYI---------RK------D-----GETFEEW-GEI-PP--EQ---AEK--

--HPEM-FRLVQN-IRDG--MTTTEIID---D-NP--AMAFRVRDIDL-LRQVL------

-----------T-AEKYAV--ENR----------PLEVSYLYGASGAGKTRSIYETHDP-

-----RSIYRVTNYRSKG-ISFE----GYHGQ-EVLVFEEFSG--QIPIEDMLNYLDIYP

LS----LPARYNDKT-A---CYTKVYITTNLPLEKQYRDEQ--WD-RPETWRAFLR----

--RIHTVVEYLPDGS---------------------TV-------I--------------

---------------------PDQK---

>pCRESS2|WP_021629801.1

MG-----SNSQ--SRKWALVINNPSE----GL----DH-SAIKEILQRF-------SPAY

YCM----ADET---A--STGTYHTHLFFYAPSPVRFATIKNRF--------PVAHIEKAY

-GTVQENRAYI---------RK------E-----GETFEEW-GEA-PP--ER---AEK--

--HPEM-FRLVQN-IRDG--MTTTEIID---D-NP--AMAFRVRDIDL-LRQTL------

-----------T-AEKYAV--ENR----------PLEVSYLYGASGAGKTRSIYEAHDP-

-----RSIYRVTNYRAKG-ISFD----GYHGQ-EVLVFEEFSG--QVPIEDMLNYLDIYP

LS----LPARYNDKT-A---CYTTVYITSNLPLEKQYRGEQ--WD-RPETWRAFLR----

--RIHNIIEFLPDGT---------------------TV-------Q--------------

---------------------PDQKR--

>pCRESS2|CCZ45692.1

MG-----YNAQ--ARKWLMVINNPAE----GL----GH-AAITEIVLKF-------HPTY

FCM----ADEI---A--TTGTFHTHIFFCTRSPVRFSTIKKRF--------PTAHIERAY

-GTPRENKEYI---------SK------T-----GETFAEW-GEL-PA--DS---EDK--

--APEM-FQLMQE-LRSG--KSTMEVLE---E-HP--NLAFRIRDIEL-LRQTI------

-----------L-AEKYSA--ENR----------KLEVTYLYGASGVGKTWGIFEQHDP-

-----WEICRITNYRARG-ISFD----GYNGQ-DVLVFEEFNS--QVPIEDMLNYLDIYP

LH----LPARYNDRV-A---CYTKVYLTSNLPLEKQYRAEQ--WD-RPETWRAFLR----

--RIHNVIEYLPDGS---------------------TV-------Q--------------

---------------------PDTK---

>pCRESS2|CBL15233.1

MG----SSNPQ--SRKWLLTINNPDD----EL----DH-NSVKNTLHLF-------SPDY

FCL----VDEI---A--TTGTKHMHIFIYSKSPIRFSTLKNRF--------PVAHIDKAN

-GSVMENRDYL---------RK------E-----GQTFEEV-GNV-PK--PV---DEN--

--SPDM-SALIEE-IENG--LDTYEIIK---L-HP--KYAFRIKEIDT-LRQTV------

-----------L-SNMFRE--KKR----------QVTVYYIYGKSGTGKTRGIYQKHRA-

-----PDICRITAYRSTG-INFD----SYHGQ-SVLVFEEFVS--QIPIEDMLNYLDIYP

LM----LPARFNDKV-A---CYDTVYITSNISLGEQYSEVQ--HY-KPETWKAFLR----

--RINFLVEYTDVNT---------------------YT-------V-TEINKV-------

---------------------KDD----

>pCRESS2|WP_021882760.1

MR--IISRDIQ--CRKWLLTINNPDE----KF----SE-SEIENILNTF-------KFRY

ACL----SREI---G--ENGTPHIHLFIYAKSRIRFSTIKKRF--------PTAHIDKAY

-GSVVDNIAYI---------TK------T-----GETFKEY-GEA-PS--AL---EEH--

--SPEL-SQILDD-IVSG--MSTSEIIT---E-YP--QHIFRVNAIDT-VRQTF------

-----------L-ADKYRE--RMR----------SVCVTYIHGASGVGKTRGIYKHFPA-

-----ESICRITSYSN-G-VKFD----SYCGQ-DVLVFEEFAS--QIPIEEMLNYLDVYP

LM----LPARYTDKV-A---CYTKVIITSNLPLNKQYVNEQ--IE-KQKTYNAFLR----

--RINYVIEYDKKGN---------------------VK-------K--TLHKEV------

---------------------SDEKDT-

>pCRESS2|WP_013978550.1

M------SDVQ--SRKWFFTFNNPAE----GD----TH-ESVAGRFSEL-------SLAY

WCL----GDEI---GA-ETGTYHTHGFIYSPSPIRFTRLKKLF--------PFAHIEKAN

-GTCKENRDYV---------AK------E-----NPTFEEH-GEL-PK--ER---EPK--

--EDRK-ERLYAM-VEAG--LTTEEIIE---L-DK--SFIFQANTIDG-LIQRR------

-----------L-ASRHKG--VNR----------SVAVIYIWGETGTGKTRSIQERHSA-

-----DGICRITSYRG-A-VSFD----AYKGE-PVLVFEEFNS--QIAIEEMLNYLDVYP

LM----LPARYSDKV-A---CFTQVYITSNIPLEKQYPEVQ--RT-RPATWRAFLR----

--RIGKIAHHLPDGS---------------------IE-------E--LMERGK------

---DAVKP--------------------

>pCRESS2|WP_051600858.1

MELTSKSKDPQ--SRGWMLTINFNGA----PL----TE-DALIELIQM--N-----TFDY

ACF----AFEK---G--EQGTLHVHIYIHSENPRRFSTMKMTF--------PRAHIEKAL

-GSPAEIRDYI---------KK------D-----GETFREL-GKI-PT--PG---ASR--

--SNNK-NKLLED-ITAG--KSTAEIIK---D-SP--DYIFKINCINT-AREEL------

-----------L-NTDHQN--SFR----------DVTVYYVYGATGTGKTYSIYQCYDA-

-----KDICRITDYPRNK-VRFD----AYMGQ-KVLVLEEFRS--EIPISSMLNYLDRYP

LK----LPARYYDRQ-A---CFTTVIITSNIPLEEQYLAIQ--DV-QPETWRALIR----

--RINYVRHYNRNGV---------------------ID-------DY-TISADP------

---SGKIIYHPLLDSP-----TEDILTS

>pCRESS2|WP_009301216.1

M------GNPQ--SRKWNLTINNPKD----GL----TR-EIITDRMNS--L-----FPNY

YCI----SDEV---S--QSGTPHTHIFIYRKSPIRFSTIRSKF--------PTCHCEKAH

-GSVLENKEYV---------SK------T-----GETFFEW-GEI-PN--EK---QEK--

--NPLN-YEVIKD-LEDG--KVIGEIVS---D-RP--ELIFKVKQIEA-LKEAL------

-----------L-I-KNAN--KFR----------SLSVIYCFGESGVGKTRMVYECHEP-

-----IDICRITNYRHKE-MSYD----VYHGE-KVLLLDNFQN--SLCIDDLIALLDIFP

MY----LPARFYDRY-S---VYEFVYLLSVLPLENQYKDIQ--KH-YPLKWNALIN----

--KISKIIEIKETGE---------------------VI-------EH-KKERY-------

---------------------INEKD--

>pCRESS2|WP_038350939.1

M------KDTR--TRKWQITINNPLE----GF----SH-DYIKAQLEKF-K-----SCVY

WCM----SDEV---G--EQETFHTHIYMACSNAVRFSTVKNRF--------EGAHFEMAQ

-GTSQQNRDYV---------FK------E-----GETHEEW-GEL-PI--ER---QGQ--

--RNDM-ADLYDM-IKQG--YSDFEIME---E-SP--AFLMNIDKIEK-ARQII------

-----------T-SEKYKN--TFR----------ELEVTYIYGKTGSGKTRSVMEKYGY-

-----PNVFRITDYQP-----FD----NYHSQ-DVVIFEEFRS--SLKIQDMLNYLDGYP

LE----LPCRYANKY-A---CYTKVYIITNIPFEEQYDNIQ--HV-SPETFNAFKR----

--RIHKILHYKDKNK---------------------IE-------K----EDYF------

---DQ---MSLL----------------

>pCRESS2|KJZ87129.1

MK-----NDSR--SRKWQITINNPVD----GY----TH-ENLKNILNNF-K-----NIVY

WCM----SDEI---G--ENKTYHTHIFLACSGAVRFSTVKKRF--------EGAHFEMAN

-GTSKQNREYV---------FK------E-----GETHEEY-GDC-PI--ER---QGQ--

--RNDL-IDLYDS-IKAG--LSNYDIIE---D-NP--NFMFDVDRIER-ARQMV------

-----------R-DEQYKN--TFR----------ELEVTYIYGKTGCGKTRGVMEQYGY-

-----SNVFRITDYNP-----FD----SYKGQ-DVIIFEEFRS--SLKIQDMLNYLDGYP

LE----LPCRYANKI-A---CFTKVYIITNIALEHQYDSIQ--KE-FNETWNAFLR----

--RIHKIKYFN-GND---------------------VD-------TFNSVNEYF------

---DR---LTLVN--------IKQESI-

>pCRESS2|WP_023977019.1

MK-----NDSS--SRKWQLTINNPVD----GF----TH-EVLKEKLKEF-K-----NLIY

WCM----SDEI---G--ENKTYHTHVFIACSGAVRFSTMQNRF--------KGAHFEMAR

-GTCKQNREYV---------FK------E-----GDTHEEY-GDC-PV--ER---QGQ--

--RNDL-IDLYDM-IKGG--MTNFDIIE---D-NP--SYMLEIDRIEK-VRQTV------

-----------R-DEQFKN--TFR----------ELEVTYIFGSTGSGKTRGVMEYFGY-

-----SNVFRVTDYDP-----FD----SYKGQ-DVVVFEEFRD--SLKISDMLNLLDGYP

LE----LPCRYANKI-A---CYTKVYIITNLDLNDQFKGVQ--VK-HPETWKAFLR----

--RIHKVIHYT-KNS---------------------VD-------EY-KLQEYL------

---DRD--LVPVD--------PEQEKI-

>pCRESS2|WP_018597672.1

------MGDLQ--SRKWQLTINNAVD----GF----TH-EHIIELANTF-K-----SLTY

MCL----SDEV---G--ETQTHHTHVYLAFRSAVRFTSLQKKF--------MGAHFEVAK

-GTSQQNRDYV---------FK------E-----GETHYEQ-GEM-PV--ER---QGK--

--RNDL-EDLYDM-IKQG--MDNYQILE---E-CP--QYMLNVDKIER-CRQIV------

-----------R-EEKYKN--TWR----------DLHVTYIYGETGSGKTRTVMEKYGY-

-----ENVYRCTDYDP-----FD----SYKGQ-DVIAFEEFRS--SLRVRDMLNYLDGYP

VE----LPCRYANKV-A---CFTQIYIITNIPLNEQYTDLQ--RA-QMETWQAFLR----

--RIHEVHVYV-GGQ---------------------VY-------KG-SCEDYI------

---NGF--LPPV---------PDEK---

>pCRESS2|CUP05665.1

MG--ENVKDSQ--SRKWQLTINNPVE----GF----TH-EKLNSILASM-A-----SVIY

YCM----ADEI---G--ENQTYHTHVFLCGRSGIRFSTLKKQF--------EGAHFEMAK

-GTAEQNMQYV---------SK------T-----GETFEEY-GEM-PI--ER---QGK--

--RNDL-DDLYGM-IKDG--LTNYEIME---Q-MP--EALLNLDKIEM-TRQTI------

-----------I-QEKYKN--QWR----------DVQVEYIYGDTGSGKTRSIMEQYGY-

-----SNVFRVTDYLP-----FD----GYKNQ-DVVIFEEFRS--SIRFTEMLTLIEGYP

VE----LPCRYANKY-A---CYTKVYIITNVPLSKQYPAVQ--LD-ESVSWLAFLR----

--RIHKVKKYT-YEG---------------------IQ-------ES-HIEITK------

---DG---FRTVL--------PREVVNG

>pCRESS2|SCH17786.1

ME-----KDSQ--SRKWQITINNPAD----GF----TH-ERIRQELESM-K-----SVIY

WCM----ADEV---G--ENGTYHTHLYLQGKGAVRFSTIKKHF--------EGAHFEMAK

-GTAMQNREYV---------SK------T-----GETFEEW-GEM-PI--ER---QGA--

--RNDI-ADLHAM-IKQG--LSNYDIME---Q-VP--EAMLMLDKIEQ-ARQTI------

-----------V-QESYKT--KWR----------NMSCYYIYGDTGTGKTRSIMEQYGY-

-----EHVFRVTDYSP-----FD----NYRGQ-DVVIFEEFRS--SFRVSDMLNYLDGYP

LE----LPCRYANKY-A---CYTKVYIISNIPLSEQYRNQ------PQETFEAFLR----

--RLNGVLHYT-GHG---------------------IE-------KS-RIELIG------

---SG---FRLIT--------PDKMEL-

>pCRESS2|CDC44519.1

MA-----KDTQ--SRKWQLTINNPVE----GF----TH-DKIKDLIYLM-K-----PVIY

WCM----ADEI---G--EEGTYHTHIFICGRSGIRFSTLKRAF--------ESAHIEMAK

-GTSLQNKEYV---------SK------T-----GETFEEY-GDM-PI--ER---QGK--

--RNDL-DDLYSM-IKEG--MSDYDILE---Q-GS--DYMLNLDMISK-TRQIL------

-----------V-QEKFKN--TFR----------KLDIVYIWGETGTGKTRSVMEGHGY-

-----GNVYRATDYLP-----FD----NYAGQ-DVILFDEFRS--SLALTDMLKYLDGYP

LE----LPCRYANRY-A---CYTKVYLISNIPLSEQYPNVK--RD-EYGSWLAFLR----

--RIQTVKYFS-QGT---------------------VK-------VS-NVSVDS------

---NG---FREIP--------LDSPNDV

>pCRESS2|BAK32345.1

MN-----QDSR--SRKWQITINNPHD----SI----TH-EAILDTIEEM-N-----GILY

YCL----SDEI---G--ESGTYHTHIYLVSNNAIRFSTMKNKF--------PTAHFEIAR

-GTSTDNRDYI---------FK------L-----GETHKEW-GEI-PL--ER---QGA--

--RNDL-ADLYDM-IKQG--MDNFDILE---S-TP--ENMMKLDKIER-TRQVI------

-----------K-ESEYRN--TFR----------KLTVTYISGDTETGKTRYVMESNGY-

-----ENVYRITDYKP-----FD----GYQGQ-PVICFEEFRS--SLPIASMLNYLDGYP

LE----LPCRYSNKI-A---CYTSVYIVSNISLEKQYEYEQ--EN-ETETWNAFLR----

--RIHHEIVHSKNGQ---------------------AE-------K--------------

---EGN----------------------

>pCRESS2|WP_044928503.1

MD--TEVRQIQ--SRKWQLTINNPLE----GY----TH-DAIKKNVCTL-K-----SLRY

FCM----SDEV---G--K--THHTHVYILFQSPVRFSTIKRLF--------PEAHIEKAY

-GSSIQNRDYI---------FK------E-----GETHEEW-GEM-PT--ER---QGE--

--RNDL-TALYEL-ISEG--KSNYDILE---E-QP--EFITQIERMDK-VRQII------

-----------Q-EESYKD--IFR----------NLEVDYLYGDTGSGKTRSIMEKFGY-

-----ANVFRVTNYKP-----FD----QYKGQ-DVIMFEEFQS--SIHINQMLIYLDGYP

VT----LPCRYSDKV-A---CYTKAYILSNIDLKEQYPDIQ--TY-SPETWKAFLR----

--RIHKVQVFK-NGK---------------------VE-------TY-SLFDYL------

---NE---FQMLT--------PKEG---

>pCRESS2|WP_037404274.1

M----GESDTR--SRKWQLTINNPIE----NL----KH-NTIKEYLSNL-D-----NLIY

WCM----CDEI---G--QEKTYHTHVYIHLKNAIRFSTLKAMF--------TTAHIEKAN

-GTAIQNKEYI---------LK------Q-----GSTFEEM-GII-PE--ES---QGK--

--RTDL-IEIFEM-AKDG--IKTVDILS---E-YP--SALLYIDKIEK-VRTEI------

-----------Q-REKFKN--TFR----------KLDVTYIYGKTGAGKTRYVMDSFGY-

-----ENVYRVTDYKM-----FD----SYSLQ-DVIIFEEFRS--SVHVKDMLSYLDGYP

ID----LPCRYSNKV-A---CFTKVFIISNIDLLAQYTDIQ--RK-EVETYRAFLR----

--RINKIIYFD-YSQ---------------------KE-------EFESVNSYL------

---ES-----------------------

>pCRESS2|CDE72464.1

M------KNTQ--SRKWQLTINNPLE----GC----SH-EEIKKAMESF-S-----TCEY

WCM----CDEI---GL-EEHTPHTHVFIYTTNGTMFNTVKNIF--------PIAHIEHCK

-GTYAQNRDYI---------RK------E-----GKTFEEF-GTM-PT--ER---QGG--

--RNDL-ADLLDM-ITAG--LDTSTILE---Q-YP--NYMLQLDKIER-TRQIM------

-----------L-ESKYKN--VFR----------EMEVQYIFGAPGSGKTRGVMEKYGF-

-----DKVYRVTDYKP-----FD----GYHGQ-DVIIFEEFRS--SLKIGDMLNYLDGYP

LS----LPCRFSNKQ-A---CYTKVFIISNIPLTAQYNDLQ--SE-QTATWKAFLR----

--RLNGVVEYLANGN---------------------VN-------NYKTIEEYY------

--------WQGMK--------P------

>pCRESS2|WP_053167095.1

MINIAPKKDSK--SRKYLLTINNPTK----MI----TH-ESIQEILATF-K-----SLLY

ACM----SDEV---A--S--QHHKHVFLAFSSPVRFSQIKKYF--------PSAHIDKSR

-GTAENNRHYV---------FK------E-----GETHWEW-GEI-PN--ER---SGN--

--RTDL-AELYEL-IQDG--FSNAEIIE---Q-NP--DNILFLQHIDR-TRKAI------

-----------L-EEKFKS--NWR----------NLDVTYIFGPTGTGKSRHVMEKYGY-

-----ENVFRITDYLP-----FD----SYRQQ-DVIVFEEFTS--SLKIQDMLNYLDGYP

LE----LPSRYSNKQ-A---TFTKVYILSNSPLRKQYETVQ--IE-KIEVWRAFLR----

--RIKKVLLFDKQGT---------------------HK-------EF-TTDEFL------

---HWTAPANILA---------------

>pCRESS2|CDB27189.1

M----CYTTNK--ARKYQLTFNNPVQ----GF----TH-AVIKTTLASF-P-----GIQY

WCM----CDEI---G--EQGTPHTHLYLYSPNAILFSTLQQRF--------MGAHMEAAK

-GSHRENRDYI---------RK------E-----GETFEES-GPL-PA--EQ---NKR--

--ESIS-SEILEL-VQSG--ASNAEILL---Q-YP--SAMNRLQHIET-ARQTL------

-----------L-EERYRN--QWR----------NLEVTYLWGPTGVGKTRSVMELYSY-

-----ENVYHVTNYDP-----FD----DYRGQ-NVILFDEFRS--SLPVADMLKYLDGYP

LM----LPCRYSNKV-A---CYTKVFLISNIPLSAQYPNVQ--LS-EPETYRAFCR----

--RINQGELEM-QAD---------------------TG-------KE-------------

---------------------P------

>pCRESS2|WP_009246639.1

ME-IVQNNNIQ--RHAFQVTINNPLK----GF----NH-LKIKKTLIEF-A-----TLRY

FCM----ADEI---G--KQGTPHTHIYVCFKSRVRFSTVQKYF--------PTAHIEKPH

-ASVQSNIDYI---------CK------R-----GDTFEEW-GTV-PL--QK----GT--

--RPDM-EELYQM-IDAG--YSNAEILA---I-NN--DYILDIDKLDK-VRTML------

-----------L-IEKYKG--KRR----I-----NLKVIYISGATGTGKTRGVLDEHGD-

-----ENVYRINDYQP-----FD----GYSCQ-PVLAFDEFRS--SLKLSDMLNYCDIYP

ID----LPARYANRF-A---CYETVYIISNWELEQQYKEVQ--ED-NPESWRAFLR----

--RIHEVRIYDRDGK---------------------VT-------NYESVEKYL------

---KRKEEFCTLT--------PEK----

>pCRESS2|CCY69022.1

MQ-I---KNVQ--RNAFQLTINNPVE----GY----TH-EKIKETLIMF-T-----TLKY

FCM----ADEI---G--GQGTYHTHIYVVFSSRVRWSKVKKNF--------DEAHIEIAK

-GSAQSNVEYI---------KK------T-----GETFEEW-GEI-PT--QR----GK--

--KADM-EELYEM-IKNG--YSNAEILA---I-NN--DYILNIDKLDK-VRTML------

-----------L-TEKYKN--ERR----L-----DLKVIYIYGATGTGKTRGVLDEHGN-

-----SNVYRVSDYLP-----FD----GYGTQ-EVIAFDEFRS--GIKISDMLNYCDIYP

IE----LPARYSNKF-A---CYSRVYIISNWSLEMQYSEVQ--KN-SPESWQAFLR----

--RIHEVHHYHADGT---------------------LD-------VYDSVEKYL------

---HRDEEFHVIS---------------

>pCRESS2|WP_052011064.1

------------------MTINNPKE----GF----SH-NEIHEILKNF-K-----TLIY

YAL----SDEC---G--T--CYHTHLFLVFSSRVRVSTIHRNF--------EGAHIEIAR

-GSISDNINYL---------KK------E-----GEKFEEY-GTR-PP--DS---QGK--

--RTDM-SELYQY-INDG--LSNAEILA---L-NQ--DYILNVDKLDK-VRNIL------

-----------L-TNRFKE--EVR----L-----DLQVIYISGATGTGKTRGVFEKDGY-

-----VNTFRVTDYAP-----FD----AYCCQ-ETIVFDEFRN--SLKLSEMLNYLDIYP

VD----LPSRYNNKV-A---CYRKVYIISNWKLEQQYSYEQ--IN-DRESYLAFLR----

--RIHKVVTYKDNGE---------------------KV-------QYNSVEEYL------

---NRGKERE------------------

>pCRESS2|CCX75435.1

MH-I---IDPQ--SYMFQLTINAPVE----GY----TH-EKIVNIIRSF-K-----TNIY

FCL----ADEQ---G--E--RYHTHIFIVFSSRVRFSMVKRYF--------PEAHIEKCR

-GNVSENVSYI---------KK------S-----GEKFEEH-GIQ-PS--DS---KGK--

--RSDL-SELYRM-IQDN--MTNAEILA---V-NQ--DYIMQIDRLDK-VRTTI------

-----------L-MERFKE--TVR----L-----DLEVIYIFGKTGTGKTRRVLEENGY-

-----INVYRVTDYNP-----FD----SYTAQ-QAICFDEFRS--SLKLKEMLLYCDIYP

IE----LPSRYSNKF-A---CYNKVYIVSNWELEKQYSELQ--RE-DKESWQAFLR----

--RIHKVIYYKDINE---------------------II-------EYPSVQAYL------

---ERNSEFRTIG--------PDKG---

>pCRESS2|WP_024346025.1

MA-----KDNQ--SRKYNMTINNPAD----GY----TH-EYIQKTLSTF-K-----SFAY

GAL----ADEV---G--EQGTPHTHVFACFSSAVRFSMIKKHF--------PTAHIESAK

-GSIAQNLDYI---------KK------G-----GDTYKEF-GER-PP--EN---LGK--

--DKDL-ETLYHM-IDEG--LSNAEIIR---I-NQ--DYIMQIDKLDK-IRTTH------

-----------L-QDKFKG--ERR----L-----DLEVTYMFGATGTGKSRGILDEYGD-

-----ENVYRVTDYDP-----FD----HYSCE-PVLVFEEFRS--GLPLSDMLNYLDIYP

IT----LKARYSNKF-A---CYTKIFVVTNWELEKQYAERQ--IT-DRASWRAFLR----

--RIHKVVQYVSKGN---------------------KI-------VYNSVEEYL------

---NRDNEFAPVG--------PGK----

>pCRESS2|WP_038278663.1

MK-----KDKQ--SRKYQLTINNPTD----CF----SH-DKIKEYLVTF-K-----SFEY

GAM----CDEI---G--EQGTPHTHIFICYRSPVRFSMIKKHF--------PTAHIEAVA

-GSIEQNIDYL---------KK------E-----GETFEEW-GDR-PP--EN---LGK--

--DKDL-EALYHM-VDEG--LSNAEIIR---L-NN--DYIMQIDKLDK-IRTTY------

-----------L-QDKFKG--ERR----L-----DLSVEYTFGSTGAGKSRGILDEFGD-

-----ANVYRVTDYDP-----FD----HYSCE-PVLVFEEFRS--SLPLSDMLNYLDIYP

IT----LKARYANKY-A---CFSRIFIVTNWELEKQYAERQ--IT-DKESFQALLR----

--RIHKVKHYVSKDE---------------------VI-------IYDSVDDYL------

---NRNNKFIPIK--------PDK----

>pCRESS2|WP_007865724.1

MT-KNINNNRR--SRKYNCVFNNTDK----NC----SH-QAIKEKLSNW-E-----NIIY

WCM----CDEI---A--K--TPHTHLFVQFKNPVYFSSIKKTF--------PSAHIEEAQ

-GTAEENRAYI---------RK------D-----GSTFEEW-GTM-PQ--TG---QGR--

--RSDL-ANLYQM-IKDG--YSNVEILE---I-NP--DNLLNLQHIDK-ARLEI------

-----------L-SSRYKA--ERR----M-----NLLVTYVSGATGYGKSRYILDNHGD-

-----SNVYRVTDYKP-----FD----TYSGE-DVIVFEEFRS--DLPIGNMLNYLDVYP

LQ----LPARYNNRQ-A---CYNFVYIVSNWKLDDQYHNIR--LE-QKETWNALIR----

--RIHKVRIYTAPGE---------------------WQ-------EY-DTIDYL------

---HG---FQPVD--------PNN----

>pCRESS2|WP_013270924.1

MT-KDTNSNRR--SRKYQLTFNNPEK----NC----SH-QAIKEQLLNW-E-----NIIY

YCM----CDEI---A--K--SAHTHLFIQFKNPVYFSSVKKSF--------PTAHIEEAQ

-GSAEENRAYL---------RK------E-----GTTFEEW-GTM-PQ--TG---QGR--

--RSDL-ANLYQM-IKDG--YSNVEILE---I-NP--DNLLNLQHIDK-ARLEI------

-----------L-SNRYKA--ERR----T-----NLVVTYVSGATGYGKSRNILDNHGD-

-----SNVYRITDYKP-----FD----TYSGE-DVLVFEEFRS--DLPIGNMLNYLDIYP

LQ----LPARYNNRQ-A---CYNFVYIVSNWILEDQYHNIR--LE-QPETYKALMR----

--RIHKVRVYESPGK---------------------WK-------EC-DTESYL------

---HG---FQYTD--------PSRT---

>pCRESS2|WP_066550639.1

MK-----KDTR--SRKYQLTWNNPQD----GE----TH-DKLKEILMKWGD-----SVVY

YCL----SDEI---G--ETGTPHTHMFVCYQNAVRFSSIKDSY--------PSAHIEVAK

-GSPESNRAYI---------RK------D-----GETFEEY-GFI-PK--EG---QGR--

--RNDL-NQLYEQ-IKAG--YTNAELLE---N-DP--DNMLRLSYIDR-TRNEL------

-----------L-IEKYKG--TRR----L-----DLQCIYVFGETECGKTKTILDEHGD-

-----ENVCRVTDYKP-----FD----HYAME-DVLVFDEFRS--DLPIGAMLDYMDIYP

LQ----LPARYNNKT-A---CYHYVYLVSNWKLEDQYHDEQ--LE-HKSSWAAFLR----

--RIKKVREYTGRDA---------------------YV-------EY-SKEEYF------

---NR---FQPVK--------PLNDK--

>pCRESS2|WP_020072285.1

MK-----KDTR--HRKYLLTINNPG-----KW----SH-EKICAVLGKM-------QLKY

WCM----ADEQ---GL-QEQTPHTHVFLVANSAIRFSTVKGYF--------PTAHLDPAC

-GTSEENRAYV---------QK------S-----GDTFEEG-GVL-PT--EN---PGQ--

--RTDW-DIALAM-LEDG--HSAMDVIR---V-QT--HLMRYRSTLEQ-IRQEL------

-----------I-AEQFRD--TFR----------ILETTYIYGTTGLGKTRFVMERYGY-

-----ENICQITGYQG----CFD----KYQSE-DVIVFDEFSS--SLKIQDMNNFLDGYP

LM----LPCRYANRV-A---CYTRAYIISNIPLEYQYANVR--LD-TPAVWNAFIR----

--RIHKVVHFTGENQ---------------------YD-------E--TTKEYF------

---SRQKMWIEIE--------PDTDKCK

>pCRESS2|WP_051639324.1

MI-----KEKR--KRKWLLTINNPQD----GI----TH-DSIKQSLQPY-------TLNY

YAI----VDET---G--AHGTYHYHVYIYFKNAIHFSSLKKLF--------PTANIQQAM

-GNSLQNRSYL---------LKSAHKQPD-----GGTFEEF-GEC-PT--EI---RGK--

--RNDL-ERMYEL-IKEG--YSNSEIIE---A-CG-KTAILHIEKLNK-LRHSY------

-----------L-IDYYKG--TRR----L-----NLKVHYISGKTGLGKSRDILDEYGD-

-----ENVYRVTDYQP-----FD----SYQNE-NVLVFEEFRS--LIRLSDMLNYLDIYP

CV----LPARYSPKI-A---CYDTVFIVSNWEFESQYYELQ--QDPQITTYEAFKR----

--RINGYVKYTEN-----------------------IT-------KYNSLNDYL------

---NRNKGFFTIT--------PKN----

>pCRESS2|EES75484.2

MA---QKNDIQ--RNKYFLTINSPEK----GY----TH-EVIYQVASNF-K-----TFQY

VAV----VDEQ---G--S--NFHTHVLLVFKSRVRWSTVQDKF--------PHAHIEEGK

-GDINQILQYM---------RK------E-----GEQFESW-GDR-PV--DT---KEK--

--VSEF-SELYDL-VYDE--VPTGEIIK---F-NP--KYIRYIDKIAP-MRIEI------

-----------M-NEKYRG--KRR----L-----DLKVIYVFGLSGTGKTRMILDRHGD-

-----ENVFRVTDYFP-----FD----SYNMQ-QVLCLEEFRD--SLTITQCLNLLDIYT

VE----LPARYANKL-G---IYKTVYMVSNWEIGKQFKSVQ--QE-HPETYHAFRR----

--RFHYLLDFR-EKN---------------------VH-------AWNSRDFDM------

---GFTARVQTILESEIGQIIPNTSANG

>pCRESS2|WP_051546484.1

MT-NNKLPLKT--MRRIFLTIENPKT----GY----TP-HKLKSMIKKM-K-----SLIY

GCY----CYET---G--ECGTEHVHIYLSLKNSIRFSTIQNHF--------PSAHIEKSE

-GNHDECIAYI---------QK------D-----GSTFWEY-GKR-PE--VK---RRR--

--KKKM-RSVLDL-IRTG--KSNLEIVQ---I-YP--SFISKMKALDE-IRQEF------

-----------L-KEKYGH--TNR----------HVECTYIYGDTRTGKTSDIIKKYGA-

-----ENVYRITSYAP-----FD----GYRGQ-DVLVFDEFRAENAMPFSNMLTYMEGYP

LE----LPARYGNKV-A---CYTKVIVISNDPLKNQYTSVD--RS--SQSWMAFLA----

--RFMSVQHYT-NNG---------------------IT-------EYGSATEYY------

---EQ----------------TNNK---

>pCRESS2|WP_066546553.1

M------STAR--TRRMQITCNNPEE----GL----SS-DRIKEIMQR--------KTEY

YCF----CFET---G--EQGTDHFHLYVKFVNPQSTRVLSKAF--------GNAHVEIIR

SSSSSQNRDYI---------RK------E-----GETFYES-TEC-PD--ESEEIQGR--

--RTDI-ERMISL-VQDG--ASNVEIVQ---A-VP--SMALKISALDQ-YRQAF------

-----------Y-EEQGK---QYR----------DVTTIYIYGRTRTGKTSSVYANHDP-

-----SEICSVMDYKT---GVWD----QYDTT-RVLLLDEYRS--SLKISELLAICDGQP

HT----LRCRYSNRV-C---LHDTVYIISNISLLQQHKEIQ--RD-EPESWEALLA----

--RIKIVRHYYDVGK---------------------YR-------DY-SVQEYL------

---QVEADFVFCD--------PVTEKKE

>pCRESS2|WP_013271491.1

-------MSTR--SRRMQITCNNPLD----GF----TS-EKIKEIMQR--------KTEY

YCF----CYET---G--ESGTHHFHLYTKFCHAQITDTISKTF--------GNAHIEVIR

NSSSIDNRDYI---------RK------E-----GTTFYES-CEC-PI--DGKENQGH--

--RNDI-DLMISL-VQDG--ASNMEVVQ---A-VS--SMALRIPAIEQ-YRQAY------

-----------W-EEKGR---GYR----------HMDIWYIYGKTRTGKTSYVYQSHHS-

-----SEIYSVVDYKN---GIWD----KYDTT-RVLLLDEYRS--ALPFSLILALCDGQP

LT----LNCRYANRV-C---LHETVYIVSNISLLEQYPNIQ--RE-EPESWNAFLA----

--RINHVRHYYEIGQ---------------------YK-------DY-TVEEYL------

---TTEQNFESCN--------PKNTSPI

>GasCSVlike|YP_007517186.1

-------MAQR--VRRYCLTLNNPTP-------------EECHHLLRE--------VFKR

GFF----ASEV---GK--KGTPHLQGFVHLKNAKTLTGLKKFLG-------SRYHVKQAN

-GTDYENWQYI---------GLLN----E-----G--IVQW-GET-PTE------EGD--

--PDAW-DSILEM-IEGG--FNNRDIVR---K-WP-SIAIRCQSAIDR-YRVEY------

-----------E-WGECR---AWR----------DVEVEYIGGPTGTGKTRGVLYHADGR

----NTDVYRCTNGKP-----FD----KYDGE-GTIVFEEFRS--QYTCRDMLNWIDGHP

LL----LPARYADRM-A---KFTKVIILSNWRFAEQYRTVA--ED-SPETYKAWLR----

--RVGTITE------------------------------------Q--------------

----------------------------

>GasCSVlike|YP_009126903.1

-------MSQR--TRRYCLTLNNPTD-------------AEIASLLQE--------TLKR

GFI----ALEV---GA--EGTPHLQGFVHLKNAKTGTALKKMLG-------SRWHWGKAN

-GTDFENWAYI---------MEDV----E-----G--LIQW-GDK-PAE------EGE--

--PDAW-DSILMM-IEAG--EDDRAIVR---K-WP-SIAIRCQSAIAK-YRASF------

-----------E-WAECR---AWR----------EVEVEYISGPTGCGKTREALYLPDGR

----NTDVYRCTNGKP-----FD----MYDGE-GTIVFEEFRS--QYTCRDMLNWVDGHP

LM----LPARYADRM-A---KFTKVIILSNWRFEEQYRTVQ--AD-SPETYKAWLR----

--RVSTISE------------------------------------R--------------

----------------------------

>pCRESS3|WP_055838650.1

MEARSSSKDPE--ARRWMLTLPESE-----------DQ-AEIEKRLGK----------KA

VVG----QLEA---AP-TTGYLHWQLYLETKSALRFSTLRKLF--------PKGHYKPAR

-DTRIQCVRYC---------TR------E------DK--NL-GQL-LT--LK---QGK--

--RTDL-DSYSEQ-ILEG--KSADEVIH---D-DP--RAVIYASHLWQ-LELIR------

-----------D-RETWGK--KFR----------ELEVHYIHGGTRTGKTSALFETYGY-

-----EAIYRVPNWKP-----FD----GYRGQ-DILLLDEYNT--SVPMVDLLKLLEGYP

LE----VSARYSDKI-A---KFTTVFIVSNLELSEQHQTIQ--HE-HPKQWAALGA----

--RLTSVSELVDDGK------------------------------KPPQLDHYL------

--------VEIVLAQARGSVVPDFEDDD

>pCRESS3|WP_016667133.1

-----------------MLTIKA-------DF----TR-EEVEEKLKKY----------D

YLG----QLEC---GE-ESGYLHWQVLIENKTAIKFETLKNKF--------RTAHCEVAK

--NLFACRKYV---------SK------E------ET--RG-GKFVPE--TK---EG---

---DAV-SDLRHA-IFED--KSADELIL---S-DG--RYRPYVAYAKE-LERIR------

-----------D-ASKFG---SPR----T-----SVNVRYLYGAPGVGKTWGVYDEFGY-

-----PDVYSPGTYIP-----WD----EYQSQ-RVLLLDEFDG--QIEFELLLKVLDIYP

LT----LPCRYQNKY-A---AWDTVIMVSNNPLESLYRRV------SASKWAALLR----

--RINVYEEMVSR--------------------------------ESRLQKTSL------

----------------------------

>pCRESS3|WP_002529618.1

MTT-------R--CRDWMLTLPEEYY----------SR-DIVEDKLRSYD----------

YIG----QLES---GK-ESGYRHFQIYVENKNAIKFETLRSKF--------PRGHYEPRR

-ESKSQCLKYC---------TK------S------DT--AG-GKFVDR--LP---RTK--

--RDIS-AEISEK-MKEN--VPASTLI----Q-DP--RYAQFLKYIEA-LETIR------

-------------LKNLGL--EDR----D-----ALEVHYLYGPSRVGKTYKILHGLNYD

----LTDIYRVSNFKP-----WD----NYEGQ-SVLLLDEFAG--QISFEFLLQVLDKYQ

LE----LNARYRNKW-A---CWTQVWIVSNLPMESLYRRV------SPEQWRALCM----

--RFTSYQRMESDRS---------------------LV----------------------

--------------NVAS----------

>pCRESS3|WP_036342632.1

MR--------A--TRDWMLTIPASKF----------DK-KEVENRLKKYQ----------

YIG----QLEK---GKTDTEYLHWQVFVHGTAAIRFDTLRNKF--------DQVHLEPRC

-GTILDCINYV---------TK------L------DTYERI-RQDGN----A---RTK--

--SRISNEEIYQE-I-SG--KTAGQIIN---D-HP--ELGMQFLKIKA-LENIK------

-----------E-EQFRGLQTEDR----E-----NIEVNYLWGPPGAGKSWHVLNEAGYD

----RRDIYRKNGYQV-----WD----NYQGQ-RVLILEDFTG--QIGIEELLQVTDIYA

TE----LDARYSNHY-A---GWEVVWIISNLRLDDLLKKY------PKELRPALVS----

--RITNVYLMED-RE---------------------MI----------------------

--------------N-------------

>pCRESS3|NP_613078.1

---MEDKRETG--YTDWLLTIRRELPD---ER----TV-DDVVNALQGIFD--------A

AIG----QPEK---G--EGGYRHYQIFAQGK-RQRFSTLKKKL--------TAAHVEPRK

-GSVSEAVGYC---------SK------E------KT--QF-GQI-RH--EKESHQGE--

--RSDL-ARLKAR-AA-G--ETVSQILL---S-EDGELAARYLGWLRA-TCDAA------

-----------Q-AAKYRT--KVR----D-----DLEVNFLYGETGVGKTSHVYESEGI-

-----GTVYTVTDYAA-----FD----KYEGE-GILLLDEFTG--QFPMPLMLKLLDKWP

MQ----LPARYSNRW-A---AFSRIWVVSNLPPNNLYSYA------PESQRRAFFR----

--RFAHFYKMDEAHQ---------------------LI----------------------

--------------EEQPVVSPQP----

>pCRESS3|WP_021975256.1

-------MPST--ARDWMLTISAEKH----------TR-QDVEELLD-ILG--------A

YIF----QQEE---GG-KSDYPHFQAFLQLQTPVHMGTLKNKF--------KKAHIEMRK

-GTVQDCVDYC---------SK------E------ET--RG-GEI-LK--DQ---QGS--

--RSDL-AELRRQ-IMDG--ASVSEVLL---N-DDACQAARYTRYLSE-LATAR------

-----------D-RVKYGR--QLR----------DITVHYLWGDPGVGKTKYIYDNNPI-

-----ENIYRVTDYRP-----WD----EYEGQ-SILVLDEFDS--QFSWDQLLVFLDRYP

VM----LPARYNNHV-A---CFTTVWIISNEPLSKQYPER------TGEKRNALLR----

--RISTNQRMLKGGE---------------------LQA------E--------------

-------------TEH-----TNTEDDL

>pCRESS3|KFI81686.1

----MSERDRQ--SRDWMLTVPAEDH----------TQ-DEVRTLFERIST--------G

AVF----QHET---GA-TTGYEHFQCFLQMKSPMRFSTLKNHL--------TGAHIEPRH

-GSVEDCVAYC---------TK------P------DT--YV-GEI-MK--DR---QGR--

--RSDL-IAFREQ-ILDG--VPVQQVLL---D-DTEAKAAHCTKWLNA-FSEAC------

-----------A-RQEYGN--KLR----------DVSVHYLYGAPGVGKTRYVYDRYPF-

-----EDVYRVTDYAP-----FD----EYDRH-RVLVLDDYDS--QLPWEQLLSYLDRYP

VT----LPARYHNHQ-A---CFDTVWIISNLPLTAQYPDI------TGARRLALLR----

--RITDCTHMLADGT---------------------LV----------------------

---------------------PQREEVN

>pCRESS3|WP_043170238.1

MIDDGKHKDSQ--SRDWMLTIRAEGH----------TE-DDVKALFEKIGV--------G

AVF----QREI---GG-KTEYEHFQCFLQVKTPMRFSTLKNHL--------TDAHIEPRR

-KTVEDCVNYC---------TK------E------ET--YV-GKI-MK--DK---QGQ--

--RSDL-IGFREQ-ILGG--MSVQEVLL---G-DTEAKAAHCTRWLGE-LEAAY------

-----------V-RKEHGG--KLR----------DLDVHYLYGAPGVGKTRYVYDKYPI-

-----EDIYRVTNYKP-----FD----EYNRH-KVLVLDEYDS--QLPWEQLLCYLDRYP

VT----LPARYHNHQ-A---CFTTVWIISNLPLSAQYPDI------VGERRFALIR----

--RLTDCSYMTPEGE---------------------LI----------------------

---------------------PRQEGGS

>pCRESS3|WP_052825216.1

MEEKAKGEDKQ--SRDWMLTIPAGRH----------TE-DDVAELLERICS--------G

AVF----QREK---GE-ETDYEHFQCFLQIPSPMRWSTLKNHL--------AQVHIEVRE

-HSVESCVNYC---------SK------D------DT--YI-GKI-MQ--DQ---QGK--

--RTDL-SDLREK-ILNG--ASVEDVLL---E-DTESKSARYVKWLSE-LAAAR------

-----------D-KKKYGR--QMR----------NVEVHYLWGAPGVGKTSYVYERYPI-

-----EDIYRVTDYQP-----FD----EYDRQ-PVLVLDEYDS--QFDWEKLLCYLDRYP

LM----LPARYHNRQ-A---CYTTVWIISNKSLDEQYPLV------QGERRLALTR----

--RLSDVRHMGEARE---------------------LII------DPKGVPEEV------

---SHEHT-----SDE-----PRSNQER

>pCRESS3|KFI87454.1

MTATVRSNNPS--ATDWMLTVSAEQN----EI----TE-ADLVDAFED-----------S

WMG----QREE---GG-HTGYRHYQLFMQATSRIRLSTVRARL--------EKNYIEPRR

-YSVASCVAYV---------SK------K------ET--IH-GDF-MH--ED---QGK--

--RTDL-EELRDA-VVKG--ASVNEILN-----DPSLKAARFMPWLEK-MVGAR------

-----------Q-AARFSQ--EDR----------EVTVHYLWGKPGLGKTRSVLDG-DR-

-----SQIFRVTNYEP-----FD----DYSGQ-STLVLDEFAG--QLPFQLLLNVLDRYP

CK----LPCRFHDTW-A---GWTTVWIISNKPLERQYQDV------EPQVRAALDR----

--RITTNEEFKSDEE---------------------FAA------REAEVNEDL------

--------LETLSDEE------------

>pCRESS3|WP_022856850.1

---HEKVKDAQ--SRDWMFTLKIE------------ND-STLTTLLGMLGSW----TALE

YVF----QHER---GS-KSGYDHFQGFLRCKSSNRFSTVKNHF--------IKVHIEARK

-GSPRQAYDYC---------TK------E------DT--QS-AHIMLD--LA---TGK--

--RNDI-EDAREL-IS-G--LTPRKIML---A-DSEGRFAHLTTYIET-YYQAR------

-----------L-SNEYAT--KER----------DVLPIYLYGETGSGKSRWVADNLGY-

-----PDVYTVSDYTP-----YD----GYTDQ-KILVFDEFHS--QRPIEEMLRLLDPYP

VE----LPARYHNKQ-A---CYRLVIVISNFPLATQYETA------EYPQRAAFQR----

--RFSNSIDMAKMEY---------------------LV------------RSHL------

---HLEST-----EDESP---PQE----

>pCRESS3|WP_023022037.1

MR--KCSKSTQ--SRSWMATISAEK-----------HR-EEIEEALEQ-----------S

YIG----QLEK---GE-E-GYRHWQLLIDGNSPIRFSTLKNKL--------PTAHLEPRR

-GPIQQAIEYA---------TK------E------ET--EH-GTI-HG--DE---RGR--

--RKDV-DIVREA-VEKG--LSVDEIFL---Q-VP--EAARMTSFVER-LVAAR------

-----------E-RAQNS---APR----------EVEVMWLYGPPGTGKTSLAVDI-GG-

-----DDFYRVTDYSP-----FD----SYAGE-KTLILDEFDG--SMPLSLVLNILDVWP

MS----LPARYANKA-A---AYTQAVMVSNESPWGYYLWE------PASRRQGLAR----

--RIDTIIHIDSYGA------------------------------DETRLRDKF------

----------------------------

>pCRESS3|AKO38848.1

ME-MSGGKNTR--HRRWFLTVPAEGE----GV----SR-DELEQALEP--------D--A

FLG----QLEQ---GKGTNQYRHWQLILVHPEPVRFSTLRRKL--------PTAHLEPVR

--DLRASLAYV---------QK------E------DT--VK-GKI-PG--PG---QGH--

--RSDL-DTLRSR-ILDGQ-ETADELIL---S-DT--GAWRHSRLVGD-LVSAR------

-----------D-RSRQE---KLR----------DVQVRVVFGDTGTGKTSAALSGLQA-

----LGSVCRVTHWAT-----FD----GYDGQ-DSLVLDEFAG--QPPLTELLTWLDVFP

VT----LNARYRARQ-A---AFVRVVLCSNAPPWTWYPWA------PKAQRAALAR----

--RLHLVEEWSGSDN------------------------------ESSEVMRRM------

-------------ANP------------

>pCRESS3|WP_052119337.1

MTDVTGDGQKY--SRCWMLRISKIEDYE--DI----EW-SDICSAFHDAYG-----SGFA

ING----QLEK---GT-KTGYLHFQVLLITKYEKKGQAIIDAI--------AKGGTEKLR

-KNIYAGVRYT---------SK------D------AT--GD-ADLVGE--QT---QGA--

--RNDL-NELRRA-IDDH--MTVDDILR---D-PDSIKSARYVSWLDR-LQRAN------

-----------S-VTPHEA-TEQR----------DVKAHYLYGSPRIGKTRLIYDNISV-

-----NQFYRVTDYQP-----FD----SYVGQ-KVLVLDEYDS--QFPITSINNFLDRYG

CE----LPARYHNSW-A---NWDEVWVISNLPINSQYSDD------NTDKKNAFIA----

--RFTDITYMDKSDD---------------------VL------------SDML------

---DAERN-----GEGSPWVFP------

>pCRESS3|WP_025221073.1

MSEDRAKVETR--SRDWCVTL--EAS----SI----TL-EDYVDHVREFFD--------A

GAL----QMEL---GEHTTGIPHIQAFFQG-KPKRFSTVVRFL--------ESPYVDKRR

-GTVKQAVSYA---------QK------D------DT--VF-GEI-MR--ES---QGK--

--REDL-LDLRKM-VDAG--LTVDEILL---E-DVEGKAARYVGWLDR-LVAAR------

-----------D-AKKMSE--LQR----------DLHCTFIWGKTGVGKTRYALEQGRS-

----LGKVARIVDYRP-----WD----MVDDT-DVIVCDEYNG--QLDLTEFLTILEGYG

AS----MRARYRNRW-P---NYSQVYVLSNTALNEMYSYE------PSERRRALFR----

--RFDRIQYMFKNGE-------------------RRLVD------QEPPISEIL------

--------VDPLR--------PSADGED

>pCRESS3|WP_033495900.1

MAGKKLNRHKR--SRAWMVTIASKYVC---------DW-SVILDELNDMTA-----YGWS

YMG----QAEE---GTGKRGMRHGH-FIVYTPRMRMGTFADHF--------PTAHIDPVE

-RTPKVVERYV---------TK------Y------DTVDES-EGF-PA------GQGK--

--RTDL-KDAVKK-VLAG--AIPDMLMR---D-DP--KLIAYDRYLDR-AYEIA------

-----------M-KEKGR---KMR----------DVHTLYVYGETGVGKSAWLYGKRHN-

-----ADVYRLTDYAP-----WD----NYRGE-SVVIVEDFDG--RMRLDDVLRWTDRYP

VE----LTARYANKH-A---LYTTVIFTSNRPMSGWYGYDE--FE---PKQGPINR----

--RVSTVCASLSDGD---------------------TISRHVA-GADESLRAMI------

---GESTG-----ETAPQPVAPDMSSPT

>CRESSV4|YP_009163936.1

---MAAKRDSA--VKRWCFTLNNYTD----------A--D-CEA-L-KKLT-TD--TCSR

AIV----GKEK---GE--NGTPHLQGFVSLKTRKRLSAMKTFLS-------PRYHFEQAK

-GTDEQNTEYC---------SKE-----G-----D-VLIDV-GEN-VKG-DK---GGG--

--FNEG-ANIKRV-VYHL--KDKEALLE---K-ML-GAYMKYKRSITE-MVSEVKSEQET

KRL----KQDLD-NATLRPWQRELKEYPH-----DRHIIWYIDGQNNGKSWFAKYCVAEL

NAK-TADTHA-----------YN-------GE-RVVIFDLSRT-GHFNYGALESIKNGCV

FS----SKYDSRQKMPI-----PHVIVFANWGPDRS-K---------------LED----

--RWV-TKTWS----------------------------------EQETFQTF-------

----------------------------

>CRESSV4|AHH31482.1

---PKVKQGSQ--ARRWCFTLNNYTE----------K--D-VTS-I-TKLT-FS--NCVF

AKV----GKEV---GD--SGTPHLQGFIHLRKRLRLACVKKLVG-------DRAHCEVAR

-GTDKDNESYC---------GKD-----A-----D-VVLTV-GEP-TIG-EQ---GGG--

--DTIH-SIARRI-ALKL--TDITELQE------W-KAYCRHSKVIQE-LSAAFVKNKNI

ADQ----AAQMS-GKPLRQWQKELKDAPN-----DREIMWYCDSVNTGKTWFSKYLVALH

NGK-SADKYA-----------YN-------GE-RVVVFDLSRS-DHFNYEVIESIKNGLM

FS----PKYTSCTKMPI-----PHVIVFANWMPDES-K---------------LAD----

--RWN-IQSLSDVKE------------------------------QPEQIDSYFD-----

---------------------PMVGDLD

>CRESSV4|YP_009021888.1

-------MNFQ--RKRWCFTVNNYTE----------D--E-FNS-I-KNFC-ER--VAKF

AVM----GREI---GS--GGVPHLQGFISLQNKVRLTGIKAIVG-------QRAHVEPAK

-GTDQKNLEYC---------TKSC--G-V-----D-GATIY-GVP-AGG-EK---GGG--

--TPLS-RRALEC-VRKR--TSISELLD---T-LA-PAYIVHKRAIEE-CASDIAGAQQF

QAE----RARYA-TTRWKPFQFNILKIPH-----PRAVHWFWDAENTGKTFIAKYLVLLH

NGK-SADKYA-----------YK-------GQ-RIVIFDFSRS-DHINYEVMESVKNGIV

FS----PKYESGMKVRT-----PHMICFANERPDTS-N---------------MMD----

--RWE-IHEIT----------------------------------RQARTPTTL------

---------------------PRDSDTH

>CRESSV4|YP_009237559.1

----MAPRGNA--AKRWTFTINNWTL----------A--D-YDK-V-LFTT-KF--EPVF

LIC----GKER---GE--LLTDHLQGFVHLKSKQRLTFLKANLS-------ERAHFECAK

-GSDEDNRDYC---------SKE-----D-----T-EPFIF-GTP-VKS-EK---GGG--

--RITA-QQVIGA-AIAL--TEREPFLE---E-FA-VPVLRHYASFEK-LAASVRRLNAL

EQL----QSEYK-EVRLKVWQQAIINTPH-----PRTIHWYWEATGTGKTWMSKYLVACH

NGK-SNDKYA-----------YA-------GE-KVVIFDFTRS-EHINYEIIESIKNGCY

FN----CKYESGMRIPT-----PHVFCFSNSPPDLS-K---------------MAD----

--RWH-ISQIR----------------------------------LLLSWSVPL------

---------------------PPGTRTV

>CRESSV4|KX388505.1

MD--KNEKYDR--ARRWAFTFHNYTD-------------ENVEY-L-K---PAD--KADF

IIF----GFEL---TK--EGIPHLQGYVEFGTALVRNTVKARLD-------PSSNVSNAQ

-KTREANINYC---------SKIE-SKDEVA---A-KYESF---I-TKT-ERH--QGE--

--RTDW-HLVKDL-AYEKQ--DISEI-E---A-FP-EYAIKYRFAIKD-MIETAKAKQVR

ADF----EEQYE-SAELRPWQSKLVDEPN-----DRKIIWIWENQNIGKSWLSCYLVAKH

NAA-TKDAHA-----------YN-------GE-GIVVFDFSRT-ERLNYQILESIKNKVL

FS----PKYNSCSKY-----KSPHVICLANWPPNRA----------------TMQD----

--RWD-VRHMKDTSE------------------------ELPSCNENSKGDGQIDEII--

--------LSTLQ--------PNSVKID

>CRESSV5|JX904231

MASSAATSGGR--AKNWCFTLNNYTD-------------DEEQC-I-GFSE-ND--HLLY

MIV----GREK---GD--SGTPHLQGYLSLNRRFTLSQIKGWLG-------SRVHLEVAR

-GSPQQNRDYC---------TK------E-----G-DYDEY-GIL-PR--TT---QGK--

--RNDF-ICYQQW-VVSL--MTMHEIAR---E-WP-GLFARYSDRLRT-IAEASLPPIDL

LGD----------SYELRPWQAELASSPD-----PREVMFFVDSDGKGKSYFCQWLLSKS

IGK-RDDAYA-----------ID------ETK-KIFVFDIPRT-EYLQYSVLEMLKDRMV

FS----AKYQSATKIQS-----PHVIVFSNEYPDMS-K---------------MDD----

--RYN-IKEL--------------------------------------------------

----------------------------

>CRESSV5|KR528545

M------PTFQ--SARWCFTVNNPTD-------------ADASA-I-ALGD-GP--LTRY

LVV----GREI---GE--SGTRHLQGFVIFHQVQSRAAVSGYI--------ARAHLEPAR

-ASSVQASDYC---------KK------D-----G-DFDEY-GVC-PR-------SGQ--

--RFDL-QALLKWFIASH--ATAHECAV---E-QP-AAYLKYPR-LIS-LFQARAPPPDG

REG------------ERRPWQHELEDEEN-----DRRVTFIVDQVGAGKTWFQQWFLSKN

IGK-RDDAHT-----------ID------ETK-EVFFFAVPRG-EFLRYEILEMLKDRMV

FS----PKYASRMKFVV-----PHVIVFSNELPDMN-K---------------MLD----

--RYY-IKEVN-------------------------------------------------

----------------------------

>CRESSV5|KR528554

MA-----RQHA--AKRWVFTINNWTA-------------AEQQA-L-ISSD-----NFDY

LCF----GRER---GD--NNTPHLQGYVILKTKLRLNNVKALPGF------RRCHLEVSR

-GTPQQAADYC---------KK------D-----G-DFEEF-GEL-PKG------QGK--

--RSDF-ENLKEW-IKSL--WDDHEIAE---E-YP-SLWGRYRSACES-FRQLFGKPIEV

VDP----------TFEPRVWQQRIIDIPD-----PRKVYFVVDENNTGKSYLSAYLISKF

VGR-RDDAHA-----------IN------PRR-SIFLFDVPRG-EYLQYTIFEQLKNRTV

FS----PKYNSITKIKL-----PHVIIFSNESPDRN-K---------------MHD----

--RYH-VTHIR------------------------------------PRADDNG------

----------------------------

>CRESSV5|KR528556

MP-----RDAP--AKQWCFTLNNYTP-------------AELTA-I-VSAG-----NFDY

LCF----GRER---GN--NNTPHLQGYLILKEKKRFSYVRQLAGL------ERAHWESPR

-STPKQASDYC---------KK------D-----G-DYDEY-GEL-PT--KK---QGQ--

--RTDF-DELKEW-IKEQ--RTDREVAE---E-FP-SLWGRYRSACIS-FLDLFSPHPTL

VQG------------TLRPWQEDLNTRPN-----DRDVMFVVDENNSGKSWFIRYLMTER

IGK-RDDAHA-----------ID------PAK-KIFFFDVPRG-EFMQYAVLEQLKNRLV

FS----PKYESRMKVHI-----PHVVVFCNEEPDRT-K---------------LRD----

--RFR-VTHIR-------------------------------------------------

----------------------------

>CRESSV5|KM874354

--------MAQ--SRSWCFTLNNYVQ-------------ADIDR-L-AFGE-TD--DCTY

LVF----GKEV---GE--SGTPHLQGFAIFPRKLRLRAVKSHIG-------NGAHLECAR

-GSPLQASDYC---------KK------D-----G-DFTEF-GSF-GG--VR---PG---

--SGRF-GQFVEWHYKEGN-QSRALIAA---T-WP-DLYVRYHTKLFE-LVGVLAPKPIL

QEG------------DPNAWQTTLIEAPD-----DRKIYFYVDETGSGKSWLTRYLMTSR

IGK-RDDAHA-----------ID------VTK-KVFLFNVPRT-EFLQYSILESLKDRMV

MS----PKYNSMMKVSV-----PHVVVFSNEEPDRT-K---------------LAD----

--RFA-VTHIR-------------------------------------------------

----------------------------

>CRESSV5|KR528561

---------MT--SCRYCFTVNNPTV-------------QDRER-L-DLAD-----SCNY

LVY----GNEI---GS--SGTPHLQGFVIFPKTKRFNAAKIAIG-------NTAHVECAR

-GSSVQAATYC---------KK------D-----G-DFREF-GEL-P---SS---QGK--

--RTDW-DIYRDW-VTDL--VSKKELVL---A-FP-GFYARYRKACFE-YAEALTPPPIL

TQS------------EPRGWQTRVDGIAN-----DRTIHFVVDPENAGKTWFCSYALTKW

IGK-RDDAYA-----------IS------TEK-SIFLMDVPRN-TFLQYSVLEMLKDRMI

FS----PKYESSFKIYV-----PHVIVFSNEQPDTS-A---------------LAD----

--RIN-IINV--------------------------------------------------

----------------------------

>CRESSV5|KR528551

MN-----RPAA--VKRWVATVNNPTQ-------------QESQT-L-RAIE-----QTSY

AVI----GREV---GE--SGTPHLQCFFIFNNRLRLRQVKAVPGL------QRAHLEPAR

-GTSAQAANYC---------KK------D-----G-DFDEY-GEL-PN-------SGK--

--TTIF-EAFRDW-YKDQ--VTERDILD---H-HP-SI-LRYPHFIEV-CHRQYGRRPTL

VEG------------QLRNWQLELSNMPD-----DRKICFVVDEENKGKSWLTAYWYSNR

IGK-RDDTYA-----------ID------VSK-RVFVFDINRG-EYFQYSVVESLKNRMI

MS----NKYKSVTKIHK-----VHVIVFCNEEPDRT-A---------------MRD----

--RYQ-MKRIT-------------------------------------------------

----------------------------

>CRESSV5|KR528562

--------MSQ--AKRWCFTINNPTP-------------AEEES-L-FHGTPPC--DFKY

LVF----GRET---GE--SGTPHLQGYFELVKKLRIHQIKGTLGF------ERSHLEVAR

-GTSLQASDYC---------KK------E-----N-AFEEF-GAL-P---PP---PGN--

--AAHF-AQLREW-VAAQ--KTIKDVWD---V-FP-TLAARYNRAVME-CIDLFGKKPVL

VEG------------DLRLWQLRLDGIAD-----DRRVIFVIDPENKGKSWLVSYWLSTR

VGK-RDDAYA-----------VD------IST-CLFVFDIPRG-QYIQYGIFEQLKNRVV

FS----NKYSSQTKINT-----PHVFVFANELPDMN-A---------------LAD----

--RYK-VINI--------------------------------------------------

----------------------------

>CRESSV5|KJ641738

-------PSPQ--AVRWVFTLNNYSD-------------DDIEK-L-SLVP--T--VCSY

LIY----GKET---CP--TTTPHLQGYLHFVKRQTLSGAKALLG------NQSYHLEASK

-GTPEQAAAYC---------KK------E-----G-SFVEF-GEP-P----K---GGK--

--RNDW-HDIRDW-MAAL--PSDRDLLD---K-AP-HLWARNRPALLK-MAQLLCPPHRL

VEG------------DPREWQERLSSLPN-----DRTIYFVVDPPNNGKTWFQKWYLSKR

MGK-RDDAYS-----------VD------DTK-SVFMINVPRG-EFLQYSILEMIKDRVV

HS----PKYESTTKFKK-----THVIVFSNEYPLLS-K---------------MQD----

--RPV-FINREGH--------------------IYEYDG-------YPTMDQNL------

----------------------------

>CRESSV5|KR528547

--------MSR--AKNWVYTLNNPTG-------------DEISKEL--FLS-TP--GVIY

HVF----GKET---GD--SGTFHLQGYVIFSQRKRLGQLREIF--------PRGHFEVSR

-GSPHQASDYC---------KK------E-----G-DFKEF-GSL-PP--QA---QGK--

--RTDW-ERLREY-VEQL--RLERDLIL---E-FP-NLVGASYN-----ISHDLHPQKRV

ANG------------SGKPTP------PR-----QPAIEFIVDPENSGKTWFCQYMITQH

IAK-RDDTFA-----------ID------ETR-TIFLFDVPRG-EFLQYNVLEQIKDRLI

FS----PKYSSTLKISN-----VHCVVFSNEEPDVS-Q---------------LSD----

--RLR-VVRLDDS-----------------------------------------------

----------------------------

>CRESSV5|KR528553

--------MAQ--AKRWVFTLNNYSG-------------ADEQL-L-ELSR-ST--DVVT

LIY----GREV---APG-TGTNHLQGMIIFATRKRLRQIRNYPPF------QRAHLEVMR

-STPARAREYC---------IK------D-----G-DFVEY-GE--PVEENR---QGR--

--RNDI-HDIIAWFIEEN--PTQEEVAQ---L-QP-IAMLRYRN-FMD-LARLRAPQPQL

IRN----------GVAREGWQMDLEHAAP-----DRHILFYVDPAGNGKTWFQQYLLTKY

TGN-FADAYA-----------VD------ESK-DIFFFNVPRG-EFFQYRLAESLKDRLV

FS----TKYASGMKIKV-----PHVVVFCNEAPDMT-K---------------LAD----

--RII-IHDL--------------------------------------------------

----------------------------

>CRESSV5|KT945163

M------PSPK--STRWCFTHNNWTE-------------PVFQQSL--LGD-EA--NVKF

GII----GKEV---GE--QGTPHLQGFLILHRQQRLSWVRRHFP-------DGCHWSIAR

-SDSETNRTYC---------KK------D-----G-DFIEF-GVF-PDA------QGK--

--RTDL-DQFIEW-LDEFESPSSPEIAK---T-HP-KMYLRYPRSVRL-AKRRCALFP-V

QEG------------DLNDWQRELEEKPD-----DRTVLFYVDPDGKGKTWFVRRYLTLH

IGQ-IKDAYA-----------VD------TNA-RVFFVNVARS-EFLPYRFLEMLKDRMV

GS----SKYESEMKIHN-----VHVVVFSNEYPDET-K---------------MAD----

--RYD-INTI--------------------------------------------------

----------------------------

>CRESSV5|KP153451

MS-ENRRTASQ--YKAWCYTFNNYSD-------------ADVER-L-K---CKD--GIEV

HVF----GKEV---GD--SGTPHLQGFIKFQSRKRAMFVKQLIG-------GNAHVEYAK

--FPDAAIEYC---------KK------D-----Q-DYFEY-GAL-IK-------AGK--

--RCDI-DDFKSF-IATAR-KYDSELRE---E-FP-KLYCRAARFMKE-YRDRCIEPPR-

-------------LHPLYEWQSKLNSEPD-----DRSITFIIDELNSGKSWYATYYKWNH

PKK-VADAYE-----------LN------ERV-RVVFMDCARAGEYLQYDFLEEIKDGMV

FS----PKYESRTKY-----SNCHVVVLMNEQPDMT-K---------------LQD----

--RYV-IVRI--------------------------------------------------

----------------------------

>CRESSV5|KJ547650

--------MSR--AKHWCFTVNNYTD-------------EDIHK-LKALLL-QP--LVSS

CIYQEVPGQES---AT--PGTPHLQGFISFKTKQSFKFTKNLVS-------DRAHVEVAK

-GTPQQNRIYC---------SKAKDRKIG-----T-EVFTY-GEQ-PK--LL---PGK--

--RNDL-YAFQQY-VKEG-N----DILE---N-HA-SVAARYPRYVRE-YIDLYVNPPEV

PDH------------PLYLWQETLTKISD-----DRQIIFVVDEVNQGKTWFAKKYCRAH

PGK-KADAYA-----------LN------TDL-RVLFLNVTRQ-EHLQYSFLEAVKDGSV

WS----PKYESRTKHQI-----PHIVVMMNQDPDFQ-L---------------LKD----

--RYH-TIYI--------------------------------------------------

----------------------------

>CRESSV5|KJ547646

--------MSQ--GKHWQFTLNNPTQ-------------DERNV-L-ALGD-QP--TTQY

LIY----GDEV---GA--SGTPHLQGHVSFVQRYRFNQVKNWVS-------PRAHLELVR

--LLRRHIEYC---------KK------D-----G-AYLEF-GTP-PDS-AK---DGK--

--RNEL-AEFRAT-VAEGVF-HSPELRE---K-HP-NVMARYPHFANS-IIRDLFPQSAP

PDL------------PLRAWQQRVVELPD-----PRKVYFIVDRQNAGKTSLAKLLHRTH

SGK-VADAYL-----------YK------ITT-KILILDVPRS-ELLQYSFIEMVKDGLL

MS----TKYESVMKT------PPHILVMMNADPDHT----------------ALTD----

--RYHYIIH---------------------------------------------------

----------------------------

>NanoAlpha|YP_003104737.1

-------MARQ--VICWCFTLNN--P----------L-----------PIFLHE--SMKY

LVY----QTEQ---G--ESGNIHFQGYIEMKKRTSLAGMKRLIP-------G-AHFEKRR

-GTQGEARAYA---------MKE-----E-----S-RIEEF-GEF-----IA---SVE--

--DKLR-EVM-----NDMK-KRPIEYIE---E-CC-NTYDKSASTLRE-FRGELKKKKAI

ATW----EL------QRKPWMDEV-DARD-----GRRIIWVYGPQGEGKTSYAKHLVKTR

GGK-TADAFA-----------W-------DHQ-ELVLFDFPRS-EYVNYGVIEQLKNGII

QS----GKYQSVIKY------YVEVIVFANFTPRSG-M---------------FDD----

--RIV-FVYA--------------------------------------------------

----------------------------

>NanoAlpha|HE654123

-------MARQ--VICWCFTLNN--P----------L-----------PLSLHE--SMKY

LVY----QTEQ---G--DSGNIHFQGYIEMKKRTSLAGMKKLIP-------G-AHFEKKK

-GTQGQARAYA---------MKE-----D-----T-RVEEY-GEF-----IP---TIE--

--DKLR-DVM-----QDMK-KRPIEYIE---E-CC-DTYDKSASTLRE-YRGELKKKQAI

ASW----EL------QRKPWMDEV-DARD-----GRRIIWVYGPLGEGKTSYAKHLVKTR

GGN-TADAFA-----------W-------DHQ-ELVLFDFPRS-EYVNYGAIEQLKNGII

QS----GKYQSVIKY------YVEVIVFANFTPRSG-M---------------FDD----

--RIV-FVYA--------------------------------------------------

----------------------------

>NanoAlpha|AKO71308.1

MI--DDRMARY--VVCWMFTINN--P----------T-----------LPVMRD--EIKY

MVY----QVER---G--QEGTRHVQGYVEMKRRSSLKQMRSFFP-------G-AHLEKRK

-GSQEEARSYC---------MKE-----D-----T-RIEEF-GAF-----KL---SCN--

--DNLF-DVI-----QDMR-KRPLEYLY---D-CP-NTFDRSKDTLYR-VQSEMNKTKAM

NSW----RT------SFSAWTSEV-ENPC-----HRRIIWVYGPNGEGKTTYAKHLMKTR

GGK-SLDCRL-----------Y-------NYE-DIVIFDIPRC-DYLNYGLLEEFKNGII

QS----GKYEPVLKI------YVEVIVMANFLPKEG-I---------------FED----

--RIK-LVSC--------------------------------------------------

----------------------------

>NanoAlpha|JF957636

-------MARY--VVCWMFTINN--P----------T-----------LPVMRD--EIKY

MVY----QVER---G--QEGTRHVQGYVEMKRRSSLKQMRVFFP-------G-AHLEKRK

-GSQEEARSYC---------MKE-----D-----T-RIEEF-GAF-----KL---SCN--

--DNLF-DVI-----QDMR-KRPLEYLY---D-CP-NTFDRSKDTLYR-VQAEMNKTRAM

NSW----RT------SFSAWTSEV-ENPC-----HRRIIWVYGPNGEGKTTYAKHLMKTK

GGK-SLDCRL-----------Y-------NYE-DIVIFDIPRC-DYLNYGLLEEFKNGII

QS----GKYEPVLKI------YVEVIVMANFLPKEG-I---------------FED----

--RIK-LVSC--------------------------------------------------

----------------------------

>NanoAlpha|AIF34798.1

MS-SGRREALK--MRAWCFTHNNYAA----------A--D-----LP-LCK-----D-RY

VVW----QHE-------KVDTDHIQGYIELSKPQRISAMIKWL--------PGAHFEERR

-GTPDQARKYC---------MEE-----D-----T-RVEER-GSYG----TT---QGK--

--RSDI-DVVREA-IAAGA--DRREVYN---A-HS-DIAAKYPRYVET-MLR-FAKEDAI

LVF-----------EPRQGFQTDLLDMAD-----SRSIHWVYDRVNNGKTYFAKYLVDKF

GGK-SVDAYA-----------YG-------GE-SIVIFDYVRD-EYVGYGVIEQLKNGIA

MS----TKYESITKRNI-----PHVIVLANFKPQEG-K---------------FSD----

--RIK-MINVT-------------------------------------------------

----------------------------

>NanoAlpha|AAA51422.1

-------MSSP--SLKWCFTLNYSSA----------A--E-REN-F-LLLK-EE--DVHY

AVV----GDEV---AP-ATGQKHLQGYLSLKKRIRLGGLKKKYG-------SRAHWEIAR

-GTDEENSKYC---------SK------E-----T-LILEL-GFP-VV--NG---SNK--

--RKIS-EMV----------RSPDRMKI---E-QP-EIFHRYQS-VNK---LK--KFKEE

FVH----PC------LDRPWQIQLTEAPD-----DRSIIWVYGPYNEGKSTYAKSLIKKD

GGK-KENLFS-----------YV----DEGSD-KHIVFDIPRC-DYLNYDVIEALKDRVI

ES----TKYKPIKIVGK-----IHVIVMANFMPDFC-K---------------IED----

--RIK-IIYC--------------------------------------------------

----------------------------

>NanoAlpha|ACB86656.1

---------MS--ASRWTFTLHYSDA----------T--E-RGK-F-LTLK-EE--DVHY

AVV----GDET---AP-NTGRKHLQGYLSLKKRFRISGIKKKYS-------SRAHWEKAR

-GSDYDNKAYC---------SK------E-----A-LILEL-GVP-CQ--TG---SNK--

--RKLA-DMV----------RSPERMKI---E-QP-EIFHRYAS-VKK---MK--EFKER

YVY----PI------LDRPWQVQLTELPD-----DRTIIWVFGPKNEGKSTYAKSLIQKD

GGK-KENLFA-----------YV----DEGST-KNVVFDLPRT-EFINYDVIEALKDRVI

ES----TKYKPVKYLNT-----VHVLVMANFLPDMC-K---------------IED----

--RIK-IVAC--------------------------------------------------

----------------------------

>NanoAlpha|AAA51426.1

---------MS--SFKWCFTLNYSSA----------A--E-RED-F-LLLK-EE--DVHY

SVV----GDEV---AP-ATGQKHLQGYLSLKKSIRLGGLKKKYG-------SRAHWEIAK

-GSDEQNRRYC---------SK------E-----T-LVLEL-GTP-VV--PG---SKK--

--RKLL-DRF----------ESPEELKM---D-DP-SKYRRCLA-VES---IKDARINSE

WVH------------ELKEWQNKLIQHPD-----DRSIIWVYGPNGEGKSTFARYLLKPG

GGK-TSDMHI-----------ITM-----DPD-NHWIIDIPRS-DYLNYGVIEQIKNRVL

IN----TKYEPCVIRGQ----NVHVIVMANVLPDYC-K---------------IED----

--RIK-IINC--------------------------------------------------

----------------------------

>NanoAlpha|YP_009058890.1

---------MA--CSNWVFTRNFQGA----------L-----------LLSFDE--RVQY

AVW----QHE-------RGTHDHIQGVIQLKKKARFSTVKEIIG-------GNPHVEKMK

-GTIEEASAYV---------QKE-----E-----T-RVASY-GDL-LK--RG---SHR--

--RKTM-ERY----------EDPEEMQL---K-DP-DTALRCNA---K-RLKED-FMKEK

TKL------------QLRPWQKELHDLPD-----DRTIIWVYGPDGEGKSMFAKELIKYG

GGK-TQDLYM-----------YA----Q-DPE-RNIAFDVPRC-EMMNYQAMEMMKNRCF

AS----TKYRSVDLCKN-----VHLVVFANVAYDPT-K---------------IED----

--RIV-IINC--------------------------------------------------

----------------------------

>NanoAlpha|KC978991

---------MA--CANWVFTRNFQGA----------L-----------SLSFDE--RVQY

AVW----QHE-------RGTHDHIQGVIQLKKKARFSTVKEIIG-------GNPHVEKMK

-GTIEEASAYV---------QKE-----E-----T-RVASY-GDL-LK--RG---SHR--

--RKTM-ERY----------EDPEEMKL---K-DP-DVALRCNA---K-RLKED-YCSCF

SSF------------KLRPWQIELHRVPD-----DRSIIWVYGPDGEGKSTFAKELIKYG

GGK-TQDLYM-----------YA----Q-DPE-RNIAFDVPRC-EMMNYQAMEMMKNRCF

AS----TKYRSIDLCKN-----VFLVVFANVEPDPT-K---------------IGD----

--RIV-IINC--------------------------------------------------

----------------------------

>NanoAlpha|NP_619760.1

---------MA--SKRWCFTLNYKTA----------L--E-RET-F-ILFSRDE---LNY

FVC----GDEI---AP-TTGQKHLQGYVSMKKLIRLGGLKKKFG-------SIAHWEIAK

-GDDFQNRDYC---------TK------E-----T-LIAEI-GAP-VK--KG---SNR--

--RKIM-EIY----------EDPEEMKL---R-DP-DTALRCKA---K-KLREE-YCSEV

SVF------------SLRPWQIELHRAPD-----DRTIIWAYGPDGEGKSTFAKELIKYG

GGK-TQDLYM-----------YA----Q-DPE-RNIAFDVPRC-EMMNYQAMEMMKNRVF

AS----TKYRPVDLCKK-----VHLIVFANVAPDPT-K---------------LED----

--RIV-IINC--------------------------------------------------

----------------------------

>NanoAlpha|NP_619759.1

M------PTLQ--GTFWCFTLNFSGD---------------------ASLSFNE--RVQY

ACW----QHE-------RVSHDHLQGYIQMKKRSTLKMMKELL--------PGAHLEVSK

-GTPEEASDYA---------MKE-----E-----T-RVATY-GEL-LK--KG---SNK--

--RKLL-DRY----------ENPEDMEL---E-DP-AKARRCRA---K-IDKEKFIAEFK

VED------------DEQEWKKILEKEAS-----PRSILWVYGPQGEGKTSKAKELITRG

GGK-KDDAYS-----------YV----E-DPT-RHVVFDIPRD-EYCNYSLIEMLKDRII

IS----NKYEPITNCYN-----IHVIVMANFLPDVT-K---------------IED----

--RIK-IIYC--------------------------------------------------

----------------------------

>NanoAlpha|U16735

M------PTRQ--STSWVFTLNFEGE---------------------IILPFNE--SVQY

ACW----QHE-------RVGHDHLQGFIQFKSRNTLRQAKYIFN-------GNPHLEIAR

--DVEKAQLYA---------MKE-----D-----S-RVAEY-GLF-IK--RG---SHK--

--RKLM-ERF----------EDGEEMKI---A-DP-SLYRRCLS---R-KMAEEQRCSSE

WNY------------DLRPWQEEVMHLPD-----YRTIIWVYGPANEGKSTFARHLLKDG

GGK-TQDMHL-----------VT----A-EPK-NNWVFDIPRV-EYVNYGVIEQVKNRVM

VN----TKYEPCVMRNH----PVHVIVFANVLPDLG-K---------------LED----

--RIK-LIRC--------------------------------------------------

----------------------------

>NanoAlpha|KC979052

M------PSQK--STYWVFTLNFKGE---------------------IILSLDT--RVQY

ALW----QHE-------YVSHHHLQGFIQMKAQSTLGQMKALIP-------G-AHFEVMK

-ADSDQARSYA---------MKE-----D-----S-RLEEY-GLY-IK--KG---SHK--

--RKVM-ERF----------SEPEEMKV---E-DP-SLYRRCLS---R-KMTEEQRSSST

WDY------------DLRPWQDSILEAPN-----YRDVLWVYGPNGEGKSTFARHLLKDG

GGK-TQDMHL-----------IT----A-EPK-NNWVFDIPRV-DYINYGVIEQVKNRVM

VN----TKYEPVIMRNN----PVHVIVFANCLPDVT-K---------------LED----

--RIK-MIYC--------------------------------------------------

----------------------------

>NanoAlpha|KF471057

------MPTIQ--SQWWCFTVFFSAT----------A-----PD-L-VLFENTH---VSY

ACW----QEEE---SP-TTRRRHLQGYLQLKGKRSLAQVKALFG-------DNPHLEKQR

-AKTDEACDYC---------MKE-----E-----T-RVSEF-GDY-CP--SG---SHK--

--RRQR-ESV----------RSPVRMAE---E-NP-SLFRRVKA---K-IAEEEFQKTAR

EIQ----IL------NLKSWQSRLQTLPD-----DRTIFWVYGPTGEGKSTFARDLYRSG

GGS-ADNSYQ-----------YI----G-QLG-NNIVFDIPRD-DYLQYSLIEMFKDRLI

VS----NKYEPLMAPNC-----IHVVVMSNFMPDFE-K---------------IQD----

--RVH-VIPCRPC-----------------------------------------HHII--

--------MKC-----------------

>NanoAlpha|JX458742

---MGSFSSPR--SRWWCFTLNFSGD----------P-----PE-L-AWLARDE---IKY

ACW----QHEK---G----THDHMQGYLQLKKPSRLTAVRKLFG-------NRPHLEVQK

-ARAEDARDYC---------MKD-----E-----S-RVAEI-GEF-IP--QG---SHK--

--GRMR-DLV----------RSPERMAE---E-NP-SVYRRVLA---A-QSVERF-RSDP

SLL----PD------PLRDWQICLLELPD-----NRSIIWIYGPAAEGKTMMAKELFRRG

GGT-ADNKYQ-----------YA----V-EVE-SHVVFDIPRD-DYIQYSLIEELKNGMI

VS----NKYEPIRVVSG---REVHVVVMCNFMPDYS-K---------------IPD----

--RIV-IVYCRKC-----------------------------------------ETCL--

--------IGC-----------------

>NanoAlpha|YP_008169853.1

--------MAK--SRNYCFTL-FSYV----------L-----------LFNLPD--WANY

LVF----QEEE---SP-STGRRHIQGYVNLKSPQSFSFLKKKLG-------DGVHLEQAR

-GSASCNRDYC---------RKT-----D-----S-RVSEF-GIL-AE--QG---SKK--

--RKTM-ESF----------EDPEELRL---S-DP-KLYRRCLA---T-RVNTEF---AG

LVL----PV------LDRPWQLLVEKVPD-----DRTIIWVYGSQNEGKTTWAKSKVQAG

GGK-GENKYS-----------YA------EHL-GHAVFDIPRQ-DVLQYTVLEEIKDRLI

RS----SKYEPIDFNDQ-----VHVVVLSNFLPQLD-SHDS--RG-NLIKKQMLRD----

--RVV-IVNIAES-----------------------------------------NETV--

--------FH------------------

>NanoAlpha|HM163578

-------MTSQ--SRNWCFTL-FSYV----------L-----------LFSLPE--WAEY

LVF----QEEQ---CS-STGRKHIQGFVTLKRSQRLSFLKNKLG-------DGVHLEIAK

-GSASSNRDYC---------TKD-----D-----T-RSSEF-GVL-AE--QG---SRK--

--RKTM-ESF----------EDPEEMRL---S-DP-KLYRRCLA---T-RVNKEF---SG

LVL----PV------LDRPWQLLAEKVPD-----DRTIIWVYGSQNEGKTTWAKSKIQAG

GGK-GENKYS-----------YA------DHL-GHAVFDLPRQ-DVLQYTVLEEIKDRLI

RS----SKYEPIDFNDR-----VHVVVLSNFLPQLD-LYDS--RG-NLVKKQMLRD----

--RVV-IINIDES-----------------------------------------DETV--

--------FH------------------

>NanoAlpha|YP_009246456.1

--------MSH--SRNWCFTI-FNYV----------L-----------LFTLPE--WANY

LIY----QEEE---CP-TTKKRHIQGYVNLKRNQRFAFLKKKLP-------DGTHIEACK

-GSSSSNRDYC---------RKD-----D-----T-RTDEF-GVF-AE--TG---SNK--

--RKTM-ERF----------EDPEELRL---A-DP-KLYRRCLA---T-KVNTEF---GG

LVL----PV------PDRPWQLVAQKVPD-----DRTIIWVYGSENEGKTTWAKTKIQDG

GGK-GENKYQ-----------YA------EHL-GHCIFDIPRQ-DNLQYTVLEEIKDRLI

RS----SKYEPIDFNDK-----VHVVVLSNFLPCLD-SYNN--RG-ELVKKPLLRD----

--RVF-LINIDES-----------------------------------------DDLT--

--------FD------------------

>NanoAlpha|ALK03646.1

--------MSQ--QRNWCFTL-FNYV----------L-----------LFSLPP--WANY

IVY----QEEI---CP-DTSRKHIQGFINLKRPQRFSFLKKNLP-------DGAHIESCK

-GSASSNRDYC---------TKD-----A-----S-RFAEF-GVF-AE--SG---SNK--

--RKTM-ERF----------DDPEELRL---A-DP-KLYRRCLA---T-KVNFEF---SS

VVL----PV------FDRPWQLLVEKIID-----DRTIIWVYGSQNEGKTTWAKKKVQDA

GGK-GENKYQ-----------YV------EHL-GNCIFDIPRQ-DNLQYTVLEEIKDRLI

RS----SKYEPIDVNDN-----VHVVVLSNFLPQLD-VYDS--RG-NVVKRQLLRD----

--RLC-IVNIDDG-----------------------------------------NSVI--

--------FH------------------

>Smaco|AIY31250.1

MH-SETTTHRRKNLQTWMITG--PR-----TI----HK-RIIRELFD--------QDVKE

YII----AKET---G--KGGYEHWQIRCKASRPDFFEYVHDR--------EPRFNIQKAT

-----EDFDYE---------RK------D-----G-HFWCS-EDN-NEI-LKC--RGELG

--M--W-QQLM----------------K----------------RLKK------------

---------------------------QS-----VREIDVVLDPANKGKSFATIALWERR

-------ALVVP---RAQTFVCS----AWKGE-WLIIIDIPRSL----YETMEEIKDGLV

FD----WRYSGKTRN-I---RGTKLVVFTNEPL----N------------LKGLSD----

--RWK-LHGIKGT-----------------------------------------------

----------------------------

>Smaco|KT862221

-----------------MMTI--PRRI---IC----SK-KLLSYIFE--------QDVKR

YIV----AIEK---G--KNGLDHFQIRLSCSDPDFFEHMKDW--------CEWAHVEKAT

-----DNFDYE---------RK------E-----G-RFWTS-DDT-TEI-RIC--RRELG

--R--W-QQIM----------------Q----------------VLKK------------

---------------------------QD-----VRTIDVVLDPVARGKSHFAIALWERG

-------ALVVP---RYSTFVCS----AYRGR-KIIIIDIPRAL----YETMEEMKDGLV

FD----PRYSGKTRN-I---RGTKVLVFTNNPL----D------------LKKLHD----

--RWN-LHGISGT-----------------------------------------------

----------------------------

>Smaco|KT862218

------------------MTG--PR-----TI----HK-RIIREIFK--------KDVKK

YII----AMET---G--NGGYEHWQIRCTASRPDFFEYVHDR--------EPRFNIQKAT

-----ESMEYE---------RK------E-----G-RFWSS-EDT-TEI-RQC--RGELG

--R--W-QQIL----------------K----------------VLKN------------

---------------------------QD-----VRTIDVVLDPVARGKSHFTIALWERG

-------ALVVP---RYSTFVCS----AYKGE-KIIIIDIPRAL----YESMEEMKDGLV

FD----PRYSGKTRN-I---RGTKVLVFTNNPL----D------------LKKLHD----

--RWN-LHGITGT-----------------------------------------------

----------------------------

>Smaco|KM573775

-----------------MLTI--PAK-------------RHWLKLFE--------VNVKK

HVV----GLEE---G--KNGYKHWQARIQLSEISFFQYMKIYY--------PKAHIEEAS

-----NTWEYE---------RK------E-----G-KFWTS-EDT-ASI-LAV--RGSL-

--R--E-QKIL----------------Q----------------ILES------------

---------------------------QG-----DREIDVWLDPSNHGKSWLTVHLWETG

-------ALVVP---RSSTFICS----SWKGE-PIVIIDIPRSL----LETMEELKDGLV

FD----HRYTGRTRN-V---RGVKVMVFTNSEL----P------------LKKLKD----

--RWR-LHGIAGS-----------------------------------------------

----------------------------

>Smaco|KM573771

-----------------MVTV--PTK----HA----CK-RQVKMILE--------IDTKR

YIF----AQEK---G--KNGLDHWQLRIQI-KEENFERLQKLF-------CNKAHIEVAN

-----DSWEYE---------RK------E-----G-KFWSS-EDT-KEI-LKI--RGKL-

--R--E-QKIL----------------Q----------------ILSS------------

---------------------------QG-----DREIDVWLDPTCHGKSWLTIHLWETG

-------ALVVP---RSSTFVCS----SWRGE-DIIVIDVPRAL----LETMEELKDGLV

FD----HRYTGKTRN-V---RGTKLMVFTNSPL----P------------LNKLTD----

--RWR-LHGISGS-----------------------------------------------

----------------------------

>Smaco|YP_009030025.1

-------------MQTYMMTI--PRT-----V----SK-RALRIMIE--------KDCKK

WII----GKEK---G--KNGYEHWQIRIETSNDNFFQWIQDHI--------PTAHVEKSD

NGV--DECRYE---------TK------E-----G-QYVTY-SDR-VQN-LIQ--RGAF-

--R--N-QRAI----------------Q----------------ALEA------------

---------------------------TN-----DRQVVVWYDETNVGKSWFTGALWERG

-------AYVTP---PTITWVAS----CYIDR-PYVIIDVPRSL----YSAIESIKDGLI

YD----TRYHSRMIN-I---RGVKVLVMTNTMP----K------------LDKLKD----

--RWC-I--CT-------------------------------------------------

----------------------------

>Smaco|KJ577810

-------------MQTYVMTI--PRT-----V----SK-RALRIMIE--------KDCKK

WVI----GKEE---G--KNGYKHWQIRIETSNDDFFEWMQDHI--------PTAHVEKSE

NGV--DACRYE---------TK------E-----G-QYVMY-SDR-VQN-LMQ--RGAF-

--R--N-QRAM----------------Q----------------ALQA------------

---------------------------TN-----DRQVVVWYDETNVGKSWFTGALWERG

-------AYVTP---PTITWVAS----CYIDR-PYVIIDVPRSL----YSAIESIKDGLI

YD----TRYHSRMIN-I---RGVKVLVMTNTMP----K------------LDKLKD----

--RWC-I--CT-------------------------------------------------

----------------------------

>Smaco|YP_009054985.1

-------------MQAYMMTI--PRT-----V----SK-RALRIMID--------VDCKK

WII----GKEE---G--KNGYKHWQIRIETSNDSFFEWMQDHI--------PTAHIERTE

CGV--DACRYE---------AK------E-----G-QYVMY-SDR-PQN-LMQ--RGEF-

--R--N-QHAL----------------Q----------------ALQQ------------

---------------------------SN-----DREVVVWYDETNVGKSWFTGALWERK

-------AYVTP---PTITWIAS----CYIER-PYIIIDIPRSL----YSAIESIKDGLI

YD----TRYHSSMMN-I---RGVKVLVMTNTMP----K------------LDKLKD----

--RWC-I--RT-------------------------------------------------

----------------------------

>Smaco|YP_009022025.1

-------------MKKYIMTV--PRS-----V----PK-KALKIMID--------VDCKK

WII----GKER---G--KNGYEHWQIRIETSNDEFFKWCKHHI--------PAAHIEEAQ

QGV--DECLYE---------RK------E-----G-QFWTS-SDR-VET-LHQ--RGTL-

--R--R-QRAL----------------L----------------ALQS------------

---------------------------TN-----DREVMVWYDANNVGKSWFCGALWERG

-------AYVTP---PTVTWVAS----CYMDR-PYVIIDIPRSL----YCAIESIKDGLI

YD----TRYHARMIN-I---RGVKVLVLTNTLP----K------------LDKLRD----

--RWC-I--FE-------------------------------------------------

----------------------------

>Smaco|AMR73073.1

----------M--VQAWMITA--PRT----HV----SK-RAIRIMID--------TDCKK

WIV----AKER---G--KNGYEHWQIRIESSNTGFFEWCKLYI--------PTAHVEKAE

RGV--DESRYE---------TK------E-----G-QYVLY-TDR-VEI-LKQ--RGKM-

--R--N-QRAL----------------E----------------ALES------------

---------------------------TN-----DREVLVWYDEGNVGKSWLTGALWERG

-------AYYVP---PTVSWVAS----CYQSR-PYVIIDIPRSL----YVAIESIKDGLV

YD----TRYHAQCIN-I---RGVKVLVLTNTQP----K------------LDKLQD----

--RWR-I--CVGR-----------------------------------------------

---------------------ITLWADF

>Smaco|KX838318

----------M--VQAWMITA--PRA----HV----SK-RAIRIMID--------TDCKK

WII----AKEK---G--KNGYEHWQIRVESSNTGFFEWCKQHI--------PTAHVEKAE

RGV--DECRYE---------TK------E-----G-QYTCY-SDR-IPV-LKQ--RGKL-

--R--N-QRAL----------------T----------------ALEA------------

---------------------------TN-----DREVLVWYDEGNVGKSWLTGALWERN

-------AYYVP---PTITWVAS----CYQDR-PYLIIDIPRSL----YVAIESIKDGLV

YD----TRYHSRLIN-I---RGVKVLVLTNTKP----K------------LDKLED----

--RWR-I--CVGQ-----------------------------------------------

---------------------YNTMGRL

>Smaco|KX838317

----------M--VQAWMITG--PRSES--RI----SK-KAIMIMLS--------KDCKK

WTI----GQEV---G--KNGYKHWQIRVESSNTNFFKWCKQHI--------PSAHVEKAE

KGV--DESRYE---------TK------E-----G-QYTQY-TDR-VEI-LKQ--RGKM-

--R--N-QRAL----------------E----------------ALEA------------

---------------------------TN-----DREVLVWYDESNVGKSWLTGALWERG

-------AYYVP---PTVSWVAS----CYQSR-PYIIIDIPRSL----YAAIESIKDGLV

YD----TRYHAQCIN-I---RGVKVLVLTNTKP----K------------LDKLAD----

--RWR-I--CV-------------------------------------------------

----------------------------

>Smaco|KP233189

---------------MYMLTI--PRD----KV----SK-RELRIMLD--------KDCKK

WII----GKET---G--KNGYKHWQIRLETSNEEFFDWCKKHI--------PTASIRKAE

--V--PKWDYE---------AK------E-----G-QYWTS-SDR-TDN-LIQ--RGEF-

--R--N-QRAI----------------Q----------------ALRA------------

---------------------------TN-----DREVLVWYDEGNVGKSWFTGALWERG

-------AYVTP---PTVTFVAS----------PYVFIDIPRSL----YCAIESIKDGLV

YD----SRYQGRMVN-I---RGVKIIVMTNNKP----D------------LDKLYD----

--RWR-M--VV-------------------------------------------------

----------------------------

>Smaco|KY086298

MN--KQAGECQ-SMQTYMLTI--PR-----KV----HK-RTLKIMLE--------QDVKK

YII----AKER---G--FGGYEHWQIRLKTSNKNFFIWCKINI--------PEAHVEEAM

-----DTWDYE---------RK------E-----G-VYWTS-DDT-NEI-RAL--RGKP-

--N--K-QRVL----------------K----------------LLKY------------

---------------------------QG-----DRNILVWYDPVKAGKSWIVGHLWEQG

-------ACYVP---PTLTWVHS----AYDNE-GLIIIDIPRSL----YTAIETIKDGLV

YD----PRYSARMKN-I---RGVKVLVMTNTYP----R------------VSALED----

--RWD-I--INGE-----------------------------------------------

----------------------------

>Smaco|YP_009252320.1

-------------MQTWVLTI--PRRT---VC----SK-KLLNYILE--------QDTKR

YII----GIEK---G--KNGLEHFQIRLSCSDPEFFEHMKEWY--------PYAHIEKSD

VGINSESMEYE---------RK------E-----G-RYWTS-MDT-TEI-RIQ--RGKP-

--N--T-QRVL----------------E----------------VLRR------------

---------------------------TN-----DREIVLWYSDKSIGKSWLVGHLWETG

-------AYVCQ---PQ-TDVAS----EYIKR-PCIVVDLPRTL----YCALESIKDGLL

KD----TRYSSDTIN-I---KGVKVLVTSNTLP----K------------FDSLFD----

--RWI-V--IEIE-----------------------------------------------

----------------------------

>Smaco|YP_009054987.1

----------M--TKTWMLTV--PRN----NT----AK-EAISKWLR--------ADVHK

WIC----AMET---G--ADGYDHWQIRLQV--NKTWEKLKEEWG-------PKAHIEEAS

-----DVWDYE---------RK------S-----G-LFFSS-QDT-PEV-RKC--RGHL-

--T--R-QAVL----------------R----------------AVQS------------

---------------------------TN-----DRQVVVWYDPENKGKSWLLGHLYETG

-------AWVVQ---AQ-TDVAS----EYINR-PMVVIDIPRTL----YVAIERIKDGLI

KD----PRYSSKTVH-I---RGVKILVTCNTMP----K------------LDKLED----

--RWI-I--IDAL-----------------------------------------------

---------------------ARIGE--

>Smaco|KJ577813

----------M--TKTWMLTV--PRN----NT----AK-EAISKWLR--------ADVHK

WIC----AMET---G--ADGYDHWQIRLQV--NKTWEKLKEEWG-------PKAHIEEAS

-----DVWDYE---------RK------S-----G-LFFSS-QDT-PEV-RKC--RGHL-

--T--R-QAVL----------------R----------------AVQS------------

---------------------------TN-----DRQVVVWYDPDNKGKSWLLGHLYETG

-------AWVVQ---AQ-TDVAS----EYINR-PMVVIDIPRTL----YVAIERIKDGLI

KD----PRYSSKTVH-I---RGVKILVTCNTMP----K------------LDKLED----

--RWI-I--IDAL-----------------------------------------------

---------------------ARIGE--

>Smaco|YP_009118276.1

----------M--TQTWMLTV--PRR----KT----EK-EGIYKWLR--------DDVHK

WTV----AMET---G--NNGYDHWQIRFQV--GKTFKQLKKEWG-------PKAHIEEAS

-----DTWEYE---------RK------S-----G-MFFSS-DDT-PEV-RKC--RGRL-

--N--R-QTIV----------------R----------------AVQD------------

---------------------------TN-----DREIVVWYDPNNKGKSWLLGHLYETG

-------AWVIQ---AQ-TDCAS----EFINR-PIVVIDIPRTL----YVAIERIKDGLI

KD----PRYNSKTVH-I---RGVKVLITCNTMP----T------------RDKLAD----

--RWV-I--VE-------------------------------------------------

----------------------------

>Smaco|KU043428

AAQRQRLQSIM--THTWMLTV--PRR----KT----AR-EGISKWLR--------DDVHK

WTV----AMET---G--DNGYDHWQIRLQV--NKTFKQLKKEWG-------PKAHIEEAS

-----DTWNYE---------RK------S-----G-LFFSS-NDT-PEV-RKC--RGRL-

--T--R-QAVV----------------Q----------------AVQG------------

---------------------------SN-----DRQIVVWYDPNNAGKSWLLGHLYETG

-------AWVVQ---AQ-TDCAS----EYIQR-PFVVIDIPRSL----YVALERIKDGLI

KD----PRYGSKTVH-I---RGVKVLVTCNTKP----K------------LDKLAD----

--RWV-I--MNTL-----------------------------------------------

---------------------AEVADRI

>Smaco|KU043430

---LRRIRETM--GKTYMLTI--PRN----DT----YR-EKIFAWFR--------KDIHK

WVL----GAEK---G--SGGYEHWQMRVQC--RFGFEELKVLF--------PTAHIEECS

-----DKWTYE---------AK------E-----G-VYWRS-NDR-REN-IQQ--RGKL-

--R--A-QRVI----------------E----------------RADG------------

---------------------------TN-----DREVVVWYDQEKAGKSWLTGAMWERG

-------AYFSV---AD-SDIAS----EYLKR-PYIIIDIPRAL----YEAIERIKDGLI

KD----PRYSSEAVN-I---HGVKVIVMTNTMP----K------------LDKLAD----

--RWK-I--ITEL-----------------------------------------------

---------------------AE-----

>Smaco|KU058671

-------------MTIWMVTL--PRN----DT----YK-ESIWMWFV--------KDVKK

WVF----GLEK---G--RFGYKHWQIRFKS--NLKFEDLKVMF--------PTGHIEEAS

-----DTWTYE---------TK------E-----G-VFWKS-TDR-PEN-RAQ--RGKL-

--N--D-QGVI----------------R----------------ALQS------------

---------------------------TN-----DREVVLWYDSEKKGKSWLTRALWERG

-------AYFIV---AD-NDVAC----EFIKR-PYVIIDIPRAL----YEAIERIKDGLI

KD----PRYSSESIN-I---SGVKVLVNSNSLP----E------------LDNLKD----

--RWK-IYPCTDI-----------------------------------------------

---------------------RQ-----

>Smaco|AIY31246.1

-------------MGIYMVTA--PRKGS--GT----GK-EKWRTFFY--------IDVHK

WTL----GAET---G--KGGYKHWQVRLQISDNEVFNKIKDEF--------PTAHIEKGS

-----DTWSYE---------CK------E-----K-MHWTS-NDT-PNI-LRV--RGHM-

--R--Y-QDVI----------------E----------------AVRR------------

---------------------------QN-----DRQIAYWYDPENKGKSWLVNHLFESC

-------AWYVP---PTLSWVAS----VYINR-EILVIDIPRSL----YTAIETIKDGLV

YD----PRYHAQMIN-I---RGVKILVLCNHEP----K------------LDALAD----

--RWY---AVALT-----------------------------------------------

----------------------------

>Smaco|YP_009163761.1

MR-----PVSM--TKCYMITA--PRDE---------EM-YRIWRYIN--------SDVHK

WII----ASEI---G--RNGYKHWQIRIKTSDPDEYRKVKIGTG---WA-IPRSHVEECS

-----DDWDYE---------TK------E-----G-RYLAS-WDT-PEV-RKL--RGQP-

--R--H-QAII----------------N----------------RLES------------

---------------------------TN-----DREVMVWYDPTNSGKSWLVGHLYETG

-------AYYLP---PTMSMMAS----LAIQR-RYVVIDIPRTL----YCAIETIKDGLI

VD----PRYSARPIN-I---RGVKVLVLSNDRP----S------------LDKLVD----

--RWV-V----NT-----------------------------------------------

----------------------------

>Smaco|YP_009054993.1

-------------MKTYMLTI--PRE----RA-RIGTE-PRLMRYIR--------EDVKK

WIV----AMET---G--RRGYEHWQVRLQA----VTEKQKVGVG---WI-IPQAHVEECS

-----DNWEYE---------AK------E-----G-RYWAS-WDT-VEV-HRM--RGRP-

--R--Y-QALL----------------N----------------KLRT------------

---------------------------TT-----DREVMVWYDPENNGKSWLVGHLFETG

-------AYYLP---PTLSTMAS----LAHQK-PYVIIDIPRTL----YCAIESIKDGLI

MD----PRYSARPIN-I---RGVKVLVLTNEMP----K------------LDALED----

--RWI-I--E-NT-----------------------------------------------

----------------------------

>Smaco|AMR73071.1

--------------MDIMATI--GRT----G-----YS-GEIMAIIR--------EDIHK

WII----GAEV---G--AGGYQHWQCRFKTSPEEMTEKLRARIG------GPSIYTAECS

-----DNWEYE---------AK------E-----G-AYLAS-WDT-MKV-RST--RGKP-

--S--Q-QVAL----------------Q----------------ALRD------------

---------------------------TN-----DRQVVVWYDPQNIGKSWLTNHLYEQG

-------AYCIP---ATMNTVAS----LAVKR-PYVIIDIPRSL----YTAIESIKDGLI

MD----PRYSAQPIN-I---RGVKVMVMTNTMP----K------------IDKLSD----

--RWV-I--H-TN-----------------------------------------------

----------------------------

>Smaco|ADB24799.1

---MHLFTEVV--SVDIMTTM--QRT----HT----NA-ERWFKIFR--------DDIHK

WVI----GLEE---G--KGGYGHWQVRCNIE---YLRAVFSWLG---PI---SIWTEECS

-----DKYTYE---------TK------E-----G-RYWAS-WDT-MGA-RQQ--RGKM-

--R--N-QGAV----------------Q----------------ALQR------------

---------------------------TN-----DREIVVWYDEQNMGKSWLCGHLFETG

-------AYYIP---PYMSTVAS----LVLQR-PLIVIDIPRSL----YTAIEAIKDGLI

MD----PRYGARPVN-I---HGIKVIVLTNTKP----K------------LDKLED----

--RWV-L--YDDY-----------------------------------------------

----------------------------

>Smaco|GQ351275

---MHLFTEGI--SVDIMTTM--QRT----HT----NA-ERWFKIFR--------DDIHK

WVI----GLEE---G--KGEYGHWQVRCNVE---YLRAVFGWLG---PI---SIWTEECS

-----DKYTYE---------TK------E-----G-KYWAS-WDT-MGA-RQQ--RGKM-

--R--N-QGAV----------------Q----------------ALQR------------

---------------------------TN-----DREIVVWYDEGNMGKSWLCGHLFETG

-------AYYIP---PYMSTVAS----LVLQR-PLIVIDIPRSL----YTAIEAIKGGLI

MD----PRYGARPVN-I---HGTKVIVLTNTKP----K------------LDKLED----

--RWV-L--YDDY-----------------------------------------------

----------------------------

>Smaco|KU043403

M------------TKTVMITM--ERR----ET----YK-EWFLFAIR--------KDIHK

WII----AAEE---G--KGGYKHWQIRIAS-DAKWTRALLIQFG------QRSIHTENAS

-----NTWTYE---------AK------E-----G-CYLAS-WDT-LEV-RQQ--RGKL-

--R--V-QYAL----------------N----------------VLEG------------

---------------------------TN-----DRQVMVWVDEENSGKSWLIGHLYETG

-------AYYAP---PYLSTIAS----LAVKK-KYALIDIPRSL----YTAIEAIKDGLI

ME----PRYSAQPIN-I---KGIKVLVVTNTRP----K------------LDKLKD----

--RWE-I--FENN-----------------------------------------------

---------------------GQEKRTL

>Smaco|YP_009118278.1

----------M--TNVYMITM--PRK----G-----YS-GAIMRFFR--------KDVHK

WIV----SPEK---G--AQGYEHWQIRFRCTPENAMIAWRQWVC---NG---FNMLEASD

-----NGWEYE---------GK------E-----G-KFLAS-WDS-KGA-RSV--RGKM-

--E--R-QATV----------------L----------------RARA------------

---------------------------TN-----DREIVVWYDPKNSGKSWLVGHLVETR

-------AYYVP---PYLTTLAS----MVKAR-PLVVIDIPRSM----YVAIEAIKDGII

VD----PRYSATVEN-V---KGIGVIVITNEKP----Q------------VGKLAD----

--RWD-I--VD-------------------------------------------------

----------------------------

>Smaco|KU043422

---------MT--NKIYMITM--PKN----------AR-NLIKNLIE--------TDLHK

WTY----GYEI---G--KNGYKHIQARIRC----DFDYLQEYF--------GQAHIEEAS

-----DEWDYE---------TK------G-----G-IYFTS-EDW-GER-LKQ--RLPL-

--K--V-QKAL----------------E----------------GLEA------------

---------------------------TN-----DREVYVWYDEKNAGKSWLCGHLWETG

-------AYVVD---S--KDVAN----EYINR-PYVIIDLPRTL----YYAIEKIKDGLL

KD----PRYQSKTVN-I---HGVKVMVMCNHRP----N------------VSKLAD----

--RWK-M--FTAA-----------------------------------------------

----------------------------

>Smaco|KU043420

--------------MSRLLIR--TRK----SV-LIKRC-RIKSSFKN--------LDCKR

WTL----GIET---G--KGGYKHCQWRVECGDDQFFEHFSEFTG---WGIEKKSHIEKS-

-----DKWDYE---------TK------E-----G-HYVKS-DDR-VEN-IIQ--RGKY-

--R--A-QRVI----------------K----------------ALRR------------

---------------------------TN-----DREVVLWYDKVNCGKSWFTGALWERG

-------AYVTA---E--KDIAS----DFLDR-PFVIIDLPRTL----YLAIERIKDGLI

KD----PRYNSRTVN-I---RGVKVLVCCNSLP----K------------LDKLKD----

--RWV-R--LEGT-----------------------------------------------

---------------------SG-----

>Smaco|YP_009252308.1

-----------------MLTM--PWT----GQ----SK-RMINLYIT--------RDIHE

WII----GYEV---G--RDGYRHIHVRFNG----DFGDVQRAF--------PGAHIEEGT

--T--METEYE---------KK------D-----G-HFVSY-EDS-PDV-LRC--RGKL-

--R--H-QRVV----------------K----------------LLEK------------

---------------------------QS-----DRGILCWYDETSIGKSFTCRHLVERR

-------AYYVP---PTVNWVCS----GYQRQ-RYLIIDIPRSL----YTGLEAIKDGLI

YD----TRYSAKLRD-I---WGVKILVLTNSLP----N------------LDALQD----

--RWM-I--INGK-----------------------------------------------

---------------------AIESKK-

>Smaco|YP_009252310.1

-------------MKKYVLTV--PRE---------ADL-RPICRML----------DAKK

WTI----GFEV---G--EHGFRHYQIRLVSSDHDFFEWCKAFL--------PTAHIEEAT

-----EERDYE---------RK------S-----G-NFLCS-DDT-DQI-RQI--RGDL-

--R--I-QKIL----------------K----------------LADD------------

---------------------------QN-----DRQISYFYDPDGAGKSWLTIHLWERG

-------CFVVP---RSKTFICS----GYKAE-EYIVIDLPRAL----YELLEDTKNRLI

FY----SRYQPITRN-I---RCPNLIVFSNHKL----D------------TKRLAD----

--RCQ-YYDLSDP-----------------------------------------------

----------------------------

>Smaco|YP_009252314.1

-------------MGKYSVTI--SAD----LW----KE-RDVVRLLD--------MDLRE

YYI----GREI---G--KGGYHHYQCAIDC----LERFNGQH--------QLGWHIEDCS

----WDKLRYC---------RK------G-----G-DYRYI-GDS-IEE-QSY--R--SR

--T--V-GTID----------------T----------------HLKK------------

---------------------------QN-----DRQISICVDTKGSGKTTHGYDRSRTG

-------YFVVP---RNATYVAM----NYDNE-PVIWIDLPRTL----ATILEDMKDGLI

YS----AKYEGQVRH-I---KGVKVLVTTNHKP----A------------YKLLAD----

--RWD-V--FTPP-----------------------------------------------

---------------------SD-----

>Smaco|AEW47007.1

--------------MKYVATI--SRT----SI----PE-HHLVRLLK--------LDLHE

AYI----GRET---G--ARGFEHYQCCIDC----LVRFNTEH--------QLGWHIEECS

----WEASNYC---------RK------T-----D-NYRYV-GDS-IEE-REY--SIATR

--N--V-ARIQ----------------F----------------HIDH------------

---------------------------RN-----DRAISICVDTIGTGKSTYGYLCARRG

-------FFVVP---RTATYIAM----HYDNQ-PVIWIDLPRSL----AECLEDIKDGLV

AS----AKYEGCLRF-I---RGVKVLVTTNHKT----T------------YKMLAD----

--RWD-I--FTPS-----------------------------------------------

---------------------S------

>Smaco|KM598409

MKKEWAKEEEC--NVQYIGTI--SAE----DW----NE-DGIIRVFE--------NDGHE

LYI----GREI---G--KHGFRHYQFCMDC----LEKYTADN--------RTGWHVERCS

----WEMSGYC---------RK------T-----G-DYRYI-GDS-REE-RYY--ALRAR

--L--I-WSFG----------------A----------------SVVK------------

---------------------------QN-----DRSITVWVDTEKAGKSTFSYILERRG

-------CLNIP---RTENFVAM----HYKGE-PLIIVDIPRDL----CRALETIKDGVI

TS----AKYQGTKMF-I---KGVKILVFTNHKT----T------------YAALED----

--RWD-V--KSLK-----------------------------------------------

---------------------P------

>Smaco|YP_009252316.1

-----MGFITE--DAFF--------P----EF----LE-KTLQEALD--------KICER

YAY----GNEV---G--KDGYEHFQCRIVCSKPTDERALRVFL-------LSNGHTSPTQ

--V--RNFEYV---------QK------E-----G-NLYCS-WEA-V-L-KQF--PGTP-

--Y--W-QIAF----------------S----------------MWKK------------

---------------------------QN-----DREILVITDDKRHGKSWLRKYMVACH

-------GTFIP---PL-KCAMA----K-PS----YIIDMPRAM----WSAIEQMKDGYL

YD----KRYSWQEKW-I---EPPKIMVFCNDFD----P------------L-MLTD----

--RWQ-SFDIT-------------------------------------------------

---------------------PE-----

>Smaco|AJF23062.1

--------MPS--TKWYDITA--SKE----KL----PR-SILEKWLN--------ERCER

WAY----GNEV---G--EGGYEHYQIRIVLKEPTDEYEMRKMW-------AAFGHVSPTH

--V--RNFDYV---------LK------D-----G-DFVCS-WIK-Y-P-ESM--VPTF-

--R--W-QCLL----------------E----------------L--E------------

---------------------------QS-----DREIDVIIDLKNTGKSWITKYLALKN

-------ATNVP---SG-EMCLK----R-PT---WYIFDMPRAT----WSAIESIKNGYL

WD----DRYTWEEKW-I---EPPKVTVFTNEYP----K------------YDLVED----

--RLR-MWA---------------------------------------------------

---------------------PETGLVA

>Smaco|AJF23060.1

--------MPS--PKWYDITA--SKE----KL----PR-SILEKWLN--------ERCER

WAY----GNEV---G--EGGYEHYQIRVVLKEPTDEYEMRKMW-------AAFGHVSPTH

--V--RNFDYV---------LK------E-----G-DYVCS-WIK-I-P-ENM--QATF-

--R--W-QALL----------------D----------------L--E------------

---------------------------QD-----DRQIDVIIDLANTGKSWLTKYCALKM

-------AINVP---SG-EMCLK----RGAQ---LYIFDMPRAV----WSAIESIKNGYL

WD----DRYTWEEKW-I---DPPRIWVFTNEYP----K------------YDLVED----

--RLR-FWA---------------------------------------------------

---------------------PPTGLVS

>Smaco|AJE25847.1

--------MPS--PKWYDITV--SAD----KV----PE-KTMVEWLN--------KYCER

YAY----GRET---G--ENGYKHYQIRLVLKVGADISEMRKVW-------SAFGHVSCTS

--V--RNFDYV---------LK------E-----G-DYVCS-WIK-I-P-ESM--VPEF-

--R--W-QALL----------------D----------------L--E------------

---------------------------QD-----DRQIDVIIDLQNSGKSWLTKYCALKM

-------AINVP---SG-EMCLK----RGAQ---LYIFDMPRAV----WSAIESIKNGYL

WD----DRYTWEEKW-I---DPPRIWVFTNEYP----K------------YDLVED----

--RLR-FWA---------------------------------------------------

---------------------PPTGLVS

>Smaco|KP233175

--------MPS--PKWYDITV--SAD----KV----PE-KTMVEWLN--------KYCER

YAY----GRET---G--ENGYKHYQIRLVLKVGADISEMRKVW-------SAFGHVSCTS

--V--RNFDYV---------LK------E-----G-DYVCS-WIK-I-P-ENM--KPEF-

--R--W-QALL----------------D----------------L--E------------

---------------------------QD-----DRQIDVIIDLQNSGKSWLTKYCALKM

-------AINVP---SG-EMCLK----RGAQ---LYIFDMPRAV----WSAIESIKNGYL

WD----DRYTWEEKW-I---DPPRIWVFTNEYP----K------------YDLVED----

--RLR-FWA---------------------------------------------------

---------------------PPTGLVS

>Smaco|AJE25851.1

--------MPS--PKWYDITV--SAD----KV----PE-KTMVEWLE--------KYCER

YAY----GRET---G--ENGYKHYQVRLVLKVGADISEMRKVW-------SAFGHVSCTS

--V--RNFDYV---------LK------E-----G-DFVCS-WIK-V-P-DAI--RAQL-

--K--W-QHLV----------------D----------------L--K------------

---------------------------QN-----DREVDCIIDVRNTGKSFVTKYMCMKN

-------AINIP---SG-TMCMK----R-AK---TYIFDIPRAT----WSAIESIKNGYM

WD----DRYTWEEMW-I---DSPRVFVFTNEYP----K------------YDLLED----

--RFR-FWA---------------------------------------------------

---------------------PELGLVQ

>Smaco|AJE25845.1

--------MTE--PKWYDITV--SKA----KC----PE-EILRKWLD--------ENGER

YAY----GRER---G--EDGYEHFQVRVVLRNPTSWETMREIW-------GNSGHCSPTS

--I--RNFDYV---------LK------E-----G-DFVCS-WIK-V-P-DAI--RAQL-

--R--W-QHLV----------------N----------------L--K------------

---------------------------QN-----DREVDCIIDLRNTGKSFVTKYMCMKN

-------AINIP---SG-TMCMK----R-AK---TYIFDIPRAT----WSAIESIKNGYM

WD----DRYTWEEMW-I---DSPRVFVFTNEYP----K------------YDLLED----

--RFR-FWA---------------------------------------------------

---------------------PELGLVQ

>Smaco|KY086301

--------MTS--PRWYDITV--SKA----KC----PE-EILRKWLD--------ENGER

YAY----GREK---G--TDGYEHFQVRVVLKNPTSEQDMWKVW-------APYGHVSPTH

--T--RNFNYV---------LK------E-----G-DFVCS-WIK-I-P-ESM--KPVF-

--R--W-QALM----------------D----------------L--E------------

---------------------------QN-----DREVDVIIDLKNTGKSWITRYLALKN

-------AINIP---SG-GMCLK----R-AV---WYIFDMPRAL----WSAIESIKNGYL

WD----DRYTWEEKW-I---DPPKVTVFTNEYP----K------------YDLIED----

--RLR-FWA---------------------------------------------------

---------------------PEVGLVA

>Smaco|YP_009252326.1

--------MSD--PKWYDVTA--PQA----VL----SA-ENVETILE--------QECER

YAY----ADEV---G--EGGYKHWQIRYVLRKGSPVEEQIMIW-------SMWKHVSPTH

--V--RNFNYI---------MK------T-----D-NYFCS-WES-E-L-REY--RLEY-

--R--W-QYTL----------------E----------------MIEQ------------

---------------------------QN-----DREIFVIQDPLGIGKTTFAKHLVANH

-------AIYIP---PL-EMAMA----K-ARR-ETFIIDVPRAM----WSAVEQIKNGFL

YD----KRYQWSEKW-I---KSPKIVVLTNELP----K-------------DKLND----

--RWN-VYQ---------------------------------------------------

---------------------PNFEY--

>Smaco|AJD07511.1

---RRYRSMTE--PKWWDCTL--SRE----ET--WHAP-DVGKQLME--------HGAER

CVV----GEEV---G--EDGYQHFQIRVVFKKPTSFEKATAIL-------PG--HWSETS

--QFGRNFDYV---------EK------E-----G-HFWRS-WET-A-L-GRF--MLDM-

--Y--W-QEIM----------------T----------------RLEK------------

---------------------------QN-----DRKVMVIVDRYNSGKTAIAMRLTAEH

-------GAYCP---EL-DWALA----H-KN---CFCLDIPRAI----WKAVEQMKNGYL

WD----KRHHWQEAF-I---MPSKGLVLTNDEP----D------------RNLLRD----

--RWD-IGHL--------------------------------------------------

----------------------DYGISN

>Smaco|KU203352

--------MSR--CKWIDATADANEN----VM----KL-EDWKNGLS--------GYFER

YAF----GCEV---AP-ETQRRHYQFRGVLKADLSNDIALALS-------DLGLHITPTH

--V--RDFEYV---------YK------D-----R-NFYCS-WEV-F-R-PEY--EVQN-

--SHVW-QQLE----------------E----------------L--D------------

---------------------------RD-----ERSIEIIWDEKNSGKTAWAMYQDYSH

-------AVYIP---PL-RCVLG----K-RE---WYIIDTPRAD----WASIEQLKNGYV

FD----TRYSFRDKY-L---SRPRVTILCNHMP----D------------YEYFAD----

--RVL-PFRIT-------------------------------------------------

---------------------PGYLWSV

>Smaco|KJ547633

--------MAN--VKWIDATIDNFEG----VK----DE-SEWRSRFA--------GLFER

YAY----GRET---AP-ETGRRHFQFRGVLKVACDASCLAYLS-------SLGFNISPTH

--V--RDFDYV---------YK------D-----R-DFFCS-WDV-Y-R-PEY--DVRN-

--SHVW-QQLE----------------D----------------M--E------------

---------------------------RD-----DRTIEIVWDERNSGKTAWAMYQDYLH

-------AVYIP---PP-RCVLG----K-RE---WYIIDTPRAD----WASIEQLKNGYV

FD----TRYSFRDRY-L---SRPRVTVLCNTLP----E------------YEYFAD----

--RVL-PFRIT-------------------------------------------------

---------------------PGYLWSV

>Smaco|AIY31243.1

--------MTN--PTWFCGTI--WKH----LT--W-TM-EQLAEWFK--------QHAKE

GVI----GLEI---SP-TSGKEHYQFKIHLDRGETLEGWKALI-------GPMGHIEIAV

--D--KNFGYE---------EK------D-----G-NFIKW-PTS-P-L-EKY--KLKL-

--R--W-QDVV----------------E----------------TFER------------

---------------------------QD-----DRRILVVVDKQGNGKSYLSRFMEATG

-------ADVCP---VISEYCLD----N-PC----YVFDLPRAM----WMGIEQIKNGLL

YE----KRYRPRKMW-I---EPPKVLVFTNDDV----P------------WDMLRD----

--RWE-AYELYQL-----------------------------------------------

---------------------PDTE---

>Smaco|KT862224

-------------------MV--RSH----NMDHG-TT-QRLA---Y--------QHAKE

GVI----GEEI---SP-TSGKTHYQCKWHLSRGESIDGWKLLI-------GPMGHVDIAV

--E--KRFGYE---------EK------D-----G-KFVKW-PES-P-I-AKH--KLAL-

--K--W-EALL----------------D----------------SIKN------------

---------------------------QD-----DRHITVVVDKQGNGKSTFSKYLEAND

-------ADVCP---VVSEYCME----F-PK----YVFDLPRAM----WSGIEQIKNGLL

YE----KRYKPRKMW-I---EPPSILVFTNDDI----P------------WELLED----

--RWD-AYRLHTL-----------------------------------------------

---------------------PDDYING

>Smaco|AIY31256.1

--------MTN--PTWFCGTI--WKD----KY--G-SK-EQLAEWFR--------LHAKE

GVI----GEEI---SP-TSGKIHYQFRIHLDRGETLKGWQELI-------KAVGHVEVCQ

--E--KKFDYE---------KK------D-----G-NWIEW-PES-Y-L-GKF--KMPL-

--L--W-QQLL----------------E----------------QWNE------------

---------------------------QD-----DRQILFVKDVKGNGKSTFGKIMEARG

-------EEVCP---VCSDFCLE----Y-PA----YIFDIPRAL----WSGIEQIKNGLL

YE----KRYKPRKVW-I---EPPKVIVFTNDEP----P------------WDLLLD----

--RWR-VFDV-EQ-----------------------------------------------

---------------------PTYNIR-

>pCRESS6|WP_034704841.1

-----MKKQAS--LTCVMIVQ--QLKEEFWTINNIQPLMEELNKRF-----HNRGYEVNE

LYG-IIHDKDERLVIEPK--EKHAHILVKFAKGDTLNNLSVTAGVD-----PQ-YIEKAK

SGGYDNLLSYLVHAKD--QD-KYQYSPDEVVTVSGEEYTSVY-N--RRM-ETWV-RGRAT

KADLS-VDYLVSE-ILDGKL-TKSQVLL---T-NEYKVYALHKRKIND-AFDTA------

-----------G-ENKSYQTIADLDAGNF-----KKTILFIMAESGAGKTVLSKKIISI-

LQSVALKDYCLT---ASNN-AFD----EYNGQ-DVLFLDDIRG-DSLSVSDWLKLLDPYT

---ISPISARYHNKM-G---SAKVIIITSTKTPSEFFSIAK---SNFHEDLGQFFR----

--RIDLLISI-EDDK------FHLSKFEKAPPAPPSH--YFRFDGTY-YKNQALDKVTKV

TIRNMQ--W----NKKKSVTK-------

>pCRESS6|WP_067483596.1

-----MKKQAN--LSCVMLVQ--QLEPEFWVINNIRPLIEELVKRF-----EKDGCEVNE

AYG-ILHDKDLISVEELK--AKHVHILIKFGKGDTLNSLAVKAGVA-----PQ-YLEKAK

SGGYDNLLSYLVHAKD--QD-KHQYFPDEVVTVSGEEYTSVY-N--RRM-ETWV-RGRAT

KADLS-VDYLVSE-ILAGKL-TKSQVLL---T-NEYRVYALHKRKIND-AFDTA------

-----------G-ESKSYQTIADLDAGNF-----KKTTLFIMAESGAGKTVLSKKIISI-

LQSVALKDYCLT---ASNN-AFD----EYNGQ-DIIFLDDIRG-DSLSVSDWLKLLDPYT

---ISPISARYHNKM-G---SAKAIIITSTKTPSEFFSIAK---GNFHEDLGQFFR----

--RIDLLINI-EDDK------FHLSKPEKAPPAPPSH--YFHFDGTY-HKNKALDMVTKT

VIRNMQ--L----NKKKPVTQ-------

>pCRESS6|WP_049499636.1

M----RKKEAN--LTTIMLVQ--QLEEKYWMPNDCLPLLKMIVNNL-----ESNNIIVKE

AYI-IKHDKDKISTVQDK--AVHIHALLKFERGASLSKIALAIRVE-----PQ-YLEKMK

SGGYDNCLAYLVHAKD--ES-KHQYQPDEVVSLRGEDYTSIY-Q--RSI-EMWV-KGRAT

KENFS-IDWLIEQ-VLDGKL-TKSNIML---T-DEYEIYGQHKRKVNE-ALDTA------

-----------G-ERRSYRTIAELEAALF-----KKTVIFIQANSGVGKTKFSKELISA-

IQAIAVKETCVT---ASTN-AFD----EYNGQ-EILFLDDIKG-DSFTVSDWLKLLDPYT

---ISPISARYHNKM-G---SARVIIITNTKLPVELFYFAK---NNYNEDLGQFIR----

--RIDLLVHI-HDD-------IFHVCPHEKREERHSY--TFQKSHAI-QRNEALEEILET

VMTNMN--W----NKAKKVIT-DNLKTQ

>pCRESS6|WP_045759092.1

M----GKKEAN--LTAIMLVQ--QLEEKYWGPNDCRPLLEMIIKKL-----KSSDIIVKE

AYI-IRHDKDKISTVEDK--AVHVHALLKFEKGASLNKIALAVEVE-----PQ-YLEKLK

SGGYDNCLAYLVHAKD--ET-KYQYRPEEVTTVLGEDYTSIY-N--RSI-ETWL-KGRAT

KENLS-VDWLVER-ILDGEV-TKRNIML---T-DEYAIYGQHKRKVNE-ALETA------

-----------G-ERRSYRTIAELEAGKF-----KKTVIFIQASSGVGKTKLSKDLIEL-

IQASAVKEACMT---ASTN-AFD----EYSGQ-EILFLDDIKG-NSFTVSDWLKLLDPYT

---ISPISARYHNKM-G---SARVIIITSTKLPVELFYFAN---GNHNEDLGQFVR----

--RIDLLVHI-HND-------IFHVCPHEKTAEHHSY--TFQKSPAI-QRDEALEEILET

VTTNMN--W----NKTKKVID-DNPNNQ

>pCRESS6|KXT86702.1

MN--KNKKEAN--LTAIMVVQ--QLEEEHWTPENCRPLLEMIIEKL-----EEQDILVKE

AYI-IKHDKDKVSTIETK--VEHVHVLLKFEKGASLNKLALAIQVE-----PQ-YLEKLK

SGGYDNCLAYLCHCKD--EN-KHQYQPEEVTTVRGEDYTSIY-H--RSM-ETWT-KGRAT

KEALS-VDWLIEK-ILSGEV-TKSNILL---T-NEYAIYGQHKRKINE-ALDTA------

-----------G-ERKSYQTIADLEAGNF-----KKTILFITAESGVGKTRYSKKLITL-

LQQIALKDYCVT---ASTN-AFD----EYNGQ-EILFLDDIKG-DSLTVSDWLKLLDPHM

---ISPISARYHNKM-G---SAKIIIITNTKEPISFFEQAK---GNMGEDLGQFVR----

--RIDYLIQI-EDK-------FHLSIPIKHIPQHYSF--KFSKIGEY-DKNIATNKLVKQ

VIRNMQ--W----NKYKKVIN-DNPNTQ

>pCRESS6|WP_014623544.1

M----AKKEAT--LTAIMVVQ--QLEDEFWEPDDCRPLLELIIKNL-----ESNDIIVKE

GYI-IKHDKDKVSVIENK--TEHIHALLKFEKGASLTKIALAIGVE-----PQ-FLEKLK

SGGYDNCLAYLVHAKD--ES-KHQYQPDEVVTVKGEDYTSVY-H--RSM-ETWV-KGRAT

KENLS-VDWLIEK-ILSGEI-TKSNIML---T-DDYAIYGQHKRKINE-ALDTA------

-----------G-ERKSYRTVAELESGKF-----KKTVLFITAESGVGKTHYSKQLIAL-

LQNIALKDYCVT---ASTN-AFD----EYNGQ-EILFLDDVKG-NSLTVSDWLKLLDPYM

---ISPISARYHNKM-G---SAKVIIITNTKEPIRFFEQAK---GNIEEDLGQFIR----

--RTDYLIEI-SDS-------FKLYVPIKHTPSHYSY--KYSKTNEL-PINLATDKLIKK

VIQNMQ--W----SKQKKVIN-DNPNNQ

>pCRESS6|WP_032497992.1

-----MVQHTN--LTAIMLVQ--QLEKEYWHSSNCRPLLDLIIEKL-----EKQGILVKE

AYI-IKHDKDKIVTSTNK--AEHVHALLKFEKGASLNKLALAIQVE-----PQ-YLEKLK

SGGYDNCLAYLCHIKS--EN-KHTYKPEEVITARGEDYTSIY-H--RRM-EIWV-RGRAT

KELLS-VDWLVEK-ILSGKL-TKNNILL---T-DEFSIYGQHKRRINE-ALETI------

-----------G-ERKSIQTIAELEAGKF-----KKTIIFITADSGMGKTQYSKKLITI-

LRNIALKECCVT---ASTN-AFD----EYNGQ-EILFLDDIRG-ESLTVSDWLKLLDPYM

---VSPISARYHNKM-G---AAKVIIITTTNDPLSFFRKAK---GSFGEDLGQFVR----

--RIDYLIQI-TDM-------FHVSASIKNTPLHYSF--KFSKIGAY-EKNKATDKIVKQ

VIRNMQ--W----NKTKKVID-DNSLL-

>pCRESS6|KEQ49321.1

MN--KNKKEAN--LTAIMLVQ--QLENEHWTAENIRPLLETVTDKL-----NKADITVKE

AYG-IKHDKDEINVIEKK--AEHIHFLFKFEKGASLNRIALAVGVE-----PQ-YLEKLK

SGGYDNCLAYLVHAKD--ET-KYQYQPEEVATVLGEDYKSIY-H--RNM-ATWV-KGRAT

KESLS-VDWLIEK-ILVGEV-TKRNIML---T-NEYAIYGQHKRKINE-ALDTA------

-----------G-ERKSYQTIDDLESGEF-----KKTILFIKAESGVGKTLYSKKLITL-

VQNIALRDYCIT---ASTN-AFD----EYNGQ-EILFLDDIRG-DSLNVSDWLKLLDPYT

---ISPVSARYHNKM-G---AAKLIIITSTKEPLSLFKQSK---GNTGEDLGQFVR----

--RIDYLVEI-TDT-------FRLAVPIKNLPTHHSF--IFSKFSCN-SINEATNKIVKR

VIQNMQ--W----NKKKKVIN-DNPGTK

>pCRESS6|WP_053092713.1

-----------------MLVQ--QLEDEHWTAKNIRPLLETVTDKL-----NRADITVKE

AYG-IKHNKDEVNVIEKK--AEHIHFLFKFEKGASLNRIALAIGVE-----PQ-YLEKLK

SGGYDNCLAYLVHAKD--ET-KHQYQPEDVTTVLGEDYKSIY-H--RSM-ATWV-KGRAT

KENLS-VDWLVEK-ILAGDI-TKENIML---T-NEYAVYGRHKQKVNE-AIETA------

-----------G-EQKSYQTIAELKEGKF-----KKTILFISAPSSAGKTRFAKELIDI-

IQKVALKDYSLT---ASTN-AFD----DYNGQ-EILFLDDMRG-SSMTASDWLKLLDPYM

---ISPISARYHNKI-G---SAKVIIITSTKKPIPFFEIAK---ENDNEDSGQFVR----

--RIDYLITI-DKS-------YNLSQPQHTTPQFHSF--KFAKSKSY-TKNKTIDILVKT

VIKNMQ--W----NKHKKVIN-DNPNNK

>pCRESS6|WP_027972054.1

M----VKKDAN--LTAIMLVQ--QLEEEYWEPENCRPLLELIIKKL-----RENDIIAKE

AYI-ILHNKDTVTIVKNK--EEHVHALLKFEKGASLNKIAFAVGIE-----PQ-YLERLK

SGGYPNCLAYLVHAKD--EN-KHQYQPEEVVTVLGEDYVSIY-H--RSM-ETWI-KGRAT

KEDLS-IDWLIDK-ILAGEV-TKSNIML---T-DSYAIYGQHKRKINE-ALDTA------

-----------G-ERRSYQTIAEMEAGKF-----KKTIIFIQAESGAGKTRLSKKFIAL-

LQKVALKDFCVT---ASTN-ALD----EVNGQ-EILLLDDLRG-SSLTVSDWLKLLDPYM

---ISPISARYHNKI-G---SSKVIIITSTKKPIDFFEVAK---DNVGEDLGQFIR----

--RIDYLLEL-GDK-------VALSVPEKQTKHFASY--DFSQKKLY-SINEAIDILVKT

TIRNMQ--W----NTQKKVIN-DNPNTQ

>pCRESS6|WP_049476139.1

M----VKKEAN--LTAIMLVQ--QLEDTYWTFDDCKPLLQAVVDKL-----NDDNISVKE

AYG-IKHDKDEITITEKK--AEHVHFLFKFDKGASISKIALAVGVE-----PQ-YLEKLK

SGGYDNCLAYLVHAKD--ET-KFQYSPEKVVTLLGEDYVSIY-N--RSM-ETWM-KGRAT

RESLS-LDWLIEK-ILSGEI-TKSNIML---T-DEYAIYGQHKRKINE-AIETS------

-----------G-ERKSYKTISELENSEF-----KKTVIFITAESGIGKTALSKQLIRI-

LQTVAIKDFCVT---ASTN-AFD----EYNAQ-DILFLDDIRG-DSLTVSDWLKLLDPYM

---ISPISARYHNKM-G---AAKVIIITSTKKPISFFESAK---GNIGEDLGQFIR----

--RIDYLLTI-DKF-------FSLSIPMKSKVMHHSY--CFSESEQY-TKNNALNHLVKT

VIRNMQ--W----NK-KERIT-DTL-TQ

>pCRESS6|WP_003035134.1

M----VKKEAN--LTAIMLVQ--QLEDTYWTPDNCKPLLQVVVDKL-----NNENISVKE

AYG-IKHDKDEITVTEKK--AEHIHFLFKFEKGASLSKIALAIGIE-----PQ-YLEKLK

SGGYDNCLAYLVHAKD--ES-KFQYSPDEVVTLLGEDYLSVY-N--RSM-ETWM-KGRAT

KEALS-VDWLIEK-ILSGEI-TKSNIML---T-DEYAIYGQHKRKINE-ALDTS------

-----------S-ERKSYKTISELENGEF-----KKTVIFITADSGIGKTTISKQLIRI-

LQKVSLKDTCVT---ASTN-AFD----EYNGQ-DILFLDDIRG-DSLTVSDWLKLLDPYM

---ISPISARYHNKM-G---SAKVIIITSTKKPVSFFEASK---GNIGEDLGQFIR----

--RIDYLLTI-NDS-------FWLSLPIKSKKTYHSY--CFSEVEQY-TKNNALDHLVKT

VIRNMQ--W----NTTKKVIN-DNQNIQ

>pCRESS6|WP_017649267.1

-----MAKSSN--LTAIMLVQ--QLEEKYWTADNCQSLLEAVVKHL-----EVENIKVDE

AYG-IIHNKDTVTVDETK--EDHIHFLFKFAKGASLEKLALSIGVE-----PQ-YLEKLK

SGGYDNCQAYLVHAKD--ES-KYQYSADEVVTVLGEDYISLY-N--RKM-ESWI-RGRAK

QDNLS-IDWIISE-ILAGKL-SKNQILL---T-DDYKVYGQHKRKINE-AIDTA------

-----------G-ERKSYKTISELEAGQF-----KKTIIFINAESGVGKTAISKKLIGI-

LQTVALKDFCVT---ASTN-AFD----EYNGQ-DILFLDDIKG-DSLTVSDWLKLLDPYM

---ISPISARYHNKM-G---SAKVIIITNTKEPMHFFEQAK---GNIGEDLGQFVR----

--RIDYLLTI-DET-------FNLSTPKKLNQLIYSY--SFSEPNQY-SKNEALDLLVKT

VIRNMQ--W----NTTKKVIN-DNQNIQ

>pCRESS6|ABJ73998.1

-----MVDKTN--LKAIMLVQ--QLEDKHWTAENCRPLLEEVVTRL-----SSENIKVEE

AYG-IIHNKDTISVDEPK--ANHVHFLFKFDKGASLQKLALAMGIE-----SQ-YLEKLK

SGGYDNCQAYLVHAKD--DS-KYQYSANEVTTILGEDYVSLY-N--RKM-KSWI-RGRAK

QENLS-VDWIIAE-VLAGQL-TKNQILL---T-DEYKVYGQHKRKINE-ALDTA------

-----------G-ERKSYKTVAELEAGKF-----KKTVIFVKADSGIGKTALSKKLIGL-

LQMVAIKDFCVT---ASTN-AFD----EYNGQ-DILFLDDIRG-DSLTVSDWLKLLDPYM

---ISPISARYHNKL-G---SAKVIIITSTKHPFKFFENAK---GIVGEDLGQFIR----

--RIDYLLTI-DGS-------FNLSTPQKLNNSTTSH--SFSQPNQQ-SRNAVLDLLIKT

VIRNMQ--W----KKQQKGII-ENSTKK

>pCRESS6|WP_015647385.1

M----IKKEAK--LTTIMLVQ--QLENEYWKPSNCRPLLEMIVEKL-----EKHGITVKD

AYI-IKHDKDKVSIFENK--EDHVHALFKFEKGASLNKLALAIQVE-----PQ-YLEKLK

SGGYDNCLAYLCHSKQ--EN-KHQYQPEEVITVRGEEYISIY-H--RSI-ETWI-KGRAT

KENLS-VDWLIEK-ILAGDL-TKSNIML---T-DEYSIYGQHKRKINE-ALETA------

-----------G-ERRSYRAIAELEAGKF-----KKTVLFINAESGVGKTQFSKNFIRL-

LKNVARNDSCVT---ASTN-PFD----EYNGQ-EILFLDDIKG-DSLTVSDWLKLLDPYM

---ISPISARYHNKM-G---SAKLIIITNTKEPLSFFEQAK---GNIGEDLGQFVR----

--RIDCLLTI-DKN-------FNISLPEKTVNPISSY--NFANPIAL-SSNETLDYLIKT

IIQNMQ--W----NK-KKAIS-DNPNIQ

>pCRESS6|CGE81062.1

MV---IKKEAR--LTAIMLVQ--QLKKDYWFPKNCLPLLEKIVEKL-----EQQDIIVKE

AYI-IKHDKDETSVLEKK--EEHVHVLLKFENGASLNKIALATKVK-----SQ-YFEKLK

SGGYDNCLAYLVHAKD--ET-KHQYSPEEVFTVRGEDYKNIY-H--RSM-ETWI-RGRAT

RDKLS-VDWLIEQ-ILTGKI-NKRTIML---S-NEYTIYGQHKRKINE-ALDTA------

-----------G-EHKSYRTVAELEAGKF-----KKTILYITAESGAGKTQFAKRLIKL-

FQKIALNDYCVT---ASTN-AFD----EYNGQ-EILFLDDIKG-DSLSISDWLKLLDPYM

---ISPISARYHNKM-G---SAKIIIITNTKEPISFFEHSK---GSVGEDLGQFVR----

--RIDYLIEI-GNN-------FHLSIPIKQKSKQYSY--EFSKVGEY-SITNATNEIVKK

VIQNLQ--W----KESKEDVF-NNP---

>pCRESS6|WP_039677656.1

M----PKKEST--LGAIMLVQ--QLENKYWTADNCRPLLEIIIKKL-----EDNGIILKE

AYI-IKHDKDEISIIKNK--AEHVHILLKFEKGASLNKIALAIEVE-----PQ-YLEKLK

SGGYDNCLAYLVHAKD--ES-KYQYQPEEVTTLLGEDYVSLY-H--RNM-KTWV-KGRAT

KENLS-IDWLIEK-ILAGEV-TKSNIML---T-DDYTIYGQHKRKINE-ALDTA------

-----------G-ERKSFQAIEDIDSGKF-----KKTIIFLQGESGQGKTKLSKSIINI-

AQRIAFNDSCST---ASTN-AFD----EYNGQ-DVLFLDDMRG-DSLTVSDWLKLLDPYT

---ISPISARYHNKM-R---AAKLIIITSTKAPLEFFSLAK---GNFGEDLGQFVR----

--RIDLLAEV-GDN-------IKLSKPVKLESMSHSF--NFQNFEVF-NRNMAIDYIIKT

IIRNMQ--W----NK-KERIT-DTL-TQ

>pCRESS6|WP_044774450.1

M----VKKEAN--LTAIMLVQ--QLEEEYWEPENCRPLLELIIKKL-----RENDVIAKE

AYI-IKHDKDTVTIIKNK--EEHVHALLKFEKGASLKKIALAISVE-----PQ-YLEKLK

SGGYDNCLAYLVHAKD--ET-KHQYQPEEVITIKGEDYTSIY-H--RSM-ETWT-KGRAI

KEDLS-VDWLIEK-ILAGEI-SKSNILL---T-DEYAIYGQHKRRINE-ALDTA------

-----------G-ERKSYEAIADLEAGKY-----KKSAIYVLADSGVGKTKFCMELIHR-

LQNIAKESYCLT---ASRN-AFD----AYQGE-EILFLDDIRG-DALSVSDWLKLNDPFM

---ISPISARYHNKM-G---SAKLIIITSTLLPSVFFSQAE---GNKNEDNGQFIR----

--RFDYQVHIPSDK-------FLLSTPEKNEPIFHSY--SFSTASEL-DKDAAMERIINT

ILDNMN--L-----KNKKVIN-DNPNTQ

>pCRESS6|WP_020997784.1

M----VKKEAN--LTAIMLVQ--QLEEEYWSVDNCRPLLEIIIQKL-----EEHDIIAKE

AYI-IKHDKDKVTIINDK--EEHVHALLKFEKGASLKKIALAIGVE-----PQ-YLERLK

SGGYDNCLAYMVHAKDF-PE-KYQYSPDEVITVLGESYKSIY-Q--RKI-ETWV-RGRAT

KEDLS-VDWLIEK-ILAGEI-SKSNILL---T-DEYAIYGQHKRRINE-ALDTV------

-----------G-ERKSYQAIIDLETGNY-----KKSALFIMADSGVGKTKFSMELIHH-

LQNIAKESYCLT---ASRN-AFD----EYQGQ-DILFLDDIRG-DSLSVSDWLKLTDPFM

---ISPISARYHNKM-G---SAKLIIITSTLLPSVFFSQAV---GNKNEDNGQFIR----

--RFDYQVYIPSDK-------FLLSVPEKNEQVFHSY--SFSTATEL-DKDTAMERIINT

IFDNMN--L-----KNKKVIN-DNPNTQ

>pCRESS6|WP_003024533.1

M----SRTQAN--LTAIMLVQ--QLEEEYWTSGNSLPLLQEVVKRL-----NQADISVSE

AYG-ILHDKDTISITELK--KKHVHFLLKFEKGASLQKIALSIGVE-----PQ-YLEKLK

SGGFENCLAYLVHAKD--IE-KHQYSPDEVVTLLGENYTSIY-N--RRM-QVWL-KGRAT

RENLS-VDYLISE-ILKGNI-TKNNILL---T-NEYKVYSLHKRKFLE-AFETF------

-----------G-ERKGYQAIADLEAGKF-----KKSVFFIHAESGKGKTRLAKHLIQL-

IQSEARKEFCLT---ASTN-AFD----EYNGQ-DILFLDDIRG-DSLTLSDWLKLLDPYT

---ISPISARYHNKM-G---SAKVIIITSTRTPIEFFQLTK---GSINEDSGQFIR----

--RIDYLLKL-SDKG------YQLAIPLRKNGTIPSF--SLGKPRLY-SRGKAIYKLVKA

VSRNMQ--W----NQ-KKTVS-DSQTTQ

>pCRESS6|ADX23728.1

-----MKKQAK--LTCAMIVQ--QLEKDFWFFEDLIPLLEEIEKRL-----VSHNCIISE

LYA-IKHDKDKLVIEELK--ASHVHILIKFEKGTTLSQLAYILGIE-----AQ-YIEKAK

SGGYDNLLAYLVHSKD--KA-KFQYSPNEVITLKGEDYLSVY-N--RRK-QIWF-KGRAT

KENLS-LDYVISE-ILEGHI-TKSQVLL---T-NDYKVYALNQRKIED-AFAAY------

-----------Q-EKKGFVTIQSLENREF-----RKTIIFITGKTASGKTSLAKEIIKS-

IKDIAFREHCIT---ASTN-SFD----NYNGQ-EILFMDDVRG-YGLTATDWLKLLDPYN

---ISPISARYKNKL-G---YAKVIIIASSVEPSLFFHSAK---NYHYEDPSQFIR----

--RLDALVKI-DTT-------YQLSIPHKIQMVLSDY--YFKNIAKG-KHEKIIQKVLSM

FLKNMK--W----KDEK-----------

>pCRESS6|WP_056938517.1

-----MAKDST--LTCVMIAQ--QLQPEFWQPNDARDLLDNVVQRL-----DKSNVKVSE

AYG-IIHNKYTETIIRQK--EDHVHFLLKFDKGNTINNLAMTIGVE-----PQ-YLEKAK

SGGYDNLLAYLVHAKD--KD-KFQYNPKDVTTAVGEDYLSVY-N--RRR-ETWL-RGRAT

QNMQS-VDYLIAQ-VLQGKL-TKSQIMS---D-EDYMVYGLNSSKING-AFTVI------

-----------G-ERKSITAQRDIEASKF-----KKKIIFISGTAGVGKTKFGKLLVRQ-

IQKAVQKECCVT---ASTN-PFD----EYSGQ-EILFLDNVRG-ETLGFLDWLKLLDPHN

---ISPISARYHNKF-G---VAKVIIITSPVPPYQFFNHPK---FNSMEDLGQFYR----

--RIDFWISF-SNNK------LLVCNPIRDFWHNSSY--RFSKNGLY-KKTNAIQRILKL

INNNMK--W----KKKKEQIT-------

>pCRESS6|WP_000201649.1

MA--TKKKEAL--LTSVAITQ--YFDPKYWEL-NVEKILEEIVRRV------GEIATVSE

AYA-IKHDKDTSIGTKLK--KPHIHALLKFEKGATLTDFAVQIGLK-----PE-YLEKAK

SGGYDNLLAYLIHAKD--KD-KYQYSPDEVISLTGKDYLKVY-H--ERH-LSWL-KGKAK

KQYKD-IDLLIDN-ILNGNI-TKKEMLL---N-KDHMLYAVHKSKVNE-VFRTI------

-----------G-EIKGTMTQHELENKKF-----KKTIFFIFGLSGLGKTKFARTLTKS-

LIQLAKLQSVLT---AGTN-MFD----EVNGE-EILLLDDVRG-DSLTASDWLKLLDPYN

---ISPISARYQNRL-G---ASKVIIITSSKHPLTFFYHAK---GNTNEDLSQYIR----

--RIAHLVTL-RGNNDN--ITFHESQPKRTINRSLSY--DFTPDNEAASKEELLSMLVST

VGLYNK--W----NKIKTPSE-DEVADN

>pCRESS6|WP_047206721.1

MA--TKKKEAL--LTSITITQ--YFETKYWEL-NIEKILEEIVRRV------SEIATVSE

AYA-IKHDKDTSIGTNLK--KPHIHALLKFEKGATLTELAVKIGLE-----PQ-YLEKAK

SGGYDNLLAYLIHAKD--KD-KYQYTPDEVFTLKGKDYLEVY-H--ERH-LSWL-KGKAK

KQYKD-IDLLIDN-ILNGTI-TKKEMLL---N-KDHMLYAVHKSKVNE-AFRTI------

-----------G-EIKGTMTQNELENKKF-----KKTILFIHGVSGTGKTTLANQIVQN-

LIQLAKLQSVLT---AGTN-MFD----EVNGE-EILLLDDVRG-DSLTASDWLKLLDPYN

---ISPISARYQNKI-G---AAKVIIITSSKHPLTFFYHTK---GNNREDLSQYIR----

--RIAHLVTL-RGNSEN--ITFHESQPKRTIDRSLSY--DFTPDNEAASKEELLSMLVST

VGLYNK--W----NKIKTPSE-DEVTDN

>pCRESS6|CMU27730.1

MT--TNKKEAI--LTSVLLTQ--QFSNGFWEL-DIEKILEEIVRRV------SEVATVSE

AYA-IKHDKDTSIVTKPA--KSHIHALLKFDKGATLSTLSKKIGLA-----EQ-HLEKAK

SGGYDNLLAYLIHAKD--KD-KYQYSPDEVFTLMGNNYLKVY-H--ERK-LSWL-KGKAK

RQYED-IDLLIDN-ILNESI-TKNEILL---E-QKRTLYAVHKARIND-TFRTV------

-----------G-EIKGTRTKYELDNEEF-----KKTILFIHGSTGLGKSKFAKELTKD-

IVQLAKLQSVVT---AATN-IFD----EVNGE-EILFLDDVRG-DSLTASDWLKLLDPFN

---ISPISARYQNKM-G---AAKVIIITSSKYPLDFFYDTK---GNDREDLSQYVR----

--RIECLATI-KGNDKN--PKFYVSYPQRMEEPSLSY--DFTDDSLLNSRQDLLSTLLSK

IAINNQ--W----DKSKTPSE-DEVEDN

>pCRESS6|WP_000044268.1

S----KRKETF--LGSILVTQ--QFDIDYWET-DIKKILAEIIERV------NQVATVAE

AYA-IKHDKDFTELTKPV--EPHIHALLKFSKGATLPELAAHIGIE-----PQ-YLEKAK

SGGYDNLLAYIIHAKD--SD-KHQYNPDEVITLLGKDYQEVY-Q--ERQ-KSWL-QGRAK

QKQED-IDLLLDD-ILNERI-TKQELLL---N-PSHLLYVVHKTRINE-AFRAI------

-----------G-EIKGTRTKQDLENGLF-----KKTILFIYGKSGLGKTRLAKELVSL-

LEQLASVQSVLT---AGTN-IFD----EVNGE-EILLLDDVRG-DSLTASDWLKILDPYS

---ISPISARYQNRI-G---SAKVIIITSTKHPLEFFYHTK---GNDREDLSQYIR----

--RFDFLISL-ESEREN--LVYFESSPTKVYQRYLSY--DFSANARLANKSYLLELVLAK

IGLNNQ--W-----KLKTSSD-DEAEHS

>pCRESS6|WP_001034312.1

M----NKKEAN--LSCIMIVQ--RLEPEYWHLNELTPLLEEVVNRV------SEIATVSD

AYA-IIHDKDINEVTKPE--KPHAHILLKFSKGETLINLSLQLGIE-----PQ-YIEKAK

AGAFDNFLAYLIHAKD--SD-KFQYDPKEVITLHGKDYLEVV-T--ERY-KSWK-KGRAK

SKNTS-LDEIYLQ-ILNQQI-SKQEILS---D-PEQILYALNKTKINE-AFMTL------

-----------G-EIKSNATKQALENGEF-----KKTIIFITGKSGLGKSRFAKTFVKE-

LISLANISDVVT---AGTN-IFD----EVNGE-EILLLDDVRG-DSFTASDWLKLLDPYN

---ISPISARYHNRM-G---SARVIIITSTKHPLEFFIHTK---GNEKEDLSQFIR----

--RTTSLVTL-YRDSPSCDTRYFHSSPKSVPNRYMSY--DFENNSEI-SKENLQEFLLAQ

VSINNR--W----DKIKNSSE-DEFQIS

>pCRESS6|WP_024385235.1

M----SKKEAK--LTCIMITQ--QLESEYWDIDDLSLLLEEVVNRV------SEIAKVSD

AYA-IIHDKDVNGITVPE--RNHAHILLKFSKGATLISLALQLGIE-----PR-YIEKAK

SGAFDNFLAYLIHAKD--SD-KFQYDPKEVITLHGKDYLEVV-T--ERY-KSWK-EGRAK

SKYTS-LDEIYLQ-ILNQQI-TKQEILS---D-PKQILYALNKTKINE-AFMTL------

-----------G-EIKSNATKLALENGEF-----QKTIIFITGKSGLGKSRFAKTFVKE-

LISLANTSDVVT---AGTN-IFD----EVNGE-EILLLDDVRG-DSLTASDWLKLLDPYN

---ISPISARYHNRM-G---SARVIILTSTKHPLEFFFDTK---GNEKEDLSQFIR----

--RTTSLVTL-YSDSPFSNTRYFYSFPKSVTNRYLTY--DFENNSVI-SKEKLLEFLLAQ

VSINNR--W----DKIKNSSE-DE----

>pCRESS6|WP_004183001.1

M----SKKEAK--LSCIMIAQ--QLEPKYWNLNELTPLLEEVVNRV------SEIAMVSD

AYA-IIHDKDVNEITEPE--NVHVHILLKFSKGATLISLALQLGIE-----SQ-YIEKAK

SGAFDNFLAYLIHAKD--SE-KFQYDPNEVTTLHGKNYLDVV-T--ERY-KSWK-KGRAK

SKNTS-LDDIYLQ-ILNQQI-SKQEILS---D-PKQIIYALNKTKINE-AFMTL------

-----------G-EIKSNATKLALENGEF-----KKSIIFITGKSGLGKSRFAKTFVKE-

LISLANISDVVT---AGTN-IFD----EINGE-EILLLDDVRG-DSLTASDWLKLLDPYN

---ISPISARYHNRM-G---SARVIIITSTKHPLEFFFHTK---GNEKEDLSQYIR----

--RIDSLVTL-YRETPKSSVEYLQSSSESVANRSLIY--DFKHNGKM-SKEELLEFLLAQ

VSLNNK--W----DKIKNSSE-DE----

>pCRESS6|WP_024400359.1

M----SKKEAK--LSCIMIVQ--QLEPEYWDIEELTLLLEEVVNRV------SEIATVSD

AYA-INHDKDVNEITELA--SPHVHILLKFTKGSTLTNFALQLGIE-----PQ-YIEKAK

VGGYDNLLAYITHQKD--PE-KFQYDPKEVITLQGKDYLEIV-K--ERY-KVWQ-QGRVK

SKNAS-LDEIYLQ-ILNQQI-SKQEILS---D-PKQILYALNKTKINE-AFMTL------

-----------G-EIKSNATKQALENGEF-----KKTIIFITGKSGLGKSRFAKTFIKE-

LISLANTSEVVT---AGTN-IFD----EVNGE-EILLLDDVRG-DSLTASDWLKLLDPYN

---ISPISARYHNRM-G---SARVIIITSTKHPLEFFFHTK---GNEKEDLSQFIR----

--RTDTLVSL-TEDFVNGPVRFFQATPEKVSNRNLSY--DFKKNYQS-DKESMLEFLLAQ

ICINNH--W----DT-------------

>pCRESS6|WP_000791389.1

MK---NKKQAK--LTCVMIAQ--QFDSQFWDL-SIKDILTEMVNRA------NTVATVSE

AYG-IKHDKDTLELQELK--SEHGHFLLKFSEGATLIDLALAIGVE-----PQ-YIEKAK

SGGYDNLLAYLIHSKD--PD-KFQYSASDVITILGKDYLEIY-K--EKF-SSWS-KGRTK

RNSEQ-IDSLIAN-ILNGKI-TKSEILL---S-SNLQLYALYKTKINE-AFMTL------

-----------G-EVKATRTKKALENGDF-----KKSIFFITGKSGLGKSVLSRELVGD-

LIQLAENSSVAT---AGTN-IFD----EVNGE-EILLLDDVRG-DSLTASDWLKLLDPYN

---ISPISARYHNKM-G---ATRVIVITSTKHPLEFFFKTK---GNEIEDLSQFIR----

--RFDSLITI-DNTAETTPIRYFQSLPKRVVNRYLSY--DFENNSEM-NKKDLLDFLVAQ

VSLNNK--W----DKIKNSLE-DEVDTE

>pCRESS6|WP_003032217.1

-----MAKESK--IQCFMVVQ--YFSEKYWSFEDIAPLLNEMVKRI------SKITTVSE

AYG-IVHDKDEEELTKPI--DSHGHLLFKLSEGMPIIKLSDIIGIE-----PQ-YIEKAK

RGGYDNLLSYLIHSKE--SN-KHQYSPQEVVSAAGKNYMEVF-K--ERF-SSWK-KGRVK

KLEDE-IDNLILD-IIQEKI-GKSEILL---D-EKHMIYTTFKTRINE-AFAVL------

-----------G-EIKGNRTKKAIENGEF-----KKTVLFIHGRSGLGKTRFAKELASK-

IQGLALRDLATT---AGTN-IMD----DLNGE-EILLLDDVRG-ESLTASDWLKLLDNYN

---ISFSSARYKNIL-P---SARIIIVTSTKHPLEFFYKCK---DNEREDLSQFIR----

--RFDSLITL-QNCQEGDNIQFFQASPRKTSNVTLNH--DFEKNNFM-NKGELVEFLLAQ

VSLNNK--W----EQEKSLSD-DKPSK-

>pCRESS6|WP_018376545.1

M----GKNKAI--LTVIAGSQ--QLRTELWKAEAPDELLKEMIEHL------SSVAKISD

AYI-IVHDKDTKYVQVLK--SFHVHFLFKFSEGATLTEIANALNLE-----IQ-YLEKPK

SGAYDNLLSYLVHAKD--HN-KFLYSPSEVVTLLGEDYETIY-N--ERI-HVWE-KGRAK

QQKFD-VEALIMD-IIVGKI-KKTEVLS---S-PDYPIYAFNKSKLNE-AFETY------

-----------A-ERKSLKTLKDLELGLF-----QKTIIFFQGASGLGKTTLAKELTHE-

IQLLSKAEAIIT---AATN-PFD----DVKGE-EIILLDDVRG-QALSASDWLKLLDPFT

---ASPISSRFKNRS-G---AVKTIIITSRKPPLEFFFKTK---DSGYEDLSQFIR----

--RIHHFITL-RTDGTG-NTIYSSSFPERKQTPSLDY--DFSTAKEM-GSKDLLKFLIET

VRLNNR--W----SYEESKKA-AESDG-

>pCRESS6|WP_020999261.1

M----TKHKAN--LTIVAGAQ--QLRTELWEIDTPEELLREMVKHL------SSVATVSD

AYI-IVHDKDKKEVQVLK--ASHVHFLFKFSEGGTLSDIANALGLE-----TQ-YLEKPK

SGAYDNLLAYLVHAKD--SN-KFHYSPSDVVTLIGENYETLY-N--ERI-RAWE-KGRAK

KLKFD-VEALIIE-IIEGKI-KKERILS---S-PDYPIYAFNKTKLNE-AFETY------

-----------A-EQKSMKTLKNLELGLF-----QKTIIFIQGSAGLGKTTLAKKLARE-

LQLLAQAEYIIT---AATN-PFD----DVKGE-EIILLDDIRG-QALSSSDWLKLLDSFT

---ASPISSRFRNRS-G---AVKTIIITSSKHPLEFFFKTK---DSGYEDLSQFVR----

--RINYLITL-RMGNSG-DTIYSNSHPKRKHTPSLDY--DFSQAKTM-SAEDTLAFLIET

VRLNNH--W----SYEEYKKA-TESDS-

>pCRESS6|WP_044762265.1

M----TQKQSN--LTVVAGTQ--QLEFEFWELDNAKELLHKMVEYL------STIVVVAD

AYI-IIHNKDWSEVKVLK--TIHVHFLFKLAEGATLPEIANALGLE-----TQ-YLEKPK

SGAYDNLLSYLIHAKE--PD-KFFYSPSEVVTLMGRDYQTIY-N--ERI-RTWE-KGRAK

KQKND-VEVLIMD-IIKGKI-KKDEILN---N-PEYAIYAFNKSKLNE-AFETY------

-----------A-ERKSLKTYKDLELGLF-----QKTIIFLQGASGLGKTTLAKELAHK-

LQFLSRVETVIT---AATN-PFD----DVRGE-EIILLDDVRG-QALSASDWLKLLDPFT

---ASPISSRFKNRS-G---AVKTIIITSSKSALEFFFKTK---DSGYEDLSQFVR----

--RINHFITL-RTDSSG-NIIYSSSFPERKHTPSLDY--DFSTAKEM-SSENLLEFLMET

VRLNNH--W----SYQDSKKA-KSDDLN

>pCRESS6|WP_054952722.1

V----AKQENN--PTSIGLTQ--YLDPSYW--QGAEAILAYVVQRL-----EATGCEVVE

AYG-IVHDKDEREVVEPK--PEHLHAVIKFAKSAPLDRLAFGIGVE-----PQ-YVEKPG

RGAFDNMLSYLTHVKY--AD-KHQYAPSEVATVRGPDYLGID-A--QRR-ETWQ-KGRAH

KIAEN-FEDMRER-VLQGEI-TRDQIML---T-DEFDIYSRHQREIDD-ALSAY------

-----------G-QRRAYRAAAKLRAGEF-----STHVVFVHGDAGIGKTRFATDFITE-

AINAANAQVYRA---ATGN-PLD----DWRGE-EVLLLDDLRA-SAMDANDWLLLLDPYN

---ASPAKARYKNKG-E---VARLIVITATIEPVEFFYYAM---GNVDEALDQFIR----

--RLASVVKVYADD----INRYLVQHIGKIEPYELTY--GPETSAEH-DADGAVAELLGG

LAVRSPDV--------------AA----

>pCRESS6|WP_041290927.1

V----AKQENN--PTSIGLTQ--YLDPSYW--QGAGPILAYVVQRL-----EAIGCEVTE

AYG-IVHDKDEREVVEPK--PDHLHAVIKFAKSAPLDRLAFGIGVE-----PQ-YVEKPG

RGAFDNMLSYLTHVKY--AD-KHQYAPSEVATVRGPDYLGID-A--QRR-ETWL-KGRAH

KVAEN-FEDMRER-VLQGEI-TRDQIML---T-DEFDIYSRHQREIDD-ALSAY------

-----------G-QRRAYRAAAKLRAGAF-----STHVVFVHGDAGIGKTRFATDFITS-

AIDATNTQVYRA---ATGN-PLD----DWRGE-EVLLLDDLRA-SAMDANDWLLLLDPYN

---ASPAKARYKNKG-E---VARLIVITATIEPVEFFFYAK---GNVDEALDQFIR----

--RLASVVKVFADD----INRYLVQHIGKIEPYELTY--GPESSVEH-DADSAVVELLSG

LAVRSPDV--------------GAA---

>pCRESS6|WP_036321578.1

M----ARKAAN--PTLVMLVQ--QIKPECWGLRAAASFSQYLVVLL-----ESVGLVIEE

AHC-ILHGDDTQLGEVLK--YLHLHLLLKFKTSAAVEKLAAILGVE-----VQ-YVEDKS

RGQHDNGLAYLTHVKY--PD-KFQYPPEQVASVRGMEYQQVY-R--ERY-PAWR-KGRAH

KRKLE-FEPFREM-VLQGEL-TRDQIML---T-DEFDIYSRHQREIDD-ALSAY------

-----------G-QRRAYRAAAKLRAGAF-----STQVVFIHGEAGVGKTRFANAFIQE-

AISCAKGQVYRA---ATTN-PLD----DWRGE-EIMLLDDLRA-SAMDANDWLLLLDPHN

---ASPARARYKNKG-E---VARLIVITATIEPVEFFFFAK---GNVDEALDQFIR----

--RLQSVVRVFEDD----ILRYLVQPVVKGDPYSLSY--GPAGGGAH-DAGAAVDELLDG

LAEHSRDV--------------GTA---

>pCRESS6|WP_051176704.1

-----MSGGNK--LQIFHITQ--QLRPELWELRRANRLLSLLVLLL-----EQTTLKIVE

AHG-IIHNCDEQNTMEPK--FPHLHATVKF-PTFKIARIAKILGVE-----TQ-FVEKP-

NGSHDAQLAYLIHAKD--ED-KYQYEPEQVATVAGQDYLEIQ-K--QRE-AEWF-LGRGK

KLALS-VDALLEQ-VLSGNI-TKENVLL---S-DEFRVYSELSDKFNR-AFVVA------

-----------G-ERKAYRAAEAMKNGDF-----HTAIFFLYGAAGSGKTRLANLLVDQ-

LIESTN-SLYRA---ASRH-SLD----DWQGQ-EIILMDDVRA-SAMSASDWLTLLDPYN

---PNPASARYQNKL-A---VARLVIITASIDPVTFFYYAK---GDVDEALDQFIR----

--RLMASVNVITDG----MPRYYYKRLGLLSERELTR--GVVDSIKC-DEPEIIARLSLD

VANSSPDVWV---EQSSSSHD-DIEFFD

>pCRESS6|WP_052506726.1

-----MANKDK--LSMCMITQ--AWLSNHWDDNNLSLVTQKFVEYL-----SEAGAEVVA

CYG-ITHDRDTREVLEPK--HLHGHWVVKFAKGLTLSDIAVAVGLA-----PQ-FIEKAK

PGGYDNMLSYLIHAKD--AE-KFSYSANDVVSVVKNEYSEIY-A--ERK-SAWE-KGRAK

KRDEL-VEELYEK-VINGEV-THDQILL---T-DEYAVYSRNIDRFEK-GFKAY------

-----------T-DRKILQAVRDLEAGKF-----NLSVLYFQGQAGHGKTATAVQLAQS-

LVVKAAESICQA---AATN-PVD----DYNGE-EILIMDDLRG-NAMRATDWLKLLDPYN

---SSPNSARYKNKR-V---VSRYIFITSIQDVYEFFYYTG---ADRSEPLDQFMR----

--RILALVKVVVGDEKRIGVYPQVSYDYRKRVETLHH--GFYAKGLH-FTSDNFGQFVEH

LFDDEKRV-----NDERNAVN-DIVADD

>pCRESS6|WP_022765681.1

M----ARTESD--LTACGIAQ--ELNPELW--FDLQRVTAIIAKRL-----EDKGIQLKS

LRG-VIHDSDTQQKVTLK--HLHAHWVIEFKKGAKLTDIALAIGLA-----SQ-YVEKPK

KGAVDNMLAYLIHIKY--PD-KYQYSPTAVYTYCGRDYMDIY-A--ERE-SVWK-KGIAT

LAAED-IDWLEEQ-ILEGKI-KKTQIFL---T-DSFKTYSHYRQRCED-AFACY------

-----------T-DKKIYTAIEAMKNKEF-----LLTTYFITGASRKGKSRFAEDLAQR-

IIDENEKSYCRT---PTSN-PLD----DYAGE-EIIIMDDSRG-CTLTAEAWLTFLDPNF

---CNPAGARYHNKP-G---IPKVLIITSTKSMLEFFFYTG---GSRSEAMDQFFA----

--RVFSRIEVIFDT-------YIIDKIEKAESYELRY--APVSLGTA-TKEEAISLLSEA

VLENVQHIYAS--DKKRAKIN-HKEEQE

>pCRESS6|AEU41945.1

MT-KPKAKRKQ--ASVFGFTQ--QFKADMWAVVDTARIMKRVAERLYK---ANAAKDL-K

YSA-IIHDKDMSFAIVPK--ELHMHAVIELPSKRDLSFISTAIGIR-----PE-QIEVP-

RGGRENMLAYLVHAKD--GD-KYQYSPSEVETFNTWDYMSYY-I--SRV-DTWN-YRKAT

KASVK-ADWLVKE-VQAGRV-SKETIML---T-DDAEVYADNMRAIND-AFQYY------

-----------A-ERQGFKTLEALRNNEF-----EMSVYYIQGAPRMGKTFFAKKFVND-

LIDTRFARSYET---ADTQ-PMD----DYAGE-EIVFMDDLRA-SAMTASGWLKLLDPLT

---TAPMSARYKNKQ-K---ATRVIVITSYLDPFTFFSYVK-GVGGSNEALEQFIG----

--RLSLIAKVLGDDRSSEAKHFVVGSNTKLLPRSTKF--------ESGDVSMALEFMIDD

VMDSSKKH-----EKPRAGFH-DVKSWN

>pCRESS6|WP_025016923.1

------MLDKR--VRSFIFTQ--YLNPKYW--INKQAIFEEIFARA-N---LEG---LKI

CAL-IVHDKDENK-G-LK--DAHIHGYLEFSKQKTIASISSVLAIE-----PQ-YVEAPK

KGGRLNCLAYLIHAKN--LD-KYQYSPEEVETFETFDYEEFI-D--DNL-EDFK-KYNAT

KKEIG-LDLALQE-VQQGKL-KLREIMR---D-ENALLYANHMNQFND-SFNFY------

-----------G-LRNAMLRLDELEQGKY-----DLTVLYIQGAPGIGKSFLAREVAQK-

VREYGNKDVFSA---SSSN-PFD----DYYGE-DILILDDLRQ-ESLKVSDWLKLFDPLN

---TARMSARYRNKM-I---VPRLVILANYQSIEQFFGSFN-----KNEDINQFIR----

--RISFSSKI-TEKSPPFDRIYSLSASKKLNE-TLNY--GTKLLFTYDDKEKFINELLTQ

HV------YPRI-EK-------------

>pCRESS6|KST89836.1

MHEKKKMRDKR--VKSFIFTQ--YQQADFWKIRNQAKIFNEIYNRL-K---LEG---LKI

IAL-VNHDKDKNA-GELK--HPHIHGYIEFEKLKTIASVSACLGIE-----PQ-YVEVPR

KGGRLNCLAYLVHAKN--LD-KYQYSPEEVQTFETFDYQEFI-D--DNL-EDFK-KYNAT

KKEIG-LDLALQE-VQQGKL-KLREIMR---D-ENALLYANHMNQFND-SFNFY------

-----------G-LRNAMLRLDELEQGKY-----NLTVLYIQGAPGIGKSFLAREVAKK-

VREFGNQDIFSA---SSSN-PFD----DYYGE-DILILDDLRQ-ESLKVSDWLKLFDPLN

---TARMSARYRNKM-I---VPRLVIVANYQSVEQFFGSFN-----KNEDINQFKR----

--RINFTSKI-SEKSPPFDRIYSLSASKKLSE-TVNY--GTKLLFSYDDRNRFINELLTQ

HV------YPRI-QKEKK----------

>pCRESS6|WP_018030886.1

-----MSDRKR--ATTFIFEQ--QLKSDYWKKKNRVAIFKEIHRRI-Q---LEESIPAK-

VAF-IIHDKDKKY-LTLV--EPHIHGYIEFASRRDLNHLASVLGLL-----PQ-YIEPSG

RGGKVNSKAYLIHAKN--PD-KYQYSPDDVETFGTFDYKEFF-Q--EHQ-LDFI-KRSAT

EKDEE-LDSVFQA-IVNGTL-TEDDIFA---N-EETFLWSYNQTKLDE-AFRAY------

-----------G-KIASKRTLRQLESGEF-----QPAVIYIHGSSGIGKTSLALELIEQ-

IIKRAKEKMYSA---GTKN-IFD----EYFGE-EIILLDDPRY-DSLLPADWLKLLDPLN

---KSHLSARYKNKL-V---IGRIIIITNYKSLKSFFGKIQ------HEDLNQYIR----

--RFNNVLEI-SKKNSEKDRFFNLSQIQELREASLLF--GEEKVYSTDDKEAFINHVLEY

YI------FPRI-ETKSAPLD-------

>pCRESS6|WP_003104234.1

------MSRKR--TTTYIFEQ--QLHAEYWKAENKTKIFQEIHRRI-K---LEEDIPAK-

VAF-IVHDKDIKY-IKPV--EPHIHAYIEFASRRDLSVLASTLGLL-----PQ-YIEPSG

QGGKVNSKAYLIHAKS--PD-KHQYAPSEVETFGTFDYVAFI-E--DNR-ADFS-KRYAV

EKDES-LDKVFQE-IINGNL-TEDDIFA---D-EETFLWSYNQTRLDE-AFRAY------

-----------G-KIASKRTLRELENGEF-----KPTIIYVHGSSGIGKTTLALEVIEE-

IIKRAKEKMYSA---GAKN-IFD----EYFGE-EVILLDDPRS-DSLLPADWLKLLDPLN

---KSYLSARYKNKL-V---IGRLIVITNYQSLKSFFGKIQ------NEDLNQYIR----

--RFNNVLEI-SKKKKEKDRFYNLSEVKELSY-QLHF--GEEDIYCTDNKADFINRVLDE

HI------LPRI-QIKKRPQN-GNA---

>pCRESS6|WP_039670385.1

-----MAKRKQ--TTTFIFEQ--QLKGEYWKAKNKQEIFQEIYNRV-K---LETDSSLK-

IAL-IVHDKDKTF-NNLV--FPHLHGYIEFSNKRDLSVLALNLGLY-----PQ-YIEPSG

RGGKINSKAYLIHAKS--PD-KYQYQPSEVETFGTFDYERFI-Q--ENL-DSFT-KQSAK

EQDES-LDMIFQE-IIKGKL-TEDDIFA---D-EKTFLWAYNQQKFDE-AFKAY------

-----------G-KISAKTTLRQLENGEF-----KPTILYIHGQSGIGKTSLAYDLVAE-

IVLQAEKKTYNA---GSKN-IFD----EYFGE-EIIVLDDPRY-DSLLPSDWLKLLDPLN

---KSYLSARYRNKL-V---IGRVIVITNYMSLSEFFRQIP------KEDINQYLR----

--RFNNVIEI-TKSG--NDRQYNLSEIRELSVP-LNF--QEFEVMSSTDKKEFLDKILEE

YI------YPRI-DKQKIQLV-CKK---

>pCRESS6|WP_018380019.1

-----MTKRKQ--TTTFIFEQ--QLQSDYWKDENKRAIFQEIYDRV-K---IDEENPIK-

IGL-IVHDKDVSF-SRLV--KPHVHGYIEFKTKRDLNILALSLGLL-----PQ-YIEPSG

KGGKINSKAYLIHAKS--PD-KYQYEASEVETFDTFDYEQFI-A--ENK-EDFA-KQSAT

EKDES-LDLVISK-VQKGEL-SYQEVME---D-DEAFLFANNQQKFRE-SFNFF------

-----------G-EREAFLRLKSLERGDY-----QLTVLYIQGEPDVGKSTLAKEIALK-

VKAKMNDDIYSA---SSSN-PFD----NYYGE-EILFLDDLRE-YNLSASDWLKLFDPLN

---SARMSARYQNKL-V---IPRLVIMPVYKTPKTFFGEVQ------AEDLNQFLR----

--RINFLLDI-SLKH---DRLYNVSELVKRKA-VLNF--KYEDMFCSDDKEWFVNKLLED

CV------YPRI-KKVKDVTN-------

>pCRESS6|EOB33201.1

MVSKKMAKRKQ--TTTYIFEQ--QLKPDFWKKENKIEIFKEIYERV-R---ISESEDLK-

IAL-IIHDKDISY-TKLV--EPHIHGYIEFSNKKDLNVLALSLGIL-----PQ-YIESSG

RGGKINSKAYLIHAKD--KD-KYQYSASDVETFDTFDYEAFI-N--QNR-EDFE-KYAAT

EKDES-LDLTLSK-IQLGEL-TYNDVME---D-DSAFLFGNNQQKFRE-GFNFY------

-----------G-ERQTFLRLKSLERKEY-----QMTVIYIQGDSDIGKSELAKNIALQ-

AQAKLNEDIYSA---SSSN-PFD----NYLGE-DILLLDDLRE-DTMRASDWLKLLDPLN

---SARMSARYQNKL-V---VPRLIIMPVYMSPKLFFGRIK------AEDLNQFLR----

--RINFLVDV-SLKH--VERLYNISEVVKRKG-VLNF--RYEDLFCAHDRDVFIEKIVEE

CI------CPRI-KEVKDVTN-------

>pCRESS6|WP_044671103.1

-----MGKRII--NKRFLFEQ--QLKSKFWKQENKGKIFRLIFDRV-R--TVEEDEFVE-

FAI-VIHDKDISY-TKLV--EPHIHGYIDYPKRIDLSKVASALGVE-----RE-RIEPKS

KGTRINALAYLIHAKD--KD-KYQYPVSDVETFDTLDYETFI-N--QNK-EDFE-NFSAT

EKEER-LDLVLSK-VQSGEL-TYLDVMK---D-DKAFLFANNQQKFRE-SFNFY------

-----------G-EREAFLRLQALQRGDY-----QLTVLYIQGKPGIGKSTLARDLALE-

TQKRLNAEIYSA---GSKN-PFD----NYYGE-EILLLDDLRK-DSISGTDWLKLFDPIN

---SARMSARYQNKL-V---VPRLVILSAYMSPKTFFGQIE------TEDLNQYLR----

--RINFSSEI-SLKY---DRYYSVAQVKEHKE-VLNF--DFEDLFSMQDKSQFITKLLDD

YI------YPRI-CEVKNV---------

>pCRESS6|WP_003048523.1

-----MTKRIR--NRRFLFEQ--QLKTSFWKQENKDKIFRLIFDRV-R--TVEEDEFVE-

FAI-VVHDKDVSY-TKLV--EPHVHGYIDFPKQIDLSKVASALGLE-----RE-RIEPKS

KGTRINALAYLIHAKD--KD-KYQYPVNDVETFDTLDYETFI-N--QNI-EDFE-KYAAT

EKDES-LDLVLSK-VQKGEL-NYLEVME---D-DEAFLFANNQQKFRE-SFNFF------

-----------G-ERETVLRLKDLKKGNY-----QLTVLYIQGEPGIGKTHLANELILE-

VSKRLREEYYPA---SSKN-PFD----NYYGE-EILFLDDLRE-DSLSASDWLKLFDPLN

---SARMSARYQNKL-V---VPRLIVMTAYMSPKQFFGNIK------TEDLNQYLR----

--RVNFSTEI-AKKH---DTFYSVSEVKKNKA-VLNF--NYEDLYSSQNKDEFITKLLEE

YI------YPRI-KNVKDVTN-------

>pCRESS6|WP_039694464.1

-----MAKRIR--NRRFLFEQ--QLKSEFWKQENKNEIFRTIFDRV-R--SVEENEFVE-

FAI-IVHDKDISY-TKLV--EPHVHAYIDFPKQMDLSKVASALGVE-----RE-RIEPKS

RGTRINALAYLIHAKD--KD-KYQYPVSDVETFDTLDYETFI-N--QNR-EDFE-KYSAT

EKDES-LDLILSK-VQTGEL-NYIDVME---N-DNAFLFANNQQRFRE-SFNFF------

-----------G-ERETVLRLKDLKQGNY-----QLTVLYIQGKPGIGKTHLANEIILS-

VAEKLREECYSA---SAKN-SFD----NYYGE-EILLLDDLRE-DSLSPSDWLKLFDPLN

---SARMSARYQNKL-V---VPRLIVMTAYMSPKQFFGQIE------TEDLNQYLR----

--RVHFSTEI-AEKH---ERFYSVSGVAKNNA-ILNF--GYEDLYSSQNKDKFINKLLEN

YI------YPRI-KENKIINY-------

>pCRESS6|WP_000746010.1

-----MKKRII--NKRFLFEQ--QLKPKFWKQENKDKIFRLIFDRV-R--TVEEDEFVE-

FAI-VVHDRDISY-AKLV--EPHVHGYIDFPKKFDLSKVASVLGVE-----RE-RIEPKS

KGTRINALAYLIHAKD--KD-KYQYPASDVETFDTLDYEAFI-N--QNK-EDLE-KYAAK

EKDES-LDLVLSK-VYKGEL-TYFDIMK---D-NNYYLMANNRQKFLE-GFDIF------

-----------G-ERESVLRLEALQNGEY-----DLTVLYIQGKPGIGKSTLARDIALE-

VQGALENGSYSA---SSKN-PFD----NYSGE-EILILDDLRE-DSLAPADWLKLFDPIN

---SARMSARYRNKL-V---VPRLVIMSAYMSPKQFFGQIQ------EEDINQYLR----

--RVNYSSEI-ARKH---ERFYSVSEVRENRE-VLNF--DYEDLFCSQDKDDFIRKLLED

CI------YPRI-KKAKDVTN-------

>pCRESS6|WP_019299400.1

------MNKIR--IKNFVFEQ--YFDQEFWKEKFKENIFQEIYDRL-K--FDTEDKNIK-

VAM-IVHDKDKKW-DKLI--EPHIHVYVELPTKRSIERIADRIGIS-----QH-FIEPKG

KNFPFNEKAYLIHAQQ--PD-KYQYEVNEVETFETIDYEQFV-L--ENE-KEFL-KRSAT

KTDES-LDLIFDK-VLFGKI-TYDEIME---D-ETYRLYANNEQKFIS-AFNSF------

-----------A-QYKARKTLKALKNKEF-----KMSVIYIQGKSGIGKSHLAGEIVEK-

ICEESEKSVYSA---SASN-PFD----EYKGE-EILLLDDLRP-DSLERADWLKVLDPMN

---KSRISARYRNKA-I---ASRVIVLTNTETAEQFFKNIK------NEDLDQYIR----

--RINLTVGI-DEKQFDYDSFYRLSESKKLLE-ELRY--GFEPVFSTENKEEFLDRLVHK

EI------LPKS-V--KG----------

>pCRESS6|BAM66968.1

------MKKKR--IKVFVFEQ--YFDSEFWKSENKDKIFQEIYDRL-K--FDTPETQLK-

IAL-IVHDKDRKN-GQLI--DPHVHVYVELPTKRSLELVADRIGIS-----QH-FIDANG

RNFSFNQKAYLIHAQQ--PT-KHQYELSEVETFGTIDYEKFI-L--DNK-DDFL-KRSAT

KENES-LDLIFDK-VLFGQI-TYDEIME---D-DSYRLYANNEQRFIS-AFNSF------

-----------S-QYKARKTLKALRNCEF-----KLSVVYIQGNSGIGKSHLAQEIVEK-

LCDESEQSIYSA---SSSN-PFD----EYKGE-EIILLDDLRP-ESMERADWLKVLDPMN

---RSRISARYRNKV-I---AARVIVLTNTETAESFFKNIK------NEDLDQYVR----

--RINLNVGI-TEKQFEYDNYYNLSETKKLGE-ERRY--GFQPVFSSDDKELFLKKLIFD

EL------LPKS-KD-KGVIR-------

>pCRESS6|WP_032941943.1

------MEDKR--IKQFVFEQ--QLKAEYWLKENKLLIFEEIYRRL-K--TESEKVKLEC

IAI-IVHYLDKND-NEFI--FPHVHLYGKYTDKRTLARIAKVLGIK-----EQ-YIEPKN

GNFEENQLAYLTHAQQ--PD-KYQYPTHEVETFGTFDYSNFI-L--INT-KKFE-KQSAT

KRDES-LDLINQQ-ILKGEL-FLEDILA---D-DDFLLYSNHKLQFKQ-AFDSY------

-----------A-ERLAFKNLKDLTTGKY-----KLTVMYFQGKSSLGKSYLARTIAQK-

VREYAEERIYSA---SSSN-PFD----DYYGE-DIILLDDIRP-DSLRKAGWLKLLDPIN

---TSRMSARFTNKQ-V---VPRLILITNTQLPEQFFNIFK------DEALDQYIR----

--RINFCTIL-SEKQYEGGVYYQLSQTKRLFS-EINF--DLEAIFSTDNQEEFTEKLLAR

YL------LPRI-KT-------------

>pCRESS6|WP_058223604.1

MY---FIEEKR--IKQFVFEQ--QLKAEYWLEQNKALIFEEIYRRL-K--TESDKVKLEC

ISL-IVHSLDKND-YELV--FPHVHLYGKYSDKRTITRIAKVLGIK-----VQ-YIEPKD

KNFEENQLAYLTHAQQ--PD-KYQYPPLEVETFGTFDYSNFI-L--SNA-KKFE-KQSAT

KRDES-LDLINQQ-ILKGEL-FLENILA---D-DDFLLYSNHKLQFKQ-AFDSY------

-----------A-ERLAFKNLKDLTTGKY-----KLTVMYFQGKSSLGKSYLARTIAQK-

VREYAEERIYSA---SSSN-PFD----DYYGE-DIILLDDIRP-DSLRKADWLKLLDPIN

---TSRMSARFTNKQ-V---VPRLILITNTQLPEQFFNIFK------DEALDQYIR----

--RINFCTIL-SEKQYEGGVYYQLSQTKRLFS-EINF--DLEAIFSTDNQEEFTEKLLAR

YL------LPRI-KIEKSKMT-------

>pCRESS6|WP_061343647.1

MA---QSKRNQ--LKSFYGTQ--QLEQEFWDNRNQRRIFEIIATRFLR---KSDNGNIAK

IAF-VLHDKDKDDTGNFI--KPHVHWLLELKNKRDLDEIAYKFFVH-----PQ-QIEKGS

KGCFLGRIGYLTHQAE--PD-KFKYDVHDVETFGTFDYADYI-S--KNS-AYFK-KRLAF

QQKMD-VDYYLQQ-VQQGIL-FLDDIFL---D-LNYNVYANNKQKFRE-AFDAY------

-----------S-ELNSFRTNRDKRLGIF-----DFTTIFIYGRSGLGKTTIAMAILDR-

LKELAKSRSYSG---SAKN-AVD----DYKAE-ELILFDDLKQ-DSFLIADWLKILDSRN

---ESTISGRFHNKP-L---SARLIILTTIESPFKYFDFG------KDEPKEQFIR----

--RLSYIINV-SSDEQMIKTNFLINTPN-----QDNY--HLKEKYKEIGMDNLLNEIYER

IK-----------GEGRKWIT-------

>pCRESS6|WP_017371219.1

MT--IKAKTIQ--AKTFFGMQ--YLEKEYWETRNQKEIFALVCEKF-R---QDYQDDIVT

LAI-VLQDKDINENGKLE--APHLHWAIHLKERTTLNKIAKAFQVE-----PQ-YIETGN

QGAMIGRLAYLTHQTE--PD-KFHYDPQDVETFGTFDYVNFV-N--NNK-MKFK-KLLAT

RAKYE-LNHLLQQ-VQVGKL-FIEDILS---D-EKYFVYANNLAKFKE-AFEAY------

-----------A-QRNSLLTIQDRVAQKF-----EFTSIYIYGKSGSGKSEIAYDILKQ-

IELLSQEHSYFG---GSKN-AVD----DYKGE-ELLLFDDVRP-ETFSPADWTKILDYKN

---KSALSGRFHNRP-L---SNRLVLMTNTQSPFEFFK--------ENEPIEQYLR----

--RLTYVIRV-EADEEMKQTKFTILAPD-----ACNY--HLREVYQLTGKAELYDTLFKQ

LM-----------NKEKKPSL-DGKSDS

>pCRESS6|WP_014571792.1

V----QQTQIQ--NIRFHITQ--FLKKEYWAVIFQKQIFMALIPLG----------LVK-

FAS-IIHDKDLQL-VTPK--PPHIHAIVEFEKKKDINVVALALGLE-----PQ-YLDTAK

RGAEENLLAYLIHAKD--KT-KYQYSPKEVSTFGTFNYMAYE-E--EHR-KRWK-NQAIV

KKDEQ-LPILLEA-IREGAL-NREQVLE---N-PDLFLYQRHIMEFQA-AFEGY------

-----------L-LKAEAETKKAIKSGEL-----KLTTLYIYGLSNAGKSRFAETLGET-

LKAKVRGTSYTT---ASTN-PFD----EYTGE-QIVILDDLRA-KSMTAENWLKILDPER

---ISKSAARYHNKI-I---SSHLIIITAPISPQSFFHQIS-----ESEELNQFMR----

--RLSLTIEI-SQGK--KERLYTVNSIERKKNNSLNK--ALGSC---------ITKLLNN

DK------TINK-SDIKAATL-PLG---

>pCRESS7|CDE19587.1

---------MK--LKQYEVVT--QPE--YLK--------SPLQDIL------RKYRTIKQ

WAY-ILHDKDK-----DA--SSHYHIYINFQQTVDSKDVAGWFGIP-----EQ-FVNKVE

GR-KTDMLMYLTH--SNDSQHKHQYDFSEVVAN--FDFKSEI-E--QAK-----------

---------II------GDF-EKYSYIH---S-LAVSEQPKCFDRLQK-LWKLQ------

-----------C-QWLS-------LNSDR-----NLKVIFVTGKSGTGKTYFARKYMRA-

--H-ELDCYVSS---SSND-PLD----MYMGQ-KGIIFDDLRD-EAFEFADILKLLDNNT

---STAMKSRFTNKV-L---NCKVMIITSFIPIKYWYKSVR--YS--CDGIEQLYR----

--RINMYVHI-TEDEIV---VYDGLSDKYVNEV---K--KLK-EENRKQREDAFGDFDFL

TE--KKDKF-------------------

>pCRESS7|CCZ68460.1

MRK--QGN-LS--MRRCEIVS--NLESELFD-------IERMKQVL-E---EKSKTCIKE

FSY-IIHDKDVYTEGELK--PKHIHLLLRFNQPQKLKNIAGWFQIP-----PN-FVSKIH

NR-WDSAVLYQIH--ANCPE-KYQYDISEVTAN--FKIENVI-----------N-NFMK-

--RNS-IDSILMD-ILNGEI-PEYQR-S---V-IPPLFRVHYAREINE-AFRCR------

-----------V-QNLQ--------ETSR-----KMECIYITGSSQAGKTTLAKKIAEE-

--K-GLPYYISS---SGTD-FLG----EYALE-PCVILDDIRP-SSINLSELLKLLDNNT

---VSAVKSRYKNKC-L--ANCKLLIITTVLDIETFYHNVF--SE-EDEPMIQFKR----

--RCGTHLRM-NKERIY-ISRWDSLKKEDILDR---Y--MPKEDQTEQDVINYVSETMPF

LKESEKMHGFEII---------------

>pCRESS7|SCG87263.1

KKE--SDS-DS--KRRIEIVS--RLTDDLFD-------INTIPDII------KKHSCIEK

YAY-IIHDKDVYTEGTLK--PPHIHLYLHFNSPQHIPQIAKWFNIG-----KQ-FLEVCH

GR-EIDVLAYLVHLTPSSAG-KYQYNPLEIVSN--FDVQEAL-C--TYS-ARRN-NTLH-

--QAD-IDLICQK-IMSGEI-CEYNKVQ---M-IGPQILFDSGYKIER-AFKIQ------

-----------Q-ETFE--------ENNR-----DTLAIYIEGPAQVGKTTFAKTIAKD-

--K-SFAYYISS---SSND-LLG----DYKQE-PVVILDDLRP-DSIGISDLLKLLDNHT

---ASTFKSRYKNKY-L---NCRIIIITSTLEISRFFEEVA--AG-KAEPIEQLKR----

--RCRIHIRM-NEKKIY-ISCWDAAKKDDIPKF---Y--HVDKELSRKEINKFVDDIIP-

--IGRRPNNGEII---------------

>pCRESS7|YP_006961027.1

---------MK--LKICEIVI--NKT--LIT-------KTKIETIL-----EAKTKAIQN

YAY-ILHDKDTYQNGDIK--APHWHIYLRFNYAYDTKHISQWFNTQ-----EN-FVSKIK

GR-FSDALMYMIH--ANRSD-KHQYDEKEVVSN--FDWKSEA-Q--QDI-FNRK-YKMD-

---AR-LKEIIDK-IESGEW-KRYDLIN---K-INGYENNIYYSAIKK-AFERR------

-----------I-DFLE--------EMKR-----EMECVFITGMSGSGKTTLAKKIAED-

--N-GYKAYVSS---ASND-VLD----NYKGQ-ECIILDDLRS-YCLVLSDLLKMLDNNT

---ASSVKSRYKNKV-L---ECKLIIITTVKSIDDFFDDIF--NK--DESITQLKR----

--RCKFHIKI-DSKYIY-TSVWNDVDKKNLLNE---F--QIK-RLSEKEEKDKIKKVLKT

DL--------------------------

>pCRESS7|YP_003617079.1

---------MK--LKICELVI--NQK--LIT-------KTKIETIL-----ETKKKAIQN

YAY-ILHDKDIYQNGDIK--APHWHIYLRFNYAQDTKHISQWFNTQ-----EN-FVSKIK

GR-FSDALMYMIH--SNRFD-KHQYDEKEVVSN--FDWKSEA-Q--QDI-FNRK-YKMD-

---AR-LKEIIDK-IESGEW-KRYDLIT---K-INGYENNIYYSAIKK-AFERR------

-----------I-DFLE--------EMER-----KMECVFITGMSSSGKTTLAKKIAED-

--N-GYKAYVSS---GSND-ILD----NYKGQ-ECIILDDLRS-YCLGLSDLIKMLDNNT

---ASSVKSRYKNKV-L---ECKLIIITTVKSIDDFFDDIF--KD-KDESIIQLKR----

--RCTYHIKI-DSKYIY-TSVWNDVKKKDLLDE---F--QIK-ELTEQEEKEKIKKALKI

DL--------------------------

>pCRESS7|ABC65805.1

---------MK--LKICELVI--NSD--KIN-------KTKIENIL-----ELKKKAIQN

YAY-ILHDKDIYQNGDLK--KPHWHIYLRFNYAHDTKHISQWFNTQ-----DN-FVSKIK

GR-FSDALMYMIH--ANRSD-KHQYDEKAVVSK--FDWKSEA-Q--QDI-FLRE-YKID-

---SR-LEEILSK-IQSGEI-KECNSTN---H-ISIIENNIYSSAIEE-AVKYR------

-----------N-NTLK--------GMDR-----QMECVFITGLSGCGKTTLAKKIAKN-

--K-KYQTYISS---GSND-VLD----DYRGE-ECIILDDLRS-NCLGLSDLLKMLDNNT

---SSSVKSRYKNKV-L---ECKLIIITTVKSIDDFFEDIF--KK--DESIIQLKR----

--RCKLHIKI-DSKYIY-SSIWNPLEMKNLLNE---F--QLK-TLSKKEAKEFIKQITNT

D---------------------------

>pCRESS7|WP_011161011.1

---------MK--LRICELVI--NSN--LIN-------QSKIENIL-----EAKKNAIQN

YAF-ILHDKDIYQNGDLK--TPHWHIYLRFNNAYDVKHIAQWFNTE-----EN-FVSKIK

GR-FSDALMYMIH--ANRTD-KHQYDEREVVSD--FDWKSEA-Q--QDI-FLRK-YKID-

---TR-LKDILTK-IHSGEI-KEYNITN---Y-ISIIEHNIYSAAIEK-AFKYR------

-----------T-NTLK--------GIER-----NMECVFITGMSGSGKTTLAKKIAKD-

--K-NYQTYISS---GSND-VLD----DYQGQ-ECIILDDLRS-NCLGLTDLLKMLDNNT

---SYSVKSRYKNKV-L---ECKLIIITTVKSIDDFFEDIF--KK--DESIIQLKR----

--RCKLHISL-DSKYIS-YSMWNPVKMENLLNK---F--QIK-ALSEKEQKEFIKKITNI

DL--------------------------

>pCRESS7|ABC65794.1

---------MK--LRICELVI--NAN--KIT-------KSKIENIL-----ELKKKAIQN

YAY-ILHDKDTYQNGDLK--SPHYHIYLRFNYAYDTKHIAQWFNTQ-----DN-FVSKIK

GR-FSDALMYMTH--ANSSD-KHQYDEKAVVSD--FDWKSEI-Q--KTN-LNKQ-FKMN-

--YAR-LKEINSQ-IISGEI-KEYNIDE---R-INVDEYYVYSAAIEK-AFKYR------

-----------V-ITLK--------RIKR-----QMECVFITGQSGSGKSTLAKKIAKD-

--N-KYNAYISS---GSND-ILD----DYRGE-ECIILDDLRS-NCLGLSDLLKMLDNNT

---ASSVKSRYKNKV-L---ECKLIIITTVKSIDDFFEDIF--RK--DETIIQLKR----

--RCTYYIEI-DSKYIY-YSYWDSIQNKNLLNE---F--KIK-SLSKQEARLKFKKSLKI

NL--------------------------

>pCRESS7|YP_001966814.1

---------MK--RRICELVI--KAD--LIK-------QTEIEKVL-----ESKKKVIQS

YAF-ILHDNDKYLNGDYK--IPHWHIMLRFHQSQEFKYIAKWFNTT-----EN-FVSQIK

GR-FTDALLYLTH--ANRAD-KHQYQNHQVVSN--FDWESEA-N--QDT-FMRK-YKLD-

---TR-LVDILNK-INSGEI-KEYNITN---H-ITIIENNIYSAAIEK-AFKYR------

-----------N-SKLR--------EMDK-----KMECVFITGQSGSGKTTLAKQISKN-

--K-NYTPFISS---GSND-VLD----GYKGQ-ECVILDDLRA-DCFGVSDLLKMLDNNT

---ASSVKSRYRNKY-L---ECNLIIITTTKTLNAFFDTVF--NKSDDEDVKQLKR----

--RCRIHIKL-DLKNIT-YSIYNPNTSERIREI---F--KTK-EQSKEEKKEFIKSTLNV

EL--------------------------

>pCRESS7|KXT29039.1

-----MNPGFR--LRRCEIVI--KED--LIK-------KDFIDKVL-----K-KKKIIKK

FAY-ILHNRDVLEDGSLK--NPHYHIVLVLKTPYDVEYIASWFKTS-----SN-FVEKIK

GN-MSDILNYLTH--KNDLS-KFQYEDYEVVSN--FNWKKTR-D--EGS-I-KK-YRLD-

---KR-LKDLLTK-IMNGEI-KEYNISK---K-ITVYENNIYATALER-AFKFR------

-----------T-NFLK--------GVKR-----NMDCIFITGKSGSGKTTYAKYLADQ-

--K-GYSIYVSS---GSND-ILD----DYQSQ-ECIILDDLRP-ECLGLSDLLKMLDNNT

---ASTVKSRYKNKV-L---ECNLMIITTTIPIKKFFDLVF-YKKEKKETIVQLQR----

--RCKVHIRM-DKKNIY-YSTFNSIKDCKILKE---F--KIK-ELNEQEQLEKIIEITG-

--SDEKGKIKKIKKLEKHKLL-------

>pCRESS7|ODR34583.1

------MP-VK--LKMCDIVS--DTT--HLS-------LETIQAV------TGDKACIRH

YAY-ILHDKDKNKDGTDA--TPHYHVFLRFEDTQDTKYVAKWFGIA-----EN-FVGKIN

GK-WTDALLYLTH--ENAPS-KFQYPETDVVSN--YDWKKEK-N--AKL---TS-QSLK-

---AR-EAEIVSL-IANGTI-KKYNYNE---Y-ITPVEYVRLNASIKA-AMNYR------

-----------A-DMLS-------HNHNR-----QLEVVYIVGGSGCGKSTYAKRLAEE-

--K-GLSCYVSS---GSND-VLD----DYGGQ-DCLILDDLRP-SCLGLSDLLKLLDNNT

---STSVKSRYKNKI-L---ECSLIIITTVKEIDEFFSKVF--EH-EDEPLKQLKR----

--RCRTMIRL-SADTIE-ISVYNDTSDRPIAEL---Y--KTE-PLTQAELTAKVEKDFCI

SGVVSGDGFMEIV---------------

>pCRESS7|CCZ93342.1

AEK--RTP-KT--WKTCEIIQ--QLE--YMS-------AEDVGS-------GLDHNAIKD

YAY-ILHDKDVNDDGSPK--AAHWHIYIRFKDSTPTDSICKWFGIT-----SN-YIGRIQ

GR-FADALAYATH--RNVPE-KYQYLDEEVKSN--FDFVKER-D--TAR---SK-EADK-

---QR-KAEISDL-IISGVI-REYNYTD---Y-ITIQEYDRFRKSIDN-AFKYR------

-----------L-DKIK--------GENR-----DMEVIYIFGDSSCGKTTYAKELAAQ-

--N-EYSCYVSS---GGED-MLD----DYKGQ-DCVILDDLRA-NDINFSSLLKLLDNHT

---QSMVRARYHNKF-L---ECKLMIITTSKSMEELFRELP--GS-DNEDITQLRR----

--RCKLYIKM-TPLTMT-IRMWQPESLKPVHGR---Y--AAR-DKSLDEAKAYVNNVLFF

PKMAKGEGFRELTDQMKLPL--------

>pCRESS7|CCY61699.1

--M--GEP-SS--WRTCEIVQ--QLE--YMS-------QEDVEA-------GLDHNAVQD

YAY-ILHDKDVHEDGTPV--APHWHIMIRFKRPVQTESLCKWFGIK-----SN-MIGYIL

GT-FGDAVAYLTH--RNKPE-KYQYLDEEIKSN--YDFKVEV-E--KAL---SK-KKAS-

---QR-KEEIIEL-IRSGIV-REYNYTE---Y-ITALEYDKFKRAIDN-AFTYR------

-----------R-DTLK--------SLDR-----HMNVIYIYGGSGTGKTTYAKQLAIN-

--K-GLSCYISS---GSND-PLD----GYKGQ-DCLILDDIRP-GDFLLSDFLKILDNNT

---QSTVKSRYKNKL-L---ECQYLIVTTSFDIPVFFDLLL--DS-EGESVKQMER----

--RCTLKIQM-NTSTMT-TYVYQPVSGKPVSEV---Y--NKR-DLTDEECQAYVDSLLLP

GKL----GFKADVEQWNQEEF-------

>pCRESS7|WP_019282500.1

MDS------IT--MRQCELVS--NIE--HLD-------LEYIKEKL-E--SATNGKSVTD

YAY-IVHDKDTYEKGDLK--APHVHLMMKFKSPQKVHCIAKWFKVK-----DN-NINKIK

SK-WVSALRYLIH--ANHPE-KHQYNVDEVIAN--FDYSEEK-D--KIN-T-HN-NKKK-

---QR-KEEIVNN-ISKGEW-KALDLTD---N-ITELEFVEFSADINK-ALNYR------

-----------Q-TFLQL------NNKGR-----DMNVIYISGGSGSGKTTLAREYAEK-

--K-GHSIYVSD---GGKN-PMD----NYMGQ-DCIILDDFRP-DVMGFSDLLKLLDNHT

---SSMVNARYYNKF-M--GECKLLIITTIDDLPDFFKKMQ--DT-KGEPIKQFER----

--RCKTKMLV-DSDTVR-FYSY--AIDKPLKDRAEAF--GVSDQISKSEIKVV-------

-------GF-------------------

>pCRESS7|WP_002578150.1

MSK--TDP--C--RVICEFQN--QIE--YTS-------ESSFKNIV-N--NLYKRGIILD

YAY-IVHDKDTYTEGNLK--KTHIHGMLRLNNSYKFSTIANWFDVT-----AQ-RIRKIE

TS-YAAACAYLIH--RNNPE-KFQYDPSMVISS--FDYNAEK-E--KAQ-LTNE-NHMK-

---KL-KKRILEE-VEAGTL-RGYNFHE---N-YAFSDRVALRSYLNN-AIEII------

-----------K-TKLN-------SNKER-----DLEVIYIHGSSGAGKTTYAKMTAKA-

--R-GLIYATSG---EDRD-PVE----TYDSH-PCMILDELRP-SSMKLTSFLKLVDNNT

---ESMAGARYHGKA-F--IECKLIIITSILPIEEFFKNLQ--AN-DNETAIQIKR----

--RCKTMFDM-DRDNIE-IYEWDDCNLEPIPTL---Y--HIE-PKSIEELRAKACEVAGI

DLVG--IPFEAVYTKIKHNPW-EHKAIP

>pCRESS7|CUN62864.1

MSE--TTP------TLCEVQT--QIP--FLP-------KEKMLSII-A--EHTTAGHIKE

WAY-ILHDKDIKEDGTPK--EPHWHIELRLTRGRRLTDIASWFGLP-----TS-CIQTSK

SGRYEPMLQYLIH--ENSSE-KYQYAESEVVAN--FNYSQAK-K--NAK-IDGD-KRIS-

---E-----IVEL-IANGTI-REFNIDE---F-VTVREYDKYRSHIKN-ALEYR------

-----------S-IILE-------KQNTR-----NMEVIYIYGKGGTGKSSFAEAIAKE-

--R-GFSFKRSA---SERD-PLA----TYKGQ-DSFALDDVRG-NTFEFQDWQGVLDNFQ

---DRPGSSRFHDKH-F--TECKLLFITTTDSAEDFWKEMS--AKRPNEDSHQFFR----

--RIKTVIHM-TGDEIL-CKRYDEETHSDTYSR---F--DVH-SETDEEQKEKLAHSLGV

DKIGEPIPFETVYEQVKHDPW-EHKAVP

>pCRESS7|WP_028509833.1

------MPDKS--WRTCEITQ--DCC--GWT-------PENVHKFI------DGWSSVKD

YAY-ILHDKDKKDDTTPR--EPHIHLMLRFSCAVHTSNILARAKIT-----EN-RIQKMK

S--WSAALNYLTH--RDEHKWKHVYDTAEVISN--FDWQLES-E--SAH-QAKQ-LRAD-

--KGR-EKEIVEA-IASGEI-RLFNLSE---H-ITSYEENLYSKAIKT-AFNRR------

-----------T-RDLK-------LKNER-----NMEVIFISGESGVGKDTFAREWCKD-

--K-GLS-YFTT---GNNDSPFD----DYMGQ-DVIIWSDARD-DVYKPAQIHTMLDNHW

---SSTQKARFVDQV-L---NCQYFIITSVKPLNEWYKNFY--SK-EGEDIKQLYR----

--RIKTWYDM-NDKDIS-CRIYDQNLNE-----IYGH--KSE-YLDNAEKRVEFARDMRS

DKEKNNANFFDVYSCLDAIHD-HGKEIR

>pCRESS8|WP_033683822.1

--------MVK--ARNVMFVQ--QIE--YF---QSSNLQDIIEYMT-N--ILK----PHR

FAG-ILHDKDIGQDGNLV--APHIHLVLQFESARSLNNLAKLTKQP-----IQ-CFEQWR

GS-VNNAYSYLVHHTESNQD-KYQYSPKEVIAN--FDYLLLL-D--TIE-RNVT-KRYE-

INTMI-IDNLLDL-LYTGNI-TKSEIEQ---R-LTGSQYAKARQKIET-VYLKR------

-----------L-ETQAELWRQEMIDKNE-----IVTIIWLFGKAGTGKTRLARQYAEQ-

--Y-DLNYFITG---SIRD-PFQ----QYNLE-HVIILDELRP-HQFDYSDLLKMFDPYN

--VKAMASSRYFDKP-L---LANIYIITSPYSPYDFFLELT-KKRKHIDSWGQLMR----

--RLSLVVEV-KKEHLQ-FYKYEPMDQMKLPNP---F---KN-Q-TDREETI--------

--DKSYQLFLEL-KKE------RNE---

>pCRESS8|EFO53527.1

--------MVK--ARNIMFVQ--QIE--YF---QSSNLQDIIEYMT-N--TLK----PIR

FAG-ILHDKDIGQDGNLV--TPHIHLVLQFESARSLNNLAKLTKQP-----IQ-CFEQWR

GS-VNNAYSYLVHHTESNQD-KYQYSPKEVIAN--FDYLLLL-D--TIE-KNVT-KRYE-

INTMI-IDNLLDL-LYIGDI-TKSEIEQ---R-LTGSQYAKARQKIET-VYLKR------

-----------L-ETQAELWRQEMIDKNE-----IVTIIWLFGKAGTGKTRLARQYAEQ-

--Y-DLNYFITG---SIRD-PFQ----QYNLE-HVIILDELRP-HQFDYSDLLKMFDPYN

--VKAMASSRYFDKP-L---LANIYIITSPYSPYDFFLELT-KKRKHIDSWGQLMR----

--RLSLVVEV-KKEYLQ-FYKYEPMDQMKLPNP---F---KN-Q-TDREETI--------

--DKSYQLFLEL-KRK------GMNKCK

>pCRESS8|WP_049523992.1

--------MVK--ARNVMFVQ--QID--YF---KTPNIQAIIKELT-D--VLK----PIR

FAG-ILHDKDIGSDGTAV--APHIHLILQFESARSLNNLAKLTSQP-----IQ-CFEQWR

GS-INNAYSYLVHHTSSDQD-KYQYSPKEVIAN--FDYLFLL-E--TIE-KNVR-KRNE-

VNSMI-IDNLLDL-LYAGEI-TKSEIEE---R-LTGSQYAKAKHKIDT-VHLKR------

-----------L-EIQAESWRHEMKNKNA-----IVTIIWLFGKAGTGKTRLARQYAEQ-

--F-DPNYFITG---SIRD-PFQ----QYNLE-HVVILDELRP-HQFDYSDLLKMFDPYN

--DRAMASSRYFDKP-L---LANVYIITSPYSPYNFFLELT-KKKQHIDSWGQLMR----

--RLTLVIEI-SKEYLQ-FYKYSPLDQMKLSNP---F---KD-E-NEKTNLI--------

--NNSYELFLEL-EEN------NEKHI-

>pCRESS8|WP_024410839.1

--------MVK--ARNVMFVQ--QIE--YL---NSSNLQGIIHDIT-E--VLK----PIR

FAG-ILHDKDKGQNGDTV--EPHIHLVLQFESARSLNNLAKLTEQP-----IQ-CFEQWR

GS-VNNAYSYLVHHTESNQD-KYQYSPKEVIAN--FDYLLLL-D--TIE-KNVT-KRHE-

VNTMI-IDNLLDL-LYTGEI-TKSEIEQ---R-LTGSQYAKAKAKIEA-VHLKQ------

-----------L-ENKSCEWQKEMREKNE-----KSIVIWLFGKAGTGKTRLARRYAKQ-

--F-SETYFITG---SIRD-PFQ----HYQME-PVIILDELRP-HQLDYSDLLKMFDPYN

--VKAIASSRYFDKP-L---LANVFIATSPYSPYDFFLELR-KGRHDVDNYQQLMR----

--RLTLVLEV-SKQFIE-MYSYDKHLGLKLPNP---Y---YQ-E--EVADYN--------

--TERLELFKNL-KGM------QDEKI-

>pCRESS8|CYX46115.1

---------MK--ARNVMFTQ--QVS--YL---TSGNLTDIIEEIR-E--KMK----PKR

IAG-IIHDKDLDENSQLV--KPHVHIVLQFDSARSLNNIAKLFNQP-----VQ-CLEAWR

GS-VNNAYSYLVHHTKSASN-KHIYDPKEVVAD--FDYIELL-E--KIR-QNVT-KQSK-

INSVI-INNLLDL-LYEGAI-SKQEIES---R-LSGSQLAKAQSKIET-IHLKR------

-----------L-EQNAREWQKEMRERNE-----KSTVIWLFGKAGTGKTRLAKHYARQ-

--Y-SDTYFITG---STRD-PFQ----HYQME-PVVILDELRP-HQFDYSDFLKLFDPYN

--EQVMASSRYFDKP-L---MANTYIVTTPYSPYDFFLELR-KSRHQVDSYQQLMR----

--RLTLVLEV-SKQFIE-MHTYDTYLQLKLPNP---F---YR-E--EKADYG--------

--QERFNLFKEL-EGK------QDETS-

>pCRESS8|CYW87437.1

---------MK--ARNVMFTQ--QVS--YL---TSGNLTDIIKEIR-E--KMK----PKK

IAG-IIHDKDLDENGQLV--KPHIHIVLQFDSARSLNNIAKLLNQP-----VQ-CLEAWR

GS-VNNAYSYLVHHTQSASN-KHIYDPKEVVAD--FDYIELL-E--KIR-QNVT-KQSK-

INSVI-INNLLDL-LYEGAI-SKKEIEQ---K-LSGSQYAKAKAKIEA-VYLKQ------

-----------L-ENRASEWQKEMREKNE-----KSIVIWLFGKAGTGKTRLARHYAKQ-

--F-NEIYFITG---SIRD-PFQ----NYQLE-PVIILDELRP-HQFDYTDLLKLFDPYN

--EQVMASSRYFDKP-I---MANIFVITSPYSPYDFFLELR-KSRHQVDSYQQLMR----

--RLTLVLEV-SKQFIE-MYNYDKHLGLKLPNP---Y---YQ-E--EAQNHN--------

--TNQFELFKDL-KGI------QNEKI-

>pCRESS8|WP_051448806.1

-----------------MYQQ--QLS--HLP-SKRKTLNSIVKSLV-E--RLE----PLK

IAA-IIHDKDVNEEGDSI--EKHVHVVLQFENQRSLERLAKVLNEP----QIS-SFQQWR

GN-VNNAYSYLVHQTKEAKG-KYQYQLEEVKAN--FDYPALM-S--DIT-KKIE-QKIK-

MKSEI-IKSLLDQ-LGAGEL-SKDEVIL---N-LTGSQFAKAKKQIND-VYQQV------

-----------Q-EQKSKFWLEEQKSKNE-----PITVIWIYGSSGTGKTALAKKYADE-

--Q-NVKYFITG---SSRD-SFQ----HYDGE-HLVILDELRP-TTFNYDDLLKMLDPFG

--ENPKAPSRFFDKS-L---MIDIFIITSPYSPKQFYDEIF-RHKKTVDSFGQLQR----

--RITFVQFI-TQDYFE-MQNYNDLEKKRQKNY---L---LE-KLKKKYNTN--------

--RQTYDKFNQL-K--------------

>pCRESS8|WP_050444210.1

------MSTTK--AVNVMYEQ--QLR--YLP-TKRKTLNSIIKLLI-E--CLK----PKK

IAG-IIHDKDINDKGEVI--EKHVHVVLQFQHARSLKNLARLLKEP----QVS-AFQKWH

GN-VNNAYSYLVHRTADAQE-KYPYSLEEVKAN--FDYPQLM-K--NIS-KKIT-GSNR-

QRNEI-IKQLLDR-LGAGEL-TREEVIS---N-LTGSQFAKAKKNIQD-VHEQV------

-----------Q-ADKAKIWLEKRKEKGE-----STTVIWIYGQSGAGKTLLAKKYATK-

--K-EQDYFITG---SSKD-SFQ----HYQGE-HIVILDELRP-KTFPYDDLLKMLDPFG

--ETPKAPSRFFDKS-L---MVDVFIITSPYSPKQFYDEIF-RRKKTIDSFKQLQR----

--RINYVQFM-SSDYFE-MQEYDVYSGSRKKNT---L---ID-NRNSPAPLD--------

--KKIHQEFSAF-DDK------KGIEIK

>pCRESS8|WP_010817837.1

------MSTTK--AVNVMYEQ--QLH--HLP-TKRRTLNSIIKQLT-E--CLK----PEK

IAG-IIHDKDVNDEGVPV--EKHVHVVLQFQHARSLENLARLIKEP----QVS-AFQKWH

GN-INNAYSYLVHRTTDAQE-KYLYSLEEVKAN--FDYPELM-K--SIS-KKIN-KSNK-

QRNEI-IKQLLDR-LGAGEL-TREEVIS---N-LTGSQFAKAKKHIQD-VHEQV------

-----------Q-ADKAKIWLEKRKEKGE-----PITVIWIYGQSETGKSLLAKKYAAK-

--K-AKNYFITG---SSKD-SFQ----YYQGE-HIVILDELRP-KTFPYDDLLKMLDPFG

--ENPKAPSRFFDKS-L---MVDVFIITSPYSPKQFYDEIF-KRKKTIDSFKQLQR----

--RISYVQFM-SSDYFE-MQEYDIYSGSRKKNT---L---IN-GTKHPATLD--------

--KKIHQEFSDF-DDK------EGSETN

>pCRESS8|WP_016226904.1

MSD--ENCRVR--SKNMMYTQ--QIR--HLPVS-----TDQLVSRV-K--KMA----PKR

YAL-IVHDSDVNEQGEPA--EDHVHVMLSFENARSINSIAKELGDE-----PQ-SIEMWK

GK-AENGYSYLIHATKDSGN-KYQYPPSRVIAN--FNYQEEI-K--RIT-EEVE-RSRQ-

TASKI----LLDS-LYKGEI-SKEELEK---R-LSGSQYGRMRRQIED-VWCKH------

-----------L-QFQAAKWREEMKKSGK-----RIKVIWISGEAGTGKTSLAKEYAEK-

--S-GRPYFITG---SSRD-IFQ----NYSGE-HTIILDEFRA-DMMKYPDLLRILDPFG

--SQVMAPSRYNDKP-L---ACDLILITSPYNPVEFYRQLF-SRA-FVDSLEQLLR----

--RISLTIEM-DASHIR-AVEYDKEAEAEKENR---Y---SR-H-YREQDST--------

--SNPKELFESM-EAPC-----EPGSLA

>pCRESS8|SCH55298.1

MTG---SAPRK--TRAMMYEQ--QLC--HLP-S-----VDEMYRRI-E--ALA----PKR

YAG-IVHDHDITDAGRPA--ADHLHVMMEFANPRSVRSVAKSLGDK-----AE-RLEAWK

AG-TENGFSYLCHRTDGARS-KYQYDPSLVRAN--FDYPAAL-A--SIE-SRVS-KARS-

HSIKI----LLDD-LLEGRI-DREGLIS---Q-LSGSEYARAKRQIED-VYARR------

-----------L-QVSAAEWRAKMRDEGR-----RVQTIWIFGPAGTGKSSLAKQYAQS-

--K-GEPFFVSG---STRD-VFQ----GYAGQ-HTIILDELRP-SSIPYADLLRVTDPYA

I-HEVMAPARYADKA-I---AADLIIVTTPYNPMEFYCEQV-RGATDIDGFGQLER----

--RLSLVVEM-QQKEIC-LSEFRVELGTSRPNP---Y---SS-F-AR-GSSG--------

--GDSAHLYEAL----------DSGTVT

>pCRESS8|WP_042900192.1

--------MPK--QRAIMYTQ--QMR--LA---ILSDWKKEIDRIV-K--LLE----PLL

WAG-ILHDKDVNEDGETV--EPHIHLMMYFKHARSPHSIAWEINERNGK-QIE-RLEFFK

-H-PNNGFSYLVHQTKDAQN-KYQYPISEVISN--FDFAKKL-E--NIR-KQVE-RNQS-

KKGEL-IREYLDM-LYDGLL-TLEEIES---E-LTGSQYAKASTRLKA-VAEKR------

-----------Q-ERLGREFLNRMKYEQK-----TKQVVYIYGESGLGKTRLAKTYAEN-

--K-NTSYFVTG---SSRD-PFQ----SYQNQ-ETIIIDELRP-DSFRYDDLLKILDPYN

--FDVFLPSRYIDKA-L---TAELIFITSPYSPKELYDNFQ-TSKR-IDRFDQLER----

--RIQTAILV-EKDNIF-YTHYNYESREFFTNP---F---ES-Q----------------

----------------------------

>pCRESS8|WP_050492321.1

MSST-NKKIPK--QRAIMYTQ--QMR--LA---ILSDWKKEIDRIV-K--LLE----PLL

WAG-ILHDKDVNEDGETV--EPHIHLMMYFKHARSPHSIAWEINERNGK-QIE-RLEFFK

-H-PNNGFSYLVHQTKDAQN-KYQYPISEVISN--FDFAKKL-E--NIR-KQVE-RNQS-

KKGEL-IREYLDM-LYDGLL-TLEEIES---E-LTGSQYAKASTRLKA-VAEKR------

-----------Q-ERLGREFLNRMKYEQK-----TKQVVYIYGESGLGKTRLAKTYAEN-

--K-NTSYFVTG---SSRD-PFQ----SYQNQ-ETIIIDELRP-DSFRYDDLLKILDPYN

--FDVFLPSRYIDKA-L---TAELIFITSPYSPKELYDNFQ-TSKR-IDRFDQLER----

--RIQTAILV-EKDNIF-YTHYNYESREFFTNP---F---ES-Q----PIHR--------

--NSFFTQFETT-RKE------------

>pCRESS8|KXA58447.1

MSST-NKKIPK--QRAIMYTQ--QMR--LA---ILSDWKKEIDRIV-K--LLE----PLL

WAG-ILHDKDVNEDGETV--EPHIHLMMYFKHARSPHSIAWEINERNGK-QIE-RLEFFK

-H-LNNGFSYLVHQTKDAQN-KYQYPISEVISN--FDFAKKL-E--NIR-KQVE-RNQS-

KKGEL-IREYLDM-LYDGLL-TLEEIES---E-LTGSQYAKASTRLKA-VAEKR------

-----------Q-ERLGREFLNRMKYEQK-----TKQVVYIYGESGLGKTRLAKTYAEN-

--K-NTSYFVTG---SSRD-PFQ----SYQNQ-ETIIIDELRP-DSFRYDDLLKILDPYN

--FDVFLPSRYIDKA-L---TAELIFITSPYSPKELYDNFQ-TSKR-IDRFDQLER----

--RIQTAILV-EKDNIF-YTHYNYESREFFTNP---F---ES-Q----SIHR--------

--NSFFTQFETT-RKE------QQ----

>pCRESS8|WP_000093566.1

MSST-NKKIPK--QRAIMYTQ--QMR--LA---ILSDWKEEIDRIV-K--LLE----PLL

WAG-ILHDKDVNEDGETV--EPHIHLMMYFKHARSPHSIAWEINERNGK-QIE-RLEFFK

-H-PNNGFSYLVHQTKDAQN-KYQYPISEVISN--FDFAKKL-E--NIR-KQVE-RNQS-

KKGEL-IREYLDM-LYDGLL-TLEEIES---E-LTGSQYAKASTRLKA-VAEKR------

-----------Q-ERLGREFLNRMKYEQK-----TKQVVYIYGESGLGKTRLAKTYAEN-

--K-NTSYFVTG---SSRD-PFQ----SYQNQ-ETIIIDELRP-DSFRYDDLLKILDPYN

--FDVFLPSRYIDKA-L---TAELIFITSPYSPKELYDNFQ-TSKR-IDRFDQLER----

--RIQTAILV-EKDNIF-YTHYNNESREFFTNP---F---ES-Q----SIHR--------

--NSFFTQFETT-RKE------QQ----

>pCRESS8|EEJ43069.1

-------MPKR--SRTFMYTQ--QLQ--HLPFD-----VAAFQSRL-E--NIN----VAE

YAF-IIHDQD-TVDGHPV--TSHIHAVLRYQNARSVDSVAKQVSDK-----AQ-YIEIWN

GN-YANAYAYLVHKTDGASR-KYQYPLDSVTSN--FDFKERI-E--SFS-ST------G-

NRNQLAIKYILDE-IVSGKI-TKEEAYQ---L-LPGSLLSKSVNNINS-AFQIR------

-----------Q-VLEAERWRKDKKASGE-----RIHVIWIFGVAGTGKTRFAIDFFKK-

--L-NMKYFKSG---SSKD-PFQ----GYSGQ-HGIILDDLRP-NGLSYADLLRFLDPWN

--LEAMAASRYFDKG-L---QADYVIITSPLGPVEFYDSLF-TDEM--DAFDQLTR----

--RLETVLYF-DRQSIM-ECELMSLNYKKVENR---W---YV-P-EEEQSE---------

--HFNLSEIQNL-DEEETN---DE----

>pCRESS8|WP_004900270.1

ADST-AEVGKK--YRQFMFVQ--QLR--YLGYD-----LTALNERI-D--IIK----PLE

FAY-VVHDKDLTEDGELI--EPHLHLALRFENPVSLKRLAESLETE-----PQ-YIAQWK

GA-ANNLYSYLIHRTETASD-RYQYEVNEVVAN--FDFPGKI-E--RIE-QAIQ-GRKG-

KRGTL-LNETLDA-LLEGNI-TLFDAFE---I-LPGRIAGKHRKKLES-AYQTR------

-----------M-ELNAKVWLENKKALGK-----PIQVFWFYGPAGVGKTRYAKQYLSR-

--IDDGEIFISG---SNRD-PFQ----NYSKSAHKVILDDIRP-NTFSYEELLRIFDPWN

--MEVSVGSRYSDKN-L---QVDIFIITSPLAPDVFVDTLQ-VFEL-DDNFDQLLR----

--RLTTVMYF-DDQYIYPAVPYLGMGKEKVINQ---W---SG-H-QGEQEHGLQ------

--RKTIEKFLEM-QEQDKL---DGKGIR

>pCRESS8|WP_062359070.1

-------MNKK--YRTFGYTQ--QLS--HLN-R-----LEDFPRLM-E--EAG----VEQ

YAY-IVHDKDRTADGVLK--ASHVHVVMKFENPRSLQAVSKIFHDS-----PQ-YVEKTR

NG-YNNMLAYLTHRTKGATM-KHQYDPDAVAAN--FDYSDAL-E--KIT-AKVE-QRDS-

KT----LDELLER-LMDYSI-TYEEAVG---E-LTASEFSRYHQRLKV-TAEYG------

-----------R-QQYAKRWIKEHEDNHK-----PIEVIWITGPAGSGKTVEAKKIARE-

--KSDKDFYVAV---GVRD-PFQ----LYTDE-NVIILDDLRA-KTFDYNTLLQLLDPFN

---STMADARYSNKT-L---IADTIIITSVYSPLDFYHMLV-PEIYDVDTYKQLER----

--RISQIIRK-RC-----------------------------------------------

----------------------------

>pCRESS8|WP_008469878.1

-------MKKR--FRQFMYTQ--DLD--HLP-N-----IKSLQSLI-K--TEP----LEE

WAY-IIHDKDKNEDNTPI--RKHLHLVLKYKNPQTLHHISNLLTDK-----DQ-YFEIWN

GR-INNAYSYLIHATNEARE-KYQYSPSEVTAS--FNFEKRI-S--KIK-DSIS-NS---

--NNK-VQTYIQR-YAEEEI-SYKQLID---Q-IGLINIAKNKRLIDT-IQNLL------

-----------I-SKHHQEWLHNFE--GK-----KMICLWLWGDAGVGKTTYAKKLLSQ-

-----ENYIVLG---SSND-YFQ----NYTDE-SFAIINDLRP-EDWKYADLLRLLDPYE

--HNKMAPSRYHDKE-L---NLEMIIITTPYSPQSFYHNSR-IFNPKIDSFEQLKR----

--RIF-PIHI-TPEFIRKEK---------------------S-YGYFAN-----------

----------------------------

>pCRESS8|WP_046324376.1

M------AEIR--ARQFMYMQ--DFA--HLK-D-----FTELTNIL-T--KAN----IQE

WAY-IIHDQDLDQGNRLI--RKHLHVVLKYANPQILSHVVRLFNDK-----PQ-YLEVWQ

GR-ISNAYSYLIHATLEAKD-KYQYDPNSVVAS--FDFPARI-T--EIQ-SSVN-QSRL-

--SKV-VANFLTQ-YANEEI-DYQELAD---I-IGLAEVAKRKSVIDN-ITKLI------

-----------A-DKKHEEWLHEYH--DR-----KAETIWLWGEAGVGKTRYANKLVRG-

-----EKVAILG---SSRD-YFQ----DYHGE-HYVILNDLRP-NDFRYADLLRLLDPYE

--HDKVAPRRYHDVK-L---NVEMLIITTPYSPFEFYQHVK-IADEKTDTFEQLKR----

--RVH-AIHI-TPKFIAEVF-----------------------QDNESEFEELF------

----------------------------

>pCRESS8|WP_049150683.1

------MQELR--ARQWMYVQ--YFK--YLP-K-----INNLTEIL-T--KDN----CQE

WAY-IVHDKDTKEDGSLI--SPHVHVLIKYSNPQTLKHVANLFKDK-----PQ-YFDIWK

GR-INNGYSYLIHSTSEAKG-KYKYSPNEVVAS--FDFSKRI-E--KIQ-RQVL-NKKI-

--SNN-SNLFIEK-YAEGEI-SYNDLEN---I-IGVVQVARHKTIIDH-INQIN------

-----------A-SNKHKEWLKSFK--GK-----PMETHWLWGAAGVGKTRYAKWLAKN-

-----DKVAILG---SSRD-YFQ----EYHGE-HIVILNDLRP-NDFNYGDLLRLLDPYE

--HNKMAPRRYHDVY-L---NLEMLIITTPYSPWSFYKQCK-IDNPEVDTYKQLNR----

--RVH-AVHV-TKDFISDIV---------------------P-YEFNMNFEN--------

--GFE-----------------------

>pCRESS8|WP_014567781.1

MG-----VPRR--ARQFMYMQ--DVD--HLK-K-----EKSLKSIL-N--KSG----ALE

WSF-IKHDQDIDENGKLI--RPHYHVILKYEYPRSILSVAQIFKDQ-----TQ-YVEIWS

GR-IANAYSYLIHETEEAAT-KHHYQDNEVVAS--FDFHKRM-E--EIR-KKIK-KS---

--SKY-VMEMIER-YANNKL-TYEELAK---E-LGVMPMAKHQQLIDR-ISQVQ------

-----------E-EEAHKRWLTKMK--GK-----SMKVIWLYGAAGVGKTRMAEIMLSK-

-----HKYVILG---SSRD-YFQ----DYHGE-HYIVLNDLRP-NDFPYSDLLRLLDPYQ

--HDKAAPSRYHDKK-L---SAEEIIITTPYSPYDFYRNIS-IHDKSVDTIDQLLR----

--RVI-PIKI-TPSFFKSVL-----------------------EKKRRAKSN--------

----------------------------

>pCRESS8|WP_007125042.1

MK-----KEIR--ARQFMYVQ--DLN--HLS-K-----ESDFKQIL-N--QSG----AME

WAY-IKHDKDKDQEGKII--RPHIHAVLKYENPQKLSTIANLFNDQ-----AQ-YVDVWK

GR-IANAYSYLLHETEEAQG-KHVYKDTEVVAS--FDFPARM-K--NIR-TKIS-KS---

--PKY-ISSLINQ-YAEGKI-QYQELED---Q-IGISQLARRKKLIDQ-IDELK------

-----------A-DKEHKEWLKKFE--GK-----PMHTLWLYGEAGVGKTRYAEYLLRK-

-----KKYVILG---SSRD-YFQ----TYNGE-HFIILNDLRP-NDFNYSDLLRILDPYQ

--HDKAAPSRYRDKK-L---SVEEIIITTPYSPKDFYRTTK-IDDRQVDTVDQLLR----

--RIQ-PIHV-TPKFIKRRL-----------------------KIKRPKHTD--------

--DNA-----------------------

>pCRESS8|WP_060461663.1

MK-K-KVKEPR--ARQFMYVQ--DLD--HLK-D-----EKDLSTIL-K--QSG----ALE

WAY-IKHDKDKDQDGKTI--RPHIHVVLKYENPQKISSVASMFKDQ-----PQ-YVGVWK

GR-IANAYSYLLHETEEAQG-KHVYKASDVVAS--FDFEARM-K--SIR-AKVT-KS---

--PKY-VSSLIDQ-YAESKI-TYDELES---Q-IGISQLARRKKLVDQ-ITELR------

-----------A-EKEHEKWLKDFK--GK-----KMKVLWLYGAAGVGKTRFAEYLFRN-

-----KKYSILG---SRRD-YFQ----DYCGE-HFVILNDLRP-RDFSYSDLLRILDPYQ

--HDKSAPSRYHDKK-L---NLEEIIITTPYAPTDFYKYVF-IDDRRVDTDEQLLR----

--RIQ-PIHI-TKSFIKKRL-----------------------KTKKSKQNG--------

--NNA-----------------------

>pCRESS8|WP_013641481.1

--------MKR--FRQFMFVQ--DVE--HMN-K-----FEELPNIL-K--SESS--RLSE

WAY-IKHNHDNDKEDTKV--RDHIHVVLKYRNPQTVAHVAKLFKDK-----SN-NVQIWI

GR-INNAYSYLVHNTDNATS-KHQYSIEDVKAS--FDFKKRI-K--DIE-ENVS-LAK--

--QRN-IKEAING-FAEGDI-DYKELIE---T-LGIVNVAKNRNLIDS-IQKIR------

-----------E-QVIQHEWWNQFN--GK-----QMASLWLWGEAGVGKTTYAERILSN-

-----EKYIVLG---NSND-YFQ----YYNGE-HYIILNDLRP-GDLKYADLLRILDPYA

---IKYTFGRYHNHP-L---LAEMIIITTPYSPREFYKNTR-IANRKIDSLTQLQR----

--RIF-EIHI-TKDFILKEN---------------------K-YQRNSKEGS--------

--D-------------------------

>pCRESS8|WP_003549058.1

MK-----KAIR--ARQFMYTQ--DLD--HLP-K-----KEDLKTLL-E--KSS----AEE

WAY-ILHDKDIGKNGKTI--RPHFHVVMKFKDAKTISRVAKLFNDK-----QE-YIEVWR

NT-IGNAYSYLIHETSNAKD-KHHYDPIEVVSS--FDFETKI-K--QIR-KKVA-KLS--

--KKD-IDDLIDD-YSNEIL-TKTDLQE---K-IGVLEMAKHKTLLDH-IDDIL------

-----------A-YKKHQKFLKDFQ--GQ-----KCTTYWIYGESGIGKTKMVREILEQ-

--LHPNNFVVLG---SQRD-HFQ----EYRGE-EFIVINDLRP-RDYEYGQLLTLLDPWE

--IDKMAPARYHDKY-L---NARAIYITTPYSPMAFYNESG-IVNSLIDSFEQLNR----

--RIL-SLHL-TSDTYNQMKA-------AEAIW---K---IK-KTKSTSQTD--------

--DKSND---------------------

>pCRESS8|KRN00682.1

MK-----KAIR--ARQFMYTQ--DLN--HLP-K-----KEDLKTIL-E--KSS----AEE

WAY-ILHNKDIDKDGKTI--RPHFHVVLKFKDAKTISRIAKLFNDK-----QE-YIEVWR

NT-IGNAYSYLIHETRNAKD-KHHYSPTEVISS--FDFETRI-K--QIR-KKVA-KPS--

--KKD-IDDLIDD-YSNEIL-TKTELQE---K-IGVLEMAKHKTLLDH-IEDIL------

-----------A-YKKHQQFLQDFK--GQ-----KCTTYWIYGESGIGKTKMVREILEQ-

--LHPNNFVILG---SQRD-HFQ----EYRGE-EFIVINDLRP-RDYEYGQLLTLLDPWE

--IDKMAPARYHDKY-L---NARAVYITTPYSPIAFYQGAN-IVNGLIDSFEQLNR----

--RII-TLHL-TEDNYDEMKA-------AEAIW---K---IK-KQKNTSHTD--------

--EKNND---------------------

>pCRESS8|CDA26462.1

MK-----KAIR--ARQFMYTQ--DLE--HLP-K-----QEQLKEIL-E--KSD----AEQ

WAY-ILHDKDVNEKGEPI--RPHFHVILKFKDAKTISRIAKLFNDQ-----QQ-YIEVWH

NT-INNGYSYLIHETTNAQN-KHHYDPSEVVAS--FDFVTRI-K--QIR-EKVN-KPS--

--KHD-IENFIDD-YSNEQL-TKEGLQE---K-IGVLEMAKHKTLLDH-IEDIL------

-----------A-YKKHQQFLKDFK--GQ-----KCTTYWIWGSSGIGKTKLVREVLEE-

--LHPNNFIILG---SQRD-HFQ----EYAGQ-EFIVINDLRP-NDYDYGQLLTLLDPWE

--IDKMAPARYHDRY-L---NARSIYITTPYDPLSFYFECN-ISNQVVDSFEQLKR----

--RIV-SLKL-TEDTYSDLKK-------AEAIW---K---IK-SQKNTSHAD--------

--DKSND---------------------

>pCRESS8|CDI43023.1

MK-----KKIR--SHNFMYTQ--DLD--HLP-S-----KDELKDRL-E--KSG----AEE

WAY-ILHDKDIDENGKKV--RPHFHVMIHFRDAKTISRVSKIFNDH-----QQ-YIEAWH

SI-INNGFSYLIHETTNAKS-KYHYDPQEVVAS--FNFEDKI-N--EIR-QKVK-KPS--

--RQA-IDNFIDD-YSNEEI-TKEELQD---K-IGVLEMAKHKTLLDH-IEDIL------

-----------A-YKNHQQFLKDFK--GQ-----KCKVYWIYGVSGIGKTKLVREILEK-

--RHPEDFCILG---SQRD-HFQ----EYKGQ-GFVVINDLRP-NDYDYGQLLTLLDPWE

--IDKMAPARYHDRY-L---NARAIYITTPYDPLSFYFECN-IANQLVDSFEQLKR----

--RII-PLQL-TKNNINKVKH-------SEATW---K---IK-SQKNTSHAD--------

--DKSND---------------------

>pCRESS8|KRK41125.1

MK-----KIIR--VRSFMYTQ--DLD--HLP-K-----PEELKDRL-E--KSG----AEE

WAY-ILHDKDTDKDDKEV--RPHFHVMLHFKDAKTISRVAKVFADQ-----EQ-YIEAWH

ST-INNGYSYLIHETNNAKK-KHHYSPSEVIAS--FDFEEKI-K--EIR-KNVK-KPS--

--RKE-IENFIDN-YSNEKL-TKEELQE---K-IGVLEMAKHKILLDH-IDDIL------

-----------A-YKKHQQFLKDFK--GQ-----RCRTYWLYGVSGIGKTKLIREILEE-

--RHPKDFFISG---SSKD-HFQ----EYKGQ-HFIVINDLRP-RDYEYGQLLTLLDPWE

--IDKTAKRRYKDVF-I---NVCAYFISTPYSPLNFYNECR-IDNRLVDTFSQLER----

--RVI-ALHL-TKDNREKIKQ-------FEAIW---K---IK-SQKNTSHAN--------

--NKSND---------------------

>pCRESS8|WP_012845653.1

MQIN-EMKVIR--SRNFMYTQ--DLD--HLP-N-----KDTLKTRL-E--KSG----AQE

WAF-ILHDKDVDENNKKI--RPHFHVMLRFKDAKTISRISKIFNDK-----QQ-YIEVWK

NS-INNGYSYLIHETSKAKN-KYHYKDSEVVAS--FDFKSKI-N--SIR-RKIN-KPS--

--KQA-VDNYIED-YANEII-SKEDLQN---N-IGVLEMAKHKNLLDH-IEDIL------

-----------A-FKKHQKFLKEFK--GK-----QCKVIWLYGKAGVGKTRLIRNFLEH-

--YYPNNFIILG---SQRD-HFQ----EYKGQ-NYIVINDLRP-NDYEYGQLLTLLDPWE

--NDKMAPARYHDKY-L---NAKAIFITTPYSPKDFYNTCN-IENIFIDSFDQLKR----

--RII-SFHI-TENNLSQLSQ-------KNKSK---R---TK-EKNNGSTIN--------

----------------------------

>pCRESS8|CDI42894.1

MK-----KTVR--SRNFMYTQ--DVD--HLP-K-----KDNLASIL-E--KSG----AQE

WAF-ILHDKDVDESKKKI--RPHYHVMIRFKDAKTITKISKIFGDK-----AQ-YIEAWH

NT-INNGYSYLLHETNGSRN-KYHYDVSEVTAS--FDFKSKI-E--SIR-RKVK-KPS--

--RQA-IEDYIED-YSNGIL-TREALQA---Q-IGVLEMAKHKTLLDH-IDQIL------

-----------D-QKKHNEFLEEFK--GQ-----KCVTYWLWGESGVGKTRLVREALEK-

C-IEPTNFCILG---SQRD-HFQ----VYEGQ-NHIVINDLRP-NDYSYGQLLMLLDPWE

--NDKMAPARYRDKY-L---NAKSIFITTPYDPFSFYNGCY-IENMVVDSFEQLKR----

--RIL-PLHV-TTKNADQIKE-------RKAIF---Q---IN-AKQKASHTD--------

---QSND---------------------

>pCRESS8|WP_011254167.1

MK-K-GKKIVR--ARQFTYVQ--DTD--HLK-E-----PKQFQDFL-S--KSG----AVE

WAY-ILHDKDADQNNK-I--RDHYHVVLKYANPQTISKIANIFKDK-----EQ-YVQIWN

GR-IDNAYSYLIHETSDAQN-KYRYSPKEVVAS--FNFEKRI-E--KIR-TSIK-RKSS-

--KND-IEEKLKD-YAENII-SLYELRK---S-IGAFTTPSIQRHIKE-IKKLH------

-----------D-EDNHQKWLRSFD--GK-----KMKVIWLYGEGGTGKTRCARAMTKD-

-----DDVVVLG---SSND-YFQ----AYDGQ-RVIIINDLRP-SDFKFGDLLKLLDPYE

--HSKEAPRRYRNVK-L---NLEKIIITTPYSPISFYNHCY-IEDKKIDKIEQLTR----

--RITQTIEV-TNEFTKKFL---------------------K-EHERDEKSD--------

----------------------------

>pCRESS8|WP_056985318.1

MI-----QKVK--SRQYMMVQ--DLD--KLP-D-----LDKLKEIL-S--GLK----AKE

WAF-IEHDKDKSENGGLV--TPHVHAVIKFENERMLDTLADTLKVK-----PQ-YLQVWK

GR-INNAYSYLIHLTSGAKN-KHIYSPKEVVAS--FDFPKRI-S--EIT-NRVS------

--KQE-IKDALNM-YANGGL-SQTELKT---K-IGNLAYAQNLETIKK-LNTVL------

-----------D-NQAHQEWLKSFQ--GQ-----KMTVDWYYGKAGVGKTRLALKEAKE-

---SGEQYCVLG---SSND-YFQ----DYDSQDHVVILDELRP-NDLKYGDLLKIMDPYQ

--HDKHAPRRYRNVA-L---NIEKLIITTPYKPETFYKMTK-IADRRVDTVEQLKR----

--RISKVINV-TPELAKKEF-----------------------GDNHEK-----------

----------------------------

>pCRESS8|WP_008472153.1

------MGELR--SHNFMYVQ--QTK--YLK-K-----PEEFSEVL-N--GIRA---DVK

WAF-IKHDKD-----EGV--EEHYHVILHYEHSSRISTVANIFDDD-----PE-RVQIWD

NR-WNNACGYLIHATKNSDG-KYPYDVSEVTAN--FDFAKKI-T--EIQ-SRVS-G----

--AKQ-IENVIKE-YGNGDI-DRDELEL---K-LGDAELAKNHIWISR-IDDIR------

-----------A-QRKHEEFLKDFE--GH-----AQETIWLWGAAGVGKSRYADFLTQG-

-----KETAKLG---SSRD-YFQ----DYHGE-SFVILNDLRP-NEFSYADLLRITDPYQ

--HDKSAPRRYHDLK-L---NLKMLIITSPYSPDDFYEYCK-VNNYQIDTFDQLKR----

--RIH-AIHI-TSEFMKEVMP--------EWQG---F---M-------------------

----------------------------

>pCRESS8|WP_013641468.1

------MGELK--SHNFMYVQ--QTK--YLK-K-----PDQFIELL-N--GRKS---ITN

WAF-IKHDKD-----DGV--EEHYHVILHYKYAARLSTVSHLFDDD-----PE-RIQIWD

DR-WNNACGYLVHATSNSDG-KHSYDVSEVTAN--FDFDEKM-K--EIQ-SRVS-G----

--SKN-IEKVITQ-YGNYEI-SREELEL---K-LGDAELAKNHVWISR-IDDLH------

-----------A-EREHENFLKEFK--GR-----AQETLWLWGEAGVGKSRYADFLTKG-

-----KKTAKLG---SSRD-YFQ----DYKGE-NYVILNDLRP-NEFSYADLLRLTDPYQ

--HDKAAPRRYHDLK-L---NLKTLIITSPYSPEDFYEYCK-VDNYQIDTFEQLKR----

--RLH-VIHV-TDELMKQVMP-------SDLIG---F-----------------------

----------------------------

>pCRESS8|WP_057906729.1

MKNK-NNKAIR--SNAYMYVQ--QLD--KLP-AKDQS-IDVLIKRI-K--NVPE---IKR

YAL-IIHDKDSDKDGNAI--KPHVHVMLELDKQRSVNKIAKALDDS-----SE-RLEYKR

HG-IENGYAYLIHQTQGAEK-KYQYSPEKVKAN--FDYPKYI-K--KLQ-QRVR-VTNK-

KSKEF-IKEVLNN-YLAGKI-SEIEAKR---K-VLPLMLPRFLRQLDA-VKSTK------

-----------F-EIESDEWFKNRSENNK-----SKSVVWISGHGGTGKTVLAKMIAEN-

--VMKSAYYMSG---SDKD-YFQ----DYNGE-HCVILDEFRP-DKISYSDLLKMLDNNR

--FDVNAPSRYHDKK-I---LADLIIITSPYNPARYYQNDE-SIRPTVDGFEQLDR----

--RITMTLCV-EKDKIS-LMKYKGYKAEKFVRE---I-P-IS-YIKDDKNYK--------

--KIIPKVLEKLHKNIGLKL--EK----

>pCRESS8|WP_057827851.1

MKNK-YNNAIR--SNAYMYVQ--QVD--NLP-AKGQS-LDALIKRV-K--SVPE---VKR

YAL-IVHNKDSDEGGNTI--KPHIHVMLELDKQRSVNKIAKALDDS-----SE-RLEYKR

HG-IENGYAYLIHRTQGAEK-KYQYSPEEVKAN--FDYPKYI-K--KLQ-QKVR-VVNK-

KSKEF-IKEVLNN-YLAGRI-SEIEAKR---Q-VLPLMLPRFLRQLDA-IKNTK------

-----------L-EVEADEWFQSRSKGDT-----PKSVIWISGSGGTGKTVLAEMVARN-

--ITGPDYYLSG---SDRD-YFQ----DYNGE-HCVILDEFRP-DKITYSDLLKMLDNNR

--FDVSAPARYHDKK-I---LADLIVITSPYDPARYYQNVE-DIRPAIDGFDQLDR----

--RLTMVLSV-EKDKIS-LMKYAGYKTESFTSK---K-P-IS-DIKNQKEYK--------

--EITPKMLAGLHKNIGLKM--RK----

>pCRESS8|WP_057827085.1

KEQK-TASSPR--SRGVMYVQ--QLD--KM---KFSS-LDALKRRV-Q--SLSK---LKR

FAM-IVHDKDTNGDNELV--KPHVHVMLEFESPRMLSAIAKELDDA-----PE-HFEHNK

NG-INNGFAYLVHRTKNASN-KYQYDPDKVIAN--FNYQGFL-D--KLK-TEVN--EGR-

YEKMG-ISNLLGD-FINGKL-TKIEAKE---I-AKPGQFAHICKRIDE-SEVQM------

-----------E-ELKADKWIQEMQGKHE-----AKKVFWIYGPAGVGKTTLAKMITES-

--LDK-GYFTSG---SSRD-YFQ----NYHGE-HCILIDELRP-NVIDYSDLLRILDPYN

--YNCNVPSRYHDHR-L---TANAIVITSPFSPGDFYIHQR-DLNTEVDAFKQLHR----

--RLNYVIQV-KPLKIR-LMELISDYVPKYRSA---TPPAIE-SGKFQENYD--------

--RCILSVLKKL-GNTNPK---EKSTIS

>pCRESS8|WP_016356676.1

-------MTKK--YRNFMYEN--QLK--YMKED-----INMLAKYV-E--EVLN---PAE

YAI-ILHDKDTINESELV--APHYHIALKFENPRNVNSVAKKFNDL-----PQ-NFEIWL

NR-PNNLYSYLIHKTSDAHS-KFQYDVTNVVAN--FDFTERI-N--KIT-KSIH-RGR--

--NEE-IRHLIDS-FGNGEI-TLKLLME---E-LSPTEYARNENQINI-IKKLL------

-----------A-NKRFEAFKKRMDSEEK-----RIEVFYLFGGTGTGKTRFAKTRYKE-

------NRYITG---SNRD-LFA----NYDGE-TVVILDELRP-NSISYNELLKITDPFN

--FENVVGSRYLDKK-L---VAETIVITTPFSPEEFYQTLK-GEQTNIDKKEQLFR----

--RIN-VFKF-DDVYIYPLI-WDQTKRVKQENQ---W---SE-SVTFKTENDI-------

--KATKNLFKMS----------------

>pCRESS8|WP_016622553.1

-------MTKK--FRNFMYEN--QIE--YMKKE-----IDKLSKYV-K--ETLN---PVE

YAI-ILHDKDFITENKLV--APHYHIALKFENPRKVNNVAKVFNDS-----PQ-NFEIWL

NR-PNNMYSYLIHKTNQAKE-KFQYDIDDVVAN--FDFKERI-N--KIT-KSIK-RNR--

--KEE-IQHLIDS-FGNGKV-TLNQLMN---E-LSPTEYARNEHQITI-VKKLL------

-----------A-NQRFEEFKSRMENEQK-----KIEVFYLFGDTGTGKTRFAKSRYKE-

------NRYITG---SNRD-LFA----NYDGE-SILILDELRP-NSISYNELLKLTDPFN

--FENVAGSRYFDKK-I---VAEKIIITSPFSPNDFYRALK-SDKSDVDKKEQFFR----

--RIN-VYKF-NYDYIIPML-WNDDKKVKIENH---W---SE-SIIFKTENDI-------

--KATRVLFE---SPQ------NDKGV-

>pCRESS8|ABP89830.1

MKKE-KVPSIR--HTAYMFCS--TVE--SVS-K---NLQEIIKLFQ-E--TLN----PFE

IAG-IIHDKDID-----T--EPHYHIIVRFKNAVWLNSIINKLSQN----QSN-FFEAWK

GK-VNNAYSYLIHRTEDASE-KHQYTVDEVIAN--FDYAERI-E--NIE-SKIQ-SNSR-

KETINVVRNLINK-IIAGDI-SFDEAIK---E-VDGYTLVKYDREFSR-AKKRR------

-----------T-EIDFENWKENALKNGF-----KREIIWLYGPSGTGKSRLCKHFAKS-

--L-GKPFYTTG---SSRD-PFQ----NVASQ-ETIIIEEIRP-GNFNYADFLLIIDPFN

--ADATASSRFFDKP-I---IATTIIINTPFSPFQFYESIS-KQVGKIDTVIQLIR----

--RITLLQEV-TDKSII-TYKFDNEKNKRIDNP---Y---CD-KDKIEFNSDV-------

--NKYKEMTLKI-EENDDR---ND----

>pCRESS8|CUR41281.1

---E-GNKITR--SQIVMYVQ--QLS--YLP-N---S-LDALERRL-K--ELKKDKGLIN

WAY-IIHNKD-RKNGKAI--EKHIHLDLRFKTRMSVKSIAKMLDDE-----TE-RIESLE

QS-WINALSYLIHRTAKSKD-KYPYDPNEVKAN--FDYIKTI-K--NAE-KSIV-G----

------ANKIVDQ-FLKEEI-DYDTAEM---L-LSAKVLSKNKKILDD-AQNFL------

-----------N-RKHYKEWVKDKKKTNQ-----SIIVVWIWGEAGTGKTSFCKDFMNE-

--R-NIEYYEAS---GHND-PFQ----NYAGE-KGLILDELRP-RNITYSDLLKILDPYD

--YDKTAVARYHNKY-L---MCDYIFVTSPFSPYSFYKNAQ-VKDT-NDSLDQLHR----

--RISILLHF-TSKDIIEVKVEGNTYKEQEARG---Q---Q---------DN--------

--LSLN-ELTDL-KG-------D-----

>pCRESS8|KRN07545.1

MKENEKARSYR--STVWMYEQ--QLE--HLPFN-----IDALKRRV-D--SLIDNYNLDK

FAM-IQHAKDVNAEGKRV--KSHIHLVMTFKERVAANSLGKVFGDQ-----PQ-QFESAK

KG-ADNAFMYLIHQTDNSRD-KYQYRVEDVVAN--FDYANFV-S--RKR-AQID-P----

--R-----DIIEL-LGAGEI-QEEQARA---M-MMANTYFKYSRRISE-VALGA------

-----------N-KLKFEKWLKNKIETKE-----SIKVIWIYGGAGTGKTRYAVEFADK-

--R-KISFFKTV---TTND-PFE----GYNGQ-KILIIDELRP-ETLKYPDVLHLLDPMS

--YEKKTVARYHNSN-I---MADFIFVTTPYDPLTFYEKIT-KLDRSVDSFEQLRR----

--RIGLLLHF-KKKSIDAEYLEKNSNSRSNENP---Y---------LNDNRSVN------

--------LEDLEKSD------DKK---

>pCRESS8|WP_003665528.1

MPEKGETPKVR--SRVWMYTQ--QVE--DLPF----DSIDALIRRV-K--TVPN---LDK

IAW-IVHDKDINKKGKKV--TPHVHVGFTLTKRTTISRMSKILNDR-----TQ-QITSVA

NS-TKNLMGYLIHHTREAQG-KHQYAPSKVHAN--FDYPSYV-E--QTE-EITS------

------TRDILDE-YANENI-SRDQAES---L-LKGSDLAHNLRNLDA-LDSYI------

-----------L-EEKRRRWVQKMKKAKK-----PIYVVWLSGAAGTGKTTYAKRYAEKH

----KLTYFVTT---SQND-PFQ----GYRGQ-QVLIIDEIRP-ETLSYADLLQICDPYL

--YEKNLTARYRNPS-F---QSSIVFLTSVYTPLEMYNAMR--VKRKIDTFNQLKR----

--RIGMNLDF-SNREIT-SFVYDFDYKRSIPNP---Y--------STSGLGNMF------

--------FNELDEQI------SEESLK

>pCRESS8|WP_006499656.1

MA-TISKSKPR--SAVLMLVQ--QVQ--LLPN----NPKHFLKSKC-E---LKRRYGLKK

YAF-ILHDQDKSKNNDLV--VPHYHLVMQFDHRVDVAAVAKIFEQG-----IE-HFESTK

TA-AKNSFAYLVHATDNSRD-KVPYDPQKVTAN--FNFRKFL-A--DSE-SELS------

------TADILDG-VAEGNI-TKDQAFD---M-LRARILVHNKKSVET-MAEEY------

-----------Q-RKHHLEWLKKRNEEGK-----GIPAVWCFGQGGTGKTSYAKHFAEEH

----GLSYFVTS---GSND-PFQ----GYQGE-EVLIIDELRP-DVLPYSDLLQLLDPFN

--FEKRLKARYFNPF-F---SSNFIFVCTVMGPIEFYNSMA-IAHKNIDTFEQLRR----

--RLAMVLLF-DHHKIA-QVIPDFRNGQYKANP---F--------ANQGHVNLL------

--------MGMLDKEQ-----LDDKGSK

>pCRESS8|WP_046923918.1

MK-K-PKKVQR--SSVFMFEQ--QVG--YMP-----P-IDQLYEKA-K--HAK---GLVE

LCY-ILHDKDRKEDGTLK--TPHYHMSMYFDHRKTVNSVAKMLNSK-----PQ-QIESTV

EA-KNNAFAYLCHRTKNAQEGKYQYSPDEVTAN--FDYPKWL-A--EQE-SKVN-N----

------ANDILEL-LNDRHI-TKDQAIE---R-IKGVAYSRNAKKIET-IAYAN------

-----------L-RADYKDWLKRMKEKKR-----DVKVLWLYGYAGTGKSHFATDLATD-

--K-NLTYKKLT---SKHD-DLTD--LSIKDQ-DVLIFDDFRP-DTLPYSSILQIFDPLN

--LGVSLDARYHNAY-L---MSEYIIVTTPFSPYEFYQSMY-IRNRKIDTFEQLSR----

--RIYATMHF-TTDEIYTVEPKIQRYVDSIPNA---W---SQ-I----AVNG--------

--KKKSHSFADFTEN-AVCID-QKKSPV

>pCRESS8|AKG47101.1

------MSDIR--SQVIMFTQ--QLK--HLD-S-----QDELIKKV-N--KLPY---LDQ

YAF-IIHNKDVNTDNSPI--TSHIHLVLCFKQRVRITSIARNLDQK-----EQ-YFEDIE

TS-RNNAFAYLIHNTTQAKKGKYQYSPNKVTAN--FDYVKLI-N--NLK-QIIF-YS---

------PKQVLAD-FNSGNI-NKLEALK---R-IKSPRIPQYVASINK-IEEIN------

-----------I-QLKQKNWIAEHEKSQK-----PIAVVWVYGFSGTGKTEFAKHIAKK-

Y-SIDNRYDFTG---STRD-LFQ----NIGTA-SSLIIDEIRP-KDIKFNDLLKITDPFN

--YRKFAPARYKDRA-I---IADTIIFTSPYSPVRFFSKYK-LDNN--DTFRQLQR----

--RITLTIEI-TTKQIIQLEPVTKPTIKAINQT---Y--STT-YIQRAISKNT-------

--QQAKISLSDL----------------

>pCRESS8|WP_046025501.1

-MAS-LKGTAR--SRVMMFEE--QDK--FF--E-ASDIGEFLKQSC-Q--KMASKYGLEE

WAF-IHHDKD-----EPA--EPHYHLTMYFKGRPMVSSIAEMLGTT-----PK-QIEKVE

TA-RKNAFMYLIHATLNAEG-KFPYPPEKVVAN--FDFIRFA-R--YNL-MQVT------

------PEGILED-LGVGKL-TKTQARE---K-FMATVLAKYNRKIND-VAEAS------

-----------L-AIQYTKWRKEREAKHY-----QLMTFWFCGPTGTGKTRYAKYLAEN-

--VFKMPYFVSG---GQRD-AMQ----DYEGE-HLIMWDELRN-N-VYYLELLRLLDPYN

--YDKAISSRYFNKN-L---MPEVMIITSPYRPDELYKIMD-ISDRKQDKLNQLTR----

--RVPLIYEF-QKEHIL-ILKWNEYNQEPSVEE---L---IK-ADSEKDS----------

---SPAEPFYGIDKHSDTNVP-DSNELS

>pCRESS8|WP_034540695.1

MKIN-KATSPR--SKVLMFEE--QDQ--HFK-D-SSSIEEFLTDRC-E--VMIKKYGLKE

YAF-IHHDKD-TNNGQPV--KPHYHLTMYFDNRPMVSSIADALDTT-----EN-QIEKVE

TA-RVNALMYLIHATRNAQG-KYQYPATDVIAN--FDYVKFV-K--DHM-LNDD------

------PTDILDD-LGNGKI-VRTQARS---R-MMAQVLAKYNRKIDE-IAEAS------

-----------L-AIQNEVWRKEHEDSHS-----ELKVYWFFGQTGTGKTRFAKYLAKE-

--IFKMPYFVTG---ARRD-AMQ----DYEGQ-HLIIWDELRD-D-VEYSELLRLLDPYN

--FDKAISSRYYNKN-L---MPDIVIITSPYSPDELYSVMR-ITDRKIDKVDQLVR----

--RVPIIHEF-CHDKII-VRKWNSYEKRPSVEE---Y---VN-QSLPINSLQL-------

--IDQFLPFYGLNHPEIDPFT-DSNRLS

>pCRESS8|WP_002821392.1

MAG--NSRPTR--SRGWMFVE--QLK--SLN-R-----TSQIQNRI-R--KAG----PEK

WAY-IIHDKDVNAQGEPI--PAHIHLMMSFKSAVTAITLAKHFSTT-----PE-RFERNK

FG-IINGFNYLVHRTQNSIDGKVRYDPNDVKAN--FNFLDLI-K--QTE-QAIK-RSKKS

KSRES-VNFILDQ-FGDGLI-NKATARI---R-LEGHILAQNSTKLQN-LDKER------

-----------A-EIDYMNWRKMMIGNHF-----VKTTIFIFGETGCGKSLLAKKIASQ-

--SYPGSVYFSG---GSND-PFQ----DYEGE-RAVILDELRP-GIIEYPDLLRILDPYS

--WDTATHSRYHNSK-L---QAQLFIITTPYDPYFFYRFTK-DLVRFMDPFEQLNR----

--RISMTVKV-DRNFIY-EMKFNGVNRFPIKNP---I---GK-MIKNQSENF--------

-------SLSDLLKTDEGEK--DSK---

>pCRESS8|YP_006939186.1

M----TTSKRR--ITKFMYTQ--QLK--YLN-S-----IEQLKNNL-E--NDAY---IQD

FAM-INHNKDLDENNQNV--AEHLHVFIKLNQQKTIDYVADLVDDK-----AQ-YIENKS

RN-EQNGYLYLLHKTKSAEH-KHQYSVDDLIVKDGSNIKEKI-E--DYE-NNLK-KYQS-

KRKTV-VQSILND-YADRII-DEKELKD---S-LTNLELAKNKKLIND-IKQVL------

-----------I-EFDFQTYLEQER--YK-----NKQVVWIFGKSSTGKSMMSQLLAKD-

YISDINDIYVTS---SNRD-PFE----DYQNQ-KVLIIEEFRN-ENIGTNELLQLLDKTN

--GQVRVGSRYSNKK-I---MADLIIINTIYEPKYFM---------FDEPIYQLLR----

--RIDKLVKL-DNQKIE-TLEYDSKKDD------------IK-SIANNVENM--------

-------TLKKI----------DDFKL-

>pCRESS4|WP_000818357.1

---AEKKIRVR--ARVYSVVQ--YEFNP----TEDLHFNEVIKNAI-----LNKETSLDN

WAY-IRHDKDKYVEGDVR--PPHWHVLLKFKNQIEFSTIAKLFNVP-----EN-LVEKKT

-GAFFDYLYYMTHEDDKQLG-KHVYERDEVETA--NEMWEHV-D--IRE-DRRA-KKLSK

-----VEVFL--D-KLTSGM-TMKQVFE---R-DS-VVFAENATLFRR-AR---------

-----------------RSYLKYA-PTPL-----VRTNYHISGAGGTGKTLIAKSMARA-

MFPD-KFFFVVG---DGR-VAFD----EYDGQ-PIIIWDDFRA-KAFERGTMWKIFAIHP

--DKVSVHVKNGETT-L---INTVNIITSVEPFTEFVNGLAFKDN-KSEDVGQAYR----

--RFPIFIEV-SKNNMFATLGLTGGERE-----LINI--ELAK-----NQSKIN-KVFKN

VINSK---IEHVE---------------

>pCRESS4|WP_000186194.1

LKKTEKTKSKR--VRRVKIIQ--YENNP----TESLNFDENILNAL-----AYFSHRTKR

WAW-VKHDKDVITEGKPK--GTHYHVVMELTNPASISAIAKRFGVP-----AQ-YVEVIE

-GAVLDCIAYLTHEDAKQYG-KHLYDRDEV-SD--SNIWIDV-D--NQK-AREA---LTK

--RADARVII--E-KISQGM-TLSQVYE---F-DN-VMAVENKNLFKT-AR---------

-----------------QEYLKNA-PVPP-----VRTNYYVYGEGGTGKSLSAKVLARS-

LRPDITKYFVVG---DGA-VPFD----GYDGQ-PIIIWDDWRA-LHFDRSLVWKLFAINP

--ERISVNVKYGSTS-L---INAVNIVTCVDPYLKFMEELEYTDKRKKEDSRQAFR----

--RFPFFIEV-TAESIARNRGLSKNEIA-----LLKF--QLAK-----QQERGG-EV-AD

ILEAKKYQIKAL----------------

>pCRESS4|CBL40434.1

---TEPKKTY----RTFEIMQ--YEYNP----TEDLHFNRVIMKAL-----A--HKTIKQ

WIY-VRHDRD----GTPK--APHWHVYIYCNPAKSLDDISKWFGVP-----TN-MIELKV

-GSFLDCAEYFTHEKQPT-----LYDDTKLYSN--MPWRQML-N--ERD-EKRA-K---Y

--DIQFRDLQRVD-VLNFGK-TLKQCKI---D-DP-VLYVKEMQILKK-CR---------

-----------------LEYLYTQ-PVPK-----SRMNIYVTGQGGVGKGHTCKALARA-

LYPELDDFFTIG---AKN-ATFE----GYDGQ-PVIIWDDRRD-YELGRENVFNVFDTIP

--QNLRQNIKYGSVK-L---LNAINIVNSVQPYTEFLDGLEYKAKDKSEDKQQSYR----

--RFPFCIEV-KNFSFYENQGFVNHDKK-----------SVRRGNA-TDRKKI-AKLFAY

PINRAKD-FEHFG---------------

>pCRESS4|CRY93789.1

---------------IFNIMQ--YEKHP----TEKL-IDEVIKVAL-----A--HKSIKQ

WAY-IDHDADVYSLGETK--PKHWHIVCRCQAAVEVSTIARWFKIP-----EN-FVDVPK

-GAFLDCVEYLTHEREEQLG-KRLYEDERVRAN--FDFREDL-E--ERA-EKRL-K---Y

--DTDPKTAMWYD-VMFNGL-TLKQALE---R-DR-WAYMEMLEKLKK-AR---------

-----------------LDYISRM-NPPD-----TRINYYVEGKGGVGKGLISRAIARS-

LYPQYDDFFEVG---AKG-APFE----GYDGQ-PVIIWNDRRA-YELGRGNVFNVFDSHP

--TKQRQNIKYGSIN-L---CNEVNIVNSVQPYAEFLDGLEYEDKNGVEDKGQSYR----

--RFPFMVIV-HEEDFMINKGFIVGEKA-----YLKH--NFRR----GQNEKLATKAVKK

ITMRSEK-LEQFK---------------

>pCRESS4|WP_021639163.1

---MAKKKGF----YNYNIMQ--YEKHP----TDEL-LTEKIKSVV-----A-KYKSIDR

WAY-IIHDKDVYT-GTKK--PKHFHIALKMKNCIELDTVAKWFGIL-----PN-YVEIPK

-GAFLDCVQYMTHERTEQQG-KFRYADLEIQAN--FDWRAEL-D--KRD-EMKA-K---W

--ELSDRQVMGQR-IMLEGL-TLRQVKA---E-DP-LLYADNLEFFRK-MR---------

-----------------GVYLSDL-EPPK-----TRINYYLCGDAGAGKGVMSKAVARA-

LFPELINFFEVG---ADN-ALFE----GYDGQ-PVLIWHDRRA-GELGRSNVYNVFDTHP

--SKQRQNIKFSSVG-L---INRVNIVNSVQPYVEFLETLAKDDNDGAE-KSQAYR----

--RFPLIINI-HPEEIYINKGFI-SQHE-----YEEH--NMRK----SKYETLRGMVVKP

VVIGQNN-SKALCRKTKEQLD------H

>pCRESS4|WP_007889993.1

---VMPK--------VFNIMQ--YCKHP----TEVL-ITEQIKSLF-----D--RRTIKL

LAY-ILHDEEDFDEGDKK--PPHFHVVFRTDRNTDLETVADWLGIP-----VQ-YVDGAR

DGTFVDLLRYLTHESEKEKG-KHRYPDEKVIAN--FDFRAMI-D--EAD-IREA-R---Y

----SPKDYYRHK-VAYEGM-SISEVIA---E-NE-DAYLKDMTFLDK-CR---------

-----------------SKYLAAFAKMPD-----LRINIYLDGAGGIGKNTASKAIAHV-

LYPDM--YFEAG---GAN-TSFE----GYDGE-PVIIWNDCRS-TRFERNELFDILDPHP

--TDARHNIKFGSVR-L---TNPINIINGIEPYNKFLDGLAYVDKRSGEDSSQAYR----

--RFPIIMCL-REDDLLFNKGVFNGTRE-----YISY--SFAK-------QRLAEVVIKP

VLDIKKI-PDEFK----KKED-------

>pCRESS4|CRY97508.1

VSEAQPKKELK--SRIFNCLQ--YEKNP----TADLHFTENILKCV-----A--HKSITR

YAY-IRHDKDVVTEGQPK--GVHWHIVLETAGLMPVSTIARWLGIP-----ES-MVEIPK

-GAFIDCVEYLRHSDIRQKG-KYVYEADEVKAN--FDWQTEV-T--EMV-LRKT-K---Y

--PLSQADFLKNE-VLYNGM-RLAEVQE---R-YP-SIYMKEQTVFDR-LR---------

-----------------MKYLVERAPLPA-----SRINFYIEGLTGYGKDTMARSIARG-

LFPELAKYFEIG---GKK-VTFD----SYDGQ-PVIIWSEFRA-EALGYEEVLGAIDIIP

--KNNRHHKKFGAVK-L---INSVNIVTSTEPYAEFLKGLESD-----PDPSQANR----

--RFPLIIPI-HVKDILINSGYLGADT------YTAY--SFGA----NTRPELLSELIKP

VIDEFKG-LDVFRDKTEDELKEDKASCK

>pCRESS4|CDA18875.1

---STNKITS----RTWELVT--DVKHP----TEEL-INKKIDTVL-----K-SHASIKE

FAY-ILHDKDTYTDGDIK--PAHFHVVMRFARAQELDSLSEWFGID-----KN-FFEKKK

-GSFFDSVLYLTHQSDKEKG-KFVYSEDEVFCH--FEFREFV-E--ACE-INKE-K---Y

--NICIKDKLRLD-VLYNGM-SIRQVKK---N-YP-MEYNDDMEGLQK-RR---------

-----------------GDYLKDA-PLPP-----YRISFYISGSGGAGKGLFSEALARA-

LIDPDGEFCYVG---SDS-VCFE----NYDGQ-PVLIWDDCRH-NKLDRGTIFNVFDTKP

--KRISQKKKYSQTN-L---INPINIVNSVEPINGFLDGLQYYDKSEAENKAQSYR----

--RFPICINV-HPTYIHVYEPFFGGE-------IYKY--PFVD----GSDSKEYLKALKP

VILNKKDSPEKYIEPFEEFLKFDVLNDN

>pCRESS4|WP_017824301.1

---YA--EQIK--GRIFTITT--DCVHP----TEVL-LTVTIEKRL-----S--KSSVEY

FAW-VLHDKDCFTEGEHK--PDHFHVVMQLKNQASVGQVARGYKLH-----PG-CVRKKE

-GTFLDCIEYLTHEHEKQLG-KHLYADDEVHSN--FDWRMAV-D--ERV-AARK-QGF-H

--AAKKM-KIRLA-VMNGQM-TLKQVRE---D-EP-GVYVQDLEKLQK-LQ---------

-----------------QDFRLHQ-PAPR-----HRTNYFIGGMAGTGKTQLAKLFART-

LFRDLDAYYVAT---DPR-VPLQ----NYKGQ-PVIIWDDYNA-LALGRSGVWQVFDDHP

--SATDVNIKYGATR-L---VHTVNIITKTTPYAEFLDGLEYTDKSKAEDRNQSWG----

--RFPVVFEV-TIHSMLLNRGFV-DDTD-----YEEV--SMRE---DAEREAATDFLLRP

MLKATEVVLD---DAKEEAGR-DLKRLE

>pCRESS4|CEI31812.1

TGRYADSDDLG--GRVFLITQ--NERYL----TRVL-MSERIGKAV-----G--KKGMTD

WAW-IKHDQDQYTVGHRK--ADHFHIAVRRKSFSTLGQIARAFGVP-----PN-AVEIKP

--AFMDLIEYLTHEHPNQAG-KYHYDDGKVHAS--FDWRPAL-D--EHK-LARA-AKA-G

--SLKKRDAVREA-VMLGEM-TLKQVRE---D-ER-AIYIQDLDKLQK-LR---------

-----------------QDFMLHQ-PAPR-----HRTNYYIGSPAGTGKTQLAKLFARM-

LYPDLDAYHVAT---DAR-VPLQ----NYKGQ-PVIIWDDYSV-PALSREGVWQVFDDHP

--SASDVNIKYGSVR-L---VHAVNIIAKTTPYAEYLDGLEYTDASEAEDRNQSWG----

--RFPVVFEV-TPDSMSVNRGFV-NDTD-----FQKV--SMKA---DDEREAATEVLLRP

IIAGADVVID---HEAEEAANRDLESVA

>pCRESS4|WP_044572803.1

TAKYA--EALK--GRCFEIVQ--DEKHP----TAIL-LTRRIEKVL-----A--KRPNDL

HSW-VRHDRDQYTDGEYK--VPHFHIAEKRKNEASVGQVARAYDVA-----PQ-YVRVKP

--AFLDLVDYQTHGLERQLG-KNLYDDSEIHAN--FAFREEV-D--KRV-AKRI-N---S

PRKQTPIDKLAMR-IQEDGL-TLRLAKE---E-DP-LSFNRAPGRMEK-SR---------

-----------------ATYLRHL-PPPS-----SRINFYFEGEGGVGKDLLAKALART-

LIPGNWVFFSVG---GEN-VGLD----GYDGQ-PVIIFEEARA-GSMGRKELFAFMNPFP

--EKQSLNVKYGATQ-P---VNTITIFTGPDDYDTFLDGLEFIDKSKAENKPQARR----

--RIPIIIPV-REGSLLVNKGFA-DNTR-----YHVY--NIEQ---EQRRIEIHAGQVAP

IVKASKD-IAEFSEELEALAHLEIRAAD

>pCRESS4|WP_067940518.1

----VKRARMQ--SRVFSVMQ--YREHP----TEVM-LTQQIDEGL-----AALGDRLHR

WAY-VWHPYDRLVEGI-K--GLHAHMVLWVAPRPTIRTVSDAFSIP-----SA-RVKPPK

KGAFFDLAEYLPHESRGRPG-KYQYGRGRIVAN--FDFGREL-D--AHM-AMRR-TAA-E

--SAKLSK-LFQA-VDKGSL-TLKQVRD---Q-EP-AIYFAKLAHFQK-LR---------

-----------------GDFLSHQ-DAPE-----SVMNFYVFGEGGTDKDLLAKALARA-

LTPD---YFKVG---GEN-VSWE----GYDGE-PVVIWEDMRV-GTASRGMLFRILEPDE

--KPV-VNIKGSKTQ-L---LNRVNIVTGPEGYEEFLRGLEYESMQHPENLGQGFR----

--RFPVIIPV-AEREIFVNSGVL-NGTR-----YERY--DLEL---TAERERVRARTVAP

IVELDA--FAEFAPQSSVEVA-----VA

>pCRESS4|WP_005464724.1

----AKRARMQ--SRVFSVMQ--YREHP----TEVM-LTQQIDEGL-----AALGDRLHR

WAY-VWHPYDRLVEGI-K--GLHAHMVLWVAPRPTIRTVSDAFSIP-----SA-RVKPPK

KGAFFDLAEYLPHESRGKPG-KYQYGRGRIVAN--FDFGREL-D--AHM-AMRR-NAA-E

--RAKLSK-LFQA-VGKGSL-TLKQVRD---Q-EP-AIYFAKLAHFQK-LR---------

-----------------GDFLAYQ-DAPE-----SVMNFYVFGEGGTGKDLLAKALARA-

LAPD---YFKVG---GEN-VSWE----GYDGE-PVVIWEDTRV-GTASRGMLFRILEPDE

--KPI-VNIKGSKTQ-L---LNRVNIVTGPEGYEEFLRGLEYESMQQAENLGQGFR----

--RFPVIIPV-AEREIFVNSGVL-NGTR-----YERY--DLEL---TAERERVRARTVAP

IVELEA--FAEFAQPSAEEIA-----AA

>pCRESS4|WP_043534193.1

----KKRARKV--SRVISVMQ--YHQHP----TEVI-FTQQLDEGL-----AALADRLYR

WAY-IWHDSDRLVEGL-K--GLHVHIVLWFKPRPTVRTVSDALTVP-----SP-RVRVPN

AGAFYDLCEYLCHETRGAPG-KYQYGRGRVVAN--FDFSASL-D--AHM-ATRH-DAA-E

--GAKLSK-LFQA-VGQGTL-TLKQVRD---Q-EP-AIYFAKLAHFQK-LR---------

-----------------GDYLAYQ-DAPE-----SVMNFYVFGEGGTGKDLLAKALARA-

LAPD---YFKVG---GDN-VSWE----GYDAE-PVVIWEDMRV-GTASRGMLFRILEADE

--KPI-VNIKNSKTQ-L---LNRVNIVTGPQDYEEFLRGLEYESMQEAENLGQGFR----

--RFPVIIPV-AEREIFVNSGVL-NGTR-----YERY--DLEL---TAERERVRARTVAP

IVELDP--FAEFAQPSAEEIA-----AA

>pCRESS4|GAC78794.1

LPK-KSRARTQ--SRVFSVMQ--YRKNP----SVVM-LTQQIDQGI-----KTLGGRLHK

WAY-IWHPYDRLVEGV-K--GLHAHMVLWIAPRPSIRTISDAFAIP-----SA-VVRTPK

KGAFYDLCEYLPHESRGSPG-KYQYGRGRVVAN--FAFGREL-D--AHM-ATRH-NAATD

--GAK-LSKLYQA-VGSGSL-TLRQVRE---R-EP-AIYFAKIAHLQK-CRD--------

------------------DYLLRA-PLPP-----FRTNYYIGGPARTGKSLYAETLARQ-

LYPGLALIYMVG---RPG-VAFQ----SYDGQ-PILIWDDYRP-LAIQRDSIWPVLDIDP

--KRVQVNKKFGAVS-L---LNSVNIITGIQSYVEFLDGLEYTDKKEVEDKDQAYG----

--RVPLVAAV-TSETFYLNRGFA-GRSD-----FDPV--NMGR---EAAKEQFRARMLGG

MV------LAELE---PEELAADSATAA

>pCRESS4|WP_006681830.1

TAEWAYANEPG--GRVFMITQ--NLSHP----TQTL-ITTQVGKAL-----AKK--GVKR

FAW-ILHDKDVYTSGSPK--APHVHVVIQRSSFASIAQVARAFGVP-----PQ-CVEP--

--AFLDLVEYLTHENPKQAG-KHLYDDSEVHASKGWDWRTDL-E--EHK-IARQ-EKG-L

--LQRRRKEAALK-VAGGEW-SLDHVRK---H-DL-ELWSAPMSHLKG-LR---------

-----------------ADYLASL-APPL-----EVVNFYVFGPGGVGKDLLAHALARS-

LNPT---YFTVG---GSN-VSFE----DYDGE-EVIIWSDFRA-SACDRGLLFRVLDAS-

--EKVIVNVKGSHTQ-L---VNRVNIVTGPDDYKTFLNGLEYATNRTSENKDQAYR----

--RFPLIIPV-QEGEIYVNLGFL-NGTR-----YERH--NLEL---AAERESAIARTVAP

ILDDES--LSEFAEPTPEEIE-----AG

>pCRESS4|WP_052038917.1

TAEWAYANEPG--GRVFMITQ--NLSHP----TQTL-ITTQVGKAL-----AKK--GVKR

FAW-ILHDKDVYTSGAPK--APHVHVVIQRSSFASIAQVARAFGVP-----PQ-CVEP--

--AFLDLVEYLTHENPKQAG-KHLYDDSEVHASKGWDWRTDL-E--EHK-IARQ-EKG-L

--LQRRRKEAALK-VAGGEW-SLDHVRK---H-DL-ELWSAPMSHLKG-LR---------

-----------------ADYLASL-APPL-----EVVNFYVFGPGGVGKDLLAHALARS-

LNPT---YFTVG---GSN-VSFE----DYDGE-EVIIWSDFRA-SACDRGLLFRVLDAS-

--EKVIVNVKGSHTQ-L---VNRVNIVTGPDDYKTFLNGLEYATNRTSENKDQAYR----

--RFPLIIPV-QEGEIYVNLGFL-NGTR-----YERH--NLEL---AAERESAIARTVAP

ILDDES--LSEFAEPTPEEIE-----AR

>pCRESS5|WP_024390948.1

SGK-----NKR--SRLFFGMR--NYKFEHD--DTEDDWKNEIKKQF-E--AVS-DPQPTE

LTY-IFHDKDIDTDGEKK--ALHVHFVARFENAIYYDTTIEKFKCE-----PR-NFEK-G

RS-ETSALLYLTHTTSEAMK-KRRYNVSELNVLTFDEYRLKI-A--GR-------EGSNK

D--ED-VARIIDE-LSEGLM-TIDDVKQ---A-FDTMTWMKNKRYFKE-AVAEY------

-----------Y-QNKY----YDWLEKGR-----TFQLVYIQGSSGIGKTSFAREIGKEF

NRLKGLRIHNAP---NDTD-FLS----GYENE-AVTVFDDLRP-NTFGYTEFLNLFEKER

---VSKYSSRFNDKA-W---FAEVAVITKSTSINDWTSKLELKSASKPNVLYQPRR----

--RFSLIIDVN-DDE-VVLSSYVLT--DRKKMLIKKF----------KCPKKTDKKFQKK

LL------MVSLGEPTKADLKAKQKGGS

>pCRESS5|WP_029176301.1

TTK-----RKR--ARLYFGMR--NYKFEHD--DTEKDWREKVSQEL-E--NV----QADE

LTY-IFHDRDIDTDGEKK--ALHVHFVARFANPMDYEPTREKFGCE-----PR-NFEK-G

RS-ESSALLYLTHTTPESAK-KTRYNVQELTVVTIDEYRIKI-A--GK-------AGSLK

D--DD-VASIIDE-LAEGTM-LLTDVKQ---R-FDTMTWMKNKRYFKE-AVAEY------

-----------Y-QDKY----LEWLEKGR-----TFSLIYLEGPSAIGKTKFANKIARRV

NKAKGIPVHNAP---NDTD-FLN----GYEQE-VVTVFDDLNP-KTFGYTEFLNLFEKER

---VAKYSSRFNDKA-W---FAEVAIITKSTSIDSWTTSLELKTDKTANILYQPRR----

--RFSLIINIE-HDL-VKISKYVLI--NPKTNALKEF----------KPPKGT-DNFQEE

VL------SKYLGEATEEDVAEDSQNGE

>pCRESS5|WP_024393234.1

MSK-----TIK--SRLFFGQR--NYDFEHS--DSKDDWKEKIKKEL-F--EID---EYSF

LAL-IFHDRDIKESNELK--ALHCHFVIRFNNPRSYSNILELTKCE-----ER-NFER-S

TN-EGAILRYLTHTTPEAAE-KTRYNVSELLVKE---YRKKI-K--SN-------IGKKE

KVVDF-VNDLAYR-LSVGEF-KPINAIA---E-FGQSIYRKEKKKFQE-DYADF------

-----------L-ETKK----KDLLLNGK-----ELSTIYIDGFSEVGKSTFAQDLANAI

NEANGKETYLAA---KKKD-WIS----KYKDE-YITIFNDVDP-YDFNFTYFLGTFETKI

---LVDVGSRYKDKT-W---FSEYAIITKSTDIHEFVNKIELREDNHFNIRYQVQR----

--RFSLIIKIE-KNK-VTLSKF------NKKGLTFKF----------DDLQKE-GSIRKE

II------LSLLD---------------

>pCRESS5|WP_050238550.1

MSK------IR--SRLFFGQR--NYEYEHT--ETESEWKQRILEEL-F--SIS---DYNY

LAL-IFHDRDIKEHNELK--GLHCHFVIRFDNPRSYDSILELTSCQ-----ER-NFQR-S

TN-QGAILRYLTHTTPEADE-KTRYNVSEIYLKT---YRTKI-K--AN-------LGRKE

KVTDF-VADLAYR-LSIGEF-KPHTAIA---E-FGQSIFRKEKKKFQE-DYNDF------

-----------L-DSKK----RDMLLNGK-----ELSTIYIEGPSEVGKSVFAQDLANSI

NLSFNRDTYLAS---KHND-WIS----KYKDE-FVTIFNDLDA-NLFSFTDFLGTFEQKI

---LVDVSSRYKDKT-W---FSEYAIITKSSDIDEFVNKLEIREDNHQNIRYQVQR----

--RINLIVKIE-KNK-LTLSQF------NKLGVIFEY----------DNI-ND-KQIRKE

II------LTLLN---------------

>pCRESS5|WP_061866456.1

MAK-------R--VRRFSISR--NKQYEHQ--ESDTDWKEKVKSET-FYLATE-YGSLKS

IAL-IFHDRDLTSDGERK--GLHCHMILEFRNPVTITS-LEKFKFEAGKFQSR-NVEA-S

KS-SSGSYRYLTHTTDKAER-KTRYEVQELIVAEYETYRDKI-K--GT-------IRPEK

D--EA-LQEAFFK-VRTGEI-FDEEVRE---R-FTTELVIKNKKFIDN-SRQMY------

-----------Q-KEVF----EDMQNNGR-----NLKTFFISGSSGLGKSRFAKDLARRI

NINNGKSIYTAP---TAKD-FIS----EYKAQ-DVTIFDDVDA-KSFGFQEFLNIFDKDN

---ITKISSRYTNKA-W---VSHYAIITKASKIRNWIERVEYEKDKEV----QVSR----

--RFDLWIELDNNNQ-VNFYQF------------KHY--P-------DDNKKS-KVARKE

IT-----------------MKFRITNPS

>pCRESS5|WP_058211405.1

NGK-------R--FRTWNITL--NYDYEH---STAEEWKNKIRQLI-A--FN---TDDGW

CAY-VFHEKDILEDGLPK--SLHVHILKNFKEAKTQTAVMKMFNVS----REA-NCTN-A

RS-ITSSARYLTHRTSQADG-KHQYNIDEVQ-TINCDYLELI-K--NK-------SDRTI

EVDEI-VMGLSIN-IGNGKL-YWLKAID---K-FDIKLWNKYSRIFEK-NFKEY------

-----------I-QHKA----EDYKLKGR-----NLTTFFIWGDSEVGKTWLAKCMCLLL

SDR----IHMVP---ASGD-IAG----LYDGE-KASLWNEVSG-LELSNKEFLDRFDPKT

---YSPSNSRGKDKH-C---LSDYFFLTSTDDLETVVNNLEFEIKR----RHEINR----

--RLPIEIKCIPKNRRFTL---------------CSV--ICKNI---ESENQM-KKAAKE

IL--------------------------

>pCRESS5|WP_017368666.1

QAK-------R--FRTYNITL--NYDYEH---DTKEQWKEKITQLI-A--SN--VSHKDW

CAY-VFHDKDLLTDGLPK--PLHVHILVGFENGKTQTAVMKIFNVS----RPK-NCQH-T

NT-IAGSARYLTHRTEQADG-KFPYEVHAVI-TINCDYLTLI-K--GK--------EKVN

NIDEF-VAELSEK-IYLGKL-YPTNIFK---E-FDDRLYKKYRKEFEK-DFQEY------

-----------L-QHKG----EEYKIKGR-----DLATIYIWGVSEGGKSHIAKRMGLLL

TDR----LHLIP---ASGD-PSG----LYQGE-EVSIFNEISG-KEFNNKEFLGLADPRN

---YSPINSRGKDKH-W---LASYLFLTSTDSRETFIKNLSFEQRQ----RHEIAR----

--RVPYEIRCVSKNQMFSV---------------GSV--ICEHI---DDEKAM-QKSALE

IL--------------------------

>pCRESS5|WP_038978316.1

YQK--DFLATR--ARTFSGQR--NYEYDHE--ATEHEWRKQMVDMF-T--AI--QDDCEY

CYF-IFHDKDVLPNGDKK--GLHVHFVIKFKNPRVIRSIMKTFGIS----RTE-NISK-V

KS-VKGSLSYLLHITKQADG-KFIYGQDRLY-KVGTDFNKLTVS--DK-------DDKDI

IVEEV-LSQVTSL-REKGEM-AD---YK---K-FPSDIYYNYTTKREY-AEREY------

-----------F-ADKL----RYRNKHGR-----WLRNLYITGKGGTGKTTLANKLGYAF

ADKRG--VHVGA---AKSD-PMG----TYKNQ-KVTILNEMQG-SLFDYREIMNVFDDHQ

---QAPVSSRTKDIN-W---TADYLIMTSSKSFERFRNETELPTGQYKDVAFQFTR----

--RFSNY----SKGQYINVFQFN-----FKKRGQMAV--SDEFY---KHENEM-DKVVSM

IVIDQDEQL-RVKQENNPEVKTDKERLT

>pCRESS5|WP_046467524.1

VQK--DYAKSR--FRSFVIQR--SYDYDTV--ATPDEWMDKIIAQF-K--KE--GAKADY

YYF-IFHDADYLPDGTLK--SLHVHIVIHFKNPRTVGAVYKAFGVS----RFE-NISK-A

KS-IKGALKYLLHITPQADG-KTVYGMDKLY-FGGEDYKELIND--NKN-QEEA-EEKRV

VIARC-KSLLTDI-ATKGTA-PGE-WWE---D-FESDVYVNYKKKFQE-MEKEY------

-----------F-YRLT----QKKKREGR-----DLRNIFVSGEGNSLKSSVAKEIALRY

ADGRG--YHIAS---PPSD-FVS----LYKNE-KVSILNEMVS-DAFNPREFMNVFDDFQ

---IGSVSSRFKDIN-W---LADKTVMTTSDTFSEFRSNT------EKDVFYQFSR----

--RVEHYVRLDLEDKVMSVYHFD-----KKKYGVFVY--SKENY---KEEKDY-QNLIDK

V-LNQKSELERIKEEREKE--TDKERKS

>pCRESS5|WP_061417941.1

MK---INQNDR--KRKFFGQI--SYEYEN---ETEEDFRDSVIQRI-K--DYC-KNEDDR

YHI-IFHDKDLKDDGSSK--PLHAHFYIDFKHAHTYSSVYKALSIS----REQ-NLEF-V

RS-SIKTCRYLTHRNERNEH-KFPYEVKDVISSENANYIDDI-M--GKI-KNHS-KEKAD

EIDEY-CLELSYQ-ISEEGL-LIMEAFE---Q-FTQKAWNSNKRQFEE-NRQEF------

-----------I-QKEF----IRMSKGER-----NHTGIYIQAEGGTGKSYLARLLAEE-

HDRLG--AH--T---PSID-LGS----GYKGE-KTIVINELDA-SGMTFRELFQVLEPDS

---ATQLSSRFKDAY-I---INDLTIITNSDTYWDWCDSW------KNIEYHQLMR----

--RIRFVIKM-EKNKKIELWNYTATRDLKEKAKLETY--TLNSV---ENEEEF-RKIASD

IL-------ERIKKNIDSKRK-DQSECN

>pCRESS5|WP_061863770.1

MK---INQNDR--KRKFFGQI--SFEYEN---ETEKDFRESVIQRI-K--DYC-KNEEDR

YHI-IFHDKDLKDDGSSK--PLHAHFYIDFKHAHTYSSVFKALSIS----REQ-NLEF-V

RS-SIKTCRYLTHRNERNEH-KFPYEVKDVISSENANYIDDI-M--GKI-KNHS-KEKSD

EIDEY-CLELSYQ-ISEEGL-LITEVFE---Q-FTQKAWNSNKRQFEE-NRQEY------

-----------I-QKEF----IRMSKGER-----NHSGIYIQAEGGTGKSYLARLLAEE-

HDRLG--AH--T---PSID-LGS----GYKGE-KTIIINELDA-SGMTFRELFQVLEPDS

---ATQLSSRFKDAY-I---INDLTIITNSDTYWDWCDSW------KNKEYHQLMR----

--RIRFVIKM-EKNKKIELWNYTATRDLKEKAKLQTY--SLKSV---ENEEEF-RKIASE

IL-------ERIKKNIDSKRK-DQSECS

>pCRESS5|WP_049535277.1

MK---INQNDR--KRKFFGQI--SFEYEN---ETEEDFRESVIQRI-K--DYC-KNEDDR

YHI-IFHDKDLKDDGSSK--PLHAHFYIDFKHAHTYSSVYKALSIS----REQ-NLEF-V

RS-SIKACRYLTHRNERNEH-KFPYEVSEVISSNNGNYIDDI-M--GKI-KNHS-KEKAD

EIDEY-CLELSYQ-ISEEGL-LVMEAFE---Q-FSQKAWNSNKRQFEE-NRQEF------

-----------I-HKEF----IRMSKGER-----NHKGIYIQAEGGTGKSYLARLLSEE-

HDRLG--AH--T---PSID-LGS----GYKGE-KTIVINELDA-SGMTFRELFQVLEPDS

---ATQLSSRFKDAY-I---INDLTIITNSDSYWDWCDSW------KNKEYHQLMR----

--RIRFVIKM-EKNKKIELWNYTATRDLKEKSKLETY--TLKSV---ENEEEF-RKIASN

IL-------KKINKNINTK-K-DQSKGS

>pCRESS5|WP_049478725.1

MK---INQNDR--KRKFFGQI--SYEYEN---KTEKDFRESVIQRI-H--DYC-KNEEDR

YHI-IFHDKDLKDDGSSK--PLHAHFYIDFKHAHTYSSVLKALSIS----RKQ-NLEF-V

RS-SIKACRYLTHRNERNEH-KFPYEVADVISSENANYIDDI-M--GKI-KNHS-KEKAD

EIDDY-CLELSYL-ISEEGL-LTTEAFE---Q-FTQKAWNSNKRQFEE-NRQEF------

-----------I-QKEF----IRMSKGER-----NHTGIYIQAEGGTGKSYLARLLAEE-

HDRLG--AH--T---PSID-LGS----GYKGE-KTIVINELDA-SGMTFRELFQILEPDS

---ATQLSSRFKDAY-I---INDLTIITNSDTYWDWCDSW------KNKEYHQLMR----

--RIRFVIKM-DKNKNIELWYYNANRDLKEKAKIQTY--TLSSV---KNEEEF-RKIASD

IL-------EKINNNIDNKRK-DQSKGS

>pCRESS5|WP_067193806.1

MAK--IDRNAR--KRKYFGQI--SYDYEN---STEDDFRKSVEQRI-K--ELC-QHENDK

YYF-IFHDKDLKDDGTPK--PLHAHFYIEFKNPRVYSTVYKALQIS----RQE-NLEF-V

RS-TIKACRYLTHRNERNES-KFPYEVSEVIASKNGTYIDDI-M--GKV-KNHS-VEKSD

EVSDY-CLELSYK-ISEDGL-LPSEAFE---Q-FTQKAWNSNKRQFED-NRQEY------

-----------I-QKEF----IRMSKGER-----HHNGIFIFAEGGTGKSFLSRLLAEE-

HDRLG--AH--T---PSID-LGS----GYQGQ-KTIIINELDA-TGMAYRELFQILEPDS

---ANQLSSRFKDAY-I---INDLTIMTNSESYWSWCNSW------NKKEYHQLMR----

--RIRFVIKM-DTNQIIELWHYYATRDLKEKARIDTF--HLKDI---QDEEAF-RNIASN

IL-------KKIQTLTPQK---DQSETT

>pCRESS5|WP_044771983.1

MA--AIKPTDR--KRKFFGQI--SYDYEN---STEVDFKQSVEKRI-K--ALC-SNQEDK

FYI-IFHDKDQKDDGTPK--PLHAHFYIDFKNPRAYSAVFKALSIS----RQE-NLEA-V

RS-SIKACRYLTHRNERNEH-KFPYEVSEVIQSPNGNYIDDI-M--GEI-KHHS-KEKSD

EVDDY-CLELSFQ-ISSEGL-LPLEAFE---Q-FTQKAWNQNKRQFEE-NRQEY------

-----------I-QKEF----ERMSRGER-----NHNSIYIQGDGNSGKSFLARLIAEQ-

HDRFG--AH--T---PSID-LGS----GYKGQ-KTMIINEFDA-SGMVYRELFQILEPNS

---ANQLSSRFKDAY-T---INDLTIITNSENYWDWVDDW------KKKEYHQLMR----

--RIRYVIKM-HQNKNIELWYYHGIRDLKEKSKIKDW--MLDPVT-NDSEPEL-TMLAQD

IL-------SEIQQFNHKQIK-DHSDSN

>pCRESS5|WP_024408358.1

MVK-PIDPNAR--KRKYFGQI--SYEYEN---NTEDDFKQSVEKRI-K--ELC-QNDDDV

FYY-IYHDKDINEDGTPK--YLHVHFVIIFKNAHSYQSVYNALKIS----RQE-NLEV-V

RS-SIKACRYLTHRNERNEG-KYPYSVEEVIQSSNGNYINSI-M--GKI-KNHS-TEKSE

EVDEY-CIELSYL-ISSDGL-LPQEAFE---Q-FTQKAWNTNKRTFEE-NRLEY------

-----------I-QQEF----EHMSRGER-----NHNGIYIQGDGNSGKSFLARLIAEQ-

HDRLA--AH--V---PSID-LGS----GYKGQ-KTMIINEFDA-SGMAYRELFQILEPDS

---ANQLSSRFKDAY-I---INDMTIITNSESYWDWVDDW------KKKEWHQLMR----

--RIRYVIKM-DQNKIIELWYYHAIRDLKEKAKIKEW--KLDSIT-KDSEPEL-TTVAQE

IL-------TEIQQANSNQIK-DHSDSN

>pCRESS5|WP_039694423.1

MTK-NIDPNAR--KRKYFGQI--SYEYEN---NTEDDFKKSVEKRI-K--EYC-QNDEDT

FYI-IFHDKDTNNDGTLK--TLHAHFYIDFKNARPFSTVFNALKIS----RQE-NLEF-V

RS-SIKACRYLTHRNERNEG-KYPYSVEEVIQSSNGDHINSI-M--GKI-KNHS-KEQSE

EVDDY-CLELSYQ-ISSEGL-LPLEAFE---Q-FTQKAWNQNKRQFEE-NRQEY------

-----------I-QKEF----ERMSRGER-----NHNSIYIQGDGNSGKSFLARLIAEQ-

HDRLG--AH--T---PSID-LGS----GYKGQ-KTMIINEFDA-SGMAYRELFQILEPDS

---ANQLSSRFKDAY-I---INDLTIMTNSEKYWDWVDDW------TKKEYHQLMR----

--RIRYIIKM-NHNKIIELWYYHAIRDLKEKAKIKEW--KLDSIT-KDSEPEL-TTVAQE

IL-------TEIQQANSNQIK-KQSDSN

>pCRESS5|WP_033583888.1

MVK-AIDPNTR--KRKFFGQI--SYEYEN---KKECDFKKSIDKRI-K--ALC-KHDEDV

YYY-IYHDKDKKEDGTPK--CLHVHFVIIFKHAHSYQSVYKSLQIS----REE-NLEF-V

RS-VIKACRYLTHRNERNEG-KYPYNVDEVIQSPNGNYINSI-T--GEI-KNHN-KEQSD

EVDEY-CLDLSYQ-ISSDGL-LPLEAFE---Q-FTQKAWNQNKRQFEE-NRLEY------

-----------I-QKEF----ERMSRGER-----NHNGIYIQGSGNSGKSFLARLIAEQ-

HDRLG--AH--I---PSID-LGS----GYKGQ-KTMIINEFDA-SGMSYRELFQILEPNS

---ANQLSSRFKDAY-I---INDLTIMTNSESYFDWVDAW------KKREYHQLMR----

--RIRYILKM-DYNKNIELWYYNSIRDLKEKAKIKVW--KLEPIT-KDSEDEL-TLIAQE

LL-------AEIQHINSKQTK-DQSDSN

>pCRESS5|WP_049481849.1

MVK-AIDPNTR--KRKFFGQI--SYEYEN---KKECDFKKSIDKRI-K--ALC-KHDEDV

YYY-IYHDKDKKEDGTPK--CLHVHFVIIFKHAHSYQSVYKSLQIS----REE-NLEF-V

RS-VIKACRYLTHRNERNEG-KYPYNVDEVIQSPNGNYINSI-T--GEI-KNHN-KEQSD

EVDEY-CLDLSYQ-ISSDGL-LPLEAFE---Q-FTQKAWNQNKRQFEE-NRLEY------

-----------I-QKEF----ERMSRGER-----NHNGIYIQGSGNSGKSFLARLIAEQ-

HDRLG--AH--V---PSID-LGS----GYKGQ-KTMIINEFDA-SGMSYRELFQILEPNS

---ANQLSSRFKDAY-I---INDLTIMTNSESYFDWVEAW------KKREYHQLMR----

--RIRYILKM-DYNKNIELWYYHSICDLKEKAKIKVW--KLEPIT-KDSEDEL-TLIAQE

LL-------AEIQHINSKQTK-DQSDSN

>pCRESS5|WP_029171254.1

MVK-AIDPNIR--KRKFFGQI--SYEYEN---KTEDDFKKSVEKRI-K--EYC-QHDEDT

YYV-IFHDKDINDEGTLK--PLHAHFYIEFKNARPFSPVYKALKIS----REE-NLEF-V

RS-SIKACRYLTHRNERNEG-KYPYNVDEVIQSSNGNYIEDI-M--GEI-KNHS-KEKSD

EVDDY-CLELCYQ-ISSEGL-LPEEAFE---Q-FTQKAWNTNKRQFEE-NRQEY------

-----------I-QQEF----ERMSRGER-----NHNGIYIHGLGNSGKSFLARLIAEQ-

HDRLG--AH--T---PSID-LGS----GYKGQ-KTIIINEFDG-SGMAYRELFQILEPNS

---ANQLSSRFKDAY-I---INDLTIMTNSETYWDWVDGW------KKREYHQLMR----

--RIRYVVKM-DQNKIIEMWYYHAIRDYKEKAKVKEW--TLNSIT-KDSEIEL-TIVAQE

LL-------AELQHTNNKQTK-DQSDSN

>pCRESS5|WP_053863690.1

-MA--DLSGVK--ARRYIAQV--SYTYQN---KTEDAFREKIIKLV-K--ALC-THDDDK

FYI-IFHDKDIDQNGELK--SLHAHIYFEFKNSRFYSSLFKTLEIS----RDK-NLQV-V

KD-KAKVCRYLTHRNEKDEG-KYQYEISEIISSKNSDYRLDI-C--GKS-KRAD-KKKSE

EEVDC-CLEISYQ-ICDQGL-LLNQAFE---E-FDQQLWNRNKKQFEI-NRQEY------

-----------I-DKEF----AHMSKGNR-----NHIGLYIQGSGGTGKTTLARFLAEE-

RDEHG--QH--A---PSTD-LGS----GYDGQ-RTIVINEFDA-SGISFRELFQILEPDA

---VTQLSSRFKDAH-I---INDLTIMTNSDDFIEWADAW------KKKEYHQLMR----

--RIPFFVKL-N-GKIAELYHYKATKDKGDKCKVKSY--NLGKT---LDEEHL-RKQAKK

LL-------SDIRHQKKIKKA-DQSEKR

>pCRESS5|WP_014735272.1

-MA--DLSKVK--ARRYIAQV--SYTYQN---NTEDAFREKIIRLV-K--ALC-THDDDK

FYI-IFHDKDIDQNGELK--SLHAHIYFEFKNSRFYSSLFKTLEIS----RDK-NLQV-V

KD-KAKVCRYLTHRNEKDEG-KYQYEISEIISSKNSDYRLDI-C--GKS-KRAD-KKKSE

EEIDC-YLEISYQ-ICEQGL-LLNQAFE---E-FDQQLWNRNKKQFEI-NRQEY------

-----------I-DKEF----VRMSKGNR-----NHIGLYIQGSGGTGKTTLARFLAEE-

RDEHG--QH--A---PSTD-FGS----GYDGQ-RTIVINEFDA-SGISFRELFQILEPDA

---VTQLSSRFKDAH-I---INDLTIMTNSDDFIEWADAW------KKKEYHQLMR----

--RIPFFVKL-N-GKIAELYHYKATKDNGDKCKVKSY--NLRKT---LDEEHL-RKQAKK

LL-------SDIRHQKKIKKA-DQSEKR

>pCRESS5|WP_018166163.1

-MA--DLSEVK--ARRYIAQV--SYTYQN---DTEEAFREKIIRLV-K--TLC-THDEDK

FYI-IFHDKDLDENGELK--SLHAHIYFEFKNSRFYSSLFKTLEIS----REK-NLQV-V

KD-KAKVCRYLTHRNEKDEG-KYQYEISEIISSQNADYRLDI-C--GKS-KKAD-NRKSE

EETDF-CLEISYQ-ISECGL-LLKQAFE---E-FDQQLWNRYKKQFEI-NRQEY------

-----------I-DKEF----IRMSKGSR-----NHIGLYIEGSGGTGKTTLARFLAEE-

RDEHG--QH--A---PSTD-FGS----GYDGQ-RTIVINEFDA-SGISFRELFQFLEPDA

---VTQLSSRFKDAH-I---INDLTIMTNSDDFIEWADAW------KKKEYHQLMR----

--RIPFFVKL-N-GKIAELYHYKATKDKGDKCKVKSY--NLGKT---LDEEHL-RKQAKK

LL-------SDIRHQKKIKKA-DQSTKR

>pCRESS5|WP_024382134.1

-MA--DLSEVK--ARRYIAQV--SYAYQN---DTEEAFREKIIRLV-K--ALC-THDDDK

FYI-IFHDKDIDQNGELK--SLHAHIYFEFKNSRFYSSLFKTLEIS----RDK-NLQV-V

KD-KAKVCRYLTHRNEKDEG-KYQYEISEIISSKNNDYRLDI-C--GKS-KRAD-KRKSE

EEIDF-CLEISYQ-ISECGL-LLNPAFE---E-FDQQLWNRYKKQFEI-NRQEY------

-----------I-DKEF----IRMSKGNR-----NHIGLYLEGSGGTGKTTLARFLAEE-

RDEHG--QH--A---PSTD-LGS----GYDGQ-RTIVINEFDA-SGISYRELFQILEPDA

---VTQLSSRFKDAH-I---INDLTIMTNSDDFIEWADAW------KKKEYHQLMR----

--RIPFFVKL-N-GKIAELYHYKATKDQGDKCKVKTY--NLGKT---LDEEHL-RKQAKK

LL-------SDIRHQKKIKKA-DQSTKR

>pCRESS5|WP_024399566.1

-MA--DLSEVK--TRRYIAQV--SYAYQN---DTEEAFREKIIRLV-K--MLC-THDEDK

FYI-IFHDKDLDENGELK--SLHAHIYFEFKNSRFYSSLFKTLEIS----REK-NLQI-V

KD-KARVCRYLTHRNEKDEG-KYQYEISEIISSKNSDYRLDI-C--GKS-KRAD-KRKSE

EEIDF-CLEISYQ-ISECGL-LLNPAFE---E-FDQQLWNRYKKQFEI-NRQEY------

-----------I-DKEF----IRMSKGNR-----NHIGLYIEGSGGTGKTTLARFLAEE-

RDEHG--QH--A---PSTD-LGS----GYDGQ-RTIVINEFDA-SGISFRELFQILEPDA

---VTQLSSRFKDAH-I---INDLTIMTNSDDFINWTDSW------KKKEYHQLMR----

--RIPFFIKL-N-SNVAELYHYKAAKDIGDKCKVKTY--NLGKT---LDEEHL-RKQAKK

LL-------SDIRHQKKIKKA-DQSTKR

>pCRESS5|WP_024389873.1

-MA--DLSEVK--ARRYIAQV--SYAYQN---DTEEAFREKIIRLV-K--ALC-THDDDK

FYI-IFHDKDIDQNGELK--SLHAHIYFEFKNSRFYSSLFKTLEIS----RDK-NLQV-V

KD-KAKVCRYLTHRNEKDEG-KYQYEISEIISSKNNDYRLDI-C--GKS-KRAD-KRKSE

EEIDF-CLEISYQ-ISECGL-LLNPAFE---E-FDQQLWNRYKKQFEI-NRQEY------

-----------I-DKEF----IRMSKGNR-----NHIGLYLEGSGGTGKTTLARFLAEE-

RDEHG--QH--A---PSTD-LGS----GYDGQ-RTIVINEFDA-SGISYRELFQILEPDA

---VTQLSSRFKDAH-I---INDLTIMTNSDDFIEWADAW------KKKEYHQLMR----

--RIPFFVKL-N-GKIAELYHYKATKDKGDKCKVKTY--NLGKT---LDEEHL-RKQAKK

LL-------SDIRHQKKIKKA-GKTTVE
