# Supplementary material for: Multiple origins of prokaryotic and eukaryotic single-stranded DNA viruses from bacterial and archaeal plasmids
Source: Nat Commun. 2019 Jul 31;10:3425. doi: 10.1038/s41467-019-11433-0 (PMC6668415; doi:10.1038/s41467-019-11433-0)
Supplement: Supplementary file 5 — Dataset 4 [file 41467_2019_11433_MOESM5_ESM.docx]

**SUPPLEMENTARY DATA 4**

# PHYLOGENETIC TREE SHOWN IN FIGURE 2A

(((((CVH76026.1|Coriobacteriaceae_bacterium_CHKCI002:1.2462595,((WP_026669310.1|Butyrivibrio_sp._AE3006:0.0844974,WP_026524352.1|Butyrivibrio_sp._MB2005|ButMB-E1:0.07362837):0.7250273[1.00],((WP_053982727.1|Lachnospiraceae_bacterium_mt14:0.8614078,((WP_000032131.1|Streptococcus_oralis|StrOra-E1:0.11497694,(WP_003102166.1|Streptococcus_iniae|StrIni-E1:0.17186522,WP_029176105.1|Streptococcus_suis:0.153752):0.06863574[0.91]):0.33174688[1.00],(WP_029694263.1|Streptococcus_suis|StrSui-E1:0.31564766,(WP_062004798.1|Streptococcus_cristatus:0.21685845,(WP_003030931.1|Streptococcus_anginosus|StrAng-E1:0.07834427,(WP_029690610.1|Streptococcus_hyovaginalis:0.19640572,WP_047207334.1|Streptococcus_agalactiae:0.14229678):0.04120362[0.91]):0.20734215[1.00]):0.12089285[0.99]):0.18351784[0.99]):0.54290444[1.00]):0.20202683[0.67],(CDF01935.1|Ruminococcus_sp._CAG_624:0.89316773,(CUO57637.1|Fusicatenibacter_saccharivorans:0.4396852,CUO23215.1|Coprococcus_comes:0.313332):0.18355237[1.00]):0.09346383[0.74]):0.29488856[1.00]):0.22409002[0.99]):0.29405794[0.99],((WP_051546484.1|Clostridium_viride|CloVir-E1:0.89069945,(WP_066546553.1|Clostridium_sp._AT5:0.2656437,WP_013271491.1|Clostridium_saccharolyticum:0.27689424):0.90710485[1.00],((WP_051600858.1|Eubacterium_sp._AB3007:0.8003177,(WP_013978550.1|Eggerthella_sp._YY7918:0.6326405,(WP_021882760.1|Ruminococcus_sp._CAG_108|RumCAG-E1:0.5259402,(CBL15233.1|Ruminococcus_bromii_L2-63:0.53909534,(WP_009301216.1|Coprobacillus_sp._3_3_56FAA:0.9409981,(CCZ45692.1|Firmicutes_bacterium_CAG_129:0.26702493,(WP_044942941.1|Flavonifractor_plautii:0.08527608,WP_021629801.1|Clostridium_sp._ATCC_BAA-442:0.04347396):0.16137727[1.00]):0.22696394[0.99]):0.08201889[0.81]):0.08619107[0.89]):0.11300647[0.99]):0.1322282[0.99]):0.21814741[1.00],((SCH60086.1|uncultured_Ruminococcus_sp.:0.66472864,WP_036328238.1|Mollicutes_bacterium_HR2|MolHR2-E1:0.87694913):0.4180182[1.00],((WP_037404274.1|Solobacterium_moorei|SolMoo-E1:0.6021121,(WP_044928503.1|Roseburia_inulinivorans|RosInu-E1:0.47565517,((WP_018597672.1|Blautia_producta:0.34961087,(SCH17786.1|uncultured_Clostridium_sp.:0.23063566,(CUP05665.1|Hungatella_hathewayi|HugHat-E1:0.27250427,CDC44519.1|Clostridium_sp._CAG_58:0.39820483):0.09122113[0.96]):0.21662173[1.00]):0.03017031[0.80],(WP_038350939.1|Eubacterium_limosum:0.3000974,(KJZ87129.1|Clostridium_sp._IBUN125C:0.21404985,WP_023977019.1|Clostridium_pasteurianum:0.22277017):0.16541785[1.00]):0.049837[0.69],BAK32345.1|Erysipelothrix_rhusiopathiae_str._Fujisawa|EryRhu-E1:0.527389):0.09245015[0.99]):0.09244064[0.99]):0.0814097[0.99],CDE72464.1|Acidaminococcus_sp._CAG_917:0.5109951,(CDB27189.1|Oscillibacter_sp._CAG_241:0.62194604,(WP_053167095.1|Planomicrobium_glaciei:0.47666618,WP_020072285.1|Clostridium_sporosphaeroides:0.7528274):0.11591842[0.97]):0.07532621[0.72]):0.06996642[0.99],(((WP_024346025.1|Clostridium_methoxybenzovorans|CloMet-E1:0.16068275,WP_038278663.1|Clostridium_celerecrescens|CloCel-E1:0.19300102):0.25464228[1.00],((WP_009246639.1|Lachnospiraceae_bacterium_6_1_63FAA|LacBac6.1-E1:0.2736556,CCY69022.1|Eubacterium_sp._CAG_161:0.238866):0.16127361[1.00],(EES75484.2|Ruminococcus_sp._5_1_39BFAA|Rum51-E1:1.0455546,(WP_052011064.1|bacterium_LF-3:0.39001822,CCX75435.1|Dorea_sp._CAG_105:0.29611942):0.13745286[0.99]):0.07914074[0.81]):0.10734703[0.99]):0.15278018[1.00],((WP_066550639.1|Clostridium_sp._AT5:0.3826408,(WP_007865724.1|Clostridium_citroniae|CloCit-E1:0.0971273,WP_013270924.1|Clostridium_saccharolyticum:0.09794088):0.37920418[1.00]):0.17993203[0.99],WP_051639324.1|Lachnospiraceae_bacterium_AC2028:0.71946955):0.07220477[0.77]):0.12295884[1.00]):0.05619566[0.96]):0.08571264[0.98]):0.19342329[0.99],(WP_033495900.1|Bifidobacterium_biavatii:1.5552081,(WP_022856850.1|Alloscardovia_omnicolens|AllOmni-E1:0.97590196,(((KFI87454.1|Bifidobacterium_ruminantium:0.8103481,WP_052119337.1|Bifidobacterium_callitrichos:1.17626):0.21779266[0.97],(WP_055838650.1|Microbacterium_sp._Leaf436:1.0536375,(WP_036342632.1|Mobiluncus_mulieris|MobMul-E1:1.1742166,(WP_016667133.1|Propionibacterium_sp._HGH0353:0.6706672,WP_002529618.1|Propionibacterium_acnes|ProAcn-E1:0.67418844):0.15116324[0.87]):0.2210267[0.99]):0.26568627[0.99],((WP_023022037.1|Corynebacterium:0.64311475,AKO38848.1|Haemophilus_ducreyi:0.9113895):0.26302505[0.99],(NP_613078.1|Bifidobacterium_pseudocatenulatum|p4M:0.77272826,WP_025221073.1|Bifidobacterium_breve:1.0276467):0.2834783[0.99]):0.1605993[0.97]):0.11141899[0.99],(WP_021975256.1|Bifidobacterium|BifLon-E1:0.5172128,(WP_052825216.1|Bifidobacterium_animalis:0.3768805,(KFI81686.1|Bifidobacterium_pullorum:0.25570372,WP_043170238.1|Bifidobacterium_pullorum|BifPul-E1:0.1806415):0.20644975[1.00]):0.14804335[0.99]):0.23014177[1.00]):0.2352102[1.00]):0.13385648[0.88]):0.41378197[1.00]):0.33353773[1.00]):0.65899473[0.99],(((WP_000818357.1|Streptococcus_agalactiae:0.69618714,WP_000186194.1|Streptococcus_agalactiae:0.58762187):0.27825165[0.99],((WP_017824301.1|Brachybacterium_muris:0.2904825,CEI31812.1|Propionibacterium_freudenreichii:0.3851118):0.39712226[1.00],(((CDA18875.1|Ruminococcus_sp._CAG_488|RumCAG-E1:0.92504257,(WP_021639163.1|Clostridium_sp._KLE_1755:0.5125839,(CBL40434.1|butyrate-producing_bacterium_SS3/4:0.7619091,CRY93789.1|uncultured_prokaryote|pRGRH0065:0.38645494):0.1015349[0.98]):0.08232089[0.97]):0.13032243[0.99],WP_007889993.1|Roseburia_inulinivorans:0.7530716,CRY97508.1|uncultured_prokaryote|pRGFK1613:0.83048916):0.21997136[1.00],(WP_044572803.1|Arthrobacter_sp._A3:0.6517754,((WP_006681830.1|Actinomyces_turicensis:0.00380489,WP_052038917.1|Actinomyces_sp._S4-C9:0.03148221):0.5515397[1.00],(GAC78794.1|Gordonia_malaquae_NBRC_108250|GorMal-E1:0.62608457,(WP_043534193.1|Actinomyces_sp._MS2:0.09383254,(WP_005464724.1|Saccharomonospora_glauca:0.0136657,WP_067940518.1|Actinomyces_radicidentis:0.10188445):0.03109042[0.99]):0.16883156[0.99]):0.5713247[1.00]):0.1788987[0.99]):0.20168094[1.00]):0.11147343[0.95]):0.15196542[0.83]):0.92921317[1.00],(((((WP_018166163.1|Streptococcus_minor:0.03297033,(WP_024399566.1|Streptococcus_suis|StrSui-E1:0.04269813,(WP_024382134.1|Streptococcus_suis:0.00275414,WP_024389873.1|Streptococcus_suis:1.5E-7):0.01068712[0.99]):0.00672834[0.71]):0.02780538[0.99],WP_014735272.1|Streptococcus_suis:0.01028209,WP_053863690.1|Streptococcus_suis:0.01814987):0.41233927[1.00],((WP_044771983.1|Streptococcus_suis:0.11631367,((WP_024408358.1|Streptococcus_suis:0.05508335,(WP_033583888.1|Streptococcus_sp._SR1|StrSR1-E1:0.00452029,WP_049481849.1|Streptococcus_constellatus:0.00460286):0.13544719[1.00]):0.03814753[1.00],(WP_039694423.1|Streptococcus_gallolyticus:0.06337939,WP_029171254.1|Streptococcus_suis:0.12811232):0.01493011[0.96]):0.0433147[0.99]):0.1301701[1.00],(WP_067193806.1|Streptococcus_sp._DD10:0.14039879,(WP_049535277.1|Streptococcus_pseudopneumoniae:0.03052284,(WP_049478725.1|Streptococcus_oralis|StrOra-E1:0.06442908,(WP_061417941.1|Streptococcus_oralis:0.01122744,WP_061863770.1|Streptococcus_infantis:0.03392991):0.02030284[0.99]):0.02445653[0.96]):0.15456866[1.00]):0.05915011[0.99]):0.2042549[0.99]):0.79688996[1.00],(WP_058211405.1|Lactococcus_lactis:0.4971042,WP_017368666.1|Lactococcus_garvieae|LacGar-E1:0.26374224):0.8759256[1.00],((WP_038978316.1|Paenibacillus_polymyxa:0.5823269,WP_046467524.1|Staphylococcus_pasteuri:0.69820166):0.6821785[1.00],(WP_061866456.1|Streptococcus_oralis:0.9159468,((WP_024393234.1|Streptococcus_suis:0.22012718,WP_050238550.1|Streptococcus_pneumoniae:0.1627617):0.6320498[1.00],(WP_024390948.1|Streptococcus_suis:0.2827531,WP_029176301.1|Streptococcus_suis:0.23806582):0.5916529[1.00]):0.22460829[0.95]):0.48848784[1.00]):0.1374237[0.90]):0.7116748[1.00],(((WP_061343647.1|Enterococcus_faecium|EntFae-E1:0.46887377,WP_017371219.1|Lactococcus_garvieae:0.5150723):0.5461056[1.00],(((WP_039670385.1|Streptococcus_macedonicus:0.2830289,(WP_018030886.1|Streptococcus:0.20730361,WP_003104234.1|Streptococcus_parauberis:0.14389573):0.13556632[1.00]):0.2620432[1.00],(((WP_025016923.1|Lactococcus_lactis:0.10967388,KST89836.1|Lactococcus_lactis_subsp._lactis:0.0986995):0.48897326[1.00],(((WP_003048523.1|Streptococcus_canis:0.05736175,WP_039694464.1|Streptococcus_gallolyticus:0.13123694):0.05684453[1.00],(WP_044671103.1|Streptococcus_suis:0.12859245,WP_000746010.1|Streptococcus_agalactiae:0.1587748):0.03819638[0.99]):0.17872833[1.00],(WP_018380019.1|Streptococcus_thoraltensis:0.14472626,EOB33201.1|Streptococcus_mitis_13/39:0.22319272):0.10498288[1.00]):0.19100149[1.00]):0.09439493[0.95],((WP_032941943.1|Lactococcus_lactis|LacLac-E1:0.05568699,WP_058223604.1|Lactococcus_lactis:0.03507739):0.45239916[1.00],(WP_019299400.1|Lactococcus_garvieae:0.16163538,BAM66968.1|Lactococcus_lactis:0.14670157):0.4290371[1.00]):0.18212534[1.00]):0.06191982[0.68]):0.33678305[1.00],(WP_014571792.1|Lactococcus_lactis:1.03779,(AEU41945.1|Lactococcus_lactis_subsp._cremoris_A76|pQA504:0.7892392,(((ADX23728.1|Streptococcus_dysgalactiae_subsp._equisimilis_ATCC_12394:0.49681595,(WP_056938517.1|Lactobacillus_suebicus:0.5708253,((WP_034704841.1|Enterococcus_gallinarum:0.1088482,WP_067483596.1|Enterococcus_thailandicus:0.06699768):0.25383225[1.00],(WP_003024533.1|Streptococcus_anginosus:0.3083217,((WP_017649267.1|Streptococcus_agalactiae:0.11047273,ABJ73998.1|Lactococcus_lactis_subsp._cremoris_SK11:0.21467315):0.10561527[1.00],((WP_049476139.1|Streptococcus_intermedius:0.11189705,WP_003035134.1|Streptococcus_anginosus:0.05655064):0.1005593[1.00],((KEQ49321.1|Streptococcus_oralis:0.14180341,WP_053092713.1|Streptococcus_oralis:0.2026465):0.07398209[1.00],(((WP_015647385.1|Streptococcus_suis:0.24846198,(WP_032497992.1|Streptococcus_suis:0.24023215,KXT86702.1|Streptococcus_oralis:0.08073865):0.03802786[0.99]):0.02248151[0.76],(WP_014623544.1|Streptococcus_equi:0.13463043,CGE81062.1|Streptococcus_pneumoniae:0.29846615):0.03142273[0.94]):0.03527077[0.99],(WP_027972054.1|Streptococcus_plurextorum:0.2026266,((WP_039677656.1|Streptococcus_constellatus:0.28330004,(WP_049499636.1|Streptococcus_parasanguinis:0.1107073,WP_045759092.1|Streptococcus_parasanguinis:0.08250625):0.16019116[1.00]):0.0508481[0.99],(WP_044774450.1|Streptococcus_suis:0.06301846,WP_020997784.1|Streptococcus_constellatus:0.09626952):0.19496918[1.00]):0.03759937[0.69]):0.03131919[0.99]):0.05311603[1.00]):0.05941268[1.00]):0.06138456[0.99]):0.07481198[0.99]):0.07717327[0.99]):0.15790291[1.00]):0.07919132[0.67]):0.18092147[1.00],((WP_044762265.1|Streptococcus_suis:0.12894256,(WP_018376545.1|Streptococcus_orisratti:0.11309987,WP_020999261.1|Streptococcus_intermedius:0.13908274):0.07167196[0.98]):0.5541625[1.00],((WP_000044268.1|Streptococcus_sp._SK140:0.23954864,(CMU27730.1|Streptococcus_pneumoniae:0.25151038,(WP_000201649.1|Streptococcus_sp._C300:0.0633828,WP_047206721.1|Streptococcus_agalactiae:0.05048267):0.1309712[1.00]):0.11616882[1.00]):0.12556727[1.00],((WP_024400359.1|Streptococcus_suis|StrSuiYS39-E1:0.16316354,(WP_004183001.1|Streptococcus_salivarius:0.10142858,(WP_001034312.1|Streptococcus_agalactiae:0.0629846,WP_024385235.1|Streptococcus_suis:0.08097888):0.02324086[0.99]):0.0243543[0.96]):0.21038902[1.00],(WP_000791389.1|Streptococcus_sp._oral_taxon_071:0.24403945,WP_003032217.1|Streptococcus_anginosus_group:0.49212348):0.04848702[0.93]):0.05511009[0.94]):0.14629541[1.00]):0.15757985[0.99]):0.3293672[1.00],((WP_051176704.1|Propionimicrobium_lymphophilum:0.8173165,(WP_036321578.1|Microbacterium_gubbeenense:0.38173008,(WP_054952722.1|Flaviflexus_sp._SIT4:0.02602765,WP_041290927.1|Kytococcus_sedentarius:0.03231581):0.19868349[1.00]):0.30624977[1.00]):0.29077384[1.00],(WP_052506726.1|Streptococcus_suis:0.7376445,WP_022765681.1|Butyrivibrio_sp._XPD2006|ButXPD-E1:0.81477207):0.11609328[0.94]):0.13770995[0.83]):0.26474044[1.00]):0.13316545[0.91]):0.24484546[0.99]):0.23640423[0.96]):0.7628136[1.00],(((WP_002578150.1|Clostridium_bolteae|CloBol-E1:0.7724039,CUN62864.1|Hungatella_hathewayi:0.71170855):0.26815805[0.99],(WP_019282500.1|Vibrio_anguillarum|VibAng-E1:0.77659625,((CCZ93342.1|Coprococcus_eutactus_CAG_665:0.44397676,CCY61699.1|Clostridium_sp._CAG_264:0.36014074):0.24775448[1.00],(((KXT29039.1|Candidatus_Phytoplasma_oryzae:0.50907135,(YP_001966814.1|Candidatus_Phytoplasma_australiense|pCPa:0.3339602,(WP_011161011.1|Onion_yellows_phytoplasma|OniYel-E1:0.0630811,(ABC65805.1|Aster_yellows_witches-broom_phytoplasma_AYWB|pAYWB-IV:0.11422873,(ABC65794.1|Aster_yellows_witches-broom_phytoplasma_AYWB|pAYWB-II:0.21214354,(YP_006961027.1|Periwinkle_leaf_yellowing_phytoplasma|p09PLY-1:0.0500456,YP_003617079.1|Rehmannia_glutinosa_phytoplasma|pPARG1:0.06299382):0.15890573[1.00]):0.06863856[0.81]):0.06166513[0.99]):0.14844376[1.00]):0.12570424[0.99]):0.31781638[1.00],(CCZ68460.1|Ruminococcus_gnavus_CAG_126:0.5849396,SCG87263.1|uncultured_Clostridium_sp.:0.6710189):0.36579746[1.00]):0.22112042[0.99],ODR34583.1|Eisenbergiella_tayi:0.5107497):0.16933294[0.99],(WP_028509833.1|Ruminococcus_sp._NK3A76:0.8773984,CDE19587.1|Acidiphilium_sp._CAG_727|AciCAG-E1:1.0591805):0.29822508[0.99]):0.15484113[0.91]):0.16516545[0.97]):0.5375683[1.00],(WP_062359070.1|Weissella_sp._DD23:0.8369665,((((AKG47101.1|Lactobacillus_vaginalis|pC107:1.1249478,((WP_046025501.1|Lactobacillus_fermentum:0.33893183,WP_034540695.1|Lactobacillus_mucosae:0.20625608):0.7681069[1.00],(KRN07545.1|Lactobacillus_sucicola_DSM_21376_=_JCM_15457:0.7841506,CUR41281.1|Lactobacillus_reuteri:0.878588,(WP_046923918.1|Lactobacillus_ruminis:0.9296714,(WP_003665528.1|Lactobacillus_reuteri|LacReu-E1:0.74275434,WP_006499656.1|Lactobacillus_mucosae|LacMuc-E1:0.741937):0.09741035[0.67]):0.10119307[0.89]):0.15410388[0.99]):0.13369998[0.95]):0.1514263[0.99],(WP_002821392.1|Oenococcus_oeni|OenOen-E1:1.0867591,(WP_057827085.1|Lactobacillus_aviarius:0.6428371,(WP_057906729.1|Lactobacillus_aviarius:0.11228075,WP_057827851.1|Lactobacillus_aviarius:0.15886834):0.7117703[1.00]):0.16619352[0.98]):0.20668381[0.99]):0.13793866[0.99],((WP_004900270.1|Leuconostoc_citreum:0.78439605,(WP_036093565.1|Leuconostoc_mesenteroides|LeuMes-E1:1.4E-7,EEJ43069.1|Leuconostoc_mesenteroides_subsp._cremoris_ATCC_19254:1.0E-8):0.68755335[1.00]):0.3073308[1.00],(ABP89830.1|Streptococcus_suis_05ZYH33:1.1059926,((((CYX46115.1|Streptococcus_suis:0.13847616,CYW87437.1|Streptococcus_suis:0.0607883):0.09074505[0.99],(WP_024410839.1|Streptococcus_suis:0.05605554,(WP_049523992.1|Streptococcus_pseudopneumoniae:0.16931172,(WP_033683822.1|Streptococcus_mitis:0.01528764,EFO53527.1|Streptococcus_infantis_SK1302:0.02168969):0.09091356[0.99]):0.2894969[1.00]):0.12937872[0.99]):0.43834227[1.00],(WP_051448806.1|Viridibacillus_arenosi|VirAre-E1:0.26519233,(WP_050444210.1|Enterococcus_faecalis:0.08627446,WP_010817837.1|Enterococcus:0.05594319):0.2147566[1.00]):0.45570916[1.00]):0.2713552[1.00],(WP_000093566.1|Streptococcus_mitis:1.4E-7,(WP_042900192.1|Streptococcus_mitis|StrMit-E1:1.0E-8,WP_050492321.1|Streptococcus_mitis:0.00572045,KXA58447.1|Streptococcus_mitis:0.00283125):0.00567997[0.80]):0.8487617[1.00],(WP_016226904.1|Lachnospiraceae_bacterium_10-1:0.41471905,SCH55298.1|uncultured_Collinsella_sp.:0.6794287):0.26425818[1.00]):0.08643587[0.89]):0.06505941[0.92]):0.14882895[0.99]):0.10856646[0.84],(((((CDA26462.1|Lactobacillus_amylovorus_CAG_719:0.13640463,(WP_003549058.1|Lactobacillus_acidophilus:0.07447135,KRN00682.1|Lactobacillus_taiwanensis_DSM_21401|LacTai-E1:0.06058201):0.1226822[1.00]):0.03574209[0.87],((CDI42894.1|Lactobacillus_helveticus_CIRM-BIA_953:0.2956298,WP_012845653.1|Lactobacillus_johnsonii:0.24036862):0.12694706[1.00],CDI43023.1|Lactobacillus_helveticus_CIRM-BIA_953:0.12240241,KRK41125.1|Lactobacillus_amylovorus_DSM_20531:0.2307495):0.05993262[0.94]):0.54300404[1.00],((WP_014567781.1|Lactobacillus_johnsonii:0.44769445,(WP_007125042.1|Lactobacillus_ultunensis:0.17222835,WP_060461663.1|Lactobacillus_crispatus:0.19595657):0.13540287[0.99]):0.22601262[1.00],((WP_049150683.1|Lactobacillus_gasseri:0.40145606,WP_046324376.1|Lactobacillus:0.391372,(WP_008472153.1|Lactobacillus_gigeriorum:0.13199829,WP_013641468.1|Lactobacillus_acidophilus:0.18433174):0.6637783[1.00]):0.15411088[1.00],(WP_008469878.1|Lactobacillus_hominis:0.3410724,WP_013641481.1|Lactobacillus_acidophilus:0.6116567):0.25721663[1.00]):0.09408422[0.99]):0.08061461[0.94]):0.1057425[0.98],(WP_011254167.1|Lactobacillus_acidophilus:0.5286376,WP_056985318.1|Lactobacillus_amylovorus|LacAmy-E1:0.60585165):0.16076797[0.99]):0.3887732[1.00],(YP_006939186.1|Staphylococcus_epidermidis|pSAP110B:1.2614088,(WP_016356676.1|Carnobacterium_maltaromaticum:0.19607568,WP_016622553.1|Enterococcus_faecalis|EntFae-E1:0.17687474):0.7898191[1.00]):0.19601269[0.98]):0.11880106[0.92]):0.22954558[0.99]):0.28288904[1.00]):0.13208114[0.95]):0.13363788[0.78]):0.20227258[0.94]):0.47777396[0.99]):0.84306544[0.99],(KXT29014.1|Candidatus_Phytoplasma_oryzae:1.0809282,(KXT29032.1|Candidatus_Phytoplasma_oryzae:0.9870109,(((WP_017193171.1|Italian_clover_phyllody_phytoplasma:0.05466932,WP_017193695.1|Vaccinium_witches-broom_phytoplasma:0.07289085):0.32945332[1.00],(WP_015083745.1|Wheat_blue_dwarf_phytoplasma:0.0237979,(YP_007008175.1|Wheat_blue_dwarf_phytoplasma|pWBD1:0.03894575,YP_001708784.1|Paulownia_witches-broom_phytoplasma|pPaWBNy-1:0.1759096):0.02951508[0.99]):0.20428869[1.00],(YP_006961991.1|Periwinkle_little_leaf_phytoplasma|pPLLHn-1:0.12566353,(((WP_013747472.1|Chinaberry_witches-broom_phytoplasma:0.07343964,(YP_001708790.1|Paulownia_witches-broom_phytoplasma|pPaWBNy-2:0.11888808,YP_007008179.1|Wheat_blue_dwarf_phytoplasma|pWBD3:0.06971444):0.03930107[0.99]):0.02870485[0.99],(WP_011412958.1|Aster_yellows_witches-broom_phytoplasma:0.11066895,(WP_011412950.1|Aster_yellows_witches-broom_phytoplasma|pAYWB-I:0.05886067,CBX25033.1|New_Jersey_aster_yellows_phytoplasma:0.02429186):0.2587148[1.00]):0.04254161[0.99]):0.05865239[1.00],(YP_001965310.1|Candidatus_Phytoplasma_australiense|pPASb11:0.14671111,YP_001965305.1|Candidatus_Phytoplasma_australiense|pPAPh2:0.36999953):0.06618552[0.99]):0.03709533[0.93]):0.16596928[1.00]):0.44400048[0.99],(BAD36752.1|Onion_yellows_phytoplasma:0.00267713,(YP_006959585.1|Onion_yellows_phytoplasma|pEcOYNIM:1.4E-7,WP_011264167.1|Onion_yellows_phytoplasma:0.00859747):0.06754499):1.1650867[0.99]):0.62843686[1.00]):0.21879163[1.00]):1.2980072[0.79]):1.987953[1.00],(AAF36424.1|Pyropia_pulchra|plasmid:0.14581142,(AAF36423.1|Pyropia_pulchra|plasmid:0.08398085,AAF36422.1|Pyropia_pulchra|plasmid:0.06552102):0.17535713[0.98]):0.022850035[1.00])OROOT;
