# Supplementary material for: Multiple origins of prokaryotic and eukaryotic single-stranded DNA viruses from bacterial and archaeal plasmids
Source: Nat Commun. 2019 Jul 31;10:3425. doi: 10.1038/s41467-019-11433-0 (PMC6668415; doi:10.1038/s41467-019-11433-0)
Supplement: Supplementary file 6 — Dataset 5 [file 41467_2019_11433_MOESM6_ESM.docx]

**SUPPLEMENTARY DATA 5**

# PHYLOGENETIC TREE SHOWN IN FIGURE 5

(pCRESS4|WP_006681830.1:0.00000357,pCRESS4|WP_052038917.1:0.00436329,((pCRESS4|GAC78794.1:0.40930715,(pCRESS4|WP_043534193.1:0.09633329,(pCRESS4|WP_067940518.1:0.04043094,pCRESS4|WP_005464724.1:0.00488585)0.999035:0.02992006)1.000000:0.14010229)1.000000:0.41175432,(pCRESS4|WP_044572803.1:0.57321309,((pCRESS4|WP_017824301.1:0.21595505,pCRESS4|CEI31812.1:0.25505265)1.000000:0.34745153,(((pCRESS4|WP_000818357.1:0.41361573,pCRESS4|WP_000186194.1:0.46400266)1.000000:0.28860720,(pCRESS4|CRY97508.1:0.62691707,((pCRESS4|CBL40434.1:0.49605106,(pCRESS4|WP_021639163.1:0.44306187,pCRESS4|CRY93789.1:0.30819741)0.596101:0.07137100)0.971416:0.07346806,(pCRESS4|CDA18875.1:0.66937645,pCRESS4|WP_007889993.1:0.62312322)0.995030:0.12302200)0.994013:0.06420273)0.999987:0.12213461)0.500101:0.05286164,(((pCRESS7|CDE19587.1:0.86972471,(((pCRESS7|CCZ93342.1:0.30112363,pCRESS7|CCY61699.1:0.32450966)1.000000:0.17224494,(pCRESS7|WP_019282500.1:0.55737476,(pCRESS7|ODR34583.1:0.42745558,((pCRESS7|KXT29039.1:0.38070351,(pCRESS7|YP_001966814.1:0.24519868,(pCRESS7|WP_011161011.1:0.03989096,(pCRESS7|ABC65805.1:0.06847774,(pCRESS7|ABC65794.1:0.15673291,(pCRESS7|YP_006961027.1:0.03435984,pCRESS7|YP_003617079.1:0.04338550)1.000000:0.10498519)0.999999:0.05712612)0.999694:0.04380290)1.000000:0.09711349)0.998777:0.09626289)1.000000:0.22588440,(pCRESS7|CCZ68460.1:0.39257575,pCRESS7|SCG87263.1:0.52307378)1.000000:0.32292023)0.999657:0.12173060)0.999351:0.10980850)0.999996:0.12708207)0.752652:0.05499849,(pCRESS7|WP_028509833.1:0.83871971,(pCRESS7|WP_002578150.1:0.68314888,pCRESS7|CUN62864.1:0.56234730)0.999995:0.23536404)0.907884:0.09211752)0.879321:0.16199649)1.000000:0.40455959,((((pCRESS6|WP_014571792.1:0.83245391,(pCRESS6|AEU41945.1:0.67769617,(((pCRESS6|WP_056938517.1:0.40918873,(pCRESS6|ADX23728.1:0.42671997,((pCRESS6|WP_034704841.1:0.06396546,pCRESS6|WP_067483596.1:0.05278131)1.000000:0.17000606,(pCRESS6|WP_003024533.1:0.22929318,((pCRESS6|WP_017649267.1:0.07849148,pCRESS6|ABJ73998.1:0.13676402)1.000000:0.07562683,((pCRESS6|WP_049476139.1:0.06879608,pCRESS6|WP_003035134.1:0.04766192)1.000000:0.07243405,((pCRESS6|WP_053092713.1:0.15070278,pCRESS6|KEQ49321.1:0.10916374)1.000000:0.04772171,(((pCRESS6|WP_027972054.1:0.13235040,(pCRESS6|WP_044774450.1:0.04176269,pCRESS6|WP_020997784.1:0.07171116)1.000000:0.15657408)0.917716:0.02380842,(pCRESS6|WP_039677656.1:0.18696381,(pCRESS6|WP_049499636.1:0.08968961,pCRESS6|WP_045759092.1:0.06634075)1.000000:0.13210478)1.000000:0.04902669)0.866762:0.01633253,(pCRESS6|WP_014623544.1:0.10259948,((pCRESS6|WP_032497992.1:0.16840793,pCRESS6|KXT86702.1:0.05287272)0.993805:0.02744507,(pCRESS6|CGE81062.1:0.18842075,pCRESS6|WP_015647385.1:0.16344563)0.826457:0.02379279)0.997526:0.02094288)0.999980:0.02307642)1.000000:0.03416841)1.000000:0.04990958)1.000000:0.05502151)0.999865:0.04828044)0.999853:0.06617377)0.999999:0.11466114)0.776964:0.05514615)0.999988:0.11213532,((pCRESS6|WP_044762265.1:0.09279453,(pCRESS6|WP_018376545.1:0.08946249,pCRESS6|WP_020999261.1:0.11405632)0.991247:0.05184188)1.000000:0.40537010,((pCRESS6|WP_000044268.1:0.20241543,(pCRESS6|CMU27730.1:0.18000626,(pCRESS6|WP_000201649.1:0.05665287,pCRESS6|WP_047206721.1:0.03123516)1.000000:0.09640438)1.000000:0.09802798)1.000000:0.09152159,((pCRESS6|WP_024400359.1:0.10867300,(pCRESS6|WP_004183001.1:0.07169429,(pCRESS6|WP_001034312.1:0.04168396,pCRESS6|WP_024385235.1:0.06043594)0.999621:0.02014962)0.989153:0.02330279)1.000000:0.14868752,(pCRESS6|WP_000791389.1:0.17309617,pCRESS6|WP_003032217.1:0.38682927)0.937678:0.03866917)0.999054:0.05276690)1.000000:0.09799196)1.000000:0.13019460)1.000000:0.19760026,(pCRESS6|WP_052506726.1:0.67129941,(pCRESS6|WP_022765681.1:0.64850202,(pCRESS6|WP_051176704.1:0.54889442,(pCRESS6|WP_036321578.1:0.29721588,(pCRESS6|WP_054952722.1:0.01878308,pCRESS6|WP_041290927.1:0.02739337)1.000000:0.13755872)1.000000:0.26270950)1.000000:0.17111716)0.676391:0.09524935)0.988394:0.09675699)1.000000:0.18586730)0.719158:0.07036981)0.993997:0.10098393,(((pCRESS6|WP_032941943.1:0.02961340,pCRESS6|WP_058223604.1:0.02677016)1.000000:0.37014542,(pCRESS6|WP_019299400.1:0.11213859,pCRESS6|BAM66968.1:0.12045835)1.000000:0.29158531)0.999996:0.09293953,((pCRESS6|WP_039670385.1:0.18869041,(pCRESS6|WP_018030886.1:0.13473216,pCRESS6|WP_003104234.1:0.10615307)1.000000:0.10750154)1.000000:0.23514728,((pCRESS6|WP_025016923.1:0.06888021,pCRESS6|KST89836.1:0.06220570)1.000000:0.35985561,(((pCRESS6|WP_003048523.1:0.04420403,pCRESS6|WP_039694464.1:0.09004336)1.000000:0.04451035,(pCRESS6|WP_044671103.1:0.09547494,pCRESS6|WP_000746010.1:0.12613865)0.999244:0.02965836)1.000000:0.13807759,(pCRESS6|WP_018380019.1:0.12804725,pCRESS6|EOB33201.1:0.14767331)1.000000:0.07699843)1.000000:0.13335647)0.999968:0.07850093)0.999135:0.06146936)1.000000:0.29215454)0.774368:0.11218895,(pCRESS6|WP_061343647.1:0.40541189,pCRESS6|WP_017371219.1:0.38585154)1.000000:0.40500967)1.000000:0.54638589,((pCRESS8|WP_062359070.1:0.69677789,((((pCRESS8|EEJ43069.1:0.47202235,pCRESS8|WP_004900270.1:0.58800002)1.000000:0.26487796,(pCRESS8|ABP89830.1:0.90879028,((pCRESS8|WP_016226904.1:0.32149114,pCRESS8|SCH55298.1:0.52006621)1.000000:0.27010806,((pCRESS8|WP_000093566.1:0.00000025,(pCRESS8|KXA58447.1:0.00212759,(pCRESS8|WP_042900192.1:0.00241294,pCRESS8|WP_050492321.1:0.00005756)0.333437:0.00209730)0.835227:0.00426722)1.000000:0.65157866,(((pCRESS8|CYX46115.1:0.10615843,pCRESS8|CYW87437.1:0.04329185)0.999977:0.06865450,(pCRESS8|WP_024410839.1:0.05599983,(pCRESS8|WP_049523992.1:0.12226693,(pCRESS8|WP_033683822.1:0.01225598,pCRESS8|EFO53527.1:0.01775759)1.000000:0.07035624)1.000000:0.17470552)0.999699:0.10091767)1.000000:0.33107297,(pCRESS8|WP_051448806.1:0.16947349,(pCRESS8|WP_050444210.1:0.06639362,pCRESS8|WP_010817837.1:0.04305662)1.000000:0.18332076)1.000000:0.31399748)0.999993:0.13320385)0.602527:0.05548713)0.932244:0.05482182)0.901523:0.03608068)0.996706:0.08532610,((pCRESS8|AKG47101.1:0.85223802,((pCRESS8|WP_046025501.1:0.29320966,pCRESS8|WP_034540695.1:0.20779645)1.000000:0.59710523,((pCRESS8|CUR41281.1:0.69680954,(pCRESS8|WP_046923918.1:0.67563880,(pCRESS8|WP_003665528.1:0.55629083,pCRESS8|WP_006499656.1:0.61835765)0.790714:0.08821081)0.601688:0.06377832)0.700300:0.06227736,pCRESS8|KRN07545.1:0.59959058)0.995461:0.11045795)0.923036:0.06888021)0.997073:0.10067062,(pCRESS8|WP_002821392.1:0.83597134,(pCRESS8|WP_057827085.1:0.49567089,(pCRESS8|WP_057906729.1:0.03904214,pCRESS8|WP_057827851.1:0.14525038)1.000000:0.48597117)0.999579:0.15385968)0.999951:0.15779295)0.991033:0.07244141)0.737652:0.08055907,(((pCRESS8|WP_012845653.1:0.14337665,(pCRESS8|CDI42894.1:0.21127420,((pCRESS8|CDA26462.1:0.09423373,(pCRESS8|WP_003549058.1:0.04964980,pCRESS8|KRN00682.1:0.04200634)1.000000:0.09245199)0.999250:0.04932857,(pCRESS8|CDI43023.1:0.08635921,pCRESS8|KRK41125.1:0.17912738)0.950946:0.04212485)0.999996:0.07787835)0.989950:0.05999998)1.000000:0.32299710,((pCRESS8|WP_056985318.1:0.50242174,pCRESS8|WP_011254167.1:0.36458533)0.999999:0.16160396,((pCRESS8|WP_014567781.1:0.32343361,(pCRESS8|WP_007125042.1:0.12259460,pCRESS8|WP_060461663.1:0.14166170)0.999998:0.09851263)1.000000:0.14263191,((pCRESS8|WP_049150683.1:0.29267459,((pCRESS8|WP_008472153.1:0.09900982,pCRESS8|WP_013641468.1:0.12821337)1.000000:0.49730325,pCRESS8|WP_046324376.1:0.25958680)0.672603:0.04425768)1.000000:0.09811390,(pCRESS8|WP_008469878.1:0.25943653,pCRESS8|WP_013641481.1:0.44652671)1.000000:0.19917969)0.998958:0.07661409)0.967753:0.06106029)0.951017:0.07851609)1.000000:0.29147265,(pCRESS8|YP_006939186.1:0.99738409,(pCRESS8|WP_016356676.1:0.16464915,pCRESS8|WP_016622553.1:0.08926500)1.000000:0.57634981)0.928024:0.09705966)0.870629:0.06993154)0.999832:0.12851932)1.000000:0.20686069,((pCRESS5|UniRef50_W1I5Y6:0.62053523,pCRESS5|UniRef50_R5VXD3:1.07539401)0.999976:0.52345089,(((((pCRESS5|WP_014735272.1:0.00636045,(pCRESS5|WP_053863690.1:0.01458781,(pCRESS5|WP_018166163.1:0.02763230,(pCRESS5|WP_024399566.1:0.03473360,(pCRESS5|WP_024382134.1:0.00198266,pCRESS5|WP_024389873.1:0.01038272)0.999956:0.00703740)0.723496:0.00655334)1.000000:0.02332631)0.397302:0.00208697)1.000000:0.32657447,((pCRESS5|WP_044771983.1:0.08942304,((pCRESS5|WP_024408358.1:0.04603082,(pCRESS5|WP_033583888.1:0.00389049,pCRESS5|WP_049481849.1:0.00398593)1.000000:0.10631164)1.000000:0.03119123,(pCRESS5|WP_039694423.1:0.04734970,pCRESS5|WP_029171254.1:0.08666949)0.588201:0.00886661)0.999976:0.03046421)1.000000:0.09347947,(pCRESS5|WP_067193806.1:0.10374988,(pCRESS5|WP_049535277.1:0.01576982,(pCRESS5|WP_049478725.1:0.04577627,(pCRESS5|WP_061417941.1:0.00963719,pCRESS5|WP_061863770.1:0.02455942)0.931700:0.01356722)0.701952:0.01737274)1.000000:0.12613950)0.726619:0.02650263)0.999999:0.17610617)1.000000:0.55036172,(pCRESS5|WP_061866456.1:0.62061140,((pCRESS5|WP_024393234.1:0.13644766,pCRESS5|WP_050238550.1:0.13567303)1.000000:0.45128693,(pCRESS5|WP_024390948.1:0.18254898,pCRESS5|WP_029176301.1:0.20182906)1.000000:0.50812064)0.515648:0.13858517)1.000000:0.38316078)0.959060:0.12071715,(pCRESS5|WP_038978316.1:0.46611283,pCRESS5|WP_046467524.1:0.43250779)1.000000:0.46464311)0.613555:0.03307401,(pCRESS5|WP_058211405.1:0.34896589,pCRESS5|WP_017368666.1:0.19879616)1.000000:0.56110524)0.825466:0.18251931)1.000000:0.50330660)0.433620:0.03533226)0.814462:0.05232955)0.982101:0.10972902,((pE194_pMV158-like|UniRef50_F0HMF3:0.89428419,((((pE194_pMV158-like|UniRef50_A0A0H5PZW4:0.58545648,pE194_pMV158-like|UniRef50_A0A0H5PV05:0.61970344)0.999872:0.18230296,(pE194_pMV158-like|UniRef50_A0A1C6AUS2:0.38967784,pE194_pMV158-like|UniRef50_A0A0E9DRD0:0.34657846)0.999767:0.16483708)0.362017:0.02457059,((pE194_pMV158-like|UniRef50_G8CNT2:0.29396729,pE194_pMV158-like|UniRef50_A0A0E9F4G4:0.57693290)1.000000:0.36059006,(pE194_pMV158-like|UniRef50_A0A0H5PZG0:0.53355663,pE194_pMV158-like|UniRef50_A0A0H5QIL6:0.56875560)1.000000:0.37224944)0.719359:0.10305136)0.971472:0.08936341,(pE194_pMV158-like|UniRef50_A0A1Y4G0S9:0.65889573,(pE194_pMV158-like|UniRef50_A0A158LH93:0.57852978,((pE194_pMV158-like|UniRef50_A0A0H5PZI7:0.61212744,pE194_pMV158-like|UniRef50_G8CNR9:0.52489151)0.809651:0.10088146,((pE194_pMV158-like|UniRef50_A0A0R3QHC2:0.53970001,((((pE194_pMV158-like|UniRef50_K7YFJ8:0.64359459,pE194_pMV158-like|UniRef50_O31070:0.37568966)1.000000:0.42813769,(((pE194_pMV158-like|UniRef50_U2EU97:0.27678410,((pE194_pMV158-like|UniRef50_A0A0H5Q0X0:0.47736680,pE194_pMV158-like|UniRef50_A0A0E9EV38:0.35750953)0.998961:0.14601045,(pE194_pMV158-like|UniRef50_K9RZV9:0.38059938,pE194_pMV158-like|UniRef50_K9RYD5:0.92940749)0.999602:0.17854891)0.735823:0.09453600)0.998500:0.16153831,((pE194_pMV158-like|UniRef50_S6CES9:1.51498392,((pE194_pMV158-like|UniRef50_U2TJ01:0.69380206,pE194_pMV158-like|UniRef50_A0A1Y3UDM3:0.66410649)0.999441:0.23575478,((pE194_pMV158-like|UniRef50_A0A087EKU7:0.11515780,(pE194_pMV158-like|UniRef50_D3R6U9:0.21833152,pE194_pMV158-like|UniRef50_A0A0S2MGE2:0.24965508)0.531106:0.05919316)1.000000:0.84042675,pE194_pMV158-like|UniRef50_A0A0H5Q8X5:0.89336892)0.632529:0.04058134)0.449782:0.06647606)0.550919:0.10746064,((pE194_pMV158-like|UniRef50_A0A1Y4QQC0:0.32058229,(pE194_pMV158-like|3DKX_A:0.12656150,pE194_pMV158-like|UniRef50_A8W662:0.35147937)0.997400:0.21236660)1.000000:0.54606360,(pE194_pMV158-like|UniRef50_W1I697:0.67124315,(pE194_pMV158-like|UniRef50_A0A0E9F9L7:0.42088670,pE194_pMV158-like|UniRef50_A0A174GG61:0.77345835)0.987823:0.21579225)1.000000:0.52977868)0.999957:0.37007677)0.985525:0.14791811)0.931806:0.10301080,(pE194_pMV158-like|UniRef50_U2QZX4:0.82902063,pE194_pMV158-like|UniRef50_UPI000481AAFB:1.00056522)0.620824:0.10025508)0.998065:0.13268502)0.975938:0.08328673,(pE194_pMV158-like|UniRef50_W7D2V3:0.41937906,pE194_pMV158-like|UniRef50_S6F6F1:0.92737258)0.996380:0.20448190)0.988942:0.07240054,(pE194_pMV158-like|UniRef50_A0A1B1IHL4:1.01429408,(pE194_pMV158-like|UniRef50_A0A0R1P770:0.90738993,pE194_pMV158-like|UniRef50_Q48831:1.00734307)0.551102:0.09648702)0.817083:0.11288021)0.761843:0.05198860)0.998412:0.11934522,(((pE194_pMV158-like|UniRef50_UPI000300949E:0.63695534,pE194_pMV158-like|UniRef50_A0A1W6BZG0:0.56792387)0.472598:0.07912917,(pE194_pMV158-like|UniRef50_W1I557:0.37091244,pE194_pMV158-like|UniRef50_A0A0Z8IYX5:0.87042451)0.997568:0.19627137)0.756573:0.09811166,pE194_pMV158-like|UniRef50_A0A0H5PZA0:0.81312569)0.788009:0.04447936)0.998241:0.09069633)1.000000:0.19161002)0.759735:0.08826488)0.762528:0.05751800)0.412740:0.06367205)1.000000:0.87851466,(((((CRESSV6|KP153501:0.58048381,(CRESSV6|KT149395:0.57891513,CRESSV6|KM598390:0.58632395)0.992351:0.16495940)0.855742:0.13132202,((CRESSV6|KM874358:0.51055276,CRESSV6|AJD07486.1:0.56609796)1.000000:0.31065904,(CRESSV6|KT732829:0.63597249,(CRESSV6|KM510189:0.44762176,CRESSV6|KP005454:0.39198477)1.000000:0.64466103)1.000000:0.26970555)0.471390:0.06270018)1.000000:0.45104156,(((CRESSV6-Wastewater|AUM61713.1:0.22657510,(CRESSV6-Wastewater|AUM61624.1:0.01213295,CRESSV6-Wastewater|AUM62043.1:0.00315232)1.000000:0.37041822)0.808041:0.05472112,(CRESSV6-Wastewater|AUM61719.1:0.25849033,CRESSV6-Wastewater|AUM61738.1:0.63747111)0.830344:0.10488755)1.000000:0.42611667,(PpulchraPlasmids|ETO15557.1:0.60953176,((PpulchraPlasmids|OLY79419.1:0.23618062,PpulchraPlasmids|OLY79389.1:0.23937670)1.000000:0.32499940,((PpulchraPlasmids|OMJ09562.1:0.59004184,(PpulchraPlasmids|OMJ11569.1:0.30074793,(PpulchraPlasmids|OMJ21113.1:0.05174562,(PpulchraPlasmids|OMJ28371.1:0.09840367,PpulchraPlasmids|OMJ13215.1:0.01863826)0.777962:0.07279580)1.000000:0.26210940)0.979673:0.10421178)0.999050:0.10441845,(PpulchraPlasmids|OLY79699.1:0.92267448,(PpulchraPlasmids|AAF36424.1:0.13636787,(PpulchraPlasmids|AAF36423.1:0.06349505,PpulchraPlasmids|AAF36422.1:0.04479978)0.918273:0.08967501)1.000000:1.06951935)0.731862:0.10847309)0.946041:0.06476396)0.995845:0.12884804)0.999990:0.19376923)0.998929:0.20489076)1.000000:0.45756541,(((Genomo|AUM61807.1:0.78377785,(Genomo|KP153522:0.90035360,(((Genomo|KM598389:0.52708675,Genomo|KJ547627:0.50096931)1.000000:0.34991944,(Genomo|KJ547626:0.68815267,(Genomo|KJ547634:0.78729544,(Genomo|YP_009109725.1:0.42660030,(Genomo|AMH87708.1:0.23366210,((Genomo|AJD07464.1:0.24166136,Genomo|AMH87693.1:0.23777247)1.000000:0.38402719,(Genomo|YP_009164036.1:0.48640691,(Genomo|AMH87702.1:0.46109867,(Genomo|AMH87733.1:0.36169638,((Genomo|YP_009109727.1:0.32939536,Genomo|YP_009252362.1:0.28717640)1.000000:0.19167780,((((Genomo|YP_009115514.1:0.20683326,Genomo|YP_003104796.1:0.19222587)1.000000:0.13472119,((Genomo|AMH87666.1:0.31022480,Genomo|YP_009252353.1:0.15348615)0.999277:0.06188699,((Genomo|YP_009115515.1:0.11110327,(Genomo|AIF34843.1:0.13911730,(Genomo|YP_009115519.1:0.07427185,Genomo|KJ547638:0.06285822)0.826766:0.03876614)0.999596:0.04179498)1.000000:0.12518861,(Genomo|YP_009252356.1:0.14471322,(Genomo|YP_009252368.1:0.09570485,Genomo|YP_009021043.1:0.04338318)1.000000:0.23528538)0.947990:0.03694865)1.000000:0.07025906)0.999909:0.04393813)0.523876:0.02138921,Genomo|AGS12486.1:0.33933198)1.000000:0.10798764,(((Genomo|YP_009109733.1:0.08117027,Genomo|KT862241:0.05158395)1.000000:0.22522809,(Genomo|AMH87678.1:0.20749325,(Genomo|KT253577:0.16341481,(Genomo|YP_009252365.1:0.18572975,Genomo|YP_009252359.1:0.14144417)1.000000:0.10615300)0.999466:0.05528492)1.000000:0.12437349)1.000000:0.11192234,(Genomo|YP_009109729.1:0.54321777,(Genomo|YP_009181999.1:0.00891909,Genomo|KT598248:0.00000023)1.000000:0.36082516)0.999997:0.14275189)1.000000:0.13370188)0.897603:0.05576231)0.653127:0.04420433)0.999980:0.10047822)0.915333:0.05374320)0.825026:0.05578565)0.941563:0.05883826)0.999789:0.16818774)1.000000:0.45683942)0.994803:0.17735700)0.760856:0.09829334)0.998137:0.14460878,((Genomo|KJ938716:0.28185304,Genomo|KM821748:0.19505989)1.000000:0.81069282,Genomo|YP_009351871.1:0.96992532)0.707930:0.10280004)0.949355:0.10284448)0.675159:0.06519102)0.999997:0.17295834,(((Gemini|ACO88014.1:0.01695021,Gemini|FJ665634:0.00399945)1.000000:0.62666895,((((Gemini|AAL96826.1:0.14949466,(Gemini|CRI68211.1:0.19076930,Gemini|YP_003828907.1:0.12059205)0.988933:0.02608313)0.889379:0.02646465,(Gemini|AFM38721.1:0.20034843,(Gemini|NP_066185.1:0.12280810,Gemini|YP_003966137.1:0.10460646)1.000000:0.06700913)0.999916:0.04334908)0.638989:0.01420736,Gemini|AGV02076.1:0.11007556)0.999990:0.11260642,(Gemini|YP_007250561.1:0.14759701,((Gemini|YP_619883.1:0.14587696,(Gemini|NP_040557.1:0.14210991,(Gemini|ALF37659.1:0.16395214,(Gemini|ALR86823.1:0.20983506,(Gemini|YP_009226627.1:0.07683934,(Gemini|YP_003778178.1:0.00973296,Gemini|KC108902:0.04213370)1.000000:0.16530862)0.994999:0.03988778)1.000000:0.12616385)0.999998:0.07486774)1.000000:0.11249571)0.429348:0.00679316,((((Gemini|ACV83312.1:0.09819987,Gemini|AER09339.1:0.10151821)1.000000:0.06128906,(Gemini|YP_002941855.1:0.11573277,Gemini|YP_006590064.1:0.09954425)0.485233:0.01756478)0.999977:0.02630303,((Gemini|AGH29892.1:0.06386051,((Gemini|ALV85583.1:0.13055992,(Gemini|CBA18089.1:0.09389846,Gemini|FJ665283:0.06890417)0.927514:0.01872398)1.000000:0.03749516,(Gemini|AMW86999.1:0.15726486,Gemini|CBH28932.1:0.14243342)0.446801:0.00725960)0.958980:0.00970089)1.000000:0.05244312,(Gemini|AHX57826.1:0.16446428,(Gemini|NP_671468.1:0.13172363,Gemini|AFD54490.1:0.14044428)0.886482:0.02347336)0.997248:0.02163616)0.980971:0.01878513)0.888089:0.01255939,((Gemini|ADN84041.1:0.10363134,Gemini|AAN76737.1:0.10982862)1.000000:0.08135174,((Gemini|YP_001285764.1:0.20370610,Gemini|AMP46444.1:0.15257909)1.000000:0.08509370,((Gemini|YP_764516.1:0.12294209,((((Gemini|CDW92215.1:0.12437844,(Gemini|YP_006905839.1:0.13486128,(Gemini|AEY63664.1:0.17160718,(Gemini|AAX39336.1:0.02060070,Gemini|FM877473:0.02067034)1.000000:0.06118729)1.000000:0.04727720)0.999990:0.03620028)0.997134:0.02443642,(Gemini|NP_620741.1:0.10565478,Gemini|YP_008411025.1:0.23033260)0.974577:0.02751919)0.999879:0.01786893,((Gemini|AHL29198.1:0.07530684,(Gemini|AFF58888.1:0.14061904,(Gemini|AGF41094.1:0.06328398,Gemini|CAJ85998.1:0.07183047)1.000000:0.03498741)0.979313:0.01437849)0.999947:0.02003506,((Gemini|BAF02752.1:0.17084837,Gemini|AGG08895.1:0.08602749)0.991383:0.01792973,((Gemini|AFA26437.2:0.05889176,((Gemini|AFB81519.1:0.10777772,Gemini|ACI06063.1:0.11141972)0.999999:0.03254147,(Gemini|AFB83419.1:0.12816867,Gemini|AFH68197.1:0.10752262)0.000000:0.00000026)1.000000:0.05548552)0.997048:0.01926975,((Gemini|AGV02071.1:0.13616618,(Gemini|ACV60535.1:0.09419226,(Gemini|AAF75542.1:0.11490124,Gemini|NP_050017.1:0.10276487)0.999689:0.03272691)0.999999:0.02958049)0.974912:0.01442851,(Gemini|AHA82274.1:0.15026124,(Gemini|AGJ03640.1:0.15904102,Gemini|ACB44970.1:0.05821011)1.000000:0.09429871)0.908096:0.03076546)0.999908:0.02395093)1.000000:0.03637607)0.999158:0.01113547)0.999999:0.01713688)0.999949:0.01913979,((((Gemini|YP_003622552.1:0.17112630,Gemini|ADW24243.1:0.13930469)0.975169:0.04562343,(Gemini|ABD67440.1:0.27391113,Gemini|AKS48121.1:0.11075945)0.999996:0.06568714)1.000000:0.06133126,((Gemini|CAM91896.1:0.10031566,(Gemini|YP_009129272.1:0.18311919,(Gemini|AJM13604.1:0.10905638,Gemini|YP_004958233.1:0.14918775)1.000000:0.07268240)0.966122:0.02703077)1.000000:0.08005289,(Gemini|AIY31184.1:0.11746865,Gemini|AEE99005.1:0.21119757)1.000000:0.06582238)0.709057:0.01300316)0.920769:0.01014362,(((Gemini|AMK07575.1:0.16335994,(Gemini|YP_002224032.1:0.19470284,Gemini|CBJ17676.1:0.05301048)0.999938:0.03641462)0.999994:0.03370260,(Gemini|YP_001040016.1:0.07795269,(Gemini|AAP73446.1:0.24562166,(Gemini|ABG90906.1:0.02805342,Gemini|ACY79450.1:0.18780063)1.000000:0.14598675)0.989700:0.04800261)0.966040:0.02486723)1.000000:0.09070815,(Gemini|ABD35287.1:0.15477331,(Gemini|YP_001333687.1:0.09762836,Gemini|YP_115511.1:0.09758844)1.000000:0.21368528)0.976983:0.04121964)0.999721:0.02430410)0.999999:0.01930419)0.928503:0.01405899)0.999994:0.03496508,(Gemini|APP87725.1:0.30512687,(Gemini|AAB87607.1:0.07606531,Gemini|AGK24653.1:0.11183204)1.000000:0.10557605)0.990310:0.04831026)0.850318:0.01255392)0.999999:0.04396505)1.000000:0.13957011)0.999992:0.02499627)0.771826:0.01787167)1.000000:0.11237306)1.000000:0.19787889)1.000000:0.22979688,((Gemini|KT214373:0.12444249,Gemini|JX094280:0.16331750)1.000000:0.42490555,(Gemini|DQ458791:0.37922870,(Gemini|YP_009021763.1:0.40579167,(((Gemini|AHM88370.1:0.37339063,((Gemini|AAK73446.1:0.00384707,Gemini|AF003952:0.01582718)1.000000:0.19589400,((Gemini|AHM88378.1:0.15428355,Gemini|Q80GM6.2:0.16181128)1.000000:0.09791468,(Gemini|YP_003288768.1:0.15365215,(Gemini|AHM88382.1:0.11045085,Gemini|P0C647.1:0.26939959)0.884867:0.03901010)0.995320:0.04224963)0.998302:0.06622206)1.000000:0.14231473)0.933899:0.05399623,(Gemini|YP_006273070.1:0.31076137,Gemini|YP_009026388.1:0.43648169)0.961186:0.07369017)0.999999:0.11052230,(((Gemini|YP_006666535.1:0.11424924,(Gemini|AFN80601.1:0.14109882,(Gemini|YP_004089627.1:0.11008882,Gemini|YP_006666523.1:0.15606018)0.999999:0.04594543)0.999090:0.06820049)1.000000:0.26386323,(Gemini|YP_003915159.1:0.18771812,Gemini|AFN80669.1:0.27068341)1.000000:0.15851978)1.000000:0.27179443,((Gemini|YP_006666531.1:0.22807155,Gemini|YP_006666527.1:0.42064105)0.999901:0.08375791,(Gemini|AFV91331.1:0.24698991,Gemini|AIT39773.1:0.42065416)0.935167:0.05984593)0.991525:0.07767977)0.910072:0.07605578)0.996327:0.08710255)0.999989:0.12534742)1.000000:0.25143016)0.999997:0.20296785)1.000000:0.19646071)0.999749:0.24509132,(pCRESS9|KXT29014.1:0.99022167,(pCRESS9|KXT29032.1:1.08667863,((pCRESS9|WP_012662291.1:0.02623037,((pCRESS9|BAD36752.1:0.00445308,pCRESS9|WP_042068233.1:0.00000013)1.000000:0.02937382,(pCRESS9|YP_006959585.1:0.00000013,(pCRESS9|WP_011264167.1:0.00673849,pCRESS9|WP_015060110.1:0.00222503)0.000000:0.00000013)0.999990:0.01468722)0.439452:0.01590460)1.000000:0.82528770,((pCRESS9|WP_017193171.1:0.02016406,pCRESS9|WP_017193695.1:0.05574589)1.000000:0.16198961,((pCRESS9|ATL14544.1:0.01590296,(pCRESS9|YP_001708784.1:0.08949547,(pCRESS9|YP_007008175.1:0.03124836,pCRESS9|WP_015083745.1:0.02938818)0.999877:0.03600714)0.995647:0.02836128)1.000000:0.10871184,(pCRESS9|YP_006961991.1:0.06490463,(((pCRESS9|WP_013747472.1:0.05514941,(pCRESS9|YP_001708790.1:0.07255814,pCRESS9|YP_007008179.1:0.04515419)0.999979:0.02702452)1.000000:0.02685073,((pCRESS9|WP_011412950.1:0.03352721,pCRESS9|CBX25033.1:0.02252298)1.000000:0.18077105,(pCRESS9|ABC65385.1:0.21376908,(pCRESS9|WP_011412958.1:0.00000038,pCRESS9|ABC65268.1:0.05894721)1.000000:0.05973894)0.948501:0.01679182)0.995977:0.02089414)0.999982:0.03341661,(pCRESS9|YP_001965305.1:0.23801454,pCRESS9|YP_001965310.1:0.11068160)0.990051:0.03816634)0.997499:0.03309337)1.000000:0.05907564)0.999780:0.09857125)0.999998:0.24761149)0.990463:0.18168491)0.999911:0.28085961)0.982900:0.27390586)0.999246:0.20667831)1.000000:0.57590700,(((((Circo|KT732825:0.88960000,((Circo|YP_009000900.1:0.49713395,((Circo|YP_009091696.1:0.39332250,(Circo|YP_004376332.1:0.45525508,Circo|ADD62475.1:0.39703618)0.999996:0.11378081)0.989618:0.06287465,((Circo|YP_009170674.1:0.49120308,((Circo|ABU48445.1:0.11797215,Circo|AHK80894.1:0.13482038)1.000000:0.39971020,((Circo|AEL28794.1:0.31568681,Circo|YP_803546.1:0.28234200)0.748858:0.04418869,(Circo|AFL02442.1:0.35530929,(Circo|ADU77009.1:0.13579934,((Circo|YP_764455.1:0.10924893,Circo|NP_573442.1:0.17326734)0.999310:0.04003980,(Circo|KU230452:0.07860297,Circo|YP_009134739.1:0.15660366)0.828073:0.01646198)0.999999:0.07323702)1.000000:0.11446077)0.933851:0.07046566)1.000000:0.18097471)0.999907:0.12066677)0.997990:0.10507456,((Circo|AIF76261.1:0.27514058,(Circo|AIF76248.1:0.13330664,(Circo|AIF76265.1:0.13626466,Circo|AIF76253.1:0.16299808)0.986692:0.06348559)0.999980:0.12139659)1.000000:0.31404267,((Circo|AGL09969.1:0.12668423,Circo|YP_009021891.1:0.11626882)1.000000:0.28110291,(Circo|AKO84203.1:0.39455875,((Circo|AIF76280.1:0.01222400,Circo|KJ641742:0.00621826)1.000000:0.23344217,(Circo|YP_007974237.1:0.18001692,Circo|AAZ78351.1:0.21970720)0.999532:0.09053416)1.000000:0.34238003)0.989034:0.11308523)0.999335:0.11371966)0.544154:0.03700219)0.999987:0.09931961)0.994745:0.08051180)0.999824:0.10010981,(Circo|AIF76251.1:0.71860146,((Circo|YP_009237526.1:0.53126906,Circo|YP_009116910.1:0.57008573)0.995699:0.11476157,((Circo|AMH87650.1:0.06397133,Circo|AMH87652.1:0.20163808)1.000000:0.68347993,((Circo|ADU76993.1:0.16874354,(Circo|ADD62477.1:0.10597244,(Circo|YP_009047065.1:0.15860810,Circo|YP_008130363.1:0.16116397)0.961466:0.06437376)0.974620:0.06404675)1.000000:0.31948320,((Circo|YP_009021870.1:0.26507420,Circo|AFS65290.1:0.23571611)0.999966:0.09034531,(Circo|AIF76254.1:0.32565849,((((Circo|AKE49355.1:0.21544776,((Circo|ADD62451.1:0.13085398,Circo|ADD62455.1:0.16592182)0.999096:0.05399409,(Circo|ADD62461.1:0.22851257,(Circo|ADU77011.1:0.15377243,Circo|AGJ74758.1:0.20963812)0.984757:0.04945666)0.469901:0.03620452)0.999603:0.04556587)0.999995:0.05444618,((Circo|YP_009110680.1:0.14379034,Circo|AGJ74756.1:0.26205499)0.999140:0.04726007,(Circo|ADD62457.1:0.21408356,(Circo|AEL87792.1:0.20811163,(Circo|AIF76266.1:0.20745255,Circo|ADD62473.1:0.09445990)1.000000:0.08981422)1.000000:0.09046050)0.945057:0.03420061)0.996647:0.04666504)0.978454:0.03336246,(Circo|AGJ74760.1:0.19909007,Circo|ADY17982.1:0.27864566)0.998518:0.04973742)0.998176:0.04248627,(((Circo|YP_004152331.1:0.20657408,(Circo|ADD62471.1:0.15319060,Circo|AIF76252.1:0.10314671)1.000000:0.07232659)0.999948:0.02790335,(Circo|YP_009021843.1:0.30010327,(Circo|AEL87790.1:0.16286493,(Circo|AEL87786.1:0.20029358,Circo|ADI48251.1:0.18856005)0.999998:0.06820703)0.999556:0.05714102)0.724526:0.02115188)0.992799:0.03734477,(Circo|AIF76249.1:0.26034337,Circo|ADD62453.1:0.22433844)1.000000:0.08470716)0.995021:0.03273321)0.999971:0.06568517)0.996119:0.02674419)1.000000:0.15784298)0.999971:0.13499009)0.471587:0.04865400)0.944727:0.05641228)0.999556:0.12015019)0.988125:0.10490586)1.000000:0.23151466,((CRESSV1|KP153497:1.06232840,((CRESSV1|KM874309:0.62887418,CRESSV1|KF133822:0.59438067)0.999996:0.23551785,(CRESSV1|FJ959078:0.72462728,(CRESSV1|KT149404:0.83272590,CRESSV1|KM874347:0.66671209)0.572958:0.09770606)0.612764:0.10253292)0.809889:0.11717189)0.996127:0.10230946,((CRESSV1|KX388513.1:0.07057229,CRESSV1|KX388515.1:0.00000060)1.000000:1.29603098,(CRESSV1|KU043424:0.55837046,((CRESSV1|KJ206566:0.40264133,CRESSV1|KU043411:0.26586470)1.000000:0.28230467,(CRESSV1|KM573766:0.64861721,(CRESSV1|KT862256:0.25847924,CRESSV1|KF246569:0.20722277)1.000000:0.61235426)0.999145:0.12305174)0.750322:0.08781522)1.000000:0.54806926)0.567827:0.14214471)0.999999:0.16062986)1.000000:0.11216182,((CRESSV3|KM598406:0.50843839,(CRESSV3|KT149409:0.61595671,(CRESSV3|KT149403:0.76954116,(((CRESSV3|KP153422:0.45100244,CRESSV3|KP153408:0.31443640)0.945913:0.06017795,(CRESSV3|KM874317:0.44030540,(CRESSV3|KM874304:0.36957907,CRESSV3|JX904581:0.47343466)0.513148:0.05425439)0.950526:0.05160752)0.976860:0.08829172,(CRESSV3|KM874300:0.71565893,(CRESSV3|JX904407:0.79777618,(CRESSV3|JX904139:0.25982223,(CRESSV3|JX904075:0.32291727,CRESSV3|JX904076:0.25798990)0.821249:0.05923128)0.998195:0.14631361)0.994821:0.10840813)0.909538:0.05351191)0.999019:0.09742246)0.515693:0.02251690)0.559400:0.08171708)0.960759:0.07241280,((CRESSV3|JX185418:0.49374441,CRESSV3|KM598404:0.68730583)0.999381:0.18104251,(CRESSV3|KJ641729:0.74818906,((CRESSV3|KM972726:0.53820260,(CRESSV3|KJ641722:0.15270610,CRESSV3|HM228875:0.11576108)1.000000:0.45941761)0.514266:0.10285120,(CRESSV3|KF738883:0.69942081,(CRESSV3|JN857329:0.38307929,CRESSV3|KJ641718:0.36688194)0.999351:0.14203860)0.409991:0.04708605)0.997036:0.09057688)0.398429:0.03197260)0.999971:0.11245933)1.000000:0.15630725)0.946157:0.06601596,((((CRESSV2|KM573776:0.24483117,CRESSV2|KM573767:0.31397780)0.999996:0.15579649,(CRESSV2|KU043397:0.44894536,CRESSV2|KU043406:0.42586896)0.999296:0.14080208)1.000000:0.33463161,(CRESSV2|FJ959082:0.61328604,(((CRESSV2|KM821755:0.60762619,((CRESSV2|JX904344:0.38141492,(CRESSV2|KT732816:0.27674646,CRESSV2|JF755415:0.19550153)1.000000:0.30722533)0.974558:0.05767095,(CRESSV2|JX904420:0.52267754,(CRESSV2|KP153364:0.43533219,CRESSV2|JX904185:0.30658040)1.000000:0.18632997)0.999764:0.10017995)0.986590:0.03915584)0.999810:0.06574748,((CRESSV2|JX904107:0.46150329,CRESSV2|JX904562:0.31508018)1.000000:0.31983932,(CRESSV2|KF738877:0.79184411,CRESSV2|KP153377:0.55822054)0.999776:0.18319093)0.999122:0.12039950)0.999981:0.08131292,((CRESSV2|KT149394:0.38607706,CRESSV2|KP153360:0.30485562)1.000000:0.48298770,((CRESSV2|KP153369:0.81401850,(CRESSV2|KT732819:0.64851868,(CRESSV2|KP153447:0.48232391,((CRESSV2|KP153485:0.24697086,(CRESSV2|KJ547648:0.15711632,CRESSV2|KT149412:0.28910745)0.828050:0.07585214)1.000000:0.45029378,(CRESSV2|KT149398:0.63901907,(CRESSV2|KP153404:0.51243339,CRESSV2|KC248416:0.29994940)0.999362:0.11079578)0.897295:0.06865324)0.999993:0.15247559)0.723590:0.05339085)0.819911:0.06176786)0.971703:0.07549766,((CRESSV2|KM821764:0.64419647,(CRESSV2|KP153483:0.73333595,CRESSV2|KP153468:0.66885766)0.572133:0.12292161)0.997486:0.11714856,(CRESSV2|KT732823:0.87988653,(CRESSV2|KM598396:0.48104537,CRESSV2|JX185415:0.47417942)0.991783:0.22607602)1.000000:0.30020183)0.987777:0.13584847)0.801178:0.03294655)0.920429:0.04433017)0.603070:0.03812749)0.870632:0.06194553)1.000000:0.38026233,(((CRESSV4|KX388505.1:0.72791735,(CRESSV4|YP_009163936.1:0.47845762,(CRESSV4|AHH31482.1:0.35445862,(CRESSV4|YP_009237559.1:0.65477867,CRESSV4|YP_009021888.1:0.52883399)1.000000:0.26482015)0.871056:0.10635439)1.000000:0.22722135)0.999421:0.16694846,(NanoAlpha|AIF34798.1:0.57097658,(((NanoAlpha|AKO71308.1:0.00000033,NanoAlpha|JF957636:0.01473029)1.000000:0.38727331,(NanoAlpha|YP_003104737.1:0.02620789,NanoAlpha|HE654123:0.05844422)1.000000:0.29412892)1.000000:0.42175546,((NanoAlpha|NP_619759.1:0.36086419,(NanoAlpha|KF471057:0.45756115,NanoAlpha|JX458742:0.34712364)1.000000:0.29918395)0.975672:0.10623424,((NanoAlpha|U16735:0.16880918,NanoAlpha|KC979052:0.16290030)1.000000:0.25857559,((NanoAlpha|AAA51426.1:0.20987807,(NanoAlpha|AAA51422.1:0.04294396,NanoAlpha|ACB86656.1:0.27295574)1.000000:0.25363227)1.000000:0.23173519,((NanoAlpha|HM163578:0.03785104,(NanoAlpha|YP_008169853.1:0.08028191,(NanoAlpha|YP_009246456.1:0.12197064,NanoAlpha|ALK03646.1:0.14808788)1.000000:0.08140670)0.752165:0.03289028)1.000000:0.67978661,(NanoAlpha|NP_619760.1:0.14440856,(NanoAlpha|YP_009058890.1:0.05773363,NanoAlpha|KC978991:0.04231512)1.000000:0.26581187)1.000000:0.21576314)0.841777:0.05850707)0.999940:0.14132713)0.989413:0.08171687)1.000000:0.31366335)1.000000:0.28270630)1.000000:0.32030725)0.982070:0.11121919,((((CRESSV5|KJ641738:0.56302052,(CRESSV5|KR528547:0.60343026,CRESSV5|KR528561:0.45389417)0.903186:0.06149813)0.833040:0.06602143,(((CRESSV5|KR528554:0.31757170,CRESSV5|KR528556:0.28611731)0.999728:0.12958905,(CRESSV5|KR528551:0.43904384,CRESSV5|KR528562:0.43867960)1.000000:0.18974857)0.998818:0.11401022,(CRESSV5|KM874354:0.49482560,(CRESSV5|JX904231:0.50221502,(CRESSV5|KT945163:0.62667829,(CRESSV5|KR528545:0.41796339,CRESSV5|KR528553:0.59558141)0.956390:0.07084068)0.955493:0.09536326)0.535763:0.11215760)0.840791:0.08894146)0.809765:0.06322072)1.000000:0.18823197,(CRESSV5|KJ547646:0.66846787,(CRESSV5|KP153451:0.58004852,CRESSV5|KJ547650:0.53837662)0.990825:0.15856003)0.999998:0.17879307)0.741002:0.09363921,(((Smaco|AIY31256.1:0.30215672,(Smaco|AIY31243.1:0.14832173,Smaco|KT862224:0.33791091)0.970855:0.13696801)1.000000:0.61545310,(Smaco|AJD07511.1:0.69759467,(Smaco|YP_009252316.1:0.61047022,(((Smaco|AJE25851.1:0.14468187,Smaco|AJE25845.1:0.06639708)1.000000:0.11942791,(Smaco|KY086301:0.08873184,(Smaco|AJF23062.1:0.06187648,(Smaco|AJF23060.1:0.00880386,(Smaco|AJE25847.1:0.00922027,Smaco|KP233175:0.00000031)1.000000:0.17291044)1.000000:0.07820491)1.000000:0.13895768)0.884848:0.08446270)1.000000:0.47177018,(Smaco|YP_009252326.1:0.45562256,(Smaco|KU203352:0.18509898,Smaco|KJ547633:0.15814915)1.000000:1.11788719)0.702980:0.13653221)0.486229:0.04390505)0.990219:0.20623512)0.999930:0.29226515)0.999969:0.42141099,((Smaco|KM598409:0.53981304,(Smaco|YP_009252314.1:0.33602118,Smaco|AEW47007.1:0.24413538)0.999603:0.25781345)1.000000:0.84877616,(Smaco|YP_009252310.1:0.59426676,(((Smaco|KM573775:0.20010282,Smaco|KM573771:0.24444176)1.000000:0.21934975,(Smaco|KT862221:0.14030374,(Smaco|AIY31250.1:0.26026084,Smaco|KT862218:0.03217588)1.000000:0.13592591)1.000000:0.20369476)0.999985:0.19371733,((Smaco|KY086298:0.34827099,Smaco|YP_009252308.1:0.78582087)0.858083:0.03638697,((Smaco|KP233189:0.21182162,(Smaco|YP_009022025.1:0.16890409,((Smaco|YP_009030025.1:0.02787190,(Smaco|KJ577810:0.01258018,Smaco|YP_009054985.1:0.11812688)0.991240:0.02381405)1.000000:0.09997075,(Smaco|KX838318:0.05691841,(Smaco|AMR73073.1:0.04872665,Smaco|KX838317:0.11567330)0.999928:0.06926819)1.000000:0.16893347)0.864019:0.03344059)0.992798:0.05719416)1.000000:0.21297589,((Smaco|AIY31246.1:0.51926591,((Smaco|YP_009163761.1:0.31012266,Smaco|YP_009054993.1:0.15956188)0.999998:0.14265105,(Smaco|AMR73071.1:0.29110622,(Smaco|KU043403:0.37004428,(Smaco|YP_009118278.1:0.65471066,(Smaco|ADB24799.1:0.00000085,Smaco|GQ351275:0.04170799)1.000000:0.32508563)0.794551:0.09739665)0.990511:0.08969640)0.999912:0.12837868)1.000000:0.17773925)0.902229:0.07635986,((((Smaco|YP_009054987.1:0.00000045,Smaco|KJ577813:0.00448721)0.999974:0.07318220,(Smaco|YP_009118276.1:0.10949826,Smaco|KU043428:0.07439763)0.999997:0.08498067)1.000000:0.24640748,(Smaco|KU043422:0.56060610,(Smaco|KU043430:0.20497463,Smaco|KU058671:0.29572928)1.000000:0.23069880)0.870090:0.04993991)0.961852:0.06451027,(Smaco|KU043420:0.60948701,Smaco|YP_009252320.1:0.44054676)0.875292:0.10387303)0.999900:0.09754966)0.999251:0.09995890)1.000000:0.12061214)1.000000:0.21924265)0.458714:0.13056907)0.989085:0.26958616)0.999973:0.49067649)1.000000:0.73732025)0.521396:0.06127297)1.000000:0.49854278)0.773452:0.13707611)1.000000:0.20759053,(((pCRESS1|WP_026669310.1:0.06006105,pCRESS1|WP_026524352.1:0.06337946)1.000000:0.58516844,((pCRESS1|WP_053982727.1:0.74486764,(pCRESS1|CDF01935.1:0.65641895,(pCRESS1|CUO57637.1:0.33627392,pCRESS1|CUO23215.1:0.21276571)1.000000:0.16961373)0.950927:0.09033289)0.999959:0.08755941,(pCRESS1|CVH76026.1:1.28404734,((pCRESS1|WP_003102166.1:0.06342853,(pCRESS1|WP_029176105.1:0.13164615,pCRESS1|WP_000032131.1:0.12196882)0.913476:0.04109939)1.000000:0.22360766,(pCRESS1|WP_029694263.1:0.29512841,(pCRESS1|WP_062004798.1:0.15531613,(pCRESS1|WP_003030931.1:0.06781309,(pCRESS1|WP_029690610.1:0.15473573,pCRESS1|WP_047207334.1:0.10214167)0.990308:0.03073807)1.000000:0.16543382)0.997111:0.07683386)0.999958:0.13760008)1.000000:0.33745572)0.775221:0.10977374)0.999994:0.19003294)1.000000:0.51155595,((pCRESS2|WP_036328238.1:0.59871873,(pCRESS2|SCH60086.1:0.60881782,((GasCSVlike|YP_007517186.1:0.10085584,GasCSVlike|YP_009126903.1:0.17866807)1.000000:0.97712923,((((pCRESS2|WP_037404274.1:0.44066996,(((((pCRESS2|WP_038350939.1:0.18802272,(pCRESS2|KJZ87129.1:0.12070593,pCRESS2|WP_023977019.1:0.17668348)1.000000:0.12548670)0.999026:0.07672498,pCRESS2|WP_018597672.1:0.27311668)0.623490:0.03473430,(pCRESS2|SCH17786.1:0.18806895,(pCRESS2|CDC44519.1:0.27110504,pCRESS2|CUP05665.1:0.21471243)0.997932:0.08314625)1.000000:0.14671985)0.880635:0.02844799,pCRESS2|BAK32345.1:0.37612016)0.999213:0.05284392,(pCRESS2|WP_044928503.1:0.34921827,pCRESS2|WP_053167095.1:0.47060906)0.568860:0.03778666)0.999990:0.05724629)0.912708:0.03316409,(pCRESS2|CDE72464.1:0.36928824,(pCRESS2|CDB27189.1:0.49164177,pCRESS2|WP_020072285.1:0.60377981)0.769824:0.06042246)0.788499:0.02916162)0.991296:0.04083027,((pCRESS2|WP_051639324.1:0.48787118,((pCRESS2|WP_066550639.1:0.30219246,(pCRESS2|WP_007865724.1:0.07100273,pCRESS2|WP_013270924.1:0.07288207)1.000000:0.27097682)1.000000:0.19265678,((pCRESS2|WP_024346025.1:0.11721509,pCRESS2|WP_038278663.1:0.13633549)1.000000:0.20007977,(pCRESS2|EES75484.2:0.75680036,((pCRESS2|WP_009246639.1:0.21608574,pCRESS2|CCY69022.1:0.14061994)1.000000:0.11929666,(pCRESS2|WP_052011064.1:0.28304429,pCRESS2|CCX75435.1:0.22063683)1.000000:0.12978083)0.888455:0.04176925)0.807980:0.04874445)1.000000:0.09988147)0.859730:0.03506026)0.996104:0.06068715,(pCRESS2|WP_051600858.1:0.63212724,(pCRESS2|WP_013978550.1:0.53612111,(pCRESS2|WP_021882760.1:0.41888704,(pCRESS2|CBL15233.1:0.36353104,(pCRESS2|WP_009301216.1:0.76235340,(pCRESS2|CCZ45692.1:0.20866625,(pCRESS2|WP_044942941.1:0.05297598,pCRESS2|WP_021629801.1:0.03140999)1.000000:0.13052645)0.999990:0.15329975)0.979139:0.07303479)0.647909:0.05606317)0.927223:0.06323184)0.866798:0.07093991)1.000000:0.23002685)0.984504:0.04156687)0.925779:0.06108936,(pCRESS2|WP_051546484.1:0.66941428,(pCRESS2|WP_066546553.1:0.21153181,pCRESS2|WP_013271491.1:0.19275707)1.000000:0.62009862)0.994087:0.11052528)0.995741:0.09551801)0.712346:0.06562343)0.621478:0.05358608)0.995413:0.11621662,((((pCRESS3|WP_055838650.1:0.75668623,(pCRESS3|WP_016667133.1:0.59715247,(pCRESS3|WP_002529618.1:0.42347234,pCRESS3|WP_036342632.1:0.78710141)0.771570:0.13152012)0.999959:0.23237649)0.962414:0.16502003,(((pCRESS3|WP_023022037.1:0.43971599,pCRESS3|AKO38848.1:0.80572796)1.000000:0.28887482,(pCRESS3|NP_613078.1:0.72401380,(pCRESS3|WP_025221073.1:0.73648062,pCRESS3|WP_052119337.1:0.87194185)0.492312:0.06336689)0.644502:0.12341488)0.824606:0.08022555,((pCRESS3|WP_021975256.1:0.36350824,(pCRESS3|WP_052825216.1:0.24706445,(pCRESS3|KFI81686.1:0.19020918,pCRESS3|WP_043170238.1:0.14412242)1.000000:0.15552128)0.999990:0.12074724)1.000000:0.18675242,pCRESS3|KFI87454.1:0.69205607)0.773526:0.06180594)0.993843:0.11022189)0.956908:0.11411471,pCRESS3|WP_033495900.1:1.26430663)0.651509:0.09690589,pCRESS3|WP_022856850.1:0.73394409)1.000000:0.35423686)1.000000:0.21525069)0.999948:0.18734104)0.999927:0.29172909)0.976596:0.18114490)0.933531:0.15242242)1.000000:0.93928446)0.983726:0.10750058)0.999984:0.12197600)0.999952:0.15758155)1.000000:0.35928000);
