# Supplementary material for: Multiple origins of prokaryotic and eukaryotic single-stranded DNA viruses from bacterial and archaeal plasmids
Source: Nat Commun. 2019 Jul 31;10:3425. doi: 10.1038/s41467-019-11433-0 (PMC6668415; doi:10.1038/s41467-019-11433-0)
Supplement: Supplementary file 7 — Dataset 6 [file 41467_2019_11433_MOESM7_ESM.docx]

**SUPPLEMENTARY DATA 6**

# PHYLOGENETIC TREE SHOWN IN FIGURE S5A

((((((((((((((((pE194_pMV158-like|UniRef50_A8W662:0.4923233495,pE194_pMV158-like|3DKX_A:0.1702541708)95.6/100:0.2953575418,pE194_pMV158-like|UniRef50_A0A1Y4QQC0:0.4437918241)99.4/100:0.7461688346,((pE194_pMV158-like|UniRef50_A0A0E9F9L7:0.5745611130,pE194_pMV158-like|UniRef50_A0A174GG61:1.0731929923)88/100:0.3094709849,pE194_pMV158-like|UniRef50_W1I697:0.9192231189)99.5/100:0.8071296041)95.3/100:0.5234283772,(pE194_pMV158-like|UniRef50_A0A0H5Q8X5:1.2088631266,((pE194_pMV158-like|UniRef50_U2TJ01:0.8574876110,pE194_pMV158-like|UniRef50_S6CES9:1.9690263869)34.5/71:0.1909989682,pE194_pMV158-like|UniRef50_A0A1Y3UDM3:0.9660332761)68.5/82:0.2413566834)64.3/82:0.2031038820)83.4/95:0.1500274843,(((pE194_pMV158-like|UniRef50_A0A0E9EV38:0.4727551097,pE194_pMV158-like|UniRef50_A0A0H5Q0X0:0.6593764292)94.4/100:0.1927708322,(pE194_pMV158-like|UniRef50_K9RZV9:0.5453604225,pE194_pMV158-like|UniRef50_K9RYD5:1.2880526354)90.8/100:0.2657317180)79.1/100:0.1099412868,pE194_pMV158-like|UniRef50_U2EU97:0.3829742454)90.5/100:0.2052461538)95.1/94:0.1917982988,(pE194_pMV158-like|UniRef50_U2QZX4:1.1815707166,pE194_pMV158-like|UniRef50_UPI000481AAFB:1.4935813362)4.7/93:0.1030116100)87.8/91:0.1834962493,(pE194_pMV158-like|UniRef50_K7YFJ8:0.9350784670,pE194_pMV158-like|UniRef50_O31070:0.4785426002)99.6/100:0.6405480128)88.8/90:0.1517462958,(pE194_pMV158-like|UniRef50_A0A0R3QHC2:0.6964559716,(pE194_pMV158-like|UniRef50_W7D2V3:0.5785268530,pE194_pMV158-like|UniRef50_S6F6F1:1.2636111130)86.7/93:0.2135630605)73.6/87:0.2072816480)84.8/83:0.1001123370,((pE194_pMV158-like|UniRef50_W1I557:0.4892622377,pE194_pMV158-like|UniRef50_A0A0Z8IYX5:1.2082926511)93/93:0.2393358675,(pE194_pMV158-like|UniRef50_A0A1B1IHL4:1.4707116413,(pE194_pMV158-like|UniRef50_A0A0R1P770:1.2641646204,pE194_pMV158-like|UniRef50_Q48831:1.4323971833)70.2/83:0.1757257877)5/73:0.0248376473)91.4/84:0.1659609844)91.4/95:0.1403649058,pE194_pMV158-like|UniRef50_A0A0H5PZA0:1.0428776247)50.2/91:0.0480107496,((pE194_pMV158-like|UniRef50_A0A0H5PZI7:0.7832718361,pE194_pMV158-like|UniRef50_G8CNR9:0.6933375002)92.5/100:0.2175094957,(pE194_pMV158-like|UniRef50_A0A1W6BZG0:0.8252586328,pE194_pMV158-like|UniRef50_UPI000300949E:0.8605188029)56.5/93:0.1081458673)83.4/92:0.1693942714)98/100:0.2392538061,(pE194_pMV158-like|UniRef50_A0A0H5PZW4:0.8515616172,pE194_pMV158-like|UniRef50_A0A0H5PV05:0.9399504818)88.5/98:0.1807112590)88.5/81:0.1047465789,(((pE194_pMV158-like|UniRef50_A0A1C6AUS2:0.4944370738,pE194_pMV158-like|UniRef50_A0A0E9DRD0:0.5101874173)93.9/100:0.2251667291,(pE194_pMV158-like|UniRef50_G8CNT2:0.3966439928,pE194_pMV158-like|UniRef50_A0A0E9F4G4:0.8171112595)99.8/100:0.5304241275)53.4/93:0.0993602844,pE194_pMV158-like|UniRef50_A0A158LH93:0.8804763147)75.3/58:0.0897358943)16.5/13:0.0207149299,(pE194_pMV158-like|UniRef50_A0A0H5PZG0:0.8507100387,pE194_pMV158-like|UniRef50_A0A0H5QIL6:0.6885723401)99.5/100:0.6097253811)61.9/20:0.0506403409,(pE194_pMV158-like|UniRef50_A0A1Y4G0S9:0.8724425108,(((pE194_pMV158-like|UniRef50_A0A087EKU7:0.2169912659,pE194_pMV158-like|UniRef50_A0A0S2MGE2:0.3509407068)54.3/88:0.0945446709,pE194_pMV158-like|UniRef50_D3R6U9:0.2276612775)100/100:1.1259598311,pE194_pMV158-like|UniRef50_F0HMF3:1.0194002290)81/99:0.2325337010)67.5/69:0.1207470658)100/100:0.5941455445,((((((((((((Gemini|ADN84041.1:0.1439673264,(Gemini|AAN76737.1:0.1007745394,(Gemini|YP_001333687.1:0.1586979190,Gemini|YP_115511.1:0.0954541277)100/100:0.3036738788)85/94:0.0571442735)99.1/97:0.0954571719,((((Gemini|AAB87607.1:0.0953817350,Gemini|AGK24653.1:0.1509893997)99.9/100:0.1410274726,Gemini|APP87725.1:0.3867825111)52.3/93:0.0582550923,((((((((Gemini|ABG90906.1:0.0320376852,Gemini|ACY79450.1:0.2507627109)100/100:0.1957161236,Gemini|AAP73446.1:0.3191579669)78.5/94:0.0590469637,Gemini|YP_001040016.1:0.1070675688)87.5/93:0.0353605333,((Gemini|YP_002224032.1:0.2563333992,Gemini|CBJ17676.1:0.0731542355)89.8/100:0.0324362433,Gemini|AMK07575.1:0.2265169914)92/100:0.0399238575)100/97:0.0952392981,Gemini|YP_003622552.1:0.2440154729)71.4/94:0.0407978418,Gemini|YP_764516.1:0.1446705483)91.9/90:0.0296566010,((((((((Gemini|ACB44970.1:0.0747435079,Gemini|AGJ03640.1:0.2062172343)100/100:0.1253841235,Gemini|AHA82274.1:0.1919681730)34.7/68:0.0445465826,(Gemini|AGV02071.1:0.1767564015,((Gemini|AAF75542.1:0.1469661470,Gemini|NP_050017.1:0.1368363569)83.8/100:0.0413360532,Gemini|ACV60535.1:0.1255407893)95.7/100:0.0352999036)75.7/100:0.0151315695)92.6/98:0.0371861820,(Gemini|AFA26437.2:0.0689928307,(((Gemini|AFB81519.1:0.1370961707,Gemini|ACI06063.1:0.1431730656)96.9/95:0.0434899249,Gemini|AFH68197.1:0.1382928452)0/85:0.0009751305,Gemini|AFB83419.1:0.1627835174)100/100:0.0748652466)58.5/87:0.0191982929)97.4/98:0.0448792658,(Gemini|AGG08895.1:0.1140856461,(Gemini|YP_006905839.1:0.1844074639,Gemini|BAF02752.1:0.1995842110)32.6/64:0.0274309779)77.5/60:0.0231898810)87.3/58:0.0163826530,(((Gemini|CAJ85998.1:0.0908083532,Gemini|AGF41094.1:0.0855057649)92.1/93:0.0464821851,Gemini|AFF58888.1:0.1818656159)57/87:0.0177378422,Gemini|AHL29198.1:0.0902506729)94.5/83:0.0316352248)93.7/75:0.0255030052,((((Gemini|CDW92215.1:0.1383587756,((Gemini|AAX39336.1:0.0249902584,Gemini|FM877473:0.0288752149)99.7/100:0.0820844981,Gemini|AEY63664.1:0.2213683027)99.7/100:0.1012990083)66.9/99:0.0379696455,Gemini|YP_008411025.1:0.3068249346)0.8/46:0.0081396073,Gemini|NP_620741.1:0.1620655792)52.8/96:0.0355212336,Gemini|ABD35287.1:0.2565572830)43.3/50:0.0146649118)90.6/97:0.0269582226,((Gemini|ADW24243.1:0.2103709301,(Gemini|AKS48121.1:0.1437006218,Gemini|ABD67440.1:0.3553774263)84/100:0.0774187375)98.7/100:0.0872295636,(Gemini|AIY31184.1:0.1511852728,Gemini|AEE99005.1:0.2811819147)95.9/100:0.0670632615)94/94:0.0402017836)88.6/66:0.0235138755)32.3/54:0.0100381084,((Gemini|YP_009129272.1:0.2460002448,Gemini|CAM91896.1:0.1348363123)39.4/88:0.0329632999,(Gemini|AJM13604.1:0.1474177549,Gemini|YP_004958233.1:0.1930242514)99.4/100:0.0928677566)100/100:0.1075512929)92.2/63:0.0310799699)48.8/65:0.0237715411,(Gemini|YP_001285764.1:0.2734905600,Gemini|AMP46444.1:0.1879926521)99.7/100:0.0996013890)95.3/99:0.0593231641)100/97:0.1683422924,(((((Gemini|NP_671468.1:0.1779643139,Gemini|AFD54490.1:0.1769365801)81.7/96:0.0330772849,Gemini|AHX57826.1:0.2181032391)0/92:0.0022579109,Gemini|YP_007250561.1:0.2219261961)81.3/81:0.0253836420,(((Gemini|AMW86999.1:0.2052427887,Gemini|CBH28932.1:0.1815269329)48.7/94:0.0101254078,((Gemini|CBA18089.1:0.1200870254,Gemini|FJ665283:0.0906702674)49.2/100:0.0249876159,Gemini|ALV85583.1:0.1689452258)99.1/99:0.0497450491)71.9/95:0.0116177831,Gemini|AGH29892.1:0.0858434368)99.5/100:0.0662220168)87.9/82:0.0260610915,((Gemini|YP_002941855.1:0.1493398202,Gemini|YP_006590064.1:0.1294451996)45.2/97:0.0235848842,(Gemini|ACV83312.1:0.1268439827,Gemini|AER09339.1:0.1304689233)99.1/100:0.0722728370)94/98:0.0331049173)87.2/82:0.0257097137)89.3/76:0.0306060253,(Gemini|YP_619883.1:0.1933670339,(Gemini|NP_040557.1:0.1899344921,(Gemini|ALF37659.1:0.2130095538,(((Gemini|YP_003778178.1:0.0133819837,Gemini|KC108902:0.0538807254)100/100:0.2142887486,Gemini|YP_009226627.1:0.1000267613)88.7/100:0.0547695814,Gemini|ALR86823.1:0.2671953512)99.8/100:0.1703813124)92.7/100:0.0937694871)99.8/100:0.1321124636)73/91:0.0144392722)99.9/92:0.1466275161,((Gemini|AGV02076.1:0.1549809358,Gemini|AAL96826.1:0.2009196446)78.4/13:0.0247216901,((Gemini|YP_003828907.1:0.1598945509,Gemini|CRI68211.1:0.2464774731)81.5/98:0.0456443519,((Gemini|NP_066185.1:0.1554802780,Gemini|YP_003966137.1:0.1430931780)97.1/100:0.0799931314,Gemini|AFM38721.1:0.2631128647)94.1/100:0.0543712172)36.1/11:0.0117509566)99.6/95:0.1551907880)99.4/100:0.2752660010,(Gemini|ACO88014.1:0.0159097277,Gemini|FJ665634:0.0104649860)100/100:0.8002461827)99.2/100:0.2996934611,(((((((Gemini|AFV91331.1:0.3314492395,Gemini|AIT39773.1:0.5478771283)17.2/77:0.0695006949,(Gemini|YP_006666531.1:0.3043885808,Gemini|YP_006666527.1:0.5497135529)90.3/89:0.1126011839)90.5/98:0.1139519582,((Gemini|YP_003915159.1:0.2431261180,Gemini|AFN80669.1:0.3614100459)98.9/100:0.2044786589,((Gemini|AFN80601.1:0.1863486537,(Gemini|YP_004089627.1:0.1427822398,Gemini|YP_006666523.1:0.2062189964)95.2/100:0.0590220923)87.6/100:0.0860377274,Gemini|YP_006666535.1:0.1471541128)100/100:0.3454658079)100/100:0.3509517044)30.3/86:0.0831182076,((((Gemini|AAK73446.1:0.0053654359,Gemini|AF003952:0.0203183197)100/100:0.2671676809,((Gemini|AHM88378.1:0.1969667660,Gemini|Q80GM6.2:0.2160784284)98.8/100:0.1306397855,((Gemini|AHM88382.1:0.1465896261,Gemini|P0C647.1:0.3574464605)24.8/34:0.0496387449,Gemini|YP_003288768.1:0.2027890849)87.2/35:0.0520222223)74.4/100:0.0815687107)100/100:0.1879679627,Gemini|AHM88370.1:0.4824016625)71.8/87:0.0761944822,(Gemini|YP_006273070.1:0.4167095118,Gemini|YP_009026388.1:0.5702144436)74.9/86:0.1013399826)96.8/100:0.1465689467)72.3/99:0.1106310058,Gemini|YP_009021763.1:0.5371765280)92.3/100:0.1565126299,Gemini|DQ458791:0.5100662589)99.4/100:0.3157305456,(Gemini|KT214373:0.1617936268,Gemini|JX094280:0.2195684074)100/100:0.5750981816)95/100:0.2521073607)97/100:0.2346893435,(((((Genomo|KM598389:0.6814520878,Genomo|KJ547627:0.6561744036)99.9/100:0.4550358148,(Genomo|KJ547626:0.9155126414,(Genomo|KJ547634:1.0339871230,((((((((((Genomo|YP_009115514.1:0.2720025364,Genomo|YP_003104796.1:0.2602394828)99.5/100:0.1771972048,((((Genomo|YP_009252368.1:0.1255648855,Genomo|YP_009021043.1:0.0589024208)100/100:0.3107664336,Genomo|YP_009252356.1:0.1938740110)49.3/79:0.0518411413,(Genomo|YP_009115515.1:0.1440363711,((Genomo|YP_009115519.1:0.0976823821,Genomo|KJ547638:0.0838971718)24.9/79:0.0507187137,Genomo|AIF34843.1:0.1844037230)95.3/100:0.0560832597)99.9/100:0.1607544929)98.4/100:0.0910765910,(Genomo|YP_009252353.1:0.1977149866,Genomo|AMH87666.1:0.4171446130)86.7/97:0.0873306988)83.3/95:0.0577088228)50.3/93:0.0352261115,Genomo|AGS12486.1:0.4393655650)99.8/100:0.1659648960,(Genomo|AMH87733.1:0.4596719608,(Genomo|YP_009109727.1:0.4169920500,Genomo|YP_009252362.1:0.3918050527)99.1/100:0.2184071298)65.3/93:0.0882906523)18.6/86:0.0518794248,(((((Genomo|YP_009252365.1:0.2479276826,Genomo|YP_009252359.1:0.1848724139)99.8/100:0.1431920726,Genomo|KT253577:0.2157955046)91.3/100:0.0736393727,Genomo|AMH87678.1:0.2702828261)99.1/100:0.1625523688,(Genomo|YP_009109733.1:0.1070850159,Genomo|KT862241:0.0690147845)100/100:0.3008730137)98.7/100:0.1667765077,((Genomo|YP_009181999.1:0.0116854480,Genomo|KT598248:0.0000028352)100/100:0.4911763743,Genomo|YP_009109729.1:0.7017745744)96/98:0.1927984355)98.5/92:0.1534845897)79.5/92:0.1332954983,Genomo|AMH87702.1:0.6110535697)86.5/75:0.0637901900,Genomo|YP_009164036.1:0.6746299742)31.9/53:0.0626326780,(Genomo|AJD07464.1:0.3164114384,Genomo|AMH87693.1:0.3171576633)100/100:0.5156197735)60.5/65:0.0695820693,Genomo|AMH87708.1:0.3000032503)93.7/67:0.2010024348,Genomo|YP_009109725.1:0.5919871563)99.8/100:0.5722090617)91.6/100:0.2376630276)31.7/92:0.1111510779)82.9/97:0.1847938497,(Genomo|YP_009351871.1:1.2871144382,(Genomo|KJ938716:0.3854255257,Genomo|KM821748:0.2405250759)100/100:1.1036944609)22.7/19:0.1258424045)88.4/20:0.1467115097,Genomo|KP153522:1.1983219954)9.3/21:0.0799603092,Genomo|AUM61807.1:1.0655006241)94.3/100:0.2397090764)86.5/100:0.2912741159,((((((((pCRESS9|YP_007008175.1:0.0412854468,pCRESS9|WP_015083745.1:0.0377274834)74.3/100:0.0469121770,pCRESS9|YP_001708784.1:0.1179576413)91.2/100:0.0374202792,pCRESS9|ATL14544.1:0.0203941834)99.9/100:0.1439157216,(((pCRESS9|YP_001965310.1:0.1449052391,pCRESS9|YP_001965305.1:0.3145949579)75.2/98:0.0502199079,(((pCRESS9|YP_001708790.1:0.0949905805,pCRESS9|YP_007008179.1:0.0599691319)92.1/100:0.0360056331,pCRESS9|WP_013747472.1:0.0715310972)94.8/100:0.0346968147,(((pCRESS9|WP_011412958.1:0.0000026939,pCRESS9|ABC65268.1:0.0791401935)99.4/100:0.0794243473,pCRESS9|ABC65385.1:0.2842350496)85/99:0.0223607229,(pCRESS9|WP_011412950.1:0.0445218970,pCRESS9|CBX25033.1:0.0287094631)100/100:0.2366090205)90.4/99:0.0276892732)94.7/99:0.0445435523)89.6/96:0.0415503739,pCRESS9|YP_006961991.1:0.0847913328)98.5/98:0.0760768356)94.4/99:0.1236345954,(pCRESS9|WP_017193171.1:0.0272323212,pCRESS9|WP_017193695.1:0.0720247473)99.5/100:0.2194093929)98.2/100:0.3570774354,((((pCRESS9|YP_006959585.1:0.0000029304,pCRESS9|WP_015060110.1:0.0028482834)0/55:0.0000024630,pCRESS9|WP_011264167.1:0.0086254980)94.3/97:0.0192740060,(pCRESS9|BAD36752.1:0.0056898446,pCRESS9|WP_042068233.1:0.0000024103)99.3/100:0.0372778742)19.6/86:0.0157264309,pCRESS9|WP_012662291.1:0.0397444685)100/100:1.1127506485)91.4/100:0.2653569714,pCRESS9|KXT29032.1:1.4691059699)95.4/100:0.4121112201,pCRESS9|KXT29014.1:1.3317814383)78.2/98:0.4220398844)92.4/100:0.2995654518,((((CRESSV6|KT732829:0.8350052584,(CRESSV6|KM510189:0.6012167386,CRESSV6|KP005454:0.5057685120)100/100:0.8561897934)98.4/100:0.3705440718,(CRESSV6|KM874358:0.6828656935,CRESSV6|AJD07486.1:0.7634655891)98.9/100:0.4071384257)49/86:0.1099949486,((CRESSV6|KT149395:0.7873781393,CRESSV6|KM598390:0.7865768800)89.9/100:0.2063916503,CRESSV6|KP153501:0.7890731657)8.2/89:0.1358338935)99.9/100:0.6106378479,((((PpulchraPlasmids|OLY79419.1:0.3165536780,PpulchraPlasmids|OLY79389.1:0.3126742745)100/100:0.4409288857,((((PpulchraPlasmids|OMJ21113.1:0.0664479767,(PpulchraPlasmids|OMJ28371.1:0.1339662874,PpulchraPlasmids|OMJ13215.1:0.0260837320)67.5/100:0.0962717709)99.1/100:0.3477008135,PpulchraPlasmids|OMJ11569.1:0.4180610031)90.7/100:0.1448635986,PpulchraPlasmids|OMJ09562.1:0.7777154024)93.8/100:0.1424739928,(PpulchraPlasmids|OLY79699.1:1.2484333972,(PpulchraPlasmids|AAF36424.1:0.1714923123,(PpulchraPlasmids|AAF36423.1:0.0842926749,PpulchraPlasmids|AAF36422.1:0.0563005164)86.7/99:0.1218129032)100/100:1.4397888495)66.9/90:0.1477471360)77.3/91:0.0854187045)91.7/93:0.1788257334,PpulchraPlasmids|ETO15557.1:0.8050542678)89.1/99:0.2297945823,(((CRESSV6-Wastewater|AUM61713.1:0.3075980964,(CRESSV6-Wastewater|AUM61624.1:0.0145830042,CRESSV6-Wastewater|AUM62043.1:0.0051523579)100/100:0.4891227156)68.7/99:0.1398145502,CRESSV6-Wastewater|AUM61719.1:0.3838336183)11.9/68:0.1283415017,CRESSV6-Wastewater|AUM61738.1:0.7974228888)99.6/100:0.5003802249)95.6/99:0.2809581843)99.4/100:0.6325178399)100/100:0.8781007423,(((((((((((((Circo|YP_007974237.1:0.2440011848,Circo|AAZ78351.1:0.2921392728)92.6/100:0.1200598224,(Circo|AIF76280.1:0.0155992013,Circo|KJ641742:0.0088113525)100/100:0.3085431493)100/100:0.4586326278,Circo|AKO84203.1:0.5189794375)63.7/99:0.1478470838,(Circo|AGL09969.1:0.1679868240,Circo|YP_009021891.1:0.1578794270)100/100:0.3880046386)95.8/91:0.1535088942,((((Circo|AIF76265.1:0.1863985073,Circo|AIF76253.1:0.2192944528)78.8/100:0.0876818807,Circo|AIF76248.1:0.1749668330)93.2/100:0.1549384727,Circo|AIF76261.1:0.3866688818)100/100:0.4175836597,(Circo|YP_009170674.1:0.6408970056,(((Circo|AFL02442.1:0.4804587875,(Circo|ADU77009.1:0.1801618521,((Circo|YP_764455.1:0.1515447307,Circo|NP_573442.1:0.2262603672)89.7/75:0.0566057973,(Circo|KU230452:0.1037464037,Circo|YP_009134739.1:0.2107181568)49.4/73:0.0186962112)94.1/76:0.1028822407)96.2/100:0.1556368273)83.5/95:0.1025948232,(Circo|YP_803546.1:0.3677303987,Circo|AEL28794.1:0.4280445404)59.1/92:0.0547544996)99.3/98:0.2295185825,(Circo|ABU48445.1:0.1529616505,Circo|AHK80894.1:0.1832529102)100/100:0.5517982884)94.5/99:0.1774283623)92.7/95:0.1222839624)17.2/41:0.0453944052)97/98:0.1393906867,((Circo|ADD62475.1:0.5298710456,Circo|YP_004376332.1:0.5952118914)95.1/99:0.1499224050,Circo|YP_009091696.1:0.5346685058)87.5/97:0.0864535154)90.4/98:0.1043796332,Circo|YP_009000900.1:0.6591355094)95.7/98:0.1443113884,((((((((((((Circo|ADD62451.1:0.1743668973,Circo|ADD62455.1:0.2286786571)80.6/100:0.0683770344,((Circo|ADU77011.1:0.2106115769,Circo|AGJ74758.1:0.2866992205)43.8/97:0.0644467442,Circo|ADD62461.1:0.3117424815)11.3/95:0.0508208725)79.5/100:0.0642982327,Circo|AKE49355.1:0.2942839021)89.8/42:0.0733735995,((Circo|AGJ74756.1:0.3555378188,Circo|YP_009110680.1:0.2004065248)91.7/49:0.0658279528,(Circo|ADD62457.1:0.2946627964,((Circo|AIF76266.1:0.2828301191,Circo|ADD62473.1:0.1269834992)98.9/100:0.1220314196,Circo|AEL87792.1:0.2819108993)98/100:0.1217974727)61.3/96:0.0453643201)75.2/49:0.0703246078)83.6/21:0.0463558323,(Circo|AGJ74760.1:0.2688922271,Circo|ADY17982.1:0.3835043214)92.2/75:0.0686259014)93.2/22:0.0576204504,(((Circo|YP_009021843.1:0.4137628935,((Circo|AEL87786.1:0.2707872549,Circo|ADI48251.1:0.2574131089)94.8/99:0.0894942315,Circo|AEL87790.1:0.2213760588)94.3/93:0.0789392674)57.8/11:0.0287465588,(Circo|YP_004152331.1:0.2857643630,(Circo|ADD62471.1:0.2068094242,Circo|AIF76252.1:0.1409512364)95.2/100:0.0946346795)88.7/45:0.0393497176)83.5/12:0.0546522200,(Circo|ADD62453.1:0.3059931215,Circo|AIF76249.1:0.3558552784)97.4/99:0.1120076481)82.7/41:0.0406138807)95.3/86:0.0880486351,Circo|AIF76254.1:0.4382393145)82.1/100:0.0340959986,(Circo|YP_009021870.1:0.3689344459,Circo|AFS65290.1:0.3168612685)94.7/100:0.1286589727)98.8/100:0.2114075792,(((Circo|YP_009047065.1:0.2164177991,Circo|YP_008130363.1:0.2169426803)52.7/56:0.0879727943,Circo|ADD62477.1:0.1437388088)76.4/56:0.0834271745,Circo|ADU76993.1:0.2326270862)100/100:0.4326786495)93.8/100:0.1640603618,(Circo|AMH87650.1:0.0872195835,Circo|AMH87652.1:0.2647372390)100/100:0.9232268256)54.6/95:0.0722176161,(Circo|YP_009237526.1:0.7081588909,Circo|YP_009116910.1:0.7823188815)88.7/98:0.1532872304)83.1/97:0.0804195576,Circo|AIF76251.1:0.9668470262)91.3/83:0.1638843167)90.2/85:0.1456494093,Circo|KT732825:1.2115645278)98.3/100:0.2843705377,(((((CRESSV1|KM874347:0.8792237233,CRESSV1|KT149404:1.1224020868)76.2/75:0.1351268298,CRESSV1|FJ959078:0.9214941788)0/22:0.1096059387,CRESSV1|KP153497:1.4626088714)16.5/66:0.1357336265,(CRESSV1|KM874309:0.8804878923,CRESSV1|KF133822:0.7767354128)95.1/100:0.2675616233)62.8/84:0.1691756067,(((((CRESSV1|KT862256:0.3462438524,CRESSV1|KF246569:0.2713667735)100/100:0.8230848967,CRESSV1|KM573766:0.8462513122)90.6/100:0.1739934069,(CRESSV1|KJ206566:0.5154571450,CRESSV1|KU043411:0.3653808611)98.7/100:0.3603718131)23.6/98:0.1241268557,CRESSV1|KU043424:0.7258377143)99.9/100:0.6865562546,(CRESSV1|KX388513.1:0.0915688067,CRESSV1|KX388515.1:0.0000025171)100/100:1.8332027567)42.6/83:0.2348402563)94.1/100:0.2293312114)94.8/100:0.1604885657,(((((((((CRESSV3|JX904407:1.1346880448,CRESSV3|JX904075:0.3450640649)87.6/59:0.1040847863,CRESSV3|JX904076:0.3576623059)44.7/59:0.0754760515,CRESSV3|JX904139:0.3602141321)99.9/79:0.3266418577,(CRESSV3|KP153422:0.6069387243,CRESSV3|KM874300:0.9270634973)0.2/76:0.0190406826)90.3/78:0.0946503098,(CRESSV3|KM874317:0.5918964465,(CRESSV3|KM874304:0.5199606712,CRESSV3|JX904581:0.6371132474)28.5/85:0.0798604340)84.3/85:0.0817206262)87.5/90:0.0816380657,CRESSV3|KP153408:0.4556373190)95.3/91:0.1389949581,CRESSV3|KT149409:0.7903201972)68/89:0.1228147188,(CRESSV3|KM598406:0.6613687787,CRESSV3|KT149403:1.0057908011)34.4/84:0.0975387827)84.9/95:0.0814859383,(CRESSV3|KJ641729:1.0303731724,((((CRESSV3|JN857329:0.5155748408,CRESSV3|KJ641718:0.5090074402)97.5/98:0.2091338492,(CRESSV3|KM972726:0.7165571493,CRESSV3|KF738883:0.8502244993)86/84:0.1395863203)37.2/78:0.0542850414,(CRESSV3|KJ641722:0.2026701527,CRESSV3|HM228875:0.1657308870)100/100:0.7274461286)88.5/92:0.1369986192,(CRESSV3|JX185418:0.6534094899,CRESSV3|KM598404:0.9370327950)94.9/99:0.2426213154)8.7/58:0.0260543065)96/100:0.1595224907)99.6/100:0.2327321604)82.9/98:0.0785815780,(((((((((CRESSV2|KM821764:0.9323846403,CRESSV2|KP153468:0.8985386552)15.2/22:0.1026319346,(CRESSV2|KP153369:0.9252390293,CRESSV2|KP153483:0.9777881185)45.6/75:0.1594303380)94.1/99:0.1904657645,(CRESSV2|KT149394:0.5656772096,CRESSV2|KP153360:0.3912970664)100/100:0.6334706333)30.6/61:0.1019812932,((((((CRESSV2|KP153404:0.6763715029,CRESSV2|KC248416:0.4353610180)87/100:0.1470140549,CRESSV2|KT149398:0.8532859811)88.4/100:0.1029431653,(CRESSV2|KP153485:0.3412162904,(CRESSV2|KJ547648:0.2037580967,CRESSV2|KT149412:0.4044625108)81.8/99:0.1061375444)100/100:0.6066962346)95/100:0.1676154098,(CRESSV2|KP153447:0.6183181819,CRESSV2|KT732819:0.9055958377)58.2/11:0.0917672374)32.6/9:0.0465372611,((CRESSV2|KT732823:1.2925214916,CRESSV2|KM598396:0.5346820048)66.8/97:0.3002768835,CRESSV2|JX185415:0.5268922442)100/100:0.5661556133)90.9/11:0.1216132017,CRESSV2|FJ959082:0.7931361844)79.7/12:0.1467102385)45.8/6:0.0715321187,(CRESSV2|KM821755:0.8889434888,(((CRESSV2|KT732816:0.3792238517,CRESSV2|JF755415:0.2658901621)100/100:0.4156914250,CRESSV2|JX904344:0.5245746811)89.5/75:0.0803117789,((CRESSV2|KP153364:0.5751279339,CRESSV2|JX904185:0.4427582593)99.2/100:0.2582128711,CRESSV2|JX904420:0.6963686732)94.2/97:0.1242690895)62.6/74:0.0429496580)85.7/98:0.0905131743)69.2/11:0.0930850210,(CRESSV2|KP153377:0.8073222090,CRESSV2|KF738877:1.0523042393)96.7/100:0.3145057536)45.4/7:0.0713645794,(CRESSV2|JX904107:0.6297844213,CRESSV2|JX904562:0.4266146332)100/100:0.4620777094)77.9/6:0.0913713298,((CRESSV2|KU043397:0.6230591192,CRESSV2|KU043406:0.5603626048)93.8/100:0.2002185827,(CRESSV2|KM573776:0.3494618772,CRESSV2|KM573767:0.4123285771)95.3/100:0.2202983265)99.9/100:0.4122164263)100/100:0.5288738114,((((CRESSV4|YP_009163936.1:0.6563400413,(CRESSV4|AHH31482.1:0.4481924145,(CRESSV4|YP_009021888.1:0.7009419902,CRESSV4|YP_009237559.1:0.8675609432)98.7/100:0.3540380927)31.7/94:0.1338094305)96.7/100:0.2937159989,CRESSV4|KX388505.1:0.9956610170)93.9/100:0.2195131583,((((NanoAlpha|YP_003104737.1:0.0273535737,NanoAlpha|HE654123:0.0846697940)99.9/100:0.4134969712,(NanoAlpha|AKO71308.1:0.0000027792,NanoAlpha|JF957636:0.0190605891)100/100:0.4943793512)99.8/100:0.5805337316,(((((NanoAlpha|AAA51422.1:0.0680546403,NanoAlpha|ACB86656.1:0.3524717208)99.9/100:0.3335485463,NanoAlpha|AAA51426.1:0.2793369796)99.6/100:0.3077881864,(((NanoAlpha|YP_009058890.1:0.0747584728,NanoAlpha|KC978991:0.0586540493)100/100:0.3529936923,NanoAlpha|NP_619760.1:0.1930851361)99.7/100:0.2945861068,((NanoAlpha|YP_008169853.1:0.1036664395,(NanoAlpha|YP_009246456.1:0.1623219432,NanoAlpha|ALK03646.1:0.1943138190)98.7/100:0.1079593572)81.9/99:0.0468264324,NanoAlpha|HM163578:0.0476713337)100/100:0.9113020096)56.3/87:0.0774102306)92.8/89:0.1846612749,(NanoAlpha|U16735:0.2200033726,NanoAlpha|KC979052:0.2207155073)100/100:0.3537845597)84.8/95:0.1070500867,(NanoAlpha|NP_619759.1:0.4953086684,(NanoAlpha|KF471057:0.5951631769,NanoAlpha|JX458742:0.4556666865)99.9/100:0.3879835187)80.3/100:0.1332035965)98.7/100:0.3902809729)98/100:0.3708083428,NanoAlpha|AIF34798.1:0.7648027841)99.1/100:0.4297081040)91.3/97:0.1453962525,(((((CRESSV5|JX904231:0.7280558009,(((CRESSV5|KR528545:0.5867707004,CRESSV5|KR528553:0.7575666218)74.2/100:0.0937183092,CRESSV5|KT945163:0.8516268436)74.7/100:0.1252797485,CRESSV5|KM874354:0.6400576391)34.9/84:0.0933164759)32.2/84:0.0943577766,((CRESSV5|KR528554:0.4092933809,CRESSV5|KR528556:0.3900309386)94.3/100:0.1820800488,(CRESSV5|KR528551:0.5896657990,CRESSV5|KR528562:0.5591793129)94.2/100:0.2330177763)94.9/100:0.1558416980)68.6/75:0.0892887898,((CRESSV5|KR528561:0.6028015166,CRESSV5|KR528547:0.8027968575)81.4/100:0.0796478878,CRESSV5|KJ641738:0.7388317030)46.9/96:0.0636141441)99.1/99:0.2493569602,((CRESSV5|KP153451:0.7658472490,CRESSV5|KJ547650:0.7216564622)74.6/100:0.1964649849,CRESSV5|KJ547646:0.8915115540)96.2/100:0.2349219979)77.4/98:0.0822384213,(((((((Smaco|AIY31250.1:0.3605666282,Smaco|KT862218:0.0386122157)97.3/100:0.1847564315,Smaco|KT862221:0.1942425068)98.6/100:0.2883269275,(Smaco|KM573775:0.2723958574,Smaco|KM573771:0.3296519424)99/100:0.2898406507)96.9/100:0.2212824300,Smaco|YP_009252310.1:0.9304061488)0.8/19:0.1427209552,((((((Smaco|YP_009030025.1:0.0382391654,(Smaco|KJ577810:0.0169937091,Smaco|YP_009054985.1:0.1620756407)81.9/100:0.0325010868)99.3/100:0.1429880585,((Smaco|AMR73073.1:0.0639271504,Smaco|KX838317:0.1597883475)91.7/100:0.0894434511,Smaco|KX838318:0.0828254667)99.9/100:0.2248197641)64.3/97:0.0464871224,Smaco|YP_009022025.1:0.2289164365)81.6/97:0.0827290937,Smaco|KP233189:0.2874736626)99.5/100:0.2773712330,((Smaco|YP_009252320.1:0.7058444093,(((Smaco|YP_009054987.1:0.0000022308,Smaco|KJ577813:0.0060241991)95.6/100:0.1058249128,(Smaco|YP_009118276.1:0.1509228581,Smaco|KU043428:0.0986165085)95.3/100:0.1065667551)100/100:0.3213428912,(((Smaco|KU043430:0.2503385584,Smaco|KU058671:0.4217879230)99.4/100:0.2603173815,Smaco|KU043420:0.7934824487)8.9/69:0.0833509625,Smaco|KU043422:0.7439778861)89.4/82:0.1099482292)82.6/80:0.0785211039)94.6/95:0.1251259282,(Smaco|AIY31246.1:0.7019379054,((Smaco|YP_009163761.1:0.4241949058,Smaco|YP_009054993.1:0.2142916739)96.5/100:0.1994606893,(Smaco|AMR73071.1:0.3966826851,(((Smaco|ADB24799.1:0.0000025425,Smaco|GQ351275:0.0555874889)100/100:0.4480664805,Smaco|YP_009118278.1:0.8851748483)44.1/96:0.1305591451,Smaco|KU043403:0.5133805370)61.5/96:0.1144968173)91.6/98:0.1715932854)98.1/100:0.2281532697)59.4/94:0.1053390741)94.8/96:0.1528189532)95.6/96:0.1539948009,(Smaco|KY086298:0.4599015270,Smaco|YP_009252308.1:1.0888114349)81/91:0.0446397460)95.8/95:0.2333879438)85/88:0.4617546216,((Smaco|YP_009252314.1:0.4450478494,Smaco|AEW47007.1:0.3362796227)72.1/100:0.3441791058,Smaco|KM598409:0.7428841483)100/100:1.3437425989)95.1/100:0.6682719198,(((((Smaco|YP_009252316.1:0.6867272662,(Smaco|KU203352:0.2355029151,Smaco|KJ547633:0.2158992232)100/100:1.5012336966)84.6/95:0.1456615341,((Smaco|AJF23062.1:0.0828989023,(Smaco|AJF23060.1:0.0122363167,((Smaco|AJE25847.1:0.0071413383,(Smaco|AJE25851.1:0.0000024323,Smaco|AJE25845.1:0.2945788906)100/90:0.3360445785)77.4/78:0.0054399897,Smaco|KP233175:0.0000023585)100/78:0.2326106662)97.5/78:0.1069086105)86.6/79:0.1092863577,Smaco|KY086301:0.2001293548)100/100:0.6596373184)54/81:0.1264074986,Smaco|YP_009252326.1:0.6942455949)91.4/100:0.2964808198,Smaco|AJD07511.1:0.9064375646)95.7/100:0.3975770022,((Smaco|AIY31243.1:0.1994564340,Smaco|KT862224:0.4529480256)52.6/100:0.1590827404,Smaco|AIY31256.1:0.4315907661)99.8/100:0.8569883690)94.5/100:0.4904808719)99.8/98:0.9690600955)78.1/95:0.0859410533)100/100:0.6793344502)60/46:0.1879757293)97/99:0.3295785412,(((((((pCRESS1|CUO57637.1:0.4490410061,pCRESS1|CUO23215.1:0.2801342929)98/100:0.2251594155,pCRESS1|CDF01935.1:0.8815674942)66.7/99:0.1073598093,pCRESS1|WP_053982727.1:1.0335475369)87.2/100:0.1042466339,((pCRESS1|WP_003102166.1:0.0849480777,(pCRESS1|WP_029176105.1:0.1725536539,pCRESS1|WP_000032131.1:0.1605795243)41.5/99:0.0513625118)99.8/100:0.2938695831,((pCRESS1|WP_062004798.1:0.2101882438,(pCRESS1|WP_003030931.1:0.0883250700,(pCRESS1|WP_047207334.1:0.1335669837,pCRESS1|WP_029690610.1:0.1998494219)68.9/100:0.0397520218)99.9/100:0.2107179761)81.4/100:0.0978355812,pCRESS1|WP_029694263.1:0.3853086660)96.5/100:0.1870484703)100/100:0.5848537580)93.1/67:0.1895171861,(pCRESS1|WP_026669310.1:0.0780054708,pCRESS1|WP_026524352.1:0.0844621068)100/100:0.8426559297)55.5/54:0.1946513919,pCRESS1|CVH76026.1:1.6290640933)99.5/100:0.5820397288,((((pCRESS2|SCH60086.1:0.6575450096,(GasCSVlike|YP_007517186.1:0.1668042351,GasCSVlike|YP_009126903.1:0.2141657720)100/100:1.2789435310)67.5/32:0.1242250927,pCRESS2|WP_036328238.1:0.8361786163)67.5/31:0.1188274055,(((((((((((pCRESS2|WP_044942941.1:0.0728145748,pCRESS2|WP_021629801.1:0.0400639492)99.6/100:0.1795333111,pCRESS2|CCZ45692.1:0.2737456198)96.7/100:0.1959068312,pCRESS2|WP_009301216.1:1.0574531575)83.3/100:0.0983840672,pCRESS2|CBL15233.1:0.5069694145)3/60:0.1079015617,(pCRESS2|WP_021882760.1:0.4559214566,pCRESS2|WP_051600858.1:0.8541629852)79.3/99:0.1426050092)84.8/99:0.1037468730,pCRESS2|WP_013978550.1:0.6714225446)99.5/100:0.2528001236,pCRESS2|WP_020072285.1:0.7897559536)96.7/67:0.1176093312,(pCRESS2|CDE72464.1:0.4886692092,pCRESS2|CDB27189.1:0.6894425447)20.6/91:0.0367369366)83.9/66:0.0391079803,(((((((pCRESS2|WP_038350939.1:0.2624391654,(pCRESS2|KJZ87129.1:0.1632954267,pCRESS2|WP_023977019.1:0.2343851883)98.1/100:0.1683988921)95.5/100:0.1077236184,pCRESS2|WP_018597672.1:0.3500822198)30.5/96:0.0448518597,((pCRESS2|CUP05665.1:0.2864677029,pCRESS2|CDC44519.1:0.3720609265)79.6/100:0.1084494344,pCRESS2|SCH17786.1:0.2517583517)99.7/100:0.1961529680)54.6/96:0.0449800833,pCRESS2|BAK32345.1:0.5136954215)91.7/100:0.0678602828,pCRESS2|WP_044928503.1:0.4911742094)27.1/97:0.0370108789,pCRESS2|WP_053167095.1:0.6244724066)83.1/98:0.0576112083,pCRESS2|WP_037404274.1:0.6020194381)84.8/66:0.0375125631)87.8/60:0.0613173423,((((((pCRESS2|WP_009246639.1:0.2910276886,pCRESS2|CCY69022.1:0.1911833372)97.5/100:0.1588275663,(pCRESS2|WP_052011064.1:0.3783910698,pCRESS2|CCX75435.1:0.2984426892)98.2/100:0.1740410686)67.1/100:0.0525390815,pCRESS2|EES75484.2:1.0407668960)51.6/88:0.0621565543,(pCRESS2|WP_024346025.1:0.1527404931,pCRESS2|WP_038278663.1:0.1878020891)100/100:0.2714030490)97.6/100:0.1281513158,((pCRESS2|WP_007865724.1:0.0952843892,pCRESS2|WP_013270924.1:0.0956983013)100/100:0.3569582583,pCRESS2|WP_066550639.1:0.4092226107)99.8/100:0.2643548063)27.6/60:0.0360352200,pCRESS2|WP_051639324.1:0.6794660541)95.8/100:0.1194220851)84/83:0.0955701595,(pCRESS2|WP_051546484.1:0.9115706594,(pCRESS2|WP_066546553.1:0.2688844149,pCRESS2|WP_013271491.1:0.2703086424)100/100:0.8616243905)90.1/98:0.1673495014)95.7/99:0.1725257862)93.1/81:0.2076795960,(((((((pCRESS3|WP_055838650.1:1.0287205803,(pCRESS3|WP_016667133.1:0.7869463086,(pCRESS3|WP_002529618.1:0.5937412815,pCRESS3|WP_036342632.1:1.0769911423)48/96:0.1671994928)96.4/100:0.2982028510)96/88:0.2812317460,(pCRESS3|NP_613078.1:0.9725055803,(pCRESS3|WP_023022037.1:0.5799816543,pCRESS3|AKO38848.1:1.0750586309)96.5/96:0.2974100601)88.9/89:0.1584091383)35.8/6:0.0738740038,(pCRESS3|KFI87454.1:0.7627652012,pCRESS3|WP_052119337.1:1.2567250705)79.2/95:0.1498447100)85.6/6:0.0942025037,(pCRESS3|WP_021975256.1:0.4847156946,((pCRESS3|KFI81686.1:0.2500427078,pCRESS3|WP_043170238.1:0.1949881358)99.3/100:0.1977222362,pCRESS3|WP_052825216.1:0.3348598937)94.1/100:0.1572818957)99.2/100:0.2685186148)34.1/5:0.0958718094,pCRESS3|WP_025221073.1:1.1580367783)91.6/82:0.1838839775,pCRESS3|WP_022856850.1:0.9540947224)41.7/28:0.1475305928,pCRESS3|WP_033495900.1:1.7530376115)97.6/100:0.3824888370)94.5/100:0.2269475749)96.3/100:0.3070403121)95.2/100:0.3994523161)86.5/99:0.2523266820,((((((((((((pCRESS6|WP_034704841.1:0.0874083759,pCRESS6|WP_067483596.1:0.0680922640)100/100:0.2300306851,((((((((pCRESS6|WP_049499636.1:0.1204524775,pCRESS6|WP_045759092.1:0.0852492178)100/100:0.1754693858,pCRESS6|WP_039677656.1:0.2526648401)97.4/92:0.0652458551,(pCRESS6|WP_027972054.1:0.1753024150,(pCRESS6|WP_044774450.1:0.0543004249,pCRESS6|WP_020997784.1:0.0952754656)100/100:0.2098716701)40.2/90:0.0326977281)81.9/97:0.0212637121,(((pCRESS6|KXT86702.1:0.0693095786,pCRESS6|WP_032497992.1:0.2240927445)84.5/100:0.0387730567,(pCRESS6|WP_015647385.1:0.2207237904,pCRESS6|CGE81062.1:0.2553026357)16.3/50:0.0300421567)80.1/52:0.0247217748,pCRESS6|WP_014623544.1:0.1390573923)92/97:0.0291807524)93.8/99:0.0440414091,(pCRESS6|KEQ49321.1:0.1438044949,pCRESS6|WP_053092713.1:0.1998783736)98.3/100:0.0624929200)97.2/100:0.0682598879,(pCRESS6|WP_049476139.1:0.0905598463,pCRESS6|WP_003035134.1:0.0643730549)99.8/100:0.0970077296)95.6/100:0.0712827067,(pCRESS6|WP_017649267.1:0.1050812200,pCRESS6|ABJ73998.1:0.1798645356)99.8/100:0.0991737281)92.4/100:0.0639606682,pCRESS6|WP_003024533.1:0.3068676796)88.5/100:0.0877424117)96/100:0.1482114628,pCRESS6|ADX23728.1:0.5869310741)76.5/99:0.0740486674,pCRESS6|WP_056938517.1:0.5413672037)97.2/100:0.1460030217,(((((pCRESS6|WP_000201649.1:0.0741526191,pCRESS6|WP_047206721.1:0.0416994707)99.9/100:0.1289896996,pCRESS6|CMU27730.1:0.2380672454)99/100:0.1200874647,pCRESS6|WP_000044268.1:0.2736130699)98.9/100:0.1245854433,((((pCRESS6|WP_001034312.1:0.0550795747,pCRESS6|WP_024385235.1:0.0798997373)89.1/100:0.0269876263,pCRESS6|WP_004183001.1:0.0935585372)83.5/100:0.0298170188,pCRESS6|WP_024400359.1:0.1454187207)100/100:0.1953683715,(pCRESS6|WP_000791389.1:0.2243341703,pCRESS6|WP_003032217.1:0.5175266298)76.1/99:0.0556189180)89.5/99:0.0683167553)95.4/100:0.1347203524,((pCRESS6|WP_018376545.1:0.1179432874,pCRESS6|WP_020999261.1:0.1510148905)91.2/99:0.0676695226,pCRESS6|WP_044762265.1:0.1234195522)100/100:0.5306818624)97.8/100:0.1800580670)100/100:0.3016148200,((((pCRESS6|WP_054952722.1:0.0243950186,pCRESS6|WP_041290927.1:0.0362152061)99.7/100:0.1809991650,pCRESS6|WP_036321578.1:0.3835906524)99.9/100:0.3302130296,pCRESS6|WP_051176704.1:0.7455592664)98.1/100:0.2284034508,(pCRESS6|WP_052506726.1:0.8144233318,pCRESS6|WP_022765681.1:0.8409523843)49.9/97:0.1442861241)53.1/91:0.1456211274)96.8/98:0.2574791429,pCRESS6|AEU41945.1:0.8576529029)85.2/99:0.1255300591,pCRESS6|WP_014571792.1:1.0892620252)94.1/100:0.1628576117,((((pCRESS6|WP_025016923.1:0.0906350542,pCRESS6|KST89836.1:0.0812344983)100/100:0.4785747658,((pCRESS6|WP_018380019.1:0.1671650247,pCRESS6|EOB33201.1:0.1951441545)98.6/100:0.0997572406,((pCRESS6|WP_044671103.1:0.1258897902,pCRESS6|WP_000746010.1:0.1650015447)89.6/100:0.0374517077,(pCRESS6|WP_003048523.1:0.0575544917,pCRESS6|WP_039694464.1:0.1182074519)97.7/100:0.0575655322)100/100:0.1811638081)99.4/100:0.1810304094)82.6/98:0.1052264922,((pCRESS6|WP_018030886.1:0.1797087340,pCRESS6|WP_003104234.1:0.1370024468)98.8/100:0.1388661394,pCRESS6|WP_039670385.1:0.2528874422)100/100:0.3134523316)90.3/100:0.0868273550,((pCRESS6|WP_019299400.1:0.1545835019,pCRESS6|BAM66968.1:0.1528304505)100/100:0.4000348388,(pCRESS6|WP_032941943.1:0.0398540300,pCRESS6|WP_058223604.1:0.0339354972)100/100:0.4791278028)96.2/100:0.1268084118)99.7/100:0.3778226274)44.7/92:0.1674434975,(pCRESS6|WP_061343647.1:0.5401126378,pCRESS6|WP_017371219.1:0.5081858720)100/100:0.5363645324)100/100:0.7269570494,(((((pCRESS7|CDE19587.1:1.0776058177,pCRESS7|WP_028509833.1:0.9800128512)93.5/77:0.3069676356,((((pCRESS7|CCZ68460.1:0.5373566910,pCRESS7|SCG87263.1:0.7210673081)100/100:0.4341989840,((((((pCRESS7|YP_006961027.1:0.0462977464,pCRESS7|YP_003617079.1:0.0595193127)100/100:0.1439699510,pCRESS7|ABC65794.1:0.2121670012)92/97:0.0774690450,pCRESS7|ABC65805.1:0.0945604932)87.2/97:0.0604925636,pCRESS7|WP_011161011.1:0.0526484852)98.9/97:0.1327836316,pCRESS7|YP_001966814.1:0.3346261002)91/100:0.1390142153,pCRESS7|KXT29039.1:0.5199008032)99.7/100:0.3080386071)92.6/100:0.1745347014,pCRESS7|ODR34583.1:0.5660764782)80.9/99:0.1428317803,pCRESS7|WP_019282500.1:0.7762024696)79.4/70:0.1228338436)57/71:0.0861304442,(pCRESS7|CCZ93342.1:0.4074295779,pCRESS7|CCY61699.1:0.4270868128)99.2/100:0.2148056195)95.1/99:0.2092458978,(pCRESS7|WP_002578150.1:0.9342723369,pCRESS7|CUN62864.1:0.7837600015)94.4/100:0.2930819657)100/100:0.6912416858,(((((((((((((pCRESS8|WP_033683822.1:0.0155374513,pCRESS8|EFO53527.1:0.0243604928)99.2/100:0.0920646419,pCRESS8|WP_049523992.1:0.1648163611)100/100:0.2389206073,pCRESS8|WP_024410839.1:0.0679965465)83.1/98:0.1324690455,(pCRESS8|CYX46115.1:0.1390685224,pCRESS8|CYW87437.1:0.0592372060)91.2/100:0.0936252269)100/100:0.4431884294,(pCRESS8|WP_051448806.1:0.2323321171,(pCRESS8|WP_050444210.1:0.0888627344,pCRESS8|WP_010817837.1:0.0561370181)99.7/100:0.2384128907)100/100:0.4280600078)95.7/100:0.1859393558,(((pCRESS8|WP_042900192.1:0.0032036304,pCRESS8|WP_050492321.1:0.0026438633)0/100:0.0001702672,pCRESS8|KXA58447.1:0.0027689342)56.9/97:0.0055581935,pCRESS8|WP_000093566.1:0.0000023844)100/100:0.8816770126)18.3/81:0.0744985055,(pCRESS8|WP_016226904.1:0.4414021692,pCRESS8|SCH55298.1:0.6677336422)99.6/100:0.3418085672)84.8/96:0.0835023461,pCRESS8|ABP89830.1:1.2296484414)56.6/96:0.0432063918,(pCRESS8|EEJ43069.1:0.6203394388,pCRESS8|WP_004900270.1:0.7782601421)99.9/100:0.3554294155)96.5/92:0.1304233214,((((((pCRESS8|WP_008469878.1:0.3476693540,pCRESS8|WP_013641481.1:0.6222903253)99.4/100:0.2774811592,((pCRESS8|WP_046324376.1:0.3491431708,(pCRESS8|WP_008472153.1:0.1344054857,pCRESS8|WP_013641468.1:0.1720293315)100/100:0.6818361406)67.9/95:0.0599791738,pCRESS8|WP_049150683.1:0.3971451115)96.7/100:0.1318027040)93.4/99:0.1155321889,(pCRESS8|WP_014567781.1:0.4472175015,(pCRESS8|WP_007125042.1:0.1681477842,pCRESS8|WP_060461663.1:0.1889463986)98/100:0.1278983979)99.2/100:0.1892092209)82.4/98:0.0826893782,(pCRESS8|WP_011254167.1:0.5077763193,pCRESS8|WP_056985318.1:0.6840704437)97.2/99:0.2176764672)20.8/84:0.0869206734,(((((pCRESS8|WP_003549058.1:0.0668582309,pCRESS8|KRN00682.1:0.0560555978)100/100:0.1265276201,pCRESS8|CDA26462.1:0.1230877613)88.3/100:0.0650480542,(pCRESS8|CDI43023.1:0.1188677145,pCRESS8|KRK41125.1:0.2372719063)51.2/88:0.0553516355)95.2/83:0.1077029539,pCRESS8|CDI42894.1:0.2827390792)64.7/83:0.0774388535,pCRESS8|WP_012845653.1:0.1962055169)100/100:0.4804825558)100/100:0.4475929516,(pCRESS8|WP_016356676.1:0.1894892622,pCRESS8|WP_016622553.1:0.1482838934)100/100:0.9048147196)75.6/37:0.1000297404)46.7/41:0.0844827376,((((pCRESS8|WP_057906729.1:0.0694929893,pCRESS8|WP_057827851.1:0.1771054425)100/100:0.6508899291,pCRESS8|WP_057827085.1:0.6607897603)94.3/100:0.2238083264,pCRESS8|WP_002821392.1:1.1406582891)92.4/100:0.2141441770,(((pCRESS8|CUR41281.1:0.9905365279,((pCRESS8|KRN07545.1:0.8059223902,pCRESS8|WP_046923918.1:0.8910212202)37.7/37:0.0855496380,(pCRESS8|WP_003665528.1:0.7467255953,pCRESS8|WP_006499656.1:0.8302629097)73.7/96:0.1141659882)34/39:0.0785055998)95.2/95:0.1609861501,(pCRESS8|WP_046025501.1:0.3912390895,pCRESS8|WP_034540695.1:0.2777602298)100/100:0.8344790189)5/93:0.0663383472,pCRESS8|AKG47101.1:1.1952303287)89.5/99:0.1492349829)88.3/75:0.0898510196)4.2/22:0.0526425443,pCRESS8|YP_006939186.1:1.5009263804)84.6/100:0.1396936044,pCRESS8|WP_062359070.1:0.9621659192)98.4/100:0.2607832186)76.2/90:0.0623205925)16.7/79:0.0806591307,(((pCRESS4|WP_000818357.1:0.6027591079,pCRESS4|WP_000186194.1:0.5706188309)99.3/100:0.3271444352,((((((pCRESS4|CBL40434.1:0.6871354828,pCRESS4|CRY93789.1:0.4055314958)69.3/92:0.0897933674,pCRESS4|WP_021639163.1:0.5828300148)87.3/99:0.1048306419,(pCRESS4|WP_007889993.1:0.8324920036,pCRESS4|CDA18875.1:0.9211391047)67.6/94:0.1451997304)93.2/94:0.0999843120,pCRESS4|CRY97508.1:0.8427209169)98.4/100:0.2115587516,(pCRESS4|WP_044572803.1:0.7763649099,((((pCRESS4|WP_067940518.1:0.0530804565,pCRESS4|WP_005464724.1:0.0065384537)93.1/100:0.0373656052,pCRESS4|WP_043534193.1:0.1283907328)98.2/100:0.1844036184,pCRESS4|GAC78794.1:0.5419533359)99.8/100:0.5211794363,(pCRESS4|WP_006681830.1:0.0016775462,pCRESS4|WP_052038917.1:0.0040624046)100/100:0.4899986956)97.1/100:0.2088674881)96.2/100:0.1497366633)37.7/51:0.1010273888,(pCRESS4|WP_017824301.1:0.2713652078,pCRESS4|CEI31812.1:0.3433167187)100/100:0.4664926089)85.7/54:0.1355331667)100/100:1.2201240557,((((((pCRESS5|WP_024390948.1:0.2574605916,pCRESS5|WP_029176301.1:0.2525207904)100/100:0.6591551485,(pCRESS5|WP_024393234.1:0.1822199383,pCRESS5|WP_050238550.1:0.1841375986)100/100:0.6358440920)49.9/98:0.2140135050,pCRESS5|WP_061866456.1:0.8496574259)100/100:0.5550539660,((((((pCRESS5|WP_061417941.1:0.0124551637,pCRESS5|WP_061863770.1:0.0323754429)34.5/99:0.0172853431,pCRESS5|WP_049478725.1:0.0608054833)42.3/97:0.0228341224,pCRESS5|WP_049535277.1:0.0209033834)100/100:0.1634447809,pCRESS5|WP_067193806.1:0.1396133927)74.9/100:0.0386319386,(pCRESS5|WP_044771983.1:0.1163409372,(((pCRESS5|WP_024408358.1:0.0554496996,(pCRESS5|WP_033583888.1:0.0051450265,pCRESS5|WP_049481849.1:0.0051940772)100/100:0.1452608802)93.8/97:0.0420452576,pCRESS5|WP_039694423.1:0.0575012063)2.5/57:0.0249724406,pCRESS5|WP_029171254.1:0.1109670464)85.2/100:0.0359367932)99.6/100:0.1175714403)99.1/100:0.2372378018,((pCRESS5|WP_053863690.1:0.0186987146,(pCRESS5|WP_018166163.1:0.0363520984,((pCRESS5|WP_024382134.1:0.0025358873,pCRESS5|WP_024389873.1:0.0137368745)91.6/100:0.0094299856,pCRESS5|WP_024399566.1:0.0453269717)11.2/95:0.0082736399)99/98:0.0307959019)9.8/85:0.0016422621,pCRESS5|WP_014735272.1:0.0098072106)100/100:0.4325341968)100/100:0.7304361001)50.5/92:0.1326233937,((pCRESS5|WP_058211405.1:0.4647655893,pCRESS5|WP_017368666.1:0.2793557725)100/100:0.7866336472,(pCRESS5|WP_038978316.1:0.5995483253,pCRESS5|WP_046467524.1:0.6118159890)99.9/100:0.6001536940)0/10:0.0883246784)47.1/81:0.2266098310,(pCRESS5|UniRef50_W1I5Y6:0.8615955032,pCRESS5|UniRef50_R5VXD3:1.5477997637)OROOT:0.8302541015)97.6/100:0.5149130560)98.2/100:0.1786166954)31.9/63:0.3470679192)93/87:0.5941455445);
