# Supplementary material for: Multiple origins of prokaryotic and eukaryotic single-stranded DNA viruses from bacterial and archaeal plasmids
Source: Nat Commun. 2019 Jul 31;10:3425. doi: 10.1038/s41467-019-11433-0 (PMC6668415; doi:10.1038/s41467-019-11433-0)
Supplement: Supplementary file 8 — Dataset 7 [file 41467_2019_11433_MOESM8_ESM.docx]

**SUPPLEMENTARY DATA 7**

# PHYLOGENETIC TREE SHOWN IN FIGURE S5B

(((pE194_pMV158-like|UniRef50_A0A1Y4G0S9:0.69643053302785951075,(((pE194_pMV158-like|UniRef50_A0A0H5QIL6:0.52972902977799551927,pE194_pMV158-like|UniRef50_A0A0H5PZG0:0.60325954169323858789):0.38076126831382511861[89],pE194_pMV158-like|UniRef50_A0A158LH93:0.60667984996529888964):0.10657775678602120217[8],(((pE194_pMV158-like|UniRef50_A0A0E9F4G4:0.60606688461172608662,pE194_pMV158-like|UniRef50_G8CNT2:0.29349101474187622163):0.41608711541255233302[98],(pE194_pMV158-like|UniRef50_A0A1C6AUS2:0.37370786870851968597,pE194_pMV158-like|UniRef50_A0A0E9DRD0:0.38152236443691428525):0.16371327589503489874[87]):0.07860712073765151187[31],((pE194_pMV158-like|UniRef50_A0A0H5PV05:0.63482435259949265838,pE194_pMV158-like|UniRef50_A0A0H5PZW4:0.62781329667425789243):0.13804852337765330383[52],(((pE194_pMV158-like|UniRef50_A0A087EKU7:0.17376778857342842222,pE194_pMV158-like|UniRef50_A0A0S2MGE2:0.25337910199338142814):0.08332625075251670621[41],pE194_pMV158-like|UniRef50_D3R6U9:0.15353113018806466283):0.81297516414005099872[100],pE194_pMV158-like|UniRef50_F0HMF3:0.74224968307320293714):0.23588871956810048003[26]):0.05337168800163715615[4]):0.09252356661297622686[2]):0.07697376461663914260[4]):0.05417640612396058819[12],(((pE194_pMV158-like|UniRef50_G8CNR9:0.52631271925971123693,pE194_pMV158-like|UniRef50_A0A0H5PZI7:0.61319050087833315565):0.20289806531067230977[68],((pE194_pMV158-like|UniRef50_UPI000300949E:0.70564103058762139842,(pE194_pMV158-like|UniRef50_A0A0H5PZA0:0.73668057448887114269,pE194_pMV158-like|UniRef50_A0A1W6BZG0:0.56575671510267522279):0.08830307230242762095[15]):0.11493220129187532719[6],(((pE194_pMV158-like|UniRef50_S6F6F1:0.96515245442394947961,pE194_pMV158-like|UniRef50_W7D2V3:0.39761814829618069567):0.20529322504734698329[39],pE194_pMV158-like|UniRef50_A0A0R3QHC2:0.46826061849004585458):0.14846238013089729790[16],((pE194_pMV158-like|UniRef50_Q48831:1.04121381852353289688,pE194_pMV158-like|UniRef50_A0A0R1P770:0.91867844724315317340):0.14419084530162221136[24],(pE194_pMV158-like|UniRef50_A0A1B1IHL4:1.09960024613440388030,(pE194_pMV158-like|UniRef50_A0A0Z8IYX5:0.87320906945099674434,pE194_pMV158-like|UniRef50_W1I557:0.37873901906531348693):0.18009875329631230834[45]):0.00000093905322139818[2]):0.14363305872394446783[3]):0.05840435362540689207[0]):0.05447494298584130284[0]):0.09616506390407879301[1],((((((pE194_pMV158-like|3DKX_A:0.14020205504842975230,pE194_pMV158-like|UniRef50_A8W662:0.35378074920315055740):0.20269108034448657385[87],pE194_pMV158-like|UniRef50_A0A1Y4QQC0:0.33788662313392592296):0.58005955848287815702[100],(pE194_pMV158-like|UniRef50_W1I697:0.68296562236106761468,(pE194_pMV158-like|UniRef50_A0A0E9F9L7:0.44140141484674239036,pE194_pMV158-like|UniRef50_A0A174GG61:0.78379343556667802062):0.21262438426151505921[74]):0.53508438895363763521[99]):0.33068932669856915263[66],pE194_pMV158-like|UniRef50_S6CES9:1.42380070876049069639):0.06557102695858772756[10],(pE194_pMV158-like|UniRef50_O31070:0.38113324658584696314,pE194_pMV158-like|UniRef50_K7YFJ8:0.67203555538830817007):0.33875224125181868651[97]):0.09321242844855755216[2],(((pE194_pMV158-like|UniRef50_A0A0H5Q8X5:0.87765492042556159102,(pE194_pMV158-like|UniRef50_U2TJ01:0.69652491763179080753,pE194_pMV158-like|UniRef50_A0A1Y3UDM3:0.72406190057993591314):0.28182376238176171102[53]):0.22601895627265999877[12],(((pE194_pMV158-like|UniRef50_K9RYD5:0.93636015741773126475,pE194_pMV158-like|UniRef50_K9RZV9:0.40951051905575092116):0.19022834692058340766[70],(pE194_pMV158-like|UniRef50_A0A0H5Q0X0:0.48180868306759960795,pE194_pMV158-like|UniRef50_A0A0E9EV38:0.37058833812229824423):0.13889642193154044802[75]):0.05289893023929249927[49],pE194_pMV158-like|UniRef50_U2EU97:0.31155962231185507783):0.16306893412525863551[58]):0.08865778562547300956[3],(pE194_pMV158-like|UniRef50_U2QZX4:0.75667730084871120955,pE194_pMV158-like|UniRef50_UPI000481AAFB:1.02857698679772102679):0.20313239534481941639[33]):0.14513902900256006601[2]):0.19831030372604052658[6]):0.12293407195943242827[16]):0.446802726400241[99],((((((((CRESSV2|FJ959082:0.60632715590735430933,(CRESSV2|KT732819:0.64378035764572416610,(CRESSV2|KP153447:0.49182740704686511712,(((CRESSV2|KT732823:0.98358864707403514949,CRESSV2|KM598396:0.39275955346971780369):0.24472665422400258217[49],CRESSV2|JX185415:0.38137090190202266315):0.46769765507790966863[98],(((CRESSV2|KP153404:0.51988874808826635032,CRESSV2|KC248416:0.30824862815021164542):0.11690659091097106714[66],CRESSV2|KT149398:0.63586931703755189549):0.07135139143812241347[65],(CRESSV2|KP153485:0.24389331290663066043,(CRESSV2|KJ547648:0.15861560717714581759,CRESSV2|KT149412:0.29735998026507404202):0.08607952920023123622[54]):0.45721872359910092909[100]):0.11665297441028227299[72]):0.04761976241937219967[22]):0.06978609355006243353[16]):0.08900053791111738055[11]):0.06783877226889210854[6],(((CRESSV2|KU043406:0.42359987963502565567,CRESSV2|KU043397:0.46558303825988950342):0.15978649548396114954[90],(CRESSV2|KM573776:0.26276894866958327990,CRESSV2|KM573767:0.30965869129398215787):0.15358279321276691043[91]):0.33823677695773285423[100],((CRESSV2|KP153377:0.56091230412912662651,CRESSV2|KF738877:0.81300176864063822268):0.20995514523980082777[79],(CRESSV2|JX904107:0.48715504655912927889,CRESSV2|JX904562:0.30210754124506145057):0.29533894071129823677[100]):0.12271602804568830514[33]):0.05868134152287229205[11]):0.03619602870869254724[4],((((CRESSV2|KM821764:0.69656868445622910357,(CRESSV2|KP153369:0.75163879920172771421,CRESSV2|KP153468:0.67570696815469999397):0.06613229241906704026[17]):0.08585739607549322527[13],CRESSV2|KP153483:0.82722035572795471481):0.14428861081497720842[21],((((CRESSV2|JF755415:0.20264370432266565203,CRESSV2|KT732816:0.28279856002050274277):0.29053875725128203156[100],CRESSV2|JX904344:0.39064169814826688043):0.05460510292967708096[26],((CRESSV2|JX904185:0.33682794788047515366,CRESSV2|KP153364:0.41962014307586520401):0.20380221447556381986[97],CRESSV2|JX904420:0.50607561074544071822):0.11386713933198978899[39]):0.04263733719846930781[17],CRESSV2|KM821755:0.63967845914506249461):0.05938696183215701890[25]):0.05846411627032987307[6],(CRESSV2|KP153360:0.30045420681535672802,CRESSV2|KT149394:0.40637375653101182049):0.51209507152885558323[100]):0.06041680647043334851[6]):0.50595701900197243006[100],((((CRESSV1|KU043424:0.55052725919318634684,((CRESSV1|KU043411:0.27552505510380037590,CRESSV1|KJ206566:0.40532466140232142893):0.29118433022024420298[99],((CRESSV1|KT862256:0.26168601680514247398,CRESSV1|KF246569:0.21303977292766676510):0.62265124876441679813[100],CRESSV1|KM573766:0.66064128623760576264):0.12052609464176688991[64]):0.09951881080284483150[45]):0.66387971768397480155[100],((CRESSV1|KF133822:0.60713421160062241988,CRESSV1|KM874309:0.67052032402077332840):0.21386265068535215139[84],((CRESSV1|FJ959078:0.71935143099530796107,(CRESSV1|KM874347:0.69792418597161165472,CRESSV1|KT149404:0.83978552844730436355):0.08974875299493910441[40]):0.12167875644189120321[23],(CRESSV1|KP153497:0.94342594518658440261,(CRESSV1|KX388513.1:0.07165751082481459922,CRESSV1|KX388515.1:0.00000093905322139818):1.29827807607621181774[100]):0.14628533449010114320[18]):0.12210095079374608251[24]):0.12806847954293018588[21]):0.14230112888333770749[77],(Circo|KT732825:0.92597835672581441102,((Circo|AIF76251.1:0.73649935850284298233,(((Circo|AMH87650.1:0.06288708326130811044,Circo|AMH87652.1:0.20708924053761021411):0.66818197394496614550[100],((Circo|YP_009047065.1:0.11339774882032629955,(Circo|YP_008130363.1:0.16265513636759990068,(Circo|ADU76993.1:0.22608419323951647883,Circo|ADD62477.1:0.10684401571753855686):0.06067791608248002122[39]):0.06680709956536845517[40]):0.33534947250756419468[100],((Circo|AIF76254.1:0.32085426015948020906,(((Circo|ADD62453.1:0.23345115389770559888,Circo|AIF76249.1:0.26081970363379819444):0.08144914200166709917[51],((Circo|YP_004152331.1:0.20925817278977873470,(Circo|ADD62471.1:0.15540893858490087420,Circo|AIF76252.1:0.10514177565007236381):0.07463281421307443875[96]):0.02885748941085290731[42],(Circo|YP_009021843.1:0.30286272703687966423,((Circo|AEL87786.1:0.20504132011955833459,Circo|ADI48251.1:0.19036420797250019898):0.06920968067709887261[76],Circo|AEL87790.1:0.16662140940997405281):0.05952377873021518634[40]):0.02170751147039477399[5]):0.04250094973232401707[7]):0.04163133969104265003[25],(((Circo|AGJ74760.1:0.25201186803099412392,((Circo|ADY17982.1:0.26429372031343534299,Circo|AGJ74756.1:0.24283283629521723590):0.06263703799655276350[18],Circo|YP_009110680.1:0.14537482068515672484):0.04407737478172178702[15]):0.02759382553395278367[2],(Circo|AKE49355.1:0.21021306625047403438,((Circo|AGJ74758.1:0.20965369267715905366,Circo|ADU77011.1:0.15989522926842683614):0.05510375422460626199[53],((Circo|ADD62455.1:0.15818685347243147121,Circo|ADD62451.1:0.13489801923400301931):0.04997057966853341027[71],Circo|ADD62461.1:0.24146021832976924393):0.02705615165610139247[12]):0.04699767164100434463[51]):0.06968494750093491563[37]):0.02323552992994918839[0],(Circo|ADD62457.1:0.21833811738135150415,(Circo|AEL87792.1:0.20515893306202734325,(Circo|ADD62473.1:0.09613120656458339075,Circo|AIF76266.1:0.21327690036157884168):0.10012050244586012671[99]):0.09036452696713238864[91]):0.04706317007574818689[27]):0.04920578355379529728[3]):0.06418231421864271924[23]):0.02629777740037775313[42],(Circo|YP_009021870.1:0.25563715854304930319,Circo|AFS65290.1:0.25784900072641797619):0.10135881168616241166[78]):0.16975395849990621011[97]):0.11806052103176296886[89]):0.05425254384986226419[29],(Circo|YP_009237526.1:0.53712107617991511344,Circo|YP_009116910.1:0.59584038461261656927):0.11480281134549694122[43]):0.05287431389930467157[25]):0.13821377871188336717[46],((((((Circo|AGL09969.1:0.12965090965761236208,Circo|YP_009021891.1:0.11789975644508493557):0.28783235924611261236[100],(Circo|AKO84203.1:0.40437476143973477560,((Circo|AIF76280.1:0.01240838661475043500,Circo|KJ641742:0.00633921268934817175):0.23798557511324883174[100],(Circo|YP_007974237.1:0.18353147840548822423,Circo|AAZ78351.1:0.22352686093103496523):0.09181791587590491799[93]):0.34725339258614407179[100]):0.11414060210681632734[68]):0.10204225261155243365[49],(Circo|AIF76261.1:0.27581188081106677856,((Circo|AIF76265.1:0.13830329520791775910,Circo|AIF76253.1:0.16628991708539264671):0.06569560109583034990[66],Circo|AIF76248.1:0.13463748087860738512):0.13113597439667201705[91]):0.33430728779599572231[100]):0.03811583224833144795[19],(Circo|YP_009170674.1:0.49651136305971871332,((Circo|ABU48445.1:0.12444286978187370230,Circo|AHK80894.1:0.13303151035179905382):0.43503231090832228389[100],(((Circo|YP_009134739.1:0.11393059463247776075,(((Circo|ADU77009.1:0.19171478135047781421,Circo|YP_764455.1:0.11829916937970384738):0.02958118272649155195[25],Circo|KU230452:0.08447771666379851052):0.03820675784773053946[30],Circo|NP_573442.1:0.17214421598256532397):0.04172344827797971195[33]):0.13371964505236619281[89],(Circo|AEL28794.1:0.33139593814409940009,Circo|AFL02442.1:0.35442793767088387469):0.06422556437500090420[38]):0.04596394696139193686[36],Circo|YP_803546.1:0.29580982395310517763):0.19937916080343737191[97]):0.09366912118705378321[52]):0.11249336570779344679[46]):0.10435192226598385967[40],((Circo|YP_004376332.1:0.46110530625926793302,Circo|ADD62475.1:0.41043365363572381632):0.10898310636644337190[73],Circo|YP_009091696.1:0.40957296675319909696):0.06687724547916860940[30]):0.08830484093344054264[38],Circo|YP_009000900.1:0.50233389393960681257):0.10221120198602656748[49]):0.09067968835019647733[53]):0.21675947975293657910[96]):0.11431220013413762038[78],((CRESSV3|KJ641729:0.76159733070475421624,(((CRESSV3|HM228875:0.12141748316195764834,CRESSV3|KJ641722:0.15150706298456642629):0.52121240114410005706[100],((CRESSV3|KF738883:0.65231436235643147636,CRESSV3|KM972726:0.53679686711799801113):0.09830444114075369277[29],(CRESSV3|JN857329:0.37702831327218744306,CRESSV3|KJ641718:0.38948897287256356581):0.16074764044588360501[71]):0.04143892746070797911[17]):0.10499297473987490026[47],(CRESSV3|KM598404:0.71011514290409394334,CRESSV3|JX185418:0.49808873689171717647):0.18722359196034288331[78]):0.01882900727118276898[22]):0.10197600005219653596[82],(CRESSV3|KT149409:0.62974655902004628150,((CRESSV3|KT149403:0.78614650490718740450,((((CRESSV3|JX904581:0.48740484954099994042,CRESSV3|KM874304:0.37243023931260421655):0.05708583332812478678[22],CRESSV3|KM874317:0.45172340492454576832):0.05612091921597341104[26],(CRESSV3|KP153408:0.32867884575827616134,CRESSV3|KP153422:0.45727928330172862292):0.05704832984802898216[15]):0.09214931047350585769[9],(CRESSV3|KM874300:0.73160840821088435337,((CRESSV3|JX904139:0.26452146938742188143,(CRESSV3|JX904076:0.26362186695183870144,CRESSV3|JX904075:0.33383351093959662270):0.05922591768307864518[56]):0.14697481536081943854[80],CRESSV3|JX904407:0.82398009180333608992):0.10853273471082870716[52]):0.04993011177696947411[13]):0.10650087203176615558[44]):0.03373104597314777880[37],CRESSV3|KM598406:0.52629388487837414079):0.05784670993010544715[22]):0.07638315830951131202[57]):0.16142648101200915423[97]):0.05111396565956340121[47]):0.13130333265883398219[48],(((NanoAlpha|AIF34798.1:0.57625322334104089883,((((((NanoAlpha|YP_008169853.1:0.08608363825838229177,NanoAlpha|HM163578:0.06640664212751117013):0.03655389291322997530[47],(NanoAlpha|YP_009246456.1:0.12561803178446473428,NanoAlpha|ALK03646.1:0.14947429331475189929):0.05081129441680517361[92]):0.70588923955722049364[100],((NanoAlpha|AAA51426.1:0.22502285905592706028,(NanoAlpha|AAA51422.1:0.04331092089605278334,NanoAlpha|ACB86656.1:0.27780468336616520597):0.25043961853826046671[100]):0.20880878449792400486[97],((NanoAlpha|KC978991:0.04375023963455596659,NanoAlpha|YP_009058890.1:0.05839356067519620164):0.30380171707917269508[100],NanoAlpha|NP_619760.1:0.11713347042504743123):0.25508656207035196317[84]):0.07702036545555572544[39]):0.12236664489277303269[38],(NanoAlpha|U16735:0.16860204739119546979,NanoAlpha|KC979052:0.17053724203777739787):0.27994589107859940702[100]):0.07784815754444321390[38],(NanoAlpha|NP_619759.1:0.37286765592506682099,(NanoAlpha|JX458742:0.35032198410673559552,NanoAlpha|KF471057:0.46734498945656627322):0.30166003995222279155[99]):0.10287902147712582601[54]):0.31986169370015654634[98],((NanoAlpha|HE654123:0.05873860309059900087,NanoAlpha|YP_003104737.1:0.02723305015012489338):0.30455510393137597003[100],(NanoAlpha|JF957636:0.01480679297542085091,NanoAlpha|AKO71308.1:0.00000093905322139818):0.38719890108307414289[100]):0.42890912973387507190[100]):0.29167447527903317406[97]):0.31565091067197531549[99],(CRESSV4|KX388505.1:0.73525064186027278001,(CRESSV4|YP_009163936.1:0.48977293974052943382,(CRESSV4|AHH31482.1:0.36012489664053221050,(CRESSV4|YP_009237559.1:0.67167009469358396334,CRESSV4|YP_009021888.1:0.53555282486453803514):0.27229330279270830273[97]):0.10726903565388452855[58]):0.23521598079416133897[92]):0.17336588250527243393[95]):0.12165124419104195175[64],(((((CRESSV5|KR528554:0.32264901526570338719,CRESSV5|KR528556:0.29253143152498101154):0.14322917319717998197[85],(CRESSV5|KR528551:0.44283191069833416353,CRESSV5|KR528562:0.45666766882223885915):0.18443867138617672796[93]):0.12144461365960752819[61],(((CRESSV5|KR528547:0.63359721131338930800,CRESSV5|KR528561:0.44590720304181724609):0.05165447844416599238[53],CRESSV5|KJ641738:0.58292053860196046955):0.07705049300759911346[26],(CRESSV5|KM874354:0.50544164660393064992,(CRESSV5|JX904231:0.52118034105524690958,((CRESSV5|KR528553:0.60885309301185108755,CRESSV5|KR528545:0.42962265707540719939):0.07691703183143777656[49],CRESSV5|KT945163:0.63929812527348894857):0.08310197210863305706[50]):0.11698127000573455003[38]):0.09267952156939963937[24]):0.05643916897432874752[12]):0.20582426644213205891[85],(CRESSV5|KJ547646:0.70858674886064443310,(CRESSV5|KP153451:0.59620086817800110879,CRESSV5|KJ547650:0.54898782660988931781):0.15122542142602093951[75]):0.18054641612647676086[90]):0.08901304134134520318[61],(((Smaco|KM598409:0.54264826705527569040,(Smaco|AEW47007.1:0.24971935216078436359,Smaco|YP_009252314.1:0.34087759928638289297):0.26984862629972189429[85]):0.84132466090747770604[100],((((Smaco|KT862221:0.13701649500786175495,(Smaco|KT862218:0.03305942404357269798,Smaco|AIY31250.1:0.26351498358509806152):0.14117368630177454247[97]):0.20248293156317062613[98],(Smaco|KM573771:0.24727943018582324020,Smaco|KM573775:0.20485297685954056979):0.22426694291358961753[99]):0.19173217679231113753[92],(((Smaco|KU043420:0.58790810141568794478,(Smaco|KP233189:0.20148925286711688587,(((Smaco|KJ577810:0.01196707483383127948,Smaco|YP_009054985.1:0.12060270058435172691):0.02601553009368402086[69],Smaco|YP_009030025.1:0.02640090003476829039):0.08304719857486622669[97],(Smaco|YP_009022025.1:0.17719829474370257794,((Smaco|AMR73073.1:0.05031262773617400930,Smaco|KX838317:0.11666642848051823889):0.07532146359120282797[87],Smaco|KX838318:0.05059470873150619802):0.17803958842901382309[98]):0.04149633309303481232[21]):0.06848607952020914491[56]):0.10371941674778589748[90]):0.16342688139235278522[28],(Smaco|YP_009252320.1:0.51298267050750001594,(((Smaco|KU058671:0.32487054520583291684,Smaco|KU043430:0.18268791269101017360):0.25418330399906013950[99],((Smaco|YP_009118278.1:0.75307369481335306727,(Smaco|YP_009163761.1:0.29775595795547055156,Smaco|YP_009054993.1:0.18602323184023764058):0.11435357481988162043[83]):0.06138311805416282046[19],(((Smaco|ADB24799.1:0.00000093905322139818,Smaco|GQ351275:0.04197128780584861996):0.39012260862822673024[100],Smaco|KU043403:0.33423390096777289004):0.12716396135668939227[43],Smaco|AMR73071.1:0.30308312233847606310):0.12513315844510830566[36]):0.16625163031281586790[94]):0.07066195457451512896[17],(Smaco|KU043422:0.63590697212766511548,(Smaco|AIY31246.1:0.52482606137049703232,((Smaco|KJ577813:0.00454736565280866502,Smaco|YP_009054987.1:0.00000093905322139818):0.07528541592630129398[96],(Smaco|KU043428:0.07884595683447601089,Smaco|YP_009118276.1:0.10800035091203886994):0.08374869633871373509[96]):0.21789678146329316544[100]):0.08181713419319525116[23]):0.02965812601991308153[3]):0.08610826920852208965[9]):0.08538714201904368251[8]):0.11746455395693544688[43],(Smaco|YP_009252308.1:0.79588587957468304168,Smaco|KY086298:0.36826183098302583963):0.06366875986969619539[21]):0.18554096730176314334[70]):0.14871343500470388110[22],Smaco|YP_009252310.1:0.59948166173857087013):0.28010179063398976673[45]):0.48368771925980014181[93],((Smaco|AIY31256.1:0.31281582431609950001,(Smaco|KT862224:0.34115004456001979394,Smaco|AIY31243.1:0.15093033713031639431):0.13392757333557306354[75]):0.64047119929297435803[100],(((((Smaco|KU203352:0.19608428467216815205,Smaco|KJ547633:0.15379587680458664534):1.12680764938409394560[100],Smaco|YP_009252316.1:0.52773021627179639292):0.13184429223374266593[59],((Smaco|AJE25851.1:0.14711241907222546987,Smaco|AJE25845.1:0.06752691963957282995):0.13182080328437137395[86],((((Smaco|KP233175:0.00000093905322139818,Smaco|AJE25847.1:0.00935287247786132162):0.17566228095829120037[81],Smaco|AJF23060.1:0.00887910786383620462):0.07952910550280017921[58],Smaco|AJF23062.1:0.06287964630849765535):0.14064959926874967544[64],Smaco|KY086301:0.09125439373283600331):0.07466394243450352741[52]):0.48150070912674902690[99]):0.06078034778020011131[27],Smaco|YP_009252326.1:0.55312765930882790144):0.22700892843680711697[67],Smaco|AJD07511.1:0.69889495199706741513):0.28728071622104445204[88]):0.44807045973398146321[93]):0.73299773165067205749[100]):0.05419606851867291714[50]):0.49389924384873512553[100]):0.14056674348404807340[80],((((pCRESS1|CVH76026.1:1.30591336578944949842,((pCRESS1|WP_003102166.1:0.06467159718365934895,(pCRESS1|WP_029176105.1:0.13356994604586133746,pCRESS1|WP_000032131.1:0.12368967737014895103):0.04145989564813142014[54]):0.22692895784190939001[100],(pCRESS1|WP_029694263.1:0.29869672378662220913,(pCRESS1|WP_062004798.1:0.15803161905497808970,(pCRESS1|WP_003030931.1:0.06861244114132129812,(pCRESS1|WP_047207334.1:0.10385009197504059542,pCRESS1|WP_029690610.1:0.15718839024110178859):0.03129159454230560217[70]):0.16813866420461595630[100]):0.07961032445787682987[75]):0.13838618772037533655[87]):0.33990835171967104245[100]):0.11145438055167328217[37],(pCRESS1|WP_053982727.1:0.76152282014774796970,((pCRESS1|CUO57637.1:0.34132986850719770899,pCRESS1|CUO23215.1:0.21702585940757163074):0.17231838928051346205[96],pCRESS1|CDF01935.1:0.66352557121384136973):0.09045750416665607196[51]):0.09283644603876541057[61]):0.20157762923557479007[54],(pCRESS1|WP_026524352.1:0.06659078634884031678,pCRESS1|WP_026669310.1:0.05893414721190944061):0.58160259189917840050[100]):0.50079136201178164889[100],(((((GasCSVlike|YP_007517186.1:0.10854119676315426846,GasCSVlike|YP_009126903.1:0.17771442271735735097):0.95014226717339822503[100],pCRESS2|SCH60086.1:0.49847568203554942778):0.11541240358146173661[32],pCRESS2|WP_036328238.1:0.59523432415393562334):0.07823180738962476344[26],((pCRESS2|WP_020072285.1:0.60553331187964365423,(((((pCRESS2|WP_013978550.1:0.49726759672514492250,(((pCRESS2|WP_021882760.1:0.33269683680438077999,pCRESS2|WP_051600858.1:0.64701307073605462961):0.10638383328777591130[47],pCRESS2|CBL15233.1:0.33724791072673265813):0.11068873990084181214[27],(pCRESS2|WP_009301216.1:0.79055155189893566714,(pCRESS2|CCZ45692.1:0.21359735602442311841,(pCRESS2|WP_021629801.1:0.03226847959002007715,pCRESS2|WP_044942941.1:0.05414457819101359259):0.13106956293088853349[100]):0.14194216706707005193[92]):0.07899537841095366497[66]):0.09517938037023525311[45]):0.23535376894169557360[91],((pCRESS2|EES75484.2:0.77450335808160775120,((pCRESS2|WP_038278663.1:0.14451454575770014688,pCRESS2|WP_024346025.1:0.11297331199022049575):0.21340767097167004374[100],((pCRESS2|CCY69022.1:0.14447792748594118217,pCRESS2|WP_009246639.1:0.21899004712001673978):0.12704270326362951393[96],(pCRESS2|WP_052011064.1:0.28690157872958554908,pCRESS2|CCX75435.1:0.22296634038170970449):0.12714754380424772573[96]):0.06363900780328379803[58]):0.04164179226261307404[31]):0.07703598162001125405[75],(pCRESS2|WP_051639324.1:0.49041936070018438132,(pCRESS2|WP_066550639.1:0.32065344790479943793,(pCRESS2|WP_007865724.1:0.06991011975653656230,pCRESS2|WP_013270924.1:0.07652707418910699444):0.26377629826935122814[100]):0.18345640857030512394[100]):0.05557824582102730698[33]):0.05258782361300568670[69]):0.07403103660321878887[34],((pCRESS2|WP_044928503.1:0.36911302630604547748,(pCRESS2|WP_018597672.1:0.27821857773785846346,((pCRESS2|BAK32345.1:0.37096620709009392458,((pCRESS2|WP_023977019.1:0.18058099129296392715,pCRESS2|KJZ87129.1:0.12188282065663778864):0.13033072363494238588[96],pCRESS2|WP_038350939.1:0.18126245485838834992):0.06534878639512296805[88]):0.06206810071980355809[29],(pCRESS2|SCH17786.1:0.19648583993318374796,(pCRESS2|CDC44519.1:0.27797671486562763876,pCRESS2|CUP05665.1:0.21834888086458406620):0.08073878306796417048[52]):0.14015697253031031422[97]):0.02270641251646482450[8]):0.06725701750069948415[59]):0.02913096894531717537[35],pCRESS2|WP_053167095.1:0.46851160947540809731):0.04597404503972853107[42]):0.03438710858762662748[10],(pCRESS2|CDE72464.1:0.36179196508404976029,pCRESS2|CDB27189.1:0.52408435010124510001):0.05621372670019642026[31]):0.05216827431056838410[6],pCRESS2|WP_037404274.1:0.42857215119726910224):0.05779067622751568600[9]):0.04660435844859465299[26],(pCRESS2|WP_051546484.1:0.67753261586418311868,(pCRESS2|WP_013271491.1:0.18279159472984793378,pCRESS2|WP_066546553.1:0.22796880417202333713):0.63638502908743732611[100]):0.11314256860297269847[50]):0.11601221414427921597[51]):0.11941376853912212541[56],((pCRESS3|WP_033495900.1:1.21742602927526832879,pCRESS3|WP_022856850.1:0.68413508735565609076):0.13798681422407987895[27],((((pCRESS3|NP_613078.1:0.67217368399516552735,pCRESS3|WP_025221073.1:0.79061184408348506025):0.13019515367246464743[39],(pCRESS3|AKO38848.1:0.81351021654846744635,pCRESS3|WP_023022037.1:0.46319350621950561298):0.25106764970633105305[75]):0.14059341877009562394[16],((pCRESS3|WP_052119337.1:0.93226297228965859798,pCRESS3|KFI87454.1:0.60784045087255833550):0.13019103245498894750[26],(pCRESS3|WP_021975256.1:0.37271377136792482876,(pCRESS3|WP_052825216.1:0.25610525026777353208,(pCRESS3|WP_043170238.1:0.14629436992743075074,pCRESS3|KFI81686.1:0.19486699301606000279):0.15380720030495090289[98]):0.12401734446885759322[88]):0.19338712463807114506[97]):0.06777216816665218579[7]):0.10358228744666495014[15],(pCRESS3|WP_055838650.1:0.75269269623536472213,(pCRESS3|WP_016667133.1:0.60632275023059856167,(pCRESS3|WP_002529618.1:0.43732714642491443691,pCRESS3|WP_036342632.1:0.79522477615123143835):0.12955382135882881345[46]):0.25108120033338077182[85]):0.15733926346015120190[49]):0.13818743556446497212[23]):0.39956758747563730916[96]):0.22809403116765347708[84]):0.22434747985211125898[81]):0.32219696132146929957[81],((((((((pCRESS9|YP_006961991.1:0.06590412781602537906,((((pCRESS9|WP_011412950.1:0.03392343801919193991,pCRESS9|CBX25033.1:0.02282477130348796110):0.18268994592620460549[100],(pCRESS9|ABC65385.1:0.21710130511876085668,(pCRESS9|ABC65268.1:0.05989937183767230638,pCRESS9|WP_011412958.1:0.00000093905322139818):0.06050297248781669190[99]):0.01697669251768086154[60]):0.02104408164568994863[64],((pCRESS9|YP_001708790.1:0.07344816351721014658,pCRESS9|YP_007008179.1:0.04567863718921127969):0.02734213828359480236[84],pCRESS9|WP_013747472.1:0.05582955644749795082):0.02729409940367007875[82]):0.03377628346543457005[65],(pCRESS9|YP_001965305.1:0.24105429540542666622,pCRESS9|YP_001965310.1:0.11202338672681667886):0.03869318413635254583[49]):0.03316943477779741439[50]):0.06042097798222542671[67],(pCRESS9|ATL14544.1:0.01587415749844603777,(pCRESS9|YP_001708784.1:0.09065725548022082747,(pCRESS9|WP_015083745.1:0.02982076323763291695,pCRESS9|YP_007008175.1:0.03145735525564914009):0.03642164356620176263[71]):0.02877611363583807216[72]):0.10936227614304570854[97]):0.09121694850102093910[71],(pCRESS9|WP_017193171.1:0.02085686079575265958,pCRESS9|WP_017193695.1:0.05598759170479748054):0.17195698008701479353[100]):0.26794467958666895457[94],(pCRESS9|WP_012662291.1:0.02580997901891243784,((pCRESS9|WP_042068233.1:0.00000093905322139818,pCRESS9|BAD36752.1:0.00449701230362932903):0.02976943839851394630[98],(pCRESS9|WP_011264167.1:0.00680552510734337875,(pCRESS9|WP_015060110.1:0.00224694332479381115,pCRESS9|YP_006959585.1:0.00000093905322139818):0.00000093905322139818[69]):0.01474548699417402818[92]):0.01686788071639762354[58]):0.82638259726656171367[100]):0.14893854861477706653[78],pCRESS9|KXT29032.1:1.14707416615931112602):0.41634855349810157010[82],((Genomo|AUM61807.1:0.79425994286518553089,(((Genomo|KP153522:0.86022383481770570146,(Genomo|KM821748:0.20847786345565050281,Genomo|KJ938716:0.27530753505517535640):0.78428019137287485751[100]):0.12621199043546768581[30],((Genomo|KJ547627:0.50868967718391455701,Genomo|KM598389:0.53290749365948431038):0.34949271067861803486[98],(Genomo|KJ547626:0.71205902364487194678,(Genomo|KJ547634:0.78974386545773400670,(Genomo|YP_009109725.1:0.43519953978240438763,(Genomo|AMH87708.1:0.22977500798612521260,((Genomo|YP_009164036.1:0.48712698833750972538,(Genomo|AMH87693.1:0.24647576829171211066,Genomo|AJD07464.1:0.24083625112556406034):0.37998321904444615216[100]):0.06064267511116423759[13],(((((Genomo|KT862241:0.05201724399426644468,Genomo|YP_009109733.1:0.08291555704299444218):0.22517473776396501606[100],(Genomo|AMH87678.1:0.20792749864015869221,((Genomo|YP_009252359.1:0.14211021545292010226,Genomo|YP_009252365.1:0.18919259210543665217):0.10949071961292095034[99],Genomo|KT253577:0.16460865447180511589):0.05727707338496650785[75]):0.12907297886717858382[98]):0.12920419386880388490[96],(Genomo|YP_009109729.1:0.53645002219996951176,(Genomo|KT598248:0.00000093905322139818,Genomo|YP_009181999.1:0.00901642260925997023):0.37733886771665992033[100]):0.14907953969970699770[59]):0.12007216260402123198[40],((Genomo|AGS12486.1:0.33671380963875596759,((Genomo|YP_003104796.1:0.19658504486754302398,Genomo|YP_009115514.1:0.20804952756297576122):0.13347342109555770939[99],((((Genomo|YP_009252368.1:0.09716157730567032136,Genomo|YP_009021043.1:0.04366479564810102354):0.23694442202957191612[100],Genomo|YP_009252356.1:0.14779299586356811624):0.03802867895444473484[39],(Genomo|YP_009115515.1:0.11301093764874356462,(Genomo|AIF34843.1:0.14144278583394584436,(Genomo|YP_009115519.1:0.07540457616839794930,Genomo|KJ547638:0.06364851233945557907):0.03900451818909723978[47]):0.04164914985882006704[83]):0.12458294553715951181[100]):0.06706363079253190029[67],(Genomo|AMH87666.1:0.31322934565215765357,Genomo|YP_009252353.1:0.15683657536339942751):0.06552108628406011492[57]):0.05082687986851498058[33]):0.02435103780857362718[26]):0.13051223961495614367[93],(Genomo|AMH87733.1:0.35747667915036462905,(Genomo|YP_009252362.1:0.30037023161414821981,Genomo|YP_009109727.1:0.31966623981283298628):0.16367277887087591792[98]):0.06651753795804495051[39]):0.03968424488263201655[28]):0.09824124775042487878[21],Genomo|AMH87702.1:0.48238972567944754521):0.06945342881182324513[19]):0.07326125153744487972[20]):0.17123171981915136231[37]):0.43988545327169392696[100]):0.18204809227270993266[64]):0.10311012124375590338[31]):0.16472110039128262660[39]):0.07251406651031322426[16],Genomo|YP_009351871.1:1.05465986575188375163):0.06812191274638629279[28]):0.20283068771569445765[89],(((Gemini|JX094280:0.15802257181770748562,Gemini|KT214373:0.13389774756103361986):0.43482763076417751957[100],(Gemini|DQ458791:0.38785878777668275630,((((Gemini|AHM88370.1:0.37762903523401813999,((Gemini|AF003952:0.01600050335460579537,Gemini|AAK73446.1:0.00391472615329281886):0.19884587048579602242[100],((Gemini|YP_003288768.1:0.15609653441169893395,(Gemini|P0C647.1:0.27319606692091957800,Gemini|AHM88382.1:0.11196639332003760492):0.03933292005799418622[34]):0.04323490499723641606[42],(Gemini|AHM88378.1:0.15649575431600770692,Gemini|Q80GM6.2:0.16377462469055870109):0.09930611131143669834[97]):0.06654211262618996148[56]):0.14585361865204288234[100]):0.05374148159274699105[54],(Gemini|YP_006273070.1:0.31538096301903184582,Gemini|YP_009026388.1:0.44096097245772597484):0.07559286944960945387[48]):0.11895663944006457102[78],(((Gemini|YP_006666527.1:0.42549975612507812128,Gemini|YP_006666531.1:0.23183171262714433181):0.08553382702816590688[52],(Gemini|AFV91331.1:0.25046054385960220268,Gemini|AIT39773.1:0.42638536998935261391):0.05983333516732786128[35]):0.07956106314645104705[57],((Gemini|AFN80669.1:0.27390863130828629890,Gemini|YP_003915159.1:0.19078218537977992275):0.15925377946759916581[100],(Gemini|YP_006666535.1:0.11555675317428885929,((Gemini|YP_006666523.1:0.15813576071424229053,Gemini|YP_004089627.1:0.11159456374387041744):0.04656602529292468845[93],Gemini|AFN80601.1:0.14252406598427347362):0.06932428654081661257[79]):0.26891987306771358845[100]):0.27581572014831717832[100]):0.07044067263144949964[57]):0.08271655567574209833[60],Gemini|YP_009021763.1:0.41917763066713298326):0.12532203169463584924[87]):0.24907163026813780582[100]):0.21378698447011115769[91],((Gemini|ACO88014.1:0.01729290901688289306,Gemini|FJ665634:0.00388442270421825078):0.62545879562483719383[100],(((((((Gemini|AMW86999.1:0.15821568278921852024,(((Gemini|CBA18089.1:0.09697164831226784409,Gemini|FJ665283:0.06780626846562383403):0.02142836889559222163[68],Gemini|ALV85583.1:0.13090169670210635711):0.03757097082337636923[43],(Gemini|CBH28932.1:0.14568667306151480978,Gemini|AGH29892.1:0.06285983481789669813):0.01315003556875556784[35]):0.00723124611919239053[9]):0.03368296674219171255[42],((((Gemini|YP_009226627.1:0.07910793897788398477,(Gemini|KC108902:0.04220658886060024012,Gemini|YP_003778178.1:0.01028111753479869772):0.16618705101712075156[100]):0.04523508795905967911[85],Gemini|ALR86823.1:0.20485218736593049926):0.13223261209392142557[98],(Gemini|NP_040557.1:0.17173274320560147355,Gemini|ALF37659.1:0.17721567870547280665):0.02742746009820531614[24]):0.10037950028041786599[86],((Gemini|ABG90906.1:0.16019008789441949170,Gemini|ACY79450.1:0.06121546047328775553):0.16222628858924864614[85],(((Gemini|APP87725.1:0.29624634386483050807,(Gemini|AGK24653.1:0.11785439481965775954,Gemini|AAB87607.1:0.07335926587372053331):0.11742717580630841756[100]):0.04210867654588378722[21],((((Gemini|ABD35287.1:0.21169568104517488827,(((Gemini|YP_006905839.1:0.13888905766123091357,((Gemini|AAX39336.1:0.02094138940208114044,Gemini|FM877473:0.02077470487520196793):0.06240411670128855059[100],Gemini|AEY63664.1:0.17502776422925234168):0.04515587210371110882[97]):0.03804121997292073121[58],Gemini|CDW92215.1:0.12717591236898842522):0.02244405081691436613[23],(Gemini|YP_008411025.1:0.23206085479310525899,Gemini|NP_620741.1:0.10901107381936014917):0.02500604794211248871[32]):0.01377152951611209340[6]):0.00842211815842693577[1],((((((Gemini|AGJ03640.1:0.16115868861432519621,Gemini|ACB44970.1:0.05885375105000141549):0.09536073418330927309[56],Gemini|AHA82274.1:0.15209375241777525756):0.03167871390126437980[27],(Gemini|AGV02071.1:0.13779370062539172959,((Gemini|NP_050017.1:0.10413565231558510882,Gemini|AAF75542.1:0.11620686595136793529):0.03301293477121756276[72],Gemini|ACV60535.1:0.09523793050994271148):0.03008572237696028467[83]):0.01387634960026266732[50]):0.02565660646040509793[18],((Gemini|AFH68197.1:0.10887567927596589201,((Gemini|ACI06063.1:0.11282203788113996656,Gemini|AFB81519.1:0.10921736700537720832):0.03274469562476667317[88],Gemini|AFB83419.1:0.12974661944275978076):0.00000093905322139818[51]):0.05740493302356896449[99],Gemini|AFA26437.2:0.05892847396619223244):0.01785284727619128320[30]):0.03714730945124750799[20],(Gemini|AGG08895.1:0.08962984683631070038,Gemini|BAF02752.1:0.17114809050669385626):0.01795822394816887213[9]):0.01037790815561474324[1],(Gemini|AHL29198.1:0.07564843836719320480,(Gemini|AFF58888.1:0.14394366320756693534,(Gemini|AGF41094.1:0.06435712688006402227,Gemini|CAJ85998.1:0.07246391281363062653):0.03449938141122809582[55]):0.01451712933017036689[13]):0.02098789166033211695[14]):0.02200773371819054330[2]):0.02278812010358131912[4],(((Gemini|AIY31184.1:0.12100208110077644019,Gemini|AEE99005.1:0.21313174364529724469):0.05209916932975737491[98],(Gemini|ADW24243.1:0.16679704367337497284,(Gemini|ABD67440.1:0.27663962837276229179,Gemini|AKS48121.1:0.11246977893212202482):0.06114839275489357406[76]):0.06358244263382199779[53]):0.02060975158096443188[14],(Gemini|CAM91896.1:0.11020061040182867196,(Gemini|YP_009129272.1:0.18703682737243770839,(Gemini|YP_004958233.1:0.14998452245125837301,Gemini|AJM13604.1:0.11140459051069709351):0.07270128146104286315[99]):0.02167847997498007981[36]):0.08656260061494766567[96]):0.02815794191037124250[5]):0.01166414418643814052[1],(((Gemini|YP_001285764.1:0.21079338363103317100,Gemini|AMP46444.1:0.14913587392232383055):0.09331135084871451657[95],Gemini|YP_764516.1:0.09173139202506748469):0.01519374856505277538[15],(Gemini|YP_003622552.1:0.18165664221884392227,((Gemini|YP_001040016.1:0.08789938905741506714,Gemini|AAP73446.1:0.26947280194732270431):0.02468825779679093696[9],((Gemini|YP_002224032.1:0.19476106831681511133,Gemini|CBJ17676.1:0.05895771445960740903):0.02893071846391031404[52],Gemini|AMK07575.1:0.17035272495771591750):0.03276681006130333779[36]):0.07210626132046467351[10]):0.03904741531509432068[2]):0.02573144532140774973[0]):0.03360235923367924787[1]):0.02814611586353191544[5],((Gemini|YP_115511.1:0.08291425780660408029,Gemini|YP_001333687.1:0.11591495790178533987):0.20680583287430226447[100],(Gemini|ADN84041.1:0.11955172719499031209,Gemini|AAN76737.1:0.09666949550655382084):0.06279832233387090679[73]):0.06071952583698799616[43]):0.03961916458990923789[7]):0.08766094573972800508[56]):0.06316953475258113571[16]):0.02286797509103387013[2],(Gemini|YP_619883.1:0.18551863005336244328,((Gemini|YP_002941855.1:0.11693313818422584882,(Gemini|ACV83312.1:0.09461139009765284769,Gemini|AER09339.1:0.10604423674538747402):0.04829016238211744638[72]):0.02553783193095272852[20],Gemini|YP_006590064.1:0.10406531278422533060):0.02718432087299552796[40]):0.00707140726111646443[1]):0.02243901914945422779[0],(Gemini|NP_671468.1:0.15334820729939138695,Gemini|AHX57826.1:0.15969863981426446475):0.01679591943697845979[20]):0.01524057720922063729[0],Gemini|AFD54490.1:0.15031088465184033742):0.03295594336466700636[7],Gemini|YP_007250561.1:0.13940006644798463853):0.10275224976858898795[41],((Gemini|AAL96826.1:0.14739640808117462911,(Gemini|YP_003828907.1:0.12175677318388859749,Gemini|CRI68211.1:0.19602687949763694664):0.03033189050476045936[53]):0.01149698647795314305[32],(Gemini|AGV02076.1:0.11753463962667790033,(Gemini|AFM38721.1:0.20282480317359427580,(Gemini|YP_003966137.1:0.10651081994377750717,Gemini|NP_066185.1:0.12479577128019717080):0.06653609801414421543[96]):0.04329030463294439995[79]):0.02373073766381517472[21]):0.12223598262431023542[81]):0.20422751786241807870[84]):0.23574024527143114249[97]):0.17563113603905119997[80]):0.28725119854421599097[76]):0.15223113025171217116[31],pCRESS9|KXT29014.1:1.10105845485610887025):0.14625912186657810676[88],(((((PpulchraPlasmids|OLY79389.1:0.24113046212459912643,PpulchraPlasmids|OLY79419.1:0.24057275781800652181):0.32893244789027720199[100],((((PpulchraPlasmids|AAF36423.1:0.06440453849694070809,PpulchraPlasmids|AAF36422.1:0.04536401247550955057):0.08986449915897117002[84],PpulchraPlasmids|AAF36424.1:0.13897912974908038786):1.08109127421256867585[100],PpulchraPlasmids|OLY79699.1:0.93171958361306450591):0.11315351139369417277[31],(PpulchraPlasmids|OMJ09562.1:0.60083770964350602384,((PpulchraPlasmids|OMJ21113.1:0.05181208638673510947,(PpulchraPlasmids|OMJ13215.1:0.01935920099198565683,PpulchraPlasmids|OMJ28371.1:0.09957608573733721380):0.07426664599421224644[72]):0.27001408883068267475[99],PpulchraPlasmids|OMJ11569.1:0.30151792518922937436):0.10211632973186098028[75]):0.10545973132474005529[67]):0.06947545252134328109[28]):0.13037121766854317673[47],PpulchraPlasmids|ETO15557.1:0.61748388441949908501):0.20079327108646541356[64],(((CRESSV6-Wastewater|AUM62043.1:0.00317642716272376795,CRESSV6-Wastewater|AUM61624.1:0.01226838988602032242):0.37532171282155168957[100],CRESSV6-Wastewater|AUM61713.1:0.22981862276568532089):0.05464405165594020397[54],(CRESSV6-Wastewater|AUM61738.1:0.65032962996232557273,CRESSV6-Wastewater|AUM61719.1:0.25994471820648762383):0.10757684419202678816[45]):0.42854662395263481978[98]):0.21996472815343329210[79],(((CRESSV6|KM598390:0.59687179480743002280,CRESSV6|KT149395:0.58777241520520728812):0.16575005565228506654[76],CRESSV6|KP153501:0.59199606015201455822):0.13659223392920255558[52],(((CRESSV6|KP005454:0.39824300312812827007,CRESSV6|KM510189:0.45472565098225742330):0.65001262171579077265[100],CRESSV6|KT732829:0.65218910519371242618):0.27563947262869287202[96],(CRESSV6|KM874358:0.51603661319256977080,CRESSV6|AJD07486.1:0.58129115790600138780):0.31690261125771479467[93]):0.06072481439256361552[36]):0.44903822580252183982[100]):0.47121967330495817539[100]):0.53061797863028581368[100]):0.15720794203615415241[74],((((pCRESS4|CEI31812.1:0.26183570171639075630,pCRESS4|WP_017824301.1:0.21645885245486293225):0.36446218523120554877[100],((pCRESS4|CRY97508.1:0.62659398593441018033,((pCRESS4|WP_021639163.1:0.44130789730724317987,(pCRESS4|CRY93789.1:0.31107043906340003936,pCRESS4|CBL40434.1:0.51615784599555469825):0.06764328716727099899[40]):0.07976095152312537295[51],(pCRESS4|WP_007889993.1:0.62235535004862307940,pCRESS4|CDA18875.1:0.69862676957547353851):0.11329257505309363319[45]):0.07799270906417185567[53]):0.15781471583945555492[78],(pCRESS4|WP_044572803.1:0.59456697549328496599,((pCRESS4|GAC78794.1:0.41881967038613265863,((pCRESS4|WP_005464724.1:0.00493117504373369580,pCRESS4|WP_067940518.1:0.04106698476112924379):0.02995529491592729618[97],pCRESS4|WP_043534193.1:0.09809544582324447592):0.13937129470761169814[98]):0.40817567753703237754[100],(pCRESS4|WP_052038917.1:0.00441563990605423669,pCRESS4|WP_006681830.1:0.00000093905322139818):0.37578961260231164010[100]):0.16563579076774512244[78]):0.12112501519816781803[73]):0.07894163978018520211[26]):0.09312370523780061360[43],(pCRESS4|WP_000186194.1:0.44126272112291908822,pCRESS4|WP_000818357.1:0.44606070460511376696):0.25017069455191959237[100]):0.91353649174035589375[100],((((pCRESS6|WP_061343647.1:0.40702165676630819302,pCRESS6|WP_017371219.1:0.39436510252724288339):0.39681935739254342588[100],((((pCRESS6|WP_039670385.1:0.19154154346332957770,(pCRESS6|WP_018030886.1:0.13729345831922548649,pCRESS6|WP_003104234.1:0.10717042362668124933):0.10914534132283520984[100]):0.23616897595412328359[100],((pCRESS6|KST89836.1:0.06335185439185954392,pCRESS6|WP_025016923.1:0.06964034710369391978):0.36470071237036016942[100],((pCRESS6|WP_018380019.1:0.12981691963422159475,pCRESS6|EOB33201.1:0.14987136619922047931):0.07809378468302811960[95],((pCRESS6|WP_000746010.1:0.12830222736957277996,pCRESS6|WP_044671103.1:0.09689749136014408348):0.02986264161387786401[82],(pCRESS6|WP_003048523.1:0.04501390840668721000,pCRESS6|WP_039694464.1:0.09137948118252317564):0.04550062032109697396[99]):0.13960750531435722754[100]):0.13570517075741833635[99]):0.08099104010682675603[72]):0.06603340019031492258[77],((pCRESS6|BAM66968.1:0.12230759889736388113,pCRESS6|WP_019299400.1:0.11343904289642373073):0.29843554360020396743[100],(pCRESS6|WP_032941943.1:0.03009575445377061717,pCRESS6|WP_058223604.1:0.02713368000751779571):0.37410469678427027418[100]):0.09206011959197200889[91]):0.29691444600887212379[97],(pCRESS6|WP_014571792.1:0.84027019004228986621,((((pCRESS6|WP_051176704.1:0.55876473772865353773,(pCRESS6|WP_036321578.1:0.30063837967189155354,(pCRESS6|WP_054952722.1:0.01893466730822329636,pCRESS6|WP_041290927.1:0.02790606693900860025):0.13983057414194027812[100]):0.27560333799976338698[100]):0.19383475876221070711[99],(pCRESS6|WP_022765681.1:0.64244480623146160703,pCRESS6|WP_052506726.1:0.64647609028860475444):0.09219603318707657313[42]):0.11908119046943431429[58],((pCRESS6|ADX23728.1:0.42279948885078810772,(pCRESS6|WP_056938517.1:0.42914724755914118726,((pCRESS6|WP_034704841.1:0.06631543293929857696,pCRESS6|WP_067483596.1:0.05199155606944982216):0.17721712641702061508[100],(((pCRESS6|WP_017649267.1:0.08023443734315720277,pCRESS6|ABJ73998.1:0.13794108538772054118):0.07710910224702807736[100],(((pCRESS6|WP_053092713.1:0.15258888484895402615,pCRESS6|KEQ49321.1:0.11066163659936645303):0.04842559423251661249[90],((((pCRESS6|WP_015647385.1:0.16582431167483557832,pCRESS6|CGE81062.1:0.19120610099241155955):0.02406874457992660282[18],(pCRESS6|KXT86702.1:0.05359282799147767340,pCRESS6|WP_032497992.1:0.17061653743333102251):0.02780699176509107001[72]):0.02114483766797495626[33],pCRESS6|WP_014623544.1:0.10400381046083319314):0.02350216334816366062[44],((pCRESS6|WP_039677656.1:0.18983300136012523640,(pCRESS6|WP_049499636.1:0.09092295469417369103,pCRESS6|WP_045759092.1:0.06716296176348943159):0.13407612518703343474[100]):0.04976060522684548626[63],((pCRESS6|WP_020997784.1:0.07266191987177499090,pCRESS6|WP_044774450.1:0.04230826036079099461):0.15899837918050241381[100],pCRESS6|WP_027972054.1:0.13389915166128976698):0.02426313130036860527[32]):0.01627966639388004136[36]):0.03450373778126299140[69]):0.04877861889343478724[86],(pCRESS6|WP_003035134.1:0.04797343472950183624,pCRESS6|WP_049476139.1:0.07001272846635811509):0.07493909813693837296[100]):0.05595751199565251327[87]):0.04573508284223219700[90],pCRESS6|WP_003024533.1:0.23673881345266009624):0.06402037484200043260[88]):0.11193245369238818965[91]):0.05532272290697250183[57]):0.11963302941707421034[98],(((pCRESS6|WP_000044268.1:0.20431159211835733802,(pCRESS6|CMU27730.1:0.18201835445043612616,(pCRESS6|WP_047206721.1:0.03193599201892469847,pCRESS6|WP_000201649.1:0.05711078020791430371):0.09799857378619784842[100]):0.09994841475143946552[99]):0.09268461353986252871[96],((pCRESS6|WP_000791389.1:0.17768271443682040478,pCRESS6|WP_003032217.1:0.39142377280569667919):0.03983115608110875810[43],(pCRESS6|WP_024400359.1:0.10964119373594294338,((pCRESS6|WP_001034312.1:0.04245404412362604324,pCRESS6|WP_024385235.1:0.06105941905470047004):0.02044983091623711266[70],pCRESS6|WP_004183001.1:0.07253196863189693977):0.02410345910808177339[70]):0.14906717685131928852[100]):0.05067950005703714123[54]):0.09963007850017153999[91],(pCRESS6|WP_044762265.1:0.09548001476422604550,(pCRESS6|WP_020999261.1:0.11561742520777201770,pCRESS6|WP_018376545.1:0.09059862330597433233):0.05101552203515396372[75]):0.41708800547005409065[100]):0.13038884019651750257[97]):0.20610927469191892003[100]):0.18127969020802597866[82],pCRESS6|AEU41945.1:0.67624086728411803016):0.08578562932027475330[64]):0.10707145016193150844[64]):0.13073017245578374967[44]):0.50786316797942532286[100],((pCRESS8|WP_062359070.1:0.73613616756731647950,(((pCRESS8|YP_006939186.1:1.10424709009854682407,(pCRESS8|WP_002821392.1:0.82485344421204886967,(pCRESS8|WP_057827085.1:0.50884455643789927581,(pCRESS8|WP_057827851.1:0.14588593580937095351,pCRESS8|WP_057906729.1:0.04138007044899726095):0.48726512872850902269[100]):0.19284584195353349223[93]):0.10841863527047894411[59]):0.05766874228630895771[20],(pCRESS8|AKG47101.1:0.88376476586218977172,(((((pCRESS8|WP_006499656.1:0.63208067359729491752,pCRESS8|WP_003665528.1:0.55936810851775164988):0.09531901904339405707[49],pCRESS8|KRN07545.1:0.61434932505935424540):0.07279891068386884490[15],pCRESS8|CUR41281.1:0.70463818233371533495):0.05562418811759171217[22],pCRESS8|WP_046923918.1:0.69195472996094897766):0.09918328476893081835[50],(pCRESS8|WP_034540695.1:0.20567271308609932912,pCRESS8|WP_046025501.1:0.30292643015421671926):0.62086130961534802264[100]):0.05998106217650002459[47]):0.11795595854898896504[57]):0.03625725965524011912[14],(((pCRESS8|WP_016356676.1:0.14741162628425780179,pCRESS8|WP_016622553.1:0.11067692058575641367):0.67316859410021412380[100],((pCRESS8|WP_012845653.1:0.14684920502044712665,(pCRESS8|CDI42894.1:0.21655142836975294074,((pCRESS8|CDA26462.1:0.09620721009658966816,(pCRESS8|WP_003549058.1:0.05049199925219290813,pCRESS8|KRN00682.1:0.04261630700391484672):0.09365197940178589253[100]):0.05074617190268582850[71],(pCRESS8|KRK41125.1:0.18281981965136520629,pCRESS8|CDI43023.1:0.08744287226077220032):0.04285687198010561944[49]):0.07774933973742535753[52]):0.06020929498780380751[43]):0.34712744403129297543[100],((pCRESS8|WP_011254167.1:0.38106112778060236002,pCRESS8|WP_056985318.1:0.50699674634873914680):0.16964415811237701370[75],((pCRESS8|WP_014567781.1:0.32973718353966147143,(pCRESS8|WP_060461663.1:0.14597455390926342145,pCRESS8|WP_007125042.1:0.12288105508545145439):0.09972237734452264746[95]):0.13948817037692839982[95],((pCRESS8|WP_049150683.1:0.29606356889347901218,(pCRESS8|WP_046324376.1:0.26481933071666885482,(pCRESS8|WP_013641468.1:0.13161547545605487008,pCRESS8|WP_008472153.1:0.09969517180248524402):0.50882629101872656197[100]):0.04677340237422658586[50]):0.09704658746860961305[72],(pCRESS8|WP_008469878.1:0.26692707649569569206,pCRESS8|WP_013641481.1:0.45138847481783817006):0.20752027707682291213[96]):0.08170100974145881600[49]):0.05603540135057822474[41]):0.07031091072150165433[50]):0.32443607920524292210[100]):0.06392603707833180438[13],((pCRESS8|WP_004900270.1:0.59645218905471364401,pCRESS8|EEJ43069.1:0.48543935945570321211):0.27506336170162387633[94],(((pCRESS8|WP_016226904.1:0.32365687863647613076,pCRESS8|SCH55298.1:0.52929170958091542598):0.27955946693314953633[100],((pCRESS8|WP_000093566.1:0.00000093905322139818,((pCRESS8|WP_042900192.1:0.00245005745080449427,pCRESS8|WP_050492321.1:0.00087511473823178416):0.00131034177301381986[80],pCRESS8|KXA58447.1:0.00215741631642361572):0.00432776226853670565[73]):0.66345107915131196208[100],((pCRESS8|WP_051448806.1:0.17201034227207831484,(pCRESS8|WP_050444210.1:0.06750362529010282830,pCRESS8|WP_010817837.1:0.04364883372684102980):0.18593395201584372867[100]):0.31931303375109060250[100],((pCRESS8|CYW87437.1:0.04443427200310839237,pCRESS8|CYX46115.1:0.10759790143976893928):0.06973865770383382012[94],(pCRESS8|WP_024410839.1:0.05660215308440028070,(pCRESS8|WP_049523992.1:0.12440638380041769973,(pCRESS8|WP_033683822.1:0.01244177037092978125,pCRESS8|EFO53527.1:0.01805408073014794917):0.07143925631602283943[100]):0.17816447418119624180[99]):0.10249180848188660375[70]):0.34102557755971529829[100]):0.13872810380057712387[80]):0.04610360015914198584[31]):0.06083373623621761428[36],pCRESS8|ABP89830.1:0.92857230089231224124):0.03626159464152269724[45]):0.09154163391536404570[27]):0.06383907918336012566[22]):0.11179755178372556557[65]):0.22165914874432976478[96],(((pCRESS7|CCY61699.1:0.33447708884619936631,pCRESS7|CCZ93342.1:0.30062845519227254787):0.16837517889956210571[100],((pCRESS7|CDE19587.1:0.79445486676207144328,pCRESS7|WP_028509833.1:0.74107756617721198822):0.23130018535345017727[55],(pCRESS7|WP_019282500.1:0.57668484349074156103,(((pCRESS7|KXT29039.1:0.38992745984644094914,(pCRESS7|YP_001966814.1:0.25090901231444856512,(pCRESS7|WP_011161011.1:0.04066216751474315688,(((pCRESS7|YP_006961027.1:0.03492851948036461429,pCRESS7|YP_003617079.1:0.04422689507624073119):0.10696013916903854279[100],pCRESS7|ABC65794.1:0.15969196467116217319):0.05834210648470305322[56],pCRESS7|ABC65805.1:0.06959051444024105804):0.04453718571498477968[62]):0.09767886375475427763[69]):0.09740031606338518666[83]):0.22485134240131515737[98],(pCRESS7|CCZ68460.1:0.39847006329717216122,pCRESS7|SCG87263.1:0.53286815715446511632):0.33123165411196775487[100]):0.12159673661044403947[58],pCRESS7|ODR34583.1:0.43804306584741253827):0.10400372726891443653[33]):0.09407361692641955098[20]):0.06707409448440102007[31]):0.15854824850454968255[59],(pCRESS7|WP_002578150.1:0.70942043582351743680,pCRESS7|CUN62864.1:0.56532121370227073065):0.20962738398983266075[78]):0.48820883935210224180[99]):0.04838623912047650827[28]):0.05691887997747677175[22],((pCRESS5|UniRef50_W1I5Y6:0.63777894122977718538,pCRESS5|UniRef50_R5VXD3:1.10111186173081598838):0.53977170269852237094[96],((((pCRESS5|WP_061866456.1:0.62602609228859162904,((pCRESS5|WP_050238550.1:0.13950846759140680953,pCRESS5|WP_024393234.1:0.13676692963327707897):0.46725951834707729615[100],(pCRESS5|WP_029176301.1:0.20544756249027062989,pCRESS5|WP_024390948.1:0.18585550162312802769):0.51172241829058318086[100]):0.14266619557807783125[54]):0.39732420826246500623[100],(((pCRESS5|WP_067193806.1:0.10722363665163546498,((pCRESS5|WP_049478725.1:0.04642986846152558772,(pCRESS5|WP_061863770.1:0.02492324328618212431,pCRESS5|WP_061417941.1:0.00975077159081438709):0.01370866233652692258[57]):0.01812201876856612756[50],pCRESS5|WP_049535277.1:0.01549444747204262347):0.12445234563863576127[100]):0.02680174293680923428[53],(pCRESS5|WP_044771983.1:0.09065954975329286458,(pCRESS5|WP_029171254.1:0.08550940661975953150,(((pCRESS5|WP_033583888.1:0.00395165774851133789,pCRESS5|WP_049481849.1:0.00404474869970653829):0.11191874404790234931[100],pCRESS5|WP_024408358.1:0.04320140972967283916):0.03281292779189019099[69],pCRESS5|WP_039694423.1:0.04422075274406021828):0.01933871815563500268[30]):0.02778748212038506873[60]):0.09288080009257994318[96]):0.17902857524989582161[96],(pCRESS5|WP_014735272.1:0.00713792062885173877,(((pCRESS5|WP_018166163.1:0.02531546071034001394,pCRESS5|WP_024399566.1:0.02931357404142806780):0.00894377412639837943[36],(pCRESS5|WP_024382134.1:0.00202512013000631565,pCRESS5|WP_024389873.1:0.01053047353791432099):0.00794038213120907242[97]):0.02753731530140137232[80],pCRESS5|WP_053863690.1:0.01425472242275070689):0.00195772326824334266[44]):0.33392871400978196839[100]):0.55861452981080272906[100]):0.12109922579285944955[31],(pCRESS5|WP_046467524.1:0.44824130844211884650,pCRESS5|WP_038978316.1:0.46724063292627388000):0.47449824744806334831[100]):0.03049880878095808864[21],(pCRESS5|WP_058211405.1:0.35724142871773045815,pCRESS5|WP_017368666.1:0.20018299124467756056)OROOT:0.58303259611362923120[100]):0.19259453725540140456[50]):0.46288875252421329831[92]):0.12761327395164751008[19]):0.18809647817170069284[55]):0.446802726400241);
