# Supplementary material for: Multiple origins of prokaryotic and eukaryotic single-stranded DNA viruses from bacterial and archaeal plasmids
Source: Nat Commun. 2019 Jul 31;10:3425. doi: 10.1038/s41467-019-11433-0 (PMC6668415; doi:10.1038/s41467-019-11433-0)
Supplement: Supplementary file 9 — Dataset 8 [file 41467_2019_11433_MOESM9_ESM.docx]

**SUPPLEMENTARY DATA 8**

# PHYLOGENETIC TREE SHOWN IN FIGURE S5C

((((((((((((((pE194_pMV158-like|UniRef50_A8W662:0.3927336872,pE194_pMV158-like|3DKX_A:0.1564290590)92.5/100:0.2434211932,pE194_pMV158-like|UniRef50_A0A1Y4QQC0:0.3818413982)99.2/100:0.6847295405,((pE194_pMV158-like|UniRef50_A0A0E9F9L7:0.4755486395,pE194_pMV158-like|UniRef50_A0A174GG61:0.9071598478)81.5/100:0.2441133353,pE194_pMV158-like|UniRef50_W1I697:0.8053852292)99.3/100:0.6527198865)94.3/100:0.4081104394,((pE194_pMV158-like|UniRef50_U2TJ01:0.7696401836,pE194_pMV158-like|UniRef50_A0A1Y3UDM3:0.8140708991)0/14:0.1403936041,pE194_pMV158-like|UniRef50_S6CES9:1.7438295963)72/100:0.1808537833)35.2/69:0.1500562496,pE194_pMV158-like|UniRef50_A0A0H5Q8X5:1.1365306318)31.3/88:0.1110579198,(((pE194_pMV158-like|UniRef50_A0A0E9EV38:0.3876234166,pE194_pMV158-like|UniRef50_A0A0H5Q0X0:0.5603014366)88.4/100:0.1462091646,(pE194_pMV158-like|UniRef50_K9RZV9:0.4611479366,pE194_pMV158-like|UniRef50_K9RYD5:1.0828990025)91.9/99:0.2254908195)83.4/100:0.1071976479,pE194_pMV158-like|UniRef50_U2EU97:0.3105762644)94.4/100:0.2243718899)90.8/92:0.1619994139,(pE194_pMV158-like|UniRef50_U2QZX4:0.9428818959,pE194_pMV158-like|UniRef50_UPI000481AAFB:1.2319849945)32/67:0.1030790041)93.7/96:0.1747638959,(pE194_pMV158-like|UniRef50_K7YFJ8:0.7620075949,pE194_pMV158-like|UniRef50_O31070:0.4556164462)99.5/100:0.5052927404)91.7/85:0.1397351738,(pE194_pMV158-like|UniRef50_A0A0R3QHC2:0.5573908648,(pE194_pMV158-like|UniRef50_W7D2V3:0.5008153991,pE194_pMV158-like|UniRef50_S6F6F1:1.0451440842)86.4/97:0.1701008792)46.8/57:0.1464295662)28.3/20:0.0684706998,((pE194_pMV158-like|UniRef50_W1I557:0.4630981020,pE194_pMV158-like|UniRef50_A0A0Z8IYX5:0.9575959244)82/27:0.1929631160,(pE194_pMV158-like|UniRef50_A0A1B1IHL4:1.2560857639,pE194_pMV158-like|UniRef50_Q48831:1.3278432414)29.3/24:0.0407640095)86.2/22:0.1453029108)86.7/76:0.0982586832,((pE194_pMV158-like|UniRef50_A0A1W6BZG0:0.6768479033,pE194_pMV158-like|UniRef50_A0A0H5PZA0:0.8055121732)84.8/87:0.1023119212,(pE194_pMV158-like|UniRef50_UPI000300949E:0.7049845432,pE194_pMV158-like|UniRef50_A0A0R1P770:1.2924738964)83.3/81:0.1040287397)85.3/80:0.1087741231)82.9/83:0.0451415162,(pE194_pMV158-like|UniRef50_A0A0H5PZI7:0.6986996193,pE194_pMV158-like|UniRef50_G8CNR9:0.6009640931)95/100:0.2189229684)91.9/96:0.1975246545,(((((pE194_pMV158-like|UniRef50_A0A1C6AUS2:0.4152530778,pE194_pMV158-like|UniRef50_A0A0E9DRD0:0.4126693439)93.9/100:0.1865268643,(pE194_pMV158-like|UniRef50_G8CNT2:0.3075077779,pE194_pMV158-like|UniRef50_A0A0E9F4G4:0.7048569810)99.9/100:0.4678677515)81.2/100:0.0809991662,pE194_pMV158-like|UniRef50_A0A158LH93:0.7446085667)79.7/40:0.0900922968,((pE194_pMV158-like|UniRef50_A0A1Y4G0S9:0.7001200885,(((pE194_pMV158-like|UniRef50_A0A087EKU7:0.1776312472,pE194_pMV158-like|UniRef50_A0A0S2MGE2:0.2953557619)33.7/92:0.0523674323,pE194_pMV158-like|UniRef50_D3R6U9:0.2117916833)100/100:0.9400012659,pE194_pMV158-like|UniRef50_F0HMF3:0.8631945324)87.7/97:0.2336679491)78.8/89:0.1420754070,(pE194_pMV158-like|UniRef50_A0A0H5PZG0:0.7099384763,pE194_pMV158-like|UniRef50_A0A0H5QIL6:0.5846866031)99.5/100:0.4989137789)0/32:0.0106818841)93.6/96:0.1107548065,(pE194_pMV158-like|UniRef50_A0A0H5PZW4:0.7110077864,pE194_pMV158-like|UniRef50_A0A0H5PV05:0.7474897005)90.7/99:0.1599797436)31.7/94:0.0544402226)100/100:0.5252874045,(((((((((((((Gemini|ADN84041.1:0.1200156295,(Gemini|AAN76737.1:0.0884759657,(Gemini|YP_001333687.1:0.1347825494,Gemini|YP_115511.1:0.0824627242)100/100:0.2577710592)82.2/96:0.0486350966)98.7/97:0.0784590629,((((Gemini|AAB87607.1:0.0819068967,Gemini|AGK24653.1:0.1264248968)99.7/100:0.1209887700,Gemini|APP87725.1:0.3354296365)65.9/94:0.0568917692,(((((((Gemini|ABG90906.1:0.0295247768,Gemini|ACY79450.1:0.2119315890)100/99:0.1662972126,Gemini|AAP73446.1:0.2673037978)72.9/92:0.0532435903,Gemini|YP_001040016.1:0.0898322399)85.9/92:0.0308710904,((Gemini|YP_002224032.1:0.2154747391,Gemini|CBJ17676.1:0.0619693890)91.4/97:0.0296433457,Gemini|AMK07575.1:0.1885038658)94/97:0.0351385314)99.7/94:0.0874880294,Gemini|YP_003622552.1:0.2053599875)68.6/93:0.0333353540,Gemini|YP_764516.1:0.1114944355)89.9/93:0.0220369505,(((((((((Gemini|ACB44970.1:0.0598580333,Gemini|AGJ03640.1:0.1835456651)100/97:0.1182563294,((Gemini|AGV02071.1:0.1556538829,((Gemini|AAF75542.1:0.1267192082,Gemini|NP_050017.1:0.1135538351)91.6/100:0.0361528466,Gemini|ACV60535.1:0.1037911168)95.9/100:0.0282900135)84.2/100:0.0162884101,Gemini|AHA82274.1:0.1863373419)2.8/93:0.0227103531)21.5/34:0.0278828936,Gemini|AFA26437.2:0.0600597420)27.9/75:0.0172779728,(((Gemini|AFB81519.1:0.1206528778,Gemini|ACI06063.1:0.1221677237)97.5/95:0.0384256072,Gemini|AFH68197.1:0.1169545423)0/29:0.0000023286,Gemini|AFB83419.1:0.1406487262)99.4/100:0.0574303142)89.7/96:0.0346474121,Gemini|BAF02752.1:0.1946630739)25.5/14:0.0145220726,((Gemini|CAJ85998.1:0.0946504925,((Gemini|AGF41094.1:0.0599310325,Gemini|AGG08895.1:0.0985022051)29.5/56:0.0290593619,Gemini|AFF58888.1:0.1501569184)60.7/27:0.0246599842)70/47:0.0221369279,Gemini|AHL29198.1:0.0774387037)91.9/24:0.0239495468)32.3/88:0.0133482223,Gemini|ABD35287.1:0.2245130347)58.2/91:0.0123420327,((Gemini|CDW92215.1:0.1294724131,(Gemini|YP_006905839.1:0.1492453853,((Gemini|AAX39336.1:0.0228811852,Gemini|FM877473:0.0226560515)99.6/100:0.0694491692,Gemini|AEY63664.1:0.1925793409)97.7/100:0.0548897474)95.6/100:0.0455400152)84.6/93:0.0246674349,(Gemini|NP_620741.1:0.1193847578,Gemini|YP_008411025.1:0.2541735589)66.7/100:0.0257547785)89.3/91:0.0168161561)98.2/96:0.0290216796,((Gemini|ADW24243.1:0.1829660331,(Gemini|AKS48121.1:0.1205496979,Gemini|ABD67440.1:0.3078838586)86.8/100:0.0681350518)99.4/100:0.0754848017,((Gemini|YP_009129272.1:0.2035062665,(Gemini|CAM91896.1:0.1205939034,(Gemini|AJM13604.1:0.1238485774,Gemini|YP_004958233.1:0.1630597762)99.8/100:0.0896168943)16.6/47:0.0191345893)99.9/100:0.0923858875,(Gemini|AIY31184.1:0.1310286509,Gemini|AEE99005.1:0.2337580478)98.6/100:0.0634575197)11.8/45:0.0090750862)96/53:0.0294995162)88.8/54:0.0196292013)94.6/82:0.0359246876)0/77:0.0138350117,(Gemini|YP_001285764.1:0.2272495409,Gemini|AMP46444.1:0.1657450736)99.6/99:0.0876070328)95.3/97:0.0488166578)100/97:0.1291747021,(Gemini|NP_040557.1:0.1628777289,(Gemini|ALF37659.1:0.1790635250,(((Gemini|YP_003778178.1:0.0114696330,Gemini|KC108902:0.0459684086)100/100:0.1823959499,Gemini|YP_009226627.1:0.0861166168)89.4/100:0.0455424425,Gemini|ALR86823.1:0.2294620059)99.8/100:0.1460465326)93.8/100:0.0770752137)99.7/100:0.0974970638)58.6/21:0.0386830282,Gemini|YP_619883.1:0.1729638588)39/24:0.0158898282,(((((Gemini|NP_671468.1:0.1497275728,Gemini|AFD54490.1:0.1499070627)89.1/91:0.0328913693,Gemini|AHX57826.1:0.1845619882)0/51:0.0000024740,Gemini|YP_007250561.1:0.1896543168)79.3/63:0.0213709935,(((Gemini|AMW86999.1:0.1736486045,Gemini|CBH28932.1:0.1556552174)59.5/58:0.0077824513,((Gemini|CBA18089.1:0.1031086417,Gemini|FJ665283:0.0768749669)44.5/100:0.0201649338,Gemini|ALV85583.1:0.1457927646)99/97:0.0417519229)63.3/59:0.0104829911,Gemini|AGH29892.1:0.0722409877)99.2/97:0.0563195228)87.8/66:0.0201991825,((Gemini|YP_002941855.1:0.1263136312,Gemini|YP_006590064.1:0.1119834465)37.3/88:0.0216320791,(Gemini|ACV83312.1:0.1079773176,Gemini|AER09339.1:0.1124441230)99.2/100:0.0626215663)96.2/88:0.0301137553)90.7/74:0.0258379250)99.9/96:0.1508449656,(Gemini|AGV02076.1:0.1266429808,((Gemini|AAL96826.1:0.1663792982,(Gemini|YP_003828907.1:0.1316195760,Gemini|CRI68211.1:0.2109754562)83.9/97:0.0309404175)60.4/95:0.0289129931,((Gemini|NP_066185.1:0.1325131215,Gemini|YP_003966137.1:0.1201607190)97.9/100:0.0725832930,Gemini|AFM38721.1:0.2258754704)95/100:0.0476874839)27/28:0.0131039185)94.5/82:0.1114578017)99.7/100:0.2458716195,(Gemini|ACO88014.1:0.0161755620,Gemini|FJ665634:0.0068226235)100/100:0.6754098420)98.4/100:0.2255211538,(((((((Gemini|AFV91331.1:0.2834807137,Gemini|AIT39773.1:0.4556435840)66.7/99:0.0618429610,(Gemini|YP_006666531.1:0.2594677186,Gemini|YP_006666527.1:0.4627511749)88.2/95:0.0924227008)90.3/96:0.0931563153,((Gemini|YP_003915159.1:0.2075352921,Gemini|AFN80669.1:0.3007667109)98.2/100:0.1744868217,((Gemini|AFN80601.1:0.1575548457,(Gemini|YP_004089627.1:0.1210677744,Gemini|YP_006666523.1:0.1738157626)94.1/100:0.0492329986)88.8/100:0.0716466783,Gemini|YP_006666535.1:0.1280391833)100/100:0.2970382735)100/100:0.2952987581)73.9/99:0.0746833176,((((Gemini|AAK73446.1:0.0048497572,Gemini|AF003952:0.0169333545)100/100:0.2199092814,((Gemini|AHM88378.1:0.1642610256,Gemini|Q80GM6.2:0.1833373434)98.2/100:0.1050074383,((Gemini|AHM88382.1:0.1220994216,Gemini|P0C647.1:0.3012357535)57.6/97:0.0438987985,Gemini|YP_003288768.1:0.1701670181)91.4/97:0.0514048952)63.5/99:0.0707602948)99.9/100:0.1597773770,Gemini|AHM88370.1:0.4154557807)78.5/98:0.0649995908,(Gemini|YP_006273070.1:0.3527891014,Gemini|YP_009026388.1:0.4836778161)54.8/95:0.0752665197)97.1/100:0.1245297549)85.4/100:0.1017822692,Gemini|YP_009021763.1:0.4417152383)91.7/100:0.1395548030,Gemini|DQ458791:0.4415802982)99.2/100:0.2898103482,(Gemini|KT214373:0.1362483202,Gemini|JX094280:0.1837102411)100/100:0.4634372438)97.2/100:0.2401571153)93.5/100:0.1637866137,((((((Genomo|KM598389:0.6148861974,Genomo|KJ547627:0.5340412263)98.8/100:0.3481056755,(Genomo|KJ547626:0.7549485659,(Genomo|KJ547634:0.8635685076,((((((((((Genomo|YP_009115514.1:0.2305161410,Genomo|YP_003104796.1:0.2127586508)99.7/100:0.1584678870,((((Genomo|YP_009252368.1:0.1046370472,Genomo|YP_009021043.1:0.0490876422)100/100:0.2559230244,Genomo|YP_009252356.1:0.1698732459)52.1/75:0.0392223794,(Genomo|YP_009115515.1:0.1221876834,((Genomo|YP_009115519.1:0.0822305835,Genomo|KJ547638:0.0700550710)15.4/72:0.0426911187,Genomo|AIF34843.1:0.1563164324)92.5/100:0.0455498562)100/100:0.1352953535)98/100:0.0731279745,(Genomo|YP_009252353.1:0.1670272814,Genomo|AMH87666.1:0.3481582944)90.3/97:0.0775063366)79.9/95:0.0446280973)53.6/95:0.0297898508,Genomo|AGS12486.1:0.3653667312)99.5/100:0.1392943435,(Genomo|AMH87733.1:0.3847794850,(Genomo|YP_009109727.1:0.3559581715,Genomo|YP_009252362.1:0.3263124908)99.7/100:0.1878964533)54.6/96:0.0707656210)28.8/92:0.0473584292,(((((Genomo|YP_009252365.1:0.2046615226,Genomo|YP_009252359.1:0.1577132010)99/100:0.1189861888,Genomo|KT253577:0.1802143605)88.9/100:0.0637326917,Genomo|AMH87678.1:0.2305625154)99.8/100:0.1495057274,(Genomo|YP_009109733.1:0.0910372842,Genomo|KT862241:0.0562225258)100/100:0.2474920540)98.8/100:0.1418821779,((Genomo|YP_009181999.1:0.0097279370,Genomo|KT598248:0.0000020725)100/100:0.4184991268,Genomo|YP_009109729.1:0.5830370927)91.7/96:0.1546866503)98.3/81:0.1311551275)61.5/80:0.1089327461,Genomo|AMH87702.1:0.5292756414)77.8/64:0.0529298979,Genomo|YP_009164036.1:0.5685422759)39.8/55:0.0549797509,(Genomo|AJD07464.1:0.2588255271,Genomo|AMH87693.1:0.2714834347)100/100:0.4523768131)57.4/58:0.0654901973,Genomo|AMH87708.1:0.2407039143)94.8/63:0.1677189809,Genomo|YP_009109725.1:0.4842078078)100/100:0.5101545584)91.5/100:0.2066245150)65.5/92:0.1233544135)72.6/93:0.1471154700,(Genomo|KJ938716:0.3123557319,Genomo|KM821748:0.2142450131)100/100:0.9876473802)79/66:0.0827818450,Genomo|AUM61807.1:0.8826831373)74.8/65:0.0901887074,Genomo|YP_009351871.1:1.1482760747)48.8/66:0.0733906725,Genomo|KP153522:1.0378492323)98.1/100:0.2317270716)95.8/100:0.3141246735,((((((((pCRESS9|YP_007008175.1:0.0360379686,pCRESS9|WP_015083745.1:0.0311119370)76.5/97:0.0395534338,pCRESS9|YP_001708784.1:0.0994130015)87.1/98:0.0306114530,pCRESS9|ATL14544.1:0.0193876332)99.8/98:0.1195112980,(((pCRESS9|YP_001965310.1:0.1236770536,pCRESS9|YP_001965305.1:0.2646440781)72.6/95:0.0425931928,(((pCRESS9|YP_001708790.1:0.0802208079,pCRESS9|YP_007008179.1:0.0508738606)93.2/100:0.0304399940,pCRESS9|WP_013747472.1:0.0611155511)95.5/100:0.0292788231,(((pCRESS9|WP_011412958.1:0.0000025058,pCRESS9|ABC65268.1:0.0650766793)98.9/100:0.0674935852,pCRESS9|ABC65385.1:0.2399614422)82.9/100:0.0180803898,(pCRESS9|WP_011412950.1:0.0375600492,pCRESS9|CBX25033.1:0.0242946402)100/100:0.2016724128)91.1/100:0.0239714329)93.5/100:0.0370310981)91.9/96:0.0344277964,pCRESS9|YP_006961991.1:0.0729810350)99.1/99:0.0658579860)94.3/98:0.1146779632,(pCRESS9|WP_017193171.1:0.0262696440,pCRESS9|WP_017193695.1:0.0590060932)99.4/100:0.1783536703)98.1/100:0.2939455805,((((pCRESS9|YP_006959585.1:0.0000029234,pCRESS9|WP_015060110.1:0.0024594256)0/58:0.0000021028,pCRESS9|WP_011264167.1:0.0074461994)93.9/99:0.0166021752,(pCRESS9|BAD36752.1:0.0049192906,pCRESS9|WP_042068233.1:0.0000029234)99.4/100:0.0321161967)35.5/83:0.0177398232,pCRESS9|WP_012662291.1:0.0292239709)100/100:0.9612173631)90.9/100:0.2093990022,pCRESS9|KXT29032.1:1.2122351582)93.6/100:0.3149606956,pCRESS9|KXT29014.1:1.1081966068)81.9/99:0.3316495229)95.2/100:0.2855923463,((((CRESSV6|KT732829:0.7303671808,(CRESSV6|KM510189:0.4974401409,CRESSV6|KP005454:0.4347955290)100/100:0.6912903595)99.2/100:0.3186995048,(CRESSV6|KM874358:0.5467179730,CRESSV6|AJD07486.1:0.6667052426)98.6/100:0.3412127697)29.1/73:0.0782355198,((CRESSV6|KT149395:0.6515446293,CRESSV6|KM598390:0.6813925669)88.4/100:0.1626979346,CRESSV6|KP153501:0.6658153405)86.5/100:0.1459372512)100/100:0.5544330393,((((PpulchraPlasmids|OLY79419.1:0.2691769220,PpulchraPlasmids|OLY79389.1:0.2627041285)100/100:0.3756716660,((((PpulchraPlasmids|OMJ21113.1:0.0492524778,(PpulchraPlasmids|OMJ28371.1:0.1077034279,PpulchraPlasmids|OMJ13215.1:0.0219992419)40.8/99:0.0866233474)99.3/100:0.3113517173,PpulchraPlasmids|OMJ11569.1:0.3212690497)87.5/100:0.1116815548,PpulchraPlasmids|OMJ09562.1:0.6705806186)93.3/100:0.1238112255,(PpulchraPlasmids|OLY79699.1:1.0440663220,(PpulchraPlasmids|AAF36424.1:0.1488713907,(PpulchraPlasmids|AAF36423.1:0.0702373581,PpulchraPlasmids|AAF36422.1:0.0517253161)92.7/100:0.1027972156)100/100:1.1784317120)66.3/63:0.1190779204)53.1/61:0.0718573689)96.2/97:0.1725822273,PpulchraPlasmids|ETO15557.1:0.6415510621)91.3/99:0.1954214366,((CRESSV6-Wastewater|AUM61713.1:0.2589521008,(CRESSV6-Wastewater|AUM61624.1:0.0110611370,CRESSV6-Wastewater|AUM62043.1:0.0058760274)100/100:0.4020067771)60.2/98:0.0531700253,(CRESSV6-Wastewater|AUM61719.1:0.2920237857,CRESSV6-Wastewater|AUM61738.1:0.7337384849)63.2/74:0.1135394330)100/100:0.5175377546)92.5/99:0.2030473032)99.1/100:0.5410669439)99.7/100:0.6926507417,((((((((((((((Circo|YP_007974237.1:0.2017135743,Circo|AAZ78351.1:0.2415023003)92.4/100:0.1035942158,(Circo|AIF76280.1:0.0129836127,Circo|KJ641742:0.0073905037)100/100:0.2504559960)99.9/100:0.3894621776,Circo|AKO84203.1:0.4348487296)74.4/99:0.1316529377,(Circo|AGL09969.1:0.1403693459,Circo|YP_009021891.1:0.1268604172)100/100:0.3057333644)71.2/95:0.1222750376,(((Circo|AIF76265.1:0.1520381145,Circo|AIF76253.1:0.1803954576)72.9/100:0.0697007451,Circo|AIF76248.1:0.1469034936)86.7/100:0.1187130147,Circo|AIF76261.1:0.3226077229)100/100:0.3631201402)1.6/72:0.0407985275,(Circo|YP_009170674.1:0.5562835383,(((Circo|AFL02442.1:0.3979557464,(Circo|ADU77009.1:0.1432351291,((Circo|YP_764455.1:0.1264025915,Circo|NP_573442.1:0.1872576930)84.9/64:0.0460848622,(Circo|KU230452:0.0866231604,Circo|YP_009134739.1:0.1752643395)58.3/64:0.0166382024)95.7/79:0.0899329577)98.1/99:0.1282840857)84/79:0.0824900026,(Circo|YP_803546.1:0.3127436555,Circo|AEL28794.1:0.3590190271)44.3/75:0.0441509355)98.9/99:0.1933038536,(Circo|ABU48445.1:0.1281839788,Circo|AHK80894.1:0.1487191216)100/100:0.4612786625)89.8/100:0.1303149141)88.5/96:0.1104700713)94.1/100:0.1159259911,((Circo|ADD62475.1:0.4389943964,Circo|YP_004376332.1:0.5044774659)90.8/100:0.1162620960,Circo|YP_009091696.1:0.4607563599)85.6/99:0.0623216808)90.8/99:0.0986575246,Circo|YP_009000900.1:0.5507655249)90.6/100:0.1039810787,(((((((((((Circo|ADD62451.1:0.1405518329,Circo|ADD62455.1:0.1865696057)81.9/100:0.0587789873,((Circo|ADU77011.1:0.1755032638,Circo|AGJ74758.1:0.2282504755)28.3/97:0.0531517867,Circo|ADD62461.1:0.2556553309)0/41:0.0411901126)68/97:0.0501215846,Circo|AKE49355.1:0.2401695153)92.2/96:0.0640257368,(((Circo|AGJ74756.1:0.2463836514,Circo|ADY17982.1:0.3041616384)27.6/64:0.0688328295,Circo|YP_009110680.1:0.1671636462)91.3/63:0.0518434168,(Circo|ADD62457.1:0.2409293222,((Circo|AIF76266.1:0.2269544689,Circo|ADD62473.1:0.1039315058)98.8/100:0.1017419359,Circo|AEL87792.1:0.2273374479)98/100:0.0964516582)84.3/98:0.0440898645)65.7/37:0.0451363274)89.5/34:0.0492745546,((Circo|YP_009021843.1:0.3444940361,(((Circo|AEL87786.1:0.2260814362,Circo|ADI48251.1:0.2070225123)95.6/100:0.0762947151,Circo|AEL87790.1:0.1824833730)94.2/100:0.0718124782,(Circo|YP_004152331.1:0.2192466144,(Circo|ADD62471.1:0.1598372463,Circo|AIF76252.1:0.1253128997)98.1/100:0.0803188551)89.3/74:0.0370878589)0/12:0.0147590223)74.9/71:0.0478780049,(Circo|ADD62453.1:0.2549083918,Circo|AIF76249.1:0.2828433731)95.7/98:0.0873158983)71.2/95:0.0413355316)84.7/8:0.0384377711,(Circo|AGJ74760.1:0.2099861673,Circo|AIF76254.1:0.3584677801)47.1/7:0.0682356596)82.7/97:0.0440206917,(Circo|YP_009021870.1:0.2868498281,Circo|AFS65290.1:0.2779225242)95.1/100:0.1082008494)98.7/100:0.1876894672,(((Circo|YP_009047065.1:0.1789363697,Circo|YP_008130363.1:0.1759297628)52.6/95:0.0685164024,Circo|ADD62477.1:0.1215594332)61.8/95:0.0650311025,Circo|ADU76993.1:0.1941371465)100/100:0.3406143603)94.9/100:0.1520807654,(Circo|AMH87650.1:0.0670487501,Circo|AMH87652.1:0.2237785360)100/100:0.7855063028)3.1/70:0.0478013477,(Circo|YP_009237526.1:0.6139324502,Circo|YP_009116910.1:0.6419301803)86.3/67:0.1271090999)77/65:0.0520050328,Circo|AIF76251.1:0.8299578601)90.7/99:0.1391658196)93.8/99:0.1642420293,Circo|KT732825:0.9909499532)98.6/100:0.2275397810,(((CRESSV1|KM874347:0.7621329558,((CRESSV1|KT149404:0.8094532745,CRESSV1|KP153497:1.1265798148)31.2/68:0.2263123791,CRESSV1|FJ959078:0.7831518238)36.6/50:0.0562508847)60.4/74:0.1773953384,(CRESSV1|KM874309:0.7380504732,CRESSV1|KF133822:0.6383669225)94.2/99:0.2145743148)65.9/92:0.1404848546,(((((CRESSV1|KT862256:0.2785634858,CRESSV1|KF246569:0.2344768834)100/100:0.7076838506,CRESSV1|KM573766:0.7179587599)88.7/100:0.1428496754,(CRESSV1|KJ206566:0.4282453385,CRESSV1|KU043411:0.3065528816)97.7/100:0.2826154376)14.7/90:0.0983878573,CRESSV1|KU043424:0.6277004419)99.7/100:0.5771717210,(CRESSV1|KX388513.1:0.0759704642,CRESSV1|KX388515.1:0.0000027664)100/100:1.5071552150)76.2/91:0.2070652135)88.3/100:0.1837427517)96.8/100:0.1384450851,((((((((CRESSV3|JX904407:0.9048504749,CRESSV3|KT149403:0.7331555431)91.3/56:0.1781879334,CRESSV3|KP153422:0.4930711352)60.5/40:0.0512519643,(((CRESSV3|JX904075:0.3890275906,CRESSV3|JX904076:0.2709363289)81.2/79:0.0597870215,CRESSV3|JX904139:0.2949866020)99.9/86:0.2809885216,CRESSV3|KM874300:0.8189039075)66.4/50:0.0341112756)82.2/53:0.0553461872,(CRESSV3|KM874317:0.5067833065,(CRESSV3|KM874304:0.4458076041,CRESSV3|JX904581:0.5067101467)1.1/66:0.0667970969)90.7/95:0.0691492591)90.3/54:0.0675073001,CRESSV3|KP153408:0.3832885490)93.1/59:0.1086247081,CRESSV3|KT149409:0.6344302261)94.3/90:0.1266676294,CRESSV3|KM598406:0.5829503254)54.4/100:0.0690948059,(((((CRESSV3|KJ641729:0.7692386135,CRESSV3|KF738883:0.6323909733)69.6/78:0.1179353468,CRESSV3|KM972726:0.6504509121)83.4/63:0.0781341133,(CRESSV3|JN857329:0.4122027814,CRESSV3|KJ641718:0.4304937168)95.4/99:0.1773266843)40.2/51:0.0678141425,(CRESSV3|KJ641722:0.1559284424,CRESSV3|HM228875:0.1405833586)100/100:0.5547891574)74.3/81:0.1184801068,(CRESSV3|JX185418:0.5690538875,CRESSV3|KM598404:0.7724765582)95.7/100:0.2020730992)93/100:0.1062241884)99.5/100:0.1937930402)63.1/87:0.0481966711,(((((((CRESSV2|KM821764:0.7117852420,CRESSV2|KP153468:0.7651483736)63.9/55:0.1267163551,CRESSV2|KP153483:0.9242634001)95.7/40:0.2038199413,(((CRESSV2|KM821755:0.6756907626,(CRESSV2|KT732816:0.3031368122,CRESSV2|JF755415:0.2328988541)99.7/100:0.2877841174)13.7/46:0.0989881318,CRESSV2|JX904344:0.4699648246)75/32:0.0296736819,((CRESSV2|KP153364:0.4591197758,CRESSV2|JX904185:0.3815485161)98.4/100:0.2112742557,CRESSV2|JX904420:0.5922397786)76.9/88:0.1014774011)80.7/95:0.0796352114)36.6/36:0.0640000388,(CRESSV2|KT149394:0.4634003426,CRESSV2|KP153360:0.3249029570)100/100:0.5852520765)15.1/19:0.0341773122,((((((CRESSV2|KP153404:0.5612842942,CRESSV2|KC248416:0.3421453279)91.4/100:0.1200385129,CRESSV2|KT149398:0.6943426609)90.6/100:0.1057816074,(CRESSV2|KP153485:0.2581795957,(CRESSV2|KJ547648:0.1754172880,CRESSV2|KT149412:0.3197724071)87.6/100:0.1029225966)100/100:0.4845134828)95.4/98:0.1294244843,((CRESSV2|KT732823:1.0625618481,CRESSV2|KM598396:0.4669598208)57.6/99:0.2508230297,CRESSV2|JX185415:0.4133645826)99.8/100:0.4554452061)62.9/39:0.0526226594,((CRESSV2|KP153447:0.5095879841,CRESSV2|KP153369:0.8991039172)49.6/18:0.0686565408,CRESSV2|KT732819:0.7468530543)28.5/18:0.0711954976)92.6/33:0.0926158152,CRESSV2|FJ959082:0.6781810967)20.9/25:0.0666432388)87.6/73:0.0791220176,(((CRESSV2|KU043397:0.5139031967,CRESSV2|KU043406:0.4659783220)93.6/100:0.1558155175,(CRESSV2|KM573776:0.2862356908,CRESSV2|KM573767:0.3413297877)96.9/100:0.1896575629)100/100:0.3470825669,((CRESSV2|JX904107:0.5340395182,CRESSV2|JX904562:0.3345107382)99.6/100:0.3506194632,(CRESSV2|KP153377:0.6467816567,CRESSV2|KF738877:0.8709543876)95.7/97:0.2208846625)64.7/94:0.1183936419)86.2/84:0.0659936380)99.9/100:0.4685142886,((((CRESSV4|YP_009163936.1:0.5489164626,(CRESSV4|AHH31482.1:0.3993218227,(CRESSV4|YP_009021888.1:0.5899798286,CRESSV4|YP_009237559.1:0.7358148755)97.7/100:0.2832606202)37/99:0.1118278171)98/100:0.2432008434,CRESSV4|KX388505.1:0.8197637057)92.1/100:0.1574319260,((((NanoAlpha|YP_003104737.1:0.0273919386,NanoAlpha|HE654123:0.0656520219)99.9/100:0.3324308002,(NanoAlpha|AKO71308.1:0.0000028299,NanoAlpha|JF957636:0.0158031645)99.9/100:0.4232316361)99.8/100:0.4836489902,(((((NanoAlpha|AAA51422.1:0.0560008769,NanoAlpha|ACB86656.1:0.2957609732)100/100:0.2876373641,NanoAlpha|AAA51426.1:0.2246887044)99.7/100:0.2554649734,(((NanoAlpha|YP_009058890.1:0.0661619106,NanoAlpha|KC978991:0.0456150949)100/100:0.3066301397,NanoAlpha|NP_619760.1:0.1589530071)99.7/99:0.2555742408,((NanoAlpha|YP_008169853.1:0.0915946767,(NanoAlpha|YP_009246456.1:0.1346359887,NanoAlpha|ALK03646.1:0.1652174235)99.6/100:0.0907885062)63/96:0.0363707858,NanoAlpha|HM163578:0.0386847148)100/100:0.7599437843)64.1/96:0.0661770743)90.7/96:0.1456891056,(NanoAlpha|U16735:0.1836325817,NanoAlpha|KC979052:0.1885112546)100/100:0.2885489916)83.9/97:0.0932313530,(NanoAlpha|NP_619759.1:0.4203802681,(NanoAlpha|KF471057:0.5037160568,NanoAlpha|JX458742:0.3922812852)99.2/100:0.3225992012)83.7/99:0.1209631629)99.3/100:0.3442707564)97.8/100:0.2995919415,NanoAlpha|AIF34798.1:0.6216033552)99.2/100:0.3889337254)90.9/99:0.1405644891,((((((CRESSV5|JX904231:0.5542519905,((CRESSV5|KR528545:0.4610439369,CRESSV5|KR528553:0.6617037367)79.8/95:0.0789637610,CRESSV5|KT945163:0.7163548590)17.6/82:0.0939611221)38.2/95:0.1265163318,CRESSV5|KM874354:0.5428814687)53.1/68:0.0921999977,((CRESSV5|KR528554:0.3475465557,CRESSV5|KR528556:0.3123060139)90.2/100:0.1417566640,(CRESSV5|KR528551:0.4706063405,CRESSV5|KR528562:0.4816149495)96.5/100:0.1955688225)92.8/85:0.1231406495)33.3/38:0.0647947544,((CRESSV5|KR528561:0.4951273823,CRESSV5|KR528547:0.6789270499)72.5/99:0.0539088882,CRESSV5|KJ641738:0.6364752471)59/97:0.0824558223)98.6/99:0.2090811610,((CRESSV5|KP153451:0.6529176487,CRESSV5|KJ547650:0.5969794969)72.8/100:0.1610351960,CRESSV5|KJ547646:0.7538895585)95.8/99:0.2049539224)70.2/98:0.0617050830,(((((((Smaco|AIY31250.1:0.2863283347,Smaco|KT862218:0.0363615250)97.6/100:0.1453942821,Smaco|KT862221:0.1577129830)99.2/100:0.2392185733,(Smaco|KM573775:0.2200995400,Smaco|KM573771:0.2689554585)98.7/100:0.2383383406)96.4/100:0.2077387410,((((((Smaco|YP_009030025.1:0.0315910219,(Smaco|KJ577810:0.0138438936,Smaco|YP_009054985.1:0.1301712827)86.6/100:0.0255691632)99.3/100:0.1143020967,((Smaco|AMR73073.1:0.0536655621,Smaco|KX838317:0.1278741723)87/100:0.0720922878,Smaco|KX838318:0.0685806916)99.9/100:0.1805530175)52.8/97:0.0336993128,Smaco|YP_009022025.1:0.1860465752)89.1/98:0.0669660409,Smaco|KP233189:0.2231106367)99.5/100:0.2052393307,(((Smaco|YP_009252320.1:0.4803304900,Smaco|KU043420:0.6666269104)71.4/65:0.1161618978,(((Smaco|YP_009054987.1:0.0000028139,Smaco|KJ577813:0.0049330565)94/100:0.0805545659,(Smaco|YP_009118276.1:0.1191088486,Smaco|KU043428:0.0823591967)95.9/100:0.0928984617)100/100:0.2815160098,((Smaco|KU043430:0.2206592744,Smaco|KU058671:0.3276492821)99.5/100:0.2541712517,Smaco|KU043422:0.6148003109)77/52:0.0403182785)84.7/50:0.0695380211)63/85:0.1078984584,(Smaco|AIY31246.1:0.5778624750,((Smaco|YP_009163761.1:0.3444328867,Smaco|YP_009054993.1:0.1725870905)97.3/100:0.1690481815,(Smaco|AMR73071.1:0.3192748367,(((Smaco|ADB24799.1:0.0000022493,Smaco|GQ351275:0.0452631906)99.9/100:0.3507292665,Smaco|YP_009118278.1:0.7386757434)63/99:0.1074351818,Smaco|KU043403:0.4094191947)50.2/99:0.0986159990)92.1/99:0.1475357009)97.6/100:0.1922836952)51.3/83:0.0826458347)95.2/95:0.1229500454)96.2/99:0.1345253428,(Smaco|KY086298:0.3950536796,Smaco|YP_009252308.1:0.8669069880)76.1/93:0.0348911596)98.3/98:0.2497096110)10.9/56:0.1535970107,Smaco|YP_009252310.1:0.6353912544)82.7/90:0.3333758257,((Smaco|YP_009252314.1:0.3554345883,Smaco|AEW47007.1:0.2733009508)84.3/99:0.2862581721,Smaco|KM598409:0.5977342815)99.9/100:0.9672310732)94.8/100:0.5819200460,(((((Smaco|YP_009252316.1:0.5843026621,(Smaco|KU203352:0.1867630549,Smaco|KJ547633:0.1840237650)100/100:1.2086969210)81.2/98:0.1055693148,((Smaco|AJF23062.1:0.0677471754,(Smaco|AJF23060.1:0.0104420179,((Smaco|AJE25847.1:0.0058131338,(Smaco|AJE25851.1:0.0021139147,Smaco|AJE25845.1:0.2365011549)100/96:0.2703796755)76/67:0.0045146217,Smaco|KP233175:0.0000022084)100/67:0.1921333510)98.1/67:0.0882886803)87.8/67:0.0856771695,Smaco|KY086301:0.1680757406)100/100:0.5631624829)29.6/65:0.1034319589,Smaco|YP_009252326.1:0.5965641036)90.8/100:0.2334049382,Smaco|AJD07511.1:0.7814259840)93.4/100:0.3178695218,((Smaco|AIY31243.1:0.1679784389,Smaco|KT862224:0.3702122065)70.4/100:0.1347210426,Smaco|AIY31256.1:0.3481377139)99.8/100:0.6538163391)96.7/100:0.3958527288)99.7/98:0.8409430636)28.8/67:0.0832991945)100/100:0.5634896732)68.6/81:0.1324856568)94.7/98:0.2255955453,(((((((pCRESS1|CUO57637.1:0.3772041334,pCRESS1|CUO23215.1:0.2254123687)98.3/100:0.1921964713,pCRESS1|CDF01935.1:0.7224401932)76.6/100:0.0817829391,pCRESS1|WP_053982727.1:0.8348364121)86.8/100:0.0876865096,((pCRESS1|WP_003102166.1:0.0742037389,(pCRESS1|WP_029176105.1:0.1438229194,pCRESS1|WP_000032131.1:0.1343266851)19.9/96:0.0402122858)99.2/100:0.2366402803,((pCRESS1|WP_062004798.1:0.1753014051,(pCRESS1|WP_003030931.1:0.0757316343,(pCRESS1|WP_047207334.1:0.1123711772,pCRESS1|WP_029690610.1:0.1686851242)54.8/100:0.0300086819)100/100:0.1768690660)87.3/100:0.0829446026,pCRESS1|WP_029694263.1:0.3204491759)96.4/100:0.1620616717)100/100:0.4909240066)95.2/96:0.1797414924,(pCRESS1|WP_026669310.1:0.0661897366,pCRESS1|WP_026524352.1:0.0683473990)100/100:0.6806399240)58.5/93:0.1545269059,pCRESS1|CVH76026.1:1.3669581153)99.2/100:0.4594600695,(((pCRESS2|SCH60086.1:0.6197202832,pCRESS2|WP_036328238.1:0.6761084910)59.6/68:0.0690384295,((((((((((((pCRESS2|WP_044942941.1:0.0588804359,pCRESS2|WP_021629801.1:0.0332330114)99.9/100:0.1394045194,pCRESS2|CCZ45692.1:0.2276290360)98.1/100:0.1684664590,pCRESS2|WP_009301216.1:0.8679537359)82.6/100:0.0789227358,(pCRESS2|CBL15233.1:0.3746669857,(pCRESS2|WP_021882760.1:0.3553698612,pCRESS2|WP_051600858.1:0.7236017023)91.1/95:0.1207312556)28.9/56:0.1136404660)93/95:0.1086519059,pCRESS2|WP_013978550.1:0.5283867597)99.9/100:0.2670735504,(((((pCRESS2|WP_009246639.1:0.2368482226,pCRESS2|CCY69022.1:0.1545403951)98.6/100:0.1349754340,(pCRESS2|WP_052011064.1:0.3129178865,pCRESS2|CCX75435.1:0.2395081119)99/100:0.1420267862)14.8/85:0.0253901776,pCRESS2|EES75484.2:0.8512221751)54.4/97:0.0470565420,(pCRESS2|WP_024346025.1:0.1205706145,pCRESS2|WP_038278663.1:0.1568546102)100/100:0.2348921275)98.8/100:0.1204292219,(((pCRESS2|WP_007865724.1:0.0723735545,pCRESS2|WP_013270924.1:0.0850788185)100/100:0.2980169613,pCRESS2|WP_066550639.1:0.3402176141)99.8/100:0.1975367239,pCRESS2|WP_051639324.1:0.5370924473)63.9/89:0.0621174732)88.7/100:0.0515823616)93.5/65:0.0691921582,((((((pCRESS2|WP_038350939.1:0.2120001495,(pCRESS2|KJZ87129.1:0.1332500743,pCRESS2|WP_023977019.1:0.1917437123)98.1/100:0.1335677361)95.9/100:0.0920044375,pCRESS2|WP_018597672.1:0.2892279869)34.8/75:0.0409545762,((pCRESS2|CUP05665.1:0.2365473487,pCRESS2|CDC44519.1:0.3043899098)73.7/99:0.0853567222,pCRESS2|SCH17786.1:0.2096809907)99.9/100:0.1587650458)38.3/77:0.0320971518,pCRESS2|BAK32345.1:0.4213221172)91.5/99:0.0604953595,pCRESS2|WP_044928503.1:0.4044872850)41.8/94:0.0225837919,pCRESS2|WP_053167095.1:0.5080907264)92.5/99:0.0528241113)86.6/58:0.0342474661,(pCRESS2|CDE72464.1:0.3842273744,pCRESS2|CDB27189.1:0.5733574140)80.9/92:0.0539493988)86.1/56:0.0501247248,(pCRESS2|WP_051546484.1:0.7387013131,(pCRESS2|WP_066546553.1:0.2311611018,pCRESS2|WP_013271491.1:0.2140970563)100/100:0.7055077433)95.3/95:0.1799601024)21.8/23:0.0355754745,pCRESS2|WP_037404274.1:0.4646488237)87.6/59:0.0729583340,pCRESS2|WP_020072285.1:0.6067647941)96.5/100:0.1285640334,(GasCSVlike|YP_007517186.1:0.1109021535,GasCSVlike|YP_009126903.1:0.1988799088)100/100:1.0590541700)67.5/77:0.0581472555)85.7/85:0.1566670183,(((((pCRESS3|WP_055838650.1:0.8377816595,(pCRESS3|WP_016667133.1:0.6601735214,(pCRESS3|WP_002529618.1:0.4957210914,pCRESS3|WP_036342632.1:0.8784115025)11/72:0.1231643701)93.2/98:0.2230796648)98/96:0.2482158445,pCRESS3|KFI87454.1:0.6763126291)7.8/50:0.0662102206,((pCRESS3|NP_613078.1:0.8161247966,(pCRESS3|WP_023022037.1:0.5228823369,(pCRESS3|AKO38848.1:0.7512783253,pCRESS3|WP_033495900.1:1.4164632702)49.8/78:0.1728937568)94.9/99:0.2257815453)28.6/72:0.0973637345,(pCRESS3|WP_052119337.1:1.0085343669,pCRESS3|WP_025221073.1:0.8494293899)0/19:0.1095654024)74.7/49:0.1088217247)62.8/57:0.0784509194,(pCRESS3|WP_021975256.1:0.4004895669,((pCRESS3|KFI81686.1:0.2085444215,pCRESS3|WP_043170238.1:0.1594415454)99.6/100:0.1701390030,pCRESS3|WP_052825216.1:0.2713172530)96/100:0.1304957410)99.4/100:0.2070415679)97.3/75:0.2056739487,pCRESS3|WP_022856850.1:0.7978570884)100/100:0.4571873686)97.8/100:0.2090378486)84.6/99:0.1937950457)98.2/98:0.3817958460)84.8/99:0.1743902106,((((((((((((pCRESS6|WP_034704841.1:0.0754742827,pCRESS6|WP_067483596.1:0.0536685078)100/100:0.1927199021,((((((((pCRESS6|WP_049499636.1:0.1041939574,pCRESS6|WP_045759092.1:0.0690474832)100/100:0.1482459043,pCRESS6|WP_039677656.1:0.2083942391)97.9/95:0.0552028900,(pCRESS6|WP_027972054.1:0.1488621788,(pCRESS6|WP_044774450.1:0.0472062300,pCRESS6|WP_020997784.1:0.0785471475)100/100:0.1760051690)33.2/89:0.0278204997)81.7/98:0.0171691573,(((pCRESS6|KXT86702.1:0.0579843541,pCRESS6|WP_032497992.1:0.1883857386)81.6/100:0.0307768319,(pCRESS6|WP_015647385.1:0.1827196448,pCRESS6|CGE81062.1:0.2128790919)43.7/86:0.0266445766)82.8/86:0.0225430298,pCRESS6|WP_014623544.1:0.1168424085)94.2/100:0.0276379611)92.5/99:0.0379653946,(pCRESS6|KEQ49321.1:0.1193872210,pCRESS6|WP_053092713.1:0.1699692242)97.3/100:0.0527316527)97/100:0.0535606351,(pCRESS6|WP_049476139.1:0.0768080013,pCRESS6|WP_003035134.1:0.0529764641)99.8/100:0.0839109868)96.7/100:0.0584931576,(pCRESS6|WP_017649267.1:0.0860499488,pCRESS6|ABJ73998.1:0.1529841556)99.7/100:0.0849842145)95.9/100:0.0559625132,pCRESS6|WP_003024533.1:0.2533647354)88/100:0.0719197383)97.1/100:0.1326364564,pCRESS6|ADX23728.1:0.4724288134)66.2/98:0.0606077282,pCRESS6|WP_056938517.1:0.4550808386)96.4/100:0.1177902048,(((((pCRESS6|WP_000201649.1:0.0655609723,pCRESS6|WP_047206721.1:0.0326202921)99.6/100:0.1029554348,pCRESS6|CMU27730.1:0.2024580494)99/100:0.1042272062,pCRESS6|WP_000044268.1:0.2315621155)98.7/100:0.1116850048,((((pCRESS6|WP_001034312.1:0.0467370680,pCRESS6|WP_024385235.1:0.0666916499)90/100:0.0233815930,pCRESS6|WP_004183001.1:0.0774536593)92/100:0.0302212047,pCRESS6|WP_024400359.1:0.1164491460)100/100:0.1634563211,(pCRESS6|WP_000791389.1:0.1804827923,pCRESS6|WP_003032217.1:0.4394992781)88.3/100:0.0480879707)83.1/100:0.0546430210)95.5/100:0.1139795868,((pCRESS6|WP_018376545.1:0.1004170587,pCRESS6|WP_020999261.1:0.1262918217)89.3/99:0.0549610859,pCRESS6|WP_044762265.1:0.1059097867)100/100:0.4347937106)97.6/100:0.1503091255)99.9/100:0.2510522397,((((pCRESS6|WP_054952722.1:0.0213313169,pCRESS6|WP_041290927.1:0.0298117628)99.6/100:0.1640669541,pCRESS6|WP_036321578.1:0.3096488908)99.9/100:0.2774063012,pCRESS6|WP_051176704.1:0.6405585522)98.6/100:0.2122383122,(pCRESS6|WP_052506726.1:0.6933698308,pCRESS6|WP_022765681.1:0.7162293959)10.2/75:0.1025975486)72.1/94:0.1120573282)97.5/97:0.2136832397,pCRESS6|AEU41945.1:0.7267196496)40.6/90:0.0947584874,pCRESS6|WP_014571792.1:0.9589728438)94.3/99:0.1305205028,((((pCRESS6|WP_025016923.1:0.0777112379,pCRESS6|KST89836.1:0.0661086962)100/100:0.3944573108,((pCRESS6|WP_018380019.1:0.1426427228,pCRESS6|EOB33201.1:0.1618064837)99.1/100:0.0857818856,((pCRESS6|WP_044671103.1:0.1052605963,pCRESS6|WP_000746010.1:0.1393236093)87.5/100:0.0316134154,(pCRESS6|WP_003048523.1:0.0487993893,pCRESS6|WP_039694464.1:0.0982370425)98.6/100:0.0486827792)100/100:0.1526686458)99.1/100:0.1520376860)83/99:0.0872816758,((pCRESS6|WP_018030886.1:0.1516188089,pCRESS6|WP_003104234.1:0.1145846669)99.7/100:0.1203626098,pCRESS6|WP_039670385.1:0.2094977752)100/100:0.2623262069)94.4/99:0.0784866376,((pCRESS6|WP_019299400.1:0.1252464938,pCRESS6|BAM66968.1:0.1324818078)100/100:0.3369310184,(pCRESS6|WP_032941943.1:0.0342936807,pCRESS6|WP_058223604.1:0.0284567646)100/100:0.3967285545)94.1/100:0.1008823745)99.7/100:0.3184471752)69.3/98:0.1438030960,(pCRESS6|WP_061343647.1:0.4663844360,pCRESS6|WP_017371219.1:0.4202915707)100/100:0.4325991932)99.9/100:0.5787009704,(((((pCRESS7|CDE19587.1:0.9130831800,pCRESS7|WP_028509833.1:0.8466587866)90/81:0.2634332242,((((pCRESS7|CCZ68460.1:0.4422815558,pCRESS7|SCG87263.1:0.6016654964)100/100:0.3744523585,((((((pCRESS7|YP_006961027.1:0.0400519975,pCRESS7|YP_003617079.1:0.0484358254)99.9/100:0.1228027117,pCRESS7|ABC65794.1:0.1734116858)87.3/99:0.0642636820,pCRESS7|ABC65805.1:0.0735174463)89.5/99:0.0532033507,pCRESS7|WP_011161011.1:0.0439993159)99.7/99:0.1182957052,pCRESS7|YP_001966814.1:0.2665874302)91.9/100:0.1018260253,pCRESS7|KXT29039.1:0.4431073930)99.1/100:0.2536904322)94.2/100:0.1413460370,pCRESS7|ODR34583.1:0.4810253072)91.4/98:0.1263517149,pCRESS7|WP_019282500.1:0.6288340201)42/73:0.0831785725)58.4/78:0.0747039121,(pCRESS7|CCZ93342.1:0.3178400561,pCRESS7|CCY61699.1:0.3710430509)98.2/100:0.1971457001)94.6/100:0.1568367328,(pCRESS7|WP_002578150.1:0.7638136445,pCRESS7|CUN62864.1:0.6596827013)96.2/100:0.2614715258)100/100:0.6113442693,((((((((((((pCRESS8|WP_033683822.1:0.0132978433,pCRESS8|EFO53527.1:0.0200362033)99.1/100:0.0742795986,pCRESS8|WP_049523992.1:0.1395372619)100/100:0.1985802199,pCRESS8|WP_024410839.1:0.0584435428)66.3/93:0.1054677273,(pCRESS8|CYX46115.1:0.1185767903,pCRESS8|CYW87437.1:0.0477996131)91.7/100:0.0811818165)100/100:0.3648978676,(pCRESS8|WP_051448806.1:0.1883806805,(pCRESS8|WP_050444210.1:0.0720070335,pCRESS8|WP_010817837.1:0.0507940806)99.8/100:0.2050242726)100/100:0.3750735981)96.7/99:0.1476756483,(((pCRESS8|WP_042900192.1:0.0026375388,pCRESS8|WP_050492321.1:0.0023280391)0/99:0.0000202302,pCRESS8|KXA58447.1:0.0023180766)64.4/98:0.0046498162,pCRESS8|WP_000093566.1:0.0000021276)100/100:0.7458259109)29.9/89:0.0615629062,(pCRESS8|WP_016226904.1:0.3586876940,pCRESS8|SCH55298.1:0.5831489077)99.6/100:0.3188076851)86.1/98:0.0672864283,pCRESS8|ABP89830.1:1.0259312816)76.4/98:0.0387458502,(pCRESS8|EEJ43069.1:0.5246209535,pCRESS8|WP_004900270.1:0.6643862140)99.6/100:0.3079975001)94.6/79:0.1025470882,((((((pCRESS8|WP_008469878.1:0.2842564774,pCRESS8|WP_013641481.1:0.5110490520)99.7/100:0.2357144149,((pCRESS8|WP_046324376.1:0.2947819706,(pCRESS8|WP_008472153.1:0.1143414601,pCRESS8|WP_013641468.1:0.1359103789)100/100:0.5591503519)67.4/96:0.0434744539,pCRESS8|WP_049150683.1:0.3289225712)96.9/100:0.1101139487)93.4/99:0.0949011723,(pCRESS8|WP_014567781.1:0.3767348450,(pCRESS8|WP_007125042.1:0.1337910364,pCRESS8|WP_060461663.1:0.1606643312)97.3/100:0.1051716178)98.9/100:0.1534802646)90.1/99:0.0697367419,(pCRESS8|WP_011254167.1:0.4232134363,pCRESS8|WP_056985318.1:0.5671930748)97.9/95:0.1898123802)45/84:0.0790178182,(((((pCRESS8|WP_003549058.1:0.0554172427,pCRESS8|KRN00682.1:0.0462089668)99.9/100:0.1044075402,pCRESS8|CDA26462.1:0.1027643886)87.8/100:0.0594237003,(pCRESS8|CDI43023.1:0.0966630628,pCRESS8|KRK41125.1:0.1996988576)60.5/100:0.0437349590)95.1/89:0.0847017811,pCRESS8|CDI42894.1:0.2411433164)62.2/89:0.0624001857,pCRESS8|WP_012845653.1:0.1618774169)100/100:0.3934450402)100/100:0.3525930237,(pCRESS8|WP_016356676.1:0.1677160145,pCRESS8|WP_016622553.1:0.1133740409)100/100:0.7732630579)76.9/51:0.0693813635)63.3/71:0.0725063849,(((((pCRESS8|WP_057906729.1:0.0500490453,pCRESS8|WP_057827851.1:0.1572574357)100/100:0.5429074360,pCRESS8|WP_057827085.1:0.5676982189)95.5/100:0.2195933419,pCRESS8|WP_002821392.1:0.9393990498)90.5/100:0.1277907094,pCRESS8|YP_006939186.1:1.2210865585)88.6/70:0.0731073730,(((((pCRESS8|CUR41281.1:0.7839490809,pCRESS8|KRN07545.1:0.6524428855)1.7/67:0.0733085322,(pCRESS8|WP_003665528.1:0.6341495430,pCRESS8|WP_006499656.1:0.6869721106)87.2/97:0.1179262851)37.5/70:0.0714268963,pCRESS8|WP_046923918.1:0.7696191879)23.5/94:0.0832992057,(pCRESS8|WP_046025501.1:0.3367228505,pCRESS8|WP_034540695.1:0.2267667787)100/100:0.6870993642)84.1/100:0.0939238248,pCRESS8|AKG47101.1:1.0209930361)91.3/98:0.1108858920)73.6/70:0.0493683452)91.9/100:0.1426058114,pCRESS8|WP_062359070.1:0.8490117583)97.4/100:0.2455476646)75.6/95:0.0494166801)53.5/90:0.0807897397,((((pCRESS4|WP_000818357.1:0.4714773424,pCRESS4|WP_000186194.1:0.5148481347)99.8/100:0.3517603978,(((pCRESS4|CBL40434.1:0.5672762533,(pCRESS4|CRY93789.1:0.3374019945,pCRESS4|WP_021639163.1:0.4884422849)34.9/86:0.0722035744)79/99:0.0841119270,(pCRESS4|WP_007889993.1:0.7033202383,pCRESS4|CDA18875.1:0.7548802878)87.3/96:0.1354097178)92/97:0.0767132075,pCRESS4|CRY97508.1:0.6973059727)93.2/100:0.1216420475)24.2/78:0.0990401672,((pCRESS4|WP_017824301.1:0.2332316022,pCRESS4|CEI31812.1:0.2830556212)100/100:0.3896033611,(pCRESS4|WP_044572803.1:0.6109715250,((((pCRESS4|WP_067940518.1:0.0446378416,pCRESS4|WP_005464724.1:0.0054018106)91.7/100:0.0287240592,pCRESS4|WP_043534193.1:0.1097860064)93.3/100:0.1433249380,pCRESS4|GAC78794.1:0.4700138294)100/100:0.4415646699,(pCRESS4|WP_006681830.1:0.0000028930,pCRESS4|WP_052038917.1:0.0048188261)100/100:0.4143170168)98.2/100:0.1819591626)90.1/100:0.1278567331)22.7/91:0.0754944579)100/100:1.1221840940,((((((pCRESS5|WP_024390948.1:0.2056395024,pCRESS5|WP_029176301.1:0.2223837441)100/100:0.5818491482,(pCRESS5|WP_024393234.1:0.1592335522,pCRESS5|WP_050238550.1:0.1468121575)100/100:0.5265183567)26.8/84:0.1753495301,pCRESS5|WP_061866456.1:0.7197919498)99.6/100:0.4525269436,((((((pCRESS5|WP_061417941.1:0.0106442486,pCRESS5|WP_061863770.1:0.0271712569)31.7/100:0.0146806451,pCRESS5|WP_049478725.1:0.0512526022)37.1/98:0.0195306856,pCRESS5|WP_049535277.1:0.0172032297)100/100:0.1330754435,pCRESS5|WP_067193806.1:0.1230882373)76/100:0.0350394662,(pCRESS5|WP_044771983.1:0.1060169505,(((pCRESS5|WP_024408358.1:0.0475886601,(pCRESS5|WP_033583888.1:0.0043980563,pCRESS5|WP_049481849.1:0.0043589752)100/100:0.1226501503)95.5/93:0.0381407413,pCRESS5|WP_039694423.1:0.0466081320)48.5/92:0.0218478506,pCRESS5|WP_029171254.1:0.0917835737)72.8/98:0.0262998378)99.2/100:0.1009185196)97.2/100:0.1760505152,((pCRESS5|WP_053863690.1:0.0158226593,(pCRESS5|WP_018166163.1:0.0308204646,((pCRESS5|WP_024382134.1:0.0021864403,pCRESS5|WP_024389873.1:0.0115688520)91.8/100:0.0079273588,pCRESS5|WP_024399566.1:0.0384338067)19.3/97:0.0070191335)98.8/99:0.0259447390)52/91:0.0015074365,pCRESS5|WP_014735272.1:0.0082742606)100/100:0.3872378436)100/100:0.6543145792)31.3/94:0.1039337560,((pCRESS5|WP_058211405.1:0.3877480512,pCRESS5|WP_017368666.1:0.2251318874)100/100:0.6850666806,(pCRESS5|WP_038978316.1:0.5137755636,pCRESS5|WP_046467524.1:0.5045984437)99.9/100:0.4901586207)31.1/77:0.0818393888)77.8/93:0.2159655514,(pCRESS5|UniRef50_W1I5Y6:0.7811903297,pCRESS5|UniRef50_R5VXD3:1.3825881854)OROOT:0.6742815342)99/99:0.4596996465)98.1/100:0.1556296237)27.2/70:0.2992448552)96.6/97:0.5252874045);
