# Supplementary material for: Multiple origins of prokaryotic and eukaryotic single-stranded DNA viruses from bacterial and archaeal plasmids
Source: Nat Commun. 2019 Jul 31;10:3425. doi: 10.1038/s41467-019-11433-0 (PMC6668415; doi:10.1038/s41467-019-11433-0)
Supplement: Supplementary file 10 — Dataset 9 [file 41467_2019_11433_MOESM10_ESM.docx]

**SUPPLEMENTARY DATA 9**

# PHYLOGENETIC TREE SHOWN IN FIGURE S6A

(Bact_ActinomyceNewRepAgi|493732575|ref|WP_006681830.1|/1701Bacteria_Actinobacteria_Actinobacteria_Ac:0.00402980,gi|917432205|ref|WP_052038917.1|_485_bp:0.00729066,((gi|453365874|dbj|GAC78794.1|_485_bp:0.39730330,(gi|759838613|ref|WP_043534193.1|_RNA_helicase_Actinomyces_sp._MS2_485_bp:0.09876811,(gi|1056502811|ref|WP_067940518.1|_RNA_helicase_Actinomyces_radicidentis_485_bp:0.04869952,gi|491607164|ref|WP_005464724.1|_485_bp:0.00925137):0.03607914[0.997036]):0.15463622[0.999999]):0.44637837[1.000000],(gi|765006636|ref|WP_044572803.1|_485_bp:0.57956216,((Bact_RuminococcNewRepAgi|524256822|emb|CDA18875.1|/1527Bacteria_Firmicutes_Clostridia_Clostridiales_:0.74683868,((Bact_ClostridiaNewRepAgi|545399497|ref|WP_021639163.1|/1525Bacteria_Firmicutes_Clostridia_Clostridia:0.42617271,(Bact_no_rankNewRepAgi|291561635|emb|CBL40434.1|/1453Bacteria_Firmicutes_Clostridia_Clostridiales/145:0.56873543,no_r_no_rankNewRepAgi|874572962|emb|CRY93789.1|/1462uncultured_prokaryote_485_bp:0.29286427):0.06501548[0.948588]):0.07372726[0.999896],(Bact_LachnospirNewRepAgi|495165195|ref|WP_007889993.1|/1493Bacteria_Firmicutes_Clostridia_Clostridia:0.60869451,no_r_no_rankNewRepAgi|874569067|emb|CRY97508.1|/1564uncultured_prokaryote_485_bp:0.62728326):0.10717835[0.986146]):0.05854164[0.620648]):0.14925401[1.000000],((Bact_DermabacteNewRepAgi|516435276|ref|WP_017824301.1|/1555Bacteria_Actinobacteria_Actinobacteria_Mi:0.19139082,Bact_PropionibaNewRepAgi|808425327|emb|CEI31812.1|/1673Bacteria_Actinobacteria_Actinobacteria_Propio:0.27014575):0.37403139[1.000000],((Bact_StreptococNewRepAgi|446741101|ref|WP_000818357.1|/1455Bacteria_Firmicutes_Bacilli_Lactobacillal:0.47259209,Bact_StreptococNewRepAgi|446108339|ref|WP_000186194.1|/1488Bacteria_Firmicutes_Bacilli_Lactobacillal:0.54959903):0.17012247[0.999750],(((((gi|933374795|emb|CUN62864.1|_Plasmid_replication_protein_Hungatella_hathewayi_485_bp:0.74399815,(Bact_AcetobacteNewRepAgi|524716769|emb|CDE19587.1|/1350Bacteria_Proteobacteria_Alphaproteobacteria_R:0.90588261,(((gi|1063361096|gb|ODR34583.1|_hypothetical_protein_BEI60_22705_Eisenbergiella_tayi_485_bp:0.45076290,(Bact_LachnospirNewRepAgi|524226796|emb|CCZ93342.1|/1406Bacteria_Firmicutes_Clostridia_Clostridiales_:0.33573804,Bact_ClostridiaNewRepAgi|524080755|emb|CCY61699.1|/1401Bacteria_Firmicutes_Clostridia_Clostridiales_:0.29386941):0.21586638[1.000000]):0.08918823[0.945330],(Bact_RuminococcNewRepAgi|655061540|ref|WP_028509833.1|/1453Bacteria_Firmicutes_Clostridia_Clostridia:0.83877157,(Bact_LachnospirNewRepAgi|488641492|ref|WP_002578150.1|/1451Bacteria_Firmicutes_Clostridia_Clostridia:0.84416532,Bact_Vibrionacegi|518112292|ref|WP_019282500.1|/1396Bacteria_Proteobacteria_Gammaproteobacteria_Vibr:0.45186337):0.12734628[0.978992]):0.07798849[0.990552]):0.07763248[0.980413],((Bact_AcholeplasNewRepAgi|1002389866|gb|KXT29039.1|/1402Bacteria_Tenericutes_Mollicutes_Acholeplasmat:0.36508658,(bRCRep1Gr1Bact_Acholeplasgi|190571873|ref|YP_001966814.1|/1375Bacteria_Tenericutes_Mollicutes_Achole:0.25993061,(Gr1bRCRep1Bact_Acholeplasgi|499474371|ref|WP_011161011.1|/1376Bacteria_Tenericutes_Mollicutes_Achole:0.04218193,(Gr1bRCRep1Bact_Acholeplasgi|84790141|gb|ABC65805.1|/1375Bacteria_Tenericutes_Mollicutes_Acholeplasma:0.07260911,(Gr1bRCRep1Bact_Acholeplasgi|84790090|gb|ABC65794.1|/1382Bacteria_Tenericutes_Mollicutes_Acholeplasma:0.16251744,(bRCRep1Gr1Bact_Acholeplasgi|410688119|ref|YP_006961027.1|/1377Bacteria_Tenericutes_Mollicutes_Achole:0.03695986,Gr1bRCRep1Bact_Acholeplasgi|296100131|ref|YP_003617079.1|/1375Bacteria_Tenericutes_Mollicutes_Achole:0.05117096):0.10849022[1.000000]):0.05777508[0.999982]):0.04780119[0.999431]):0.10187078[1.000000]):0.13136651[0.999990]):0.22817717[1.000000],(Bact_RuminococcNewRepAgi|524198135|emb|CCZ68460.1|/1412Bacteria_Firmicutes_Clostridia_Clostridiales_:0.43296056,gi|1052950894|emb|SCG87263.1|_Plasmid_replication_protein_uncultured_Clostridium_sp._485_bp:0.53665169):0.30508842[1.000000]):0.11116605[0.989412]):0.14012790[0.994734]):0.18022075[0.999688]):0.43693168[1.000000],(Bact_LeuconostoNewRepAgi|1011462511|ref|WP_062359070.1|/1331Bacteria_Firmicutes_Bacilli_Lactobacilla:0.71681679,((((((gi|524264796|emb|CDA26462.1|_485_bp:0.09389551,(Bact_LactobacilNewRepAgi|489644618|ref|WP_003549058.1|/1366Bacteria_Firmicutes_Bacilli_Lactobacillal:0.05412589,Bact_LactobacilNewRepAgi|948885057|gb|KRN00682.1|/1366Bacteria_Firmicutes_Bacilli_Lactobacillales_La:0.04447415):0.09270289[1.000000]):0.02287259[0.810832],((gi|550819626|emb|CDI43023.1|_485_bp:0.08462966,Bact_LactobacilNewRepAgi|948608749|gb|KRK41125.1|/1366Bacteria_Firmicutes_Bacilli_Lactobacillales_La:0.18299685):0.03811896[0.883097],(Bact_LactobacilNewRepAgi|502608600|ref|WP_012845653.1|/1415Bacteria_Firmicutes_Bacilli_Lactobacillal:0.17234907,gi|550819760|emb|CDI42894.1|_485_bp:0.20289592):0.09799514[1.000000]):0.03810408[0.979706]):0.35561532[1.000000],((Bact_LactobacilNewRepAgi|499573384|ref|WP_011254167.1|/1360Bacteria_Firmicutes_Bacilli_Lactobacillal:0.39640599,Bact_LactobacilNewRepAgi|949549434|ref|WP_056985318.1|/1338Bacteria_Firmicutes_Bacilli_Lactobacillal:0.49611775):0.17144899[1.000000],((Bact_LactobacilNewRepAgi|504380679|ref|WP_014567781.1|/1342Bacteria_Firmicutes_Bacilli_Lactobacillal:0.32694394,(Bact_LactobacilNewRepAgi|494200615|ref|WP_007125042.1|/1347Bacteria_Firmicutes_Bacilli_Lactobacillal:0.12658500,Bact_LactobacilNewRepAgi|983177949|ref|WP_060461663.1|/1350Bacteria_Firmicutes_Bacilli_Lactobacillal:0.13551616):0.09630317[0.999994]):0.14765363[1.000000],((Bact_LactobacilNewRepAgi|809080192|ref|WP_046324376.1|/1346Bacteria_Firmicutes_Bacilli_Lactobacillal:0.29166512,(Bact_LactobacilNewRepAgi|896129273|ref|WP_049150683.1|/1348Bacteria_Firmicutes_Bacilli_Lactobacillal:0.29302969,(Bact_LactobacilNewRepAgi|495747574|ref|WP_008472153.1|/1340Bacteria_Firmicutes_Bacilli_Lactobacillal:0.10651029,Bact_LactobacilNewRepAgi|503406807|ref|WP_013641468.1|/1341Bacteria_Firmicutes_Bacilli_Lactobacillal:0.12620776):0.52018468[1.000000]):0.01954277[0.387626]):0.11140834[1.000000],(Bact_LactobacilNewRepAgi|495745299|ref|WP_008469878.1|/1335Bacteria_Firmicutes_Bacilli_Lactobacillal:0.24520761,Bact_LactobacilNewRepAgi|503406820|ref|WP_013641481.1|/1345Bacteria_Firmicutes_Bacilli_Lactobacillal:0.46214789):0.19872315[1.000000]):0.06679733[0.993268]):0.06670888[0.968948]):0.06355309[0.680513]):0.36228392[1.000000],(((bRCRep1Gr1Bact_Leuconostogi|488910317|ref|WP_002821392.1|/1412Bacteria_Firmicutes_Bacilli_Lactobacil:0.87190269,(Bact_LactobacilNewRepAgi|951455024|ref|WP_057827085.1|/1437Bacteria_Firmicutes_Bacilli_Lactobacillal:0.50496561,(Bact_LactobacilNewRepAgi|951595551|ref|WP_057906729.1|/1442Bacteria_Firmicutes_Bacilli_Lactobacillal:0.05731172,Bact_LactobacilNewRepAgi|951455790|ref|WP_057827851.1|/1442Bacteria_Firmicutes_Bacilli_Lactobacillal:0.14040479):0.55266990[1.000000]):0.09934375[0.991595]):0.13180718[0.999916],(Bact_LactobacilNewRepAgi|818476398|gb|AKG47101.1|/1398Bacteria_Firmicutes_Bacilli_Lactobacillales_La:0.79528546,((Bact_LactobacilNewRepAgi|802106399|ref|WP_046025501.1|/1437Bacteria_Firmicutes_Bacilli_Lactobacillal:0.28227355,Bact_LactobacilNewRepAgi|736525609|ref|WP_034540695.1|/1453Bacteria_Firmicutes_Bacilli_Lactobacillal:0.16842150):0.57514054[1.000000],(Bact_LactobacilNewRepAgi|948892254|gb|KRN07545.1|/1423Bacteria_Firmicutes_Bacilli_Lactobacillales_La:0.60695817,(Bact_LactobacilNewRepAgi|970370106|emb|CUR41281.1|/1389Bacteria_Firmicutes_Bacilli_Lactobacillales_L:0.65519276,(Gr1bRCRep1Bact_Lactobacilgi|489761587|ref|WP_003665528.1|/1422Bacteria_Firmicutes_Bacilli_Lactobacil:0.60401315,(Bact_LactobacilNewRepAgi|822102754|ref|WP_046923918.1|/1452Bacteria_Firmicutes_Bacilli_Lactobacillal:0.58663311,Gr1bRCRep1Bact_Lactobacilgi|493545821|ref|WP_006499656.1|/1410Bacteria_Firmicutes_Bacilli_Lactobacil:0.56812308):0.10107515[0.755493]):0.06550045[0.734863]):0.04860857[0.453970]):0.11054505[0.983309]):0.07684700[0.972282]):0.09005789[0.981237]):0.08885448[0.998708],((gi|227352896|gb|EEJ43069.1|_485_bp:0.47484479,Bact_LeuconostoNewRepAgi|491038601|ref|WP_004900270.1|/1436Bacteria_Firmicutes_Bacilli_Lactobacillal:0.62241506):0.18672582[1.000000],(Bact_StreptococNewRepAgi|145689324|gb|ABP89830.1|/1422Bacteria_Firmicutes_Bacilli_Lactobacillales_St:0.88818875,((Bact_LachnospirNewRepAgi|510893728|ref|WP_016226904.1|/1409Bacteria_Firmicutes_Clostridia_Clostridia:0.35942644,gi|1052710859|emb|SCH55298.1|_Plasmid_replication_protein_uncultured_Collinsella_sp._485_bp:0.46423817):0.20888158[1.000000],((gi|446015711|ref|WP_000093566.1|_485_bp:0.00000043,(Bact_Streptococgi|913906122|ref|WP_050492321.1|/1398Bacteria_Firmicutes_Bacilli_Lactobacillales_Stre:0.00000013,(gi|985376877|gb|KXA58447.1|_485_bp:0.00214088,bRCRep1Gr1no_r_no_rankgi|757662115|ref|WP_042900192.1|/1368no_rank_485_bp:0.00240193):0.00000022[0.000000]):0.00644994[0.912720]):0.62307957[1.000000],(((Bact_StreptococNewRepAgi|992650469|emb|CYX46115.1|/1391Bacteria_Firmicutes_Bacilli_Lactobacillales_S:0.09596998,gi|994516497|emb|CYW87437.1|_485_bp:0.04099638):0.06649665[0.999761],(Bact_StreptococNewRepAgi|637012935|ref|WP_024410839.1|/1392Bacteria_Firmicutes_Bacilli_Lactobacillal:0.04529690,(Bact_StreptococNewRepAgi|896644444|ref|WP_049523992.1|/1392Bacteria_Firmicutes_Bacilli_Lactobacillal:0.11674082,(gi|727222881|ref|WP_033683822.1|_replication_protein_Streptococcus_mitis_485_bp:0.00979858,Bact_StreptococNewRepAgi|308116017|gb|EFO53527.1|/1400Bacteria_Firmicutes_Bacilli_Lactobacillales_St:0.01835396):0.06673704[1.000000]):0.19069066[1.000000]):0.09633676[0.999984]):0.34543772[1.000000],(gi|916841750|ref|WP_051448806.1|_hypothetical_protein_Viridibacillus_arenosi_485_bp:0.18865870,(Bact_EnterococcNewRepAgi|913624207|ref|WP_050444210.1|/1401Bacteria_Firmicutes_Bacilli_Lactobacillal:0.06186730,Bact_EnterococcNewRepAgi|498517862|ref|WP_010817837.1|/1401Bacteria_Firmicutes_Bacilli_Lactobacillal:0.03413002):0.17105647[1.000000]):0.34053521[1.000000]):0.14380936[0.999996]):0.06213318[0.577549]):0.07909461[0.992693]):0.04801182[0.970499]):0.07229885[0.939045]):0.10814076[0.999915]):0.05382815[0.775378],(Bact_CarnobacteNewRepAgi|511302329|ref|WP_016356676.1|/1390Bacteria_Firmicutes_Bacilli_Lactobacillal:0.13269023,gi|514893627|ref|WP_016622553.1|_plasmid_replication_protein_Enterococcus_faecalis_485_bp:0.12842646):0.69243711[1.000000]):0.05124009[0.531105],gi|410500862|ref|YP_006939186.1|_485_bp:1.08152375):0.07293212[0.958060]):0.18959096[1.000000]):0.04813374[0.823949],((((Bact_LactobacilNewRepAgi|949495867|ref|WP_056938517.1|/1444Bacteria_Firmicutes_Bacilli_Lactobacillal:0.41108849,(Bact_StreptococNewRepAgi|323126431|gb|ADX23728.1|/1431Bacteria_Firmicutes_Bacilli_Lactobacillales_St:0.41767628,((Bact_EnterococcNewRepAgi|736699323|ref|WP_034704841.1|/1450Bacteria_Firmicutes_Bacilli_Lactobacillal:0.07868687,gi|1055941163|ref|WP_067483596.1|_485_bp:0.04283198):0.16278552[1.000000],(Bact_StreptococNewRepAgi|489114682|ref|WP_003024533.1|/1461Bacteria_Firmicutes_Bacilli_Lactobacillal:0.23403941,((Bact_StreptococNewRepAgi|516245304|ref|WP_017649267.1|/1455Bacteria_Firmicutes_Bacilli_Lactobacillal:0.08788579,Bact_StreptococNewRepAgi|116108858|gb|ABJ73998.1|/1496Bacteria_Firmicutes_Bacilli_Lactobacillales_St:0.12364820):0.08643546[1.000000],((Bact_StreptococNewRepAgi|896589942|ref|WP_049476139.1|/1454Bacteria_Firmicutes_Bacilli_Lactobacillal:0.05566826,Bact_StreptococNewRepAgi|489125337|ref|WP_003035134.1|/1456Bacteria_Firmicutes_Bacilli_Lactobacillal:0.05433507):0.09308701[1.000000],((Bact_StreptococNewRepAgi|921142507|ref|WP_053092713.1|/1446Bacteria_Firmicutes_Bacilli_Lactobacillal:0.15497767,Bact_StreptococNewRepAgi|662350772|gb|KEQ49321.1|/1459Bacteria_Firmicutes_Bacilli_Lactobacillales_St:0.12337384):0.04198931[0.999995],((Bact_StreptococNewRepAgi|505460283|ref|WP_015647385.1|/1455Bacteria_Firmicutes_Bacilli_Lactobacillal:0.17349219,(Bact_StreptococNewRepAgi|504436442|ref|WP_014623544.1|/1456Bacteria_Firmicutes_Bacilli_Lactobacillal:0.10491425,(Bact_StreptococNewRepAgi|873968101|emb|CGE81062.1|/1452Bacteria_Firmicutes_Bacilli_Lactobacillales_S:0.19268267,(Bact_StreptococNewRepAgi|695295662|ref|WP_032497992.1|/1452Bacteria_Firmicutes_Bacilli_Lactobacillal:0.16765368,Bact_StreptococNewRepAgi|1002507522|gb|KXT86702.1|/1458Bacteria_Firmicutes_Bacilli_Lactobacillales_S:0.05109804):0.03279958[0.999851]):0.01679235[0.739116]):0.02509825[0.985734]):0.01399713[0.638997],(Bact_StreptococNewRepAgi|654502558|ref|WP_027972054.1|/1457Bacteria_Firmicutes_Bacilli_Lactobacillal:0.16027917,((Bact_StreptococNewRepAgi|766591324|ref|WP_044774450.1|/1455Bacteria_Firmicutes_Bacilli_Lactobacillal:0.04201264,Bact_StreptococNewRepAgi|538448394|ref|WP_020997784.1|/1456Bacteria_Firmicutes_Bacilli_Lactobacillal:0.07063792):0.16241065[1.000000],(Bact_StreptococNewRepAgi|746719592|ref|WP_039677656.1|/1454Bacteria_Firmicutes_Bacilli_Lactobacillal:0.18985384,(Bact_StreptococNewRepAgi|896617134|ref|WP_049499636.1|/1457Bacteria_Firmicutes_Bacilli_Lactobacillal:0.09122510,Bact_StreptococNewRepAgi|787744000|ref|WP_045759092.1|/1457Bacteria_Firmicutes_Bacilli_Lactobacillal:0.05473710):0.11863810[1.000000]):0.04200526[0.999891]):0.02131452[0.576455]):0.02203134[0.979895]):0.03321026[1.000000]):0.04652353[0.999952]):0.06409274[1.000000]):0.05309334[0.999984]):0.05436675[0.999023]):0.11971836[1.000000]):0.05556677[0.898209]):0.10539387[0.999996],((Bact_StreptococNewRepAgi|766570477|ref|WP_044762265.1|/1461Bacteria_Firmicutes_Bacilli_Lactobacillal:0.10376424,(Bact_StreptococNewRepAgi|517187727|ref|WP_018376545.1|/1459Bacteria_Firmicutes_Bacilli_Lactobacillal:0.08024158,Bact_StreptococNewRepAgi|538452261|ref|WP_020999261.1|/1459Bacteria_Firmicutes_Bacilli_Lactobacillal:0.11951088):0.03449808[0.556220]):0.43830913[1.000000],(Bact_StreptococNewRepAgi|489122401|ref|WP_003032217.1|/1457Bacteria_Firmicutes_Bacilli_Lactobacillal:0.39281072,((Bact_StreptococNewRepAgi|636940791|ref|WP_024400359.1|/1441Bacteria_Firmicutes_Bacilli_Lactobacillal:0.09416058,(Bact_StreptococNewRepAgi|490287353|ref|WP_004183001.1|/1456Bacteria_Firmicutes_Bacilli_Lactobacillal:0.06419479,(Bact_StreptococNewRepAgi|446957056|ref|WP_001034312.1|/1471Bacteria_Firmicutes_Bacilli_Lactobacillal:0.03820837,Bact_StreptococNewRepAgi|636879286|ref|WP_024385235.1|/1456Bacteria_Firmicutes_Bacilli_Lactobacillal:0.05834671):0.01694845[0.997694]):0.02321562[0.994703]):0.14675022[1.000000],(Bact_StreptococNewRepAgi|446714058|ref|WP_000791389.1|/1471Bacteria_Firmicutes_Bacilli_Lactobacillal:0.19985237,(Bact_StreptococNewRepAgi|445966413|ref|WP_000044268.1|/1463Bacteria_Firmicutes_Bacilli_Lactobacillal:0.19825144,(Bact_StreptococNewRepAgi|887581151|emb|CMU27730.1|/1468Bacteria_Firmicutes_Bacilli_Lactobacillales_S:0.18323635,(Bact_StreptococNewRepAgi|446123794|ref|WP_000201649.1|/1463Bacteria_Firmicutes_Bacilli_Lactobacillal:0.05678599,Bact_StreptococNewRepAgi|827365198|ref|WP_047206721.1|/1463Bacteria_Firmicutes_Bacilli_Lactobacillal:0.03466641):0.10007756[1.000000]):0.09490397[1.000000]):0.12799550[1.000000]):0.02334028[0.557558]):0.02808836[0.494167]):0.10060897[1.000000]):0.08840193[0.999880]):0.24165125[1.000000],((Bact_PropionibaNewRepAgi|916569613|ref|WP_051176704.1|/1584Bacteria_Actinobacteria_Actinobacteria_Pr:0.60493076,(Bact_MicrobacteNewRepAgi|738369377|ref|WP_036321578.1|/1492Bacteria_Actinobacteria_Actinobacteria_Mi:0.29879407,(gi|939719551|ref|WP_054952722.1|_485_bp:0.01117415,Bact_DermacoccaNewRepAgi|752621566|ref|WP_041290927.1|/1475Bacteria_Actinobacteria_Actinobacteria_Mi:0.03297303):0.15326202[1.000000]):0.28976393[1.000000]):0.14910191[0.999995],(Bact_LachnospirNewRepAgi|551021517|ref|WP_022765681.1|/1551Bacteria_Firmicutes_Clostridia_Clostridia:0.61190737,Bact_StreptococNewRepAgi|918462265|ref|WP_052506726.1|/1499Bacteria_Firmicutes_Bacilli_Lactobacillal:0.51065560):0.09545847[0.918021]):0.11446135[0.997119]):0.13170051[0.999989],((Bact_StreptococNewRepAgi|504384690|ref|WP_014571792.1|/1433Bacteria_Firmicutes_Bacilli_Lactobacillal:0.78201258,Gr1bRCRep1Bact_Streptococgi|358750970|gb|AEU41945.1|/1508Bacteria_Firmicutes_Bacilli_Lactobacillales:0.60799497):0.06239198[0.407617],((Bact_EnterococcNewRepAgi|1001889292|ref|WP_061343647.1|/1441Bacteria_Firmicutes_Bacilli_Lactobacilla:0.39953020,Bact_StreptococNewRepAgi|515940636|ref|WP_017371219.1|/1446Bacteria_Firmicutes_Bacilli_Lactobacillal:0.41903697):0.50330470[1.000000],(((gi|696366931|ref|WP_032941943.1|_485_bp:0.03057106,Bact_StreptococNewRepAgi|960317102|ref|WP_058223604.1|/1456Bacteria_Firmicutes_Bacilli_Lactobacillal:0.02224560):0.35787585[1.000000],(Bact_StreptococNewRepAgi|518129192|ref|WP_019299400.1|/1437Bacteria_Firmicutes_Bacilli_Lactobacillal:0.10000599,Bact_StreptococNewRepAgi|422001715|dbj|BAM66968.1|/1443Bacteria_Firmicutes_Bacilli_Lactobacillales_S:0.12206902):0.33167100[1.000000]):0.07712482[0.999799],((Bact_StreptococNewRepAgi|746711150|ref|WP_039670385.1|/1438Bacteria_Firmicutes_Bacilli_Lactobacillal:0.19236480,(Bact_StreptococNewRepAgi|516667150|ref|WP_018030886.1|/1436Bacteria_Firmicutes_Bacilli_Lactobacillal:0.14450490,Bact_StreptococNewRepAgi|489194916|ref|WP_003104234.1|/1448Bacteria_Firmicutes_Bacilli_Lactobacillal:0.09543358):0.10877685[1.000000]):0.26086204[1.000000],((Bact_StreptococNewRepAgi|640587184|ref|WP_025016923.1|/1430Bacteria_Firmicutes_Bacilli_Lactobacillal:0.08100176,Bact_StreptococNewRepAgi|959256163|gb|KST89836.1|/1512Bacteria_Firmicutes_Bacilli_Lactobacillales_St:0.05308021):0.34947606[1.000000],(((Bact_StreptococNewRepAgi|489138744|ref|WP_003048523.1|/1442Bacteria_Firmicutes_Bacilli_Lactobacillal:0.04333404,Bact_StreptococNewRepAgi|746742170|ref|WP_039694464.1|/1449Bacteria_Firmicutes_Bacilli_Lactobacillal:0.07783891):0.04629638[1.000000],(Bact_StreptococNewRepAgi|765385984|ref|WP_044671103.1|/1440Bacteria_Firmicutes_Bacilli_Lactobacillal:0.10043900,Bact_StreptococNewRepAgi|446668664|ref|WP_000746010.1|/1442Bacteria_Firmicutes_Bacilli_Lactobacillal:0.13056404):0.02964967[0.980268]):0.14121958[1.000000],(Bact_StreptococNewRepAgi|517191201|ref|WP_018380019.1|/1438Bacteria_Firmicutes_Bacilli_Lactobacillal:0.12898250,Bact_StreptococNewRepAgi|484887870|gb|EOB33201.1|/1484Bacteria_Firmicutes_Bacilli_Lactobacillales_St:0.15567640):0.09333103[1.000000]):0.15243813[1.000000]):0.07524090[0.993385]):0.05355702[0.999391]):0.16197868[0.999994]):0.18784260[1.000000]):0.12278651[0.994502]):0.53963669[1.000000]):0.08109487[0.767970],((gi|1008240811|ref|WP_061866456.1|_485_bp:0.62714800,((gi|636907128|ref|WP_024393234.1|_485_bp:0.12678135,gi|912699815|ref|WP_050238550.1|_485_bp:0.14810459):0.53183677[1.000000],(gi|636897466|ref|WP_024390948.1|_485_bp:0.20116597,gi|656228629|ref|WP_029176301.1|_485_bp:0.15882805):0.44197371[1.000000]):0.21795962[0.999943]):0.26000981[1.000000],((((gi|516919872|ref|WP_018166163.1|_485_bp:0.02687579,(gi|636937158|ref|WP_024399566.1|_485_bp:0.03180418,(gi|636870655|ref|WP_024382134.1|_485_bp:0.00214034,gi|636890832|ref|WP_024389873.1|_485_bp:0.00000024):0.00781894[0.999959]):0.00665323[0.952278]):0.01759648[0.987513],(gi|504548170|ref|WP_014735272.1|_485_bp:0.00938589,gi|928783372|ref|WP_053863690.1|_hypothetical_protein_Streptococcus_suis_485_bp:0.01493368):0.00299339[0.380221]):0.30040349[1.000000],((Bact_StreptococNewRepAgi|766587633|ref|WP_044771983.1|/1450Bacteria_Firmicutes_Bacilli_Lactobacillal:0.10084578,((Bact_StreptococNewRepAgi|636994187|ref|WP_024408358.1|/1452Bacteria_Firmicutes_Bacilli_Lactobacillal:0.04681482,(Bact_StreptococNewRepAgi|726979138|ref|WP_033583888.1|/1452Bacteria_Firmicutes_Bacilli_Lactobacillal:0.00424883,gi|896597713|ref|WP_049481849.1|_485_bp:0.00423749):0.10789326[1.000000]):0.03364739[1.000000],(gi|746742088|ref|WP_039694423.1|_hypothetical_protein_Streptococcus_gallolyticus_485_bp:0.04547699,gi|656223019|ref|WP_029171254.1|_hypothetical_protein_Streptococcus_suis_485_bp:0.08962157):0.00971026[0.762462]):0.03778162[0.999933]):0.09370059[1.000000],(gi|1055542165|ref|WP_067193806.1|_hypothetical_protein_Streptococcus_sp._DD10_485_bp:0.10605098,(gi|896656789|ref|WP_049535277.1|_hypothetical_protein_Streptococcus_pseudopneumoniae_485_bp:0.01632200,(gi|1002966178|ref|WP_061417941.1|_hypothetical_protein_Streptococcus_oralis_485_bp:0.00649752,(gi|1008212779|ref|WP_061863770.1|_485_bp:0.02273776,gi|896594168|ref|WP_049478725.1|_485_bp:0.04218589):0.01011581[0.975431]):0.02541070[0.999999]):0.11929947[1.000000]):0.03552158[0.760857]):0.20722759[1.000000]):0.69195569[1.000000],((gi|960289695|ref|WP_058211405.1|_485_bp:0.32913714,gi|515938083|ref|WP_017368666.1|_hypothetical_protein_Lactococcus_garvieae_485_bp:0.23577716):0.53008287[1.000000],(gi|742672785|ref|WP_038978316.1|_485_bp:0.47351891,gi|815846062|ref|WP_046467524.1|_485_bp:0.46798899):0.53484299[1.000000]):0.12245311[0.796037]):0.16474273[0.961385]):0.49133686[1.000000]):0.18463306[0.985381],((((aRR_ETO15557.1_485_bp:0.62610132,((aRR_OLY79419.1|Replication-associated_485_bp:0.23041987,aRR_OLY79389.1_485_bp:0.26391808):0.35898922[1.000000],((aRR_OMJ09562.1|Replication-associated_485_bp:0.57319081,(aRR_OMJ11569.1_485_bp:0.22472268,(aRR_OMJ21113.1|Replication-associated_485_bp:0.05033841,(aRR_OMJ28371.1_485_bp:0.11411609,aRR_OMJ13215.1_485_bp:0.01939111):0.08258650[0.767157]):0.32143549[1.000000]):0.11651897[0.988905]):0.10433194[0.993770],(aRR_OLY79699.1_485_bp:0.95966779,(gi|7108461|gb|AAF36424.1|AF106328_1_replicase_Porphyra_pulchra_485_bp:0.13562813,(bRCRep5Euka_Bangiaceaegi|7108459|gb|AAF36423.1|AF106327_1/1378Eukaryota_Bangiophyceae_Bangiales_Bang:0.06379848,Euka_BangiaceaeNewRepAgi|7108457|gb|AAF36422.1|AF106326_1/1378Eukaryota_Bangiophyceae_Bangiales_Bang:0.04401411):0.10666927[0.960475]):1.02084969[1.000000]):0.16410896[0.925813]):0.07852961[0.850215]):0.09306852[0.863462]):0.43092968[1.000000],((KP153501|CRESS_unclass_1..879_485_bp:0.61542681,(KT149395|CRESS_unclass_1..894_485_bp:0.60329423,KM598390|CRESS_unclass_1..891_485_bp:0.63911212):0.13804558[0.959560]):0.07139691[0.554130],((KM874358|CRESS_unclass_1..885_485_bp:0.56172600,aRR_AJD07486.1_485_bp:0.60967423):0.32781605[1.000000],(KT732829|CRESS_unclass_1..1092_485_bp:0.64694689,(KM510189|CRESS_unclass_1..1086_485_bp:0.45874365,KP005454|CRESS_unclass_1..1074_485_bp:0.39983074):0.59153192[1.000000]):0.25836515[1.000000]):0.14356239[0.944855]):0.47396596[1.000000]):0.41601951[1.000000],(((KP153522|CRESS_unclass_1..1053_485_bp:0.97323222,(aRR_YP_009351871.1_485_bp:1.07015281,(aRR_AUM61807.1_485_bp:0.94840573,((KJ938716|CRESS_unclass_1..1140_485_bp:0.28060752,KM821748|CRESS_unclass_1..1110_485_bp:0.20128375):0.86786424[1.000000],((KM598389|CRESS_unclass_1..1062_485_bp:0.53200785,KJ547627|CRESS_unclass_1..948_485_bp:0.48679969):0.36180644[1.000000],(KJ547626|CRESS_unclass_1..873_485_bp:0.70961495,(((((YP_009109733.1|Faeces_485_bp:0.09067920,KT862241|Genomovirus_1..1026_485_bp:0.05091890):0.25112013[1.000000],(AMH87678.1|Pacific_485_bp:0.21014264,(KT253577|Genomovirus_1..1008_485_bp:0.17134291,(YP_009252365.1|Faeces_485_bp:0.18720136,YP_009252359.1|Faeces_485_bp:0.14354067):0.10823862[1.000000]):0.05209077[0.888852]):0.11320925[1.000000]):0.13211693[1.000000],((((YP_009252353.1|Faeces_485_bp:0.14818404,AMH87666.1|Pacific_485_bp:0.32360751):0.07017784[0.999999],((YP_009115515.1|Sewageassociated_485_bp:0.11509091,(AIF34843.1|Sewageassociated_485_bp:0.14148396,(KJ547638|Genomovirus_1..984_485_bp:0.06589942,YP_009115519.1|Sewageassociated_485_bp:0.07214140):0.03998242[0.794331]):0.04504651[0.999802]):0.12902198[1.000000],(YP_009252356.1|Faeces_485_bp:0.14950946,(YP_009252368.1|Faeces_485_bp:0.10334244,YP_009021043.1|Cassava_485_bp:0.05006841):0.24553122[1.000000]):0.04065764[0.966956]):0.06276442[1.000000]):0.03597070[0.999367],(AGS12486.1|Hypericum_485_bp:0.36099621,(YP_009115514.1|Sewageassociated_485_bp:0.21468819,YP_003104796.1|Sclerotinia_485_bp:0.19597998):0.12392986[1.000000]):0.02901281[0.545720]):0.12890279[1.000000],((YP_009109727.1|Faeces_485_bp:0.33513681,YP_009252362.1|Faeces_485_bp:0.27295749):0.20175412[1.000000],(AMH87733.1|Pacific_485_bp:0.39262586,(AMH87702.1|Pacific_485_bp:0.43937426,((AJD07464.1|Odonata_485_bp:0.22845716,AMH87693.1|Pacific_485_bp:0.25296935):0.40633834[1.000000],(YP_009164036.1|Gemycircularvirus_485_bp:0.49069881,(YP_009109725.1|Faeces_485_bp:0.59356376,AMH87708.1|Pacific_485_bp:0.19801827):0.08758091[0.924506]):0.07079217[0.962069]):0.05785923[0.963314]):0.11385660[0.999993]):0.04134611[0.604296]):0.06234244[0.980024]):0.12008122[1.000000]):0.09136937[0.974673],(YP_009109729.1|Faeces_485_bp:0.55129281,(YP_009181999.1|Soybean_485_bp:0.01228127,KT598248|Genomovirus_1..1014_485_bp:0.00000019):0.37649446[1.000000]):0.07488857[0.606218]):0.54610817[1.000000],KJ547634|Genomovirus_1..993_485_bp:0.82925529):0.09981326[0.596078]):0.09188556[0.940786]):0.10908159[0.986709]):0.11752292[0.969801]):0.07752079[0.813631]):0.05732027[0.510064]):0.23424352[1.000000],(((ACO88014.1|Eragrostis_485_bp:0.01869257,FJ665634|Geminivirus_1..1104_485_bp:0.00292332):0.62928581[1.000000],((AGV02076.1|Cotton_485_bp:0.11023232,((AAL96826.1|Cotton_485_bp:0.14808251,(CRI68211.1|Pepper_485_bp:0.19443326,YP_003828907.1|Melon_485_bp:0.11883870):0.02845975[0.992532]):0.02565087[0.720283],(AFM38721.1|Jacquemontia_485_bp:0.20464331,(NP_066185.1|Horseradish_485_bp:0.11364858,YP_003966137.1|Spinach_485_bp:0.10154662):0.06393882[0.999999]):0.04903847[0.999983]):0.01679081[0.856744]):0.11461781[0.999996],((((NP_040557.1|Beet_485_bp:0.13824267,(ALF37659.1|Beet_485_bp:0.16076554,(ALR86823.1|Turnip_485_bp:0.21262731,(YP_009226627.1|Turnip_485_bp:0.08100249,(YP_003778178.1|Turnip_485_bp:0.00999399,KC108902|Geminivirus_1..1116_485_bp:0.04162900):0.15819538[1.000000]):0.04142319[0.996551]):0.13476168[1.000000]):0.06987306[0.999978]):0.11391029[1.000000],YP_619883.1|Tomato_485_bp:0.14320154):0.01187074[0.536571],(((AGH29892.1|Tomato_485_bp:0.06432276,((ALV85583.1|Pavonia_485_bp:0.12524539,(CBA18089.1|Sida_485_bp:0.08629200,FJ665283|Geminivirus_1..1080_485_bp:0.07318794):0.02309996[0.949604]):0.04018574[1.000000],(AMW86999.1|Sida_485_bp:0.16658706,CBH28932.1|Sida_485_bp:0.12942152):0.00929027[0.524641]):0.01028617[0.963867]):0.05486410[1.000000],(AHX57826.1|Jacquemontia_485_bp:0.16058778,(NP_671468.1|Macroptilium_485_bp:0.14557099,AFD54490.1|Macroptilium_485_bp:0.14691932):0.01765873[0.688943]):0.01961970[0.997667]):0.01988070[0.979029],(((ACV83312.1|Tomato_485_bp:0.10051954,AER09339.1|Cleome_485_bp:0.09696206):0.05974470[1.000000],(YP_002941855.1|Passionfruit_485_bp:0.10700850,YP_006590064.1|Soybean_485_bp:0.11169889):0.01976601[0.842075]):0.01450761[0.988908],((ADN84041.1|Rhynchosia_485_bp:0.11562754,(AAN76737.1|Macroptilium_485_bp:0.07732500,(YP_001333687.1|Corchorus_485_bp:0.12371686,YP_115511.1|Corchorus_485_bp:0.06822563):0.24026445[1.000000]):0.04419230[0.999681]):0.06740390[1.000000],((YP_001285764.1|Spilanthes_485_bp:0.22432818,AMP46444.1|Deinbollia_485_bp:0.14076054):0.06659021[1.000000],((AAB87607.1|Cowpea_485_bp:0.07731700,AGK24653.1|Soybean_485_bp:0.10583236):0.13081854[1.000000],((((ABD67440.1|Mungbean_485_bp:0.27772758,AKS48121.1|Rhynchosia_485_bp:0.10900308):0.07272031[1.000000],ADW24243.1|Kudzu_485_bp:0.14202726):0.03911147[0.997310],YP_003622552.1|Soybean_485_bp:0.15372559):0.04936882[1.000000],(((AIY31184.1|Tomato_485_bp:0.11242125,AEE99005.1|Tomato_485_bp:0.23115448):0.07431804[1.000000],((ABD35287.1|Alternanthera_485_bp:0.19999148,(((aRR_AHL29198.1_485_bp:0.06223537,CAJ85998.1|Malvastrum_485_bp:0.09504388):0.01597509[0.939600],(AFF58888.1|Clerodendrum_485_bp:0.12580050,(AGG08895.1|Lindernia_485_bp:0.07984950,AGF41094.1|Papaya_485_bp:0.06421253):0.03368175[0.999990]):0.02522117[0.999961]):0.01709769[0.808153],(BAF02752.1|Pepper_485_bp:0.17429117,(((AFB81519.1|Cotton_485_bp:0.10830106,ACI06063.1|Mesta_485_bp:0.10977177):0.03679750[1.000000],(AFB83419.1|Cotton_485_bp:0.12831655,AFH68197.1|Hollyhock_485_bp:0.11146683):0.00000020[0.000000]):0.05430406[1.000000],(AFA26437.2|Pedilanthus_485_bp:0.05273089,((ACB44970.1|Tomato_485_bp:0.05206340,AGJ03640.1|Chilli_485_bp:0.16926708):0.11346291[1.000000],(AHA82274.1|Tomato_485_bp:0.17712355,(AGV02071.1|Okra_485_bp:0.14137194,(ACV60535.1|Tomato_485_bp:0.10081282,(AAF75542.1|Tobacco_485_bp:0.11779786,NP_050017.1|Pepper_485_bp:0.09644784):0.03408099[0.999522]):0.02330633[0.999994]):0.01215827[0.955453]):0.02121509[0.828000]):0.02101813[0.555674]):0.01290741[0.556156]):0.03342230[0.999988]):0.01389052[0.806657]):0.01543280[0.938825]):0.01146164[0.703533],((CDW92215.1|Tomato_485_bp:0.12058736,(YP_006905839.1|Jatropha_485_bp:0.13958583,(AEY63664.1|Watermelon_485_bp:0.17654811,(AAX39336.1|African_485_bp:0.02149861,FM877473|Geminivirus_1..1077_485_bp:0.02163410):0.06466374[1.000000]):0.04355794[1.000000]):0.04320091[1.000000]):0.02026575[0.982947],(YP_764516.1|Okra_485_bp:0.12277095,(YP_008411025.1|Hemidesmus_485_bp:0.22156536,NP_620741.1|Tomato_485_bp:0.10755139):0.01656603[0.906872]):0.02183340[0.991830]):0.01908138[0.999921]):0.02675233[0.999902]):0.02861800[0.997840],(((AJM13604.1|Asystasia_485_bp:0.11652168,YP_004958233.1|West_485_bp:0.15577219):0.06466984[1.000000],(YP_009129272.1|Apple_485_bp:0.20286932,CAM91896.1|Tomato_485_bp:0.10011822):0.02745078[0.964266]):0.07675145[1.000000],(aRR_APP87725.1_485_bp:0.30623833,((YP_001040016.1|Tomato_485_bp:0.07218839,(AAP73446.1|Dolichos_485_bp:0.25821725,(ABG90906.1|Merremia_485_bp:0.02177213,ACY79450.1|Sweet_485_bp:0.20364026):0.14055657[1.000000]):0.04645161[0.996609]):0.02524244[0.970096],(AMK07575.1|Lycianthes_485_bp:0.17183860,(YP_002224032.1|Jatropha_485_bp:0.19405532,CBJ17676.1|Croton_485_bp:0.05915358):0.02964899[0.998714]):0.03283416[0.999939]):0.07769824[1.000000]):0.03973130[0.999924]):0.01008341[0.797179]):0.03565338[0.999975]):0.01862596[0.994312]):0.01205166[0.978225]):0.04128847[1.000000]):0.15118764[1.000000]):0.01312751[0.637249]):0.03852435[1.000000]):0.01809178[0.896217],YP_007250561.1|Tomato_485_bp:0.14743833):0.10830917[1.000000]):0.20045232[1.000000]):0.25878423[1.000000],((KT214373|Geminivirus_1..951_485_bp:0.13012224,JX094280|Geminivirus_1..948_485_bp:0.17625270):0.46072953[1.000000],(DQ458791|Geminivirus_1..1005_485_bp:0.39874011,(YP_009021763.1|Axonopus_485_bp:0.39894649,(((AHM88370.1|Maize_485_bp:0.36561814,((AAK73446.1|Maize_485_bp:0.00506301,AF003952|Geminivirus_1..1068_485_bp:0.01567685):0.22855805[1.000000],(P0C647.1|Panicum_485_bp:0.27827627,((AHM88382.1|Urochloa_485_bp:0.13377573,YP_003288768.1|Saccharum_485_bp:0.16495814):0.03074679[0.931741],(AHM88378.1|Sugarcane_485_bp:0.15300276,Q80GM6.2|Sugarcane_485_bp:0.16998197):0.12016719[1.000000]):0.02904154[0.869303]):0.04263224[0.884792]):0.15081167[1.000000]):0.06817078[0.991647],(YP_006273070.1|Wheat_485_bp:0.33499707,YP_009026388.1|Sugarcane_485_bp:0.44671073):0.07666774[0.894846]):0.11380074[0.999999],(((YP_006666535.1|Digitaria_485_bp:0.12743918,(AFN80601.1|Paspalum_485_bp:0.14715646,(YP_004089627.1|Bromus_485_bp:0.10986656,YP_006666523.1|Paspalum_485_bp:0.16417049):0.04293344[0.999977]):0.06852066[0.995399]):0.27302926[1.000000],(YP_003915159.1|Digitaria_485_bp:0.19303245,AFN80669.1|Chloris_485_bp:0.27765485):0.16714416[1.000000]):0.25753714[1.000000],((YP_006666531.1|Sporobolus_485_bp:0.24800003,YP_006666527.1|Sporobolus_485_bp:0.42720476):0.08290982[0.998963],(AIT39773.1|Switchgrass_485_bp:0.46095391,AFV91331.1|Dragonflyassociated_485_bp:0.27374700):0.05416208[0.809342]):0.08224555[0.995762]):0.06482172[0.875253]):0.08298428[0.988441]):0.13657716[0.999998]):0.23296147[1.000000]):0.22148695[1.000000]):0.08565586[0.935149]):0.21064202[0.977071],(((aRR_WP_012662291.1_485_bp:0.03524369,((gi|51172577|dbj|BAD36752.1|_485_bp:0.00446934,aRR_WP_042068233.1_485_bp:0.00000017):0.03023944[1.000000],(bRC-Rep5Bact_Acholeplasgi|410687490|ref|YP_006959585.1|/1-434Bacteria_Tenericutes_Mollicutes_Acholep:0.00000013,(gi|499583384|ref|WP_011264167.1|_485_bp:0.00678379,aRR_WP_015060110.1_485_bp:0.00224011):0.00000013[0.000000]):0.01629296[0.999983]):0.02156835[0.482844]):0.88765973[1.000000],((bRC-Rep5Bact_Acholeplasgi|515760571|ref|WP_017193171.1|/1-381Bacteria_Tenericutes_Mollicutes_Acholep:0.02018353,bRC-Rep5Bact_Acholeplasgi|515761095|ref|WP_017193695.1|/1-386Bacteria_Tenericutes_Mollicutes_Acholep:0.05601985):0.22050937[1.000000],((aRR_ATL14544.1_485_bp:0.01628547,(bRC-Rep5Bact_Acholeplasgi|169546398|ref|YP_001708784.1|/1-367Bacteria_Tenericutes_Mollicutes_Acholep:0.09844069,(bRC-Rep5Bact_Acholeplasgi|425702627|ref|YP_007008175.1|/1-392Bacteria_Tenericutes_Mollicutes_Acholep:0.03572245,gi|504896643|ref|WP_015083745.1|_485_bp:0.02913957):0.02686394[0.994181]):0.02688035[0.998014]):0.11253021[1.000000],(bRC-Rep5Bact_Acholeplasgi|410688730|ref|YP_006961991.1|/1-396Bacteria_Tenericutes_Mollicutes_Acholep:0.06636084,(((gi|503512976|ref|WP_013747472.1|_485_bp:0.05492593,(bRC-Rep5Bact_Acholeplasgi|169546405|ref|YP_001708790.1|/1-367Bacteria_Tenericutes_Mollicutes_Acholep:0.08008320,bRC-Rep5Bact_Acholeplasgi|425702632|ref|YP_007008179.1|/1-367Bacteria_Tenericutes_Mollicutes_Acholep:0.04848262):0.02727514[0.999957]):0.02666220[1.000000],((Bact_Acholeplasgi|499732216|ref|WP_011412950.1|/1-372Bacteria_Tenericutes_Mollicutes_Acholeplasmatal:0.03893103,gi|307902848|emb|CBX25033.1|_485_bp:0.02152631):0.18969255[1.000000],(aRR_ABC65385.1_485_bp:0.20854242,(Bact_Acholeplasgi|499732224|ref|WP_011412958.1|/1-368Bacteria_Tenericutes_Mollicutes_Acholeplasmatal:0.00000034,aRR_ABC65268.1_485_bp:0.05910370):0.06458625[1.000000]):0.01643436[0.936847]):0.02443639[0.998326]):0.03812353[0.999997],(bRC-Rep5Bact_Acholeplasgi|190410559|ref|YP_001965305.1|/1-376Bacteria_Tenericutes_Mollicutes_Acholep:0.24607964,bRC-Rep5Bact_Acholeplasgi|190410564|ref|YP_001965310.1|/1-377Bacteria_Tenericutes_Mollicutes_Acholep:0.11593131):0.03374606[0.892258]):0.03470498[0.997115]):0.06342842[1.000000]):0.07428399[0.985761]):0.29482857[0.999999]):0.14804820[0.916447],(Bact_AcholeplasNewRepAgi|1002389859|gb|KXT29032.1|/1-455Bacteria_Tenericutes_Mollicutes_Acholeplasma:0.70339737,Bact_AcholeplasNewRepAgi|1002389832|gb|KXT29014.1|/1-442Bacteria_Tenericutes_Mollicutes_Acholeplasma:0.99172055):0.50665384[1.000000]):0.60035271[1.000000]):0.34534481[1.000000]):0.57801073[1.000000],(((Bact_CoriobacteNewRepAgi|1016804942|emb|CVH76026.1|/1360Bacteria_Actinobacteria_Coriobacteriia_Corio:1.17748713,((Bact_LachnospirNewRepAgi|651929165|ref|WP_026669310.1|/1380Bacteria_Firmicutes_Clostridia_Clostridia:0.06821903,bRCRep2Gr2Bact_Lachnospirgi|651421429|ref|WP_026524352.1|/1382Bacteria_Firmicutes_Clostridia_Clostri:0.04530544):0.61990185[1.000000],(((Gr2bRCRep2Bact_Streptococgi|445954276|ref|WP_000032131.1|/1411Bacteria_Firmicutes_Bacilli_Lactobacil:0.09657487,(Gr2bRCRep2Bact_Streptococgi|489192824|ref|WP_003102166.1|/1395Bacteria_Firmicutes_Bacilli_Lactobacil:0.10202139,Bact_StreptococNewRepAgi|656228408|ref|WP_029176105.1|/1411Bacteria_Firmicutes_Bacilli_Lactobacillal:0.11570064):0.04396792[0.774278]):0.24762877[1.000000],(bRCRep2Gr2no_r_no_rankgi|658497599|ref|WP_029694263.1|/1424no_rank_485_bp:0.26784389,(Bact_StreptococNewRepAgi|1011060458|ref|WP_062004798.1|/1420Bacteria_Firmicutes_Bacilli_Lactobacilla:0.16440997,(Gr2bRCRep2Bact_Streptococgi|489121099|ref|WP_003030931.1|/1423Bacteria_Firmicutes_Bacilli_Lactobacil:0.06117394,(Bact_StreptococNewRepAgi|658493909|ref|WP_029690610.1|/1423Bacteria_Firmicutes_Bacilli_Lactobacillal:0.15943368,Bact_StreptococNewRepAgi|827365996|ref|WP_047207334.1|/1424Bacteria_Firmicutes_Bacilli_Lactobacillal:0.10182607):0.03551091[0.996924]):0.16732138[1.000000]):0.10250375[0.999990]):0.11599164[0.995037]):0.45274178[1.000000],(Bact_LachnospirNewRepAgi|928958162|ref|WP_053982727.1|/1360Bacteria_Firmicutes_Clostridia_Clostridia:0.78449875,(Bact_RuminococcNewRepAgi|524805683|emb|CDF01935.1|/1343Bacteria_Firmicutes_Clostridia_Clostridiales_:0.62363408,(gi|933077947|emb|CUO57637.1|_485_bp:0.32215790,gi|933018020|emb|CUO23215.1|_485_bp:0.24231440):0.16136838[1.000000]):0.07890997[0.928336]):0.06500025[0.994189]):0.16275892[0.999987]):0.14853579[0.896438]):0.37450171[1.000000],(((Bact_RuminococcNewRepAgi|518916410|ref|WP_020072285.1|/1344Bacteria_Firmicutes_Clostridia_Clostridia:0.54979791,(((Gr3_4bRCRep4no_r_no_rankgi|739545959|ref|WP_037404274.1|/1322no_rank_485_bp:0.45853575,(Bact_PlanococcaNewRepAgi|921222095|ref|WP_053167095.1|/1349Bacteria_Firmicutes_Bacilli_Bacillales_Pl:0.45863312,(bRCRep4Gr3_4Bact_Lachnospirgi|769153456|ref|WP_044928503.1|/1333Bacteria_Firmicutes_Clostridia_Clost:0.36730680,(Gr3_4bRCRep4Bact_Erysipelotgi|334296474|dbj|BAK32345.1|/1309Bacteria_Firmicutes_Erysipelotrichia_Ery:0.36718376,(Bact_LachnospirNewRepAgi|517426576|ref|WP_018597672.1|/1327Bacteria_Firmicutes_Clostridia_Clostridia:0.24942852,((gi|1053015557|emb|SCH17786.1|_485_bp:0.19129070,(gi|933361505|emb|CUP05665.1|_485_bp:0.20984063,Bact_ClostridiaNewRepAgi|524513064|emb|CDC44519.1|/1336Bacteria_Firmicutes_Clostridia_Clostridiales_:0.25678097):0.07777016[0.999006]):0.14674897[1.000000],(gi|740530313|ref|WP_038350939.1|_485_bp:0.19638770,(Bact_ClostridiaNewRepAgi|800881782|gb|KJZ87129.1|/1332Bacteria_Firmicutes_Clostridia_Clostridiales_C:0.15415149,Bact_ClostridiaNewRepAgi|565897363|ref|WP_023977019.1|/1332Bacteria_Firmicutes_Clostridia_Clostridia:0.14692191):0.12631376[1.000000]):0.06745236[0.969900]):0.04176557[0.482625]):0.03654322[0.907840]):0.04147110[0.978141]):0.03111014[0.532978]):0.04552427[0.748950]):0.03611099[0.980378],(Bact_OscillospiNewRepAgi|524374305|emb|CDB27189.1|/1309Bacteria_Firmicutes_Clostridia_Clostridiales_:0.51706493,Bact_AcidaminocNewRepAgi|524774299|emb|CDE72464.1|/1332Bacteria_Firmicutes_Negativicutes_Acidaminoco:0.35182967):0.05203055[0.827778]):0.02277763[0.903813],(((Bact_LachnospirNewRepAgi|917032612|ref|WP_051639324.1|/1354Bacteria_Firmicutes_Clostridia_Clostridia:0.52599002,(((bRCRep4Gr3_4Bact_Lachnospirgi|636819923|ref|WP_024346025.1|/1335Bacteria_Firmicutes_Clostridia_Clost:0.13322224,bRCRep4Gr3_4no_r_no_rankgi|740446819|ref|WP_038278663.1|/1341no_rank_485_bp:0.13508876):0.18731828[1.000000],((Gr3_4bRCRep4Bact_Lachnospirgi|496540491|ref|WP_009246639.1|/1343Bacteria_Firmicutes_Clostridia_Clost:0.20079841,Bact_EubacteriaNewRepAgi|524091900|emb|CCY69022.1|/1342Bacteria_Firmicutes_Clostridia_Clostridiales_:0.19942283):0.11424549[0.999999],(bRCRep4Gr3_4Bact_Ruminococcgi|534538258|gb|EES75484.2|/1432Bacteria_Firmicutes_Clostridia_Clostridia:0.76990409,(Bact_no_rankNewRepAgi|917404352|ref|WP_052011064.1|/1311Bacteria/1311bacterium_LF3_485_bp:0.27630984,Bact_LachnospirNewRepAgi|523989767|emb|CCX75435.1|/1340Bacteria_Firmicutes_Clostridia_Clostridiales_:0.24522491):0.10122468[0.999808]):0.06981841[0.961742]):0.06851074[0.999957]):0.10654003[1.000000],(gi|1054787244|ref|WP_066550639.1|_485_bp:0.31808903,(bRCRep4Gr3_4Bact_Lachnospirgi|495140917|ref|WP_007865724.1|/1335Bacteria_Firmicutes_Clostridia_Clost:0.07742263,gi|503035948|ref|WP_013270924.1|_485_bp:0.06175689):0.27642005[1.000000]):0.16967484[1.000000]):0.02957777[0.538636]):0.06794399[0.999984],(Bact_EubacteriaNewRepAgi|916994146|ref|WP_051600858.1|/1402Bacteria_Firmicutes_Clostridia_Clostridia:0.63031734,(Bact_EggerthellNewRepAgi|503744474|ref|WP_013978550.1|/1321Bacteria_Actinobacteria_Coriobacteriia_Eg:0.54087698,((Bact_RuminococcNewRepAgi|291542123|emb|CBL15233.1|/1323Bacteria_Firmicutes_Clostridia_Clostridiales_:0.37149353,Gr3_4bRCRep4no_r_no_rankgi|546651982|ref|WP_021882760.1|/1325no_rank_485_bp:0.37561564):0.07805242[0.724153],(Bact_ErysipelotNewRepAgi|496658723|ref|WP_009301216.1|/1320Bacteria_Firmicutes_Erysipelotrichia_Erys:0.79629546,(Bact_no_rankNewRepAgi|524176347|emb|CCZ45692.1|/1320Bacteria_Firmicutes/1320Firmicutes_bacterium_CAG:0.20759501,(gi|769170695|ref|WP_044942941.1|_485_bp:0.05940388,Bact_ClostridiaNewRepAgi|545388984|ref|WP_021629801.1|/1321Bacteria_Firmicutes_Clostridia_Clostridia:0.03780180):0.12981220[1.000000]):0.17079497[0.999999]):0.07970284[0.985708]):0.09469470[0.996650]):0.07925113[0.945005]):0.20580147[1.000000]):0.01783301[0.902276],(Bact_RuminococcNewRepAgi|916939772|ref|WP_051546484.1|/1338Bacteria_Firmicutes_Clostridia_Clostridia:0.63786116,(gi|1054783115|ref|WP_066546553.1|_485_bp:0.17291675,Bact_LachnospirNewRepAgi|503036515|ref|WP_013271491.1|/1353Bacteria_Firmicutes_Clostridia_Clostridia:0.20277610):0.67032604[1.000000]):0.12904094[0.999530]):0.05071217[0.989515]):0.10618733[0.999721]):0.09722347[0.995945],(Gr3_4bRCRep4no_r_no_rankgi|738376067|ref|WP_036328238.1|/1301no_rank_485_bp:0.65126946,(gi|1052781588|emb|SCH60086.1|_485_bp:0.46376666,(YP_007517186.1_485_bp:0.14821678,YP_009126903.1_485_bp:0.13462702):0.99677956[1.000000]):0.16530575[0.988440]):0.10469574[0.521910]):0.15902946[0.999525],(((Bact_MicrobacteNewRepAgi|946916017|ref|WP_055838650.1|/1411Bacteria_Actinobacteria_Actinobacteria_Mi:0.71787631,(Gr3_4bRCRep3no_r_no_rankgi|738390844|ref|WP_036342632.1|/1318no_rank_485_bp:0.74977969,(Bact_PropionibaNewRepAgi|514978936|ref|WP_016667133.1|/1293Bacteria_Actinobacteria_Actinobacteria_Pr:0.61036425,Gr3_4bRCRep3Bact_Propionibagi|488485987|ref|WP_002529618.1|/1309Bacteria_Actinobacteria_Actinobacter:0.44524327):0.09811753[0.612713]):0.26839236[1.000000]):0.17828583[0.992156],(((bRCRep3Gr3_4Bact_Bifidobactgi|547239181|ref|WP_021975256.1|/1330Bacteria_Actinobacteria_Actinobacter:0.36024958,(Bact_BifidobactNewRepAgi|919428384|ref|WP_052825216.1|/1368Bacteria_Actinobacteria_Actinobacteria_Bi:0.25635225,(Bact_BifidobactNewRepAgi|672986434|gb|KFI81686.1|/1310Bacteria_Actinobacteria_Actinobacteria_Bifidob:0.18662802,Gr3_4bRCRep3Bact_Bifidobactgi|759448466|ref|WP_043170238.1|/1343Bacteria_Actinobacteria_Actinobacter:0.13709472):0.14956140[1.000000]):0.11370048[0.999855]):0.20057274[1.000000],(Bact_BifidobactNewRepAgi|917512920|ref|WP_052119337.1|/1380Bacteria_Actinobacteria_Actinobacteria_Bi:0.95487107,Bact_BifidobactNewRepAgi|672992267|gb|KFI87454.1|/1362Bacteria_Actinobacteria_Actinobacteria_Bifidob:0.59273776):0.15477169[0.984458]):0.08325885[0.909454],(Bact_BifidobactNewRepAgi|643502474|ref|WP_025221073.1|/1397Bacteria_Actinobacteria_Actinobacteria_Bi:0.86517148,(bRCRep3Gr3_4Bact_Bifidobactgi|20069877|ref|NP_613078.1|/1341Bacteria_Actinobacteria_Actinobacteria_B:0.70832399,(Bact_CorynebactNewRepAgi|552777527|ref|WP_023022037.1|/1304Bacteria_Actinobacteria_Actinobacteria_Co:0.46317093,Bact_PasteurellNewRepAgi|857119895|gb|AKO38848.1|/1328Bacteria_Proteobacteria_Gammaproteobacteria_Pa:0.84596796):0.27247157[0.999999]):0.06743643[0.716996]):0.11875917[0.994478]):0.10295337[0.991044]):0.13144572[0.983034],(bRCRep3Gr3_4Bact_Bifidobactgi|551236367|ref|WP_022856850.1|/1352Bacteria_Actinobacteria_Actinobacter:0.65279189,Bact_BifidobactNewRepAgi|705399483|ref|WP_033495900.1|/1445Bacteria_Actinobacteria_Actinobacteria_Bi:1.23715975):0.17187019[0.859076]):0.34381413[1.000000]):0.15274990[0.999984]):0.36676718[1.000000],((((AIF34798.1|Sewageassociated_485_bp:0.60322844,(((AKO71308.1|Banana_485_bp:0.00000033,JF957636|Nanovirus_1..861_485_bp:0.01436652):0.37830680[1.000000],(YP_003104737.1|Faba_485_bp:0.01922473,HE654123|Nanovirus_1..861_485_bp:0.06782764):0.31214766[1.000000]):0.45365729[1.000000],(((HM163578|Alpha_1..945_485_bp:0.03860879,(YP_008169853.1|Cuban_485_bp:0.07764808,(YP_009246456.1|Cucurbit_485_bp:0.11888682,ALK03646.1|Alphasatellite_485_bp:0.14892941):0.08393656[1.000000]):0.03329166[0.853530]):0.73955906[1.000000],(NP_619759.1|Milk_485_bp:0.37344708,(KF471057|Alpha_1..948_485_bp:0.45623082,JX458742|Alpha_1..924_485_bp:0.33146294):0.24444275[1.000000]):0.07371192[0.708336]):0.03052210[0.496926],((AAA51426.1|Banana_485_bp:0.20736815,(AAA51422.1|Banana_485_bp:0.05286605,ACB86656.1|Banana_485_bp:0.26610593):0.28569829[1.000000]):0.29873248[1.000000],((U16735|Alpha_1..858_485_bp:0.14281756,KC979052|Alpha_1..855_485_bp:0.20043956):0.26106253[1.000000],(NP_619760.1|Milk_485_bp:0.22690532,(YP_009058890.1|Faba_485_bp:0.06000169,KC978991|Alpha_1..837_485_bp:0.03931365):0.21891175[1.000000]):0.26080240[1.000000]):0.05581160[0.869427]):0.07971151[0.996327]):0.29504034[1.000000]):0.27603006[1.000000]):0.32204042[1.000000],(NewKX388505.1|complement_1757..3067__485_bp:0.80726816,(((YP_009021888.1|Cyanoramphus_485_bp:0.52218613,YP_009237559.1|Lake_485_bp:0.65158010):0.25381692[1.000000],AHH31482.1|Dragonfly_485_bp:0.36943790):0.10025693[0.713582],YP_009163936.1|Palaemonetes_485_bp:0.50264367):0.21947489[0.999993]):0.17295799[0.999511]):0.08128265[0.772147],((((JX904231|CRESS_unclass_1..1122_485_bp:0.50935320,(KT945163|CRESS_unclass_1..909_485_bp:0.65355784,(KR528545|CRESS_unclass_1..909_485_bp:0.43226778,KR528553|CRESS_unclass_1..918_485_bp:0.58063729):0.07207067[0.968570]):0.08547019[0.936389]):0.13888135[0.999944],((KM874354|CRESS_unclass_1..915_485_bp:0.45110270,((KR528554|CRESS_unclass_1..948_485_bp:0.29690673,KR528556|CRESS_unclass_1..915_485_bp:0.28378951):0.13964872[0.999877],(KR528551|CRESS_unclass_1..906_485_bp:0.40076877,KR528562|CRESS_unclass_1..906_485_bp:0.45353591):0.17667348[1.000000]):0.08775651[0.869370]):0.10333454[0.972438],(KJ641738|CRESS_unclass_1..960_485_bp:0.55905792,(KR528547|CRESS_unclass_1..870_485_bp:0.62039298,KR528561|CRESS_unclass_1..885_485_bp:0.42642113):0.04799952[0.877304]):0.08788518[0.982276]):0.04688568[0.478177]):0.18159057[1.000000],(KJ547646|CRESS_unclass_1..891_485_bp:0.66135913,(KP153451|CRESS_unclass_1..951_485_bp:0.63002353,KJ547650|CRESS_unclass_1..924_485_bp:0.52144445):0.13675895[0.863206]):0.15953713[0.999958]):0.09231954[0.752518],(((AIY31256.1|Dromedary_485_bp:0.31304820,(AIY31243.1|Dromedary_485_bp:0.15430081,KT862224|Smacovirus_1..816_485_bp:0.31873992):0.10450992[0.975322]):0.65863562[1.000000],(AJD07511.1|Odonataassociated_485_bp:0.64863921,(YP_009252316.1|Bovine_485_bp:0.63923032,(((AJE25851.1|Human_485_bp:0.14635311,AJE25845.1|Human_485_bp:0.05758130):0.11973600[0.999999],(KY086301|Smacovirus_1..747_485_bp:0.07627158,(AJF23062.1|Human_485_bp:0.06724214,(AJF23060.1|Human_485_bp:0.00780220,(AJE25847.1|Human_485_bp:0.00867788,KP233175|Smacovirus_1..750_485_bp:0.00000030):0.15962714[1.000000]):0.07919546[1.000000]):0.14483083[1.000000]):0.08588769[0.724981]):0.41530263[1.000000],(YP_009252326.1|Bovine_485_bp:0.45809994,(KU203352|CRESS_unclass_1..810_485_bp:0.17952440,KJ547633|CRESS_unclass_1..780_485_bp:0.15531307):1.08457210[1.000000]):0.17120914[0.903188]):0.07197929[0.785988]):0.18182357[0.977764]):0.29052539[0.999939]):0.44379268[0.999987],((KM598409|Smacovirus_1..921_485_bp:0.53403208,(AEW47007.1|Circoviridae_485_bp:0.29158959,YP_009252314.1|Bovine_485_bp:0.32458784):0.27755750[0.990039]):1.01541030[1.000000],((((KU043420|CRESS_unclass_1..852_485_bp:0.63632571,(KP233189|Smacovirus_1..699_485_bp:0.20090957,(YP_009022025.1|Turkey_485_bp:0.17518715,((YP_009030025.1|PoSCV_485_bp:0.02781278,(KJ577810|Smacovirus_1..732_485_bp:0.01273982,YP_009054985.1|Porcine_485_bp:0.11040908):0.02355124[0.989081]):0.07450607[1.000000],(KX838318|Smacovirus_1..819_485_bp:0.06048580,(AMR73073.1|Human_485_bp:0.05621025,KX838317|Smacovirus_1..789_485_bp:0.10875971):0.08288259[0.999997]):0.17202132[1.000000]):0.02475459[0.533518]):0.08301342[0.999995]):0.08185536[0.994678]):0.15196195[0.999998],((AIY31246.1|Dromedary_485_bp:0.52244896,((YP_009163761.1|Rat_485_bp:0.26898288,YP_009054993.1|Porcine_485_bp:0.15514117):0.11690490[0.998741],(YP_009118278.1|Lemur_485_bp:0.63128028,(KU043403|CRESS_unclass_1..819_485_bp:0.36723534,(AMR73071.1|Human_485_bp:0.29023803,(ADB24799.1|Chimpanzee_485_bp:0.00000110,GQ351275|Smacovirus_1..816_485_bp:0.03546096):0.38571634[1.000000]):0.08949457[0.946067]):0.07875959[0.923578]):0.11993708[0.992016]):0.15739538[1.000000]):0.08295174[0.848245],(YP_009252320.1|Porcine_485_bp:0.51657558,(((YP_009054987.1|Porcine_485_bp:0.00000036,KJ577813|Smacovirus_1..789_485_bp:0.00400571):0.09799727[1.000000],(YP_009118276.1|Gorilla_485_bp:0.10762338,KU043428|CRESS_unclass_1..840_485_bp:0.08971801):0.08049523[0.999944]):0.23507137[1.000000],(KU043422|CRESS_unclass_1..777_485_bp:0.59731017,(KU043430|CRESS_unclass_1..867_485_bp:0.24104691,KU058671|CRESS_unclass_1..804_485_bp:0.35615527):0.18635774[1.000000]):0.08219646[0.990106]):0.05945517[0.924729]):0.08413082[0.999550]):0.09781565[0.999967]):0.14467298[1.000000],(YP_009252308.1|Sheep_485_bp:0.79245516,KY086298|Smacovirus_1..777_485_bp:0.35413299):0.04489846[0.905995]):0.17009931[0.997493],(YP_009252310.1|Sheep_485_bp:0.71033862,((KM573775|Smacovirus_1..762_485_bp:0.20579085,KM573771|Smacovirus_1..735_485_bp:0.23315748):0.24492185[1.000000],(KT862221|Smacovirus_1..783_485_bp:0.13913185,(AIY31250.1|Dromedary_485_bp:0.28599666,KT862218|Smacovirus_1..735_485_bp:0.03600083):0.14465860[1.000000]):0.19650732[1.000000]):0.17609840[0.999924]):0.09064396[0.522734]):0.33823024[0.999270]):0.38696648[0.979489]):0.82471782[1.000000]):0.06395513[0.652818]):0.49073460[1.000000],((((KT732825|CRESS_unclass_1..837_485_bp:0.80743501,((YP_009000900.1|Anguilla_485_bp:0.48045436,((YP_009091696.1|Silurus_485_bp:0.38707736,(ADD62475.1|Human_485_bp:0.34120784,YP_004376332.1|Barbel_485_bp:0.44739938):0.16149309[1.000000]):0.06492464[0.982166],((YP_009170674.1|Tadarida_485_bp:0.51580233,((ABU48445.1|Swan_485_bp:0.12031496,AHK80894.1|Duck_485_bp:0.13993940):0.43305130[1.000000],(YP_803546.1|Gull_485_bp:0.30228376,((ADU77009.1|Circovirus_485_bp:0.14293312,((YP_764455.1|Raven_485_bp:0.10962582,NP_573442.1|Canary_485_bp:0.16437903):0.03946785[0.999657],(KU230452|CRESS_unclass_1..873_485_bp:0.07993590,YP_009134739.1|Zebra_485_bp:0.15485395):0.02043248[0.985970]):0.07270741[0.999999]):0.12855525[1.000000],(AFL02442.1|Beak_485_bp:0.36461186,AEL28794.1|Bat_485_bp:0.32408282):0.06191845[0.853017]):0.05050392[0.528972]):0.19554859[1.000000]):0.11367787[0.967127]):0.09256847[0.997450],((AIF76261.1|Bat_485_bp:0.26605031,(AIF76248.1|Bat_485_bp:0.13250292,(AIF76265.1|Bat_485_bp:0.14353501,AIF76253.1|Bat_485_bp:0.16667986):0.05974321[0.985313]):0.12120753[0.999956]):0.34296503[1.000000],((AGL09969.1|Bat_485_bp:0.13110344,YP_009021891.1|Mink_485_bp:0.10802022):0.27244349[1.000000],(AKO84203.1|Fox_485_bp:0.39314645,((AIF76280.1|Bat_485_bp:0.01193137,KJ641742|CRESS_unclass_1..948_485_bp:0.01528363):0.23577971[1.000000],(YP_007974237.1|Bat_485_bp:0.18355777,AAZ78351.1|Porcine_485_bp:0.20981971):0.07926130[0.998292]):0.34825876[1.000000]):0.10530932[0.970346]):0.11222419[0.995538]):0.03556735[0.512286]):0.10203084[0.999834]):0.09323748[0.998668]):0.09340328[0.999768],(AIF76251.1|Bat_485_bp:0.73964853,(((AMH87650.1|Pacific_485_bp:0.09734448,AMH87652.1|Pacific_485_bp:0.19720849):0.72020750[1.000000],((ADU76993.1|Cyclovirus_485_bp:0.18758943,(ADD62477.1|Cyclovirus_485_bp:0.11878890,(YP_009047065.1|Cyclovirus_485_bp:0.16149701,YP_008130363.1|Human_485_bp:0.16711465):0.06227324[0.953007]):0.05035197[0.981996]):0.30429823[1.000000],(AIF76254.1|Bat_485_bp:0.29620825,((AIF76249.1|Bat_485_bp:0.25206802,ADD62453.1|Cyclovirus_485_bp:0.23381092):0.06812359[0.999923],(((YP_009021843.1|Dragonfly_485_bp:0.26764112,AKE49355.1|Cyclovirus_485_bp:0.18399611):0.07684446[0.999996],((YP_004152331.1|Cyclovirus_485_bp:0.21212281,(ADD62471.1|Cyclovirus_485_bp:0.13430215,AIF76252.1|Bat_485_bp:0.12354978):0.07315133[1.000000]):0.04270915[0.999998],(AEL87790.1|Bat_485_bp:0.18137856,(AEL87786.1|Bat_485_bp:0.21054955,ADI48251.1|Bat_485_bp:0.19117349):0.06124173[0.999904]):0.05294765[0.995848]):0.03861255[0.991141]):0.01729613[0.850578],((ADY17982.1|Dragonfly_485_bp:0.29105691,(AGJ74760.1|Dragonfly_485_bp:0.19070976,(YP_009021870.1|Human_485_bp:0.26169254,AFS65290.1|Dragonfly_485_bp:0.26206448):0.08909829[0.999997]):0.03293772[0.792416]):0.06924764[0.999999],(((ADD62451.1|Cyclovirus_485_bp:0.13250320,ADD62455.1|Cyclovirus_485_bp:0.15886373):0.04353322[0.995726],(ADD62461.1|Cyclovirus_485_bp:0.23418229,(ADU77011.1|Cyclovirus_485_bp:0.14779056,AGJ74758.1|Dragonfly_485_bp:0.22057822):0.05399659[0.997814]):0.03885197[0.774587]):0.08444897[1.000000],((YP_009110680.1|Bat_485_bp:0.14666298,AGJ74756.1|Dragonfly_485_bp:0.27035575):0.05303542[0.999959],(ADD62457.1|Cyclovirus_485_bp:0.22244536,(AEL87792.1|Bat_485_bp:0.21684515,(AIF76266.1|Bat_485_bp:0.21269859,ADD62473.1|Cyclovirus_485_bp:0.09257451):0.08929706[1.000000]):0.08470905[1.000000]):0.03389844[0.925272]):0.05083161[0.999510]):0.02057649[0.786371]):0.05031479[0.999935]):0.04140808[0.981390]):0.06785329[0.995565]):0.16164158[1.000000]):0.12399702[0.999928]):0.05325371[0.806467],(YP_009237526.1|Lake_485_bp:0.52035096,YP_009116910.1|Sewageassociated_485_bp:0.58760765):0.11134616[0.995463]):0.05813374[0.964009]):0.11578217[0.999498]):0.13712015[0.999143]):0.19689080[1.000000],(((KM874309|CRESS_unclass_1..957_485_bp:0.67276899,KF133822|CRESS_unclass_1..873_485_bp:0.58739763):0.18753281[0.999944],(((KT149404|CRESS_unclass_1..975_485_bp:0.85090878,KM874347|CRESS_unclass_1..912_485_bp:0.63796900):0.06982054[0.508170],FJ959078|CRESS_unclass_1..1230_485_bp:0.69221040):0.14756893[0.996693],(KP153497|CRESS_unclass_1..963_485_bp:0.90115391,(NewKX388513.1|complement_1908..3059__485_bp:0.06754576,NewKX388515.1|complement_1325..2740__485_bp:0.00000111):1.27758858[1.000000]):0.18876202[0.958283]):0.14134138[0.990599]):0.12029656[0.975317],((b81c81ec2fabc56fb906dfdae75b4597250b6618c2a9e5d7d592074574688346-01_485_bp:0.34496947,((AUF34977.1_485_bp:0.52915813,(((9e1d9b352c6c57b8bf4c20295d58383736f38c02650e268d4687499f14df912e-00_485_bp:0.22666242,(41834aaa2f2e36cb0531dc8dada4b7084d7cf50a8b65d6fd756c9b40bd7d5721-00_485_bp:0.29651255,(0bd68b5a40c5a6044c3ff40ca9815e30c548f9eeb6ef87aca3f144ce65cf7441-00_485_bp:0.18311575,YP_009345097.1_485_bp:0.25908677):0.25977852[1.000000]):0.06197325[0.948613]):0.07448017[0.999993],(36ff03fbe1a9a1060a16d972e21b61fe3bbf57259b2dabec48e9b740ac32cdb4-00_485_bp:0.34209674,fb8f6287d1d0a95e973165257530b7322c29b1a18a92f17aa84d58d4e9a32e97-00_485_bp:0.35523638):0.05315470[0.500555]):0.09187736[0.999999],(((5bc306a9607207a0efbbea55c33a7c25fdc354f8e2e77d7f1d8398e102b42c13-10_485_bp:0.26223073,(65c9a819eeecc58815473d6730a3a9d710279d3bcb36cbe602dbb43ca9714427-00_485_bp:0.16598922,YP_009109635.1_485_bp:0.19125491):0.15963678[1.000000]):0.08367154[0.998229],((YP_009345107.1_485_bp:0.17844287,(2851f14acec255faf0601dc5f18e5a234ecb640e1516df67be746e6316b0d50f-00_485_bp:0.21391339,YP_009345086.1_485_bp:0.16520118):0.09882801[1.000000]):0.10814909[1.000000],((YP_004046698.1|Chaetoceros_485_bp:0.10954883,(YP_009111348.1_485_bp:0.04426598,BAP99817.1_485_bp:0.04043002):0.05922183[0.997443]):0.28150010[1.000000],(YP_473359.1_485_bp:0.21282284,((YP_009001777.1_485_bp:0.15782271,a43e97046e8aad84b088372367f8909fe0bea7fa95b4e398e7eaa90494b94205-10_485_bp:0.16512702):0.13330056[1.000000],(BAL05205.1_485_bp:0.23355798,YP_004286322.1_485_bp:0.24844868):0.07762131[0.999865]):0.06994336[0.999073]):0.13560225[1.000000]):0.11462347[1.000000]):0.07746272[0.999839]):0.04765369[0.965993],((827d7105bece8830299875f2b521c58893e8baa105c1d682254ad2968e2d9817-00_485_bp:0.18049876,fc270c7af120b5db22a92e67a52615ba84787c103ed6948ced3f64c154ae819d-00_485_bp:0.26135465):0.18791373[1.000000],(51dc4f0b8ef1f29067aa5edd136201ee8177aa724a7e9096a2a83f9aea624d2a-00_485_bp:0.27363655,(282ad189c388a58a159bc47ec08d617866074d3026c12ef4c7ce1fa861ba629f-00_485_bp:0.15897770,(bd4c33c6592559bdffb711a3ce94a3264224e9910e589e1eba5213be9394799d-00_485_bp:0.24909579,e297896c0a2982c2684f01eb18de03f161b1701381986ca875d14abb8d518137-01_485_bp:0.15422804):0.18204685[1.000000]):0.06395606[0.994725]):0.04144259[0.998015]):0.07377273[0.999997]):0.09507944[0.992847]):0.08068070[0.962594]):0.06266191[0.985513],(BAN59850.1|Thalassionema_485_bp:0.63189378,833ee4171fb5d86e62b3fe0f3c1e026947ca78ff9113d1c474570a16e65ae785-10_485_bp:0.62421562):0.13789777[0.991165]):0.12603027[0.831151]):1.43131592[1.000000],((KU043424|CRESS_unclass_1..1056_485_bp:0.59863429,(KM573766|CRESS_unclass_1..1035_485_bp:0.67725902,(KT862256|CRESS_unclass_1..1206_485_bp:0.25722295,KF246569|CRESS_unclass_1..1077_485_bp:0.23464392):0.58983380[1.000000]):0.13134346[0.995827]):0.06838195[0.622267],(KJ206566|CRESS_unclass_1..1113_485_bp:0.41134615,KU043411|CRESS_unclass_1..1104_485_bp:0.30255625):0.26694500[1.000000]):0.61057705[1.000000]):0.14197071[0.742953]):0.14108764[0.999670]):0.12370786[1.000000],((KT149409|CRESS_unclass_1..873_485_bp:0.67798643,(KM598406|CRESS_unclass_1..816_485_bp:0.52290701,(KT149403|CRESS_unclass_1..945_485_bp:0.75216785,((JX904581|CRESS_unclass_1..792_485_bp:0.50563715,((KM874317|CRESS_unclass_1..822_485_bp:0.42920333,(KP153422|CRESS_unclass_1..876_485_bp:0.47617967,KP153408|CRESS_unclass_1..816_485_bp:0.30751373):0.06904282[0.969255]):0.06011242[0.858651],(KM874300|CRESS_unclass_1..777_485_bp:0.73116233,KM874304|CRESS_unclass_1..798_485_bp:0.35320932):0.06194432[0.695979]):0.04393621[0.889566]):0.08374813[0.996549],(JX904407|CRESS_unclass_1..990_485_bp:0.79955381,(JX904139|CRESS_unclass_1..822_485_bp:0.27689401,(JX904075|CRESS_unclass_1..834_485_bp:0.32991142,JX904076|CRESS_unclass_1..819_485_bp:0.28273295):0.07396515[0.999945]):0.13063990[0.999219]):0.13701032[0.999856]):0.09690528[0.999123]):0.05444684[0.996903]):0.05602802[0.825758]):0.05281166[0.957858],((JX185418|CRESS_unclass_1..870_485_bp:0.48199350,KM598404|CRESS_unclass_1..885_485_bp:0.74370679):0.17305170[0.999041],(KJ641729|CRESS_unclass_1..936_485_bp:0.79929280,((KJ641722|CRESS_unclass_1..804_485_bp:0.14927383,HM228875|CRESS_unclass_1..795_485_bp:0.13545340):0.50175142[1.000000],((JN857329|CRESS_unclass_1..930_485_bp:0.40104729,KJ641718|CRESS_unclass_1..864_485_bp:0.40524091):0.15759964[0.999982],(KF738883|CRESS_unclass_1..864_485_bp:0.63094576,KM972726|CRESS_unclass_1..906_485_bp:0.46402114):0.09637070[0.974998]):0.04207815[0.597849]):0.09755242[0.983568]):0.04642934[0.430324]):0.11547053[0.999999]):0.16542490[1.000000]):0.02606725[0.441507],(((KP153377|CRESS_unclass_1..861_485_bp:0.60749739,(KT732819|CRESS_unclass_1..828_485_bp:0.63694885,((KM573776|CRESS_unclass_1..1008_485_bp:0.25824582,KM573767|CRESS_unclass_1..1002_485_bp:0.31601465):0.21287531[1.000000],(KU043397|CRESS_unclass_1..1200_485_bp:0.49042080,KU043406|CRESS_unclass_1..996_485_bp:0.41319267):0.13148962[0.990060]):0.23246667[1.000000]):0.11569047[0.994694]):0.08832582[0.880346],(KF738877|CRESS_unclass_1..888_485_bp:0.97651145,((JX904420|CRESS_unclass_1..840_485_bp:0.57171232,((JX904344|CRESS_unclass_1..819_485_bp:0.40414481,(KP153364|CRESS_unclass_1..813_485_bp:0.44469423,JX904185|CRESS_unclass_1..813_485_bp:0.32293639):0.21376516[1.000000]):0.07368596[0.983626],(KM821755|CRESS_unclass_1..870_485_bp:0.60736151,(KT732816|CRESS_unclass_1..849_485_bp:0.28730570,JF755415|CRESS_unclass_1..735_485_bp:0.19999279):0.26550277[1.000000]):0.09896255[0.948451]):0.03215352[0.831576]):0.04208219[0.997943],(((KT149394|CRESS_unclass_1..846_485_bp:0.45513053,KP153360|CRESS_unclass_1..840_485_bp:0.29424641):0.51274729[1.000000],(FJ959082|CRESS_unclass_1..1032_485_bp:0.59863881,(KP153447|CRESS_unclass_1..858_485_bp:0.49451351,(((KP153485|CRESS_unclass_1..855_485_bp:0.24439774,(KJ547648|CRESS_unclass_1..840_485_bp:0.15909228,KT149412|CRESS_unclass_1..831_485_bp:0.28375785):0.07415467[0.953743]):0.47740202[1.000000],(KT149398|CRESS_unclass_1..846_485_bp:0.65228730,(KP153404|CRESS_unclass_1..903_485_bp:0.47800877,KC248416|CRESS_unclass_1..831_485_bp:0.33820616):0.10944152[0.999238]):0.06067674[0.943121]):0.11297020[0.999123],(JX185415|CRESS_unclass_1..999_485_bp:0.41700652,(KM598396|CRESS_unclass_1..1035_485_bp:0.36253412,KT732823|CRESS_unclass_1..1089_485_bp:1.02235078):0.22485086[0.927883]):0.44350227[1.000000]):0.05408915[0.564382]):0.08686397[0.974240]):0.11606918[0.997420]):0.03971613[0.706079],(KP153483|CRESS_unclass_1..1140_485_bp:0.82666618,(KM821764|CRESS_unclass_1..1584_485_bp:0.70306093,(KP153369|CRESS_unclass_1..882_485_bp:0.72342546,KP153468|CRESS_unclass_1..1167_485_bp:0.71836204):0.06631934[0.553211]):0.06763544[0.668315]):0.16052681[0.999305]):0.05506567[0.961671]):0.09073085[0.999858]):0.05287244[0.790568]):0.04357719[0.643695],(JX904107|CRESS_unclass_1..1044_485_bp:0.53926303,JX904562|CRESS_unclass_1..888_485_bp:0.37415371):0.29429850[1.000000]):0.53060356[1.000000]):0.12481279[0.818880]):0.18598884[0.999967]):0.19854457[0.972502]):0.43223084[1.000000]):0.79188774[1.000000]):0.10530809[0.515226]):0.07091548[0.956316]):0.11294640[0.999999]):0.11427805[0.968097]):0.44624987[1.000000])OROOT;
