# Supplementary material for: Multiple origins of prokaryotic and eukaryotic single-stranded DNA viruses from bacterial and archaeal plasmids
Source: Nat Commun. 2019 Jul 31;10:3425. doi: 10.1038/s41467-019-11433-0 (PMC6668415; doi:10.1038/s41467-019-11433-0)
Supplement: Supplementary file 11 — Dataset 10 [file 41467_2019_11433_MOESM11_ESM.docx]

**SUPPLEMENTARY DATA 10**

# PHYLOGENETIC TREE SHOWN IN FIGURE S6B

(Bact_ActinomyceNewRepAgi|493732575|ref|WP_006681830.1|/1701Bacteria_Actinobacteria_Actinobacteria_Ac:0.00000033,gi|917432205|ref|WP_052038917.1|_467_bp:0.00920373,(gi|765006636|ref|WP_044572803.1|_467_bp:0.59651496,((Bact_DermabacteNewRepAgi|516435276|ref|WP_017824301.1|/1555Bacteria_Actinobacteria_Actinobacteria_Mi:0.19117796,Bact_PropionibaNewRepAgi|808425327|emb|CEI31812.1|/1673Bacteria_Actinobacteria_Actinobacteria_Propio:0.26709777):0.36913750[1.000000],((gi|453365874|dbj|GAC78794.1|_467_bp:0.32565776,(gi|759838613|ref|WP_043534193.1|_RNA_helicase_Actinomyces_sp._MS2_467_bp:0.09290549,(gi|1056502811|ref|WP_067940518.1|_RNA_helicase_Actinomyces_radicidentis_467_bp:0.04795832,gi|491607164|ref|WP_005464724.1|_467_bp:0.00337384):0.03208198[0.997044]):0.22026923[1.000000]):0.44933715[1.000000],((no_r_no_rankNewRepAgi|874569067|emb|CRY97508.1|/1564uncultured_prokaryote_467_bp:0.64997500,((Bact_ClostridiaNewRepAgi|545399497|ref|WP_021639163.1|/1525Bacteria_Firmicutes_Clostridia_Clostridia:0.41961308,(Bact_no_rankNewRepAgi|291561635|emb|CBL40434.1|/1453Bacteria_Firmicutes_Clostridia_Clostridiales/145:0.53580401,no_r_no_rankNewRepAgi|874572962|emb|CRY93789.1|/1462uncultured_prokaryote_467_bp:0.29562190):0.06528376[0.677568]):0.06841018[0.976968],(Bact_LachnospirNewRepAgi|495165195|ref|WP_007889993.1|/1493Bacteria_Firmicutes_Clostridia_Clostridia:0.64079048,Bact_RuminococcNewRepAgi|524256822|emb|CDA18875.1|/1527Bacteria_Firmicutes_Clostridia_Clostridiales_:0.63397766):0.11183274[0.923627]):0.10325882[0.999940]):0.10433887[0.998651],((Bact_StreptococNewRepAgi|446741101|ref|WP_000818357.1|/1455Bacteria_Firmicutes_Bacilli_Lactobacillal:0.39262637,Bact_StreptococNewRepAgi|446108339|ref|WP_000186194.1|/1488Bacteria_Firmicutes_Bacilli_Lactobacillal:0.47177624):0.13631512[0.997609],((UniRef50_R5VXD3_467_bp:0.93210018,((UniRef50_W1I5Y6_467_bp:1.03512228,((gi|1008240811|ref|WP_061866456.1|_467_bp:0.63292341,((gi|636907128|ref|WP_024393234.1|_467_bp:0.11369711,gi|912699815|ref|WP_050238550.1|_467_bp:0.12921420):0.46152354[1.000000],(gi|636897466|ref|WP_024390948.1|_467_bp:0.20260417,gi|656228629|ref|WP_029176301.1|_467_bp:0.16449655):0.46429883[1.000000]):0.12964806[0.796835]):0.37717702[1.000000],(((gi|504548170|ref|WP_014735272.1|_467_bp:0.00479283,(gi|928783372|ref|WP_053863690.1|_hypothetical_protein_Streptococcus_suis_467_bp:0.01265369,((gi|636870655|ref|WP_024382134.1|_467_bp:0.00000015,gi|636890832|ref|WP_024389873.1|_467_bp:0.01088577):0.00756671[0.999963],(gi|636937158|ref|WP_024399566.1|_467_bp:0.02046968,gi|516919872|ref|WP_018166163.1|_467_bp:0.02011973):0.00967343[0.955500]):0.02727663[1.000000]):0.00232765[0.485411]):0.30184874[1.000000],((gi|896656789|ref|WP_049535277.1|_hypothetical_protein_Streptococcus_pseudopneumoniae_467_bp:0.01577825,(gi|896594168|ref|WP_049478725.1|_467_bp:0.03090840,(gi|1002966178|ref|WP_061417941.1|_hypothetical_protein_Streptococcus_oralis_467_bp:0.00917859,gi|1008212779|ref|WP_061863770.1|_467_bp:0.02238363):0.01081786[0.996413]):0.01694458[0.992335]):0.07721052[1.000000],(gi|1055542165|ref|WP_067193806.1|_hypothetical_protein_Streptococcus_sp._DD10_467_bp:0.10738551,(Bact_StreptococNewRepAgi|766587633|ref|WP_044771983.1|/1450Bacteria_Firmicutes_Bacilli_Lactobacillal:0.08031188,(gi|656223019|ref|WP_029171254.1|_hypothetical_protein_Streptococcus_suis_467_bp:0.08279717,(gi|746742088|ref|WP_039694423.1|_hypothetical_protein_Streptococcus_gallolyticus_467_bp:0.04205919,(Bact_StreptococNewRepAgi|636994187|ref|WP_024408358.1|/1452Bacteria_Firmicutes_Bacilli_Lactobacillal:0.04391197,(Bact_StreptococNewRepAgi|726979138|ref|WP_033583888.1|/1452Bacteria_Firmicutes_Bacilli_Lactobacillal:0.00209379,gi|896597713|ref|WP_049481849.1|_467_bp:0.00210836):0.10356039[1.000000]):0.03675501[1.000000]):0.02030291[0.954827]):0.03141905[0.999951]):0.11020603[1.000000]):0.03210550[0.587050]):0.20130132[1.000000]):0.59648923[1.000000],((gi|960289695|ref|WP_058211405.1|_467_bp:0.30126158,gi|515938083|ref|WP_017368666.1|_hypothetical_protein_Lactococcus_garvieae_467_bp:0.22721372):0.55636391[1.000000],(gi|742672785|ref|WP_038978316.1|_467_bp:0.46589735,gi|815846062|ref|WP_046467524.1|_467_bp:0.41935313):0.40604632[1.000000]):0.11083273[0.633078]):0.14154778[0.916507]):0.23710117[0.998605]):0.29318740[0.998192],((((Bact_StreptococNewRepAgi|504384690|ref|WP_014571792.1|/1433Bacteria_Firmicutes_Bacilli_Lactobacillal:0.70613738,Gr1bRCRep1Bact_Streptococgi|358750970|gb|AEU41945.1|/1508Bacteria_Firmicutes_Bacilli_Lactobacillales:0.61448832):0.08928291[0.652298],(((Bact_LactobacilNewRepAgi|949495867|ref|WP_056938517.1|/1444Bacteria_Firmicutes_Bacilli_Lactobacillal:0.37957180,(Bact_StreptococNewRepAgi|323126431|gb|ADX23728.1|/1431Bacteria_Firmicutes_Bacilli_Lactobacillales_St:0.38336905,((Bact_EnterococcNewRepAgi|736699323|ref|WP_034704841.1|/1450Bacteria_Firmicutes_Bacilli_Lactobacillal:0.05869233,gi|1055941163|ref|WP_067483596.1|_467_bp:0.05324341):0.16021365[1.000000],(Bact_StreptococNewRepAgi|489114682|ref|WP_003024533.1|/1461Bacteria_Firmicutes_Bacilli_Lactobacillal:0.20328375,((Bact_StreptococNewRepAgi|516245304|ref|WP_017649267.1|/1455Bacteria_Firmicutes_Bacilli_Lactobacillal:0.07832240,Bact_StreptococNewRepAgi|116108858|gb|ABJ73998.1|/1496Bacteria_Firmicutes_Bacilli_Lactobacillales_St:0.13184136):0.07690235[1.000000],((Bact_StreptococNewRepAgi|896589942|ref|WP_049476139.1|/1454Bacteria_Firmicutes_Bacilli_Lactobacillal:0.06816655,Bact_StreptococNewRepAgi|489125337|ref|WP_003035134.1|/1456Bacteria_Firmicutes_Bacilli_Lactobacillal:0.04280007):0.07944018[1.000000],((Bact_StreptococNewRepAgi|662350772|gb|KEQ49321.1|/1459Bacteria_Firmicutes_Bacilli_Lactobacillales_St:0.10483544,Bact_StreptococNewRepAgi|921142507|ref|WP_053092713.1|/1446Bacteria_Firmicutes_Bacilli_Lactobacillal:0.14614687):0.04081856[0.999993],((Bact_StreptococNewRepAgi|504436442|ref|WP_014623544.1|/1456Bacteria_Firmicutes_Bacilli_Lactobacillal:0.09037671,((Bact_StreptococNewRepAgi|695295662|ref|WP_032497992.1|/1452Bacteria_Firmicutes_Bacilli_Lactobacillal:0.15969558,Bact_StreptococNewRepAgi|1002507522|gb|KXT86702.1|/1458Bacteria_Firmicutes_Bacilli_Lactobacillales_S:0.05607179):0.02468188[0.983923],(Bact_StreptococNewRepAgi|505460283|ref|WP_015647385.1|/1455Bacteria_Firmicutes_Bacilli_Lactobacillal:0.14097436,Bact_StreptococNewRepAgi|873968101|emb|CGE81062.1|/1452Bacteria_Firmicutes_Bacilli_Lactobacillales_S:0.18120902):0.02360951[0.774438]):0.02168427[0.999455]):0.01507529[0.991419],((Bact_StreptococNewRepAgi|746719592|ref|WP_039677656.1|/1454Bacteria_Firmicutes_Bacilli_Lactobacillal:0.17892799,(Bact_StreptococNewRepAgi|896617134|ref|WP_049499636.1|/1457Bacteria_Firmicutes_Bacilli_Lactobacillal:0.08538657,Bact_StreptococNewRepAgi|787744000|ref|WP_045759092.1|/1457Bacteria_Firmicutes_Bacilli_Lactobacillal:0.06277251):0.10496825[1.000000]):0.05336974[1.000000],(Bact_StreptococNewRepAgi|654502558|ref|WP_027972054.1|/1457Bacteria_Firmicutes_Bacilli_Lactobacillal:0.12418998,(Bact_StreptococNewRepAgi|766591324|ref|WP_044774450.1|/1455Bacteria_Firmicutes_Bacilli_Lactobacillal:0.03603460,Bact_StreptococNewRepAgi|538448394|ref|WP_020997784.1|/1456Bacteria_Firmicutes_Bacilli_Lactobacillal:0.06725999):0.14451212[1.000000]):0.01849113[0.435193]):0.01993759[0.932264]):0.03375792[1.000000]):0.04803759[0.999999]):0.05449868[1.000000]):0.05604311[0.999984]):0.05326438[0.996822]):0.09986343[0.999998]):0.04632614[0.650679]):0.09590130[0.999986],((Bact_StreptococNewRepAgi|766570477|ref|WP_044762265.1|/1461Bacteria_Firmicutes_Bacilli_Lactobacillal:0.09204577,(Bact_StreptococNewRepAgi|517187727|ref|WP_018376545.1|/1459Bacteria_Firmicutes_Bacilli_Lactobacillal:0.07585247,Bact_StreptococNewRepAgi|538452261|ref|WP_020999261.1|/1459Bacteria_Firmicutes_Bacilli_Lactobacillal:0.11496756):0.03827294[0.722119]):0.37263886[1.000000],((Bact_StreptococNewRepAgi|445966413|ref|WP_000044268.1|/1463Bacteria_Firmicutes_Bacilli_Lactobacillal:0.19054893,(Bact_StreptococNewRepAgi|887581151|emb|CMU27730.1|/1468Bacteria_Firmicutes_Bacilli_Lactobacillales_S:0.17216856,(Bact_StreptococNewRepAgi|446123794|ref|WP_000201649.1|/1463Bacteria_Firmicutes_Bacilli_Lactobacillal:0.05381326,Bact_StreptococNewRepAgi|827365198|ref|WP_047206721.1|/1463Bacteria_Firmicutes_Bacilli_Lactobacillal:0.03297714):0.08353508[1.000000]):0.09953528[1.000000]):0.09429665[1.000000],((Bact_StreptococNewRepAgi|636940791|ref|WP_024400359.1|/1441Bacteria_Firmicutes_Bacilli_Lactobacillal:0.09277723,(Bact_StreptococNewRepAgi|490287353|ref|WP_004183001.1|/1456Bacteria_Firmicutes_Bacilli_Lactobacillal:0.05878807,(Bact_StreptococNewRepAgi|446957056|ref|WP_001034312.1|/1471Bacteria_Firmicutes_Bacilli_Lactobacillal:0.03438123,Bact_StreptococNewRepAgi|636879286|ref|WP_024385235.1|/1456Bacteria_Firmicutes_Bacilli_Lactobacillal:0.05179182):0.01150687[0.971501]):0.02194608[0.992500]):0.13767426[1.000000],(Bact_StreptococNewRepAgi|446714058|ref|WP_000791389.1|/1471Bacteria_Firmicutes_Bacilli_Lactobacillal:0.17079257,Bact_StreptococNewRepAgi|489122401|ref|WP_003032217.1|/1457Bacteria_Firmicutes_Bacilli_Lactobacillal:0.37842462):0.04545704[0.978070]):0.04718136[0.996286]):0.10109219[1.000000]):0.09869299[0.999998]):0.18140455[1.000000],((Bact_PropionibaNewRepAgi|916569613|ref|WP_051176704.1|/1584Bacteria_Actinobacteria_Actinobacteria_Pr:0.54165161,(Bact_MicrobacteNewRepAgi|738369377|ref|WP_036321578.1|/1492Bacteria_Actinobacteria_Actinobacteria_Mi:0.29532490,(gi|939719551|ref|WP_054952722.1|_467_bp:0.01466505,Bact_DermacoccaNewRepAgi|752621566|ref|WP_041290927.1|/1475Bacteria_Actinobacteria_Actinobacteria_Mi:0.03000385):0.13527609[1.000000]):0.23934771[1.000000]):0.16859417[0.999999],(Bact_LachnospirNewRepAgi|551021517|ref|WP_022765681.1|/1551Bacteria_Firmicutes_Clostridia_Clostridia:0.60202385,Bact_StreptococNewRepAgi|918462265|ref|WP_052506726.1|/1499Bacteria_Firmicutes_Bacilli_Lactobacillal:0.59424528):0.07606075[0.536977]):0.13331091[0.999315]):0.15277197[1.000000]):0.12157923[0.999758],((Bact_EnterococcNewRepAgi|1001889292|ref|WP_061343647.1|/1441Bacteria_Firmicutes_Bacilli_Lactobacilla:0.35739116,Bact_StreptococNewRepAgi|515940636|ref|WP_017371219.1|/1446Bacteria_Firmicutes_Bacilli_Lactobacillal:0.42877343):0.41328519[1.000000],(((gi|696366931|ref|WP_032941943.1|_467_bp:0.03082659,Bact_StreptococNewRepAgi|960317102|ref|WP_058223604.1|/1456Bacteria_Firmicutes_Bacilli_Lactobacillal:0.02244799):0.32724961[1.000000],(Bact_StreptococNewRepAgi|518129192|ref|WP_019299400.1|/1437Bacteria_Firmicutes_Bacilli_Lactobacillal:0.08926551,Bact_StreptococNewRepAgi|422001715|dbj|BAM66968.1|/1443Bacteria_Firmicutes_Bacilli_Lactobacillales_S:0.11446263):0.29421995[1.000000]):0.06692039[0.999546],((Bact_StreptococNewRepAgi|640587184|ref|WP_025016923.1|/1430Bacteria_Firmicutes_Bacilli_Lactobacillal:0.07507792,Bact_StreptococNewRepAgi|959256163|gb|KST89836.1|/1512Bacteria_Firmicutes_Bacilli_Lactobacillales_St:0.06398907):0.28991633[1.000000],((Bact_StreptococNewRepAgi|746711150|ref|WP_039670385.1|/1438Bacteria_Firmicutes_Bacilli_Lactobacillal:0.17214473,(Bact_StreptococNewRepAgi|516667150|ref|WP_018030886.1|/1436Bacteria_Firmicutes_Bacilli_Lactobacillal:0.12826540,Bact_StreptococNewRepAgi|489194916|ref|WP_003104234.1|/1448Bacteria_Firmicutes_Bacilli_Lactobacillal:0.09031499):0.11320695[1.000000]):0.25701718[1.000000],(((Bact_StreptococNewRepAgi|489138744|ref|WP_003048523.1|/1442Bacteria_Firmicutes_Bacilli_Lactobacillal:0.04259885,Bact_StreptococNewRepAgi|746742170|ref|WP_039694464.1|/1449Bacteria_Firmicutes_Bacilli_Lactobacillal:0.08063608):0.04502372[1.000000],(Bact_StreptococNewRepAgi|765385984|ref|WP_044671103.1|/1440Bacteria_Firmicutes_Bacilli_Lactobacillal:0.09798104,Bact_StreptococNewRepAgi|446668664|ref|WP_000746010.1|/1442Bacteria_Firmicutes_Bacilli_Lactobacillal:0.12555418):0.03363759[0.999948]):0.14808399[1.000000],(Bact_StreptococNewRepAgi|517191201|ref|WP_018380019.1|/1438Bacteria_Firmicutes_Bacilli_Lactobacillal:0.13486784,Bact_StreptococNewRepAgi|484887870|gb|EOB33201.1|/1484Bacteria_Firmicutes_Bacilli_Lactobacillales_St:0.12862621):0.06577189[0.999990]):0.12951130[0.999996]):0.11534240[0.982819]):0.08083805[0.999920]):0.20662481[1.000000]):0.09936315[0.905340]):0.47318856[1.000000],((Bact_LeuconostoNewRepAgi|1011462511|ref|WP_062359070.1|/1331Bacteria_Firmicutes_Bacilli_Lactobacilla:0.67372327,(gi|410500862|ref|YP_006939186.1|_467_bp:1.02048107,((Bact_CarnobacteNewRepAgi|511302329|ref|WP_016356676.1|/1390Bacteria_Firmicutes_Bacilli_Lactobacillal:0.13945343,gi|514893627|ref|WP_016622553.1|_plasmid_replication_protein_Enterococcus_faecalis_467_bp:0.08151928):0.62813785[1.000000],(((Bact_LactobacilNewRepAgi|502608600|ref|WP_012845653.1|/1415Bacteria_Firmicutes_Bacilli_Lactobacillal:0.12013302,(gi|550819760|emb|CDI42894.1|_467_bp:0.19707941,((gi|524264796|emb|CDA26462.1|_467_bp:0.08742603,(Bact_LactobacilNewRepAgi|489644618|ref|WP_003549058.1|/1366Bacteria_Firmicutes_Bacilli_Lactobacillal:0.04966713,Bact_LactobacilNewRepAgi|948885057|gb|KRN00682.1|/1366Bacteria_Firmicutes_Bacilli_Lactobacillales_La:0.03423256):0.08749412[1.000000]):0.04571414[0.992336],(gi|550819626|emb|CDI43023.1|_467_bp:0.08008795,Bact_LactobacilNewRepAgi|948608749|gb|KRK41125.1|/1366Bacteria_Firmicutes_Bacilli_Lactobacillales_La:0.17197918):0.03888997[0.599554]):0.07859101[1.000000]):0.05359708[0.997156]):0.30469965[1.000000],(((Bact_LactobacilNewRepAgi|504380679|ref|WP_014567781.1|/1342Bacteria_Firmicutes_Bacilli_Lactobacillal:0.31100297,(Bact_LactobacilNewRepAgi|494200615|ref|WP_007125042.1|/1347Bacteria_Firmicutes_Bacilli_Lactobacillal:0.11548642,Bact_LactobacilNewRepAgi|983177949|ref|WP_060461663.1|/1350Bacteria_Firmicutes_Bacilli_Lactobacillal:0.13806088):0.08832913[0.999982]):0.13764451[1.000000],((Bact_LactobacilNewRepAgi|896129273|ref|WP_049150683.1|/1348Bacteria_Firmicutes_Bacilli_Lactobacillal:0.27764580,(Bact_LactobacilNewRepAgi|809080192|ref|WP_046324376.1|/1346Bacteria_Firmicutes_Bacilli_Lactobacillal:0.23830479,(Bact_LactobacilNewRepAgi|495747574|ref|WP_008472153.1|/1340Bacteria_Firmicutes_Bacilli_Lactobacillal:0.10060237,Bact_LactobacilNewRepAgi|503406807|ref|WP_013641468.1|/1341Bacteria_Firmicutes_Bacilli_Lactobacillal:0.13009496):0.48614875[1.000000]):0.03460449[0.537939]):0.07396295[0.999996],(Bact_LactobacilNewRepAgi|495745299|ref|WP_008469878.1|/1335Bacteria_Firmicutes_Bacilli_Lactobacillal:0.25719806,Bact_LactobacilNewRepAgi|503406820|ref|WP_013641481.1|/1345Bacteria_Firmicutes_Bacilli_Lactobacillal:0.41871934):0.18458578[1.000000]):0.07294912[0.999508]):0.06001201[0.974991],(Bact_LactobacilNewRepAgi|949549434|ref|WP_056985318.1|/1338Bacteria_Firmicutes_Bacilli_Lactobacillal:0.48461702,Bact_LactobacilNewRepAgi|499573384|ref|WP_011254167.1|/1360Bacteria_Firmicutes_Bacilli_Lactobacillal:0.35709228):0.14587120[0.999999]):0.08313324[0.995097]):0.29870006[1.000000],(((gi|227352896|gb|EEJ43069.1|_467_bp:0.48565017,Bact_LeuconostoNewRepAgi|491038601|ref|WP_004900270.1|/1436Bacteria_Firmicutes_Bacilli_Lactobacillal:0.59481706):0.17365600[1.000000],(((Bact_LachnospirNewRepAgi|510893728|ref|WP_016226904.1|/1409Bacteria_Firmicutes_Clostridia_Clostridia:0.30009353,gi|1052710859|emb|SCH55298.1|_Plasmid_replication_protein_uncultured_Collinsella_sp._467_bp:0.48242572):0.24480808[1.000000],((gi|985376877|gb|KXA58447.1|_467_bp:0.00000019,(gi|446015711|ref|WP_000093566.1|_467_bp:0.00223402,(Bact_Streptococgi|913906122|ref|WP_050492321.1|/1398Bacteria_Firmicutes_Bacilli_Lactobacillales_Stre:0.00121040,bRCRep1Gr1no_r_no_rankgi|757662115|ref|WP_042900192.1|/1368no_rank_467_bp:0.00246677):0.00104770[0.333428]):0.00223260[0.494337]):0.62117207[1.000000],(((Bact_StreptococNewRepAgi|992650469|emb|CYX46115.1|/1391Bacteria_Firmicutes_Bacilli_Lactobacillales_S:0.09223563,gi|994516497|emb|CYW87437.1|_467_bp:0.04778015):0.06741686[0.999983],(Bact_StreptococNewRepAgi|637012935|ref|WP_024410839.1|/1392Bacteria_Firmicutes_Bacilli_Lactobacillal:0.05369319,(Bact_StreptococNewRepAgi|896644444|ref|WP_049523992.1|/1392Bacteria_Firmicutes_Bacilli_Lactobacillal:0.11725831,(gi|727222881|ref|WP_033683822.1|_replication_protein_Streptococcus_mitis_467_bp:0.01266438,Bact_StreptococNewRepAgi|308116017|gb|EFO53527.1|/1400Bacteria_Firmicutes_Bacilli_Lactobacillales_St:0.01825725):0.06172056[1.000000]):0.14733216[1.000000]):0.10429365[0.999928]):0.30835946[1.000000],(gi|916841750|ref|WP_051448806.1|_hypothetical_protein_Viridibacillus_arenosi_467_bp:0.16150127,(Bact_EnterococcNewRepAgi|913624207|ref|WP_050444210.1|/1401Bacteria_Firmicutes_Bacilli_Lactobacillal:0.06810806,Bact_EnterococcNewRepAgi|498517862|ref|WP_010817837.1|/1401Bacteria_Firmicutes_Bacilli_Lactobacillal:0.04253924):0.16735186[1.000000]):0.29152426[1.000000]):0.13027556[0.999997]):0.06185064[0.734307]):0.06213716[0.959550],Bact_StreptococNewRepAgi|145689324|gb|ABP89830.1|/1422Bacteria_Firmicutes_Bacilli_Lactobacillales_St:0.86435965):0.03765161[0.884367]):0.08709293[0.995937],((Bact_LactobacilNewRepAgi|818476398|gb|AKG47101.1|/1398Bacteria_Firmicutes_Bacilli_Lactobacillales_La:0.80515451,((Bact_LactobacilNewRepAgi|802106399|ref|WP_046025501.1|/1437Bacteria_Firmicutes_Bacilli_Lactobacillal:0.30841783,Bact_LactobacilNewRepAgi|736525609|ref|WP_034540695.1|/1453Bacteria_Firmicutes_Bacilli_Lactobacillal:0.18113804):0.59981492[1.000000],(Bact_LactobacilNewRepAgi|948892254|gb|KRN07545.1|/1423Bacteria_Firmicutes_Bacilli_Lactobacillales_La:0.58492841,(Bact_LactobacilNewRepAgi|970370106|emb|CUR41281.1|/1389Bacteria_Firmicutes_Bacilli_Lactobacillales_L:0.69270733,(Gr1bRCRep1Bact_Lactobacilgi|489761587|ref|WP_003665528.1|/1422Bacteria_Firmicutes_Bacilli_Lactobacil:0.56235771,(Bact_LactobacilNewRepAgi|822102754|ref|WP_046923918.1|/1452Bacteria_Firmicutes_Bacilli_Lactobacillal:0.57697010,Gr1bRCRep1Bact_Lactobacilgi|493545821|ref|WP_006499656.1|/1410Bacteria_Firmicutes_Bacilli_Lactobacil:0.56982210):0.09764055[0.893703]):0.05871225[0.681224]):0.04400521[0.409444]):0.09734774[0.980871]):0.07664638[0.925156]):0.08189788[0.966846],(bRCRep1Gr1Bact_Leuconostogi|488910317|ref|WP_002821392.1|/1412Bacteria_Firmicutes_Bacilli_Lactobacil:0.86626127,(Bact_LactobacilNewRepAgi|951455024|ref|WP_057827085.1|/1437Bacteria_Firmicutes_Bacilli_Lactobacillal:0.50950525,(Bact_LactobacilNewRepAgi|951595551|ref|WP_057906729.1|/1442Bacteria_Firmicutes_Bacilli_Lactobacillal:0.05414672,Bact_LactobacilNewRepAgi|951455790|ref|WP_057827851.1|/1442Bacteria_Firmicutes_Bacilli_Lactobacillal:0.13197928):0.50329319[1.000000]):0.09374353[0.990579]):0.11638925[0.999678]):0.07653044[0.999058]):0.07087490[0.998341]):0.04408713[0.606739]):0.04629267[0.595186]):0.06711332[0.937990]):0.17163326[1.000000],(((Bact_LachnospirNewRepAgi|524226796|emb|CCZ93342.1|/1406Bacteria_Firmicutes_Clostridia_Clostridiales_:0.30628383,Bact_ClostridiaNewRepAgi|524080755|emb|CCY61699.1|/1401Bacteria_Firmicutes_Clostridia_Clostridiales_:0.33811542):0.14487371[1.000000],((Bact_Vibrionacegi|518112292|ref|WP_019282500.1|/1396Bacteria_Proteobacteria_Gammaproteobacteria_Vibr:0.53070288,(gi|1063361096|gb|ODR34583.1|_hypothetical_protein_BEI60_22705_Eisenbergiella_tayi_467_bp:0.41357999,((Bact_AcholeplasNewRepAgi|1002389866|gb|KXT29039.1|/1402Bacteria_Tenericutes_Mollicutes_Acholeplasmat:0.37109332,(bRCRep1Gr1Bact_Acholeplasgi|190571873|ref|YP_001966814.1|/1375Bacteria_Tenericutes_Mollicutes_Achole:0.23496389,(Gr1bRCRep1Bact_Acholeplasgi|499474371|ref|WP_011161011.1|/1376Bacteria_Tenericutes_Mollicutes_Achole:0.03086586,(Gr1bRCRep1Bact_Acholeplasgi|84790141|gb|ABC65805.1|/1375Bacteria_Tenericutes_Mollicutes_Acholeplasma:0.06446740,(Gr1bRCRep1Bact_Acholeplasgi|84790090|gb|ABC65794.1|/1382Bacteria_Tenericutes_Mollicutes_Acholeplasma:0.15248083,(bRCRep1Gr1Bact_Acholeplasgi|410688119|ref|YP_006961027.1|/1377Bacteria_Tenericutes_Mollicutes_Achole:0.02881271,Gr1bRCRep1Bact_Acholeplasgi|296100131|ref|YP_003617079.1|/1375Bacteria_Tenericutes_Mollicutes_Achole:0.04741021):0.10414314[1.000000]):0.04539591[0.998036]):0.04559111[0.999973]):0.09986770[1.000000]):0.09738936[0.999608]):0.19372030[1.000000],(Bact_RuminococcNewRepAgi|524198135|emb|CCZ68460.1|/1412Bacteria_Firmicutes_Clostridia_Clostridiales_:0.37662841,gi|1052950894|emb|SCG87263.1|_Plasmid_replication_protein_uncultured_Clostridium_sp._467_bp:0.51674759):0.30746723[1.000000]):0.11249640[0.999649]):0.09212566[0.995311]):0.09158495[0.988806],(Bact_AcetobacteNewRepAgi|524716769|emb|CDE19587.1|/1350Bacteria_Proteobacteria_Alphaproteobacteria_R:0.73358033,Bact_RuminococcNewRepAgi|655061540|ref|WP_028509833.1|/1453Bacteria_Firmicutes_Clostridia_Clostridia:0.69799144):0.21812963[0.999977]):0.06622481[0.974104]):0.09432824[0.982896],((Bact_LachnospirNewRepAgi|488641492|ref|WP_002578150.1|/1451Bacteria_Firmicutes_Clostridia_Clostridia:0.67261250,gi|933374795|emb|CUN62864.1|_Plasmid_replication_protein_Hungatella_hathewayi_467_bp:0.57172565):0.09205862[0.567572],(UniRef50_W1I2Y7_467_bp:1.34979708,(UniRef50_R5ENB8_467_bp:0.94781810,UniRef50_A0A0E9FQH7_467_bp:0.57682437):0.36412671[0.720831]):0.60156070[0.973545]):0.14018496[0.807813]):0.42972562[1.000000]):0.03665087[0.618156]):0.06620238[0.934424]):0.10300219[0.950020]):0.06963356[0.480010],(((((UniRef50_K7YFJ8_467_bp:0.63820126,UniRef50_O31070_467_bp:0.47427885):0.37058195[1.000000],((UniRef50_A0A158LH93_467_bp:0.58653498,(((UniRef50_A0A0H5PZW4_467_bp:0.56436102,UniRef50_A0A0H5PV05_467_bp:0.59049901):0.16531075[0.999675],((UniRef50_A0A1C6AUS2_467_bp:0.36743737,UniRef50_A0A0E9DRD0_467_bp:0.34089180):0.17007485[0.999979],((UniRef50_G8CNT2_467_bp:0.27795890,UniRef50_A0A0E9F4G4_467_bp:0.56787317):0.32260161[1.000000],(UniRef50_A0A0H5PZG0_467_bp:0.56023650,UniRef50_A0A0H5QIL6_467_bp:0.54443696):0.32000948[0.999999]):0.06696794[0.463517]):0.01582145[0.390905]):0.03521317[0.756391],(UniRef50_A0A1Y4G0S9_467_bp:0.59621461,(UniRef50_F0HMF3_467_bp:0.65524137,(lcl|UniRef50_D3R6U9_467_bp:0.15268185,(UniRef50_A0A087EKU7_467_bp:0.15564052,UniRef50_A0A0S2MGE2_467_bp:0.23621602):0.07736179[0.732016]):0.69931930[1.000000]):0.24498691[0.999708]):0.13832375[0.996096]):0.08097533[0.954566]):0.18170283[0.999998],(UniRef50_Q48831_467_bp:1.11959911,(((UniRef50_A0A0R1P770_467_bp:1.00064271,(UniRef50_A0A1W6BZG0_467_bp:0.68629810,UniRef50_A0A0H5PZA0_467_bp:0.64634001):0.09302246[0.729883]):0.11070340[0.947654],(UniRef50_A0A1B1IHL4_467_bp:1.03218344,(UniRef50_A0A0R3QHC2_467_bp:0.43541925,(UniRef50_S6F6F1_467_bp:0.88599494,UniRef50_W7D2V3_467_bp:0.37991032):0.21765062[0.999713]):0.09874241[0.608570]):0.08665821[0.962844]):0.02561706[0.409654],((UniRef50_UPI000300949E_467_bp:0.64833900,(UniRef50_A0A0H5PZI7_467_bp:0.55083526,UniRef50_G8CNR9_467_bp:0.46019434):0.12101022[0.972191]):0.09230019[0.724037],(UniRef50_W1I557_467_bp:0.36356828,UniRef50_A0A0Z8IYX5_467_bp:0.82268175):0.18601760[0.980742]):0.06773106[0.861599]):0.07647397[0.996233]):0.02979319[0.426813]):0.11962711[0.998082]):0.08763311[0.665342],(UniRef50_U2QZX4_467_bp:0.96251156,((((UniRef50_A0A0H5Q0X0_467_bp:0.43989097,UniRef50_A0A0E9EV38_467_bp:0.37366637):0.13955703[0.997467],(UniRef50_K9RZV9_467_bp:0.38182001,UniRef50_K9RYD5_467_bp:0.88320785):0.20390082[0.999971]):0.08178581[0.870828],(UniRef50_UPI000481AAFB_467_bp:1.04855963,lcl|UniRef50_U2EU97_467_bp:0.16396267):0.16779266[0.990291]):0.12446754[0.998002],(UniRef50_A0A0H5Q8X5_467_bp:0.86557966,(UniRef50_S6CES9_467_bp:1.41135799,(UniRef50_U2TJ01_467_bp:0.66807818,UniRef50_A0A1Y3UDM3_467_bp:0.71180632):0.12116816[0.419356]):0.18257740[0.904209]):0.16935238[0.971498]):0.08149693[0.969653]):0.08821648[0.917880]):0.17558169[0.998463],((UniRef50_A0A1Y4QQC0_467_bp:0.28295681,(UniRef50_A8W662_467_bp:0.31471706,3DKX_A_467_bp:0.13915769):0.22015837[0.999468]):0.55262806[1.000000],(UniRef50_W1I697_467_bp:0.68962725,(UniRef50_A0A174GG61_467_bp:0.79929093,UniRef50_A0A0E9F9L7_467_bp:0.43968470):0.19077073[0.981112]):0.57966541[1.000000]):0.30506891[0.997821]):0.73522212[1.000000],(((833ee4171fb5d86e62b3fe0f3c1e026947ca78ff9113d1c474570a16e65ae785-10_467_bp:0.55128144,(BAN59850.1|Thalassionema_467_bp:0.55988024,(AUF34977.1_467_bp:0.54790223,(((5bc306a9607207a0efbbea55c33a7c25fdc354f8e2e77d7f1d8398e102b42c13-10_467_bp:0.26089399,(65c9a819eeecc58815473d6730a3a9d710279d3bcb36cbe602dbb43ca9714427-00_467_bp:0.15835862,YP_009109635.1_467_bp:0.19513913):0.14147813[1.000000]):0.08041709[0.999976],((fb8f6287d1d0a95e973165257530b7322c29b1a18a92f17aa84d58d4e9a32e97-00_467_bp:0.36938112,(36ff03fbe1a9a1060a16d972e21b61fe3bbf57259b2dabec48e9b740ac32cdb4-00_467_bp:0.33603731,(9e1d9b352c6c57b8bf4c20295d58383736f38c02650e268d4687499f14df912e-00_467_bp:0.22944330,(41834aaa2f2e36cb0531dc8dada4b7084d7cf50a8b65d6fd756c9b40bd7d5721-00_467_bp:0.26916196,(b81c81ec2fabc56fb906dfdae75b4597250b6618c2a9e5d7d592074574688346-01_467_bp:0.48972788,(0bd68b5a40c5a6044c3ff40ca9815e30c548f9eeb6ef87aca3f144ce65cf7441-00_467_bp:0.21481624,YP_009345097.1_467_bp:0.21424208):0.20493697[1.000000]):0.06117408[0.991711]):0.05725691[0.999070]):0.07251320[0.984844]):0.04946440[0.929387]):0.13149507[1.000000],((YP_009345107.1_467_bp:0.15892952,(2851f14acec255faf0601dc5f18e5a234ecb640e1516df67be746e6316b0d50f-00_467_bp:0.19460972,YP_009345086.1_467_bp:0.13367504):0.10497217[1.000000]):0.12297343[1.000000],((YP_004046698.1|Chaetoceros_467_bp:0.10875136,(YP_009111348.1_467_bp:0.04787794,BAP99817.1_467_bp:0.04268233):0.06403679[0.993461]):0.27581304[1.000000],(YP_473359.1_467_bp:0.26120936,((YP_009001777.1_467_bp:0.14833909,a43e97046e8aad84b088372367f8909fe0bea7fa95b4e398e7eaa90494b94205-10_467_bp:0.19784372):0.11060218[1.000000],(BAL05205.1_467_bp:0.23286972,YP_004286322.1_467_bp:0.26580355):0.07531251[0.999716]):0.06646453[0.997122]):0.11822686[1.000000]):0.12026294[1.000000]):0.03936220[0.582723]):0.06985442[0.999451]):0.05261474[0.999995],((827d7105bece8830299875f2b521c58893e8baa105c1d682254ad2968e2d9817-00_467_bp:0.15975966,fc270c7af120b5db22a92e67a52615ba84787c103ed6948ced3f64c154ae819d-00_467_bp:0.27007878):0.18372474[1.000000],((bd4c33c6592559bdffb711a3ce94a3264224e9910e589e1eba5213be9394799d-00_467_bp:0.23827321,e297896c0a2982c2684f01eb18de03f161b1701381986ca875d14abb8d518137-01_467_bp:0.16458661):0.16515620[1.000000],(51dc4f0b8ef1f29067aa5edd136201ee8177aa724a7e9096a2a83f9aea624d2a-00_467_bp:0.22668523,282ad189c388a58a159bc47ec08d617866074d3026c12ef4c7ce1fa861ba629f-00_467_bp:0.16121816):0.05145889[0.959762]):0.06223316[0.999984]):0.05319882[0.999923]):0.09555442[0.999627]):0.08071428[0.951530]):0.08718742[0.568639]):1.14230735[1.000000],((((KT149395|CRESS_unclass_1..894_467_bp:0.55967335,KM598390|CRESS_unclass_1..891_467_bp:0.59341629):0.11048127[0.809894],(((KM874358|CRESS_unclass_1..885_467_bp:0.46343130,aRR_AJD07486.1_467_bp:0.59928312):0.29080731[1.000000],(KT732829|CRESS_unclass_1..1092_467_bp:0.60029312,(KM510189|CRESS_unclass_1..1086_467_bp:0.44315830,KP005454|CRESS_unclass_1..1074_467_bp:0.36845813):0.58266754[1.000000]):0.25509860[1.000000]):0.12689141[0.971118],KP153501|CRESS_unclass_1..879_467_bp:0.58983059):0.09836409[0.936846]):0.51458395[1.000000],((gi|7108461|gb|AAF36424.1|AF106328_1_replicase_Porphyra_pulchra_467_bp:0.09792483,(bRCRep5Euka_Bangiaceaegi|7108459|gb|AAF36423.1|AF106327_1/1378Eukaryota_Bangiophyceae_Bangiales_Bang:0.05843259,Euka_BangiaceaeNewRepAgi|7108457|gb|AAF36422.1|AF106326_1/1378Eukaryota_Bangiophyceae_Bangiales_Bang:0.04293027):0.13359434[0.999851]):1.00263864[1.000000],(aRR_OLY79699.1_467_bp:0.98393804,((aRR_OMJ09562.1|Replication-associated_467_bp:0.55434888,(aRR_OMJ11569.1_467_bp:0.21090223,(aRR_OMJ21113.1|Replication-associated_467_bp:0.05926822,(aRR_OMJ28371.1_467_bp:0.10480133,aRR_OMJ13215.1_467_bp:0.01722782):0.06910560[0.625791]):0.28767291[1.000000]):0.08626467[0.956532]):0.10320372[0.970411],((aRR_OLY79419.1|Replication-associated_467_bp:0.22626010,aRR_OLY79389.1_467_bp:0.25178061):0.28836316[1.000000],aRR_ETO15557.1_467_bp:0.65277994):0.11020657[0.961197]):0.04812727[0.359148]):0.14344576[0.969167]):0.31561407[0.999979]):0.35106007[0.999994],((((aRR_YP_009351871.1_467_bp:0.93475628,((KJ547626|CRESS_unclass_1..873_467_bp:0.58924055,(KJ938716|CRESS_unclass_1..1140_467_bp:0.25163314,KM821748|CRESS_unclass_1..1110_467_bp:0.21861300):0.80076378[1.000000]):0.11235830[0.636279],(KJ547634|Genomovirus_1..993_467_bp:0.63911386,((AJD07464.1|Odonata_467_bp:0.21883594,AMH87693.1|Pacific_467_bp:0.23826198):0.36352614[1.000000],((YP_009164036.1|Gemycircularvirus_467_bp:0.47926433,(AMH87708.1|Pacific_467_bp:0.18754917,YP_009109725.1|Faeces_467_bp:0.56460299):0.06590755[0.910852]):0.06306417[0.561211],((((((YP_009115514.1|Sewageassociated_467_bp:0.18715811,YP_003104796.1|Sclerotinia_467_bp:0.18645505):0.13198870[1.000000],((AMH87666.1|Pacific_467_bp:0.30422532,YP_009252353.1|Faeces_467_bp:0.14915626):0.05265339[0.997904],((YP_009115515.1|Sewageassociated_467_bp:0.10477213,(AIF34843.1|Sewageassociated_467_bp:0.12977196,(KJ547638|Genomovirus_1..984_467_bp:0.06183540,YP_009115519.1|Sewageassociated_467_bp:0.06544825):0.03481712[0.652715]):0.04453156[0.999778]):0.11833421[1.000000],(YP_009252356.1|Faeces_467_bp:0.14641741,(YP_009252368.1|Faeces_467_bp:0.10100817,YP_009021043.1|Cassava_467_bp:0.04138995):0.22926065[1.000000]):0.03737710[0.958223]):0.06742905[1.000000]):0.04403954[0.999972]):0.02099229[0.404010],AGS12486.1|Hypericum_467_bp:0.32473291):0.13743812[1.000000],(AMH87733.1|Pacific_467_bp:0.35515718,(YP_009109727.1|Faeces_467_bp:0.30600289,YP_009252362.1|Faeces_467_bp:0.26778771):0.15972595[1.000000]):0.06375850[0.902050]):0.04201849[0.893570],(((YP_009109733.1|Faeces_467_bp:0.08212932,KT862241|Genomovirus_1..1026_467_bp:0.05127445):0.22297196[1.000000],(AMH87678.1|Pacific_467_bp:0.19986856,(KT253577|Genomovirus_1..1008_467_bp:0.15442097,(YP_009252365.1|Faeces_467_bp:0.18197315,YP_009252359.1|Faeces_467_bp:0.13334724):0.10715978[1.000000]):0.05324977[0.996889]):0.12757093[1.000000]):0.12203607[1.000000],(YP_009109729.1|Faeces_467_bp:0.51905285,(YP_009181999.1|Soybean_467_bp:0.01176119,KT598248|Genomovirus_1..1014_467_bp:0.00000019):0.37302846[1.000000]):0.14578636[0.999995]):0.10887297[1.000000]):0.09890396[0.996807],AMH87702.1|Pacific_467_bp:0.48571923):0.05767435[0.770322]):0.05726345[0.833781]):0.52729765[1.000000]):0.18039358[0.999265]):0.10246149[0.897185]):0.10158764[0.649464],(KP153522|CRESS_unclass_1..1053_467_bp:0.88978480,(aRR_AUM61807.1_467_bp:0.79939076,(KM598389|CRESS_unclass_1..1062_467_bp:0.45900850,KJ547627|CRESS_unclass_1..948_467_bp:0.47335552):0.36018846[1.000000]):0.09087781[0.611114]):0.05570480[0.373966]):0.21864427[1.000000],(((ACO88014.1|Eragrostis_467_bp:0.01747433,FJ665634|Geminivirus_1..1104_467_bp:0.00350435):0.59022527[1.000000],((AGV02076.1|Cotton_467_bp:0.09923893,((AAL96826.1|Cotton_467_bp:0.13913957,(YP_003828907.1|Melon_467_bp:0.11813039,CRI68211.1|Pepper_467_bp:0.18534890):0.02529159[0.961651]):0.02569424[0.922465],(AFM38721.1|Jacquemontia_467_bp:0.19511787,(NP_066185.1|Horseradish_467_bp:0.11104210,YP_003966137.1|Spinach_467_bp:0.09664743):0.06254671[1.000000]):0.04558970[0.999970]):0.01669551[0.907931]):0.10397531[0.999977],(YP_007250561.1|Tomato_467_bp:0.13842971,(((((ACV83312.1|Tomato_467_bp:0.09922024,AER09339.1|Cleome_467_bp:0.09066899):0.06179435[1.000000],(YP_002941855.1|Passionfruit_467_bp:0.10707201,YP_006590064.1|Soybean_467_bp:0.09704465):0.01829492[0.629700]):0.02555952[0.999960],((AGH29892.1|Tomato_467_bp:0.05825227,((ALV85583.1|Pavonia_467_bp:0.12081818,(CBA18089.1|Sida_467_bp:0.08193755,FJ665283|Geminivirus_1..1080_467_bp:0.06675845):0.02050509[0.956599]):0.03819268[1.000000],(AMW86999.1|Sida_467_bp:0.15830730,CBH28932.1|Sida_467_bp:0.12885905):0.00747185[0.455131]):0.01044094[0.974952]):0.05239373[1.000000],(AHX57826.1|Jacquemontia_467_bp:0.15717987,(NP_671468.1|Macroptilium_467_bp:0.13267153,AFD54490.1|Macroptilium_467_bp:0.13996239):0.01834175[0.757927]):0.01958577[0.996289]):0.01853531[0.980447]):0.01435164[0.841908],((ADN84041.1|Rhynchosia_467_bp:0.11354551,(AAN76737.1|Macroptilium_467_bp:0.07431891,(YP_001333687.1|Corchorus_467_bp:0.12221893,YP_115511.1|Corchorus_467_bp:0.06657375):0.23351046[1.000000]):0.04224352[0.999893]):0.06461951[1.000000],((YP_001285764.1|Spilanthes_467_bp:0.21361212,AMP46444.1|Deinbollia_467_bp:0.13642474):0.06915437[1.000000],((AAB87607.1|Cowpea_467_bp:0.07655744,AGK24653.1|Soybean_467_bp:0.10247999):0.12716544[1.000000],((YP_003622552.1|Soybean_467_bp:0.15287021,(ADW24243.1|Kudzu_467_bp:0.14008606,(ABD67440.1|Mungbean_467_bp:0.26843916,AKS48121.1|Rhynchosia_467_bp:0.10342185):0.07092087[1.000000]):0.03802779[0.995931]):0.05127446[1.000000],(aRR_APP87725.1_467_bp:0.32606863,(((AIY31184.1|Tomato_467_bp:0.10431493,AEE99005.1|Tomato_467_bp:0.21526023):0.07631095[1.000000],(((BAF02752.1|Pepper_467_bp:0.17794252,(aRR_AHL29198.1_467_bp:0.06910175,(CAJ85998.1|Malvastrum_467_bp:0.08812753,(AFF58888.1|Clerodendrum_467_bp:0.13885706,(AGG08895.1|Lindernia_467_bp:0.08173194,AGF41094.1|Papaya_467_bp:0.05809559):0.02710141[0.895197]):0.02450336[0.997164]):0.01525871[0.972679]):0.01596543[0.987226]):0.01262335[0.774515],(ABD35287.1|Alternanthera_467_bp:0.18628959,((AFA26437.2|Pedilanthus_467_bp:0.05662254,((AFB81519.1|Cotton_467_bp:0.10208159,ACI06063.1|Mesta_467_bp:0.11283007):0.03357084[0.999999],(AFB83419.1|Cotton_467_bp:0.12490391,AFH68197.1|Hollyhock_467_bp:0.10941235):0.00000026[0.000000]):0.05042663[1.000000]):0.01389821[0.780169],((AGJ03640.1|Chilli_467_bp:0.15650568,ACB44970.1|Tomato_467_bp:0.05759142):0.10135054[1.000000],(AHA82274.1|Tomato_467_bp:0.16451110,(AGV02071.1|Okra_467_bp:0.13038010,(ACV60535.1|Tomato_467_bp:0.09609181,(AAF75542.1|Tobacco_467_bp:0.11108314,NP_050017.1|Pepper_467_bp:0.09576278):0.03486713[0.999770]):0.02247593[0.999979]):0.01416912[0.980967]):0.02354231[0.998896]):0.01792635[0.995468]):0.02568168[0.999878]):0.01746049[0.630830]):0.01679348[0.999957],((CDW92215.1|Tomato_467_bp:0.11896421,(YP_006905839.1|Jatropha_467_bp:0.12526791,(AEY63664.1|Watermelon_467_bp:0.17390062,(AAX39336.1|African_467_bp:0.02118209,FM877473|Geminivirus_1..1077_467_bp:0.02073436):0.06504334[1.000000]):0.04600868[1.000000]):0.03879020[0.999999]):0.02307253[0.999155],(YP_008411025.1|Hemidesmus_467_bp:0.21203653,NP_620741.1|Tomato_467_bp:0.10229462):0.02371164[0.927464]):0.01343567[0.990376]):0.02776245[0.999996]):0.01830132[0.998817],(((AJM13604.1|Asystasia_467_bp:0.10864796,YP_004958233.1|West_467_bp:0.13990312):0.06573706[1.000000],(YP_009129272.1|Apple_467_bp:0.18871173,CAM91896.1|Tomato_467_bp:0.09483819):0.02632273[0.944033]):0.08401924[1.000000],(YP_764516.1|Okra_467_bp:0.09881013,((YP_001040016.1|Tomato_467_bp:0.06576958,(AAP73446.1|Dolichos_467_bp:0.23903688,(ABG90906.1|Merremia_467_bp:0.02086839,ACY79450.1|Sweet_467_bp:0.18563087):0.14249391[1.000000]):0.04641460[0.995295]):0.02062520[0.908015],(AMK07575.1|Lycianthes_467_bp:0.16931697,(YP_002224032.1|Jatropha_467_bp:0.18819655,CBJ17676.1|Croton_467_bp:0.05394950):0.02754494[0.991632]):0.03823032[0.999998]):0.09982360[1.000000]):0.01481946[0.930243]):0.00614623[0.508358]):0.01491471[0.970465]):0.02395577[0.994732]):0.02326447[0.999804]):0.01170208[0.961058]):0.04834114[1.000000]):0.14447738[1.000000]):0.02441814[0.999930],((NP_040557.1|Beet_467_bp:0.14542680,(ALF37659.1|Beet_467_bp:0.15969973,(ALR86823.1|Turnip_467_bp:0.21386596,(YP_009226627.1|Turnip_467_bp:0.08064922,(YP_003778178.1|Turnip_467_bp:0.00977338,KC108902|Geminivirus_1..1116_467_bp:0.03744140):0.15200002[1.000000]):0.03424696[0.914514]):0.11964175[1.000000]):0.07055016[0.999993]):0.11132161[1.000000],YP_619883.1|Tomato_467_bp:0.14371468):0.00780814[0.453238]):0.01923419[0.952081]):0.10688685[1.000000]):0.18577212[1.000000]):0.25305762[1.000000],((KT214373|Geminivirus_1..951_467_bp:0.12916253,JX094280|Geminivirus_1..948_467_bp:0.16030736):0.40597802[1.000000],(DQ458791|Geminivirus_1..1005_467_bp:0.33517489,(((YP_006666535.1|Digitaria_467_bp:0.10584440,(AFN80601.1|Paspalum_467_bp:0.12905312,(YP_004089627.1|Bromus_467_bp:0.09998747,YP_006666523.1|Paspalum_467_bp:0.14460702):0.03590090[0.999988]):0.06791650[0.999184]):0.25275879[1.000000],(YP_003915159.1|Digitaria_467_bp:0.18696972,AFN80669.1|Chloris_467_bp:0.25670478):0.16346900[1.000000]):0.23558246[1.000000],(((AHM88370.1|Maize_467_bp:0.34639052,((AAK73446.1|Maize_467_bp:0.00423128,AF003952|Geminivirus_1..1068_467_bp:0.01569993):0.21122334[1.000000],(P0C647.1|Panicum_467_bp:0.26761752,((AHM88382.1|Urochloa_467_bp:0.12721956,YP_003288768.1|Saccharum_467_bp:0.15570519):0.02823325[0.860179],(AHM88378.1|Sugarcane_467_bp:0.15117013,Q80GM6.2|Sugarcane_467_bp:0.16139740):0.11195768[1.000000]):0.03048904[0.919408]):0.03697520[0.836456]):0.14260422[1.000000]):0.06783881[0.996044],(YP_006273070.1|Wheat_467_bp:0.31537561,YP_009026388.1|Sugarcane_467_bp:0.41208654):0.06625144[0.888514]):0.11167189[1.000000],(YP_009021763.1|Axonopus_467_bp:0.43031438,(AIT39773.1|Switchgrass_467_bp:0.42518253,(AFV91331.1|Dragonflyassociated_467_bp:0.24893768,(YP_006666531.1|Sporobolus_467_bp:0.23377688,YP_006666527.1|Sporobolus_467_bp:0.39033491):0.07652273[0.999618]):0.03739738[0.594586]):0.08483435[0.999777]):0.04804741[0.507722]):0.03738931[0.560821]):0.14365322[1.000000]):0.23165198[1.000000]):0.19098884[0.999993]):0.13761092[0.999911]):0.20010835[0.989576],(((aRR_WP_012662291.1_467_bp:0.03361297,((gi|51172577|dbj|BAD36752.1|_467_bp:0.00446681,aRR_WP_042068233.1_467_bp:0.00000013):0.02847648[1.000000],(bRC-Rep5Bact_Acholeplasgi|410687490|ref|YP_006959585.1|/1-434Bacteria_Tenericutes_Mollicutes_Acholep:0.00000013,(gi|499583384|ref|WP_011264167.1|_467_bp:0.00677574,aRR_WP_015060110.1_467_bp:0.00223707):0.00000013[0.000000]):0.01575717[0.999982]):0.02153084[0.565921]):0.76066530[1.000000],((bRC-Rep5Bact_Acholeplasgi|515760571|ref|WP_017193171.1|/1-381Bacteria_Tenericutes_Mollicutes_Acholep:0.01650386,bRC-Rep5Bact_Acholeplasgi|515761095|ref|WP_017193695.1|/1-386Bacteria_Tenericutes_Mollicutes_Acholep:0.05277951):0.19632888[1.000000],((aRR_ATL14544.1_467_bp:0.01512688,(bRC-Rep5Bact_Acholeplasgi|169546398|ref|YP_001708784.1|/1-367Bacteria_Tenericutes_Mollicutes_Acholep:0.09062518,(bRC-Rep5Bact_Acholeplasgi|425702627|ref|YP_007008175.1|/1-392Bacteria_Tenericutes_Mollicutes_Acholep:0.03034052,gi|504896643|ref|WP_015083745.1|_467_bp:0.02921770):0.03165502[0.998571]):0.03241456[0.999861]):0.10201371[1.000000],(bRC-Rep5Bact_Acholeplasgi|410688730|ref|YP_006961991.1|/1-396Bacteria_Tenericutes_Mollicutes_Acholep:0.05974384,(((gi|503512976|ref|WP_013747472.1|_467_bp:0.05459019,(bRC-Rep5Bact_Acholeplasgi|169546405|ref|YP_001708790.1|/1-367Bacteria_Tenericutes_Mollicutes_Acholep:0.07477337,bRC-Rep5Bact_Acholeplasgi|425702632|ref|YP_007008179.1|/1-367Bacteria_Tenericutes_Mollicutes_Acholep:0.04434900):0.02662509[0.999946]):0.02672365[1.000000],((Bact_Acholeplasgi|499732216|ref|WP_011412950.1|/1-372Bacteria_Tenericutes_Mollicutes_Acholeplasmatal:0.03271229,gi|307902848|emb|CBX25033.1|_467_bp:0.02275748):0.17815435[1.000000],(aRR_ABC65385.1_467_bp:0.19713001,(Bact_Acholeplasgi|499732224|ref|WP_011412958.1|/1-368Bacteria_Tenericutes_Mollicutes_Acholeplasmatal:0.00000051,aRR_ABC65268.1_467_bp:0.05739380):0.05938372[1.000000]):0.01559184[0.953665]):0.02056926[0.996456]):0.03518244[0.999990],(bRC-Rep5Bact_Acholeplasgi|190410564|ref|YP_001965310.1|/1-377Bacteria_Tenericutes_Mollicutes_Acholep:0.11576516,bRC-Rep5Bact_Acholeplasgi|190410559|ref|YP_001965305.1|/1-376Bacteria_Tenericutes_Mollicutes_Acholep:0.23760137):0.03555792[0.946913]):0.03505060[0.998592]):0.05842136[1.000000]):0.08094895[0.975360]):0.31238326[1.000000]):0.17694126[0.999266],(Bact_AcholeplasNewRepAgi|1002389832|gb|KXT29014.1|/1-442Bacteria_Tenericutes_Mollicutes_Acholeplasma:1.06722898,Bact_AcholeplasNewRepAgi|1002389859|gb|KXT29032.1|/1-455Bacteria_Tenericutes_Mollicutes_Acholeplasma:0.95545398):0.12979390[0.694675]):0.44418263[1.000000]):0.26895768[0.999997]):0.49914506[1.000000]):0.12030893[0.675949],((((Bact_LachnospirNewRepAgi|651929165|ref|WP_026669310.1|/1380Bacteria_Firmicutes_Clostridia_Clostridia:0.06695092,bRCRep2Gr2Bact_Lachnospirgi|651421429|ref|WP_026524352.1|/1382Bacteria_Firmicutes_Clostridia_Clostri:0.04783366):0.57809043[1.000000],(Bact_CoriobacteNewRepAgi|1016804942|emb|CVH76026.1|/1360Bacteria_Actinobacteria_Coriobacteriia_Corio:1.24158378,(((Gr2bRCRep2Bact_Streptococgi|489192824|ref|WP_003102166.1|/1395Bacteria_Firmicutes_Bacilli_Lactobacil:0.07434506,(Bact_StreptococNewRepAgi|656228408|ref|WP_029176105.1|/1411Bacteria_Firmicutes_Bacilli_Lactobacillal:0.12083477,Gr2bRCRep2Bact_Streptococgi|445954276|ref|WP_000032131.1|/1411Bacteria_Firmicutes_Bacilli_Lactobacil:0.11608770):0.03686031[0.665086]):0.22999177[1.000000],(bRCRep2Gr2no_r_no_rankgi|658497599|ref|WP_029694263.1|/1424no_rank_467_bp:0.29228864,(Bact_StreptococNewRepAgi|1011060458|ref|WP_062004798.1|/1420Bacteria_Firmicutes_Bacilli_Lactobacilla:0.15327354,(Gr2bRCRep2Bact_Streptococgi|489121099|ref|WP_003030931.1|/1423Bacteria_Firmicutes_Bacilli_Lactobacil:0.06165749,(Bact_StreptococNewRepAgi|658493909|ref|WP_029690610.1|/1423Bacteria_Firmicutes_Bacilli_Lactobacillal:0.14172745,Bact_StreptococNewRepAgi|827365996|ref|WP_047207334.1|/1424Bacteria_Firmicutes_Bacilli_Lactobacillal:0.10747964):0.03343379[0.997615]):0.15871911[1.000000]):0.08527496[0.998824]):0.12730892[0.999698]):0.42263744[1.000000],(Bact_LachnospirNewRepAgi|928958162|ref|WP_053982727.1|/1360Bacteria_Firmicutes_Clostridia_Clostridia:0.71702549,(Bact_RuminococcNewRepAgi|524805683|emb|CDF01935.1|/1343Bacteria_Firmicutes_Clostridia_Clostridiales_:0.58557999,(gi|933077947|emb|CUO57637.1|_467_bp:0.31504824,gi|933018020|emb|CUO23215.1|_467_bp:0.21345585):0.15121889[1.000000]):0.08328070[0.980845]):0.07710850[0.999870]):0.06871210[0.769892]):0.13707546[0.763269]):0.50214299[1.000000],(((Bact_RuminococcNewRepAgi|518916410|ref|WP_020072285.1|/1344Bacteria_Firmicutes_Clostridia_Clostridia:0.53293686,(((Gr3_4bRCRep4no_r_no_rankgi|739545959|ref|WP_037404274.1|/1322no_rank_467_bp:0.41329097,(((Bact_LachnospirNewRepAgi|517426576|ref|WP_018597672.1|/1327Bacteria_Firmicutes_Clostridia_Clostridia:0.25592712,(gi|1053015557|emb|SCH17786.1|_467_bp:0.18701755,(gi|933361505|emb|CUP05665.1|_467_bp:0.20801784,Bact_ClostridiaNewRepAgi|524513064|emb|CDC44519.1|/1336Bacteria_Firmicutes_Clostridia_Clostridiales_:0.26213047):0.06852462[0.884154]):0.13502522[1.000000]):0.02992181[0.759346],(Gr3_4bRCRep4Bact_Erysipelotgi|334296474|dbj|BAK32345.1|/1309Bacteria_Firmicutes_Erysipelotrichia_Ery:0.38369477,(gi|740530313|ref|WP_038350939.1|_467_bp:0.18278511,(Bact_ClostridiaNewRepAgi|800881782|gb|KJZ87129.1|/1332Bacteria_Firmicutes_Clostridia_Clostridiales_C:0.12954309,Bact_ClostridiaNewRepAgi|565897363|ref|WP_023977019.1|/1332Bacteria_Firmicutes_Clostridia_Clostridia:0.16085299):0.11910969[1.000000]):0.03113604[0.672507]):0.07531974[0.913533]):0.07383816[1.000000],(bRCRep4Gr3_4Bact_Lachnospirgi|769153456|ref|WP_044928503.1|/1333Bacteria_Firmicutes_Clostridia_Clost:0.33316405,Bact_PlanococcaNewRepAgi|921222095|ref|WP_053167095.1|/1349Bacteria_Firmicutes_Bacilli_Bacillales_Pl:0.43989203):0.03345895[0.484763]):0.05411907[0.999951]):0.03787061[0.984814],(Bact_OscillospiNewRepAgi|524374305|emb|CDB27189.1|/1309Bacteria_Firmicutes_Clostridia_Clostridiales_:0.49581989,Bact_AcidaminocNewRepAgi|524774299|emb|CDE72464.1|/1332Bacteria_Firmicutes_Negativicutes_Acidaminoco:0.33838822):0.04505617[0.579790]):0.02246620[0.848921],(((Bact_LachnospirNewRepAgi|917032612|ref|WP_051639324.1|/1354Bacteria_Firmicutes_Clostridia_Clostridia:0.48834197,((gi|1054787244|ref|WP_066550639.1|_467_bp:0.28929685,(bRCRep4Gr3_4Bact_Lachnospirgi|495140917|ref|WP_007865724.1|/1335Bacteria_Firmicutes_Clostridia_Clost:0.07015030,gi|503035948|ref|WP_013270924.1|_467_bp:0.07245344):0.26318389[1.000000]):0.17950964[1.000000],((bRCRep4Gr3_4Bact_Lachnospirgi|636819923|ref|WP_024346025.1|/1335Bacteria_Firmicutes_Clostridia_Clost:0.13052288,bRCRep4Gr3_4no_r_no_rankgi|740446819|ref|WP_038278663.1|/1341no_rank_467_bp:0.12533846):0.19622079[1.000000],(bRCRep4Gr3_4Bact_Ruminococcgi|534538258|gb|EES75484.2|/1432Bacteria_Firmicutes_Clostridia_Clostridia:0.74883984,((Gr3_4bRCRep4Bact_Lachnospirgi|496540491|ref|WP_009246639.1|/1343Bacteria_Firmicutes_Clostridia_Clost:0.21074780,Bact_EubacteriaNewRepAgi|524091900|emb|CCY69022.1|/1342Bacteria_Firmicutes_Clostridia_Clostridiales_:0.16162699):0.09616772[0.999947],(Bact_no_rankNewRepAgi|917404352|ref|WP_052011064.1|/1311Bacteria/1311bacterium_LF3_467_bp:0.27657693,Bact_LachnospirNewRepAgi|523989767|emb|CCX75435.1|/1340Bacteria_Firmicutes_Clostridia_Clostridiales_:0.22039906):0.14135212[1.000000]):0.04608214[0.918341]):0.05038397[0.851840]):0.09720368[1.000000]):0.02947586[0.545439]):0.06769165[0.999992],(Bact_EubacteriaNewRepAgi|916994146|ref|WP_051600858.1|/1402Bacteria_Firmicutes_Clostridia_Clostridia:0.60690104,(Bact_EggerthellNewRepAgi|503744474|ref|WP_013978550.1|/1321Bacteria_Actinobacteria_Coriobacteriia_Eg:0.51210506,((Bact_RuminococcNewRepAgi|291542123|emb|CBL15233.1|/1323Bacteria_Firmicutes_Clostridia_Clostridiales_:0.32686953,Gr3_4bRCRep4no_r_no_rankgi|546651982|ref|WP_021882760.1|/1325no_rank_467_bp:0.39169087):0.08015864[0.608572],(Bact_ErysipelotNewRepAgi|496658723|ref|WP_009301216.1|/1320Bacteria_Firmicutes_Erysipelotrichia_Erys:0.72379246,(Bact_no_rankNewRepAgi|524176347|emb|CCZ45692.1|/1320Bacteria_Firmicutes/1320Firmicutes_bacterium_CAG:0.19569190,(gi|769170695|ref|WP_044942941.1|_467_bp:0.05091580,Bact_ClostridiaNewRepAgi|545388984|ref|WP_021629801.1|/1321Bacteria_Firmicutes_Clostridia_Clostridia:0.03855204):0.11942032[1.000000]):0.16839991[1.000000]):0.06523076[0.961587]):0.07369334[0.993474]):0.08325591[0.972907]):0.19033040[1.000000]):0.02439391[0.958277],(Bact_RuminococcNewRepAgi|916939772|ref|WP_051546484.1|/1338Bacteria_Firmicutes_Clostridia_Clostridia:0.64308315,(gi|1054783115|ref|WP_066546553.1|_467_bp:0.20125397,Bact_LachnospirNewRepAgi|503036515|ref|WP_013271491.1|/1353Bacteria_Firmicutes_Clostridia_Clostridia:0.20860717):0.59447384[1.000000]):0.12157165[0.999684]):0.04268938[0.927273]):0.10793730[0.999978]):0.11943808[0.999950],(Gr3_4bRCRep4no_r_no_rankgi|738376067|ref|WP_036328238.1|/1301no_rank_467_bp:0.56519925,(gi|1052781588|emb|SCH60086.1|_467_bp:0.49138780,(YP_007517186.1_467_bp:0.11285215,YP_009126903.1_467_bp:0.16756925):0.86501887[1.000000]):0.11714979[0.905074]):0.08685916[0.906027]):0.13604077[0.996757],(Bact_MicrobacteNewRepAgi|946916017|ref|WP_055838650.1|/1411Bacteria_Actinobacteria_Actinobacteria_Mi:0.63002182,(((Gr3_4bRCRep3no_r_no_rankgi|738390844|ref|WP_036342632.1|/1318no_rank_467_bp:0.70503743,(Gr3_4bRCRep3Bact_Propionibagi|488485987|ref|WP_002529618.1|/1309Bacteria_Actinobacteria_Actinobacter:0.42655245,Bact_PropionibaNewRepAgi|514978936|ref|WP_016667133.1|/1293Bacteria_Actinobacteria_Actinobacteria_Pr:0.58279535):0.12317396[0.885178]):0.31511203[1.000000],((Bact_CorynebactNewRepAgi|552777527|ref|WP_023022037.1|/1304Bacteria_Actinobacteria_Actinobacteria_Co:0.37380991,Bact_PasteurellNewRepAgi|857119895|gb|AKO38848.1|/1328Bacteria_Proteobacteria_Gammaproteobacteria_Pa:0.77610287):0.37023782[1.000000],((bRCRep3Gr3_4Bact_Bifidobactgi|547239181|ref|WP_021975256.1|/1330Bacteria_Actinobacteria_Actinobacter:0.36267728,(Bact_BifidobactNewRepAgi|919428384|ref|WP_052825216.1|/1368Bacteria_Actinobacteria_Actinobacteria_Bi:0.25650242,(Bact_BifidobactNewRepAgi|672986434|gb|KFI81686.1|/1310Bacteria_Actinobacteria_Actinobacteria_Bifidob:0.18369580,Gr3_4bRCRep3Bact_Bifidobactgi|759448466|ref|WP_043170238.1|/1343Bacteria_Actinobacteria_Actinobacter:0.11926460):0.13780437[1.000000]):0.10612307[0.999310]):0.19797746[1.000000],((Bact_BifidobactNewRepAgi|672992267|gb|KFI87454.1|/1362Bacteria_Actinobacteria_Actinobacteria_Bifidob:0.57037787,Bact_BifidobactNewRepAgi|917512920|ref|WP_052119337.1|/1380Bacteria_Actinobacteria_Actinobacteria_Bi:0.89014352):0.13025294[0.722497],(Bact_BifidobactNewRepAgi|643502474|ref|WP_025221073.1|/1397Bacteria_Actinobacteria_Actinobacteria_Bi:0.75820659,bRCRep3Gr3_4Bact_Bifidobactgi|20069877|ref|NP_613078.1|/1341Bacteria_Actinobacteria_Actinobacteria_B:0.62598444):0.20011157[0.999467]):0.04853171[0.584379]):0.08512870[0.928654]):0.07818844[0.913167]):0.05396535[0.888126],(Bact_BifidobactNewRepAgi|705399483|ref|WP_033495900.1|/1445Bacteria_Actinobacteria_Actinobacteria_Bi:1.16713334,bRCRep3Gr3_4Bact_Bifidobactgi|551236367|ref|WP_022856850.1|/1352Bacteria_Actinobacteria_Actinobacter:0.71965122):0.16585551[0.937122]):0.15441078[0.979405]):0.35312671[1.000000]):0.18441353[1.000000]):0.25884047[1.000000],(((KT149409|CRESS_unclass_1..873_467_bp:0.59949881,(KM598406|CRESS_unclass_1..816_467_bp:0.48285897,(KT149403|CRESS_unclass_1..945_467_bp:0.76378245,(((KP153422|CRESS_unclass_1..876_467_bp:0.43540526,KP153408|CRESS_unclass_1..816_467_bp:0.31992642):0.05120567[0.897048],(KM874317|CRESS_unclass_1..822_467_bp:0.42727411,(KM874304|CRESS_unclass_1..798_467_bp:0.35454465,JX904581|CRESS_unclass_1..792_467_bp:0.46097083):0.05490362[0.548364]):0.05474613[0.911739]):0.09070221[0.974188],(KM874300|CRESS_unclass_1..777_467_bp:0.68931164,(JX904407|CRESS_unclass_1..990_467_bp:0.77902241,(JX904139|CRESS_unclass_1..822_467_bp:0.25296416,(JX904075|CRESS_unclass_1..834_467_bp:0.32219089,JX904076|CRESS_unclass_1..819_467_bp:0.23875585):0.06098529[0.988974]):0.12948315[0.997163]):0.10114066[0.995550]):0.04854049[0.939004]):0.10571910[0.998293]):0.03645829[0.614954]):0.05951402[0.829216]):0.06715594[0.988912],((JX185418|CRESS_unclass_1..870_467_bp:0.45712139,KM598404|CRESS_unclass_1..885_467_bp:0.64144852):0.14386518[0.991521],(KJ641729|CRESS_unclass_1..936_467_bp:0.69356542,((KJ641722|CRESS_unclass_1..804_467_bp:0.13621059,HM228875|CRESS_unclass_1..795_467_bp:0.11526983):0.47880485[1.000000],((JN857329|CRESS_unclass_1..930_467_bp:0.35465218,KJ641718|CRESS_unclass_1..864_467_bp:0.40387159):0.13853547[0.999935],(KM972726|CRESS_unclass_1..906_467_bp:0.52584272,KF738883|CRESS_unclass_1..864_467_bp:0.60950413):0.10727461[0.973172]):0.04648219[0.707515]):0.07646715[0.979463]):0.05834337[0.619617]):0.11156048[1.000000]):0.15723644[1.000000],(((((AIF34798.1|Sewageassociated_467_bp:0.59958151,(((AKO71308.1|Banana_467_bp:0.00000034,JF957636|Nanovirus_1..861_467_bp:0.01428781):0.35066136[1.000000],(YP_003104737.1|Faba_467_bp:0.01998158,HE654123|Nanovirus_1..861_467_bp:0.05854183):0.29607855[1.000000]):0.37496789[1.000000],(((U16735|Alpha_1..858_467_bp:0.14382680,KC979052|Alpha_1..855_467_bp:0.17013936):0.24527402[1.000000],((AAA51426.1|Banana_467_bp:0.21579361,(AAA51422.1|Banana_467_bp:0.05430527,ACB86656.1|Banana_467_bp:0.24868193):0.25484386[1.000000]):0.21645604[1.000000],((HM163578|Alpha_1..945_467_bp:0.03795941,(YP_008169853.1|Cuban_467_bp:0.07290135,(YP_009246456.1|Cucurbit_467_bp:0.11713186,ALK03646.1|Alphasatellite_467_bp:0.12516190):0.07503448[1.000000]):0.03198072[0.770699]):0.66774925[1.000000],(NP_619760.1|Milk_467_bp:0.15598200,(YP_009058890.1|Faba_467_bp:0.04269750,KC978991|Alpha_1..837_467_bp:0.03976349):0.25926901[1.000000]):0.20033649[1.000000]):0.05036430[0.737717]):0.14028526[0.999994]):0.07290257[0.948784],(NP_619759.1|Milk_467_bp:0.33421736,(KF471057|Alpha_1..948_467_bp:0.43999905,JX458742|Alpha_1..924_467_bp:0.33284416):0.26785562[1.000000]):0.10259617[0.984749]):0.29595090[1.000000]):0.29223929[1.000000]):0.33227118[1.000000],(NewKX388505.1|complement_1757..3067__467_bp:0.78561105,(((YP_009237559.1|Lake_467_bp:0.65341326,YP_009021888.1|Cyanoramphus_467_bp:0.47606510):0.25597287[1.000000],AHH31482.1|Dragonfly_467_bp:0.36815571):0.12318798[0.935752],YP_009163936.1|Palaemonetes_467_bp:0.44627310):0.23625211[0.999999]):0.15092945[0.996274]):0.10659345[0.988620],((((KM874354|CRESS_unclass_1..915_467_bp:0.50578886,(KJ641738|CRESS_unclass_1..960_467_bp:0.58093736,(KR528547|CRESS_unclass_1..870_467_bp:0.57002697,KR528561|CRESS_unclass_1..885_467_bp:0.39320917):0.04928456[0.899973]):0.06538765[0.852848]):0.07501813[0.996675],(((KT945163|CRESS_unclass_1..909_467_bp:0.62549125,(KR528545|CRESS_unclass_1..909_467_bp:0.40229879,KR528553|CRESS_unclass_1..918_467_bp:0.57636273):0.06198620[0.680909]):0.10052421[0.991646],JX904231|CRESS_unclass_1..1122_467_bp:0.46361378):0.12147135[0.999945],((KR528554|CRESS_unclass_1..948_467_bp:0.28392427,KR528556|CRESS_unclass_1..915_467_bp:0.28179556):0.14509265[0.999998],(KR528551|CRESS_unclass_1..906_467_bp:0.41990369,KR528562|CRESS_unclass_1..906_467_bp:0.43712644):0.18717975[1.000000]):0.08517958[0.975818]):0.06124024[0.804268]):0.17991726[1.000000],(KJ547646|CRESS_unclass_1..891_467_bp:0.60372551,(KJ547650|CRESS_unclass_1..924_467_bp:0.51522488,KP153451|CRESS_unclass_1..951_467_bp:0.54271956):0.10743689[0.630561]):0.18565854[0.999999]):0.07654412[0.684629],(((AIY31256.1|Dromedary_467_bp:0.29829179,(AIY31243.1|Dromedary_467_bp:0.15431474,KT862224|Smacovirus_1..816_467_bp:0.30392968):0.11606940[0.991998]):0.63894383[1.000000],(AJD07511.1|Odonataassociated_467_bp:0.64148485,(YP_009252316.1|Bovine_467_bp:0.59813389,(((AJE25851.1|Human_467_bp:0.13622396,AJE25845.1|Human_467_bp:0.06150096):0.09245247[0.999983],(KY086301|Smacovirus_1..747_467_bp:0.06934677,(AJF23062.1|Human_467_bp:0.06827318,(AJF23060.1|Human_467_bp:0.00864638,(AJE25847.1|Human_467_bp:0.00864530,KP233175|Smacovirus_1..750_467_bp:0.00000043):0.15300817[1.000000]):0.06978921[0.999999]):0.13618503[1.000000]):0.10161930[0.984585]):0.43374787[1.000000],(YP_009252326.1|Bovine_467_bp:0.45301975,(KU203352|CRESS_unclass_1..810_467_bp:0.17787511,KJ547633|CRESS_unclass_1..780_467_bp:0.15508043):1.00911749[1.000000]):0.12427314[0.744577]):0.04620303[0.558269]):0.19395714[0.993345]):0.28512006[0.999877]):0.40593603[0.999996],((KM598409|Smacovirus_1..921_467_bp:0.48921908,(AEW47007.1|Circoviridae_467_bp:0.27159889,YP_009252314.1|Bovine_467_bp:0.30895835):0.26981430[0.999430]):0.88213717[1.000000],((YP_009252310.1|Sheep_467_bp:0.70688958,((KM573775|Smacovirus_1..762_467_bp:0.18482133,KM573771|Smacovirus_1..735_467_bp:0.24548539):0.21224432[1.000000],(KT862221|Smacovirus_1..783_467_bp:0.13850086,(AIY31250.1|Dromedary_467_bp:0.25786226,KT862218|Smacovirus_1..735_467_bp:0.03073767):0.12788139[1.000000]):0.20131468[1.000000]):0.14323484[0.999029]):0.09410073[0.626057],((YP_009252308.1|Sheep_467_bp:0.78635399,KY086298|Smacovirus_1..777_467_bp:0.34228292):0.04759639[0.764356],((KU043420|CRESS_unclass_1..852_467_bp:0.57479489,(KP233189|Smacovirus_1..699_467_bp:0.18947930,((YP_009030025.1|PoSCV_467_bp:0.03084155,(KJ577810|Smacovirus_1..732_467_bp:0.01143619,YP_009054985.1|Porcine_467_bp:0.11250292):0.02281072[0.949961]):0.07638946[1.000000],(YP_009022025.1|Turkey_467_bp:0.16388470,(KX838318|Smacovirus_1..819_467_bp:0.06175570,(AMR73073.1|Human_467_bp:0.05586664,KX838317|Smacovirus_1..789_467_bp:0.10728696):0.07850216[0.999995]):0.16950641[1.000000]):0.04225733[0.532744]):0.07119340[0.999989]):0.09226160[0.996600]):0.15774778[1.000000],((YP_009252320.1|Porcine_467_bp:0.49684317,(((YP_009054987.1|Porcine_467_bp:0.00000034,KJ577813|Smacovirus_1..789_467_bp:0.00391502):0.09928496[1.000000],(YP_009118276.1|Gorilla_467_bp:0.10406038,KU043428|CRESS_unclass_1..840_467_bp:0.07361623):0.07837336[0.999964]):0.21895220[1.000000],(KU043422|CRESS_unclass_1..777_467_bp:0.53231000,(KU043430|CRESS_unclass_1..867_467_bp:0.19597797,KU058671|CRESS_unclass_1..804_467_bp:0.33479535):0.20107006[1.000000]):0.09474854[0.998627]):0.06264957[0.983190]):0.06285998[0.996672],(AIY31246.1|Dromedary_467_bp:0.47924678,((YP_009163761.1|Rat_467_bp:0.28707529,YP_009054993.1|Porcine_467_bp:0.15601617):0.13103476[0.999980],(AMR73071.1|Human_467_bp:0.26497685,(KU043403|CRESS_unclass_1..819_467_bp:0.35539288,(YP_009118278.1|Lemur_467_bp:0.58114350,(ADB24799.1|Chimpanzee_467_bp:0.00000119,GQ351275|Smacovirus_1..816_467_bp:0.03985098):0.31889760[1.000000]):0.08761303[0.792114]):0.07310406[0.998594]):0.14054478[0.999999]):0.18987321[1.000000]):0.05462939[0.655276]):0.11449673[0.999998]):0.11321340[1.000000]):0.16172077[0.999821]):0.31748170[0.991844]):0.38881266[0.999889]):0.68093372[1.000000]):0.06285635[0.651983]):0.54742534[1.000000],((((FJ959082|CRESS_unclass_1..1032_467_bp:0.59102763,(KT149394|CRESS_unclass_1..846_467_bp:0.35864510,KP153360|CRESS_unclass_1..840_467_bp:0.30351069):0.45470201[1.000000]):0.03114815[0.439119],((KM821755|CRESS_unclass_1..870_467_bp:0.58055161,((JX904344|CRESS_unclass_1..819_467_bp:0.39335899,(KT732816|CRESS_unclass_1..849_467_bp:0.26709785,JF755415|CRESS_unclass_1..735_467_bp:0.19368967):0.26883435[1.000000]):0.05391962[0.981069],(JX904420|CRESS_unclass_1..840_467_bp:0.47547077,(KP153364|CRESS_unclass_1..813_467_bp:0.38863080,JX904185|CRESS_unclass_1..813_467_bp:0.30113501):0.19122864[1.000000]):0.09851126[0.999875]):0.05772861[0.999348]):0.07799617[0.999913],(KP153369|CRESS_unclass_1..882_467_bp:0.80108832,((KP153483|CRESS_unclass_1..1140_467_bp:0.73580355,(KM821764|CRESS_unclass_1..1584_467_bp:0.60611649,KP153468|CRESS_unclass_1..1167_467_bp:0.67612190):0.09467917[0.945640]):0.07306104[0.897271],(KT732823|CRESS_unclass_1..1089_467_bp:0.85506763,(KM598396|CRESS_unclass_1..1035_467_bp:0.40824407,JX185415|CRESS_unclass_1..999_467_bp:0.49503663):0.25177206[0.999958]):0.30792224[1.000000]):0.07284050[0.869903]):0.07175759[0.570737]):0.06351908[0.988602]):0.05825506[0.998932],((KP153447|CRESS_unclass_1..858_467_bp:0.42675928,((KP153485|CRESS_unclass_1..855_467_bp:0.25136157,(KJ547648|CRESS_unclass_1..840_467_bp:0.14400858,KT149412|CRESS_unclass_1..831_467_bp:0.28350711):0.06624900[0.544437]):0.44269628[1.000000],(KT149398|CRESS_unclass_1..846_467_bp:0.61638895,(KP153404|CRESS_unclass_1..903_467_bp:0.49795002,KC248416|CRESS_unclass_1..831_467_bp:0.27547187):0.10638749[0.999726]):0.04725136[0.746955]):0.17718860[1.000000]):0.08233381[0.855371],KT732819|CRESS_unclass_1..828_467_bp:0.61603309):0.09936058[0.976475]):0.05858995[0.868120],(((KM573776|CRESS_unclass_1..1008_467_bp:0.26063393,KM573767|CRESS_unclass_1..1002_467_bp:0.27991565):0.17016602[0.999996],(KU043397|CRESS_unclass_1..1200_467_bp:0.42153319,KU043406|CRESS_unclass_1..996_467_bp:0.38243026):0.14333575[0.999837]):0.31918385[1.000000],((JX904107|CRESS_unclass_1..1044_467_bp:0.47693235,JX904562|CRESS_unclass_1..888_467_bp:0.30036061):0.27769853[1.000000],(KP153377|CRESS_unclass_1..861_467_bp:0.52172873,KF738877|CRESS_unclass_1..888_467_bp:0.77521746):0.21201946[0.999987]):0.10159571[0.943943]):0.05475707[0.962004]):0.39492627[1.000000]):0.13547037[0.999884],((((KJ206566|CRESS_unclass_1..1113_467_bp:0.38917862,KU043411|CRESS_unclass_1..1104_467_bp:0.25533995):0.24775421[1.000000],(KU043424|CRESS_unclass_1..1056_467_bp:0.56062411,(KM573766|CRESS_unclass_1..1035_467_bp:0.60112606,(KT862256|CRESS_unclass_1..1206_467_bp:0.26581535,KF246569|CRESS_unclass_1..1077_467_bp:0.18234244):0.60938130[1.000000]):0.15419400[0.999777]):0.08345046[0.767815]):0.65238505[1.000000],((KM874309|CRESS_unclass_1..957_467_bp:0.68852192,KF133822|CRESS_unclass_1..873_467_bp:0.56388755):0.18689314[0.999502],((KM874347|CRESS_unclass_1..912_467_bp:0.68764428,FJ959078|CRESS_unclass_1..1230_467_bp:0.68910076):0.09508347[0.532153],(KT149404|CRESS_unclass_1..975_467_bp:0.77861439,(KP153497|CRESS_unclass_1..963_467_bp:0.92633287,(NewKX388513.1|complement_1908..3059__467_bp:0.06567298,NewKX388515.1|complement_1325..2740__467_bp:0.00000140):1.21829800[1.000000]):0.10157741[0.468288]):0.11346837[0.624577]):0.12842123[0.991736]):0.14085560[0.965704]):0.14063199[0.999940],(KT732825|CRESS_unclass_1..837_467_bp:0.85018287,((YP_009000900.1|Anguilla_467_bp:0.47777424,((YP_009091696.1|Silurus_467_bp:0.36782956,(YP_004376332.1|Barbel_467_bp:0.44453708,ADD62475.1|Human_467_bp:0.34749215):0.15731373[1.000000]):0.06393382[0.982641],(((AIF76261.1|Bat_467_bp:0.26372964,(AIF76248.1|Bat_467_bp:0.13220351,(AIF76265.1|Bat_467_bp:0.13909444,AIF76253.1|Bat_467_bp:0.15825307):0.05464364[0.959573]):0.12481181[0.999996]):0.32108355[1.000000],((AGL09969.1|Bat_467_bp:0.12414579,YP_009021891.1|Mink_467_bp:0.11314625):0.22683258[1.000000],(AKO84203.1|Fox_467_bp:0.38089828,((AIF76280.1|Bat_467_bp:0.01563482,KJ641742|CRESS_unclass_1..948_467_bp:0.00262828):0.22762611[1.000000],(YP_007974237.1|Bat_467_bp:0.17331916,AAZ78351.1|Porcine_467_bp:0.19919665):0.08130796[0.999116]):0.34854665[1.000000]):0.11616220[0.990944]):0.10533004[0.997736]):0.04331385[0.624287],(YP_009170674.1|Tadarida_467_bp:0.47536823,((ABU48445.1|Swan_467_bp:0.12104723,AHK80894.1|Duck_467_bp:0.13193741):0.40108723[1.000000],(YP_803546.1|Gull_467_bp:0.29822346,((ADU77009.1|Circovirus_467_bp:0.14328869,((YP_764455.1|Raven_467_bp:0.10009643,NP_573442.1|Canary_467_bp:0.16659145):0.03561426[0.998378],(KU230452|CRESS_unclass_1..873_467_bp:0.07753625,YP_009134739.1|Zebra_467_bp:0.14305537):0.02390863[0.995873]):0.06232397[0.999972]):0.12525765[1.000000],(AFL02442.1|Beak_467_bp:0.34324273,AEL28794.1|Bat_467_bp:0.32803155):0.05461942[0.631129]):0.04429702[0.610785]):0.18768046[1.000000]):0.08944342[0.830728]):0.09777381[0.998696]):0.10672063[0.999993]):0.08707161[0.999079]):0.08307860[0.999757],(AIF76251.1|Bat_467_bp:0.72678576,(((AMH87650.1|Pacific_467_bp:0.07984214,AMH87652.1|Pacific_467_bp:0.18412850):0.65568576[1.000000],((ADU76993.1|Cyclovirus_467_bp:0.17042322,(ADD62477.1|Cyclovirus_467_bp:0.11249807,(YP_009047065.1|Cyclovirus_467_bp:0.15671531,YP_008130363.1|Human_467_bp:0.15964424):0.05975373[0.887971]):0.05748079[0.977261]):0.29898194[1.000000],(AIF76254.1|Bat_467_bp:0.29200914,(AGJ74756.1|Dragonfly_467_bp:0.23243724,((ADD62457.1|Cyclovirus_467_bp:0.21405208,(AEL87792.1|Bat_467_bp:0.20030644,(AIF76266.1|Bat_467_bp:0.20594405,ADD62473.1|Cyclovirus_467_bp:0.08624280):0.09304943[1.000000]):0.08043182[0.999998]):0.03274192[0.970150],(YP_009110680.1|Bat_467_bp:0.17482002,((ADY17982.1|Dragonfly_467_bp:0.27899934,(AGJ74760.1|Dragonfly_467_bp:0.17895004,(YP_009021870.1|Human_467_bp:0.23297506,AFS65290.1|Dragonfly_467_bp:0.25857866):0.08870476[0.999998]):0.03203164[0.893708]):0.05876131[0.999995],(((ADD62451.1|Cyclovirus_467_bp:0.13040349,ADD62455.1|Cyclovirus_467_bp:0.15441526):0.04227078[0.987650],(ADD62461.1|Cyclovirus_467_bp:0.22249944,(ADU77011.1|Cyclovirus_467_bp:0.14255823,AGJ74758.1|Dragonfly_467_bp:0.20825192):0.05149457[0.991062]):0.04248201[0.513803]):0.07478035[1.000000],((((ADD62471.1|Cyclovirus_467_bp:0.13359018,AIF76252.1|Bat_467_bp:0.11345358):0.08859943[1.000000],(YP_004152331.1|Cyclovirus_467_bp:0.17468257,(AIF76249.1|Bat_467_bp:0.26066963,ADD62453.1|Cyclovirus_467_bp:0.22402371):0.06123793[0.985250]):0.05134865[0.952694]):0.03129844[0.981828],(AEL87790.1|Bat_467_bp:0.17272370,(AEL87786.1|Bat_467_bp:0.19715645,ADI48251.1|Bat_467_bp:0.18168823):0.06216230[0.999983]):0.05211474[0.995771]):0.05418573[0.997869],(AKE49355.1|Cyclovirus_467_bp:0.17290689,YP_009021843.1|Dragonfly_467_bp:0.26068621):0.05790845[0.998244]):0.04100104[0.997866]):0.02049593[0.827618]):0.04177314[0.955954]):0.02867108[0.876503]):0.05411445[0.999283]):0.05854171[0.933416]):0.17142844[1.000000]):0.12329120[0.999959]):0.04907581[0.462508],(YP_009237526.1|Lake_467_bp:0.50390894,YP_009116910.1|Sewageassociated_467_bp:0.58503273):0.11812667[0.996101]):0.05259581[0.923096]):0.10712000[0.992970]):0.12595804[0.998438]):0.17731648[0.999998]):0.11847095[1.000000]):0.03601170[0.590421]):0.20648811[1.000000]):0.20905036[0.999421]):0.24093506[0.999705]):0.11482824[0.897375]):0.76539773[1.000000]):0.15661371[0.999333]):0.11111401[0.998736]):0.09319462[0.771385]):0.05267335[0.621678]):0.47407169[1.000000])OROOT;
