# Supplementary material for: Multiple origins of prokaryotic and eukaryotic single-stranded DNA viruses from bacterial and archaeal plasmids
Source: Nat Commun. 2019 Jul 31;10:3425. doi: 10.1038/s41467-019-11433-0 (PMC6668415; doi:10.1038/s41467-019-11433-0)
Supplement: Supplementary file 13 — Description of Additional Supplementary Files [file 41467_2019_11433_MOESM13_ESM.pdf]

Title: Supplementary Data 1.

- Composition of the 2 superclusters and 2 orphan clusters of HUH endonucleases;
- Accession numbers for the HUH endonucleases depicted in the network shown in Figure 1;
- Composition of the 9 groups of bacterial viral-like Reps;
- Genomic neighborhoods of the integrated plasmids.

Title: Supplementary Data 2.

- Sequence alignment used to generate the tree shown in Figure 2A.

Title: Supplementary Data 3.

- Sequence alignment used to generate the tree shown in Figure 5.

Title: Supplementary Data 4.

- Maximum likelihood tree shown in Figure 2A in newick format.

Title: Supplementary Data 5.

- Maximum likelihood tree shown in Figure 5 in newick format.

Title: Supplementary Data 6.

- Constrained tree shown in Supplementary figure S5A in newick format.

Title: Supplementary Data 7.

- Constrained tree shown in Supplementary figure S5B in newick format.

Title: Supplementary Data 8.

- Constrained tree shown in Supplementary figure S5C in newick format.

Title: Supplementary Data 9.

- Maximum likelihood tree shown in Supplementary figure S6A in newick format.

Title: Supplementary Data 10.

- Maximum likelihood tree shown in Supplementary figure S6B in newick format.

Fondation reconnue
d'utilité publique
habilitée à recevoir
dons et legs

25 rue du Docteur Roux, 75015 Paris
Téléphone : +33 (0) 1 40 61 37 22
Télécopie : 33 (0) 1 45 68 88 34
E-mail : krupovic@pasteur.fr
